# Genome‐wide screen and functional analysis in *Xanthomonas* reveal a large number of mRNA‐derived sRNAs, including the novel RsmA‐sequester RsmU

**DOI:** 10.1111/mpp.12997

**Published:** 2020-09-23

**Authors:** Dong‐Jie Tang, Xiao‐Lin Chen, Yu Jia, Yu‐Wei Liang, Yuan‐Ping He, Ting‐Ting Lu, Chuan‐Rang Zhu, Bin Han, Shi‐Qi An, Ji‐Liang Tang

**Affiliations:** ^1^ State Key Laboratory for Conservation and Utilization of Subtropical Agro‐bioresources College of Life Science and Technology Guangxi University Nanning China; ^2^ National Center for Gene Research & Institute of Plant Physiology and Ecology Shanghai Institutes of Biological Sciences Chinese Academy of Sciences Shanghai China; ^3^ National Biofilms Innovation Centre Biological Sciences University of Southampton Southampton UK; ^4^ Present address: Plant Protection Research Institute Guangxi Academy of Agricultural Science 174 Daxue Road Nanning Guangxi 530007 China

**Keywords:** RsmA, RsmU, small noncoding RNA, sRNA, virulence, *Xanthomonas*

## Abstract

Although bacterial small noncoding RNAs (sRNAs) are known to play a critical role in various cellular processes, including pathogenesis, the identity and action of such sRNAs are still poorly understood in many organisms. Here we have performed a genome‐wide screen and functional analysis of the sRNAs in *Xanthomonas campestris* pv. *campestris* (Xcc), an important phytopathogen. The 50–500‐nt RNA fragments isolated from the wild‐type strain grown in a virulence gene‐inducing condition were sequenced and a total of 612 sRNA candidates (SRCs) were identified. The majority (82%) of the SRCs were derived from mRNA, rather than specific sRNA genes. A representative panel of 121 SRCs were analysed by northern blotting; 117 SRCs were detected, supporting the contention that the overwhelming majority of the 612 SRCs identified are indeed sRNAs. Phenotypic analysis of strains overexpressing different candidates showed that a particular sRNA, RsmU, acts as a negative regulator of virulence, the hypersensitive response, and cell motility in Xcc. In vitro electrophoretic mobility shift assay and in vivo coimmunoprecipitation analyses indicated that RsmU interacted with the global posttranscriptional regulator RsmA, although sequence analysis displayed that RsmU is not a member of the sRNAs families known to antagonize RsmA. Northern blotting analyses demonstrated that RsmU has two isoforms that are processed from the 3′‐untranslated region of the mRNA of *XC1332* predicted to encode ComEA, a periplasmic protein required for DNA uptake in bacteria. This work uncovers an unexpected major sRNA biogenesis strategy in bacteria and a hidden layer of sRNA‐mediated virulence regulation in Xcc.

## Graphical abstract

The RsmA‐sequestering noncoding RNA RsmU derived from the 3′‐UTR of *XC1332* mRNA negatively regulates virulence, the hypersensitive response, and cell motility in *Xanthomonas campestris*.

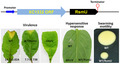

## 1 INTRODUCTION

A successful infection and colonization of a host by pathogenic bacteria requires a rapid and efficient adaptation to the specific host niches via coordinating the expression of bacterial virulence factors and virulence‐associated behaviours (Quereda and Cossart, 2017). Complex regulatory networks act to sense the host environment and appropriately modulate virulence factor production. Expression of genes in a virulence regulation network is tightly controlled at the transcriptional, posttranscriptional, translational, and posttranslational levels in response to specific stimuli (Quereda and Cossart, 2017). Such control can involve the action of bacterial small noncoding RNAs (sRNAs) (Ha and Kim, 2014; Borges and Martienssen, 2015; Nitzan *et al*., 2017) as well as regulatory proteins (Ribet and Cossart, 2015; Quereda and Cossart, 2017).

Bacterial sRNAs are usually between 50 and 400 nucleotides (nt) in length (Quereda and Cossart, 2017). Most bacterial sRNAs are transcribed from sRNA genes located in the intergenic region between open reading frames (ORFs) or within the coding region of ORFs, but a few sRNAs known to be generated by processing from longer transcripts such as mRNAs and riboswitches (Vogel *et al*., 2003; Kawano *et al*., 2005; Loh *et al*., 2009; Chao *et al*., 2012; Chao and Vogel, 2016). sRNA genes do not have an ORF structure, thus cannot be predicted in standard genome annotation. Over the last decade, several experimental and computational approaches have been developed to identify sRNAs, of which the high‐throughput cDNA library sequencing approach (i.e., RNA‐Seq) is the most powerful (Sharma and Vogel, 2009; Liu and Camilli, 2011; Barquist and Vogel, 2015). A large number of sRNAs have been identified from human and animal bacterial pathogens such as *Listeria monocytogenes* (Toledo‐Arana *et al*., 2009), *Staphylococcus aureus* (Lasa *et al*., 2011), *Salmonella enterica* serovar Typhimurium (Westermann *et al*., 2016), *Vibrio cholerae* (Mandlik *et al*., 2011), *Helicobacter pylori* (Sharma *et al*., 2010), *Chlamydia trachomatis* (Albrecht *et al*., 2010), and *Pseudomonas aeruginosa* (Wurtzel *et al*., 2012).

It is known that most bacterial sRNAs act as posttranscriptional regulators, controlling gene expression by pairing with their mRNA targets to modulate their translation and/or stability; only a few sRNAs function by binding to proteins to modify their activity (Hör *et al*., 2018). Perhaps the best‐known bacterial protein‐binding sRNA is RsmB/CsrB, which regulates gene expression through controlling the activity of the global posttranscriptional regulator protein RsmA/CsrA (Romeo and Babitzke, 2018). The cellular processes regulated by sRNAs are manifold, including primary and secondary metabolism (Bobrovskyy and Vanderpool, 2013), iron homeostasis (Oglesby‐Sherrouse and Murphy, 2013), envelope stress response (Klein and Raina, 2017), quorum sensing (Bejerano‐Sagie and Xavier, 2007), and pathogenesis (Quereda and Cossart, 2017). A body of work has shown that sRNAs play a central role in the virulence regulation of many human and animal bacterial pathogens (Gripenland *et al*., 2010; Li *et al*., 2013; Quereda and Cossart 2017). In contrast, the expression profile and function of sRNAs in plant pathogenic bacteria are relatively poorly understood, with only a few virulence‐related sRNAs identified from a limited number of organisms (Abendroth *et al*., 2014; Dequivre *et al*., 2015; Kwenda *et al*., 2016).

*Xanthomonas campestris* pv. *campestris* (Xcc) is the causal agent of black rot disease of cruciferous crops (Mansfield *et al*., 2012; Vicente and Holub, 2013) and is a member of a genus of phytopathogenic bacteria that infect many economically important crops causing severe disease and economic losses (Ryan *et al*., 2011; An *et al*., 2019). Work in the last decade has identified three virulence‐related sRNAs amongst a number of other sRNAs in different *Xanthomonas* species (Jiang *et al*., 2010; Liang *et al*., 2011; Schmidtke *et al*., 2012, 2013; Hu *et al*., 2018). Nevertheless, the inventory of sRNAs in *Xanthomonas* is probably far from complete. Here we have used RNA‐Seq and sRNA overexpression approaches to perform a genome‐wide screening and functional analysis of sRNAs in Xcc. This work has identified a large number of sRNAs, including many that are derived from mRNA as well as a novel RsmA‐sequestering sRNA. Our finding uncovers an unexpected major sRNA biogenesis pathway in bacteria and a hidden sRNA‐mediated virulence regulation pathway in Xcc.

## 2 RESULTS

### 2.1 Identification of sRNA candidates in Xcc by RNA‐Seq

The main purpose of this study was to identify the sRNAs expressed by Xcc grown in the minimal medium MMX, a condition mimicking the environment of plant tissues (Daniels *et al*., 1984a; Tang *et al*., 2006). To optimize sRNA identification, two different types of sRNA libraries were generated using RNAs with sizes ranging from 50 to 200 nt and from 200 to 500 nt. The RNAs were isolated from the Xcc wild‐type strain 8,004 (Table S1) grown to mid‐log phase in MMX. To increase the opportunity to capture weakly expressed sRNAs, the RNA samples were treated with an rRNA removal kit to reduce the abundant rRNA (5S, 16S, and 23S rRNA) molecules prior to the construction of the libraries, which were subjected to ultradeep sequencing using the Illumina/Solexa sequencing platform as described previously (Jovanka *et al*., 2011; Barquist and Vogel, 2015). After sequencing, the raw reads obtained from the two libraries were mixed together to generate a data set for further analysis. After clipping, linker removal, and quality control, the clean sequencing reads were mapped to the genome of Xcc 8,004 (Qian *et al*., 2005) using the SOAP2 program (Li *et al*., 2009), allowing mismatch of fewer than 2 nt. Reads that did not fulfil this requirement were removed from the data set. In total, 37.3 million (652‐fold genome coverage) mappable reads were obtained (Table S2). Target transcripts (TTs) and sRNA candidates (SRCs) were identified from the RNA‐Seq data first by visualized mapping of RNA‐Seq reads to the Xcc 8,004 genome and then by computational analysis plus manual inspection of the visualized mapping data (described in section 4). A total of 676 TTs (TT001–TT676) were determined (Figure S1 and Table S3). Nearly all known Xcc sRNAs, such as the housekeeping structural RNAs (tmRNA, rnpB, 6S RNA, tRNAs) and the previously identified sRNAs (sRNA‐Xcc1 to sRNA‐Xcc3) (Jiang *et al*., 2010), were present in the identified TTs (Figure S1 and Table S3), suggesting that the sRNA screening in this work is efficient. After removal of the known sRNAs and small ORF mRNAs, the remaining 612 TTs were taken as SRCs (SRC001–SRC612) (Figure S1 and Table S4).

**FIGURE 1:**
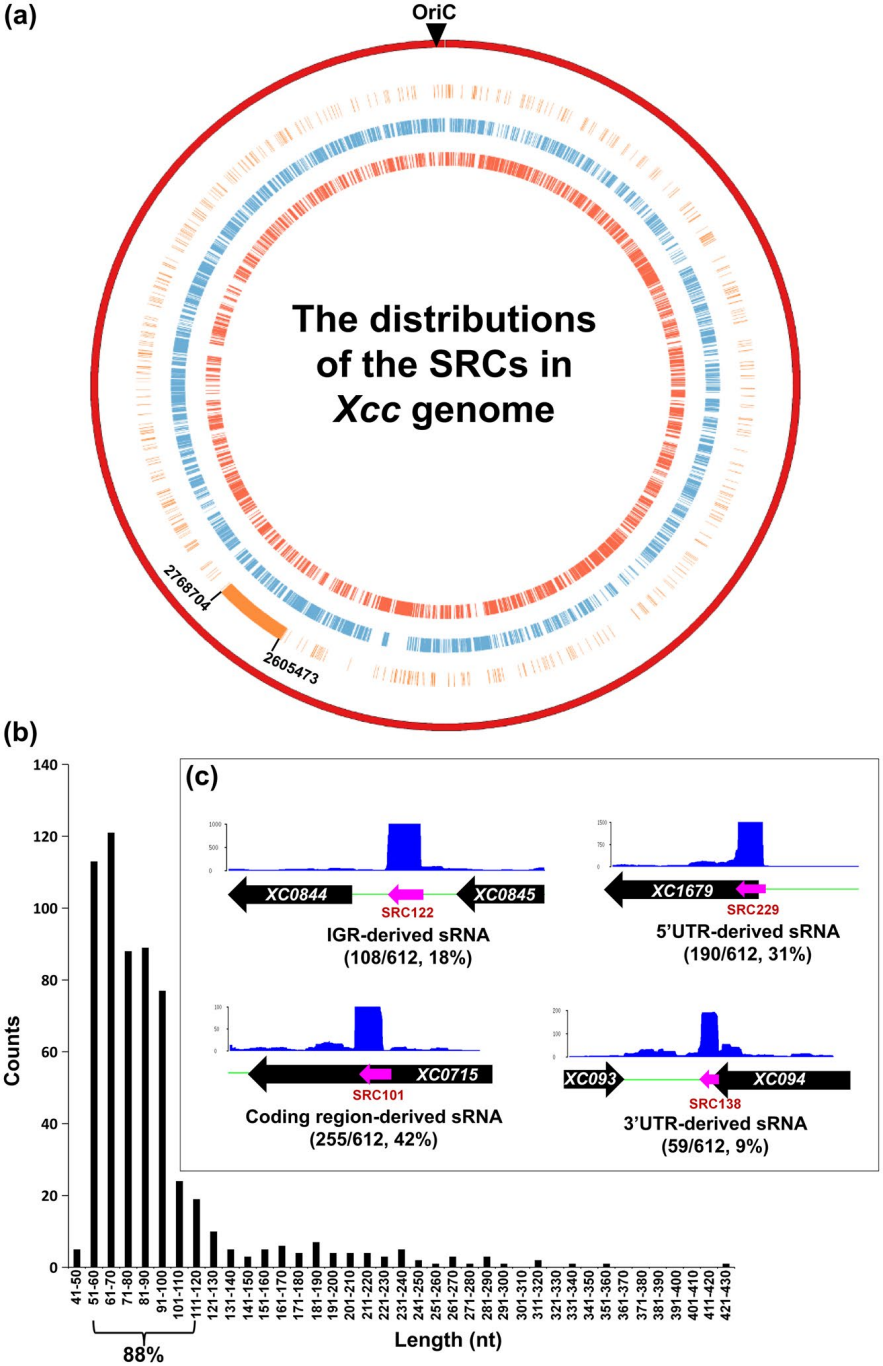
The sRNome of *Xanthomonas campestris* pv. *campestris* (Xcc) strain 8,004 at mid‐log phase in minimal medium. (a) Circular representation of the distributions of the 612 small noncoding RNA (sRNA) candidates (SRCs) on Xcc strain 8,004 genome. The circles show the following information from outside to inside: (1) the genome of strain 8,004 (red); (2) SRCs (orange); (3) protein‐coding genes on the minus strand (blue); (4) protein‐coding genes on the plus strand (orange red). (b) Length distribution of the 612 SRCs. (c) Classification of the 612 SRCs according to their genomic locations: IGR‐derived SRCs means the SRC is derived from the intergenic region, 5′‐UTR‐, 3′‐UTR‐, and coding region‐derived SRCs means the SRC is derived from the 5′‐untranslated, 3′‐untranslated, and the protein‐coding region of an mRNA, respectively. A representative of a typical visualized mapping pattern of the four classes of SRCs is shown. The black arrows indicate the protein‐coding gene and its transcriptional orientation and the gene ID is shown inside the arrow. The pink arrows indicate an SRC and its direction. The *y* axis is the number of mapped reads

The SRCs were randomly scattered on the genome, no sRNA cluster or island was found, although the SRC density in the 2,605,473–2,768,704 region was much higher than in other regions (Figure 1a). The average length of the SRCs was 91 nt, the longest one was 423 nt (SRC064), and the shortest one was 44 nt (SRC411), and 88% of SRCs were between 51 and 120 nt (Figure 1b). Sequence comparison among the SRCs revealed that SRC017/SRC434/SRC565 and SRC020/SRC291 were homologous sRNAs (Figure S2); no sequence similarity was found among the other SRCs. Seven riboswitches, a TPP (RF0059), a cobalamin (RF00174), an SAM (RF00162), a glycine (RF00504), an SAH (FR01057), an FMN (FR0050), and a yybP‐ykoY (FR0080) were identified (Table S5) by searching the Rfam database v. 13.0 (http://rfam.xfam.org/search?tab=searchSequenceBlock#tabview=tab) (Kalvari *et al*., 2018). Of the SRCs, 108 (18%) were derived from the intergenic region (IGR), 504 (82%) were derived from protein‐coding transcripts (255 [42%] from the coding region, 190 [31%] from the 5′‐untranslated region [5′‐UTR], and 59 [9%] from the 3′‐UTR of an mRNA) (Figure 1c and Table S4).

### 2.2 117 out of 121 SRCs are expressed sRNAs

**FIGURE 2:**
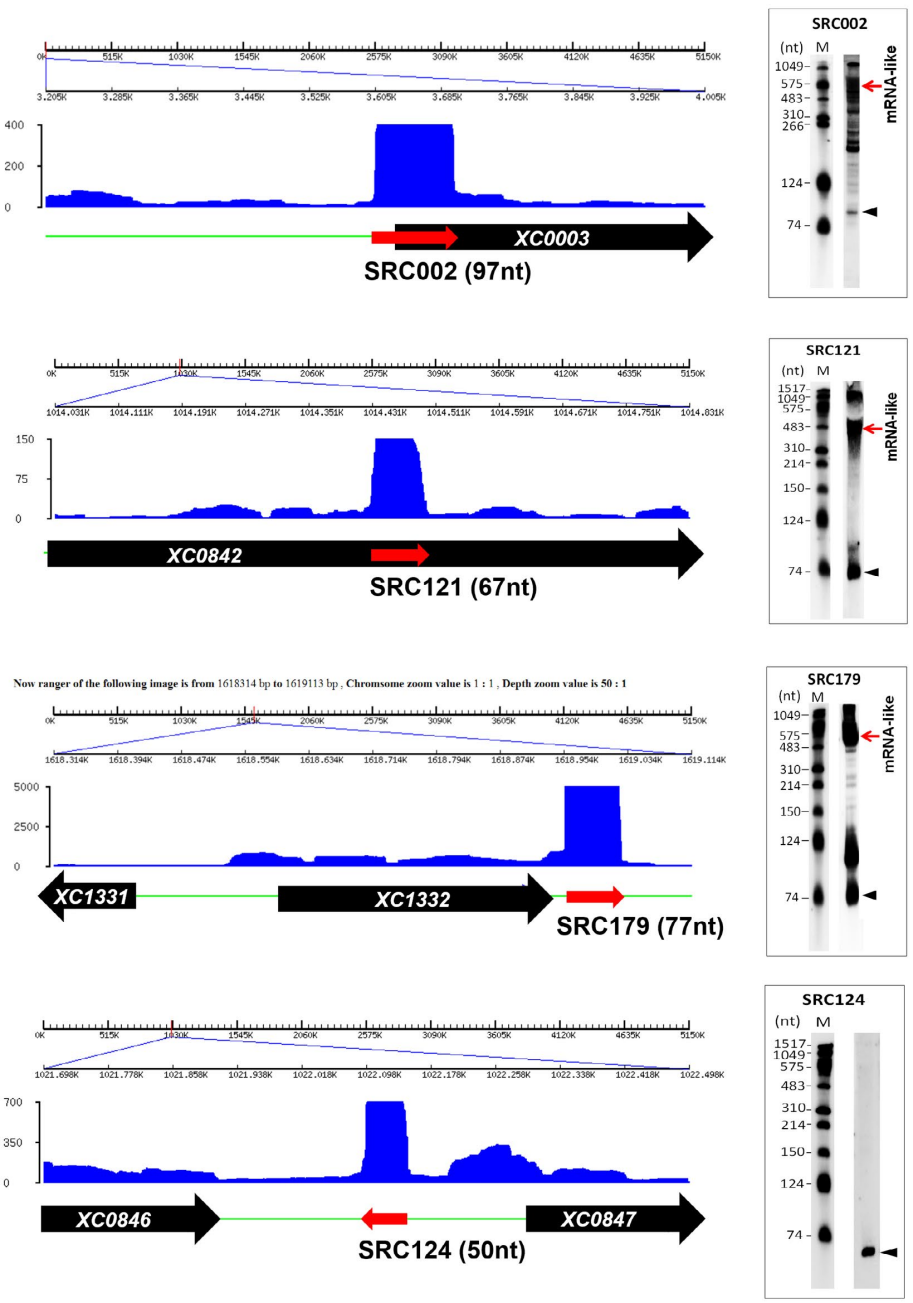
Detection of the expression of *Xanthomonas campestris* pv. *campestris* (Xcc) small noncoding RNAs (sRNAs) by northern blotting. Bacterial cells of Xcc strain 8,004 were cultured in MMX medium to mid‐log phase and total RNAs were isolated from the cells. Three micrograms of the total RNAs were used for northern blotting. To ensure the accuracy in evaluation of the size of signal bands, DIG‐labelled RNA molecular weight marker (M) was used. The picture shows a few representatives of the northern blotting results and all northern blotting results are shown in Figure S3. The filled triangle inside the northern blotting result picture indicates the signal band corresponding to the size of the target sRNA candidate (SRC) predicted by RNA‐Seq. On the left‐hand side of the northern blotting result picture is the visualized mapping pattern of the corresponding SRC. The black arrows indicate the protein‐coding gene and its transcriptional orientation, and the gene ID is shown inside the arrow. The red arrows indicate the SRC and its transcriptional direction. The *y* axis is the number of the mapped reads

Northern blot analysis was used to detect the expression of individual SRCs. A total of 121 SRCs (including representatives of high‐, middle‐, and low‐abundance SRCs) (Table S6) were selected for northern blotting analysis, using digoxigenin (DIG)‐labelled RNA probes. Because the transcriptional orientation of the SRCs is unclear (because we used the nonstrand‐specific RNA‐Seq method), a forward probe and a reverse probe were used to detect the expression of a given SRC (Table S7). As a result, of the 121 SRCs under study, the expression of 117 sRNAs (sRX001–sRX117; sRX: sRNA of Xcc) were detectable in the tested conditions (Figure S3 and Table S6). Some representative northern blot results are shown in Figure 2. It should be noted that, in most cases, more than one signal band was detected in the northern blotting. In addition to the signal band whose size is in agreement with the length of predicted SRC, one or more other signal bands (longer and/or shorter) were also detected (Figures 2 and S3). In a few cases (such as SRC002, SRC018, SRC089, and SRC098), more than 10 signal bands were detected (Figures 2 and S3). Unexpectedly, the abundance of the signal bands corresponding to the predicted SRC was not always the highest among the detected bands (Figures 2 and S3), indicating that some higher‐abundance RNA species were excluded during cDNA library construction, possibly due to bias in the step of reverse transcription using the random primers. It is worth noting that, in most cases, an mRNA‐like transcript could also be detected using an sRNA‐specific probe (Figures 2 and S3), suggesting that the sRNA overlapped in sense with the mRNA. It is very likely that these sRNAs are the processing products of the mRNAs. Secondary structure prediction showed that all confirmed Xcc sRNAs are predicted to fold into highly stable RNA structures (Figure S4).

### 2.3 sRX061 has two isoforms and plays an important role in virulence, hypersensitive response, and swarming motility

An overexpression approach was used to identify any potential virulence‐related sRNAs from the sRNAs confirmed by northern blotting. sRNA overexpression strains of Xcc were constructed by cloning the entire gene of interest for the sRNA into the broad host range expression vector pBBad (Sukchawalit *et al*., 1999), and then transferring the recombinant plasmid into the wild‐type strain 8,004 to generate the sRNA overexpression strain WT/pBBad(sRNA). This led to the transcription of the target sRNA being driven by the P*_BAD_* promoter, which expressed at a very high level in Xcc in the absence of the inducer arabinose (Figure S5). This characteristic of P*_BAD_* can ensure the overexpression of the target sRNA in the absence of arabinose. Furthermore, to ensure the accurate processing of the primary transcripts of the target sRNA, the native rho‐independent terminator of the target sRNA (if it has a predicted one) was also included in the sequence cloned.

In total, overexpression strains of 44 northern blot‐confirmed sRNAs were constructed in this work (Table S1). Semiquantitative reverse transcription (RT)‐PCR analysis revealed that the expression level of these target sRNAs in the overexpression strains was more than 5‐fold higher compared to the wild‐type strain carrying the vector pBBad alone when grown in MMX without addition of arabinose (Figure S5). To determine the effect of sRNA overexpression on the virulence of Xcc, the virulence of these overexpression strains was tested in the host plant Chinese radish (*Raphanus sativus* var. *radiculus*) and compared to that of the pBBad‐carrying control strain. The result showed that all sRNA overexpression strains caused a wild‐type virulence phenotype with the exception of WT/pB061 (the sRX061 [SRC179] overexpression strain), which displayed a significant reduction in virulence (Figures S6 and 3a). Semiquantitative RT‐PCR analysis revealed that the expression level of sRX061 in the overexpression strain was c.90‐fold higher than that in the pBBad‐carrying control strain (Figure S5). These results indicate that sRX061 plays an important role in the virulence of Xcc.

**FIGURE 3:**
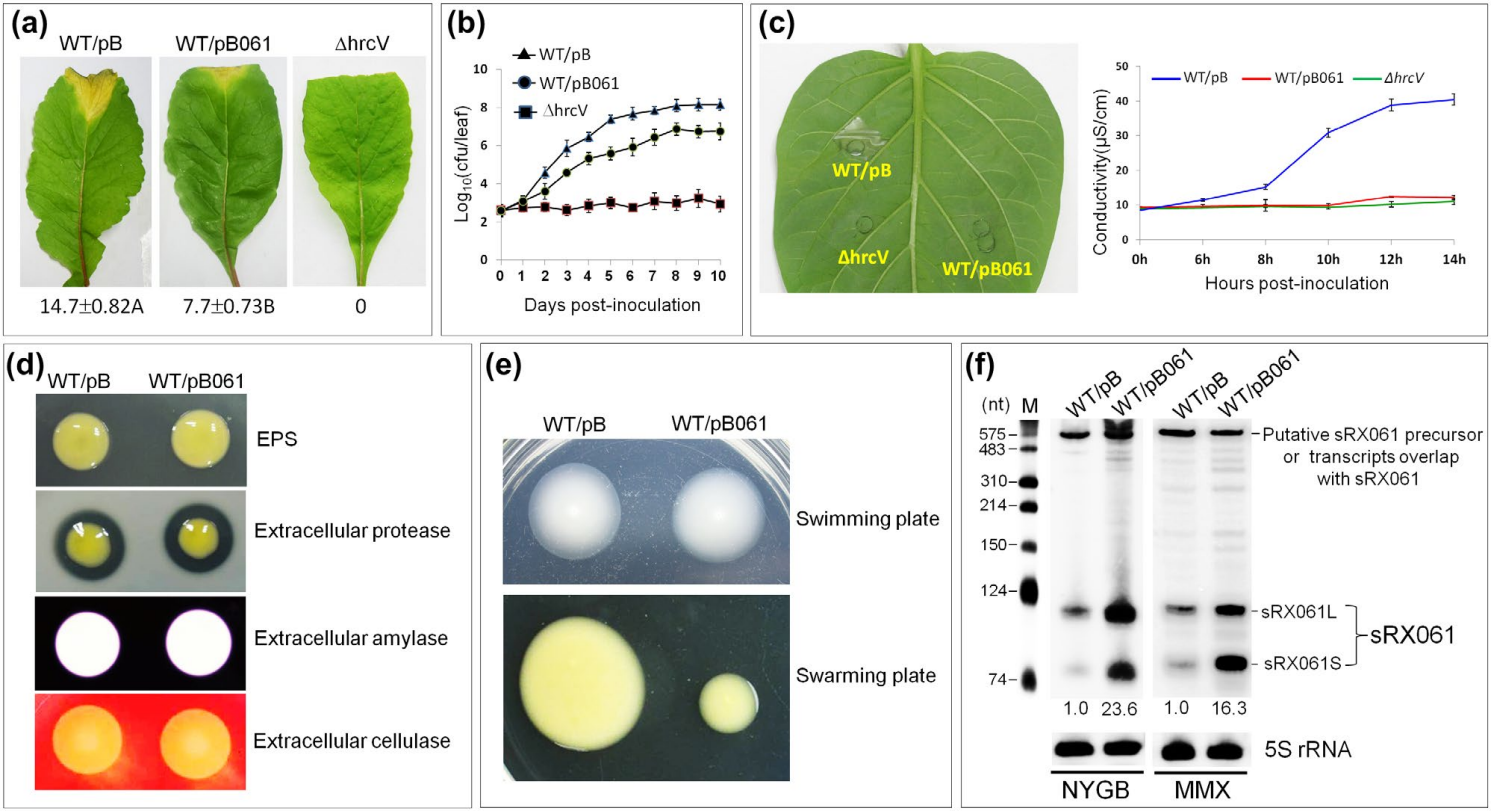
Effects of overexpressed sRX061 (SRC179) on virulence, in planta growth, hypersensitive response (HR), virulence factor production, and cell motility of *Xanthomonas campestris* pv. *campestris* (Xcc). (a) Black rot symptoms and the average lesion lengths (in mm) caused by the pBBad‐carrying wild‐type strain WT/pB and the sRX061 overexpression strain WT/pB061 on inoculated leaves of Chinese radish. Images were taken at day 10 postinoculation. Values under each leaf are the average lesion lengths (mean ± *SD*) from three repeats, each with 50 leaves. The nonpathogenic Xcc strain ΔhrcV was used as a negative control. Different letters after the values of the average lesion lengths indicate significant differences (*t* test, *p* < .01). (b) The growth rates of the strains WT/pB and WT/pB061 in inoculated leaves of Chinese radish. Bacterial cells were recovered from the inoculated leaves every day in a period of 10 days postinoculation. Data are the mean ± *SD* from a representative experiment, and similar results were obtained in two other independent experiments. The nonpathogenic Xcc strain ΔhrcV was used as a negative control. (c) The left part is the HR induced on nonhost plant pepper leaves (*Capsicum annuum* 'ECW‐10R') by Xcc strains. The photograph was taken 24 hr after inoculation. The right part is the electrolyte leakage induced by Xcc strains in the nonhost plant pepper leaf tissues. The result presented is from a representative experiment and similar results were obtained in two other independent experiments. The type III‐deficient mutant strain ΔhrcV was used as a negative control. (d) Detection of extracellular polysaccharide (EPS) production and the activity of extracellular enzymes. Zones of clearance around the spot are due to the degradation of the substrate by enzyme activity. Three plates were inoculated in each experiment. The experiment was repeated three times and similar results were obtained. The relative EPS yield was evaluated by the size of colonies and the relative activity of an enzyme was indicated by the diameter of the clear zone. (e) Swarming motility and swimming motility assay. The strains WT/pB and WT/pB061 were examined on the swarming plate and swimming plate. Two microlitres of overnight culture (OD_600_ = 1.0) of each strain was spotted onto the swarming assay plate and swimming assay plate, and results were observed and photographed after incubation at 28 °C for 3 days. (f) Detection of sRX061 levels in the strains WT/pB and WT/pB061 by northern blotting. Strains were cultured in MMX medium or NYG medium at 28 °C with shaking at 200 rpm for 24 hr. Total RNAs were isolated and 3 μg was used for northern blotting with a digoxigenin‐labelled sRX061 probe. 5S rRNA was probed as a loading control

To test whether the reduced virulence of the sRX061 overexpression strain was correlated to a reduced growth rate of the pathogen in the host, we investigated the growth of the sRX061 overexpression strain in planta. The result showed that the total bacterial number of the sRX061 overexpression strain in the infected leaves was approximately 10‐fold lower than that of the pBBad‐carrying control strain at each of the tested time points from 2 to 10 days postinoculation (dpi) (Figure 3b), although it grew comparably to the pBBad‐carrying control strain in rich and minimal media (Figure S7).

To determine whether overexpression of sRX061 has any effect on the ability of Xcc to trigger the hypersensitive response (HR), the sRX061 overexpression strain was inoculated into leaves of pepper cultivar ECW‐10R, a nonhost plant typically used to test the HR of Xcc (Castañeda *et al*., 2005). The results showed that although the pBBad‐carrying control strain could induce a typical HR, the sRX061 overexpression strain was not able to do this (Figure 3c). This observation was supported by quantitative assays of electrolyte leakage as a measure of the loss of cell integrity (Figure 3c). These results reveal that sRX061 is a negative regulator of HR in Xcc.

These findings prompted an examination of the effects of sRX061 overexpression on the production of specific factors and traits associated with virulence. Extracellular polysaccharide (EPS) and extracellular enzymes including protease, endoglucanase, and amylase are known to contribute to the virulence of Xcc (Ryan *et al*., 2011). When the production of EPS and extracellular enzymes by the overexpression strain were compared to that of the pBBad‐carrying control strain, no significant differences were observed (Figure 3d). This indicates that sRX061 is not involved in the regulation of the production of these virulence factors. The effects of sRX061 overexpression on the cell motility of Xcc was also examined. The sRX061 overexpression strain displayed a severe swarming motility defect (Figure 3e), although there was no apparent effect on swimming motility (Figure 3e).

As shown in Figure 2, northern blotting using an sRX061 (SRC179) probe detected three major bands (c.600, c.120, and c.80 nt). To determine whether all of the three transcripts were overexpressed in the sRX061 overexpression strain, northern blotting was performed to detect the transcripts and their level in the sRX061 overexpression strain in comparison to that in the pBBad‐carrying control strain. The results showed that, in accordance with the findings shown in Figure 2, the sRX061 probe could detect all three transcripts in both strains (Figure 3f). However, in the sRX061 overexpression strain, the c.80‐ and c.120‐nt transcripts but not the c.600‐nt transcript were overexpressed (Figure 3f), revealing that expression of the cloned *sRX061* gene (with the rho‐independent terminator coding sequence) can generate both a c.∼80‐ and a c.120‐nt transcript. For simplification, we named the c.80‐nt transcript sRX061S and the c.120‐nt transcript sRX061L. sRX061S corresponds to the 77‐nt SRC179 (Table S4). Hereafter, we redefine sRX061 as an Xcc sRNA that has two isoforms: sRX061S and sRX061L.

To determine whether sRX061L and sRX061S have independent functions, we tried to construct a strain in which only sRX061L or sRX061S was overexpressed. However, the strain harbouring pBBad carrying the sRX061L‐coding sequence always overexpressed both sRX061L and sRX061S, while no overexpression was observed in the strain that harbours pBBad carrying the sRX061S‐coding sequence (Figure S8). It seems that sRX061L is essential for the production of sRX061S. As a consequence, it remains unclear whether sRX061L and sRX061S have independent functions.

### 2.4 sRX061S and sRX061L are processed from the 3′‐UTR of *comEA* (*XC1332*) mRNA

**FIGURE 4:**
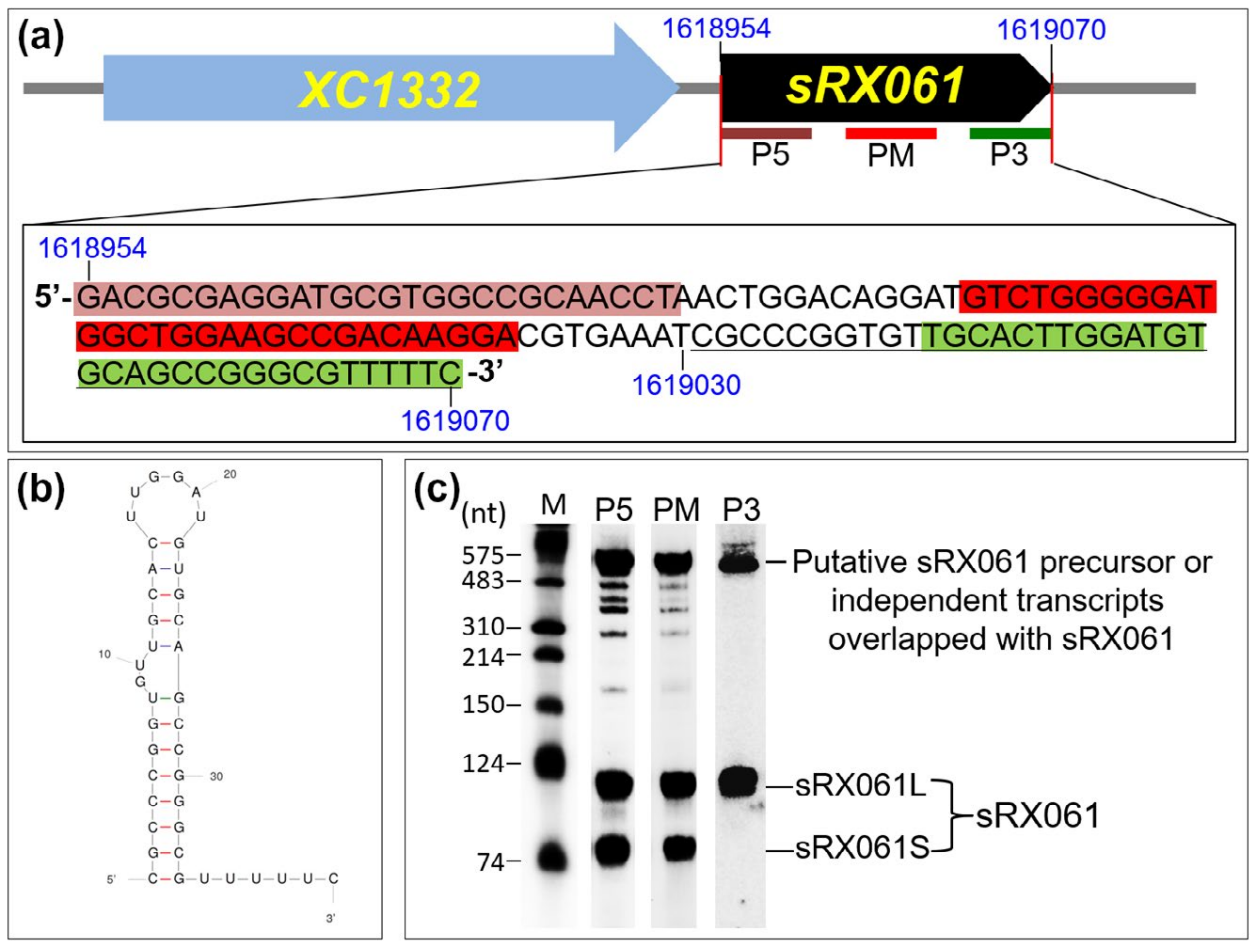
Detection of sRX061 by northern blotting using short probes complementary to different parts of sRX061. (a) The position and sequence of the 30‐nt probes complementary to the 5′ region (P5), middle region (PM), and 3′ region (P3) of sRX061. Probes are shown as thick coloured lines, and their sequences are highlighted with a coloured shadow. (b) A typical rho‐independent terminator present at the 3′‐end of sRX061. The sRX061 terminator sequence is underlined in (a). (c) sRX061 transcripts detected by northern blotting using the P5, PM, and P3 probes. The wild‐type *Xanthomonas campestris* pv. *campestris* strain 8,004 was cultured in MMX medium to mid‐log phase and total RNAs were isolated. Three micrograms of the RNAs were used for northern blotting

Genome mapping results revealed that the coding sequence for the 77‐nt sRX061S is from nucleotide position 1,618,954 to 1,619,030 in the genome of Xcc strain 8,004, and the coding sequence for sRX061L was predicted to be from nucleotide position 1,618,954 to 1,619,070 (Figure 4a). Terminator prediction revealed that a typical rho‐independent terminator (Figure 4b) is present between nucleotide positions 1,619,031 and 1,619,070; this corresponds to positions 78 to 117 of sRX061L and is absent in sRX061S. It is possible that sRX061S is generated from sRX061L by removal of the rho‐independent terminator. To test this, three short probes specific for the 5′ (P5), 3′ (P3), and middle region (PM) of *sRX061* (Figure 4a) were generated and used for northern blotting analysis to detect the sRX061L and sRX061S transcripts. The results showed that sRX061L was detectable by all three probes, while sRX061S could only be detected by P5 and PM but not P3 (Figure 4c). These findings indicate that the rho‐independent terminator is present in sRX061L but lacking in sRX061S and suggest that sRX061S is generated from SRX061L by removing the terminator.

**FIGURE 5:**
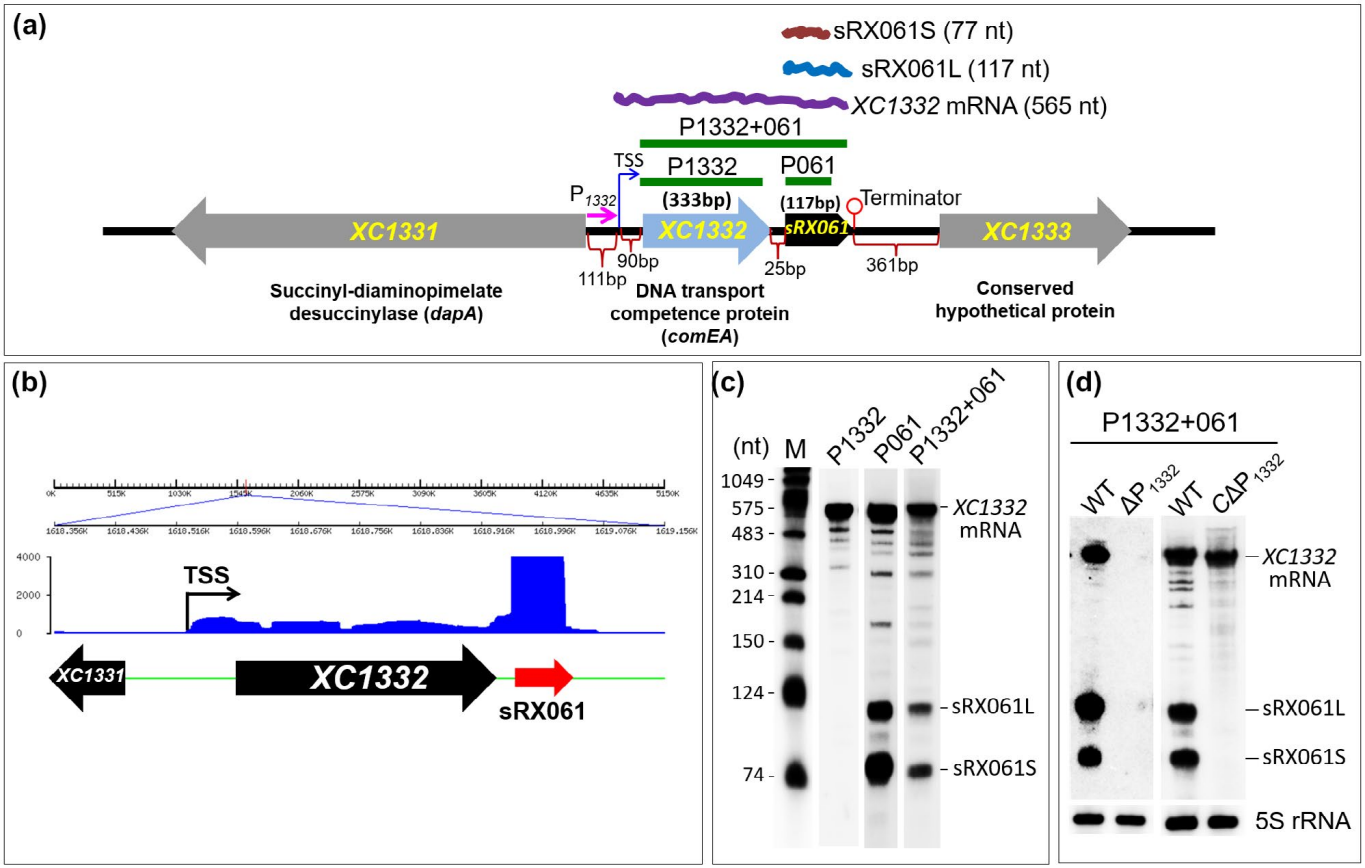
Genetic organization of the *sRX061* locus, identification of sRX061 by sRNA transcriptome analysis, and detection of sRX061 expression by northern blotting. (a) The genetic organization of the *sRX061* locus and the specific probes used in northern blotting analyses below. (b) The visualized mapping pattern of *sRX061* region and the precise genome location of *sRX061* predicted according to RNA‐Seq data. The genome location of the protein‐coding genes *XC1331*, *XC1332*, and *XC1333* is based on the genome sequencing and annotation data of *Xanthomonas campestris* pv. *campestris* (Xcc) strain 8,004. The black arrows indicate the protein‐coding gene and its transcriptional orientation and the gene ID is shown inside the arrow. The red arrow indicates sRX061 and its transcriptional direction. The *y* axis is the number of mapped reads. (c) Detection of the transcripts transcribed from the *XC1332* region by northern blotting using the probes specific to the *XC1332* coding region (P1332), sRX061 (P061), or the *XC1332* coding region and sRX061 (P1332 + 061). (d) Detection of sRX061 expression in wild‐type strain (WT), the promoter P*_1332_* deletion mutant (ΔP*_1332_*) and strain CΔP*_1332_* (ΔP_1332_ carrying a recombinant plasmid containing the *XC1332* coding sequence) by northern blotting using P1332 + 061 probe. The positions of the probes used in the experiment are shown as thick green lines in (a). 5S rRNA was probed as a loading control. TSS, transcriptional start site

As shown in Figure 3f, northern blotting using the sRX061 probe also detected a c.600‐nt transcript in addition to sRX061S and sRX061L. It is possible that the c.600‐nt transcript is the precursor of sRX061, or an independent transcript that overlaps with sRX061, or a nonspecific hybridization signal. According to the genetic organization of the *sRX061* locus in the genome of Xcc strain 8,004 (Figure 5a), the *sRX061* sequence is located within the 3′‐UTR of *XC1332*, which encodes ComEA, a protein essential for the transfer of external DNA into the periplasm of gram‐negative bacterial cells (Seitz *et al*., 2014). The *sRX061* sequence begins 25 bp downstream of the stop codon of *XC1332*. The visualized mapping pattern of the RNA‐Seq reads in the *XC1332* region also shows that the 77‐nt sRX061S transcript is within the 3′‐UTR of *XC1332* mRNA (Figure 5b). Because *XC1332* and *sRX061* are transcribed in the same orientation, it is very likely that the c.600‐nt transcript is the *XC1332* mRNA. The c.600‐nt transcript could be detected by northern blotting using a probe specific for the *XC1332* coding region (P1332), or for sRX061 (P061), or for the *XC1332* coding region plus sRX061 (P1332 + 061) (Figure 5a,c), demonstrating that the c.600‐nt transcript is indeed the *XC1332* mRNA and that sRX061 is contained within the *XC1332* mRNA sequence. In addition, sRX061 could be detected by the probe specific for the *XC1332* coding region plus sRX061 (P1332 + 061) but not the probe specific for the *XC1332* coding region (P1332) (Figure 5a,c), demonstrating that sRX061 overlaps with the 3′‐UTR of *XC1332* mRNA.

To determine whether sRX061 has its own promoter or is cotranscribed with *XC1332*, a *XC1332* promoter (P*_1332_*) deletion mutant ΔP_1332_ (Table S1) was constructed and the effect of P*_1332_* deletion on sRX061 expression was determined by northern blotting. The results showed that neither sRX061L nor sRX061S was detectable in the P*_1332_* deletion mutant (Figure 5c), suggesting that *sRX061* is cotranscribed with *XC1332*. To rule out the possibility that the disappearance of sRX061 in the P*_1332_* deletion mutant is due to absence of activation by XC1332, the XC1332‐expressing plasmid pL1332 was constructed and introduced into the P*_1332_* deletion mutant to create the complemented strain CΔP_1332_ (Table S1). Northern blotting showed that sRX061 expression could not be rescued by *XC1332* in trans, although the *XC1332* mRNA signal was restored (Figure 5d). Taken together, these results demonstrate that sRX061 is derived from the 3′‐UTR of *XC1332* mRNA. The mechanism by which *XC1332* mRNA is processed to generate sRX061L and sRX061S remains to be investigated.

To determine whether the 3′‐UTR of *XC1332* plays a role in the accumulation of *XC1332* mRNA, the *XC1332* 3′‐UTR deletion mutant Δ3′UTR was constructed and the *XC1332* mRNA level in Δ3′UTR was tested by northern blotting. The result showed that the level of *XC1332* mRNA in Δ3′UTR was slightly lower compared to the wild‐type strain (Figure S10), suggesting that the 3′‐UTR of *XC1332* plays a minor role in *XC1332* mRNA accumulation. As shown in Figure 3, overexpression of sRX061 resulted in a significant reduction in virulence, loss of HR, and impairment of swarming motility. To clarify whether deletion of sRX061 has any effect on these phenotypes, the virulence, HR, and swarming motility of the sRX061‐deficient strain CΔP_1332_ were examined. The results showed that the virulence, HR, and swarming motility of the sRX061‐deficient strain CΔP_1332_ were similar to the wild‐type strain (Figure S11), implying that sRX061 is dispensable for virulence, HR, and swarming motility. In addition, we also tested the virulence, HR, and swarming motility of ΔP_1332_, a mutant strain that cannot generate either sRX061 or *XC1332* mRNA. The results showed that the virulence, HR, and swarming motility of the mutant strain ΔP_1332_ and the wild‐type strain were comparable (Figure S11), suggesting that *XC1332* mRNA and XC1332 protein are not essential for virulence, HR, and swarming motility in Xcc.

### 2.5 sRX061 is a novel RsmA/CsrA‐sequestering sRNA

**FIGURE 6:**
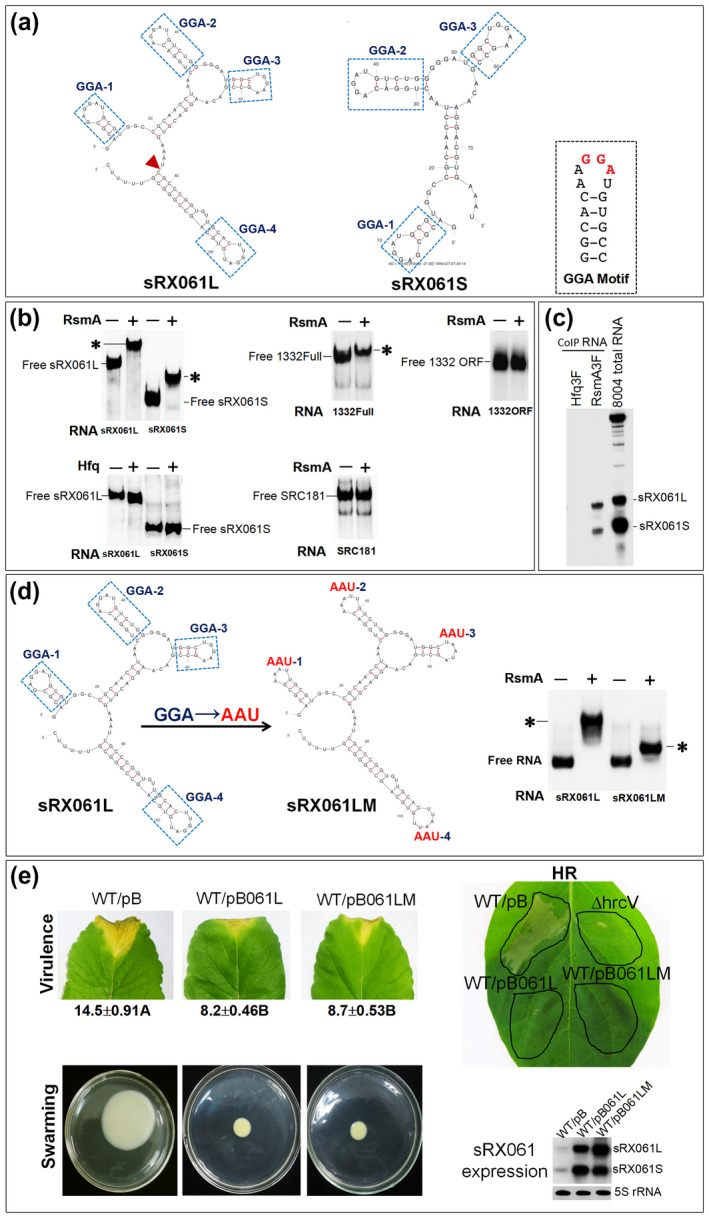
The potential secondary structure of sRX061L and sRX061S, and the interaction of sRX061L and sRX061S with RsmA protein in vivo and in vitro. (a) The secondary structure of SRX061L and SRX061S predicted by using M‐fold software (http://unafold.rna.albany.edu/?q=mfold/RNA‐Folding‐Form). GGA motifs are highlighted by dotted line boxes. (b) Detection of the binding of sRX061L, sRX061S, 1332Full, and 1332ORF with RsmA in vitro by electrophoretic mobility shift assay (EMSA). SRC181 was used as a negative RNA control and Hfq was used as a negative protein control. Bands referring to the protein–RNA complex are marked with an asterisk (*). (c) Demonstration of the specific binding of sRX061L and sRX061S with RsmA in vivo by coimmunoprecipitation (Co‐IP) analysis. Cell extracts were prepared from the wild‐type strain 8‐004 and the RsmA‐3 × FLAG expressing strain RsmA3F. Co‐IP was carried out in the extracts with anti‐FLAG antibody to isolate the RsmA–RNA complex and RNAs (CoIP RNA) were extracted from the RsmA–RNA complex prepared from the wild‐type *Xanthomonas campestris* pv. *campestris* (Xcc) strain 8,004 and the RsmA‐3 × FLAG‐expressing strain RsmA3F. The existence of sRX061L and sRX061S in the CoIP RNA samples was detected by northern blotting using an sRX061‐specific probe. The total RNA of strain 8,004 (8,004 total RNA) was used as a positive control. The bands corresponding to sRX061L and sRX061S are indicated. (d) The effect of substitution of AAU for all of the four predicted GGA motifs in sRX061 on RsmA‐binding ability. Left: the sequence and predicted secondary structure of sRX061L and sRX061LM. Right: EMSA detection of in vitro binding of RsmA to sRX061L and sRX061LM, respectively. (e) Effect of substitution of AAU for all four predicted GGA motifs in sRX061 on the biological function of sRX061. The virulence, hypersensitive response (HR), and swarming motility of sRX061L overexpression strain (WT/pB061L) and sRX061LM overexpression strain (WT/pB061LM) were tested and compare to those of the pBBad‐carrying wild‐type strain (WT/pB). The values under each leaf are the average lesion lengths (mean ± *SD*) from three repeats, each with 50 leaves. Different letters after the values of the average lesion lengths indicate significant differences (*t* test, *p* < .01). In addition, the levels of sRX061 in WT/pB, WT/pB061L, and WT/pB061LM were detected by northern blotting. Strains were cultured in NYG medium at 28 °C with shaking at 200 rpm for 24 hr. Total RNAs were isolated and 3 μg was used for northern blotting with digoxigenin‐labelled sRX061 probe. 5S rRNA was probed as a loading control

Investigation of the Rfam database v. 13.0 (Kalvari *et al*., 2018) showed no homologue of sRX061, indicating that this is a novel sRNA. The secondary structure prediction, using M‐fold software (http://unafold.rna.albany.edu/?q=mfold/RNA‐Folding‐Form) (Zuker, 2003), showed that sRX061L and sRX061S contain four and three GGA motifs, respectively (Figure 6a). This RNA structural element has been shown to be able to bind specifically with the bacterial global posttranscriptional regulator protein RsmA (also called CsrA). Binding of sRNAs with this motif to RsmA/CsrA results in sequestration and hence they can antagonize the activity of RsmA/CsrA (Romeo and Babitzke, 2018).

The genome of Xcc strain 8,004 contains an *rsmA* gene (*XC2506*; *rsmA*_Xcc_) (Qian *et al*., 2005; Chao *et al*., 2008). To determine whether sRX061L and sRX061S can bind with RsmA_Xcc_ directly, an electrophoretic mobility shift assay (EMSA) was performed. The result showed that both sRX061L and sRX061S bind to RsmA_Xcc_ (but not the RNA chaperone Hfq) in vitro (Figure 6b). Results also showed that the full‐length *XC1332* mRNA (including 5′‐UTR, coding region, and 3′‐UTR, designated as 1332Full) but not the coding region (designated as 1332ORF) could bind to RsmA (Figure 6b). Furthermore, the RsmA_Xcc_‐RNA coimmunoprecipitation (Co‐IP) experiment also showed that both sRX061S and sRX061L could specifically bind to RsmA_Xcc_ in vivo. These results demonstrate that sRX061S and sRX061L are RsmA‐binding sRNAs.

To determine whether the four predicted GGA motifs in sRX061 are important for RsmA binding, the RsmA‐binding ability of sRX061LM, a mutant construct derived from sRX061L in which all of the four predicted GGA motifs in sRX061L were changed to AAU, was tested by EMSA. The result showed that sRX061LM could still bind with RsmA efficiently in vitro (Figure 6d). However, as shown in Figure 6d, the position of the band corresponding to RsmA–sRX061LM complex was much lower than that of RsmA–sRX061L complex, indicating that the molecular weight of RsmA–sRX061LM complex is smaller than that of the RsmA–sRX061L complex, possibly due to fewer RsmA molecules in the RsmA–sRX061LM complex compared to the RsmA–sRX061L complex. These data suggest that some of the four predicted GGA motifs, if not all, are important for RsmA binding and that other RsmA‐binding motif(s) in sRX061L may also play a role.

Given that RsmA binds specifically to sRX061 and the full‐length *XC1332* mRNA, it is possible that the stability of the two RNAs is influenced by RsmA binding. To test this possibility, the accumulation of sRX061 and *XC1332* mRNA in the *rsmA* deletion mutant ΔrsmA was tested by northern blotting. The result showed that sRX061 was undetectable in ΔrsmA and *XC1332* mRNA was more unstable in ΔrsmA compared to the wild‐type strain (Figure S12), implying that RsmA contributes to *XC1332* mRNA stability and is essential for sRX061 production.

**FIGURE 7:**
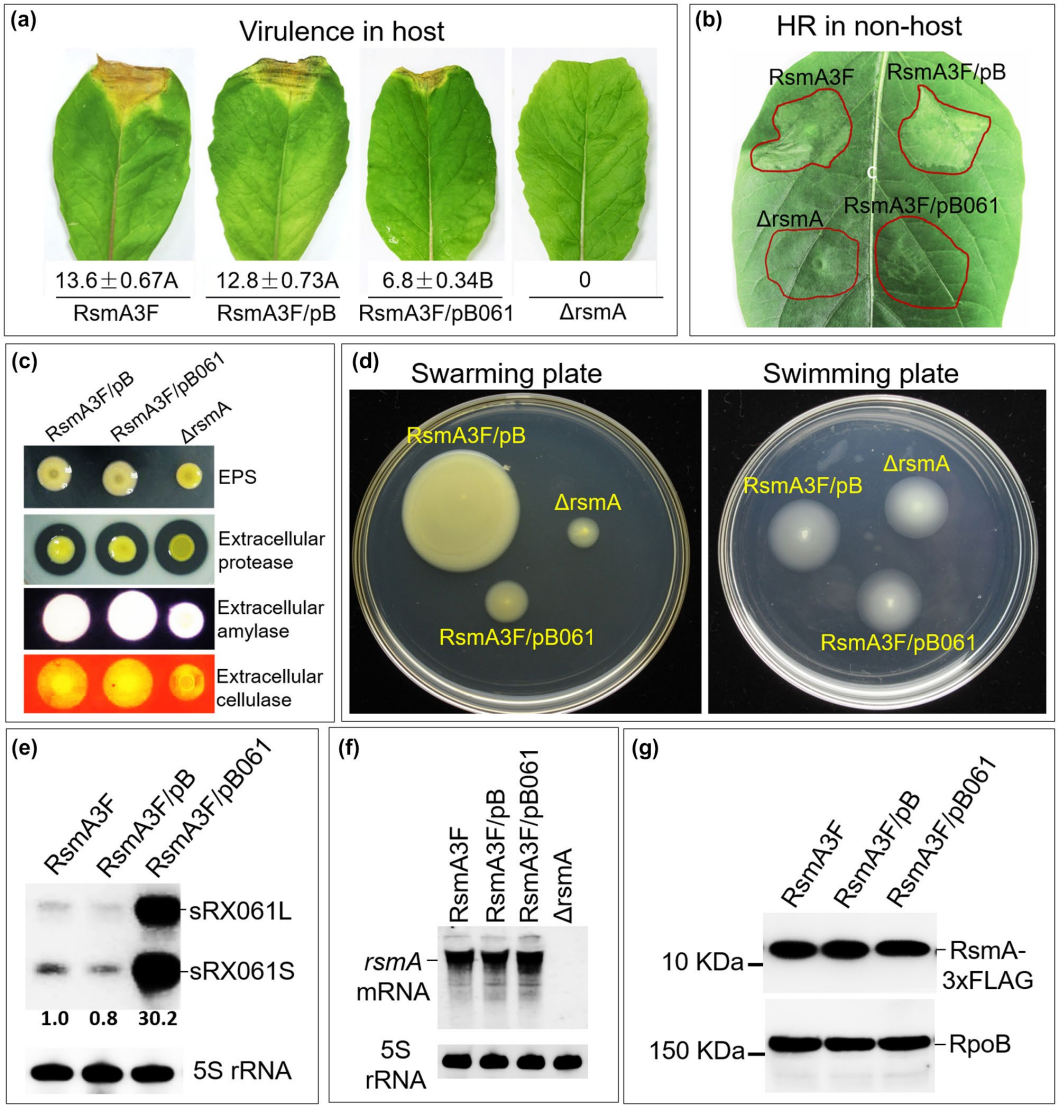
Phenotypic comparison between sRX061 overexpression strain and the *rsmA* deletion mutant, and the effect of sRX061 overexpression on the cellular level of RsmA protein and *rsmA* mRNA. (a) Black rot symptoms and the average lesion lengths (in mm) caused by the pBBad‐carrying wild‐type strain WT/pB and the sRX061 overexpression strain RsmA3F/pB061 in inoculated leaves of Chinese radish. Images were taken at 10 days postinoculation. The values under each leaf are the average lesion lengths (mean ± *SD*) from three repeats, each with 50 leaves. Different letters after the values of the average lesion lengths indicate significant differences (*t* test, *p* < .01). (b) The hypersensitive response (HR) induced in nonhost plant pepper leaves (*Capsicum annuum* 'ECW‐10R') by *Xanthomonas campestris* pv. *campestris* (Xcc) strains. An overnight culture of each strain (OD_600_ = 1.0) was resuspended in 10 mM sodium phosphate buffer to OD_600_ = 0.001 and approximately 5 µl of the suspension was infiltrated into the leaf mesophyll tissue with a 1‐ml blunt‐end plastic syringe. The photograph was taken 24 hr after infiltration. (c) Detection of extracellular polysaccharide (EPS) production and the activity of extracellular enzymes. Zones of clearance around the spots are due to the degradation of the substrate by enzyme activity. The relative EPS yield was evaluated by the size of the colonies, and the relative activity of the enzyme was indicated by the diameter of the clear zone. Three plates were inoculated in each experiment. The experiment was repeated three times and similar results were obtained. (d) Swarming motility and swimming motility assay. Two microlitres of overnight culture (OD_600_ = 1.0) of each strain was spotted onto the swarming assay plate and the swimming assay plate, and results were observed and photographed after incubation at 28 °C for 3 days. (e) Detection of sRX061 levels in the pBBad‐carrying wild‐type strain WT/pB and the sRX061 overexpression strain RsmA3F/pB061. The Xcc RsmA‐3 × FLAG‐expressing strain RsmA3F, RsmA3F carrying pBBad (RsmA3F/pB), and the SRX061 overexpression strain RsmA3F/pB061 were cultured in MMX medium at 28 °C with shaking at 200 rpm for 24 hr. Total RNAs were isolated and 3 μg was used for northern blotting with the digoxigenin‐labelled sRX061 probe. 5S rRNA was probed as a loading control. (f) Detection of RsmA level in different Xcc strains by western blotting. The detection of RNA polymerase subunit β (RpoB) served as an internal loading control. Three replications were done in the experiment, which was repeated three times. The results presented are from a representative experiment and similar results were obtained in all other independent experiments. (g) Detection of *rsmA* mRNA levels in different Xcc strains by northern blotting. 5S rRNA was probed as a loading control

It is known that a role of the RsmA/CsrA‐binding sRNAs is to inactivate the activity of RsmA/CsrA (Romeo and Babitzke, 2018). If sRX061 really acts as an RsmA‐antagonizing sRNA in Xcc, overexpression of sRX061 should result in a phenotypic similarity to an *rsmA*_Xcc_ deletion mutant. Previous work showed that deletion of *rsmA*_Xcc_ resulted in a loss of virulence and HR as well as a significant reduction in cell motility and the production of the virulence factors EPS and extracellular enzymes (amylase and cellulase) (Chao *et al*., 2008). The sRX061 overexpression strain displayed a phenotype similar to that of the *rsmA*_Xcc_ deletion mutant in terms of reduction of virulence, loss of HR, and swarming motility (Figure 7a,b,d). However, overexpression of sRX061 did not affect the production of EPS and extracellular enzymes (amylase and cellulase) (Figure 7c). The findings indicate that sRX061 may act as an RsmA_Xcc_‐sequestering sRNA in Xcc, but that the regulons of sRX061 and RsmA_Xcc_ are not identical. To determine the effect of GGA → AAU substitution on the function of sRX061, an sRX061LM overexpression strain (named WT/pB061LM) was constructed and its virulence, HR, and swarming motility were tested. The result showed that WT/pB061LM also induced a significant reduction in virulence, loss of HR, and impairment of swarming motility, similar to the wild‐type strain overexpressing sRX061L (WT/pB061L) (Figure 6e), implying that substitution of AAU for all of the four predicted GGA motifs in sRX061 did not affect the function of sRX061.

To rule out the possibility that the *rsmA*_Xcc_ deletion mutant‐like phenotype of the sRX061 overexpression strain is due to a reduction in RsmA_Xcc_ accumulation (rather than inactivation by sequestration), the levels of RsmA were measured in different Xcc strains. For this, the sRX061‐expressing plasmid pB061 was introduced into strain RsmA3F, which expresses a recombinant RsmA_Xcc_ protein with a 3 × FLAG tag at its C‐terminus (RsmA_Xcc_‐3 × FLAG) (Table S1). Simultaneously, the vector pBBad alone was introduced into RsmA3F to generate the control strain RsmA3F/pB (Table S1). The northern blotting result showed that sRX061 was expressed 30‐fold in RsmA3F/pB061 compared to the control strains RsmA3F and RsmA3F/pB (Figure 7e) and that the *rsmA* mRNA levels were similar in the three strains (i.e., RsmA3F, RsmA3F/pB, and RsmA3F/pB061) (Figure 7f). Western blotting analysis revealed that RsmA_Xcc_‐3 × FLAG accumulated in a similar level in the three strains RsmA3F, RsmA3F/pB, and RsmA3F/pB061 (Figure 7g). These results demonstrate that sRX061 overexpression does not affect the expression of RsmA_Xcc_ at either the transcriptional or translational levels.

Using the sequence of sRX061L and sRX061S to search the Rfam database (http://rfam.xfam.org/search?tab=searchSequenceBlock#tabview=tab) (Kalvari *et al*., 2018) revealed that sRX061 is not homologous to any known RNAs. Because all known RsmA/CsrA‐sequestering sRNAs have been collected in the Rfam database, this result indicates that sRX061 represents the first member of a novel family of RsmA/CsrA‐sequestering sRNAs.

## 3 DISCUSSION

In this study, we identified 612 SRCs of Xcc by RNA‐Seq of the 50–500 nt RNA fragments. More than 88% of the identified Xcc SRCs were 51 to 120 nt in length (Figure 1b). No SRC smaller than 44 nt or bigger than 423 nt was found. The fact that 117 out of 121 SRCs identified by RNA‐Seq were detectable by northern blotting when the bacterial cells were grown in the same conditions suggests that the overwhelming majority of the 612 SRCs are sRNAs. The SRCs that were detectable in the northern blotting were named sRX (small RNA of Xcc).

Of the 612 SRCs, only 108 (18%) were derived from the IGR, with the majority (82%) being derived from mRNA, comprising 31% from the 5′‐UTR, 9% from the 3′‐UTR, and 42% from within the region coding for proteins. Northern blotting analysis demonstrated that the transcriptional orientation of these mRNA‐derived sRNAs was always the same as that of their overlapping protein‐coding gene. Promoter prediction revealed no typical bacterial promoter in the 100 bp flanking region of these mRNA‐derived sRNAs. These results strongly suggest that these sRNAs are generated by excision/processing from mRNA rather than by independent transcription of sRNA genes. The majority of bacterial sRNAs so far identified are encoded within IGRs and are independently transcribed (Vogel *et al*., 2003; Kawano *et al*., 2005; Loh *et al*., 2009; Chao *et al*., 2012; Chao and Vogel, 2016; Dar and Sorek 2018). Nevertheless, a few 3′‐UTR‐derived sRNAs (Kawano *et al*., 2005; Kim *et al*., 2014; Chao and Vogel, 2016; Chao *et al*., 2017; Eisenhardt *et al*., 2018; Miyakoshi *et al*., 2019), 5′‐UTR‐derived sRNAs (Kawano *et al*., 2005; Drecktrah *et al*., 2018), and protein coding region‐derived sRNAs (called decay‐generated noncoding RNAs [decRNAs]) (Dar and Sorek, 2018) have been reported in different bacterial species. Surprisingly, our study demonstrated that, in Xcc, mRNA processing is the primary source of cellular sRNA, suggesting a close link between sRNA biogenesis and mRNA decay.

We employed the overexpression approach to study the function of the sRNAs. Overexpressing sRX061 in the wild‐type strain gave a significant effect for various aspects, including a severe reduction in virulence, indicating that the sRNA sRX061 plays an important role in Xcc pathogenesis. Northern blotting revealed that sRX061 has two isoforms, sRX061L and sRX061S. Taking this result together with the RNA‐Seq and related genome data, sRX061L is 117 nt in length and contains a 40‐nt rho‐independent terminator, while sRX061S lacks the terminator and is only 77 nt. The two isoforms were always present when sRX061L was cloned into the pBBad vector; however, neither could be detected when sRX061S was cloned into the same vector even though there is an rho‐independent terminator present downstream of the cloning site in the vector. The reason for this phenomenon is unclear, although our work has demonstrated that both sRX061S and sRX061L are processed from the 3′‐UTR of *XC1332* mRNA. It is possible that the rho‐independent terminator is indispensable for the processing of sRX061.

In vitro EMSA and in vivo Co‐IP analyses revealed that sRX061 interacts physically with the regulator protein RsmA. It is well known that RsmA/CsrA is a family of RNA‐binding proteins that is widely distributed and highly conserved in different bacterial species and plays an important role in the regulation of various cellular processes (Vakulskas *et al*., 2015; Romeo and Babitzke, 2018). RsmA/CsrA acts as a global posttranscriptional regulator, controlling gene expression mainly through directly binding to its target mRNA to affect the translation and/or stability of the target mRNA, and its activity can be antagonized by some regulatory sRNAs (Romeo and Babitzke, 2018). Previous work showed that deletion of the RsmA in Xcc resulted in a complete loss of virulence, HR, and cell motility as well as a significant reduction in extracellular enzymes and polysaccharide production (Chao *et al*., 2008). Given that sRX061 and RsmA interact with each other and that overexpression of sRX061 and deletion of RsmA gave a similar effect for some aspects (virulence, HR, and cell motility), sRX061 may be a RsmA‐antagonizing sRNA, although the phenotypes of the sRX061 overexpression strain and the *rsmA* deletion strain are not identical (e.g., the production of extracellular enzymes and polysaccharides is severely reduced by deleting *rsmA* but is not significantly affected by overexpressing sRX061). Both sRX061L and sRX061S can interact with RsmA in vitro and in vivo (Figure 6), indicating that both isoforms of sRX061 may be functional.

RsmA has also been characterized in other two *Xanthomonas* species, *X. oryzae* pv. *oryzae* (the rice leaf blight pathogen) (Zhu *et al*., 2011) and *X*. *citri* subsp. *citri* (the citrus canker pathogen) (Andrade *et al*., 2014). However, no RsmA‐sequestering sRNA has been identified in these bacteria. As in Xcc, deletion of the *rsmA* gene in these pathogens also severely affects virulence, HR, and cell motility, as well as the production of extracellular enzymes and polysaccharides (Zhu *et al*., 2011; Andrade *et al*., 2014). Several families of RsmA/CsrA‐sequestering sRNAs, such as CsrB/RsmB, CsrC, RsmV, RsmW, RsmX, RsmY, and RsmZ, have been identified from *Escherichia coli*, *Erwinia carotovorum*, *P. aeruginosa*, *Pseudomonas fluorescens*, and *S. enterica* (Miller *et al*., 2016; Janssen *et al*., 2018; Romeo and Babitzke, 2018). Using the sequence of CsrB/RsmB, CsrC, RsmV, RsmW, RsmX, RsmY, and RsmZ to search the NCBI genome database (https://blast.ncbi.nlm.nih.gov/) revealed that no significant homologue is present in any sequenced *Xanthomonas* genome. An NCBI BLAST search (https://blast.ncbi.nlm.nih.gov/) revealed that sRX061 is highly conserved (>90% nucleotide sequence identity) in all *Xanthomonas* strains whose genomes have been determined but in no other bacteria. These data suggest that sRX061 may represent a novel family of RsmA/CsrA‐sequestering sRNAs that is limited to the genus *Xanthomonas*. As a consequence, we renamed sRX061 as RsmU.

In summary, genome‐wide screening and functional analysis of sRNAs in the important Xcc led to the discovery of a large number of mRNA‐derived sRNAs and a novel RsmA‐sequestering sRNA that plays an important role in virulence regulation of Xcc. Our study uncovered an unexpected major sRNA biogenesis pathway and a hidden layer of sRNA‐mediated virulence regulation in Xcc. The discovery of a large number of mRNA‐derived sRNAs suggests that sRNA biogenesis in bacteria is far more complex than previously thought.

## 4 EXPERIMENTAL PROCEDURES

### 4.1 Bacterial strains and growth conditions

The bacterial strains and plasmids used in this work are listed in Table S1. *E. coli* strains were grown in Luria Bertani medium (Miller, 1972) at 37 °C. Xcc strains were grown in the rich medium NYG (Daniels *et al*., 1984b) or the minimal medium MMX (Daniels *et al*., 1984a) at 28 °C. Antibiotics were used at the following final concentrations: ampicillin 100 μg/ml, chloramphenicol 100 μg/ml, kanamycin 25 μg/ml, rifampicin 50 μg/ml, and tetracycline 15 μg/ml for *E. coli* and 5 μg/ml for Xcc.

### 4.2 Preparation of total RNAs for construction of Illumina/Solexa sequencing cDNA library

Overnight cultures of the Xcc wild‐type strain 8,004 (Table S1) were inoculated in 50 ml of the rich medium NYG and incubated overnight at 28 °C in a shaking incubator at 200 rpm. Bacterial cells were collected, washed with sterilized water, and resuspended in the minimal medium MMX to a concentration of OD_600_ = 1.0. Five millilitres of the suspension was inoculated into a 100‐ml flask containing 50 ml of fresh MMX and incubated at 28 °C with 200 rpm shaking. Bacterial cells were harvested at exponential phase (OD_600_ ≈ 0.8) and then total RNAs were extracted by using the hot phenol method (Rochester *et al*., 1986). Genomic DNA was removed from RNA samples by treatment with RNase‐free DNase (Qiagen). The quantity of the isolated total RNA was determined by absorbance (A) at 260 and 280 nm using a Qubit fluorometer (Thermo Fisher Scientific), and the quality was assessed using Nano‐chips for Agilent's 2100 Bioanalyzer (Thermo Fisher Scientific). The RNA samples with A_260_/A_280_ = 1.8–2.2, 23S:16S ≥ 1, and RIN ≥ 8 were considered high quality and used for cDNA library construction. The high‐quality RNA was stored at −80 °C until needed.

### 4.3 Library construction and Illumina/Solexa sequencing

First, rRNAs (23S, 16S, and 5S) were removed by using the Ribo‐Zero rRNA Removal Kit (Gram‐Negative Bacteria) (Epicentre), then 50 µg of RNA was fractionated by denaturing 8% polyacrylamide gel (7 M urea, 0.5 × TBE buffer) electrophoresis (PAGE). The gel containing target RNAs with sizes of about 50–200 and 200–500 nt were excised, and RNAs were extracted from the excised gel using the small RNA Gel Extraction Kit D9106 (TaKaRa). Illumina/Solexa paired‐end sequencing libraries were constructed from the 50–200 nt RNA sample and the 200–500 nt RNA sample using the Illumina mRNA‐Seq sample preparation kit (the first cDNA strand was synthesized using random hexamer primers) following the manufacturer's instructions. Fragments of 100–200 bp (for the library of the 50–200 nt RNA sample) or 200–300 bp (for the library of the 200–500 nt RNA sample) double‐stranded cDNA were excised and enriched by PCR for 10 cycles. Libraries were assayed for quality by using Agilent's 2100 Bioanalyzer (Thermo Fisher Scientific). Each cDNA library was diluted to 5 pM and sequenced by the Illumina/Solexa HiSeq2000 platform using the PE‐91 program. Sequencing was performed at the Beijing Genomics Institute at Shenzhen, China. The raw reads of the two sequencing runs (the 50–200 nt library and the 200–500 nt library) of an RNA sample were combined to generate one raw read set for further analysis. The raw reads from all RNA samples have been deposited in the NCBI Sequence Read Archive (accession number PRJNA598782).

### 4.4 Genome mapping and visualization of the Illumina/Solexa sequencing reads

After clipping, linker removal, and quality control, the clean sequencing reads were mapped to the genome of Xcc strain 8,004 (Qian *et al*., 2005) using the SOAP2 program (Li *et al*., 2009) allowing two nucleotides mismatch. Reads that did not fulfil this requirement were removed from the data set. The mapped reads data sets were processed using a visualized mapping program (National Center for Gene Research, Shanghai, China), which outputs a graph showing the detailed mapping information of the RNA‐Seq data in any region on the reference genome, including the sequence coverage at each genomic position at single‐nucleotide resolution.

### 4.5 Definition, identification, and quantification of the TTs

As mentioned above, only the RNA molecules between 50 and 500 nt were selected for sequencing in this study. Thus, theoretically, the following three distinct classes of RNA molecules should be captured: (a) full‐length transcripts of 50–500 nt, (b) stable 50–500 nt RNA fragments processed from a long transcript, and (c) unstable 50–500 nt RNA fragments, possibly the mRNA degradation intermediates. The TTs belong to the first two classes; the last class may be considered as background transcripts. TTs were identified from the visualized mapping data based on two criteria: (a) a TT is a contig whose length is between 50 and 500 nt, and (b) the coverage of the contig is ≥2‐fold greater than that of its 50 nt flanking region. TTs were first identified by program (National Center for Gene Research, Shanghai, China), and then inspected and corrected manually (Figure S9). The transcriptional level of each TT was quantified by calculating the number of reads mapped to it. A comparable value was further generated by calculating the reads per kilobases per million reads (RPKM) value.

### 4.6 Identification of SRCs from TTs

All identified TTs were scanned for small ORFs using the online software FramePlot v. 2.3.2 (http://www.nih.go.jp/~jun/cgi‐bin/frameplot.pl) (Ishikawa and Hotta, 1999), applying an ORF length detection cut‐off ≥30 amino acids. Except for small ORFs, all other identified TTs were considered to be putative sRNAs.

### 4.7 Homology searches and phylogenetic analysis of sRNAs

Homology searches were based on scans of the bacterial NCBI genome database (https://blast.ncbi.nlm.nih.gov/) and the Rfam database v. 13.0 (http://rfam.xfam.org/) (Kalvari *et al*., 2018).

### 4.8 Prediction of promoters and terminators

σ_70_ promoters were identified using the promoter prediction program (PPP) (http://bioinformatics.biol.rug.nl/websoftware/ppp/) and the neural network for promoter prediction (NNPP) (Reese, 2001). Rho‐independent terminators were predicted using TransTermHP (Kingsford *et al*., 2007) and RNAMotif (Macke *et al*., 2001). Because our Illumina/Solexa sequencing did not allow for identification of the transcribed strand, except for those sRNAs whose transcribed strands have been determined by northern blotting, for each identified sRNA a region of 100 nt flanking sequence was scanned for the existence of σ_70_ promoters or rho‐independent terminators.

### 4.9 Northern blotting

Xcc cells were cultured and collected as described above. Total RNA was isolated using the PureLink RNA Mini kit (Thermo Fisher Scientific), and 3–5 μg of total RNA were separated on 6% denaturing (8 M urea) polyacrylamide gel and transferred to a positively charged nylon membrane (Roche Applied Science). After UV‐crosslinking, the membrane was hybridized with a DIG‐labelled RNA probe (prepared using a DIG RNA labelling kit [Roche Applied Science]) at 68 °C for 8 hr. Signal bands were detected using the DIG‐Northern Starter Kit (Roche Applied Science) and visualized with an ImageQuant LAS 500 imager (GE Healthcare). Signal bands were quantified using GelQuant.NET software (biochemlabsolutions.com).

### 4.10 Construction of sRNA overexpression strains

An entire sRNA gene was amplified by PCR using Xcc strain 8,004 genomic DNA as template and the sRNA gene‐specific primer pair (Table S7). After confirmation by sequencing, the DNA fragment was cloned into the broad host range expression vector pBBad (Sukchawalit *et al*., 1999) to generate the recombinant plasmid named pBsRX (Table S1). The plasmid pBsRX was transferred into Xcc strain 8,004 by triparental conjugation. The transconjugants carrying pBsRX were screened on NYG plates containing rifampicin and kanamycin. A confirmed transconjugant representative was named WT/pBsRX (Table S1) and chosen for further study.

### 4.11 Measurement of sRNA expression level by semiquantitative RT‐PCR

Xcc cells were cultured and collected as described above. Total RNA was isolated using the PureLink RNA Mini kit (Thermo Fisher Scientific), and any contaminating DNA was removed by gDNA Eraser (TaKaRa) treatment. cDNAs were synthesized from the RNA by reverse transcription using a RevertAid First Strand cDNA Synthesis kit (Fermentas China Co., Ltd) and used as the template for PCR amplification of a target sRNA gene. PCR was performed with a cycler using the following cycle parameters: 30 cycles of 94 °C for 15 s, 60 °C for 15 s, and 72 °C for 15 s. The resulting amplification products were analysed in 1.2% agarose gels and quantified using GelQuant.NET software. The 16S rRNA gene of Xcc strain 8,004 was used as the internal control to verify absence of significant variation at the cDNA level in the two RNA samples.

### 4.12 Virulence assay and determination of bacterial growth in planta

The virulence of Xcc strains was tested on the leaves of Chinese radish (*R. sativus* var. *radiculus*) using the leaf‐clipping method (An *et al*., 2017). Xcc strains were grown in NYG medium at 28 °C with shaking at 200 rpm for 15 hr. Cell concentration was adjusted to OD_600_ = 0.001 in water. Two to three fully expanded leaves per plant were inoculated by leaf clipping (the leaves were cut with scissors dipped in the bacterial suspensions). Lesion length was measured and photographs were taken 10 dpi. Fifty leaves were inoculated for each strain in each independent experiment. The experiment was repeated three times.

The growth of bacteria in radish leaves was measured by homogenizing a group of leaves (five leaves for each sampling) in 9 ml of sterile water. Diluted homogenates were plated on NYG medium supplemented with rifampicin (for the wild‐type strain) or rifampicin plus kanamycin (for the mutant strain). Bacterial colonies were counted after incubation at 28 °C for 3 days.

### 4.13 HR test

The HR of Xcc was tested on the nonhost plant pepper (*Capsicum annuum* 'ECW‐10R') (Castañeda *et al*., 2005). The plants were inoculated by infiltrating approximately 5 μl of bacterial culture (OD_600_ = 0.01) suspended in 10 mM sodium phosphate buffer (Na_2_HPO_4_ 5.8 mM, NaH_2_PO_4_ 4.2 mM, pH 7.0) into the abaxial leaf surface using a 1‐ml blunt‐end plastic syringe. The inoculated plants were maintained in a greenhouse with a 12‐hr day‐and‐night cycle with illumination by fluorescent lamps and a constant temperature of 28 °C. HR symptoms were photographed 24 hr postinoculation (hpi). For quantification of the HR, electrolyte leakage induced by Xcc was measured at 0, 6, 8, 10, 12, and 14 hpi using the method described by Torres *et al*. (2005). Briefly, 0.7‐cm diameter leaf discs were taken from injected leaves and incubated in vials containing 10 ml of double distilled H_2_O at room temperature with continuous stirring. After 30 min, the conductivity of the liquid was measured using a DDSJ‐318 conductivity meter. Three assays of six leaf disks per treatment were carried out. Student's two‐tailed *t* test was performed for comparison of mean conductivity of two strains. Each experiment was performed at least twice.

### 4.14 Cell motility assay

To test swarming motility, an overnight culture (OD_600_ = 1.0) of each Xcc strain was inoculated onto NY plates containing 2% glucose and 0.6% agar using a toothpick, and then incubated at 28 °C for 3 days. To detect swimming motility, the bacterial cells were stabbed into 0.28% agar plates composed of 0.03% Bacto peptone and 0.03% yeast extract followed by incubation at 28 °C for 4 days.

### 4.15 Test of extracellular enzymes and EPS production

Relative activities of extracellular enzymes were assayed as described previously (Tang *et al*., 1987). Two microlitres of Xcc cultures (OD_600_ = 1.0) was spotted onto NYG plates containing 1% (wt/vol) skimmed milk (for protease), 0.5% (wt/vol) carboxymethyl cellulose (for cellulase), or 0.1% (wt/vol) starch (for amylase) and incubated at 28 °C for 24 hr. The starch plates were stained with KI/I_2_ and the cellulose plates were stained with Congo red. Zones of clearance around the spot due to the degradation of the substrate were photographed. Three plates were inoculated in each experiment, and each experiment was repeated three times. The relative activity of the enzyme was indicated by the diameter of the clear zone. EPS production was measured as described by Chao *et al*. (2008). Overnight cultures (OD_600_ = 1.0) of Xcc strains (2 μl) were spotted onto NYG plates containing 2% (wt/vol) glucose. Results were observed after incubation at 28 °C for 4 days.

### 4.16 Purification of (His)_6_‐RsmA protein

The (His)_6_‐RsmA_Xcc_ production from *E. coli* BL2506 (Table S1) was cultured at 37 °C with shaking at 200 rpm. When cell density reached about OD_600_ = 0.6, isopropyl‐β‐d‐thiogalactopyranoside (IPTG) was added at a final concentration of 1 mM and the culture was further incubated at 37 °C for 3 hr. Cells were then harvested by centrifugation at 4 °C for 20 min at 3,500 × g and resuspended in phosphate‐buffered saline (137 mM NaCl, 2.7 mM KCl, 10 mM Na_2_HPO_4_, 1.8 mM KH_2_PO_4_, pH 7.4) and ruptured by sonication, followed by centrifugation at 12,000 × g at 4 °C for 15 min. (His)_6_‐RsmA was purified from the cell‐free extract by using a 6 × His‐tagged Protein Purification Kit (CWBIO). Purified (His)_6_‐RsmA protein was quantified using a BCA protein quantification kit (Beyotime Biotechnology). The purified (His)_6_‐RsmA protein was stored at −80 °C.

### 4.17 RNA gel mobility shift assay

RNA gel mobility shift assay was carried out as described previously (Yakhnin *et al*., 2012) with some minor modifications. DNA templates for generating sRX061L, sRX061S, 1332Full, 1332ORF, and SRC181 RNA were produced by PCR amplification using the genomic DNA of Xcc strain 8,004 as template and the primer pairs 061S‐iv‐F/061S‐iv‐R, 061L‐iv‐F/061L‐iv‐R, 1332Full‐iv‐F/1332Full‐iv‐R, 1332ORF‐iv‐F/1332ORF‐iv‐R, and SRC181‐iv‐F/SRC181‐iv‐R (Table S7), respectively. DIG‐labelled sRX061L, sRX061S, 1332Full, 1332ORF, and SRC181 were generated by in vitro transcription using a DIG RNA labelling kit. The in vitro binding of the unlabelled (His)_6_‐RsmA protein with the DIG‐labelled RNA was performed using the EMSA/Gel‐Shift Kit (Beyotime Biotechnology). (His)_6_‐RsmA protein was mixed with 1 nM biotin‐labelled RNA in 9 μl of binding buffer (40 mM Tris‐HCl, 150 mM KCl, 10 mM MgCl_2_, 1 mM dithiothreitol, 0.01% Triton X‐100, pH 7.5) for 30 min at 28 °C to allow the formation of protein–RNA complex. After addition of 1 μl of colourless EMSA/Gel‐Shift loading buffer, the sample was immediately separated by 6% native PAGE and then transferred to a positively charged nylon membrane (Roche Applied Science). Signal bands were detected according to the EMSA/Gel‐Shift Kit instructions and visualized with an ImageQuant LAS 500 imager (GE Healthcare).

### 4.18 Construction of the Xcc strains ΔP_1332_, CΔP_1332_, and Δ3′UTR

The *XC1332* promoter deletion strain ΔP_1332_ was constructed by using the unmarked deletion mutant construction method described by Ried and Collmer (1987). Briefly, a 643‐bp DNA fragment upstream of the *XC1332* promoter and a 683‐bp DNA fragment downstream of the promoter were amplified by PCR using the genomic DNA of Xcc strain 8,004 as template and the primer pairs DP1332L‐F/DP1332L‐R and DP1332R‐F/DP1332R‐R (Table S7), respectively. After confirmation by sequencing, the two DNA fragments were linked together by fusion PCR and cloned into the suicide plasmid pK18mobsacB, generating the recombinant plasmid pKP1332UD (Table S1). The plasmid pKP1332UD was introduced from *E. coli* DH5α into Xcc 8,004 by triparental conjugation using the helper plasmid pRK2073 (Table S1). Single‐crossover integration mutant transconjugants were selected on NYG plates containing rifampicin and kanamycin, and further confirmed by their sucrose‐sensitive phenotype. One of the confirmed mutant transconjugants was grown overnight in NYG medium, then diluted and plated onto NYG plates containing rifampicin and 5% sucrose. After incubation at 28 °C for 3 days, sucrose‐resistant/kanamycin‐sensitive colonies were screened and checked for the deletion of the *XC1332* promoter by PCR using the total DNA of the colonies as template and the primer pair DP1332con‐F/DP1332con‐R (Table S7). The confirmed promoter deletion mutant was named ΔP_1332_ (Table S1) and used for further study.

The strain CΔP_1332_ was constructed by introducing the XC1332‐expressing plasmid pL1332 into strain ΔP_1332_ by triparental conjugation. To construct pL1332, a 525‐bp DNA fragment containing the entire *XC1332* gene was amplified by PCR using the genomic DNA of strain 8,004 as template and C1332F/C1332R (Table S7) as primers. After confirmation by sequencing, the DNA fragment was cloned into the promoterless cloning sites of the vector pLAFR6 to generate the recombinant plasmid pL1332 (Table S1).

The *XC1332* 3′UTR deletion strain Δ3′UTR was constructed by the same method for the construction of ΔP_1332_ except using different primers, which are listed in Table S7.

## Supporting information


**FIGURE S1** The visualized mapping patterns of the 676 identified target transcripts (TTs). “Start” and “End” indicate the putative transcription start and end positions in the genome of Xcc strain 8,004. “Length” indicates the length of the TT. RPKM represents the value of the reads per kilobases per million reads. The vertical ordinate represents the number of mapped reads and the horizontal ordinate indicates the genome position and genetic organization of the mapped region

**FIGURE S2** Sequence alignments of Xcc SRCs homologues using the Vector NTI program. The result reveals that the small RNAs SRC017/SRC434/SRC565 and SRC020/SRC291 are homologous

**FIGURE S3** Detection of the expression of sRNAs in Xcc by northern blotting. The bacterial cells of Xcc wild‐type strain 8,004 were cultured in the minimal medium MMX to mid‐log phase and total RNAs were isolated from the cells. Three micrograms of total RNA was separated by PAGE and transferred to a positively charged nylon membrane. After UV‐crosslinking, the membrane was hybridized with a DIG‐labelled RNA probe at 68 °C for 8 hr and then signal bands were detected. To ensure the accuracy in evaluation of the size of signal bands, DIG‐labelled RNA molecular weight marker (M) was loaded in each PAGE gel. The filled triangle inside the northern blotting result picture indicates the position corresponding to the size of the target SRC predicted by RNA‐Seq. Above the northern blotting result picture is the visualized mapping pattern of the corresponding SRC. The pink arrow indicates the transcriptional direction of the SRC. The lowermost arrow indicates the protein‐coding gene and its transcriptional orientation and the gene’s ID is shown inside the arrow. The *y* axis represents the number of the mapped reads. RPKM, reads per kilo bases per million reads

**FIGURE S4** The predicted secondary structures of the sRNAs detectable by northern blotting. The secondary structures were predicted by using M‐fold online software (http://unafold.rna.albany.edu/?q=mfold/RNA‐Folding‐Form) (Zuker, 2003). One representative structure for each sRNA is shown

**FIGURE S5** Detection of the expression level of target sRNA in the pBBad‐carrying wild‐type strain (WT/pB) and its overexpression strain (OE‐SR) by semiquantitative RT‐PCR. Xcc cells were cultured in the minimal medium MMX to mid‐log phase. Total RNAs from the cells were isolated and contaminated DNA was removed. cDNAs were synthesized from the RNAs by reverse transcription and used as template for PCR amplification using the sRNA gene‐specific primers. The PCR was performed with a cycler using the following cycle parameters: 30 cycles of 94 °C for 15 s, 60 °C for 15 s, and 72 °C for 15 s. The amplification products were analysed in 1.2% agarose gels and signal bands were quantified using GelQuant.NET software provided by biochemlabsolutions.com. Values under each gel image are the relative signal density of the corresponding PCR product. The 16S rRNA gene was used as the internal control to verify absence of significant variation at cDNA level in the two RNA samples

**FIGURE S6** Comparison of the virulence of the sRNA overexpression strains and the wild‐type strain. The virulence of the Xcc strains was tested in the leaves of Chinese radish (*Raphanus* *sativus* var. *radiculus*) using the leaf‐clipping method (An *et al*., 2017). Xcc strains were grown in NYG medium at 28 °С with shaking at 200 rpm for 15 hr. Cell concentration was adjusted to OD_600_ = 0.001. Two to three fully expanded leaves per plant were inoculated by leaf clipping: the leaves were cut with scissors dipped in the bacterial suspensions. Lesion length was measured 10 days postinoculation. Fifty leaves were inoculated for each strain in each independent experiment. The experiment was repeated three times. Data are the mean ± *SD* from a representative experiment. Similar results were obtained in two other independent experiments. The asterisks above the column represent the significant difference (*p* = .01 by *t* test) versus the wild‐type strain

**FIGURE S7** Comparison of the growth of Xcc strains in the minimal medium MMX and the rich medium NYG. The pBBad‐carrying wild‐type strain WT/pB and the sRX061 overexpression strain WT/pB061 were separately inoculated in 5 ml of NYG medium and incubated overnight at 28 °С in a shaking incubator at 200 rpm. The cells were washed with water and resuspended to a concentration of OD_600_ = 1.0, and then 2 μl of the resuspension for each strain was spotted onto NYG and MMX plates, respectively, and incubated at 28 °С. Results were observed at 48 hr (for NYG plate) or 96 hr (for MMX plate) of incubation. Each experiment was repeated three times and similar results were obtained

**FIGURE S8** Detection of sRX061S and sRX061L levels in the Xcc wild‐type strain 8,004 (WT), WT carrying an empty pBBad (WT/pB), WT carrying a pBBad containing sRX061S‐coding sequence (WT/pB061S) or sRX061L‐coding sequence (WT/pB061L). The strains were cultured in the minimal medium MMX at 28 °C with shaking at 200 rpm for 24 hr. Total RNAs were isolated and 3 μg of the total RNAs were separated by 6% denaturing (8 M urea) polyacrylamide gel electrophoresis and transferred to a positively charged nylon membrane. After UV‐crosslinking, the membrane was hybridized with the DIG‐labelled sR061 probe. The hybridization was performed at 68 °C for 8 hr. Signal bands were detected using a DIG‐Northern Starter Kit, visualized with an ImageQuant LAS 500 imager, and quantified using GelQuant.NET software provided by biochemlabsolutions.com. 5S rRNA was probed as a loading control

**FIGURE S9** Identification of target transcripts (TTs) based on the visualized mapping pattern. The two representative graphical diagrams show the mapping details of the RNA‐Seq reads obtained from the 50–500 nt RNA libraries in the genome of Xcc strain 8,004. (a) A typical graphical diagram of a mapped region containing a TT. (b) A typical graphical diagram of a mapped region without a significant TT. The vertical ordinate represents the number of mapped reads and the horizontal ordinate indicates the genome position and genetic organization of the mapped region

**FIGURE S10** The effect of *XC1332* 3′‐UTR deletion on its mRNA accumulation. (a) The genetic organization of the *XC1332* 3′‐UTR deletion strain. (b) Detection of the *XC1332* mRNA level in wild‐type strain (WT) and the *XC1332* 3′‐UTR deletion strain Δ3′UTR by northern blotting using the P1332+061 probe. *S*trains were cultured in NYG medium at 28 °C with shaking at 200 rpm for 24 hr. Total RNAs were isolated and 3 μg was used for northern blotting with a DIG‐labelled RNA probe. The positions of the P1332+061 probe are shown as a thick red line in (a). 5S rRNA was probed as a loading control

**FIGURE S11** Phenotype analysis of ΔP_1332_ and CΔP_1332_ strains. (a) Black rot symptoms and the average lesion lengths (in mm) caused by wild‐type strain (WT), ΔP_1332_ and CΔP_1332_ on inoculated leaves of Chinese radish. Images were taken at 10 days postinoculation. Values under each leaf are the average lesion lengths (mean ± *SD*) from three repeats, each with 50 leaves. The same letter after the values of the average lesion lengths indicates no significant difference (*t* test, *p* < .01). (b) The hypersensitive response (HR) induced on nonhost plant pepper leaves (*Capsicum annuum* “ECW‐10R”) by Xcc strains. The photograph was taken 24 hr after inoculation. The result presented is from a representative experiment and similar results were obtained in two other independent experiments. The type III‐deficient mutant strain (ΔhrcV) was used as a negative control. (c) The result of a swarming motility assay. Two microlitres of overnight culture (OD_600_ = 1.0) of each strain was spotted onto the swarming assay plate, and results were observed and photographed after incubation at 28 °C for 3 days

**FIGURE S12** The effect of *rsmA* deletion on the accumulation of sRX061 and *XC1332* mRNA. (a) The genetic organization of *sRX061* locus and the specific probes used in this northern blotting. (b) Detection of the sRX061 and *XC1332* mRNA transcripts in wild‐type strain (WT) and *rsmA* deletion mutant (ΔrsmA) by northern blotting using a probe specific for the coding region of *XC1332* (P1332) and the sRX061‐specific probe (P061). *S*trains were cultured in NYG medium at 28 °C with shaking at 200 rpm for 24 hr. Total RNAs were isolated and 3 μg of these was used for northern blotting with a DIG‐labelled RNA probe. The positions of P1332 and P061 are shown as a thick red line in (a). 5S rRNA was probed as a loading control

**TABLE S1** Bacterial strains and plasmids used in this work

**TABLE S2** Distribution of the RNA‐Seq reads mapped on the genome of Xcc strain 8,004

**TABLE S3** A summary of the identified target transcripts (TTs) (676 in total)

**TABLE S4** A summary of the 612 identified sRNA candidates (SRCs)

**TABLE S5** The identified seven putative riboswitches

**TABLE S6** A summary of the 117 detectable sRNAs from 121 SRCs by northern blotting

**TABLE S7** Primers and DNA oligonucleotides used in this work

## Data Availability

The raw sequencing data have been deposited in the NCBI Sequence Read Archive at https://www.ncbi.nlm.nih.gov/sra, accession number PRJNA598782.
