# Supplementary material for: Genome‐wide screen and functional analysis in Xanthomonas reveal a large number of mRNA‐derived sRNAs, including the novel RsmA‐sequester RsmU
Source: Mol Plant Pathol. 2020 Sep 23;21(12):1573–90. doi: 10.1111/mpp.12997 (PMC7694677; doi:10.1111/mpp.12997)

Fig. S1. The visualized mapping patterns of the 676 identified target transcripts (TTs). “Start” and “End” indicate the putative transcription start and end positions in the genome of *Xcc* strain 8004. “Length” indicates the length of the TT. RPKM represents the value of the reads per kilo bases per million reads. The vertical ordinate represents the number of mapped reads and the horizontal ordinate indicates the genome position and genetic organization of the mapped region.

Transcript	Start	End	Length (bp)	RPKM	Inside gene	Overlap gene	Upstream gene	Downstream gene
TT001	1	115	115	148.1	Null	XC0001	Null	XC0002

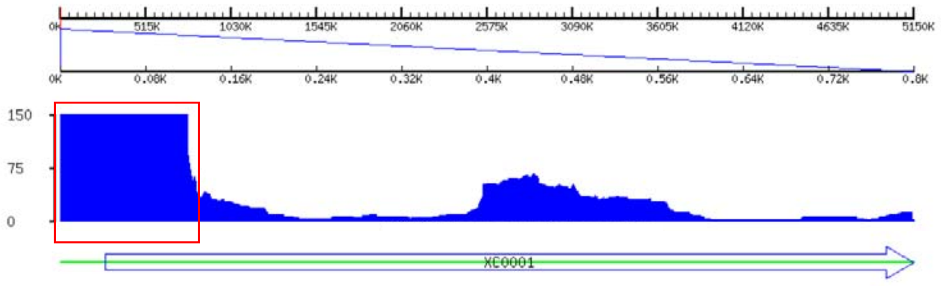

Transcript	Start	End	Length (bp)	RPKM	Inside gene	Overlap gene	Upstream gene	Downstream gene
TT002	3605	3701	97	263.6	Null	XC0003	XC0002	XC0004

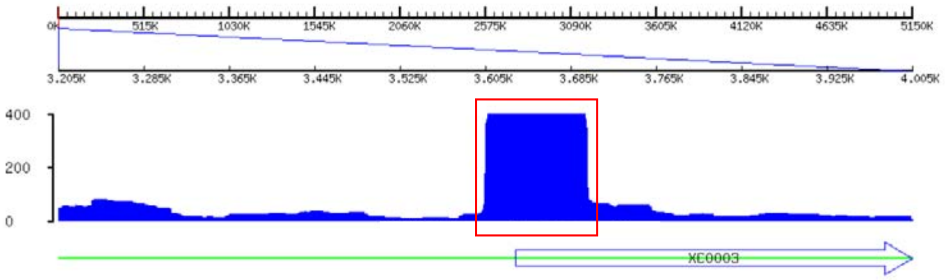

Transcript	Start	End	Length (bp)	RPKM	Inside gene	Overlap gene	Upstream gene	Downstream gene
TT003	8254	8318	65	201.0	Null	XC0006	XC0005	XC0007

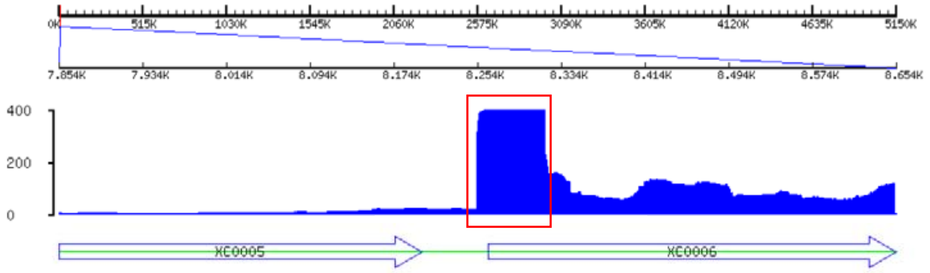

Transcript	Start	End	Length (bp)	RPKM	Inside gene	Overlap gene	Upstream gene	Downstream gene
TT004	10489	10568	80	223.4	Null	XC0008	XC0007	XC0009

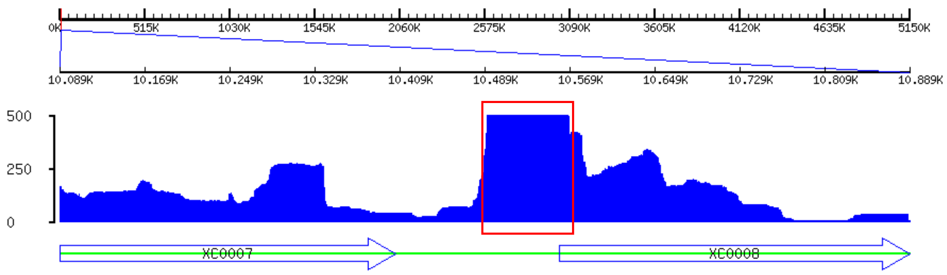

Transcript	Start	End	Length (bp)	RPKM	Inside gene	Overlap gene	Upstream gene	Downstream gene
TT005	11540	11606	67	100.3	XC0009	Null	XC0008	XC0010

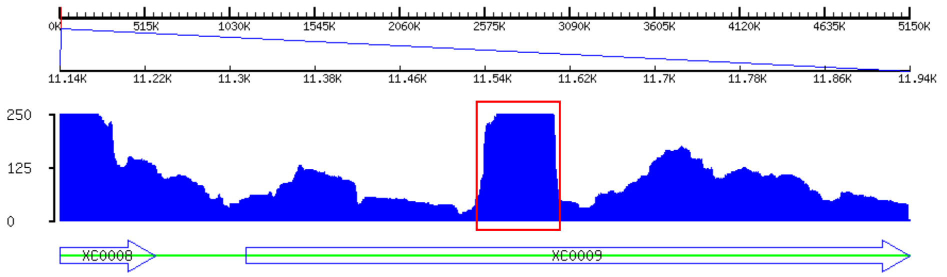

Transcript	Start	End	Length (bp)	RPKM	Inside gene	Overlap gene	Upstream gene	Downstream gene
TT006	12884	12975	92	221.4	Null	XC0011	XC0010	XC0012

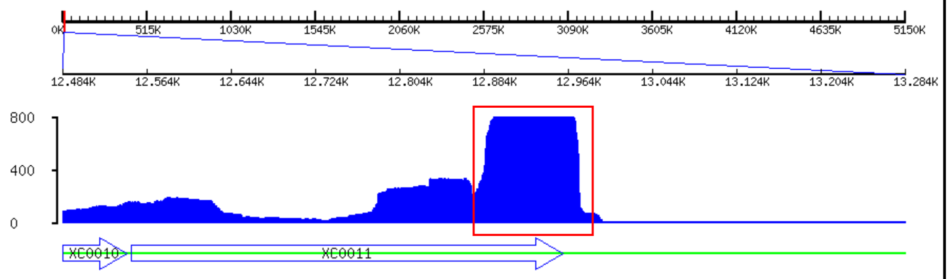

Transcript	Start	End	Length (bp)	RPKM	Inside gene	Overlap gene	Upstream gene	Downstream gene
TT007	19739	19801	63	45.7	XC0017	Null	XC0016	XC0018
TT008	20127	20185	59	80.5	XC0017	Null	XC0016	XC0018

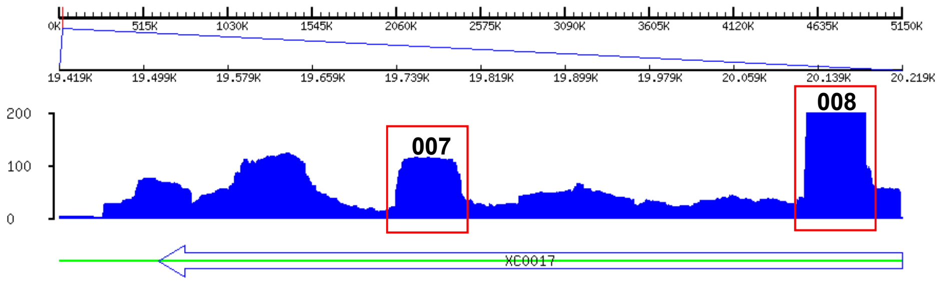

Transcript	Start	End	Length (bp)	RPKM	Inside gene	Overlap gene	Upstream gene	Downstream gene
TT009	44065	44146	82	212.9	Null	XC0032	XC0031	XC0033

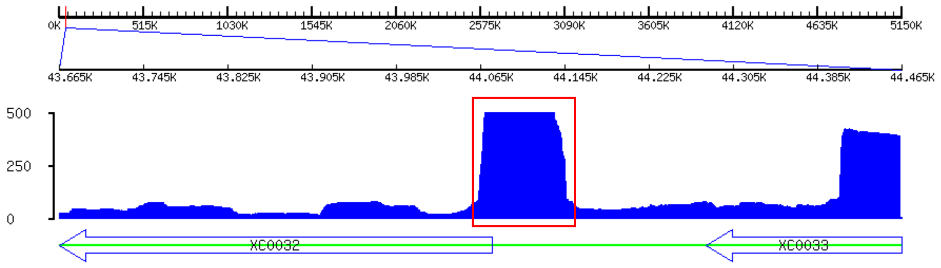

Transcript	Start	End	Length (bp)	RPKM	Inside gene	Overlap gene	Upstream gene	Downstream gene
TT010	44407	44484	77	95.1	XC0033	Null	XC0032	XC0034

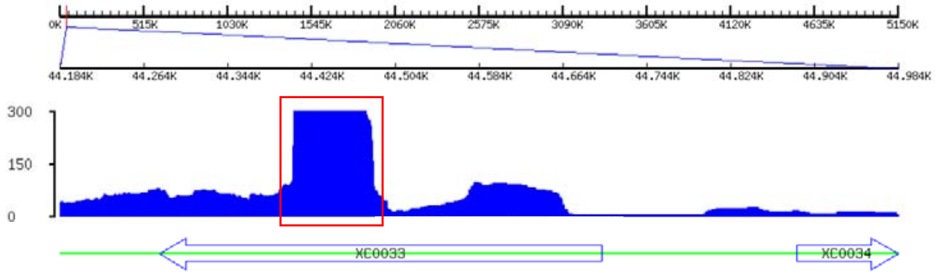

Transcript	Start	End	Length (bp)	RPKM	Inside gene	Overlap gene	Upstream gene	Downstream gene
TT011	73643	73693	51	117.4	Null	XC0057	XC0056	XC0058

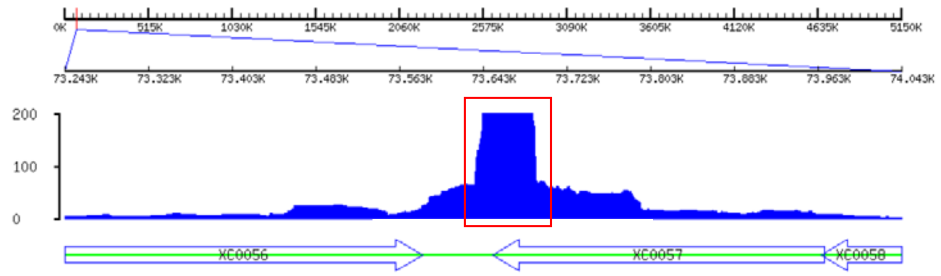

Transcript	Start	End	Length (bp)	RPKM	Inside gene	Overlap gene	Upstream gene	Downstream gene
TT012	77120	77175	56	69.7	Null	Null	XC0062	XC0063

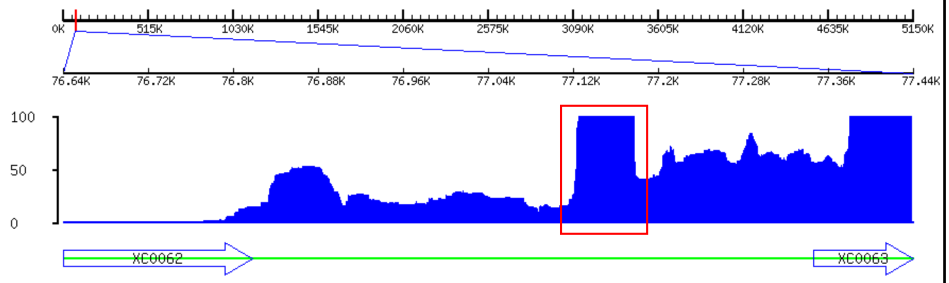

Transcript	Start	End	Length (bp)	RPKM	Inside gene	Overlap gene	Upstream gene	Downstream gene
TT013	77385	77474	90	107.1	XC0063	Null	XC0062	XC0064

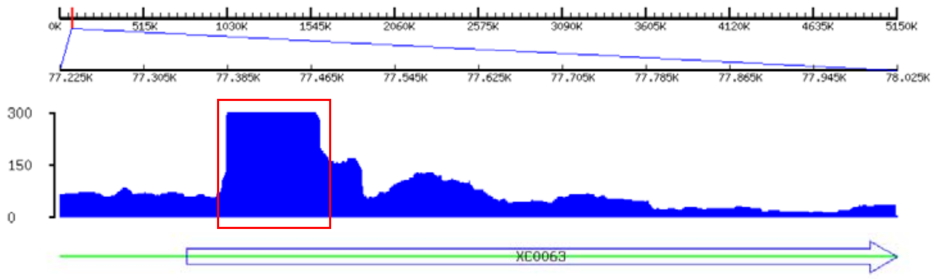

Transcript	Start	End	Length (bp)	RPKM	Inside gene	Overlap gene	Upstream gene	Downstream gene
TT014	79055	79114	60	40.4	Null	Null	XC0063	XC0064

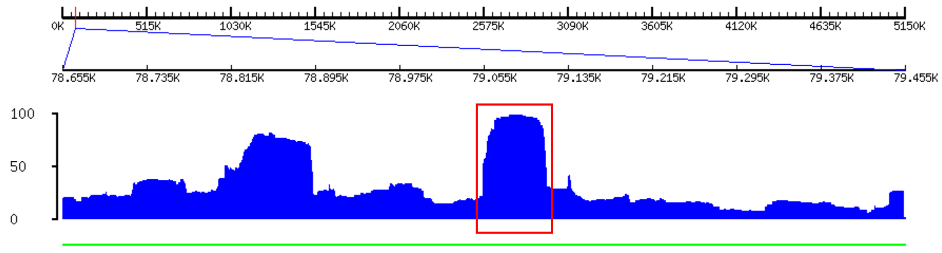

Transcript	Start	End	Length (bp)	RPKM	Inside gene	Overlap gene	Upstream gene	Downstream gene
TT015	79664	79718	55	75.8	Null	Null	XC0064	XC0065

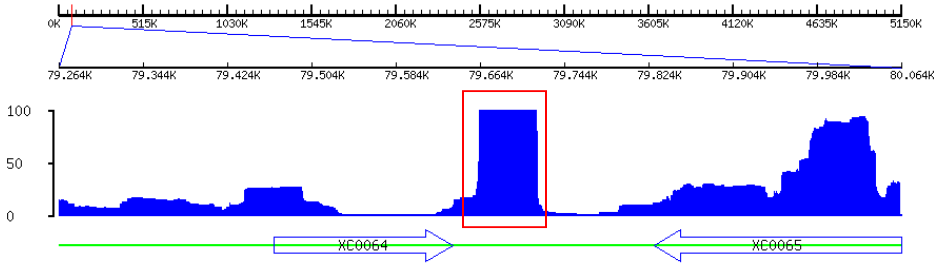

Transcript	Start	End	Length (bp)	RPKM	Inside gene	Overlap gene	Upstream gene	Downstream gene
TT016	81165	81218	54	59.7	Null	Null	XC0065	XC0066

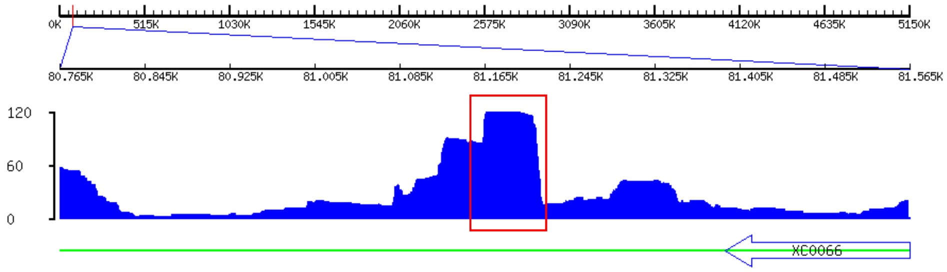

Transcript	Start	End	Length (bp)	RPKM	Inside gene	Overlap gene	Upstream gene	Downstream gene
TT017	83883	83949	67	389.9	Null	Null	XC0068	XC0069

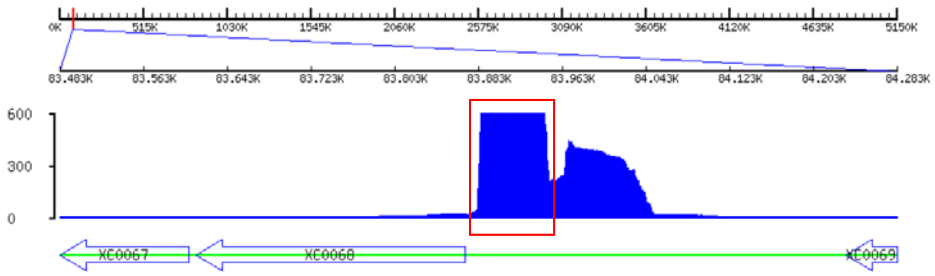

Transcript	Start	End	Length (bp)	RPKM	Inside gene	Overlap gene	Upstream gene	Downstream gene
TT018	96245	96331	87	112.2	Null	XC0082	XC0081	XC0083
TT019	96345	96430	86	109.3	XC0082	Null	XC0081	XC0083

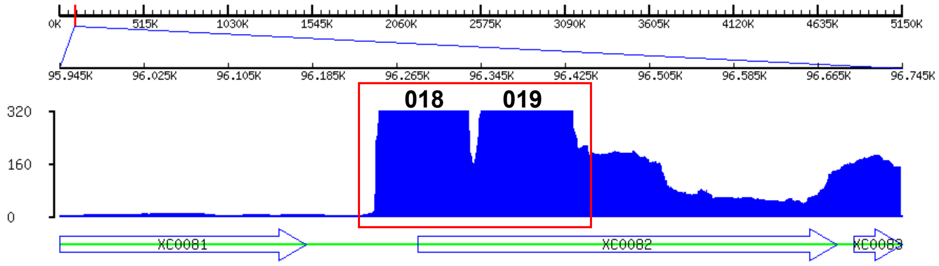

Transcript	Start	End	Length (bp)	RPKM	Inside gene	Overlap gene	Upstream gene	Downstream gene
TT020	113628	113830	203	261.8	Null	Null	XC0096	XC0097

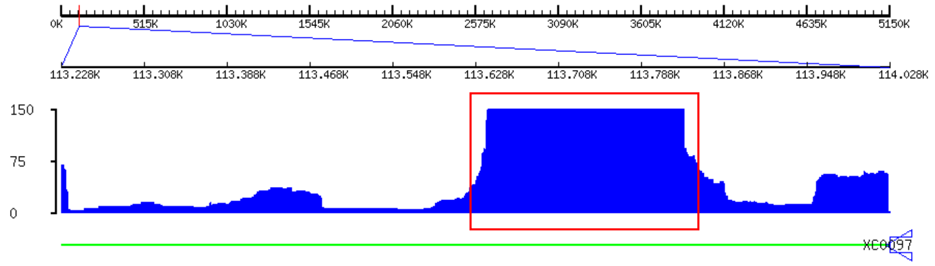

Transcript	Start	End	Length (bp)	RPKM	Inside gene	Overlap gene	Upstream gene	Downstream gene
TT021	141901	141975	75	59.5	Null	XC0118	XC0117	XC0119

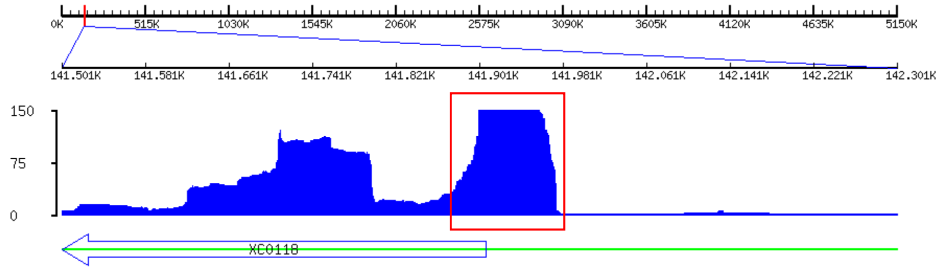

Transcript	Start	End	Length (bp)	RPKM	Inside gene	Overlap gene	Upstream gene	Downstream gene
TT022	142449	142507	59	75.2	Null	XC0119	XC0118	XC0120

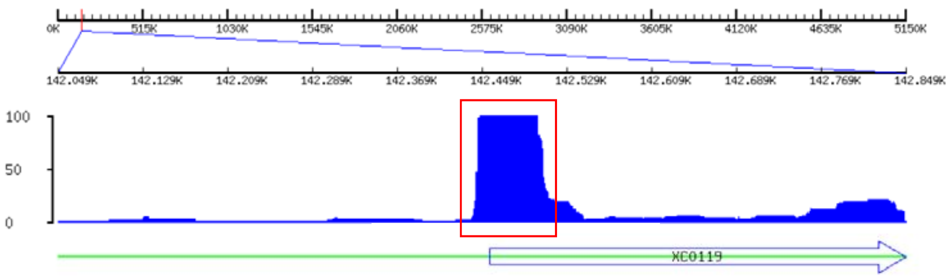

Transcript	Start	End	Length (bp)	RPKM	Inside gene	Overlap gene	Upstream gene	Downstream gene
TT023	146692	146747	56	88.6	Null	Null	XC0122	XC0123

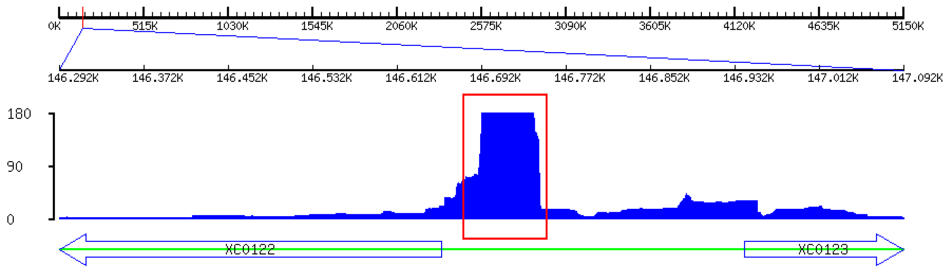

Transcript	Start	End	Length (bp)	RPKM	Inside gene	Overlap gene	Upstream gene	Downstream gene
TT024	165210	165280	71	61.1	Null	XC0136	XC0134	XC0137

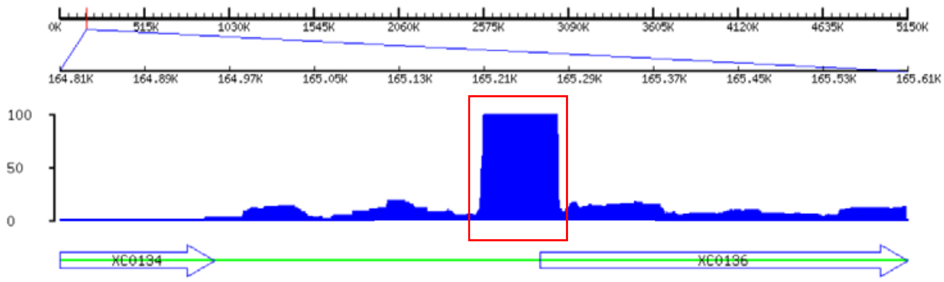

Transcript	Start	End	Length (bp)	RPKM	Inside gene	Overlap gene	Upstream gene	Downstream gene
TT025	174860	174916	57	177.8	Null	Null	XC0140	XC0141

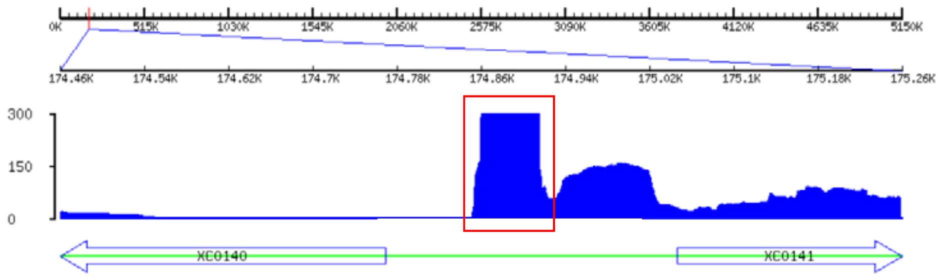

Transcript	Start	End	Length (bp)	RPKM	Inside gene	Overlap gene	Upstream gene	Downstream gene
TT026	214031	214120	90	108.8	Null	XC0171	XC0170	XC0172

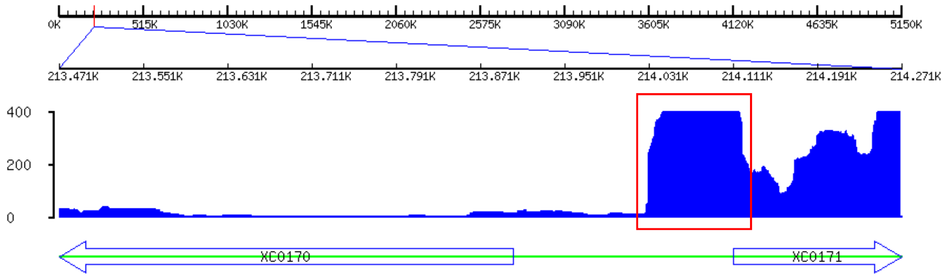

Transcript	Start	End	Length (bp)	RPKM	Inside gene	Overlap gene	Upstream gene	Downstream gene
TT027	214270	214333	64	224.9	XC0171	Null	XC0170	XC0172

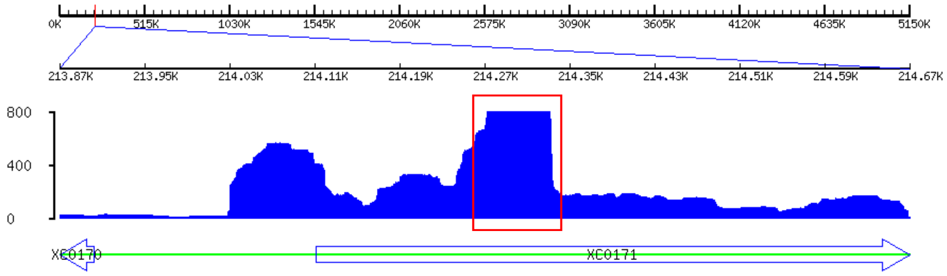

Transcript	Start	End	Length (bp)	RPKM	Inside gene	Overlap gene	Upstream gene	Downstream gene
TT028	225413	225476	64	109.8	Null	Null	XC0179	XC0180

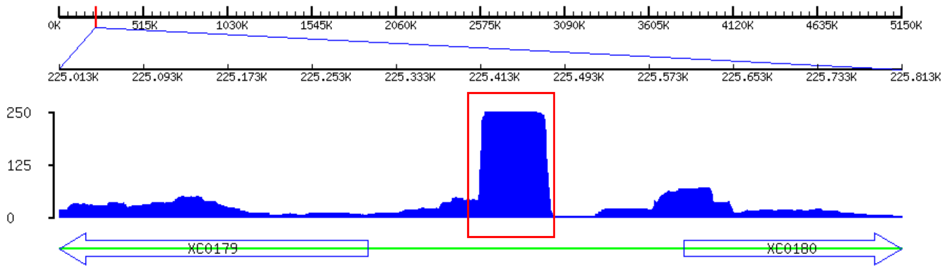

Transcript	Start	End	Length (bp)	RPKM	Inside gene	Overlap gene	Upstream gene	Downstream gene
TT029	230567	230759	193	170.9	Null	XC185/186	XC0184	XC0187

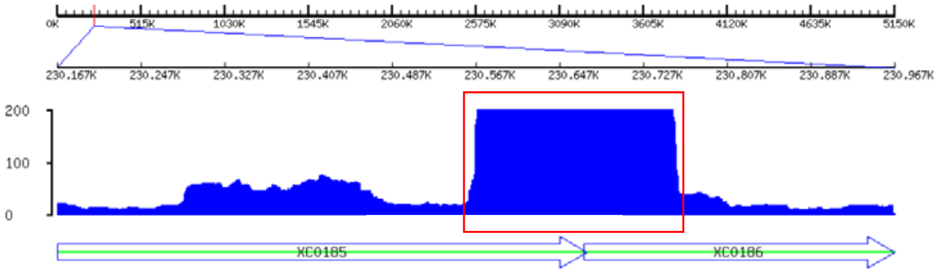

Transcript	Start	End	Length (bp)	RPKM	Inside gene	Overlap gene	Upstream gene	Downstream gene
TT030	239805	239882	78	42.7	Null	Null	XC0191	XC0192

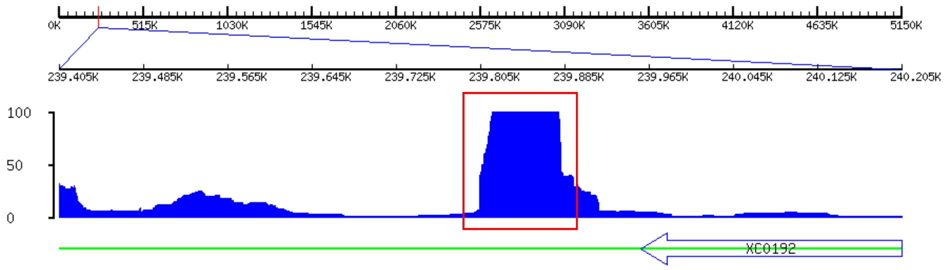

Transcript	Start	End	Length (bp)	RPKM	Inside gene	Overlap gene	Upstream gene	Downstream gene
TT031	257175	257244	70	126.5	Null	XC0207	XC0206	XC0208

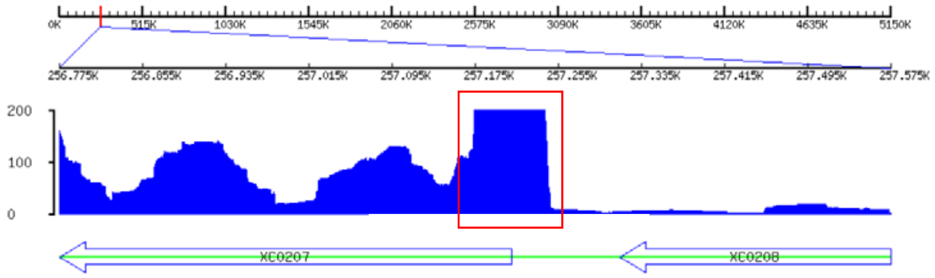

Transcript	Start	End	Length (bp)	RPKM	Inside gene	Overlap gene	Upstream gene	Downstream gene
TT032	262750	262802	53	287.8	Null	Null	XC0214	XC0215
TT033	262819	263474	655	302.1	XC0215	Null	XC0214	XC0216

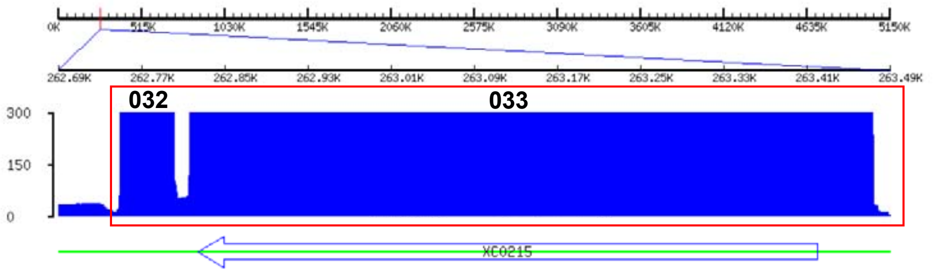

Transcript	Start	End	Length (bp)	RPKM	Inside gene	Overlap gene	Upstream gene	Downstream gene
TT034	263377	263474	98	1235.9	Null	XC0215	XC0214	XC0216

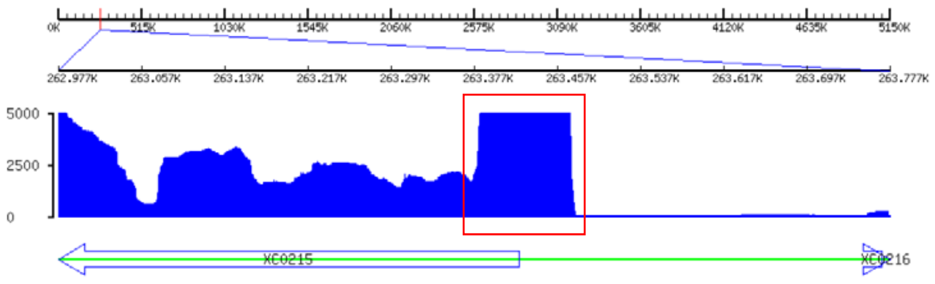

Transcript	Start	End	Length (bp)	RPKM	Inside gene	Overlap gene	Upstream gene	Downstream gene
TT035	278860	279124	264	203.7	XC0230	Null	XC0229	XC0231

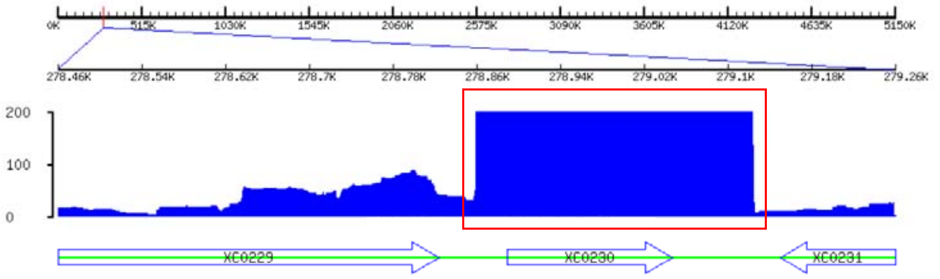

Transcript	Start	End	Length (bp)	RPKM	Inside gene	Overlap gene	Upstream gene	Downstream gene
TT036	278860	278923	64	639.9	Null	XC0230	XC0229	XC0231

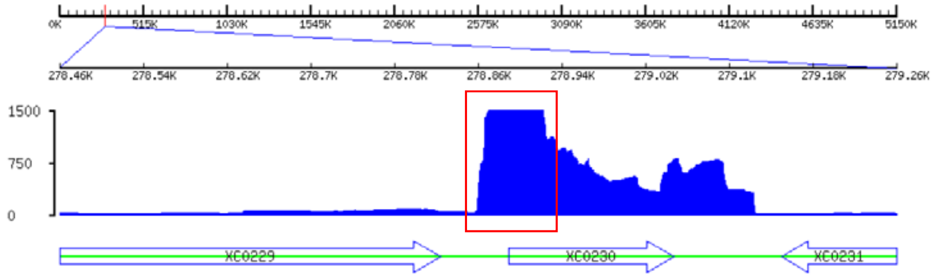

Transcript	Start	End	Length (bp)	RPKM	Inside gene	Overlap gene	Upstream gene	Downstream gene
TT037	292444	292545	102	360.4	Null	Null	XC0241	XC0242

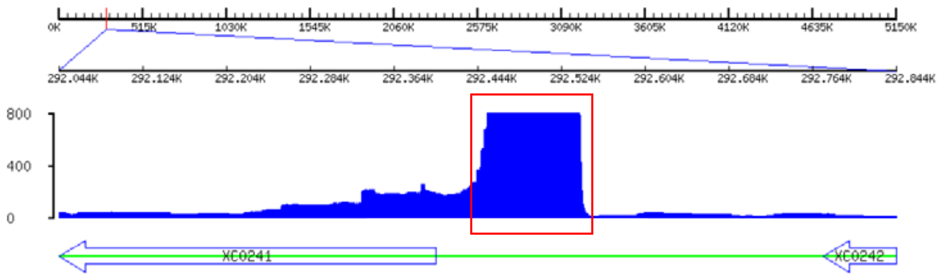

Transcript	Start	End	Length (bp)	RPKM	Inside gene	Overlap gene	Upstream gene	Downstream gene
TT038	318332	318488	157	41.6	Null	XC0264	XC0263	XC0265

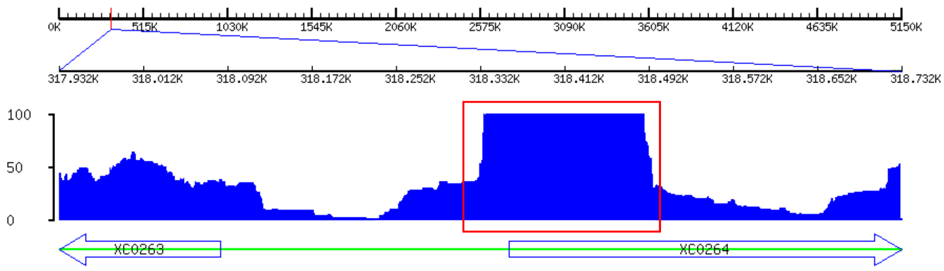

Transcript	Start	End	Length (bp)	RPKM	Inside gene	Overlap gene	Upstream gene	Downstream gene
TT039	328623	328686	64	1243.9	XC0274	Null	XC0273	XC0275

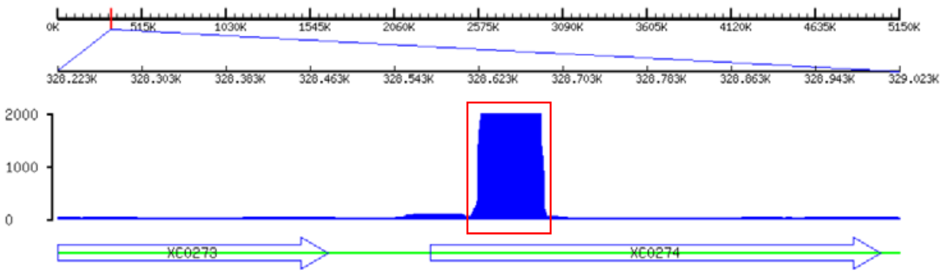

Transcript	Start	End	Length (bp)	RPKM	Inside gene	Overlap gene	Upstream gene	Downstream gene
TT040	330632	330722	90	1594.6	XC0277	Null	XC0275	XC0278
TT041	330757	330846	89	109.31	XC0278	Null	XC0275	XC0278

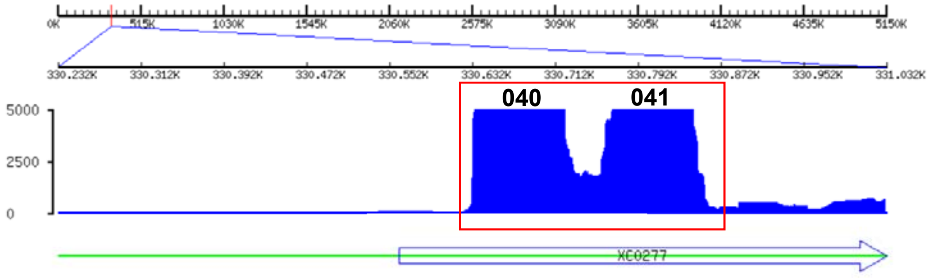

Transcript	Start	End	Length (bp)	RPKM	Inside gene	Overlap gene	Upstream gene	Downstream gene
TT042	337275	337739	464	457.1	XC0284	Null	XC0283	XC0285

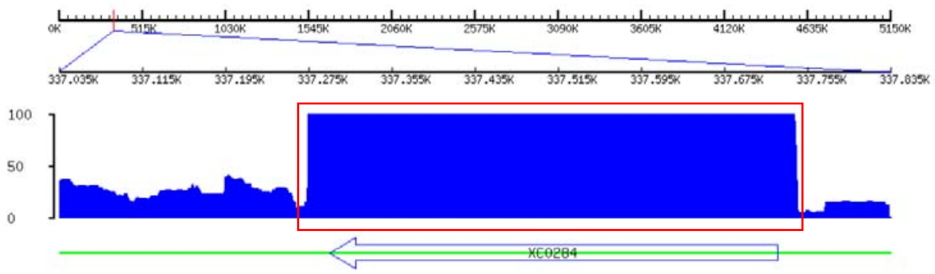

Transcript	Start	End	Length (bp)	RPKM	Inside gene	Overlap gene	Upstream gene	Downstream gene
TT043	337275	337504	230	711.6	Null	XC0284	XC0283	XC0285

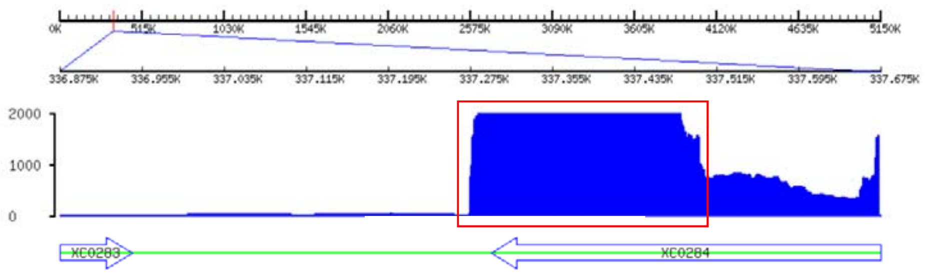

Transcript	Start	End	Length (bp)	RPKM	Inside gene	Overlap gene	Upstream gene	Downstream gene
TT044	337655	337739	85	377.7	Null	XC0284	XC0283	XC0285

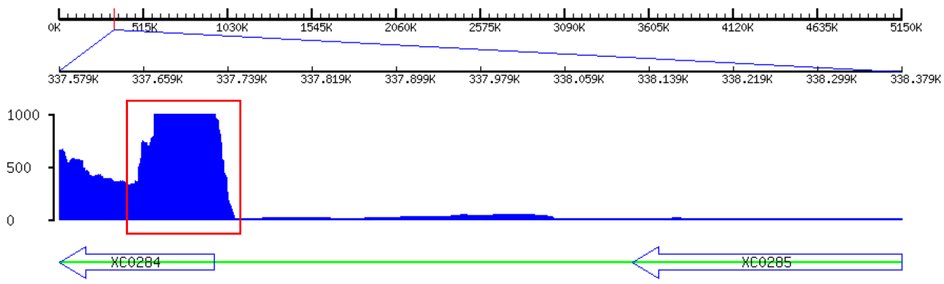

Transcript	Start	End	Length (bp)	RPKM	Inside gene	Overlap gene	Upstream gene	Downstream gene
TT045	346558	346643	86	100.7	XC0292	Null	XC0291	XC0293

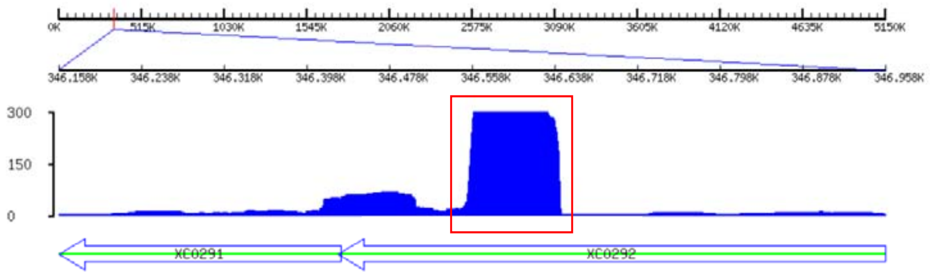

Transcript	Start	End	Length (bp)	RPKM	Inside gene	Overlap gene	Upstream gene	Downstream gene
TT046	387979	388077	99	393.8	Null	XC0328	XC0327	XC0329

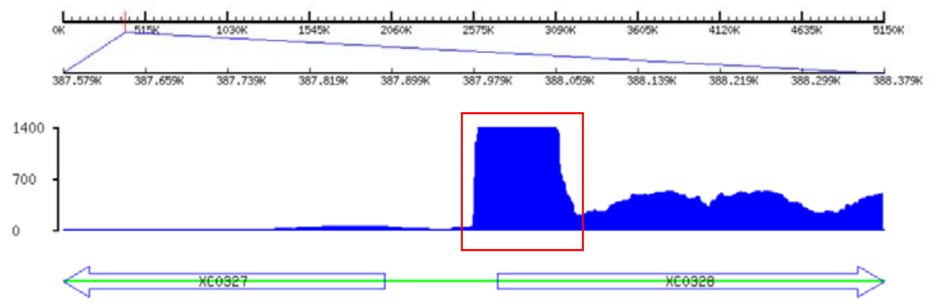

Transcript	Start	End	Length (bp)	RPKM	Inside gene	Overlap gene	Upstream gene	Downstream gene
TT047	388414	388470	57	140.9	XC0328	Null	XC0327	XC0329

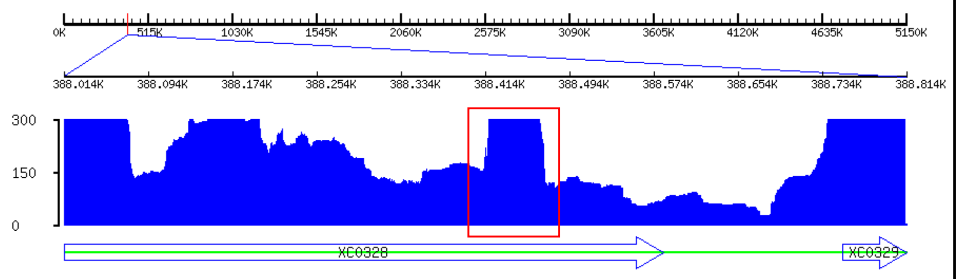

Transcript	Start	End	Length (bp)	RPKM	Inside gene	Overlap gene	Upstream gene	Downstream gene
TT048	391153	391204	52	159.1	Null	XC0331	XC0330	XC0332

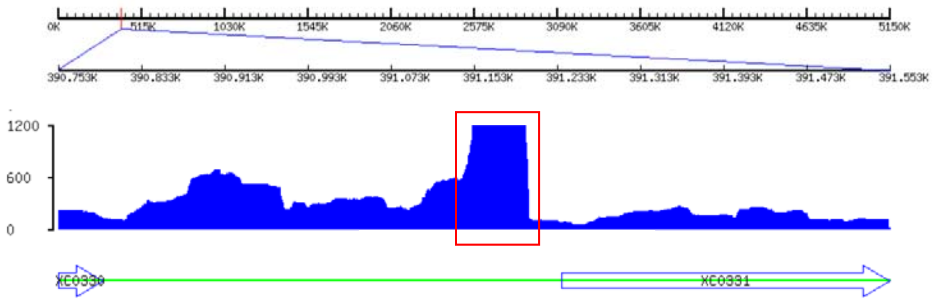

Transcript	Start	End	Length (bp)	RPKM	Inside gene	Overlap gene	Upstream gene	Downstream gene
TT049	391686	391747	62	74.1	XC0331	Null	XC0330	XC0332
TT050	392000	392058	59	77.1	XC0331	Null	XC0330	XC0332

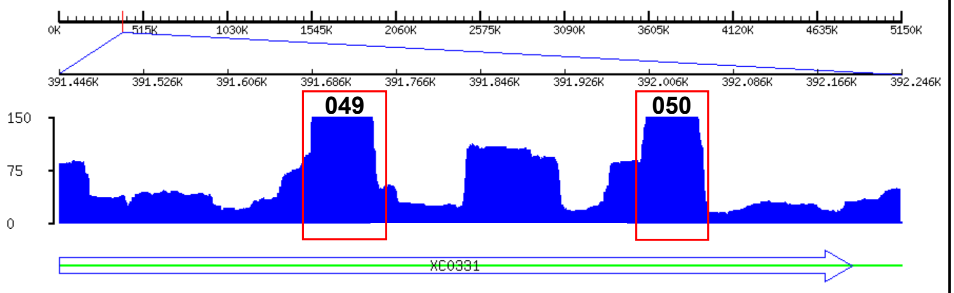

Transcript	Start	End	Length (bp)	RPKM	Inside gene	Overlap gene	Upstream gene	Downstream gene
TT051	395405	395472	68	2288.1	XC0333	Null	XC0332	XC0334

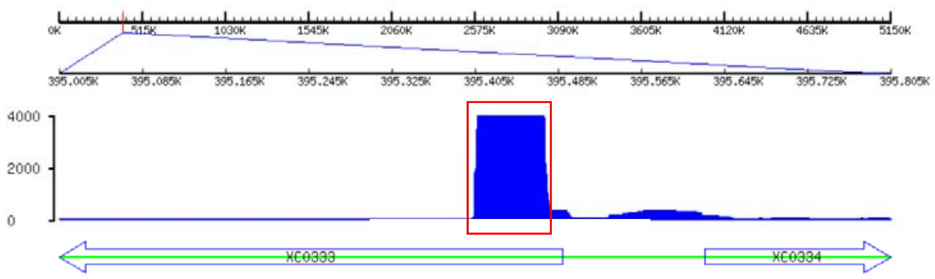

Transcript	Start	End	Length (bp)	RPKM	Inside gene	Overlap gene	Upstream gene	Downstream gene
TT052	410078	410152	74	17009.9	Null	Null	XC0350	XC0351

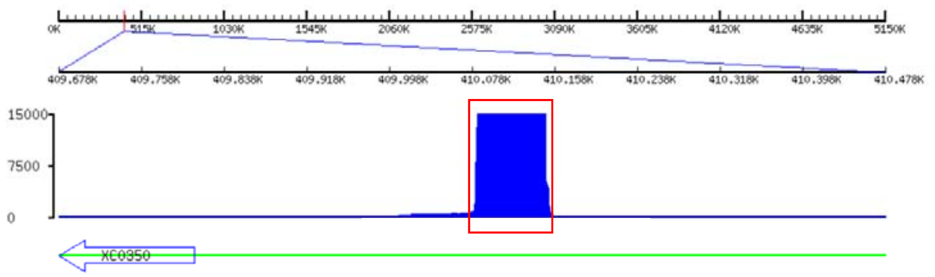

Transcript	Start	End	Length (bp)	RPKM	Inside gene	Overlap gene	Upstream gene	Downstream gene
TT053	413078	413167	90	38.7	XC0353	Null	XC0352	XC0354
TT054	413186	413278	93	29.3	XC0353	Null	XC0352	XC0354

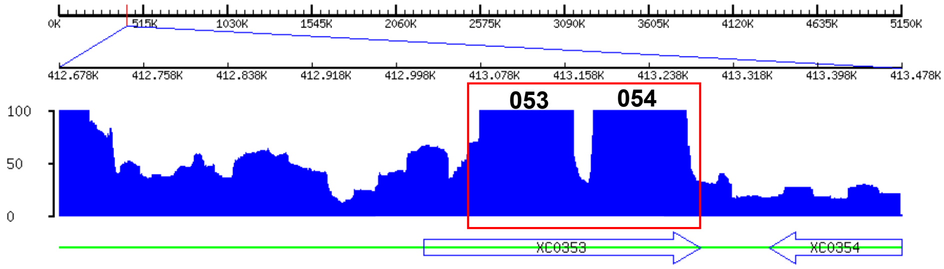

Transcript	Start	End	Length (bp)	RPKM	Inside gene	Overlap gene	Upstream gene	Downstream gene
TT055	413571	413631	61	91.3	Null	XC0354	XC0353	XC0355

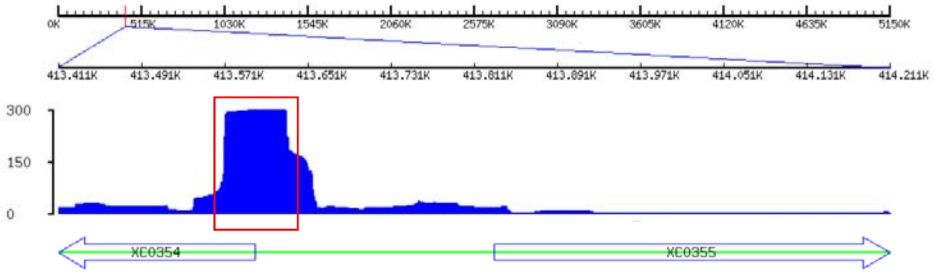

Transcript	Start	End	Length (bp)	RPKM	Inside gene	Overlap gene	Upstream gene	Downstream gene
TT056	419488	419557	70	158.5	Null	XC0360	XC0359	XC0361

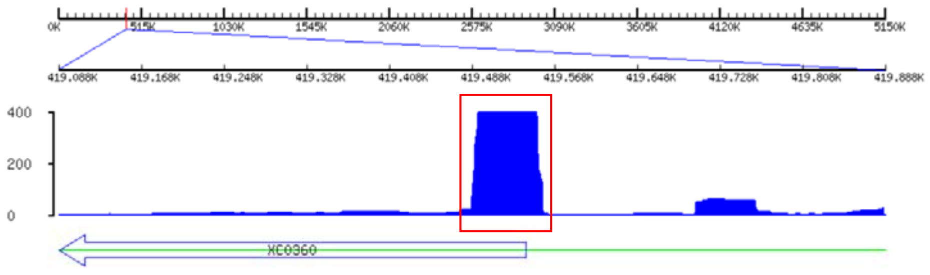

Transcript	Start	End	Length (bp)	RPKM	Inside gene	Overlap gene	Upstream gene	Downstream gene
TT057	447714	447766	53	276.5	XC0386	Null	XC0385	XC0387

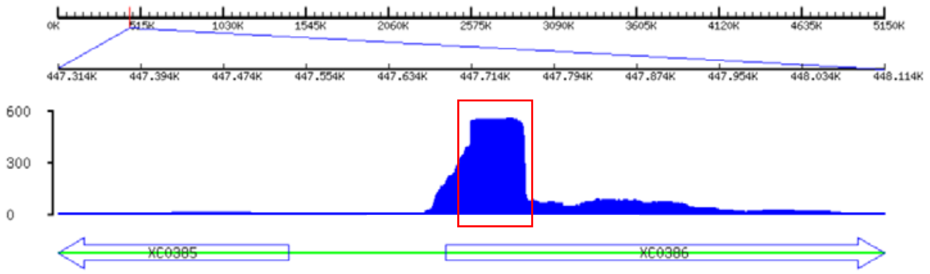

Transcript	Start	End	Length (bp)	RPKM	Inside gene	Overlap gene	Upstream gene	Downstream gene
TT058	453239	453295	57	627.5	Null	Null	XC0389	XC0390

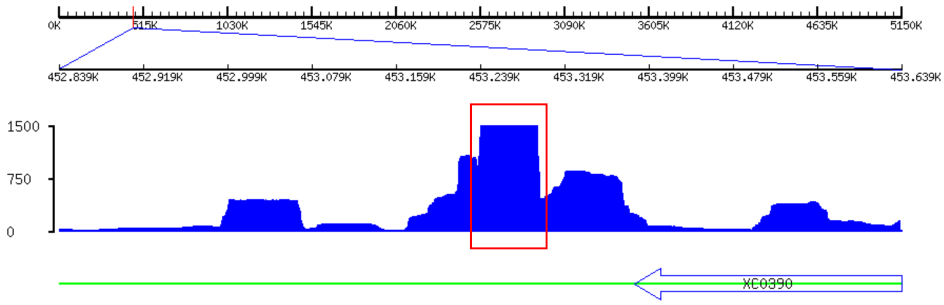

Transcript	Start	End	Length (bp)	RPKM	Inside gene	Overlap gene	Upstream gene	Downstream gene
TT059	453784	453855	72	197.5	XC0390	Null	XC0389	XC0391

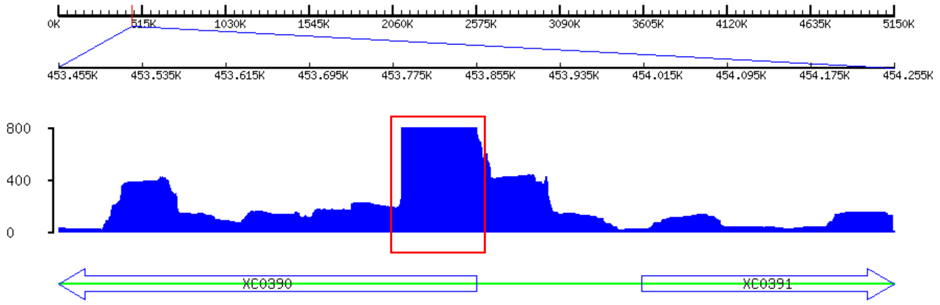

Transcript	Start	End	Length (bp)	RPKM	Inside gene	Overlap gene	Upstream gene	Downstream gene
TT060	456160	456216	78	64.1	Null	XC0394	XC0393	XC0395

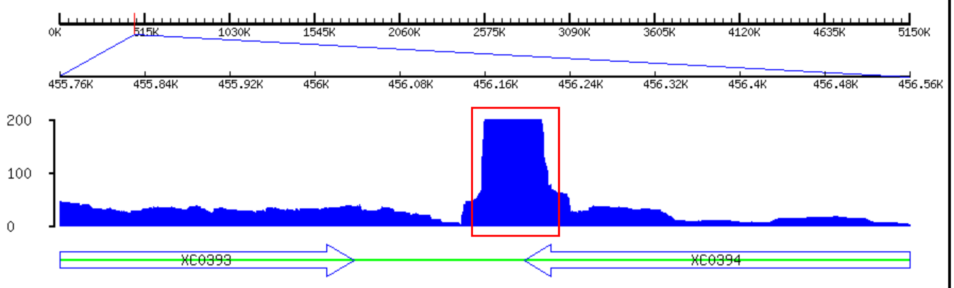

Transcript	Start	End	Length (bp)	RPKM	Inside gene	Overlap gene	Upstream gene	Downstream gene
TT061	465779	465848	88	555.1	Null	XC4333	XC0402	XC0403

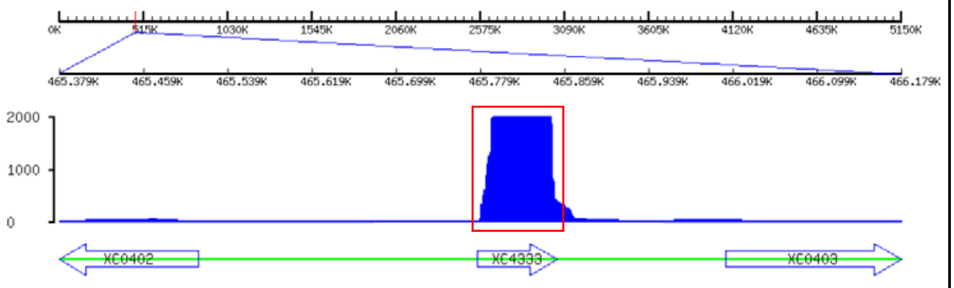

Transcript	Start	End	Length (bp)	RPKM	Inside gene	Overlap gene	Upstream gene	Downstream gene
TT062	471914	471986	73	102.1	Null	Null	XC0408	XC0409

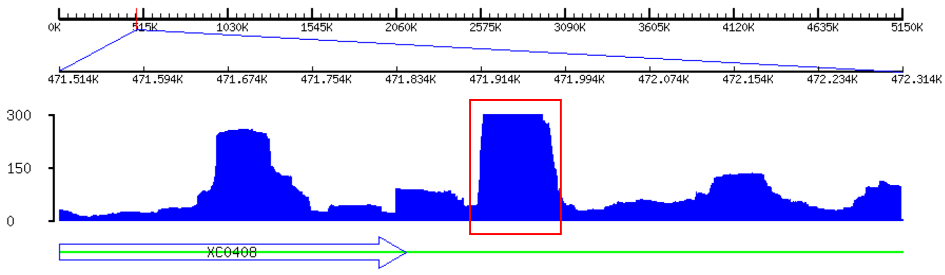

Transcript	Start	End	Length (bp)	RPKM	Inside gene	Overlap gene	Upstream gene	Downstream gene
TT063	480582	480641	60	2317.6	Null	XC0414	XC0413	XC0415

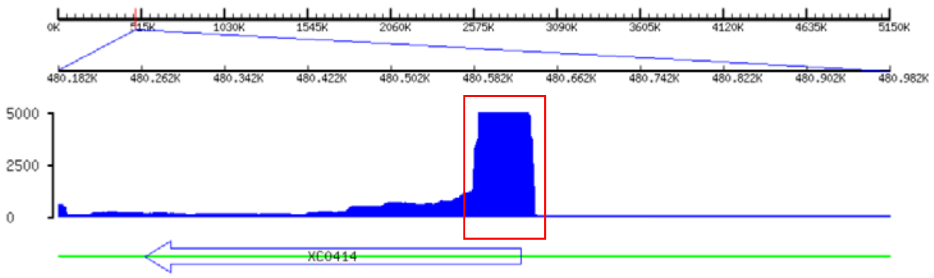

Transcript	Start	End	Length (bp)	RPKM	Inside gene	Overlap gene	Upstream gene	Downstream gene
TT064	486775	486840	66	105.1	Null	Null	XC0416	XC0417

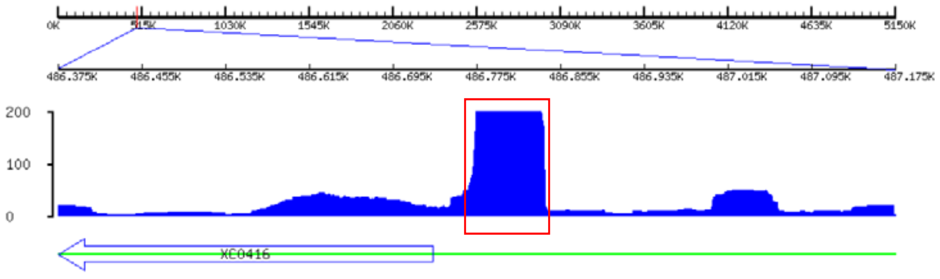

Transcript	Start	End	Length (bp)	RPKM	Inside gene	Overlap gene	Upstream gene	Downstream gene
TT065	488417	488478	62	180.9	XC0417	Null	XC0416	XC0418

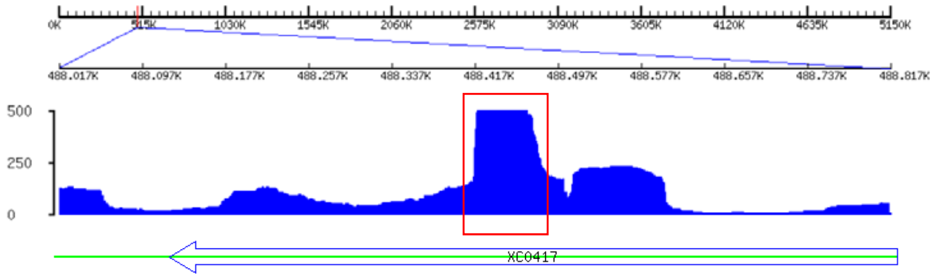

Transcript	Start	End	Length (bp)	RPKM	Inside gene	Overlap gene	Upstream gene	Downstream gene
TT066	491031	491085	55	112.3	XC0419	Null	XC0418	XC0420

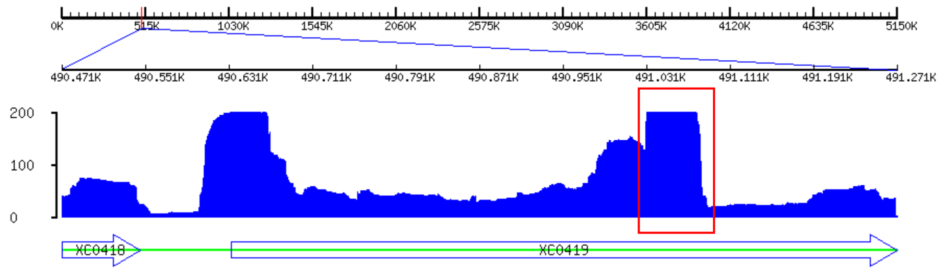

Transcript	Start	End	Length (bp)	RPKM	Inside gene	Overlap gene	Upstream gene	Downstream gene
TT067	491321	491385	65	53.1	XC0419	Null	XC0418	XC0420

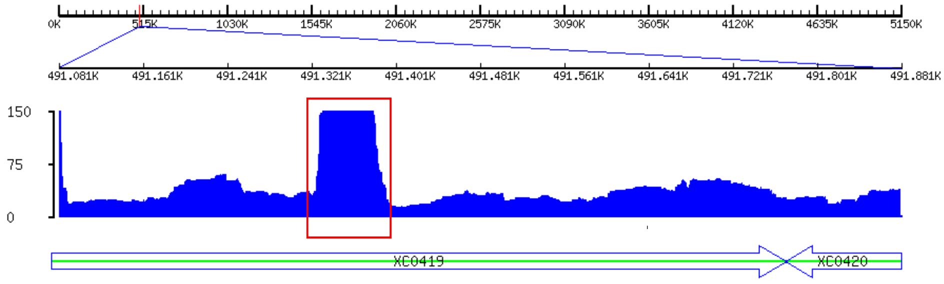

Transcript	Start	End	Length (bp)	RPKM	Inside gene	Overlap gene	Upstream gene	Downstream gene
TT068	522064	522154	91	137.8	Null	Null	XC0438	XC0439
TT069	522210	522633	423	301.9	Null	Null	XC0438	XC0439

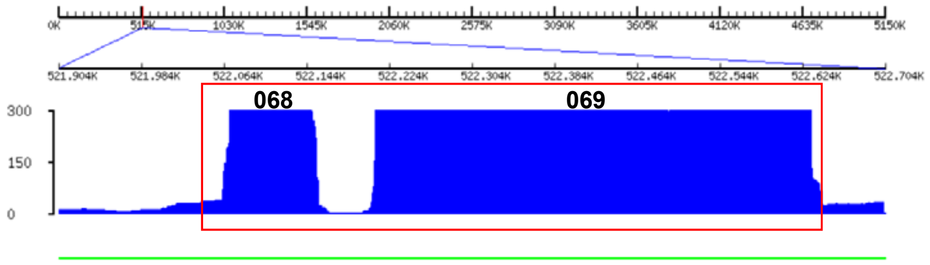

Transcript	Start	End	Length (bp)	RPKM	Inside gene	Overlap gene	Upstream gene	Downstream gene
TT070	526278	526332	55	76.2	XC0443	Null	XC0442	XC0444

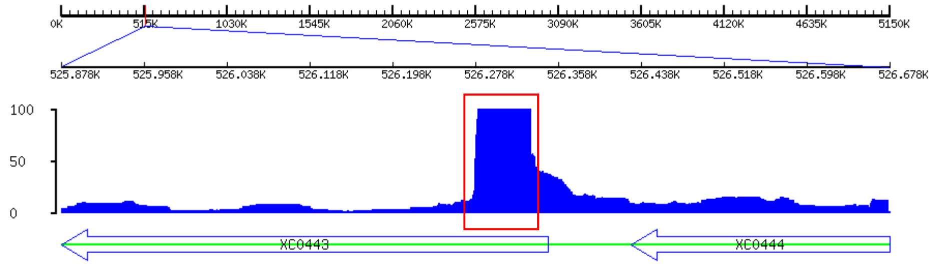

Transcript	Start	End	Length (bp)	RPKM	Inside gene	Overlap gene	Upstream gene	Downstream gene
TT071	551604	551667	64	190.7	Null	XC0463	XC0462	XC0464

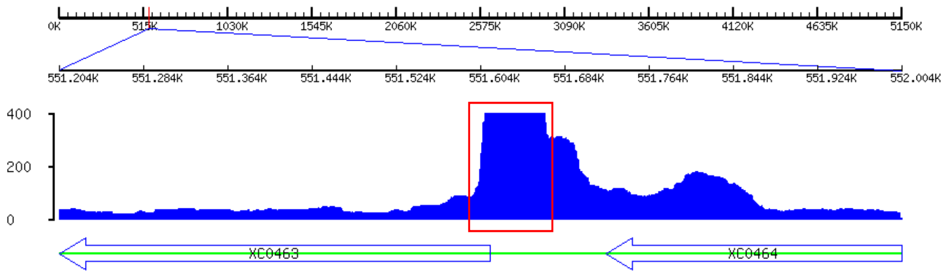

Transcript	Start	End	Length (bp)	RPKM	Inside gene	Overlap gene	Upstream gene	Downstream gene
TT072	574674	574724	51	88.9	XC0485	Null	XC0484	XC0486

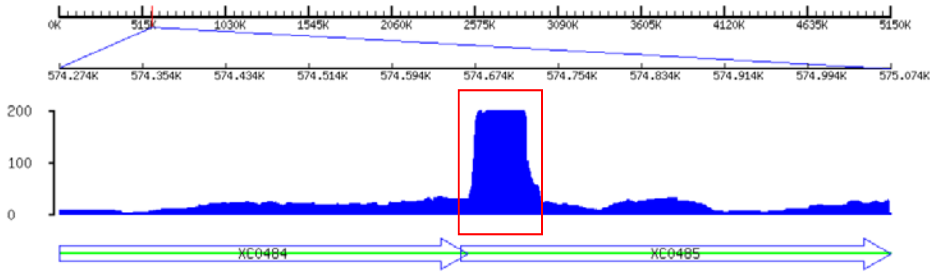

Transcript	Start	End	Length (bp)	RPKM	Inside gene	Overlap gene	Upstream gene	Downstream gene
TT073	576332	576410	79	200.4	XC0486	Null	XC0485	XC0487

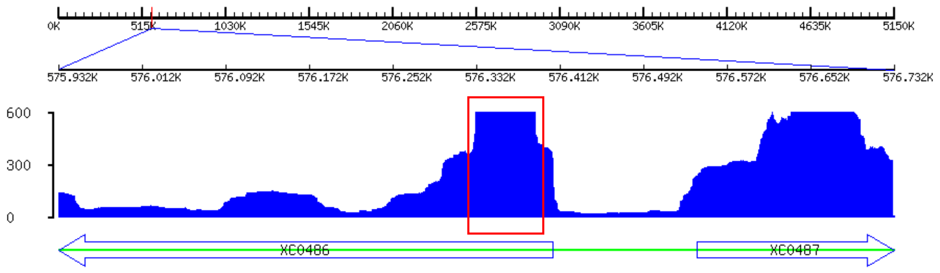

Transcript	Start	End	Length (bp)	RPKM	Inside gene	Overlap gene	Upstream gene	Downstream gene
TT074	578891	578983	93	1286.2	Null	XC0490	XC0489	XC0491

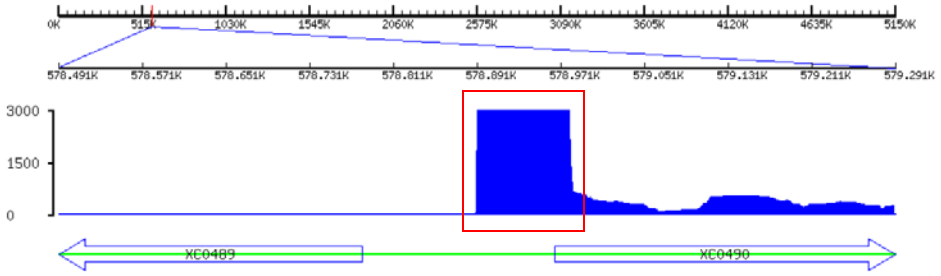

Transcript	Start	End	Length (bp)	RPKM	Inside gene	Overlap gene	Upstream gene	Downstream gene
TT075	580611	580674	63	2280.7	XC4334	Null	XC0491	XC0492
TT076	580741	580805	64	1500.3	XC4335	Null	XC0491	XC0492

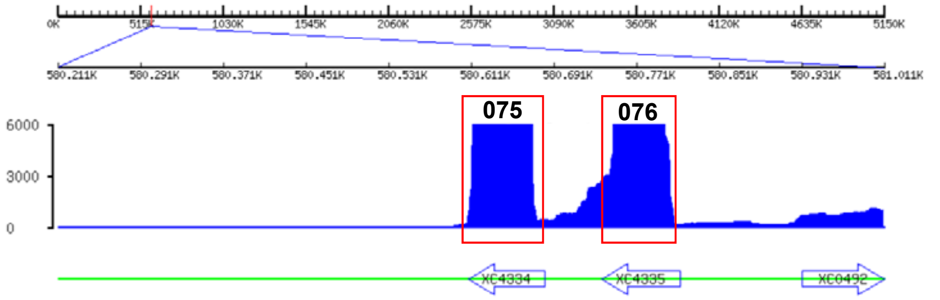

Transcript	Start	End	Length (bp)	RPKM	Inside gene	Overlap gene	Upstream gene	Downstream gene
TT077	581608	581674	67	107.4	Null	XC0493	XC0492	XC0494

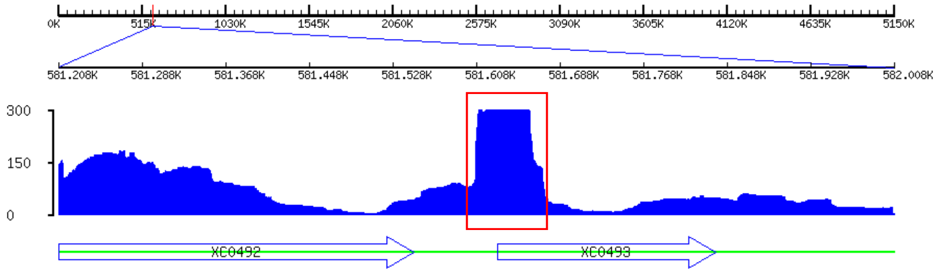

Transcript	Start	End	Length (bp)	RPKM	Inside gene	Overlap gene	Upstream gene	Downstream gene
TT078	587862	587940	79	42.3	Null	Null	XC0497	XC0498

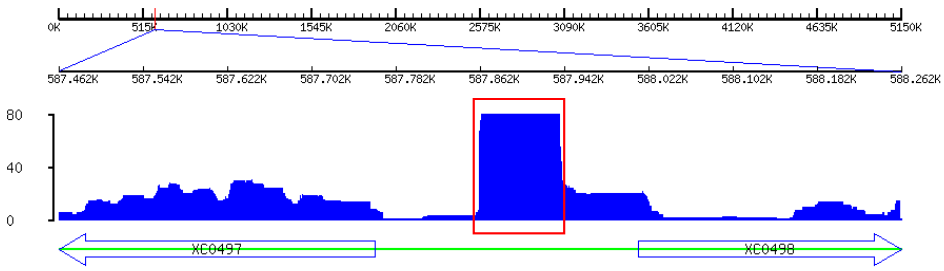

Transcript	Start	End	Length (bp)	RPKM	Inside gene	Overlap gene	Upstream gene	Downstream gene
TT079	621291	621369	79	71.1	Null	XC0525	XC0524	XC0526

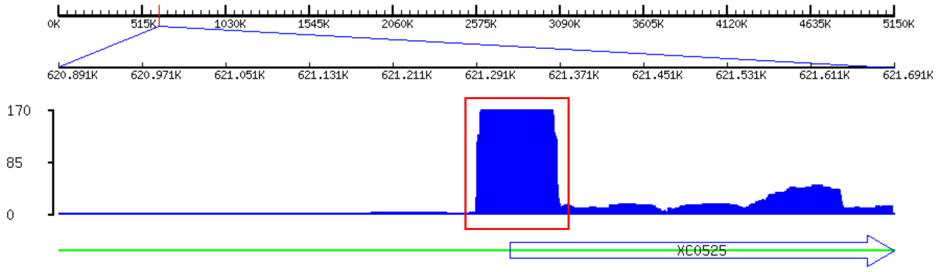

Transcript	Start	End	Length (bp)	RPKM	Inside gene	Overlap gene	Upstream gene	Downstream gene
TT080	624944	625033	90	42.6	XC0528	Null	XC0527	XC0529

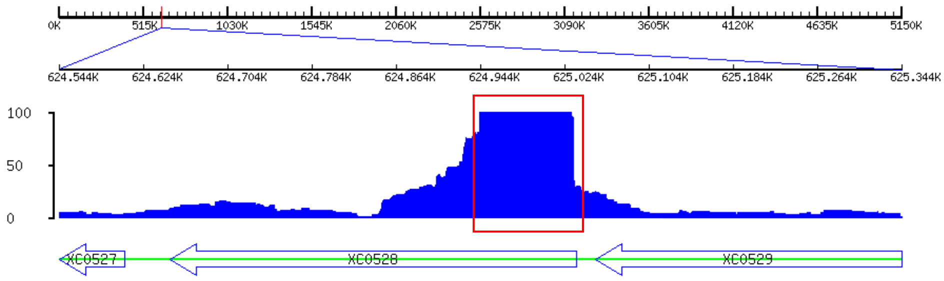

Transcript	Start	End	Length (bp)	RPKM	Inside gene	Overlap gene	Upstream gene	Downstream gene
TT081	626223	626282	60	146.7	Null	Null	XC0530	XC0531

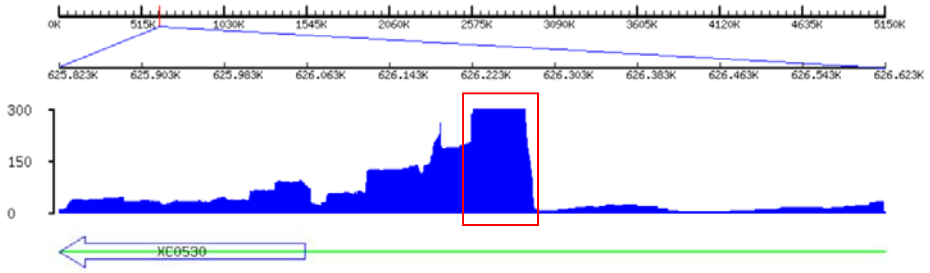

Transcript	Start	End	Length (bp)	RPKM	Inside gene	Overlap gene	Upstream gene	Downstream gene
TT082	632099	632168	70	79.7	Null	Null	XC0533	XC0534

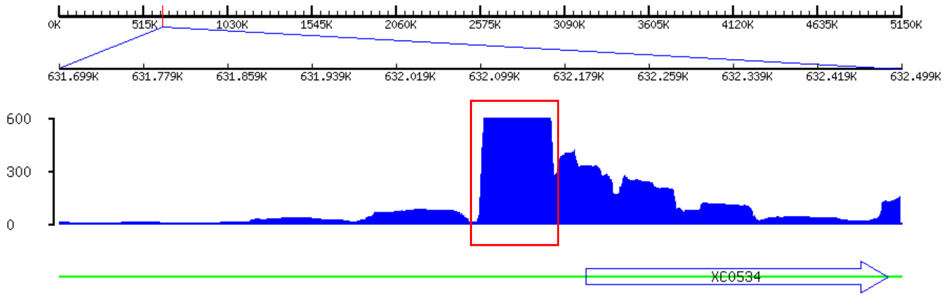

Transcript	Start	End	Length (bp)	RPKM	Inside gene	Overlap gene	Upstream gene	Downstream gene
TT083	633720	633783	64	64.9	XC0535	Null	XC0534	XC0536
TT084	633817	633876	60	46.2	XC0535	Null	XC0534	XC0536

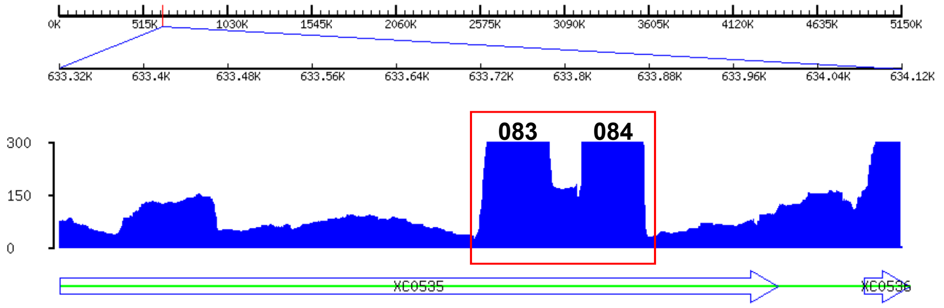

Transcript	Start	End	Length (bp)	RPKM	Inside gene	Overlap gene	Upstream gene	Downstream gene
TT085	639092	639163	72	56.2	XC0538	Null	XC0537	XC0539
TT086	639326	639384	59	134.2	XC0538	Null	XC0537	XC0539

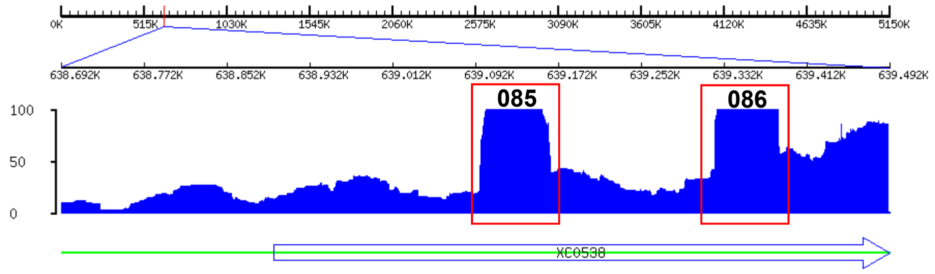

Transcript	Start	End	Length (bp)	RPKM	Inside gene	Overlap gene	Upstream gene	Downstream gene
TT087	677097	677186	90	421.1	Null	Null	XC0563	XC0564

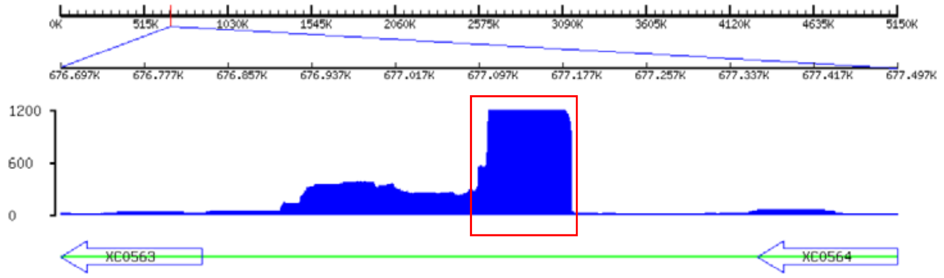

Transcript	Start	End	Length (bp)	RPKM	Inside gene	Overlap gene	Upstream gene	Downstream gene
TT088	697976	698036	61	201.1	XC0584	Null	XC0583	XC0585

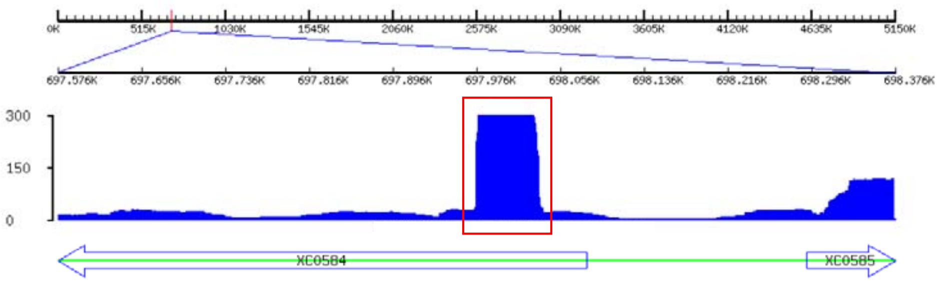

Transcript	Start	End	Length (bp)	RPKM	Inside gene	Overlap gene	Upstream gene	Downstream gene
TT089	704129	704202	74	261.9	XC0588	Null	XC0587	XC0589

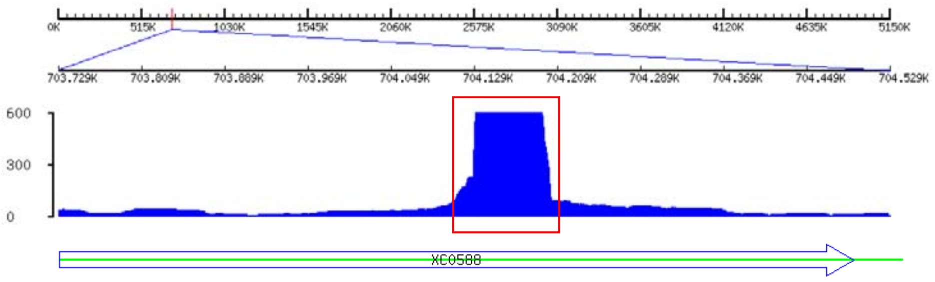

Transcript	Start	End	Length (bp)	RPKM	Inside gene	Overlap gene	Upstream gene	Downstream gene
TT090	704921	705070	150	295.1	Null	XC0589	XC0588	XC0590

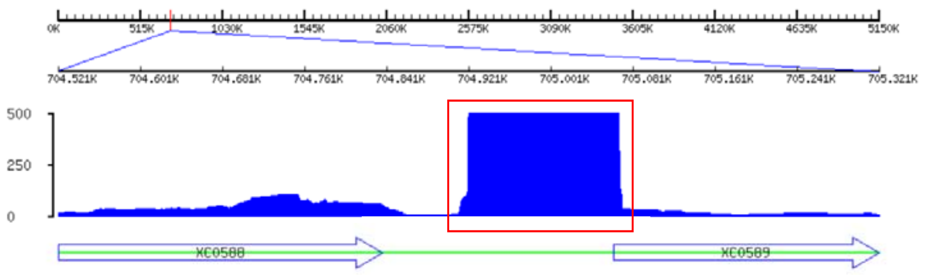

Transcript	Start	End	Length (bp)	RPKM	Inside gene	Overlap gene	Upstream gene	Downstream gene
TT091	752527	752580	54	33.4	Null	Null	XC0631	XC0632

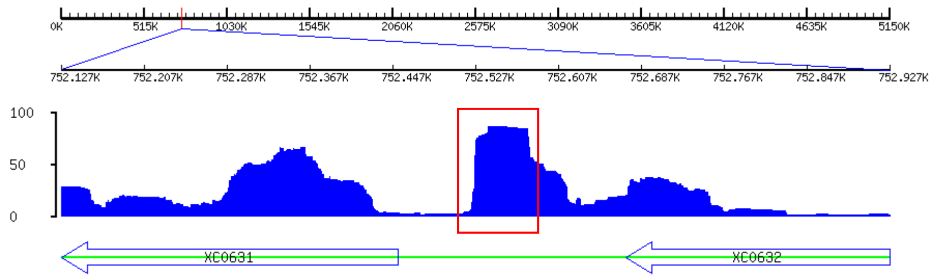

Transcript	Start	End	Length (bp)	RPKM	Inside gene	Overlap gene	Upstream gene	Downstream gene
TT092	753719	753782	64	115.3	XC0633	Null	XC0632	XC0634

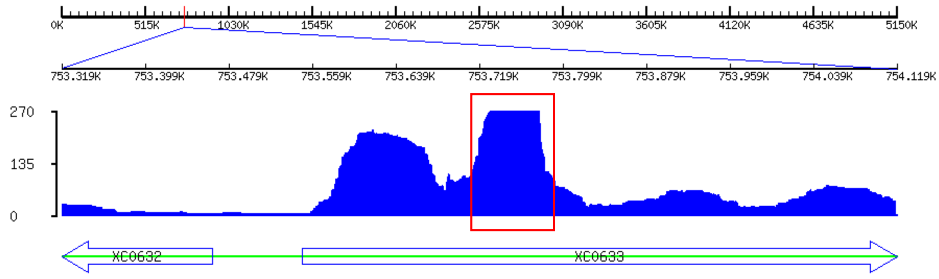

Transcript	Start	End	Length (bp)	RPKM	Inside gene	Overlap gene	Upstream gene	Downstream gene
TT093	766606	766671	66	299.9	Null	Null	XC0638	XC0639

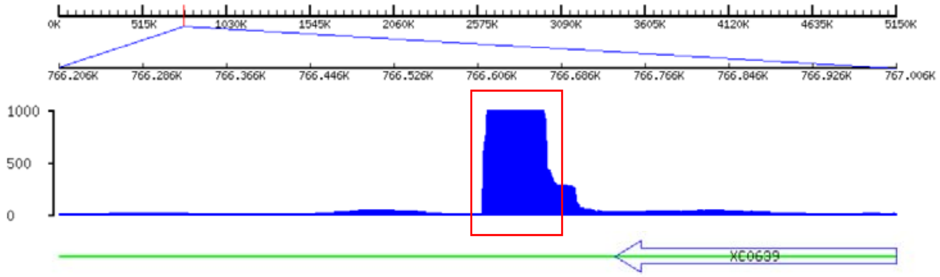

Transcript	Start	End	Length (bp)	RPKM	Inside gene	Overlap gene	Upstream gene	Downstream gene
TT094	767128	767235	108	57.6	XC0639	Null	XC0638	XC0640

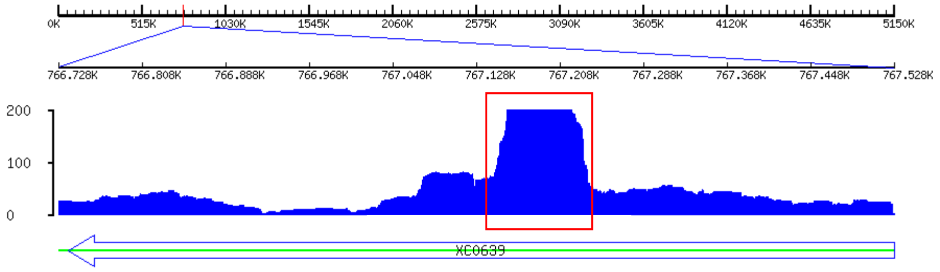

Transcript	Start	End	Length (bp)	RPKM	Inside gene	Overlap gene	Upstream gene	Downstream gene
TT095	768166	768225	60	297.1	Null	XC0639	XC0638	XC0640

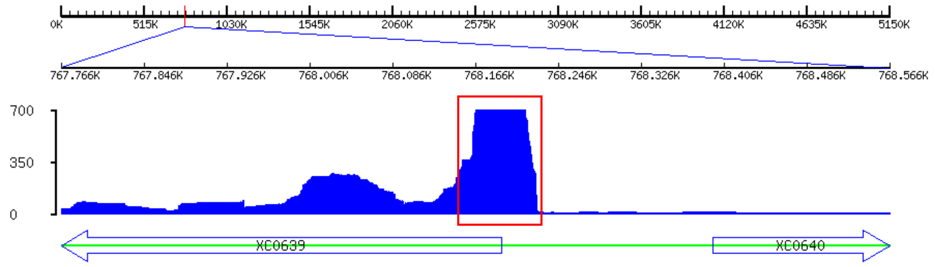

Transcript	Start	End	Length (bp)	RPKM	Inside gene	Overlap gene	Upstream gene	Downstream gene
TT096	781908	781975	68	69.4	Null	XC0647 /XC0648	XC0646	XC0649

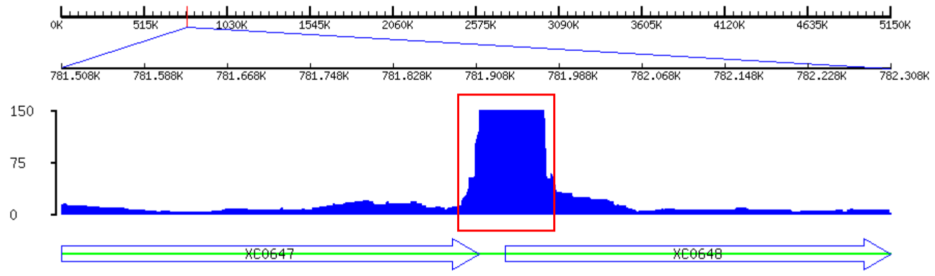

Transcript	Start	End	Length (bp)	RPKM	Inside gene	Overlap gene	Upstream gene	Downstream gene
TT097	791697	791786	90	38.4	Null	XC0658	XC0657	XC0659

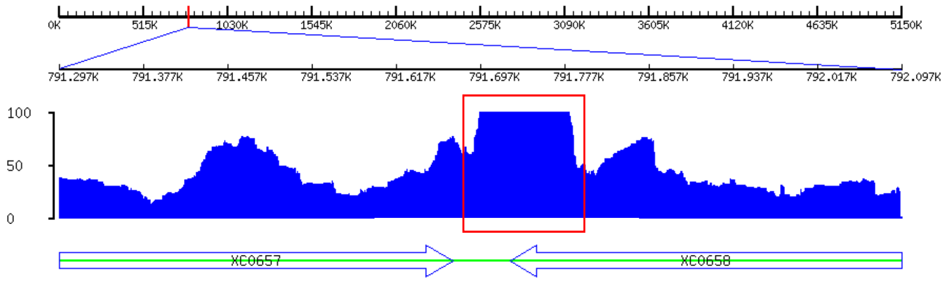

Transcript	Start	End	Length (bp)	RPKM	Inside gene	Overlap gene	Upstream gene	Downstream gene
TT098	793036	793100	65	70.2	Null	XC0659	XC0658	XC0660

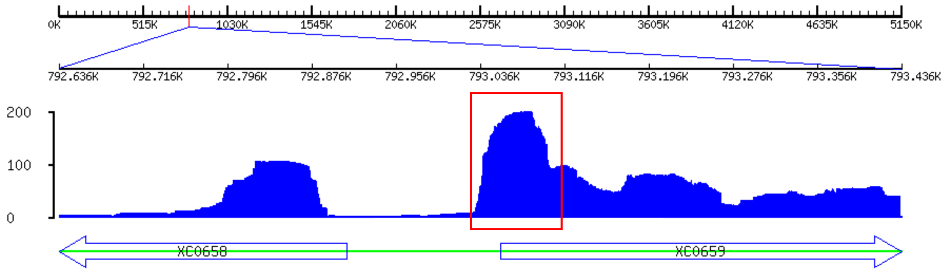

Transcript	Start	End	Length (bp)	RPKM	Inside gene	Overlap gene	Upstream gene	Downstream gene
TT099	793576	793633	58	80.9	XC0659	Null	XC0658	XC0660

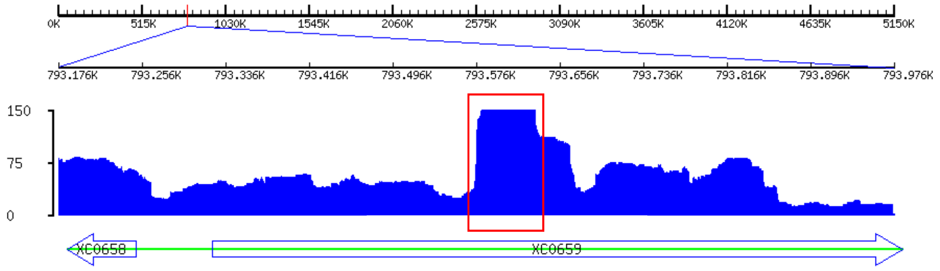

Transcript	Start	End	Length (bp)	RPKM	Inside gene	Overlap gene	Upstream gene	Downstream gene
TT100	830789	830861	73	253.9	XC0691	Null	XC0690	XC0692

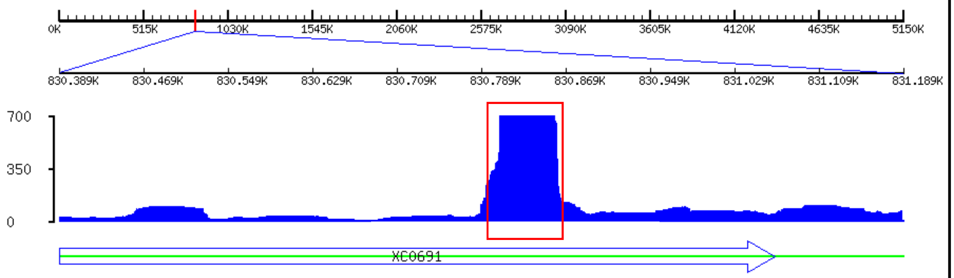

Transcript	Start	End	Length (bp)	RPKM	Inside gene	Overlap gene	Upstream gene	Downstream gene
TT101	848204	848259	56	274.8	Null	Null	XC0705	XC0706
TT102	848460	848519	60	54.7	XC0706	Null	XC0705	XC0707

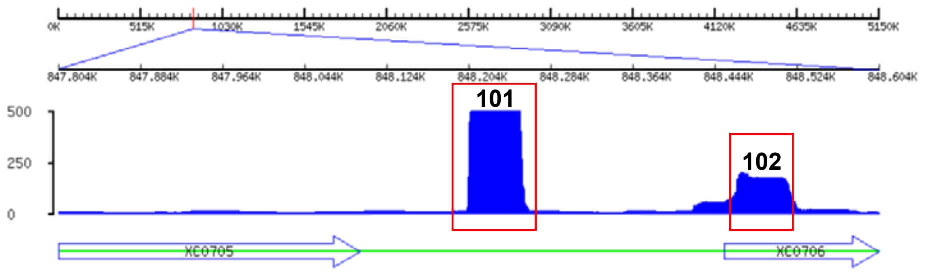

Transcript	Start	End	Length (bp)	RPKM	Inside gene	Overlap gene	Upstream gene	Downstream gene
TT103	849532	849588	57	155.5	XC0706	Null	XC0705	XC0707

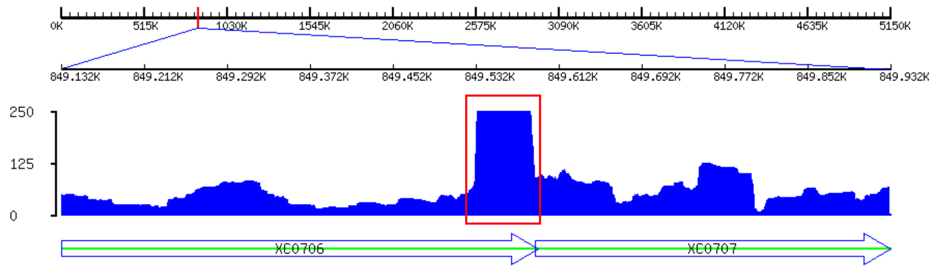

Transcript	Start	End	Length (bp)	RPKM	Inside gene	Overlap gene	Upstream gene	Downstream gene
TT104	853570	853651	81	26.5	Null	Null	XC0709	XC0711
TT105	853703	853786	84	114.6	Null	XC0710	XC0709	XC0711

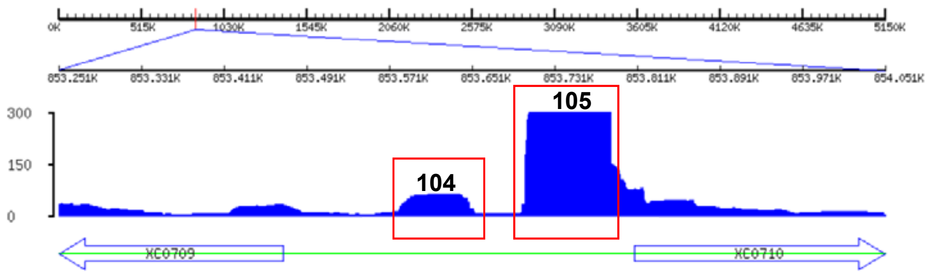

Transcript	Start	End	Length (bp)	RPKM	Inside gene	Overlap gene	Upstream gene	Downstream gene
TT106	854937	855002	65	158.4	XC0711	Null	XC0710	XC0712
TT107	855059	855156	97	72.7	XC0711	Null	XC0710	XC0712

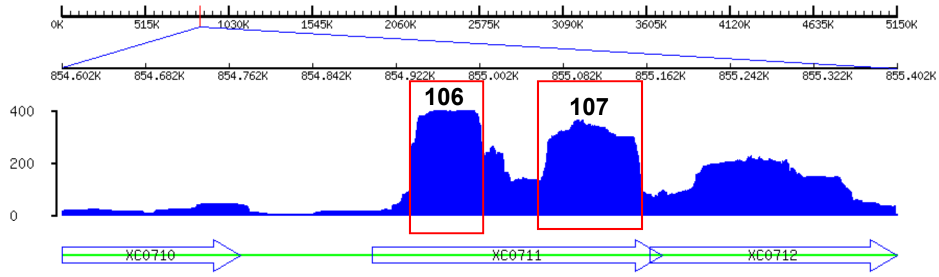

Transcript	Start	End	Length (bp)	RPKM	Inside gene	Overlap gene	Upstream gene	Downstream gene
TT108	857025	857080	56	810.9	Null	Null	XC0713	XC0714

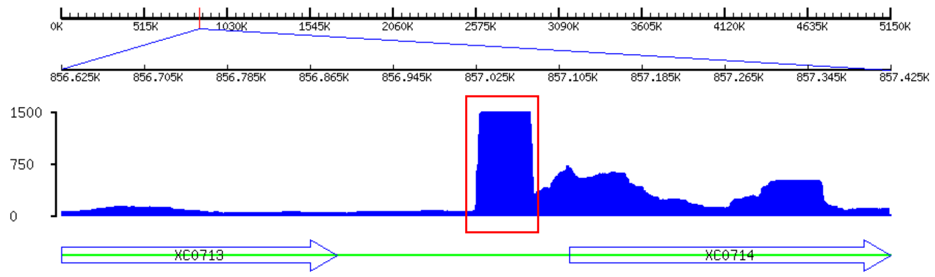

Transcript	Start	End	Length (bp)	RPKM	Inside gene	Overlap gene	Upstream gene	Downstream gene
TT109	859744	859833	90	38.1	XC0715	Null	XC0714	XC0716

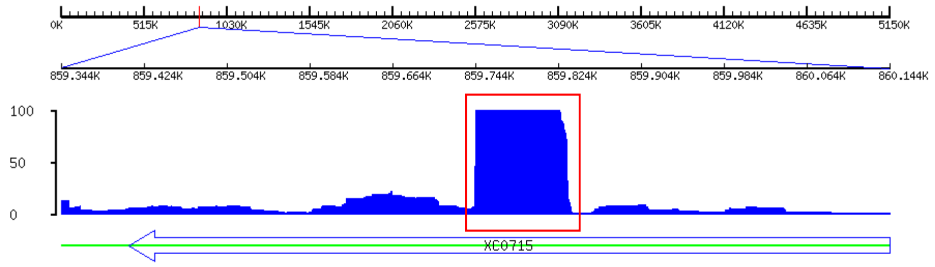

Transcript	Start	End	Length (bp)	RPKM	Inside gene	Overlap gene	Upstream gene	Downstream gene
TT110	861445	861551	107	32.9	XC0717	Null	XC0716	XC0718

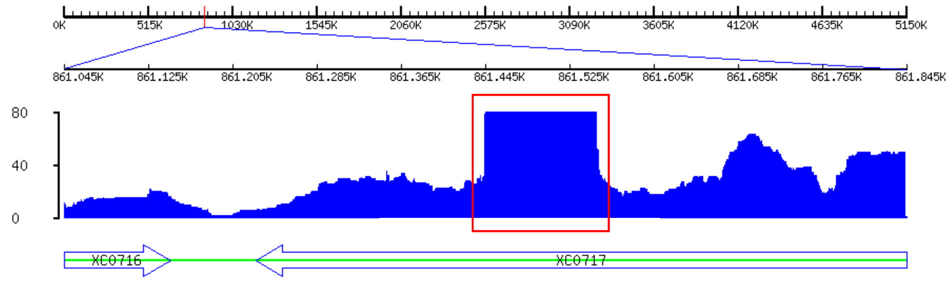

Transcript	Start	End	Length (bp)	RPKM	Inside gene	Overlap gene	Upstream gene	Downstream gene
TT111	862234	862334	101	576.2	Null	XC0718	XC0717	XC0719

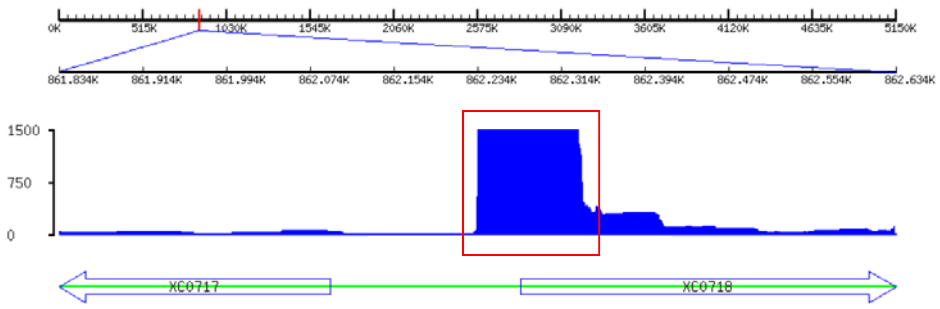

Transcript	Start	End	Length (bp)	RPKM	Inside gene	Overlap gene	Upstream gene	Downstream gene
TT112	867931	868360	429	1168.1	XC0723	Null	XC0722	XC0724

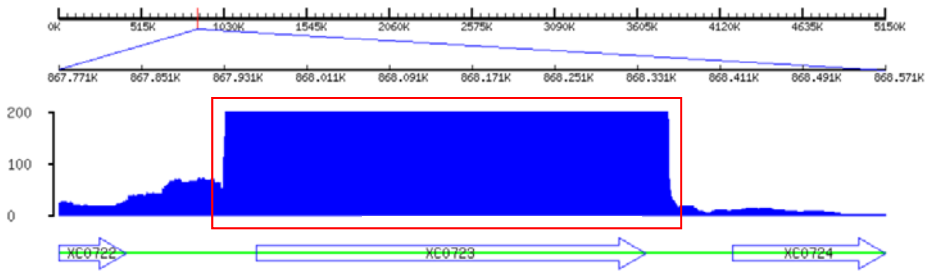

Transcript	Start	End	Length (bp)	RPKM	Inside gene	Overlap gene	Upstream gene	Downstream gene
TT113	867931	868054	124	1111.6	Null	XC0723	XC0722	XC0724
TT114	868058	868137	80	807.7	XC0723	Null	XC0722	XC0724
TT115	868138	868230	93	1962.1	XC0723	Null	XC0722	XC0724
TT116	868271	868360	90	1376.4	Null	XC0723	XC0722	XC0724

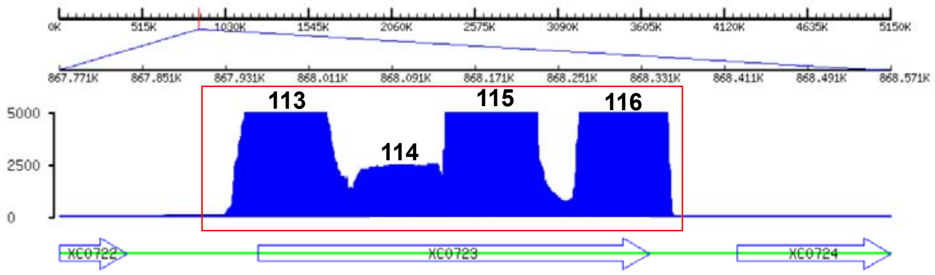

Transcript	Start	End	Length (bp)	RPKM	Inside gene	Overlap gene	Upstream gene	Downstream gene
TT117	872102	872531	429	494.1	XC0727	Null	XC0726	XC0728

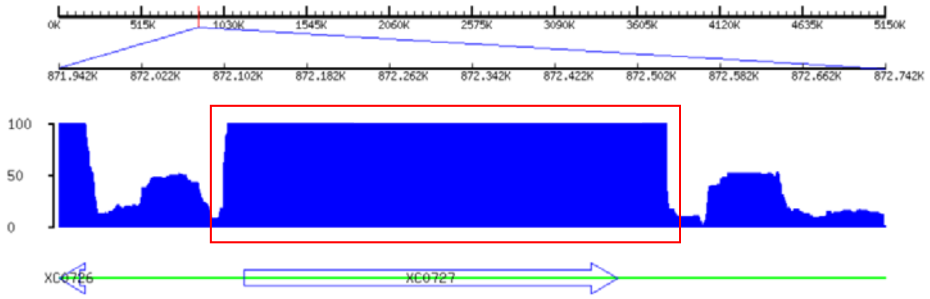

Transcript	Start	End	Length (bp)	RPKM	Inside gene	Overlap gene	Upstream gene	Downstream gene
TT118	872436	872531	116	771.7	Null	XC0727	XC0726	XC0728

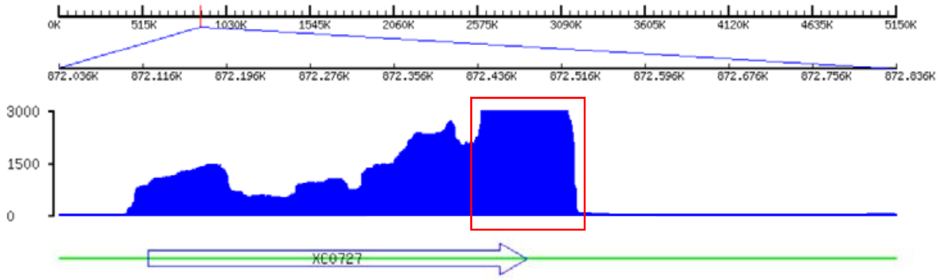

Transcript	Start	End	Length (bp)	RPKM	Inside gene	Overlap gene	Upstream gene	Downstream gene
TT119	872797	872880	84	50.8	Null	Null	XC0727	XC0728
TT120	872917	873019	103	304.7	Null	XC0728	XC0727	XC0729

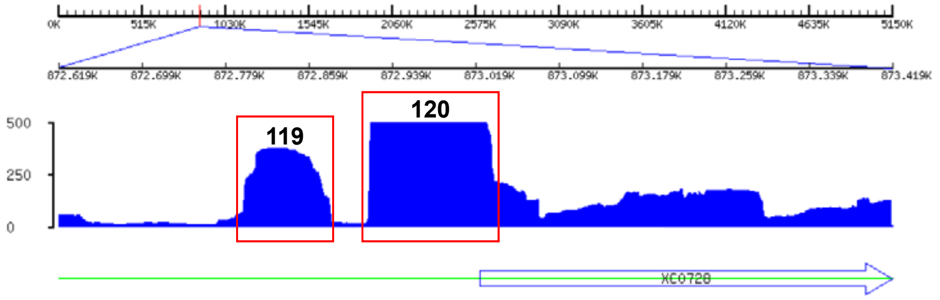

Transcript	Start	End	Length (bp)	RPKM	Inside gene	Overlap gene	Upstream gene	Downstream gene
TT121	923871	923936	66	195.5	Null	XC0768	XC0767	XC0769

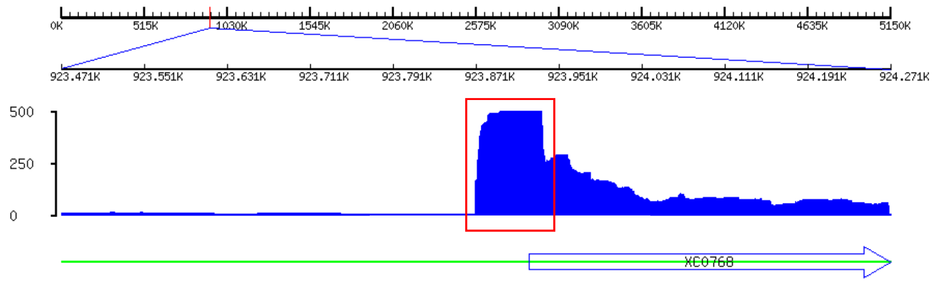

Transcript	Start	End	Length (bp)	RPKM	Inside gene	Overlap gene	Upstream gene	Downstream gene
TT122	951277	951358	82	68.4	Null	XC0796	XC0795	XC0797

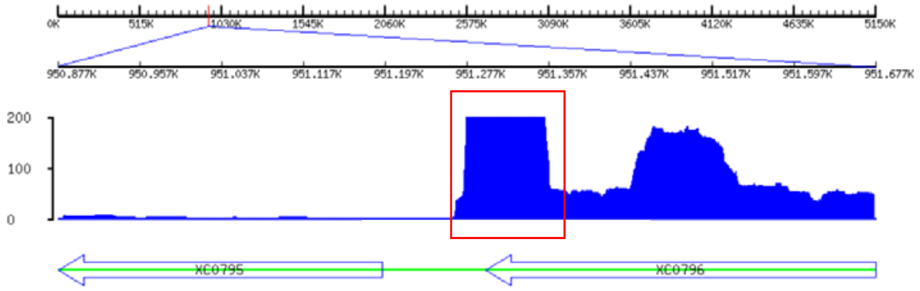

Transcript	Start	End	Length (bp)	RPKM	Inside gene	Overlap gene	Upstream gene	Downstream gene
TT123	951867	951935	69	194.5	XC0796	Null	XC0795	XC0797

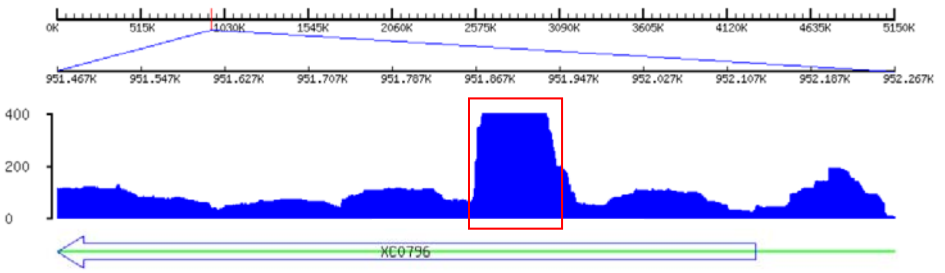

Transcript	Start	End	Length (bp)	RPKM	Inside gene	Overlap gene	Upstream gene	Downstream gene
TT124	971506	971565	60	58.1	XC0811	Null	XC0810	XC0812

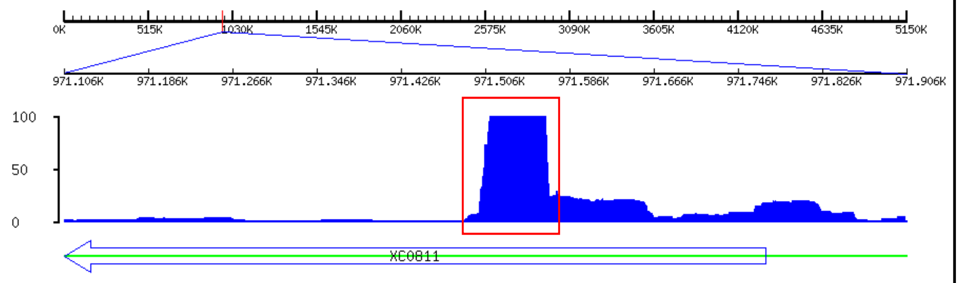

Transcript	Start	End	Length (bp)	RPKM	Inside gene	Overlap gene	Upstream gene	Downstream gene
TT125	997549	997605	56	42.3	XC0830	Null	XC0829	XC0831
TT126	997786	997835	50	102.4	XC0830	Null	XC0829	XC0831
TT127	998027	998098	72	88.6	Null	XC0831	XC0830	XC0832

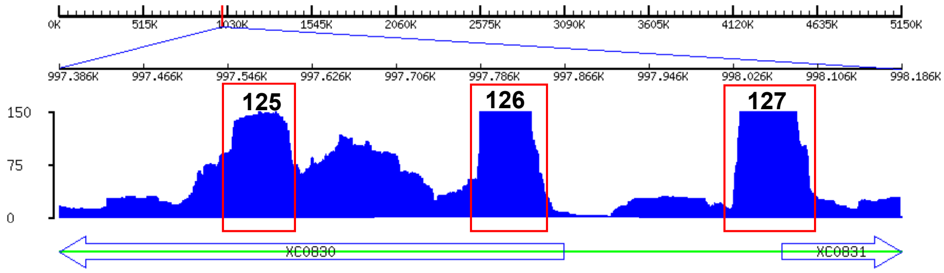

Transcript	Start	End	Length (bp)	RPKM	Inside gene	Overlap gene	Upstream gene	Downstream gene
TT128	1009676	1009743	68	162.5	XC0838	Null	XC0837	XC0839

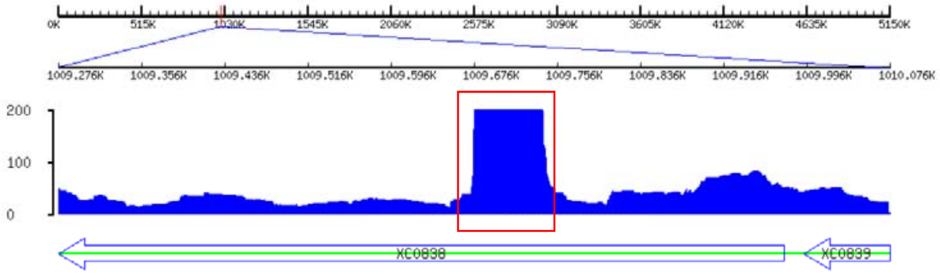

Transcript	Start	End	Length (bp)	RPKM	Inside gene	Overlap gene	Upstream gene	Downstream gene
TT129	1013277	1013372	96	2433.3	Null	Null	XC0841	XC0842

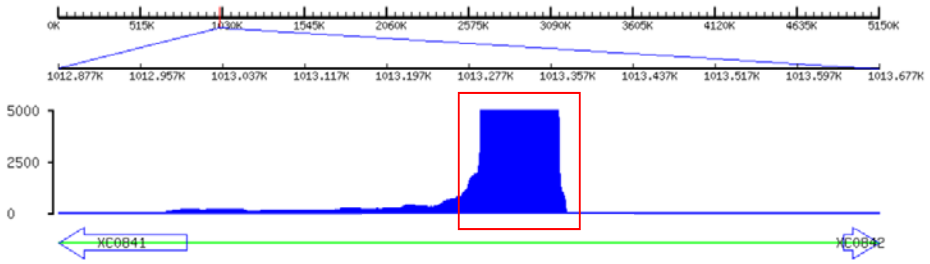

Transcript	Start	End	Length (bp)	RPKM	Inside gene	Overlap gene	Upstream gene	Downstream gene
TT130	1014431	1014497	67	53.9	XC0842	Null	XC0841	XC0843

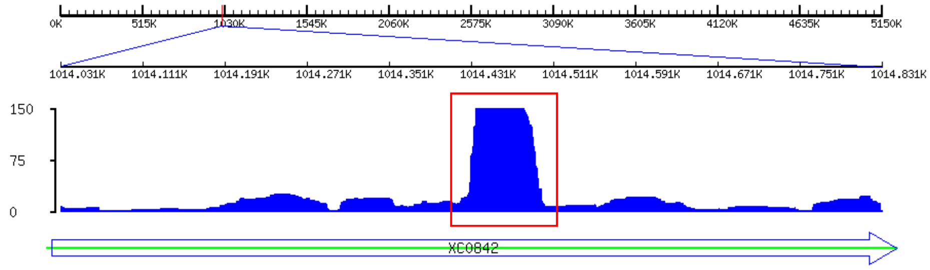

Transcript	Start	End	Length (bp)	RPKM	Inside gene	Overlap gene	Upstream gene	Downstream gene
TT131	1019341	1019428	88	1519.9	Null	Null	XC0844	XC0845

Now ranger of the following image is from 1018941 bp to 1019740 bp , Chromsome zoom value is 1 : 1 , Depth zoom value is 10 : 1

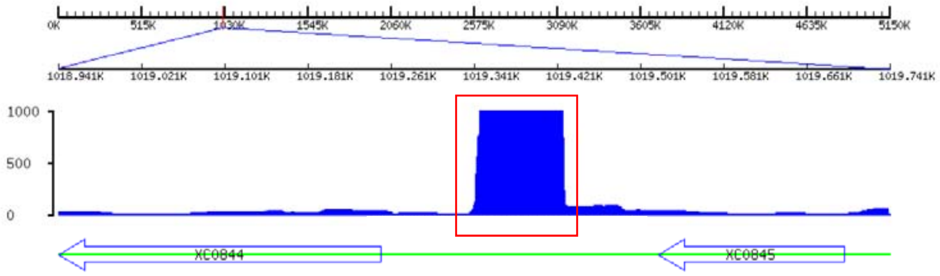

Transcript	Start	End	Length (bp)	RPKM	Inside gene	Overlap gene	Upstream gene	Downstream gene
TT132	1019825	1020040	215	178.2	Null	XC0846	XC0845	XC0847

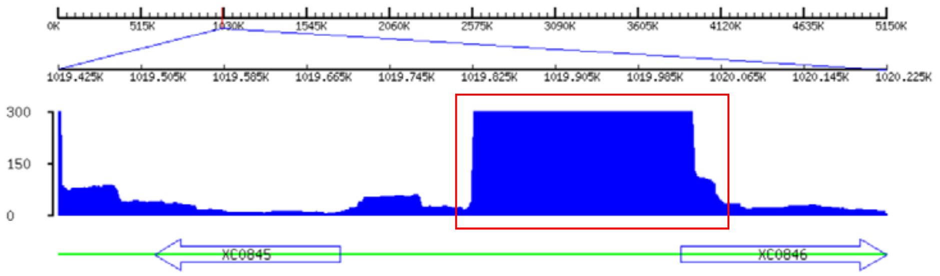

Transcript	Start	End	Length (bp)	RPKM	Inside gene	Overlap gene	Upstream gene	Downstream gene
TT133	1022098	1022147	50	360.9	Null	Null	XC0846	XC0847

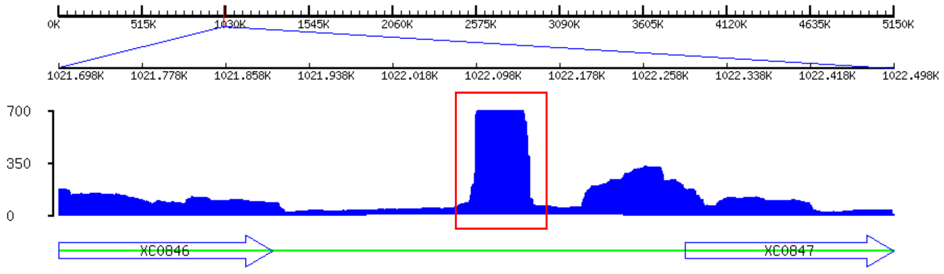

Transcript	Start	End	Length (bp)	RPKM	Inside gene	Overlap gene	Upstream gene	Downstream gene
TT134	1025928	1026043	116	207.8	XC0849	Null	XC0848	XC0850

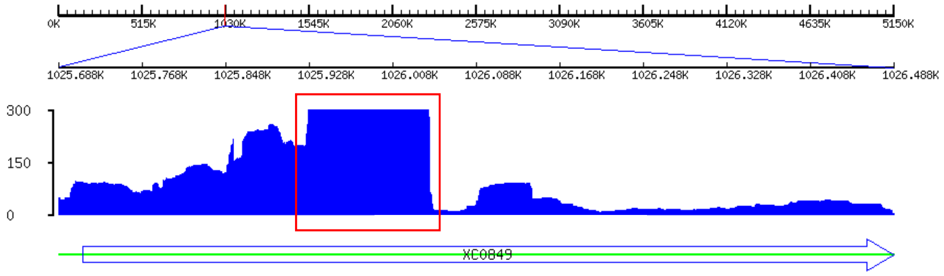

Transcript	Start	End	Length (bp)	RPKM	Inside gene	Overlap gene	Upstream gene	Downstream gene
TT135	1034180	1034253	73	43.7	Null	Null	XC0853	XC0854
TT136	1034363	1034451	89	100.5	Null	XC0854	XC0853	XC0855

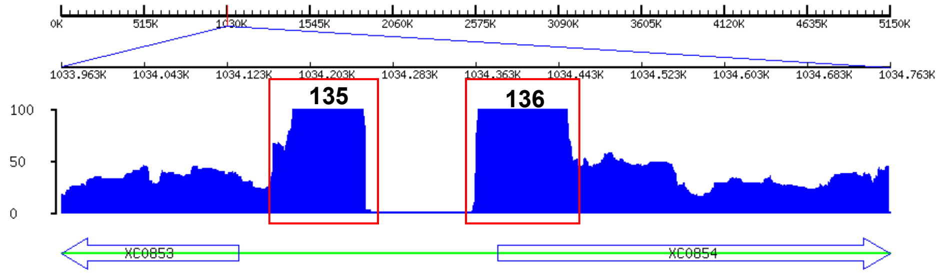

Transcript	Start	End	Length (bp)	RPKM	Inside gene	Overlap gene	Upstream gene	Downstream gene
TT137	1035293	1035345	53	137.23	XC0855	Null	XC0854	XC0856

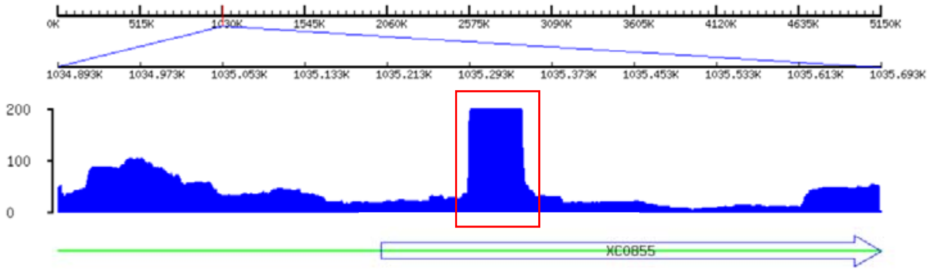

Transcript	Start	End	Length (bp)	RPKM	Inside gene	Overlap gene	Upstream gene	Downstream gene
TT138	1036765	1036832	68	13.1	XC0857	Null	XC0856	XC0858
TT139	1037060	1037130	71	79.9	XC0857	Null	XC0856	XC0858

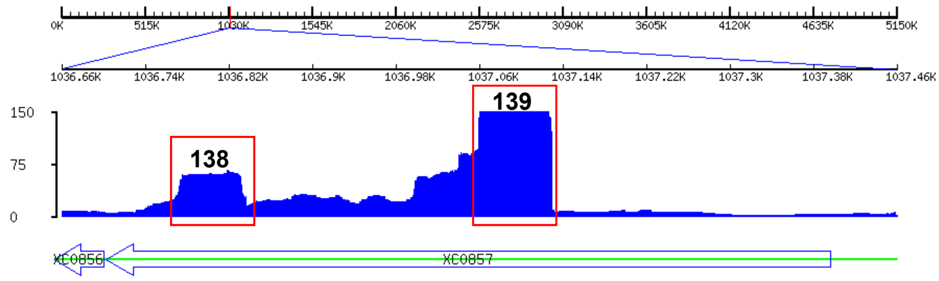

Transcript	Start	End	Length (bp)	RPKM	Inside gene	Overlap gene	Upstream gene	Downstream gene
TT140	1051759	1051813	55	52.7	Null	Null	XC0872	XC0873
TT141	1051878	1051967	90	140.9	XC0873	Null	XC0872	XC0874

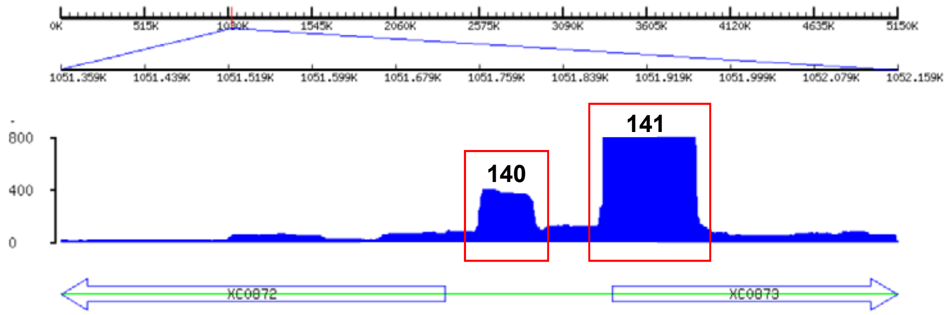

Transcript	Start	End	Length (bp)	RPKM	Inside gene	Overlap gene	Upstream gene	Downstream gene
TT142	1052567	1052660	94	149.1	Null	Null	XC0873	XC0874

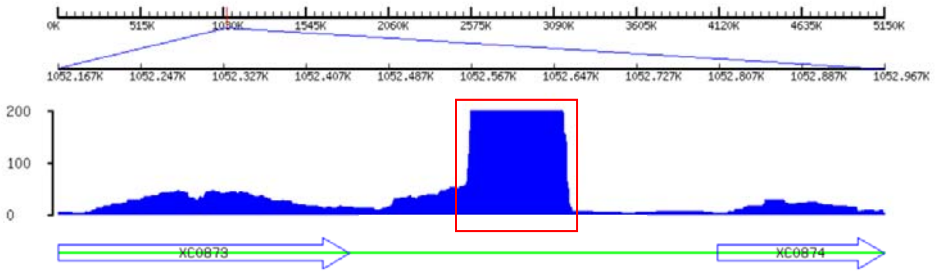

Transcript	Start	End	Length (bp)	RPKM	Inside gene	Overlap gene	Upstream gene	Downstream gene
TT143	1074880	1075087	207	25.1	Null	XC0891	XC0890	XC0892

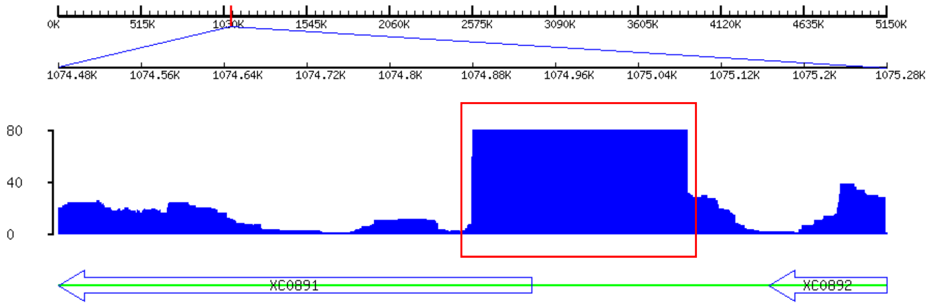

Transcript	Start	End	Length (bp)	RPKM	Inside gene	Overlap gene	Upstream gene	Downstream gene
TT144	1080412	1080475	63	195.9	Null	Null	XC0894	XC0895

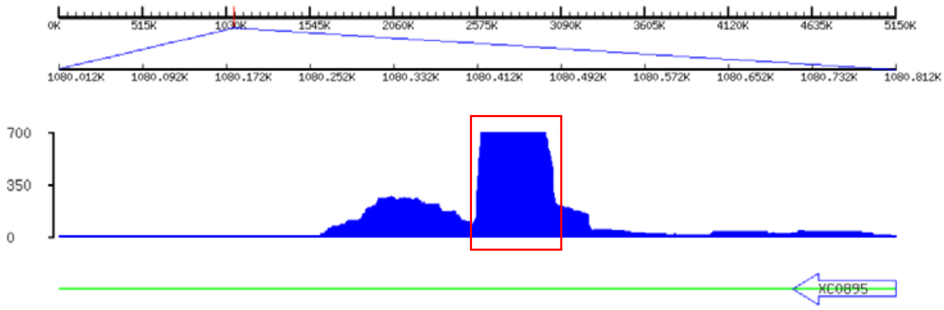

Transcript	Start	End	Length (bp)	RPKM	Inside gene	Overlap gene	Upstream gene	Downstream gene
TT145	1085288	1085468	181	49972.9	Null	Null	XC0901	XC0902

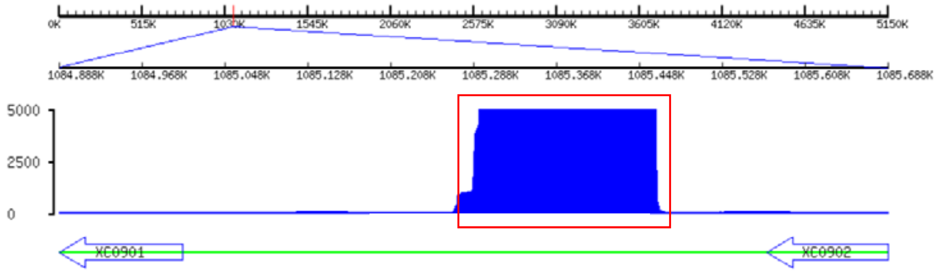

Transcript	Start	End	Length (bp)	RPKM	Inside gene	Overlap gene	Upstream gene	Downstream gene
TT146	1088492	1088680	188	167.3	Null	Null	XC0905	XC0906

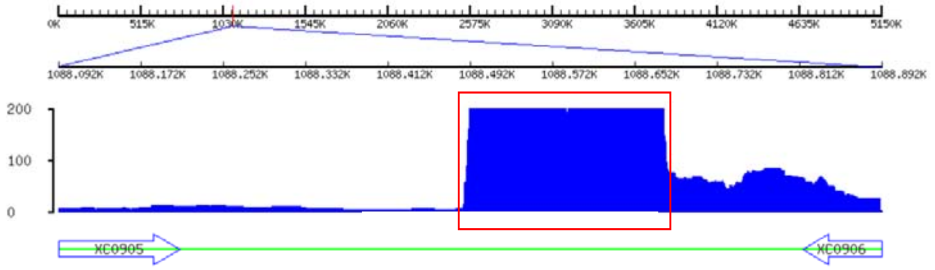

Transcript	Start	End	Length (bp)	RPKM	Inside gene	Overlap gene	Upstream gene	Downstream gene
TT147	1097403	1097479	77	438.3	XC0916	Null	XC0915	XC0917

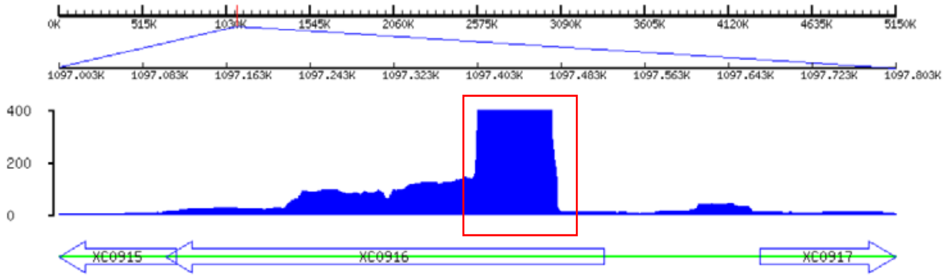

Transcript	Start	End	Length (bp)	RPKM	Inside gene	Overlap gene	Upstream gene	Downstream gene
TT148	1108108	1108160	53	93.4	Null	XC0924	XC0923	XC0925

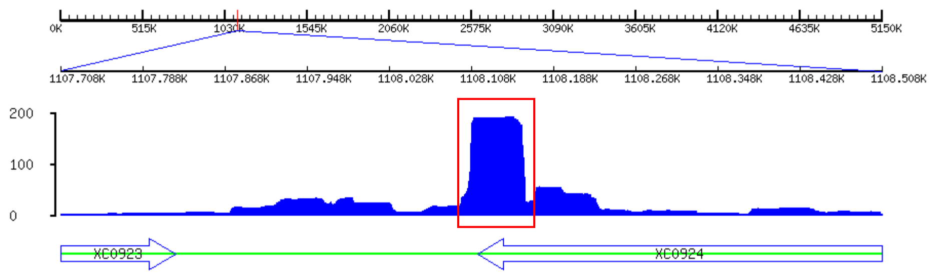

Transcript	Start	End	Length (bp)	RPKM	Inside gene	Overlap gene	Upstream gene	Downstream gene
TT149	1110302	1110379	78	11.9	Null	XC0925	XC0924	XC0926

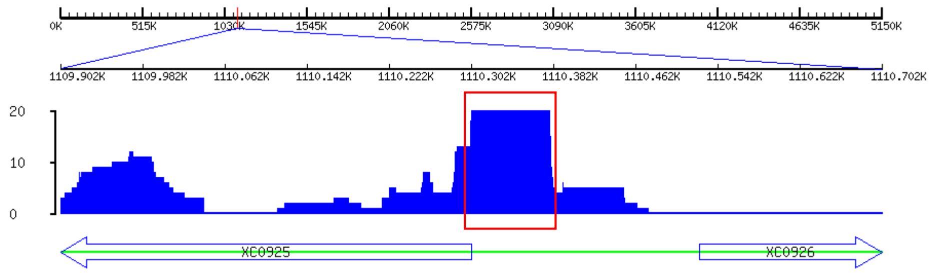

Transcript	Start	End	Length (bp)	RPKM	Inside gene	Overlap gene	Upstream gene	Downstream gene
TT150	1135264	1135538	274	130.6	XC0945	Null	XC0944	XC0946

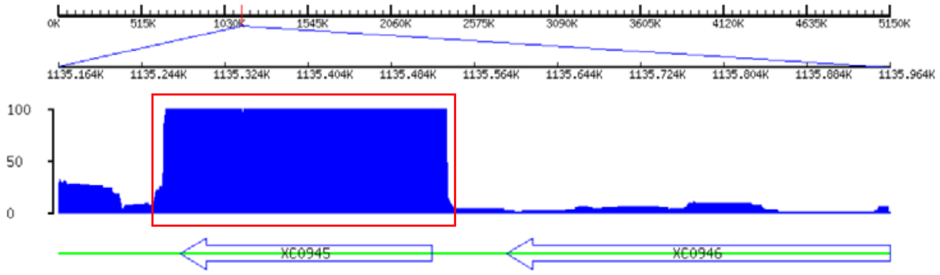

Transcript	Start	End	Length (bp)	RPKM	Inside gene	Overlap gene	Upstream gene	Downstream gene
TT151	1162926	1163013	88	39.6	Null	XC0969	XC0968	XC0970

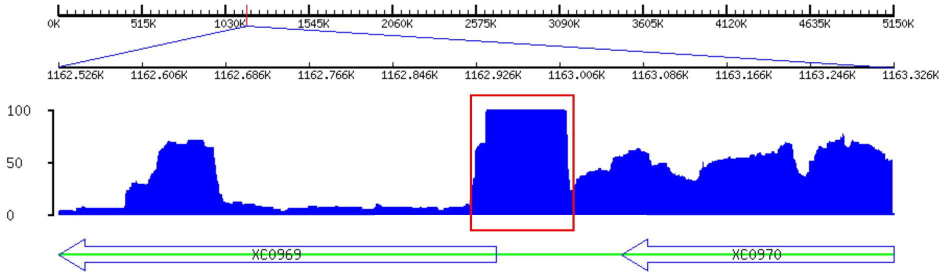

Transcript	Start	End	Length (bp)	RPKM	Inside gene	Overlap gene	Upstream gene	Downstream gene
TT152	1170965	1171020	56	20.2	Null	Null	XC0975	XC0976

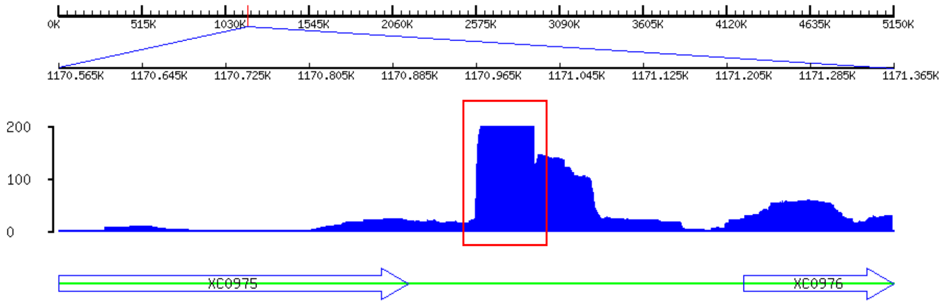

Transcript	Start	End	Length (bp)	RPKM	Inside gene	Overlap gene	Upstream gene	Downstream gene
TT153	1176661	1176720	59	962.6	XC0980	Null	XC0979	XC0981

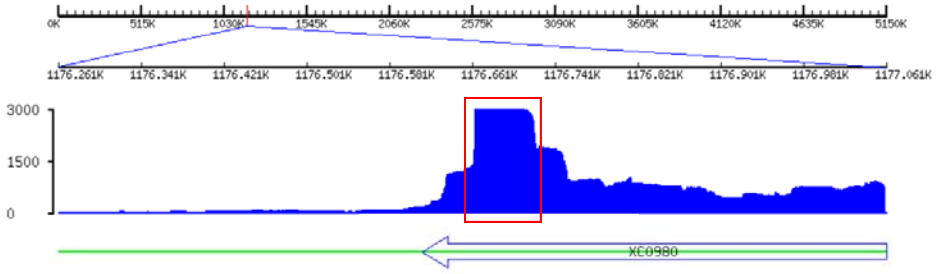

Transcript	Start	End	Length (bp)	RPKM	Inside gene	Overlap gene	Upstream gene	Downstream gene
TT154	1189861	1189937	77	147.4	XC0991	Null	XC0990	XC0992

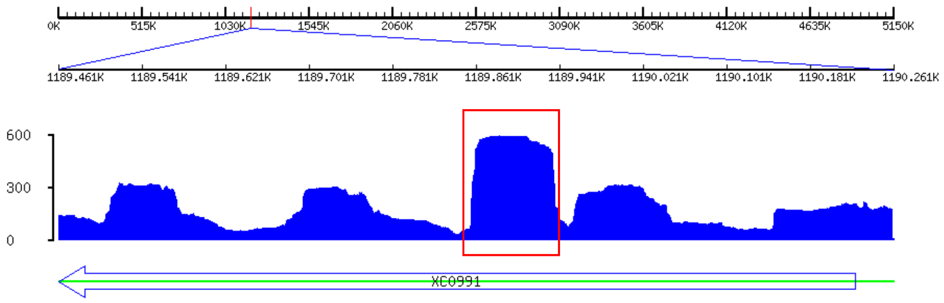

Transcript	Start	End	Length (bp)	RPKM	Inside gene	Overlap gene	Upstream gene	Downstream gene
TT155	1190631	1190685	55	2095.7	Null	Null	XC0990	XC0992

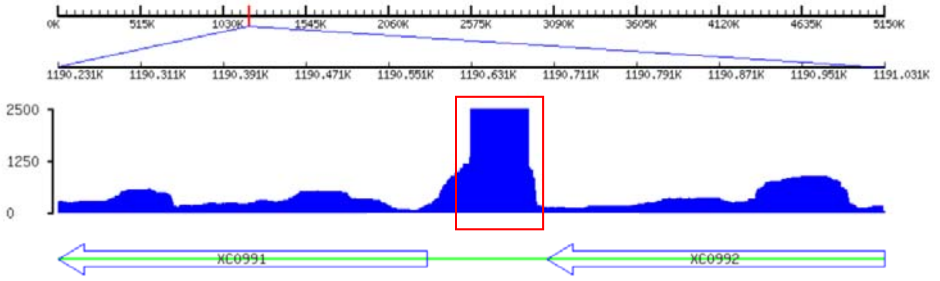

Transcript	Start	End	Length (bp)	RPKM	Inside gene	Overlap gene	Upstream gene	Downstream gene
TT156	1222132	1222192	61	195.9	Null	XC1009	XC1008	XC1010

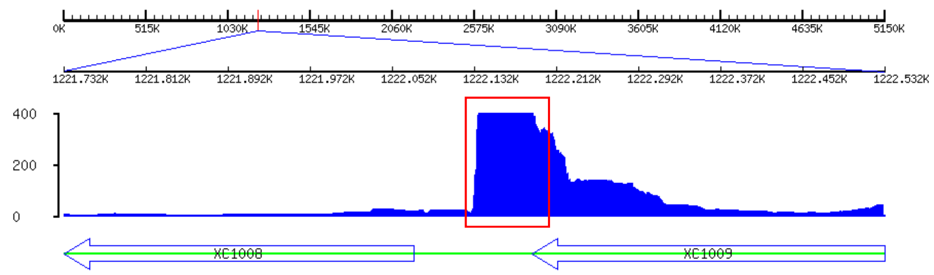

Transcript	Start	End	Length (bp)	RPKM	Inside gene	Overlap gene	Upstream gene	Downstream gene
TT157	1222577	1222649	73	155.9	Null	XC1009	XC1008	XC1010

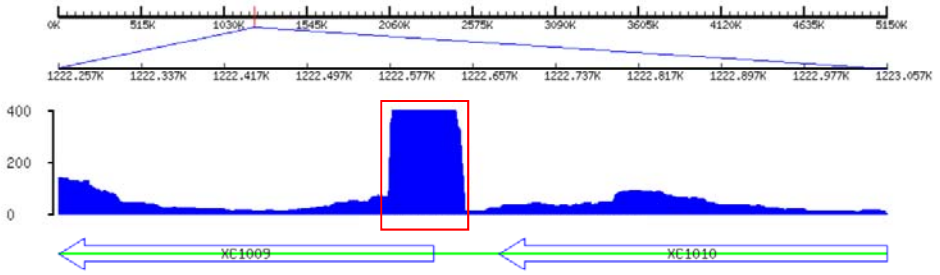

Transcript	Start	End	Length (bp)	RPKM	Inside gene	Overlap gene	Upstream gene	Downstream gene
TT158	1230874	1230938	65	493.9	Null	XC4336	XC1017	XC1018
TT159	1231053	1231129	77	491.6	Null	Null	XC4336	XC1018

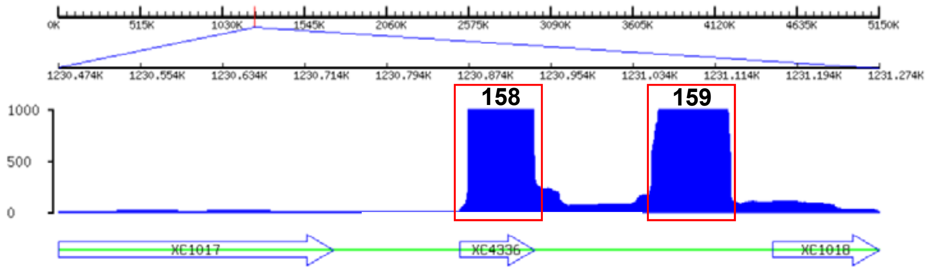

Transcript	Start	End	Length (bp)	RPKM	Inside gene	Overlap gene	Upstream gene	Downstream gene
TT160	1233305	1233394	90	99.5	Null	Null	XC1018	XC1019

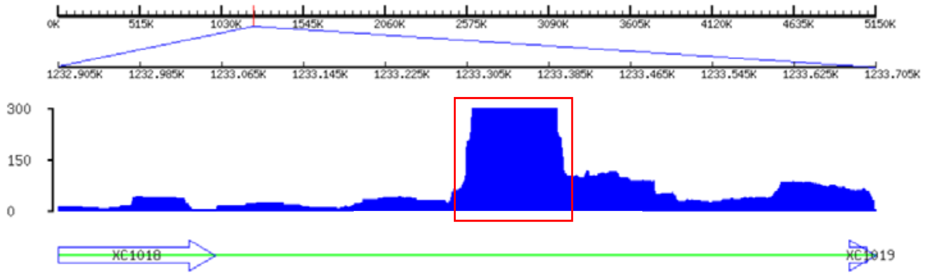

Transcript	Start	End	Length (bp)	RPKM	Inside gene	Overlap gene	Upstream gene	Downstream gene
TT161	1241514	1241570	57	308.1	Null	Null	XC1023	XC1024

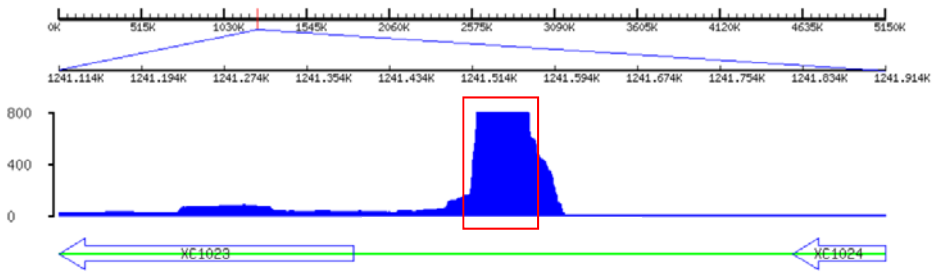

Transcript	Start	End	Length (bp)	RPKM	Inside gene	Overlap gene	Upstream gene	Downstream gene
TT162	1259596	1259664	69	85.2	Null	XC1044	XC1043	XC1045

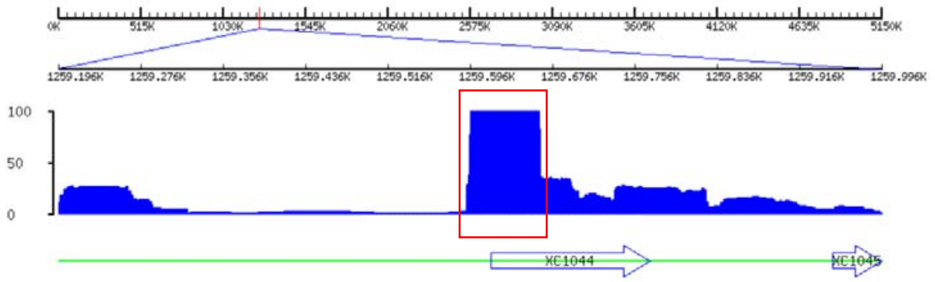

Transcript	Start	End	Length (bp)	RPKM	Inside gene	Overlap gene	Upstream gene	Downstream gene
TT163	1260414	1260464	50	98.6	Null	XC1046	XC1045	XC1047
TT164	1260784	1260859	76	128.1	Null	XC1046	XC1045	XC1047

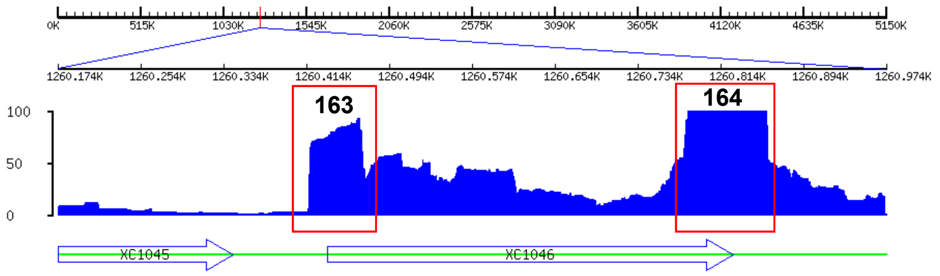

Transcript	Start	End	Length (bp)	RPKM	Inside gene	Overlap gene	Upstream gene	Downstream gene
TT165	1269031	1269101	71	1206.7	Null	Null	XC1053	XC1054

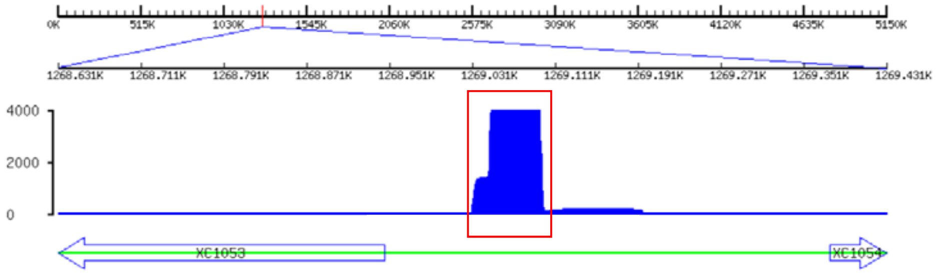

Transcript	Start	End	Length (bp)	RPKM	Inside gene	Overlap gene	Upstream gene	Downstream gene
TT166	1295653	1295721	69	70.4	Null	Null	XC1075	XC1076

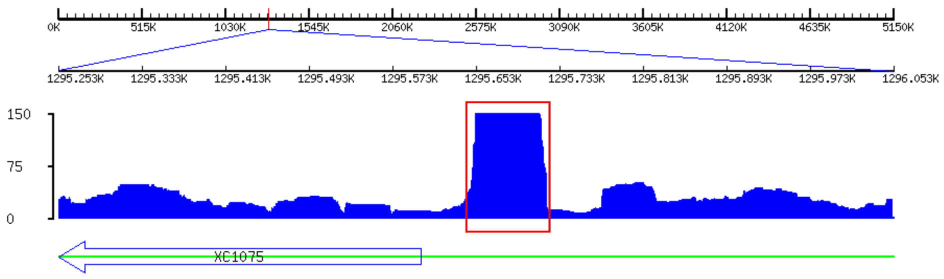

Transcript	Start	End	Length (bp)	RPKM	Inside gene	Overlap gene	Upstream gene	Downstream gene
TT167	1296858	1296927	70	100.9	XC1076	Null	XC1075	XC1077

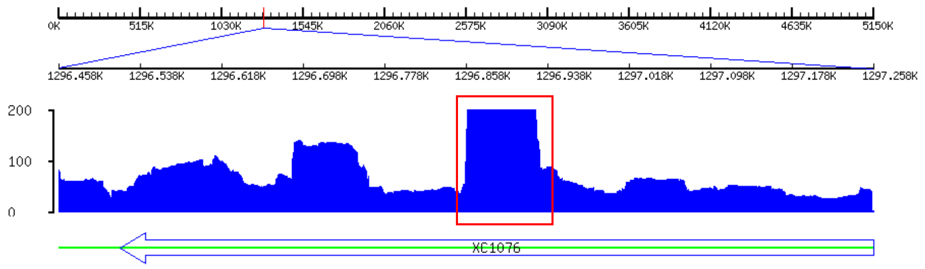

Transcript	Start	End	Length (bp)	RPKM	Inside gene	Overlap gene	Upstream gene	Downstream gene
TT168	1316918	1317118	201	133.9	Null	Null	XC1090	XC1091

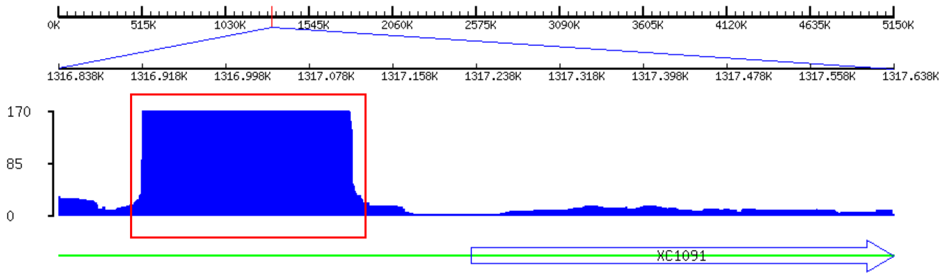

Transcript	Start	End	Length (bp)	RPKM	Inside gene	Overlap gene	Upstream gene	Downstream gene
TT169	1341918	1341977	60	123.5	Null	Null	XC1110	XC1111

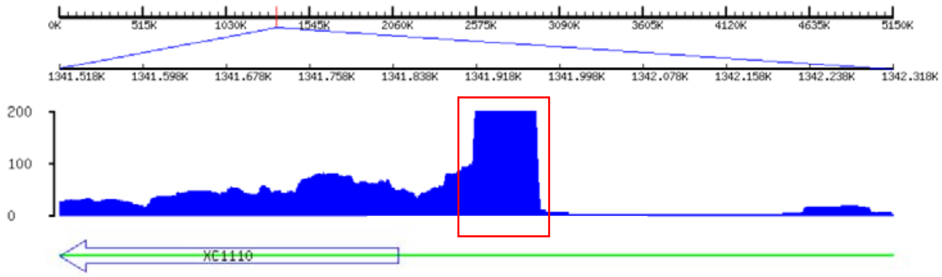

Transcript	Start	End	Length (bp)	RPKM	Inside gene	Overlap gene	Upstream gene	Downstream gene
TT170	1348210	1348271	62	61.9	XC1113	Null	XC1112	XC1114

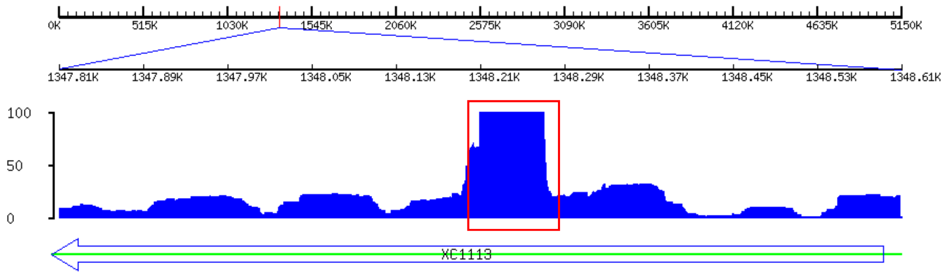

Transcript	Start	End	Length (bp)	RPKM	Inside gene	Overlap gene	Upstream gene	Downstream gene
TT171	1379140	1379198	59	82.9	XC1136	Null	XC1135	XC1137

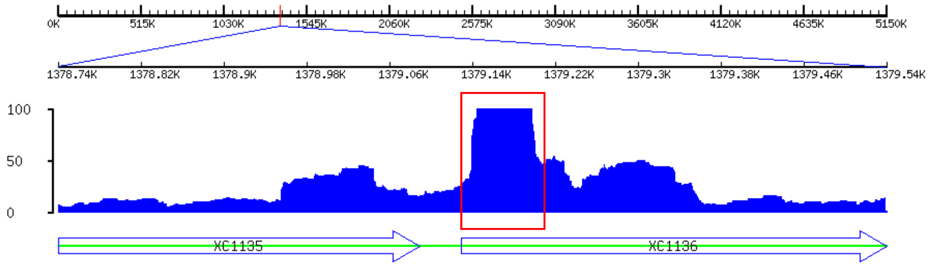

Transcript	Start	End	Length (bp)	RPKM	Inside gene	Overlap gene	Upstream gene	Downstream gene
TT172	1381928	1382023	96	228.4	Null	XC1139 /XC1140	XC1138	XC1141

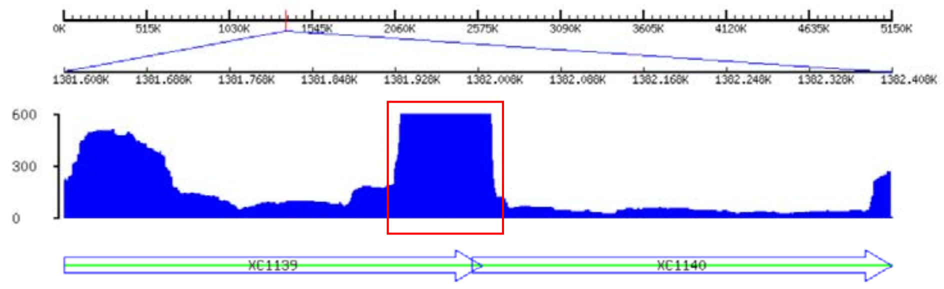

Transcript	Start	End	Length (bp)	RPKM	Inside gene	Overlap gene	Upstream gene	Downstream gene
TT173	1383289	1383342	54	96.9	XC1140	Null	XC1139	XC1141

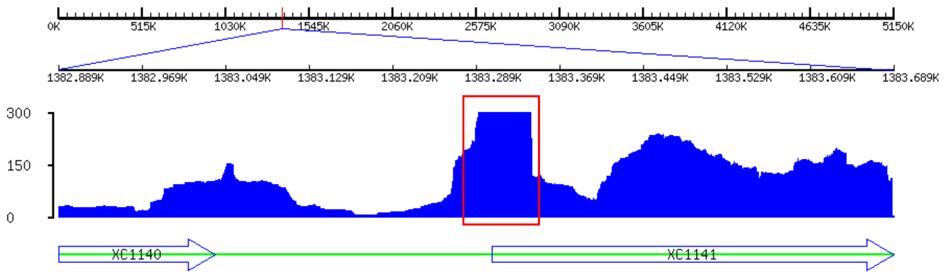

Transcript	Start	End	Length (bp)	RPKM	Inside gene	Overlap gene	Upstream gene	Downstream gene
TT174	1387682	1387742	60	308.7	XC4337	Null	XC1145	XC1147

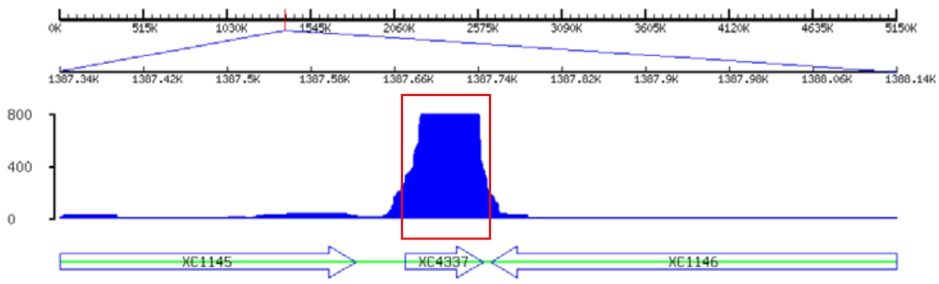

Transcript	Start	End	Length (bp)	RPKM	Inside gene	Overlap gene	Upstream gene	Downstream gene
TT175	1397137	1397310	173	17.2	Null	XC1156	XC1155	XC1157

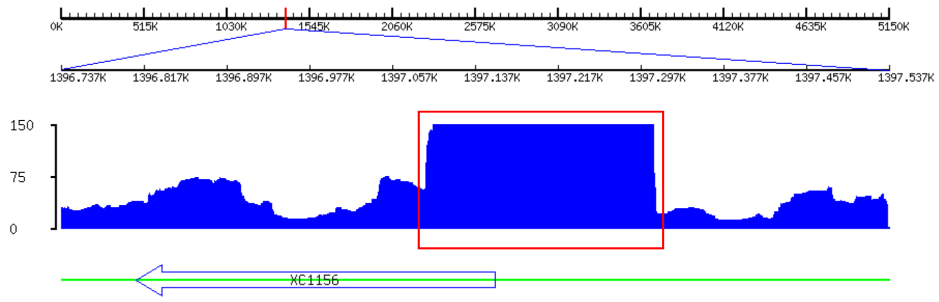

Transcript	Start	End	Length (bp)	RPKM	Inside gene	Overlap gene	Upstream gene	Downstream gene
TT176	1412286	1412380	95	194.1	Null	XC1166	XC1165	XC1167

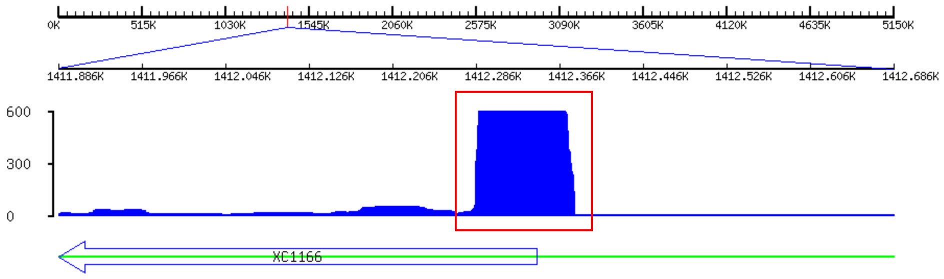

Transcript	Start	End	Length (bp)	RPKM	Inside gene	Overlap gene	Upstream gene	Downstream gene
TT177	1417998	1418254	256	1185.6	Null	XC1172	XC1171	XC1173

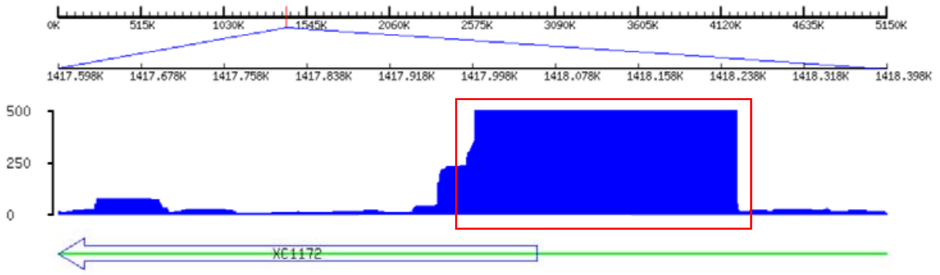

Transcript	Start	End	Length (bp)	RPKM	Inside gene	Overlap gene	Upstream gene	Downstream gene
TT178	1418118	1418254	136	1567.7	Null	Null	XC1172	XC1173

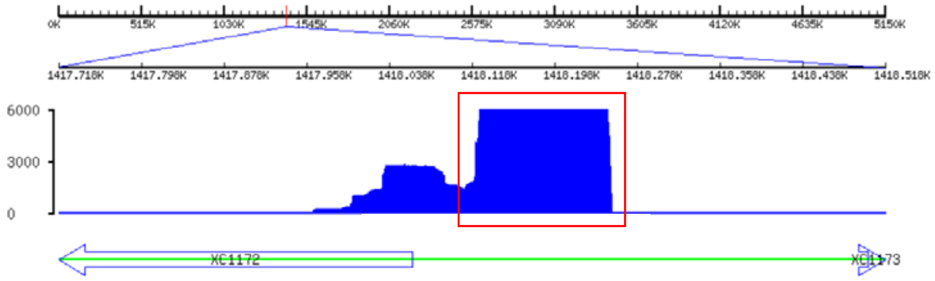

Transcript	Start	End	Length (bp)	RPKM	Inside gene	Overlap gene	Upstream gene	Downstream gene
TT179	1427477	1427565	89	32.9	Null	XC1177	XC1176	XC1178
TT180	1427619	1427690	71	29.1	Null	Null	XC1176	XC1178

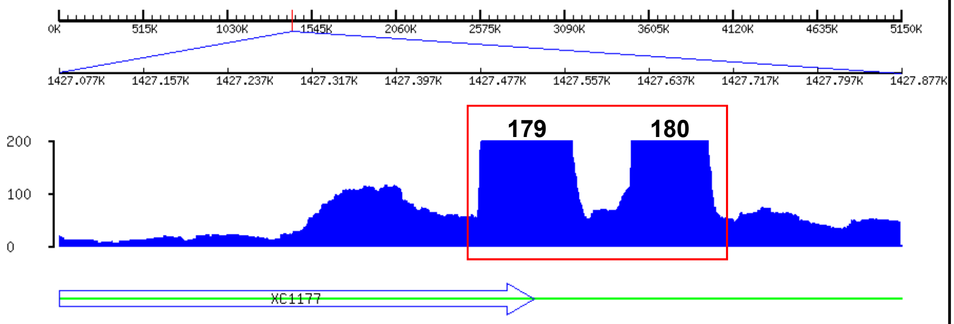

Transcript	Start	End	Length (bp)	RPKM	Inside gene	Overlap gene	Upstream gene	Downstream gene
TT181	1469473	1469563	91	354.5	Null	XC1210	XC1209	XC1211

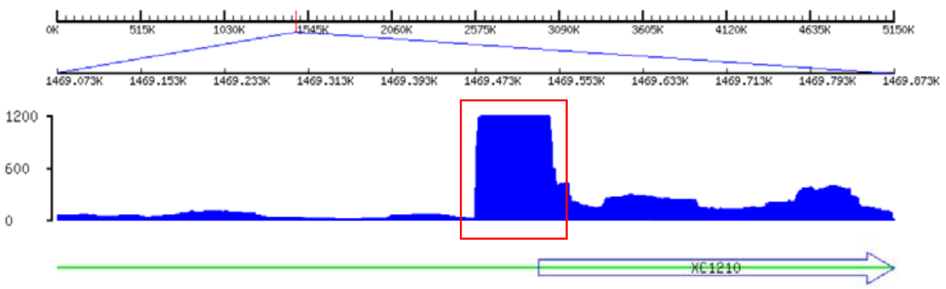

Transcript	Start	End	Length (bp)	RPKM	Inside gene	Overlap gene	Upstream gene	Downstream gene
TT182	1508410	1508509	100	150.2	Null	XC1234	XC1233	XC1235

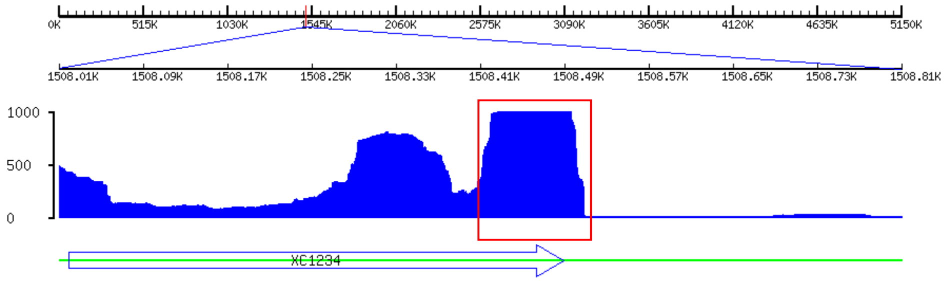

Transcript	Start	End	Length (bp)	RPKM	Inside gene	Overlap gene	Upstream gene	Downstream gene
TT183	1511287	1511378	92	150.7	Null	XC1235	XC1234	XC1236

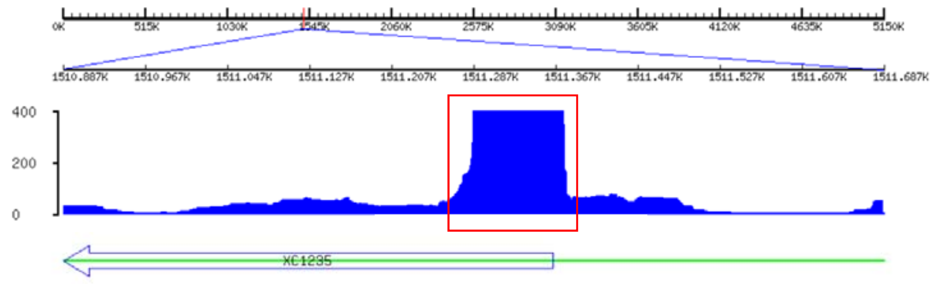

Transcript	Start	End	Length (bp)	RPKM	Inside gene	Overlap gene	Upstream gene	Downstream gene
TT184	1531053	1531262	210	456.5	Null	XC1251	XC1250	XC1252

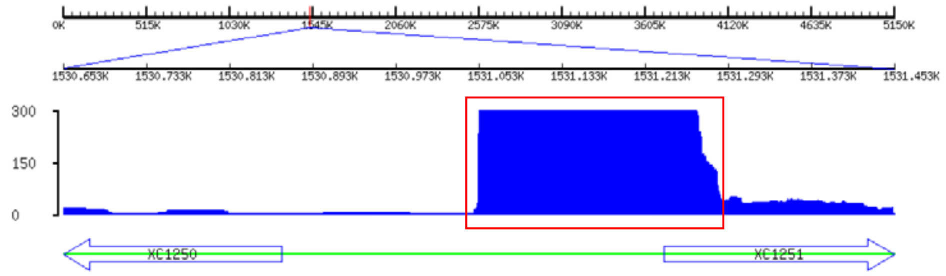

Transcript	Start	End	Length (bp)	RPKM	Inside gene	Overlap gene	Upstream gene	Downstream gene
TT185	1558850	1558916	66	16.2	Null	Null	XC1270	XC1271

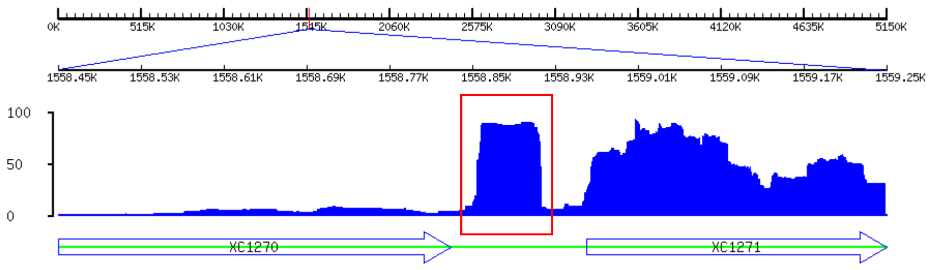

Transcript	Start	End	Length (bp)	RPKM	Inside gene	Overlap gene	Upstream gene	Downstream gene
TT186	1577666	1577921	256	92.3	XC1289	Null	XC1288	XC1290

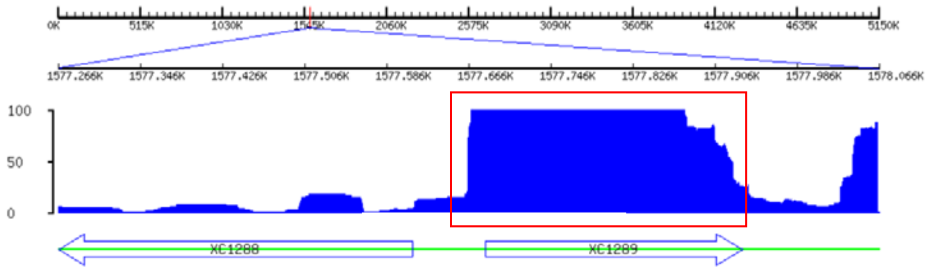

Transcript	Start	End	Length (bp)	RPKM	Inside gene	Overlap gene	Upstream gene	Downstream gene
TT187	1578022	1578382	360	60.6	Null	XC1290	XC1289	XC1291

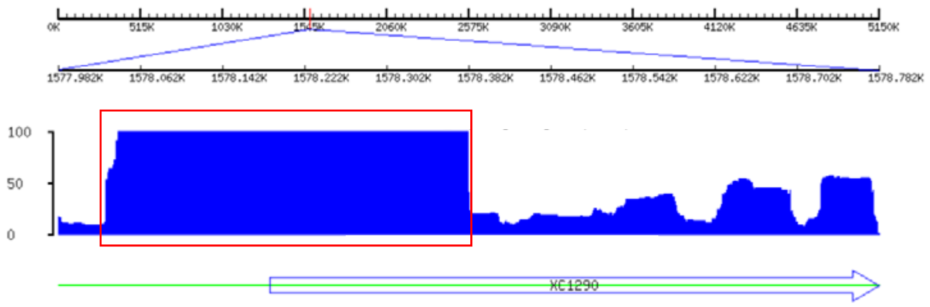

Transcript	Start	End	Length (bp)	RPKM	Inside gene	Overlap gene	Upstream gene	Downstream gene
TT188	1578816	1578872	57	71.5	XC1290	Null	XC1289	XC1291

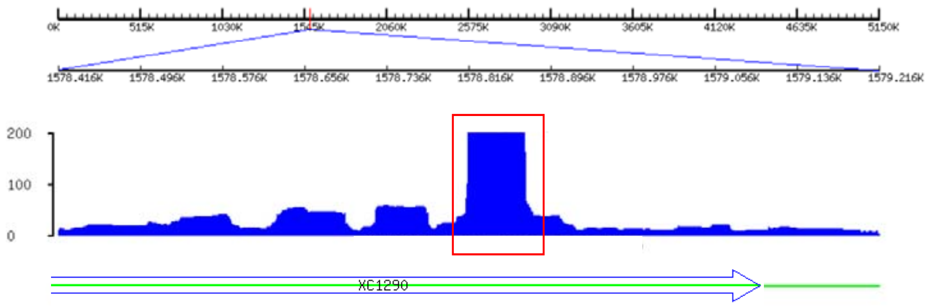

Transcript	Start	End	Length (bp)	RPKM	Inside gene	Overlap gene	Upstream gene	Downstream gene
TT189	1594827	1594894	68	53.7	Null	XC1303	XC1302	XC1304

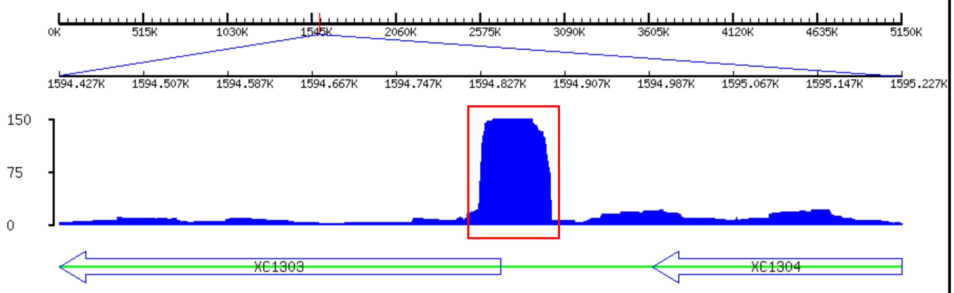

Transcript	Start	End	Length (bp)	RPKM	Inside gene	Overlap gene	Upstream gene	Downstream gene
TT190	1596957	1597013	57	45.4	XC1305	Null	XC1304	XC1306

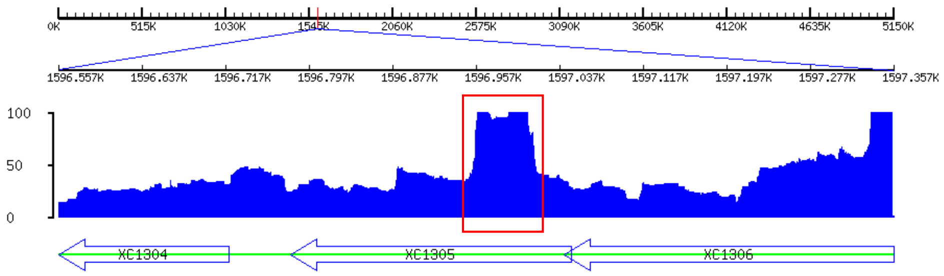

Transcript	Start	End	Length (bp)	RPKM	Inside gene	Overlap gene	Upstream gene	Downstream gene
TT191	1600577	1600670	94	300.9	Null	XC1311	XC1310	XC1312

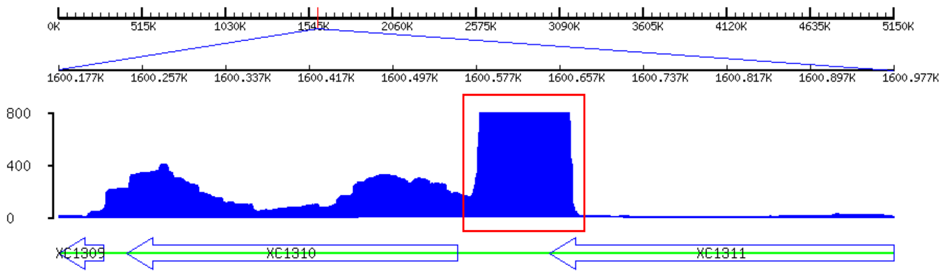

Transcript	Start	End	Length (bp)	RPKM	Inside gene	Overlap gene	Upstream gene	Downstream gene
TT192	2109208	1609364	157	171.8	Null	XC1321	XC1320	XC1322

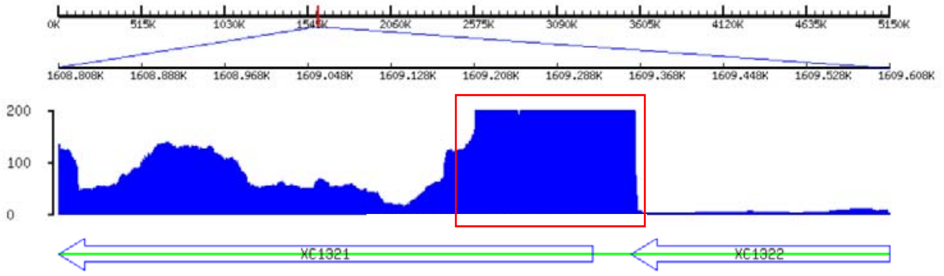

Transcript	Start	End	Length (bp)	RPKM	Inside gene	Overlap gene	Upstream gene	Downstream gene
TT193	1618520	1619034	514	997.4	XC1332	Null	XC1331	XC1333

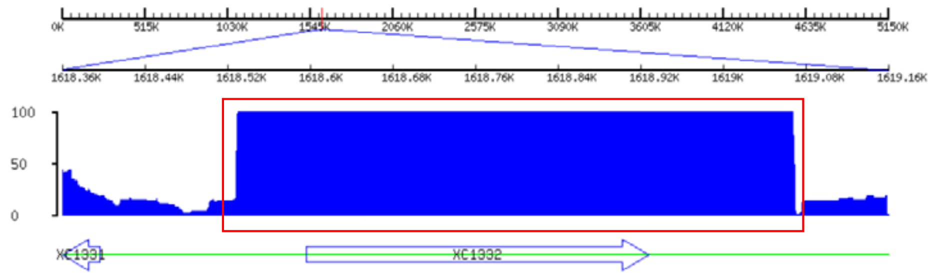

Transcript	Start	End	Length (bp)	RPKM	Inside gene	Overlap gene	Upstream gene	Downstream gene
TT194	1618954	1619030	77	5188.6	Null	Null	XC1332	XC1333

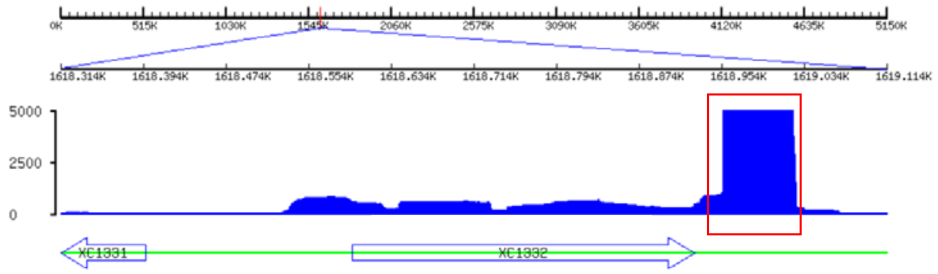

Transcript	Start	End	Length (bp)	RPKM	Inside gene	Overlap gene	Upstream gene	Downstream gene
TT195	1629893	1629982	90	80.3	XC1343	Null	XC1342	XC1344
TT196	1630002	1630092	91	38.6	XC1344	Null	XC1342	XC1344

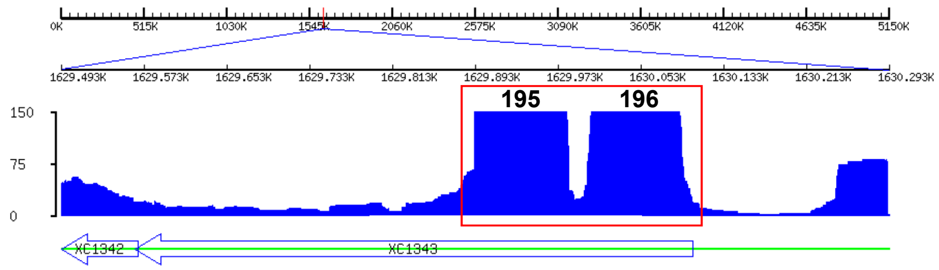

Transcript	Start	End	Length (bp)	RPKM	Inside gene	Overlap gene	Upstream gene	Downstream gene
TT197	1633658	1633717	60	596.1	Null	Null	XC1346	XC1347

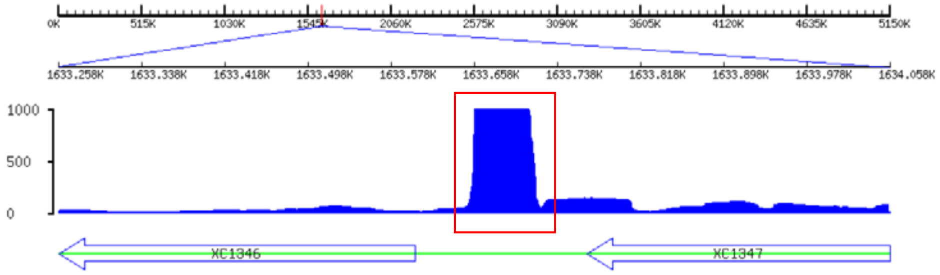

Transcript	Start	End	Length (bp)	RPKM	Inside gene	Overlap gene	Upstream gene	Downstream gene
TT198	1634567	1634660	94	193.9	XC1347	Null	XC1346	XC1348

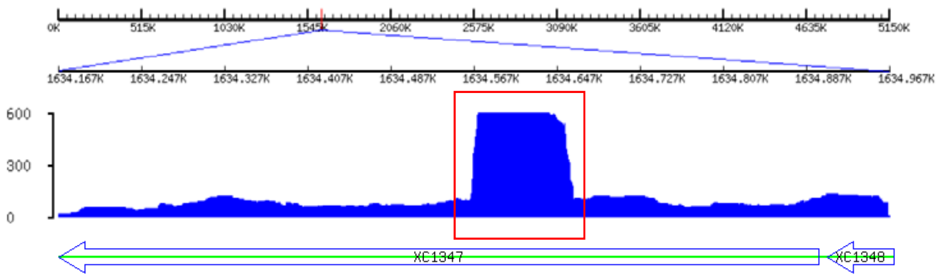

Transcript	Start	End	Length (bp)	RPKM	Inside gene	Overlap gene	Upstream gene	Downstream gene
TT199	1638354	1638439	86	37.8	Null	XC1352	XC1351	XC1353

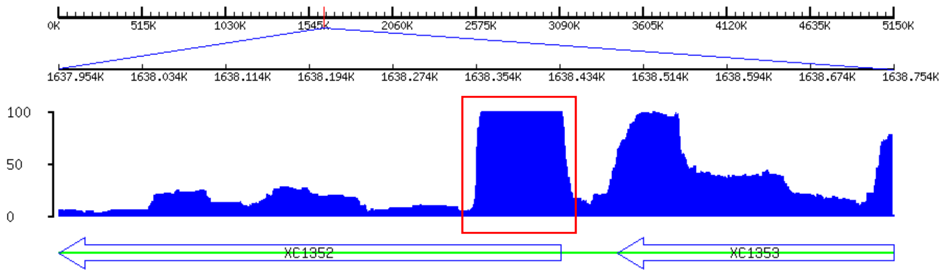

Transcript	Start	End	Length (bp)	RPKM	Inside gene	Overlap gene	Upstream gene	Downstream gene
TT200	1653515	1653608	94	186.7	Null	XC1369	XC1368	XC1370
TT201	1653608	1653737	58	584.9	XC1369	Null	XC1368	XC1370

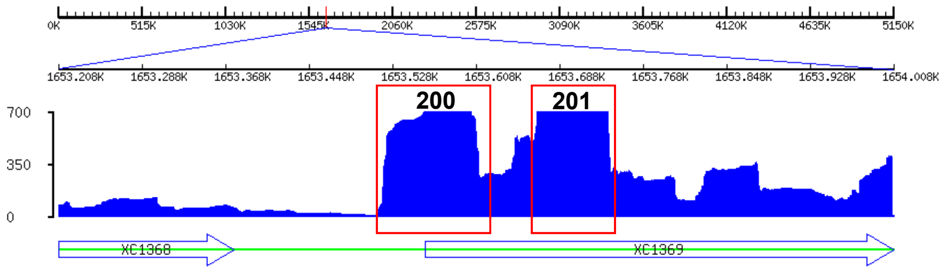

Transcript	Start	End	Length (bp)	RPKM	Inside gene	Overlap gene	Upstream gene	Downstream gene
TT202	1654243	1654307	64	826.2	Null	Null	XC1369	XC1370

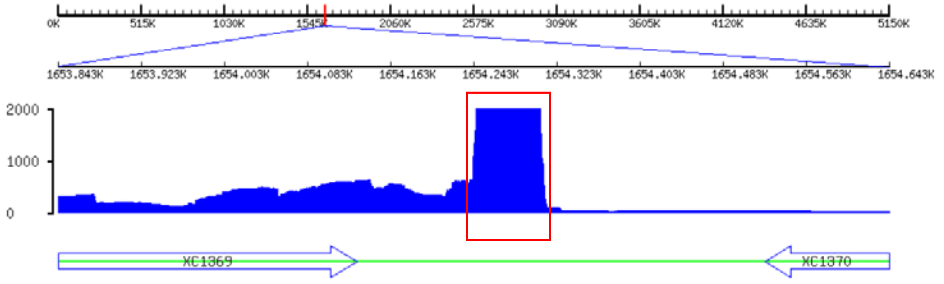

Transcript	Start	End	Length (bp)	RPKM	Inside gene	Overlap gene	Upstream gene	Downstream gene
TT203	1664428	1664494	66	14.3	XC1377	Null	XC1376	XC1378

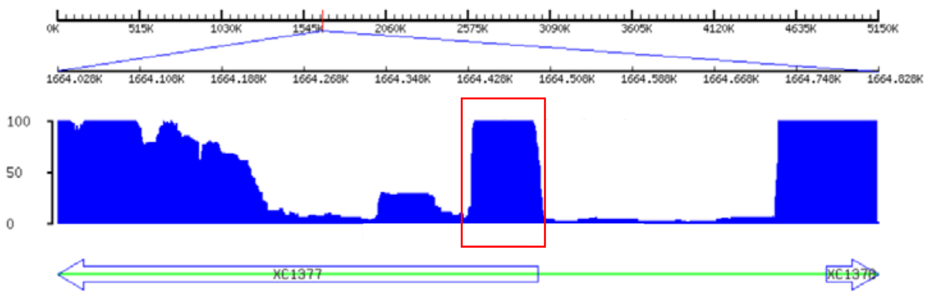

Transcript	Start	End	Length (bp)	RPKM	Inside gene	Overlap gene	Upstream gene	Downstream gene
TT204	1666368	1666457	90	59.7	XC1380	Null	XC1379	XC1381

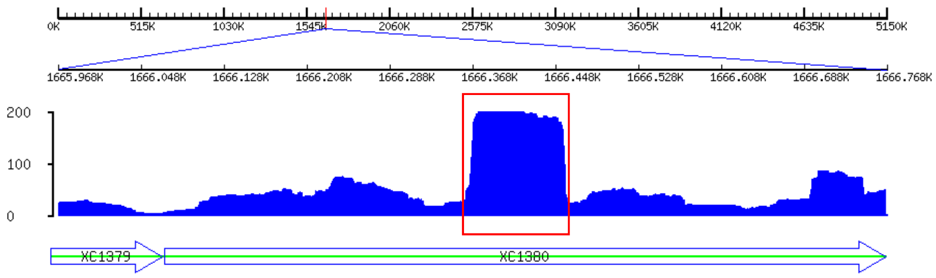

Transcript	Start	End	Length (bp)	RPKM	Inside gene	Overlap gene	Upstream gene	Downstream gene
TT205	1671801	1671860	60	154.9	Null	Null	XC1383	XC1384

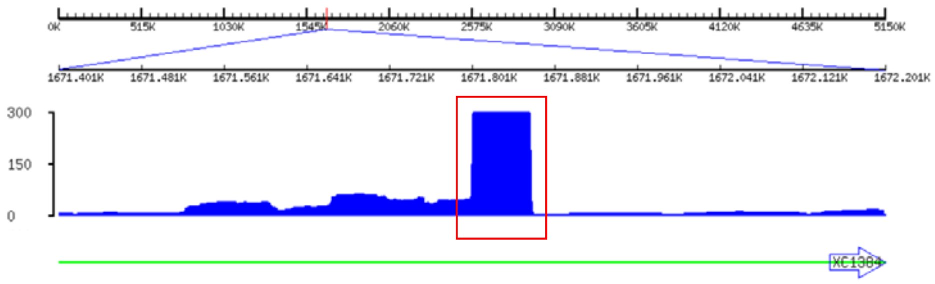

Transcript	Start	End	Length (bp)	RPKM	Inside gene	Overlap gene	Upstream gene	Downstream gene
TT206	1673650	1673706	57	202.4	XC1385	Null	XC1384	XC1386

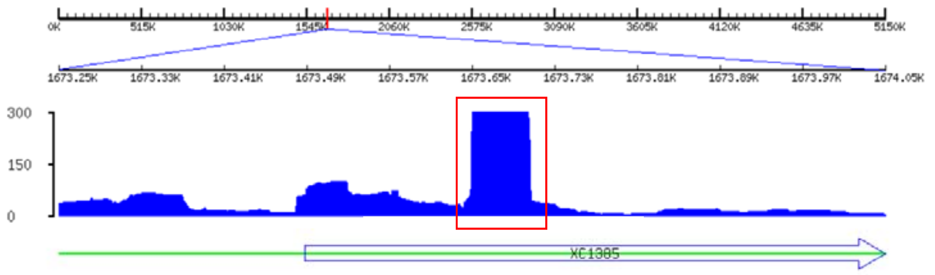

Transcript	Start	End	Length (bp)	RPKM	Inside gene	Overlap gene	Upstream gene	Downstream gene
TT207	1690686	1690779	94	56.2	Null	Null	XC1401	XC1402

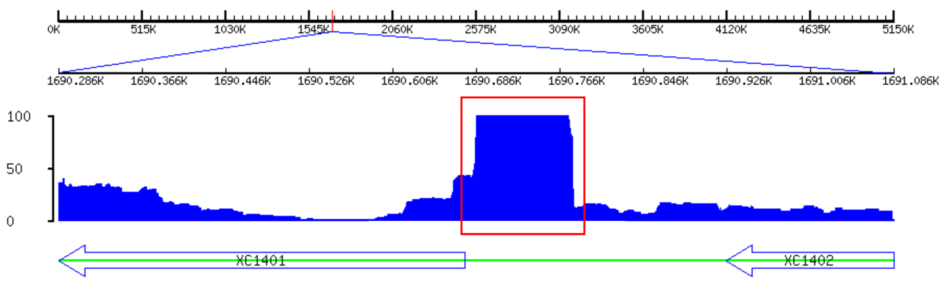

Transcript	Start	End	Length (bp)	RPKM	Inside gene	Overlap gene	Upstream gene	Downstream gene
TT208	1725118	1725256	139	96.3	Null	XC1430	XC1428	XC1431
TT209	1725291	1725380	90	97.8	XC1430	Null	XC1429	XC1431

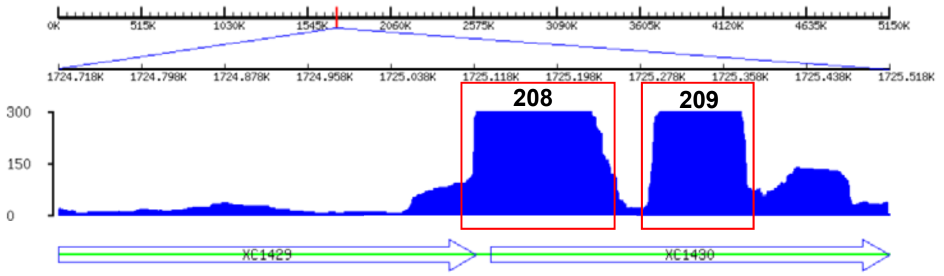

Transcript	Start	End	Length (bp)	RPKM	Inside gene	Overlap gene	Upstream gene	Downstream gene
TT210	1747942	1748009	68	157.8	Null	Null	XC1450	XC1451

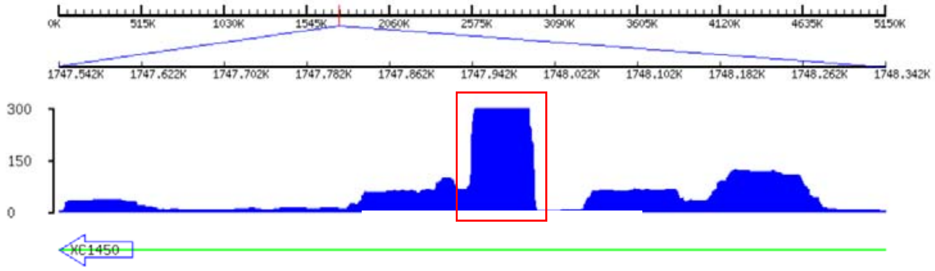

Transcript	Start	End	Length (bp)	RPKM	Inside gene	Overlap gene	Upstream gene	Downstream gene
TT211	1750038	1750126	89	214.1	XC1451	Null	XC1450	XC1452

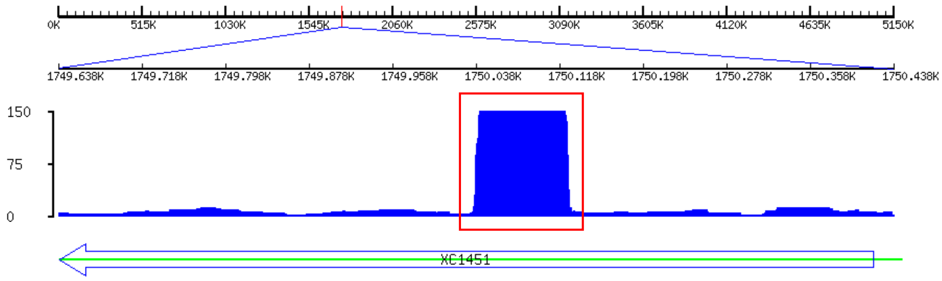

Transcript	Start	End	Length (bp)	RPKM	Inside gene	Overlap gene	Upstream gene	Downstream gene
TT212	1751067	1751161	95	110.3	Null	Null	XC1451	XC1452

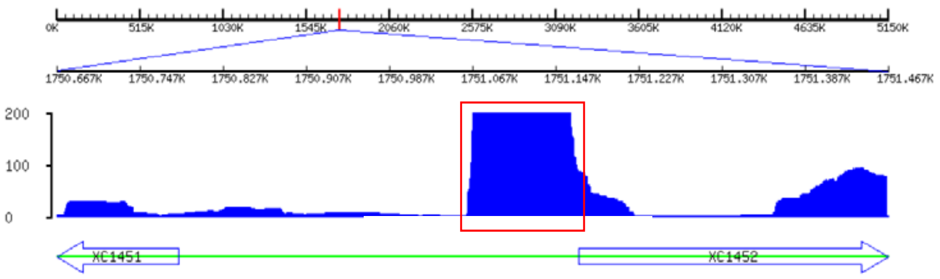

Transcript	Start	End	Length (bp)	RPKM	Inside gene	Overlap gene	Upstream gene	Downstream gene
TT213	1791899	1791966	68	72.1	Null	Null	XC1485	XC1486

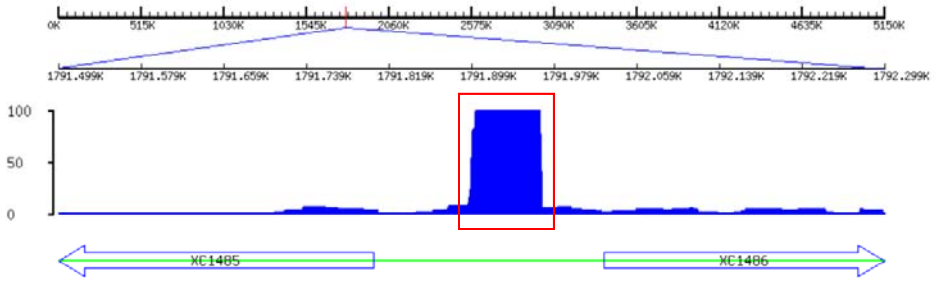

Transcript	Start	End	Length (bp)	RPKM	Inside gene	Overlap gene	Upstream gene	Downstream gene
TT214	1794789	1794847	59	23.4	Null	Null	XC1488	XC1489

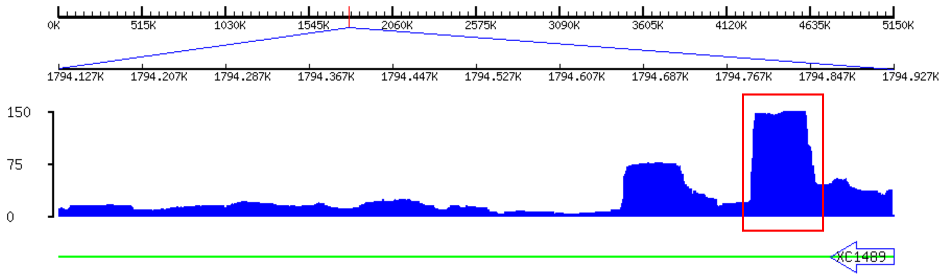

Transcript	Start	End	Length (bp)	RPKM	Inside gene	Overlap gene	Upstream gene	Downstream gene
TT215	1795129	1795191	63	1795129	Null	XC1489	XC1488	XC1490

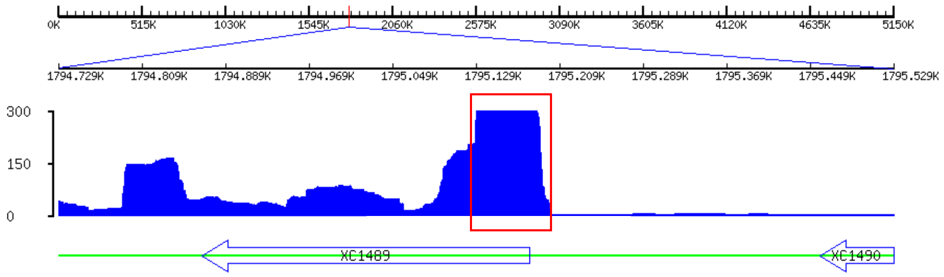

Transcript	Start	End	Length (bp)	RPKM	Inside gene	Overlap gene	Upstream gene	Downstream gene
TT216	1798597	1798657	61	206.9	Null	Null	XC1492	XC1493

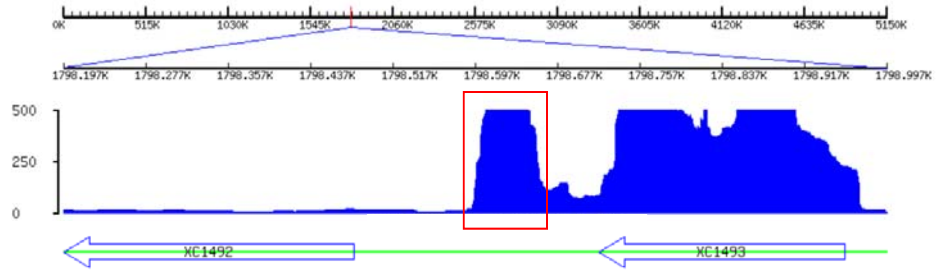

Transcript	Start	End	Length (bp)	RPKM	Inside gene	Overlap gene	Upstream gene	Downstream gene
TT217	1798594	1798658	65	151.2	Null	Null	XC1492	XC1493
TT218	1798734	1798971	237	160.1	XC1493	Null	XC1492	XC1494

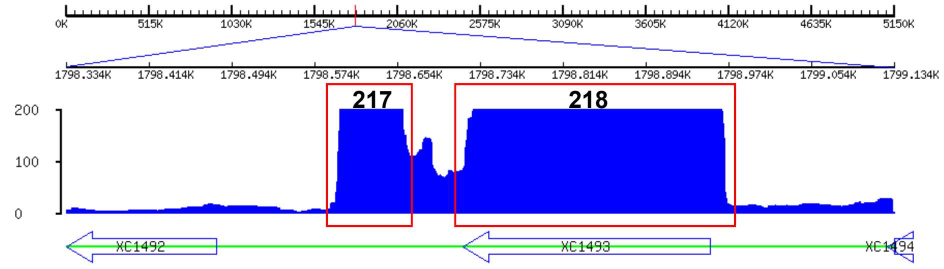

Transcript	Start	End	Length (bp)	RPKM	Inside gene	Overlap gene	Upstream gene	Downstream gene
TT219	1807102	1807157	56	249.3	Null	XC1501	XC1500	XC1502
TT220	1807496	1807551	56	211.5	Null	XC1501	XC1500	XC1502

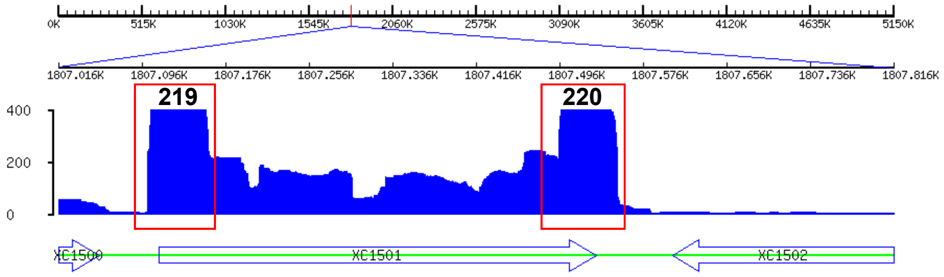

Transcript	Start	End	Length (bp)	RPKM	Inside gene	Overlap gene	Upstream gene	Downstream gene
TT221	1814332	1814398	67	105.9	Null	Null	XC1507	XC1508

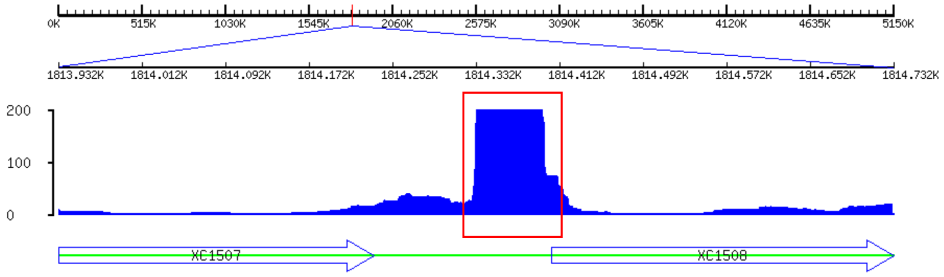

Transcript	Start	End	Length (bp)	RPKM	Inside gene	Overlap gene	Upstream gene	Downstream gene
TT222	1830452	1830516	65	71.7	XC1520	Null	XC1519	XC1521

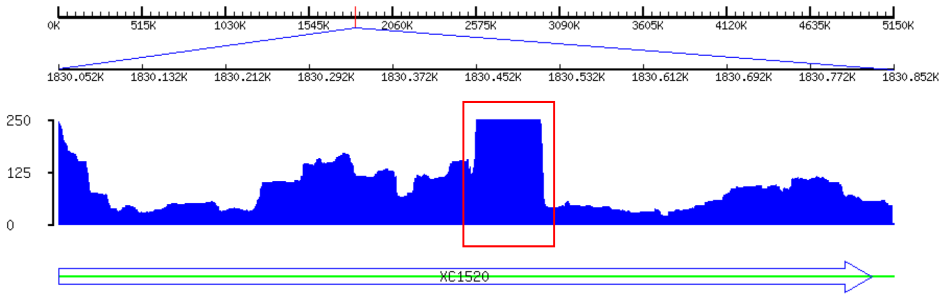

Transcript	Start	End	Length (bp)	RPKM	Inside gene	Overlap gene	Upstream gene	Downstream gene
TT223	1843442	1843503	62	376.9	Null	XC1534	XC1533	XC1535

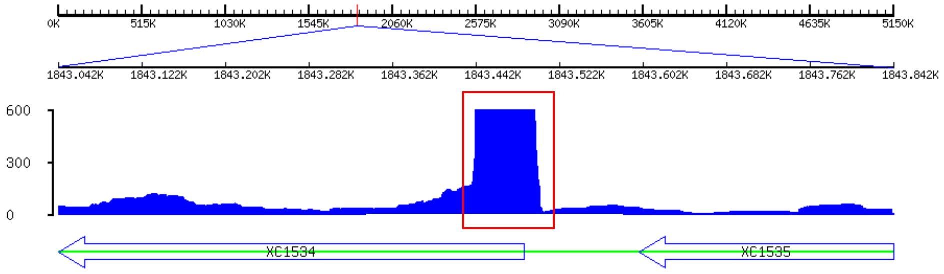

Transcript	Start	End	Length (bp)	RPKM	Inside gene	Overlap gene	Upstream gene	Downstream gene
TT224	1844170	1844248	79	54.9	Null	XC1535/ XC1536	XC1534	XC1537

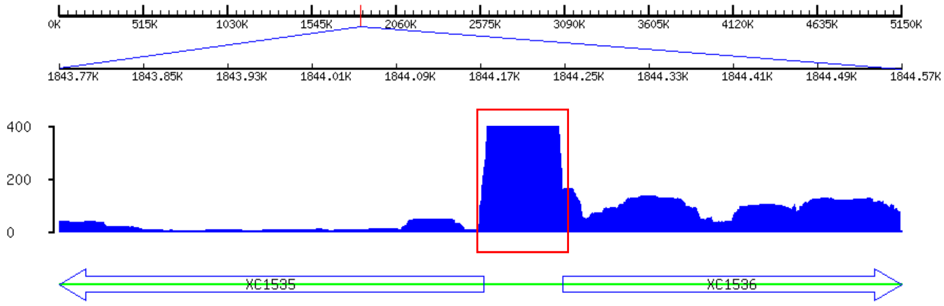

Transcript	Start	End	Length (bp)	RPKM	Inside gene	Overlap gene	Upstream gene	Downstream gene
TT225	1857558	1857611	54	90.3	XC1545	Null	XC1544	XC1546

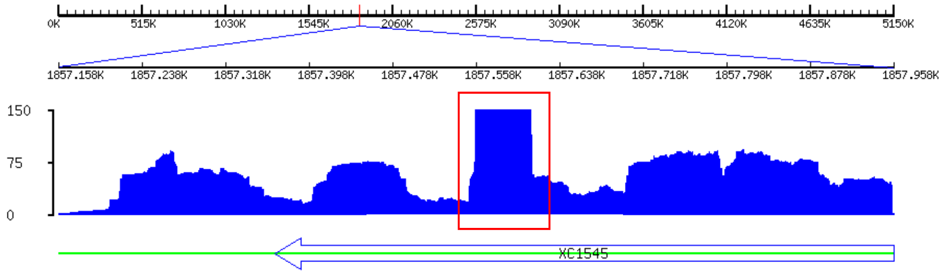

Transcript	Start	End	Length (bp)	RPKM	Inside gene	Overlap gene	Upstream gene	Downstream gene
TT226	1878565	1878635	70	15.2	XC1560	Null	XC1559	XC1561
TT227	1878860	1878944	85	32.8	Null	Null	XC1560	XC1561

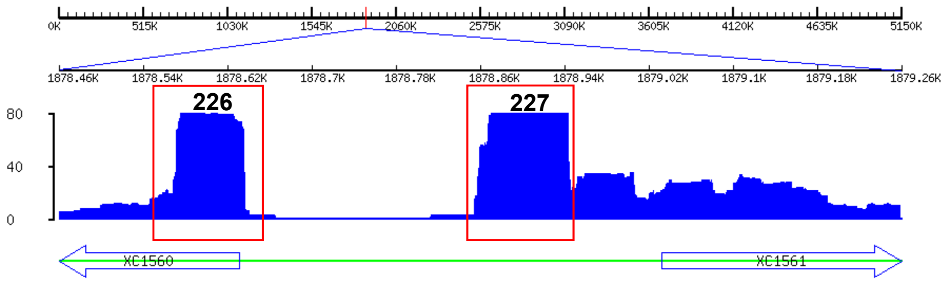

Transcript	Start	End	Length (bp)	RPKM	Inside gene	Overlap gene	Upstream gene	Downstream gene
TT228	1891848	1892048	200	179.9	Null	XC1572 /XC1573	XC1571	XC1574

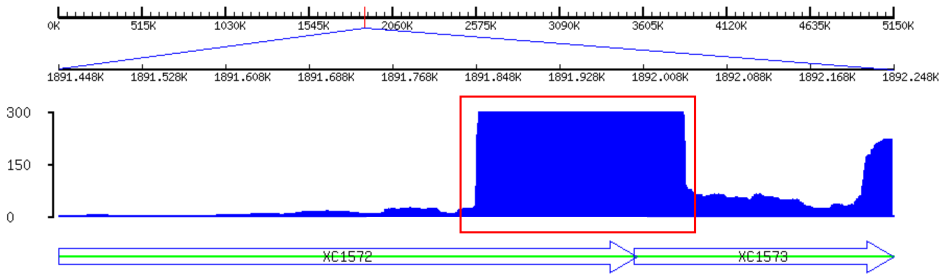

Transcript	Start	End	Length (bp)	RPKM	Inside gene	Overlap gene	Upstream gene	Downstream gene
TT229	1896637	1896730	94	151.1	Null	XC1578	XC1577	XC1579

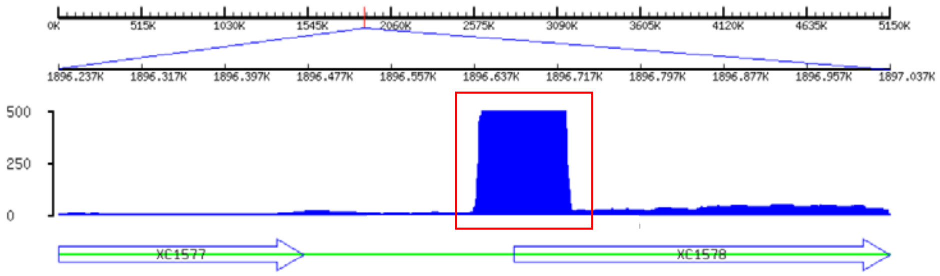

Transcript	Start	End	Length (bp)	RPKM	Inside gene	Overlap gene	Upstream gene	Downstream gene
TT230	1908312	1908365	53	2282.1	XC4338	Null	XC1588	XC1589

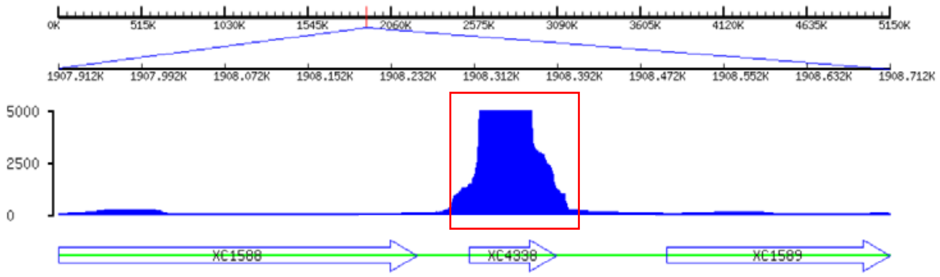

Transcript	Start	End	Length (bp)	RPKM	Inside gene	Overlap gene	Upstream gene	Downstream gene
TT231	1923549	1923619	70	4672.8	XC4339	Null	XC1602	XC1603

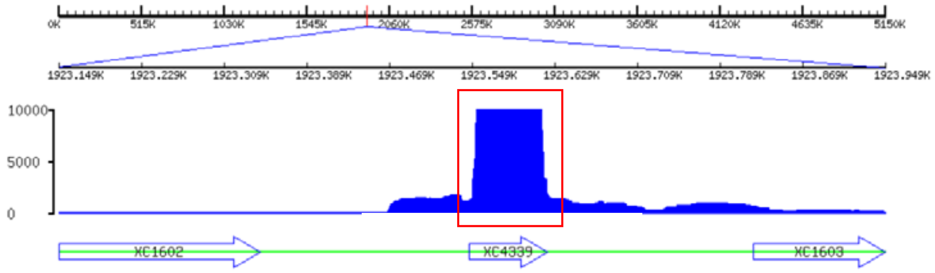

Transcript	Start	End	Length (bp)	RPKM	Inside gene	Overlap gene	Upstream gene	Downstream gene
TT232	1925066	1925156	91	93.1	XC1604	Null	XC1603	XC1605
TT233	1925180	1925269	90	134.1	XC1604	Null	XC1603	XC1605

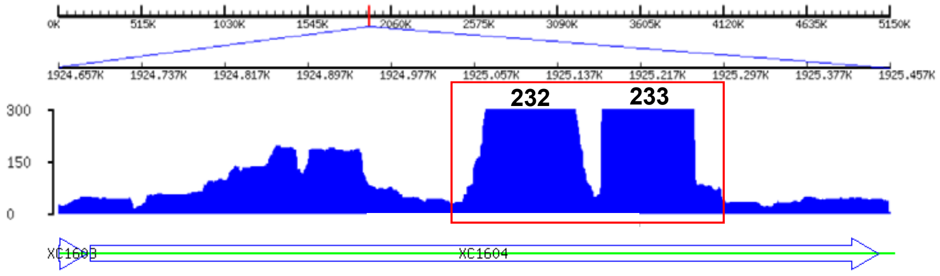

Transcript	Start	End	Length (bp)	RPKM	Inside gene	Overlap gene	Upstream gene	Downstream gene
TT234	1925989	1926039	51	152.9	XC1605	Null	XC1604	XC1606

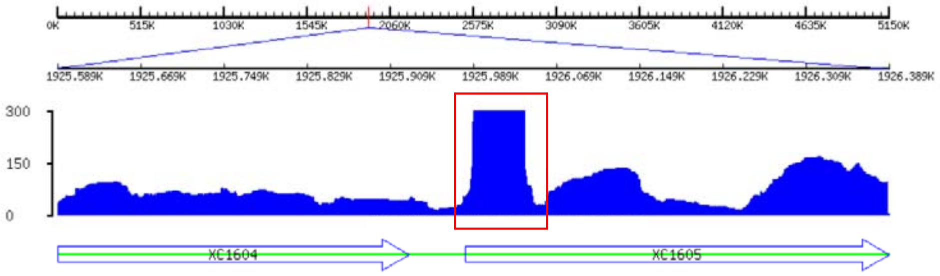

Transcript	Start	End	Length (bp)	RPKM	Inside gene	Overlap gene	Upstream gene	Downstream gene
TT235	1935900	1935956	57	51.7	Null	XC1612	XC1611	XC1613

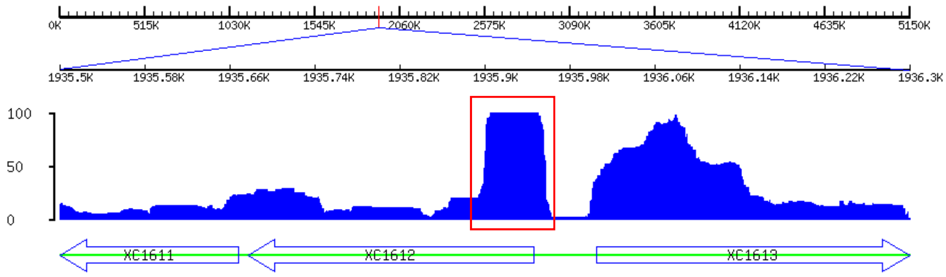

Transcript	Start	End	Length (bp)	RPKM	Inside gene	Overlap gene	Upstream gene	Downstream gene
TT236	1952411	1952494	83	3231.1	Null	XC4340	XC1626	XC1627

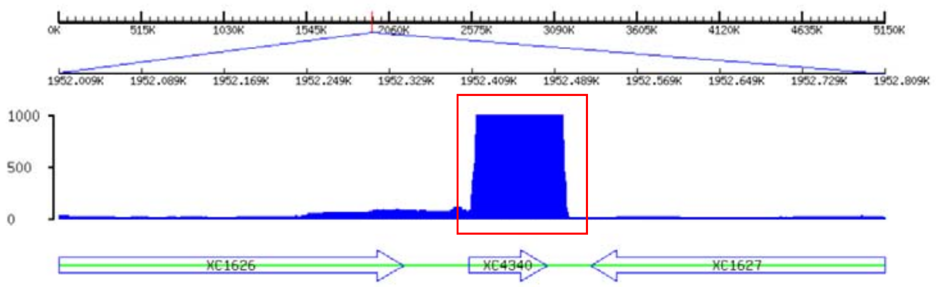

Transcript	Start	End	Length (bp)	RPKM	Inside gene	Overlap gene	Upstream gene	Downstream gene
TT237	1955313	1955402	90	2951.2	XC4341	Null	XC1628	XC1629

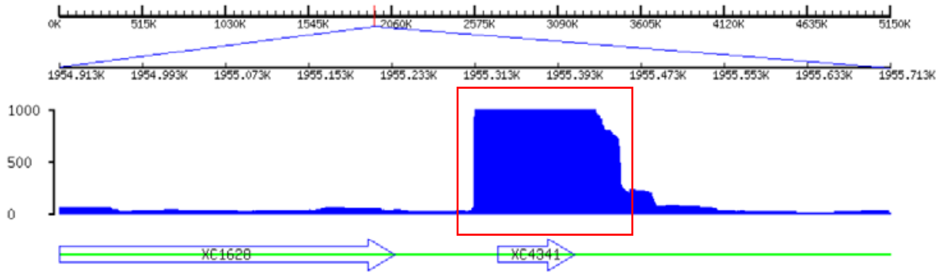

Transcript	Start	End	Length (bp)	RPKM	Inside gene	Overlap gene	Upstream gene	Downstream gene
TT238	1955793	1955849	57	297.2	Null	XC1629	XC4341	XC1630

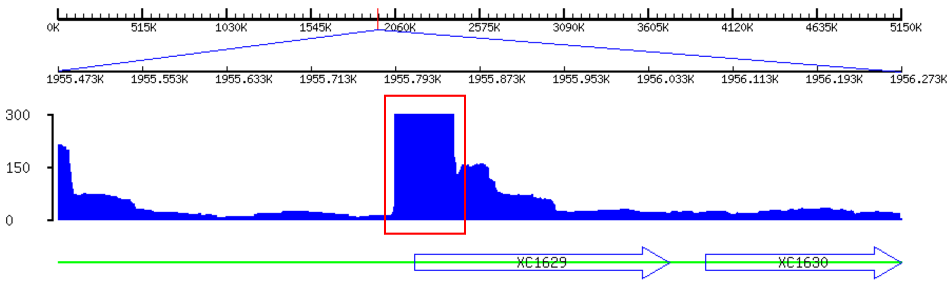

Transcript	Start	End	Length (bp)	RPKM	Inside gene	Overlap gene	Upstream gene	Downstream gene
TT239	1958154	1958316	162	365.4	Null	XC1631	XC1630	XC1632

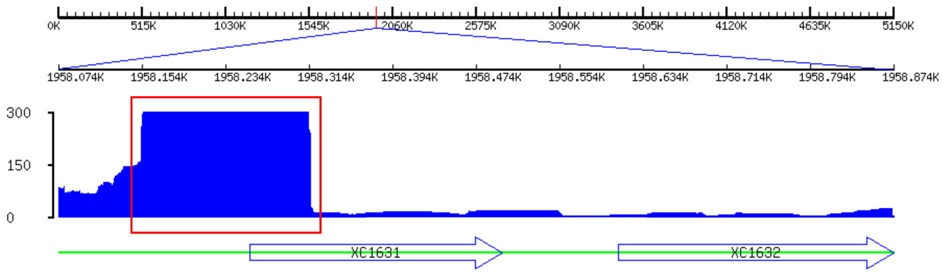

Transcript	Start	End	Length (bp)	RPKM	Inside gene	Overlap gene	Upstream gene	Downstream gene
TT240	1964190	1964283	94	226.1	XC1638	Null	XC1637	XC1639

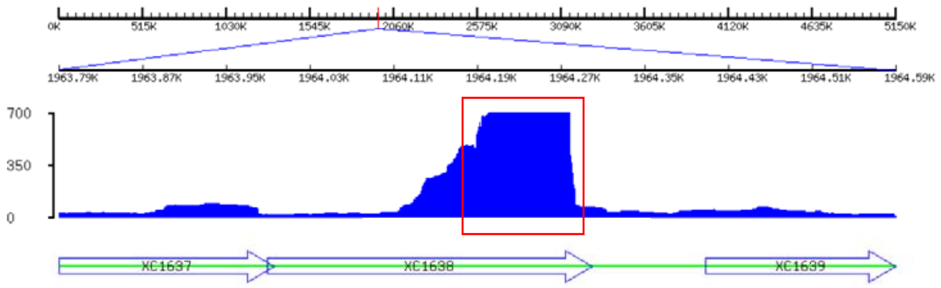

Transcript	Start	End	Length (bp)	RPKM	Inside gene	Overlap gene	Upstream gene	Downstream gene
TT241	1987096	1987333	237	924.1	Null	XC1650	XC1649	XC1651

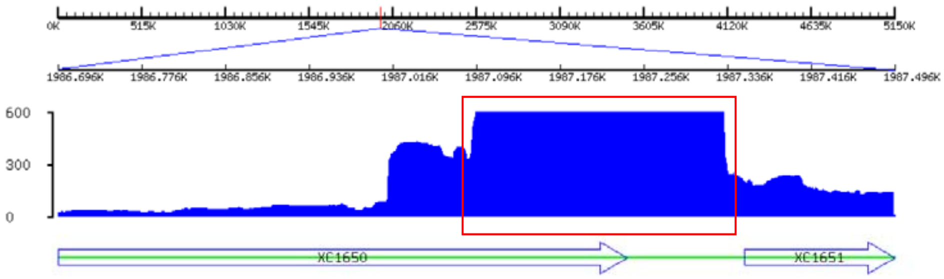

Transcript	Start	End	Length (bp)	RPKM	Inside gene	Overlap gene	Upstream gene	Downstream gene
TT242	1987898	1988088	191	419.5	Null	XC1652	XC1651	XC1653

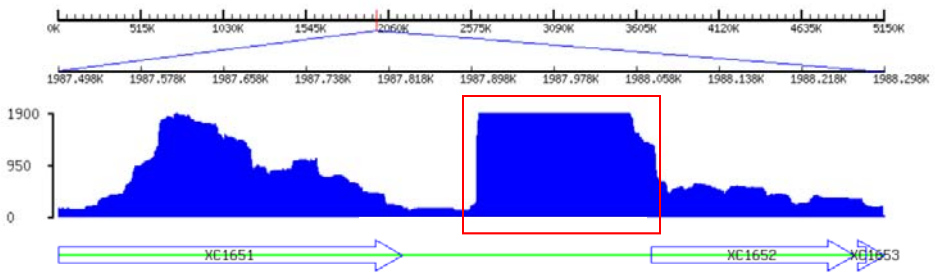

Transcript	Start	End	Length (bp)	RPKM	Inside gene	Overlap gene	Upstream gene	Downstream gene
TT243	1988338	1988392	55	333.3	XC1653	Null	XC1652	XC1654

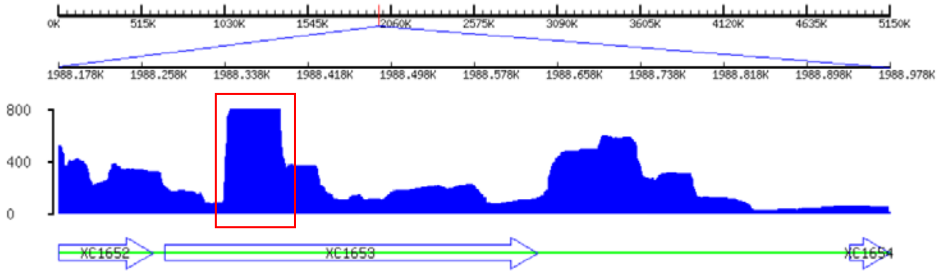

Transcript	Start	End	Length (bp)	RPKM	Inside gene	Overlap gene	Upstream gene	Downstream gene
TT244	1992387	1992456	69	74.6	XC1655	Null	XC1654	XC1656
TT245	1992504	1992592	88	62.9	XC1656	Null	XC1655	XC1657

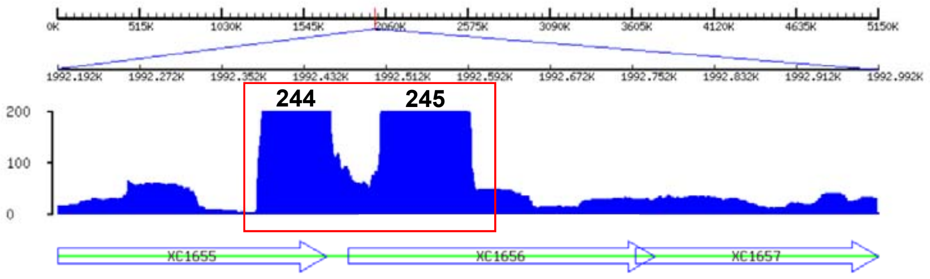

Transcript	Start	End	Length (bp)	RPKM	Inside gene	Overlap gene	Upstream gene	Downstream gene
TT246	1993177	1993237	60	29595.6	XC4342	Null	XC1657	XC1658

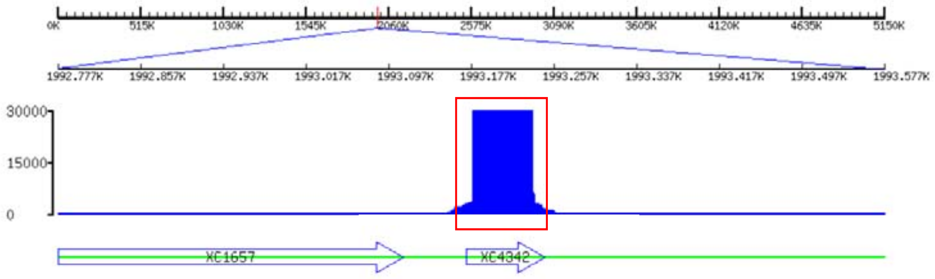

Transcript	Start	End	Length (bp)	RPKM	Inside gene	Overlap gene	Upstream gene	Downstream gene
TT247	1993596	1993830	235	639.4	Null	XC1658	XC4342	XC1659

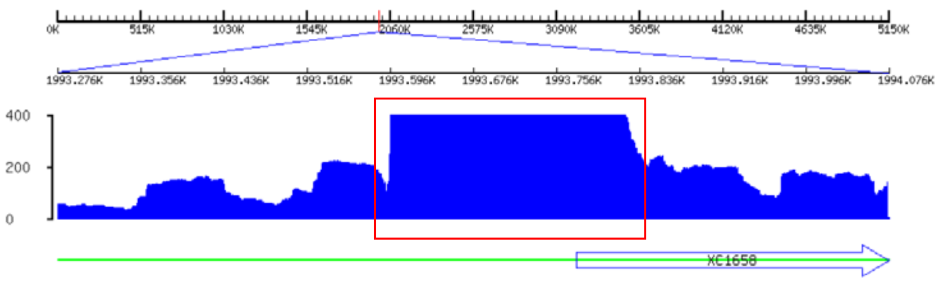

Transcript	Start	End	Length (bp)	RPKM	Inside gene	Overlap gene	Upstream gene	Downstream gene
TT248	1996966	1997054	89	87.1	XC1660	Null	XC1659	XC1661

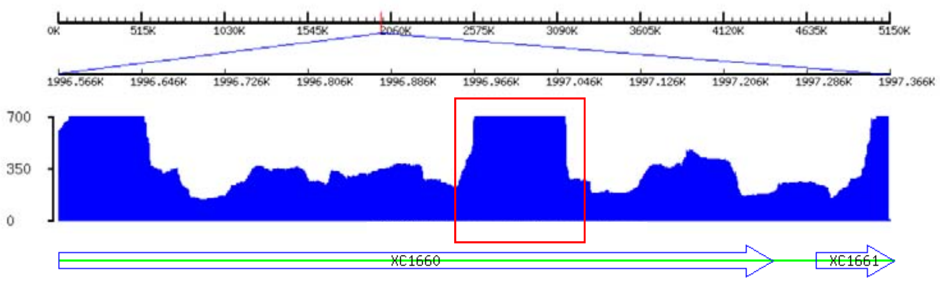

Transcript	Start	End	Length (bp)	RPKM	Inside gene	Overlap gene	Upstream gene	Downstream gene
TT249	2017621	2017680	60	193.4	XC1678	Null	XC1677	XC1679

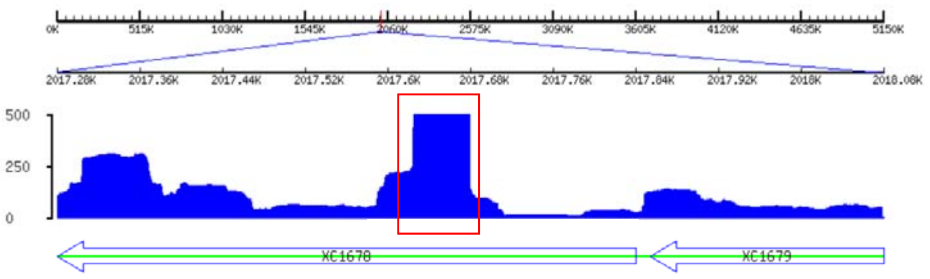

Transcript	Start	End	Length (bp)	RPKM	Inside gene	Overlap gene	Upstream gene	Downstream gene
TT250	2018414	2018507	94	634.9	Null	XC1679	XC1678	XC1680

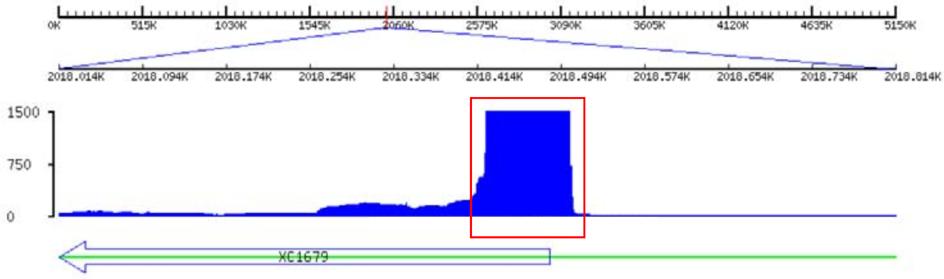

Transcript	Start	End	Length (bp)	RPKM	Inside gene	Overlap gene	Upstream gene	Downstream gene
TT251	2022475	2022552	77	215.1	XC4343	Null	XC1682	XC1683

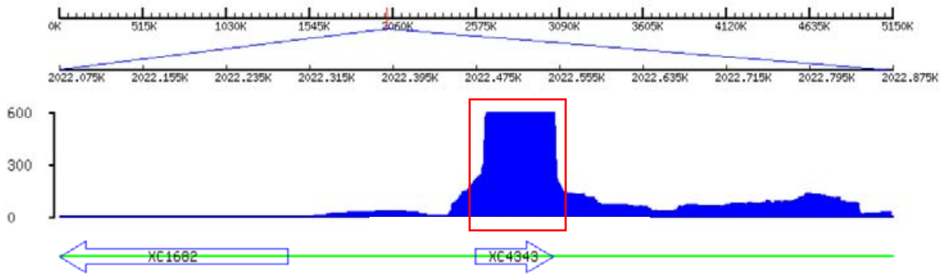

Transcript	Start	End	Length (bp)	RPKM	Inside gene	Overlap gene	Upstream gene	Downstream gene
TT252	2027555	2027808	254	98.9	XC1687	Null	XC1686	XC1688

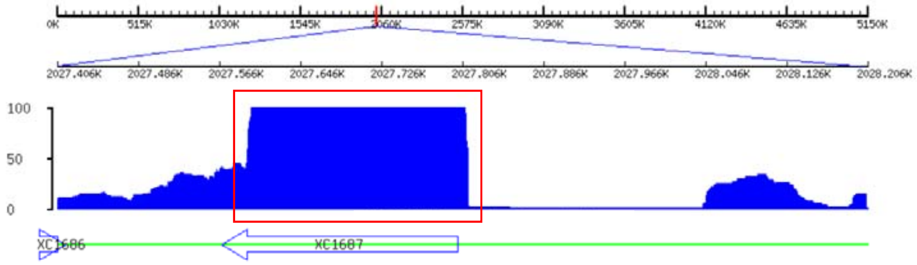

Transcript	Start	End	Length (bp)	RPKM	Inside gene	Overlap gene	Upstream gene	Downstream gene
TT253	2099001	2099060	60	56.7	Null	XC1735	XC1734	XC1736

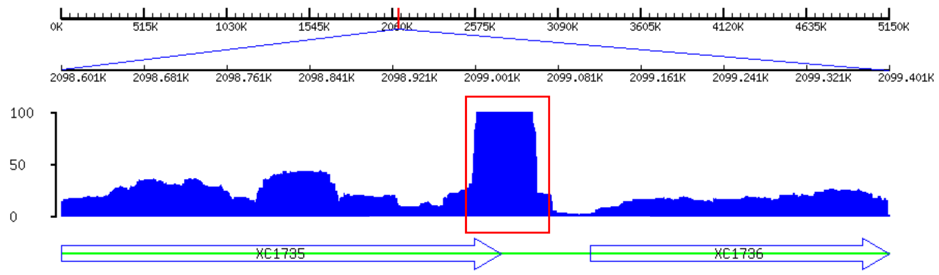

Transcript	Start	End	Length (bp)	RPKM	Inside gene	Overlap gene	Upstream gene	Downstream gene
TT254	2117629	2117706	78	53.3	XC1747	Null	XC1746	XC1748

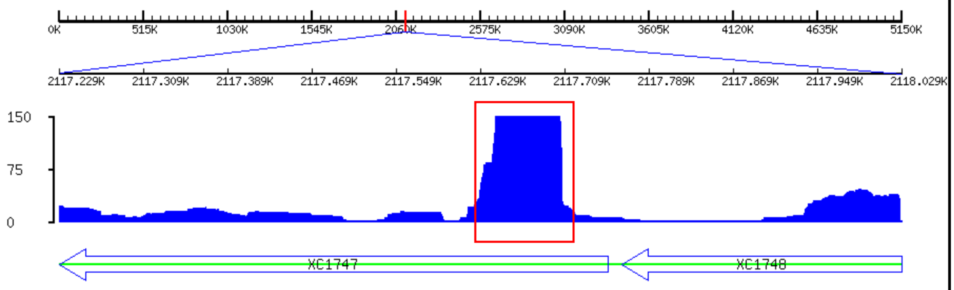

Transcript	Start	End	Length (bp)	RPKM	Inside gene	Overlap gene	Upstream gene	Downstream gene
TT255	2134040	2134267	228	146.5	Null	XC1756, XC1757	XC1755	XC1758

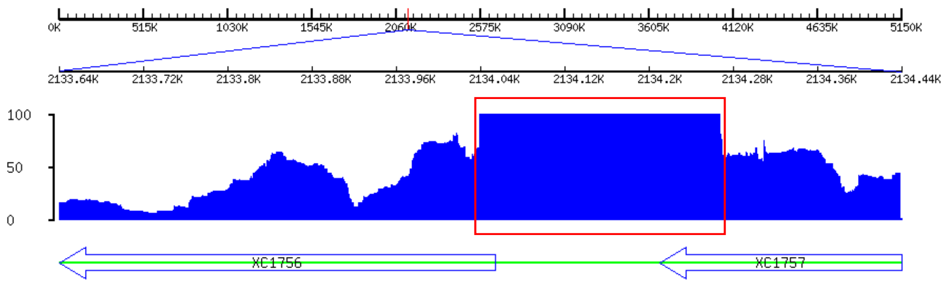

Transcript	Start	End	Length (bp)	RPKM	Inside gene	Overlap gene	Upstream gene	Downstream gene
TT256	2146561	2146651	90	38.4	XC1765	Null	XC1764	XC1766
TT257	2146707	2146801	95	79.6	Null	XC1765	XC1764	XC1766

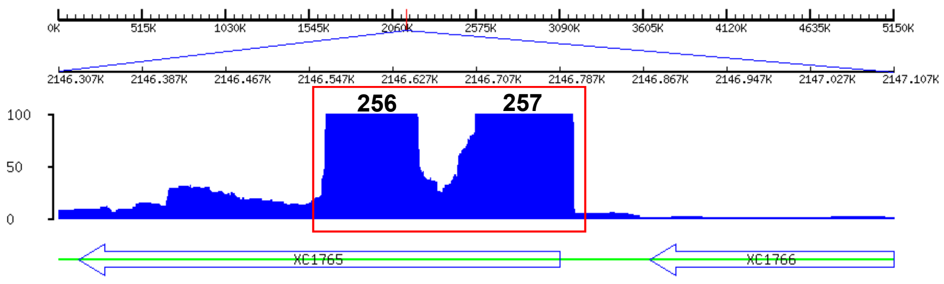

Transcript	Start	End	Length (bp)	RPKM	Inside gene	Overlap gene	Upstream gene	Downstream gene
TT258	2170287	2170349	63	478.5	Null	XC1783	XC1782	XC1784

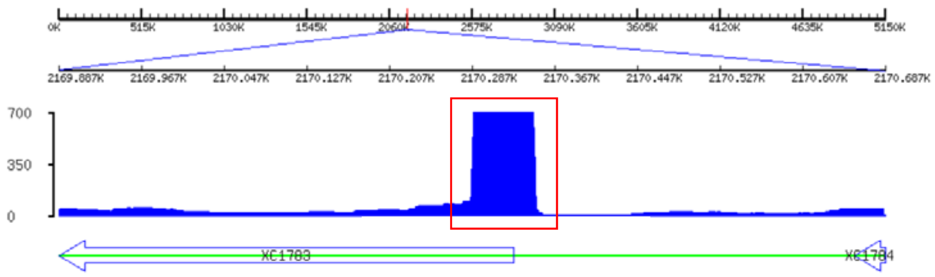

Transcript	Start	End	Length (bp)	RPKM	Inside gene	Overlap gene	Upstream gene	Downstream gene
TT259	2195687	2195760	74	302.4	Null	XC1807	XC1806	XC1808

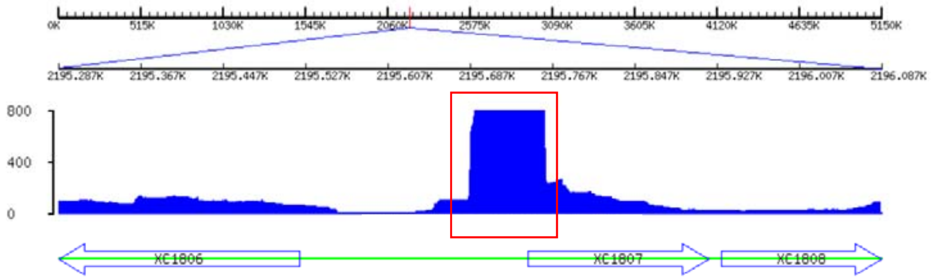

Transcript	Start	End	Length (bp)	RPKM	Inside gene	Overlap gene	Upstream gene	Downstream gene
TT260	2213815	2213865	51	77.2	XC1820	Null	XC1819	XC1821

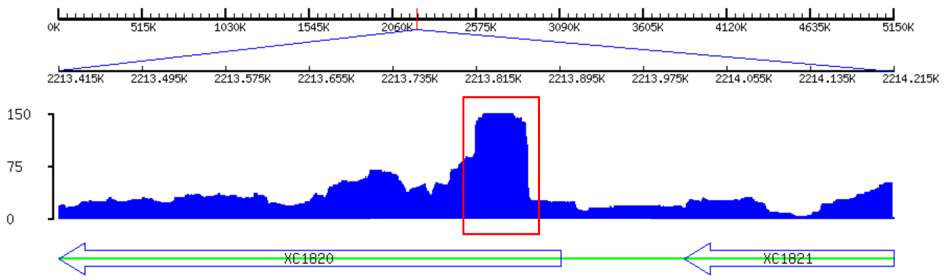

Transcript	Start	End	Length (bp)	RPKM	Inside gene	Overlap gene	Upstream gene	Downstream gene
TT261	2218048	2218110	63	18.3	XC1824	Null	XC1823	XC1825

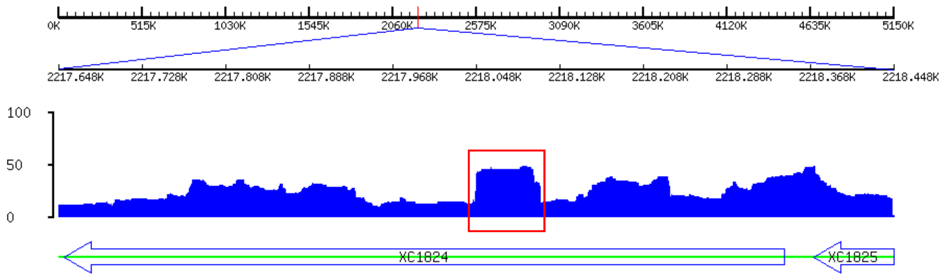

Transcript	Start	End	Length (bp)	RPKM	Inside gene	Overlap gene	Upstream gene	Downstream gene
TT262	2221405	2221619	214	2615.9	Null	XC1828	XC1827	XC1829

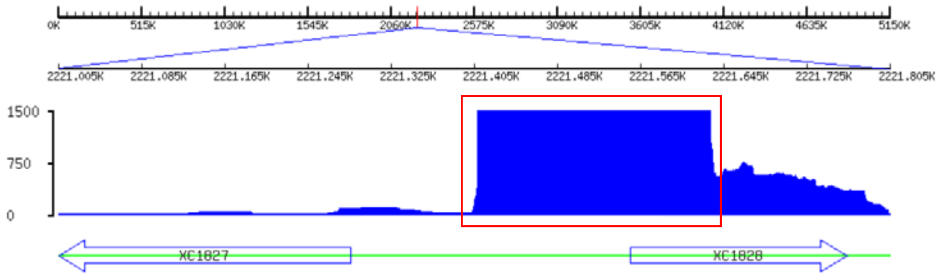

Transcript	Start	End	Length (bp)	RPKM	Inside gene	Overlap gene	Upstream gene	Downstream gene
TT263	2223778	2223838	61	58.9	XC1831	Null	XC1830	XC1832

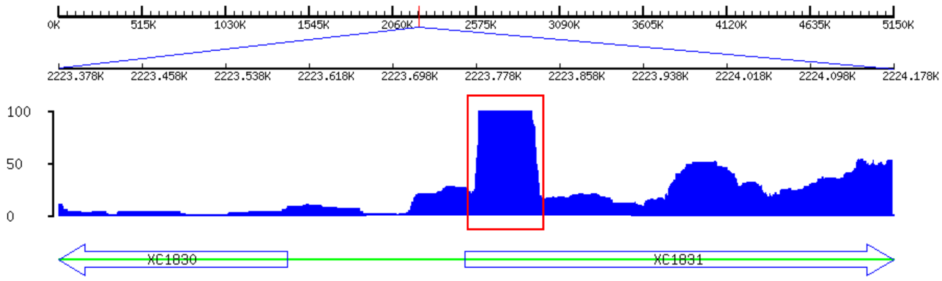

Transcript	Start	End	Length (bp)	RPKM	Inside gene	Overlap gene	Upstream gene	Downstream gene
TT264	2230280	2230330	50	9.2	XC1836	Null	XC1835	XC1837
TT265	2230540	2230624	85	272.1	Null	XC1836	XC1835	XC1837

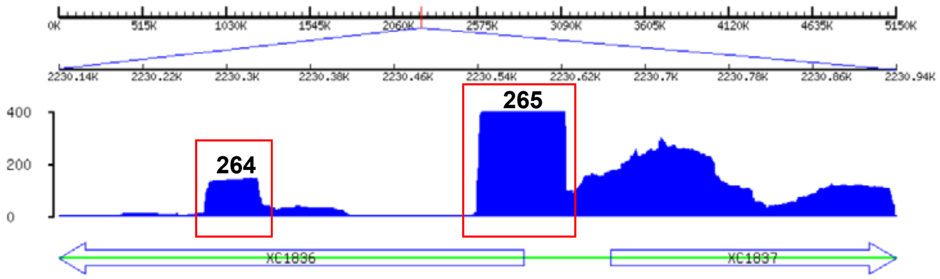

Transcript	Start	End	Length (bp)	RPKM	Inside gene	Overlap gene	Upstream gene	Downstream gene
TT266	2248862	2249165	304	79.8	XC1853	Null	XC1852	XC1854

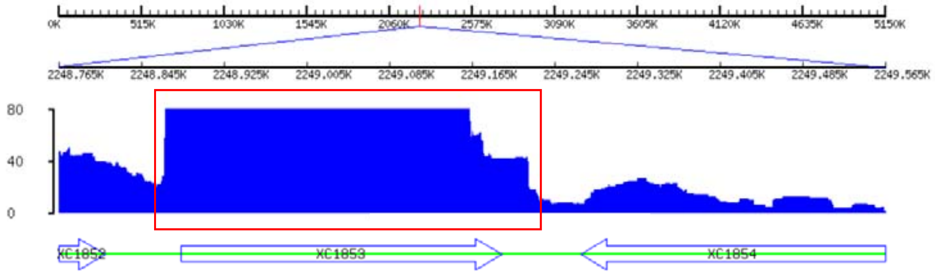

Transcript	Start	End	Length (bp)	RPKM	Inside gene	Overlap gene	Upstream gene	Downstream gene
TT267	2256215	2256330	116	72.1	Null	XC1860	XC1859	XC1861

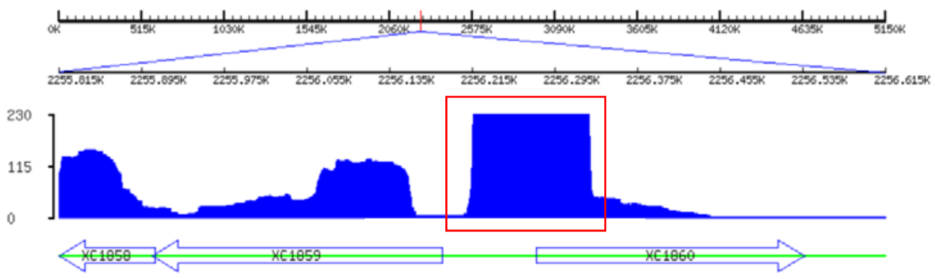

Transcript	Start	End	Length (bp)	RPKM	Inside gene	Overlap gene	Upstream gene	Downstream gene
TT268	2278165	2278223	58	9.2	XC1882	Null	XC1881	XC1883

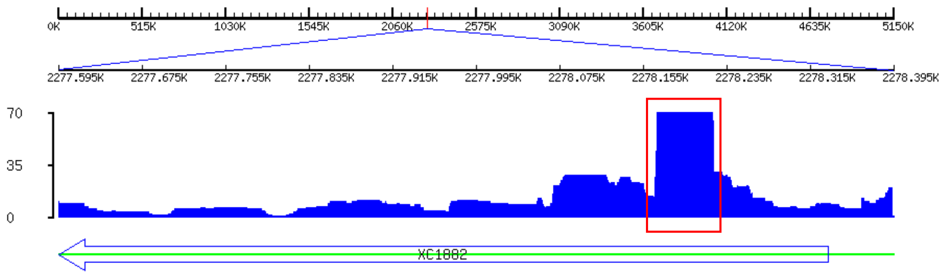

Transcript	Start	End	Length (bp)	RPKM	Inside gene	Overlap gene	Upstream gene	Downstream gene
TT269	2278395	2278480	86	356.7	XC4345	Null	XC1882	XC1883

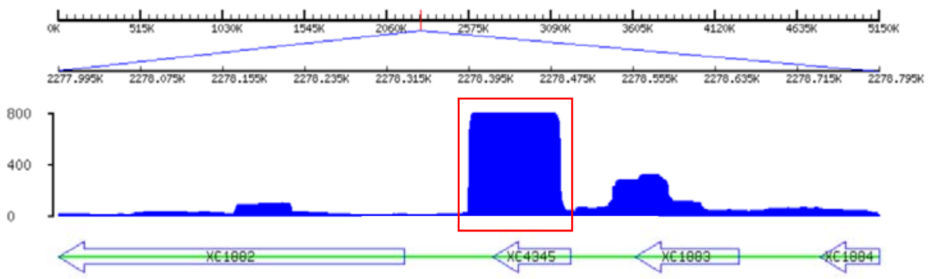

Transcript	Start	End	Length (bp)	RPKM	Inside gene	Overlap gene	Upstream gene	Downstream gene
TT270	2281622	2281711	90	82.2	Null	XC1886	XC1885	XC1887

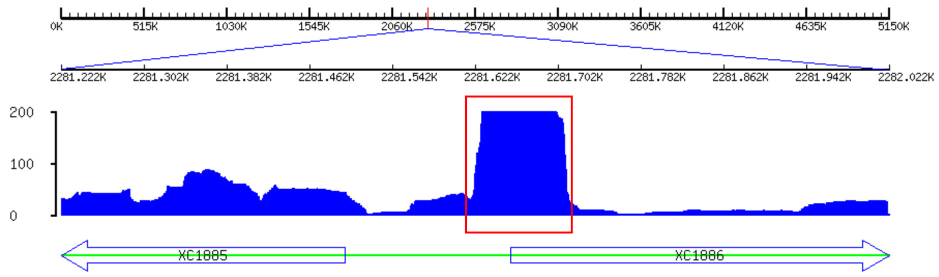

Transcript	Start	End	Length (bp)	RPKM	Inside gene	Overlap gene	Upstream gene	Downstream gene
TT271	2292655	2292725	70	36.2	Null	XC1896	XC1895	XC1897
TT272	2292876	2292945	70	43.6	XC1897	Null	XC1896	XC1898

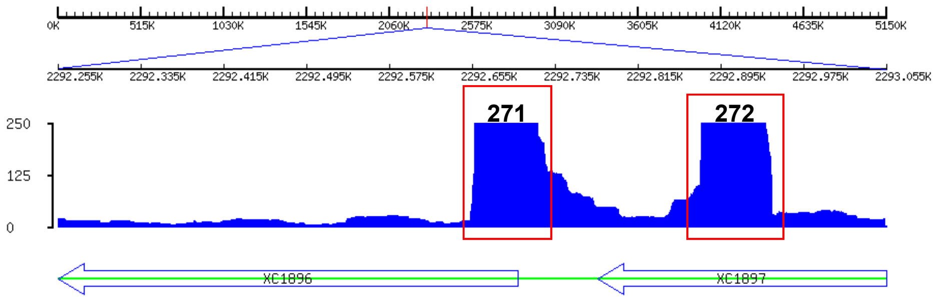

Transcript	Start	End	Length (bp)	RPKM	Inside gene	Overlap gene	Upstream gene	Downstream gene
TT273	2298646	2298706	61	348.8	Null	Null	XC1903	XC1904

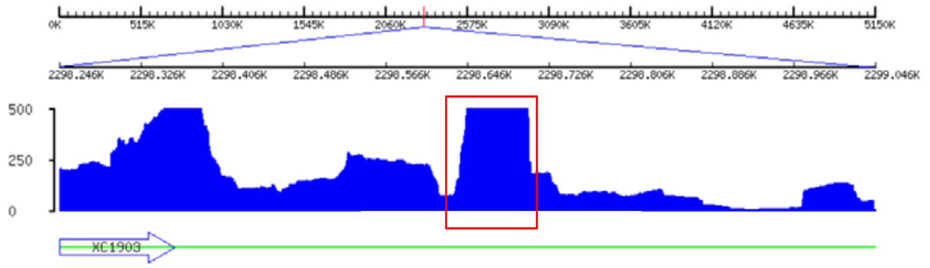

Transcript	Start	End	Length (bp)	RPKM	Inside gene	Overlap gene	Upstream gene	Downstream gene
TT274	2303046	2303141	96	240.9	Null	Null	XC1906	XC1907

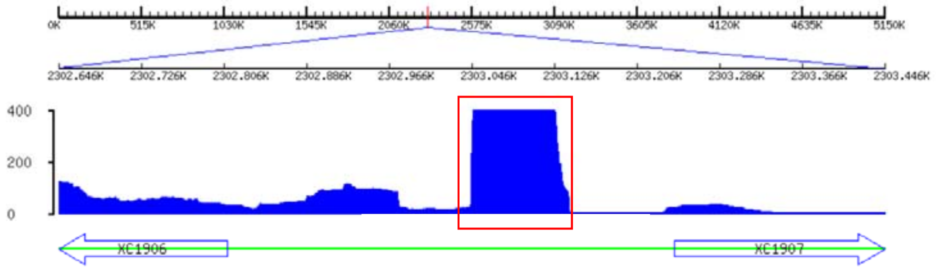

Transcript	Start	End	Length (bp)	RPKM	Inside gene	Overlap gene	Upstream gene	Downstream gene
TT275	2334041	2334111	71	412.3	Null	XC1934	XC1933	XC1935
TT276	2334327	2334413	87	48.7	XC1934	Null	XC1933	XC1935

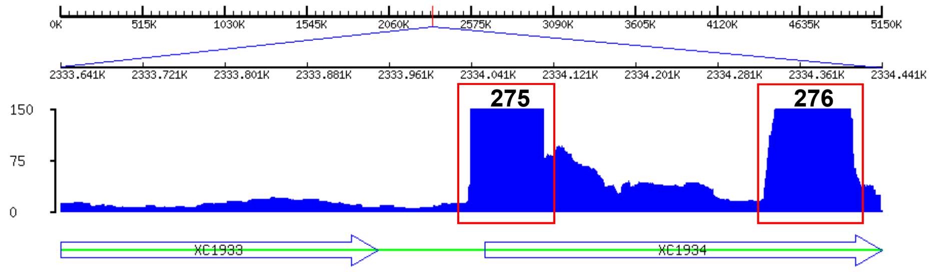

Transcript	Start	End	Length (bp)	RPKM	Inside gene	Overlap gene	Upstream gene	Downstream gene
TT277	2358598	2358674	72	915.7	XC1953	Null	XC1952	XC1954

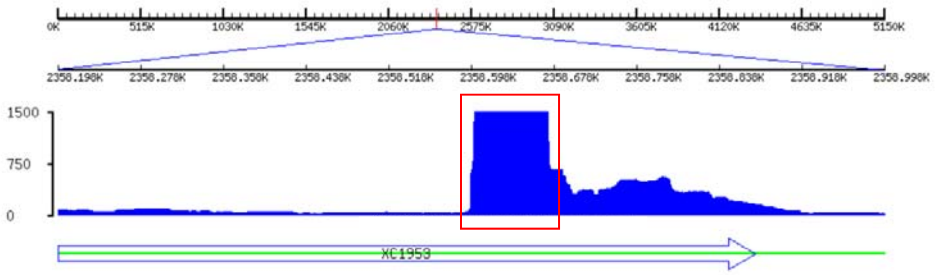

Transcript	Start	End	Length (bp)	RPKM	Inside gene	Overlap gene	Upstream gene	Downstream gene
TT278	2359088	2359178	91	89.2	XC1954	Null	XC1953	XC1955

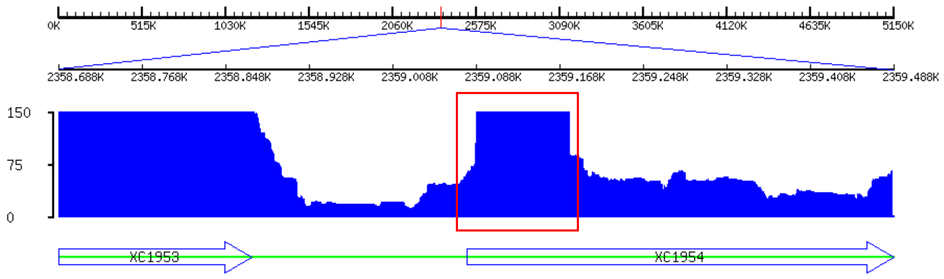

Transcript	Start	End	Length (bp)	RPKM	Inside gene	Overlap gene	Upstream gene	Downstream gene
TT279	2372820	2372917	97	70.2	Null	XC1966	XC1965	XC4346

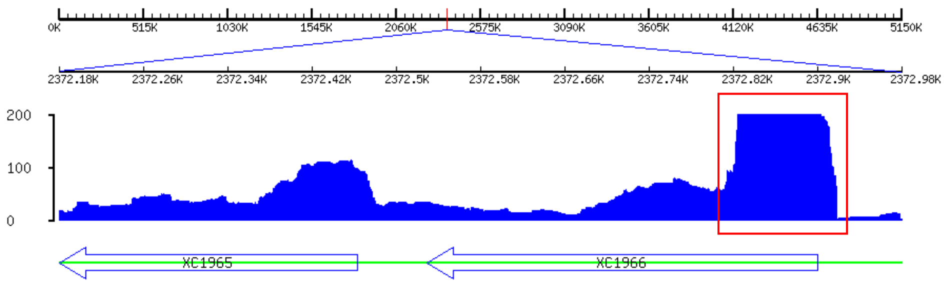

Transcript	Start	End	Length (bp)	RPKM	Inside gene	Overlap gene	Upstream gene	Downstream gene
TT280	2373053	2373118	65	11464.2	XC4346	Null	XC1966	XC1967

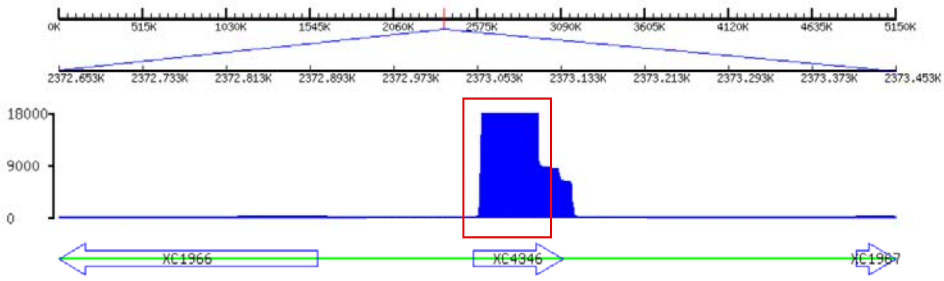

Transcript	Start	End	Length (bp)	RPKM	Inside gene	Overlap gene	Upstream gene	Downstream gene
TT281	2373869	2374083	214	90.1	Null	XC4347 /XC4348	XC1967	XC4349

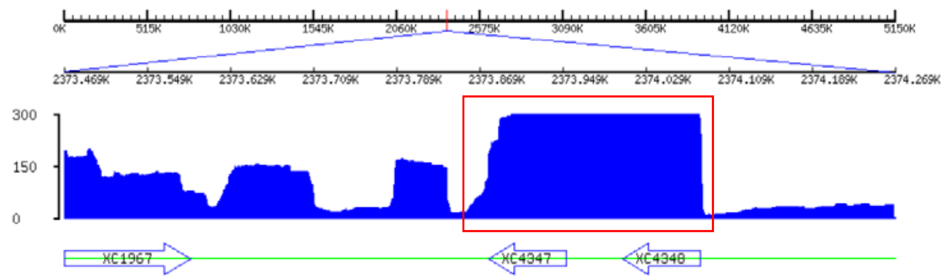

Transcript	Start	End	Length (bp)	RPKM	Inside gene	Overlap gene	Upstream gene	Downstream gene
TT282	2374836	2375033	197	398.1	XC4349 /XC4350	Null	XC4348	XC1968

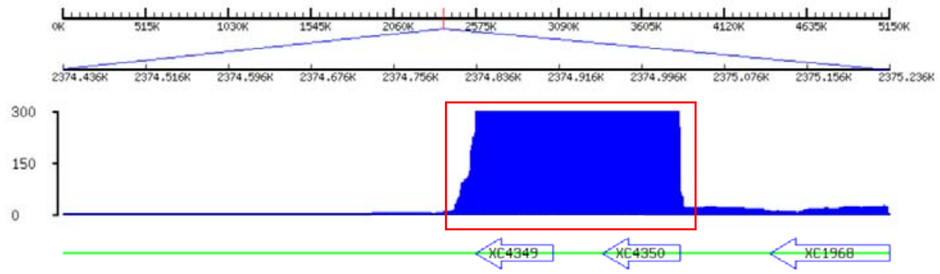

Transcript	Start	End	Length (bp)	RPKM	Inside gene	Overlap gene	Upstream gene	Downstream gene
TT283	2414123	2414194	71	452.2	XC4351	Null	XC2001	XC4352
TT284	2414283	2414352	70	35981.3	XC4352	Null	XC4351	XC4353
TT285	2414412	2414492	81	1081.9	XC4353	Null	XC4352	XC2002

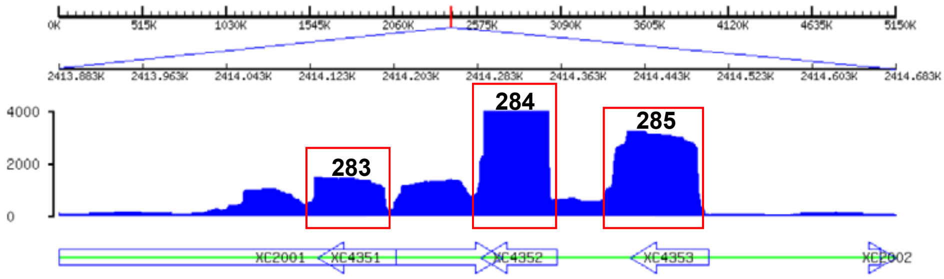

Transcript	Start	End	Length (bp)	RPKM	Inside gene	Overlap gene	Upstream gene	Downstream gene
TT286	2418445	2418513	69	719.7	XC2004	Null	XC2003	XC2005

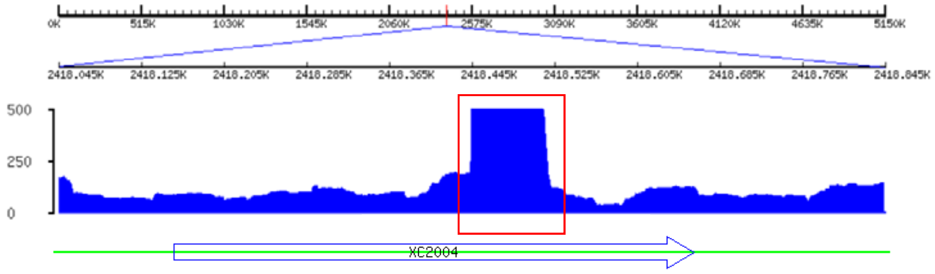

Transcript	Start	End	Length (bp)	RPKM	Inside gene	Overlap gene	Upstream gene	Downstream gene
TT287	2426223	2426278	56	57.9	Null	Null	XC2012	XC2013

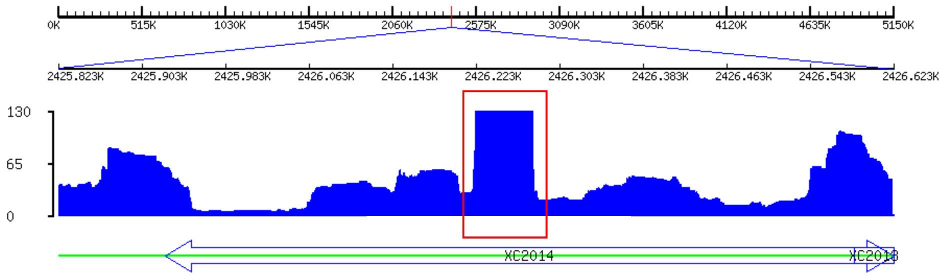

Transcript	Start	End	Length (bp)	RPKM	Inside gene	Overlap gene	Upstream gene	Downstream gene
TT288	2428848	2428904	57	389.2	Null	Null	XC2014	XC2015

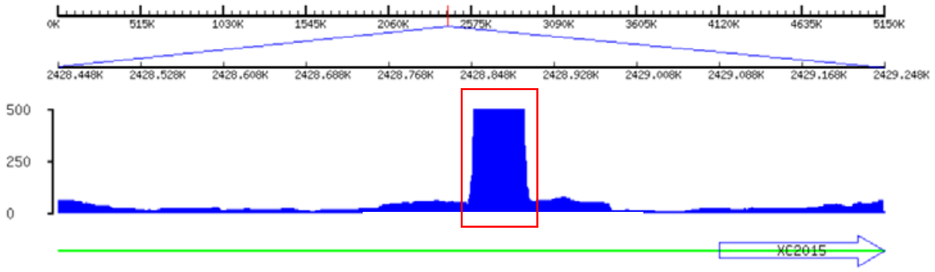

Transcript	Start	End	Length (bp)	RPKM	Inside gene	Overlap gene	Upstream gene	Downstream gene
TT289	2431128	2431185	58	105.8	Null	Null	XC2016	XC2017

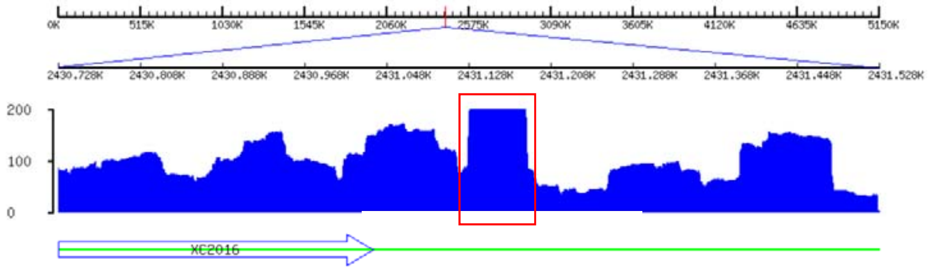

Transcript	Start	End	Length (bp)	RPKM	Inside gene	Overlap gene	Upstream gene	Downstream gene
TT290	2438148	2438207	60	87.9	XC2022	Null	XC2021	XC2023

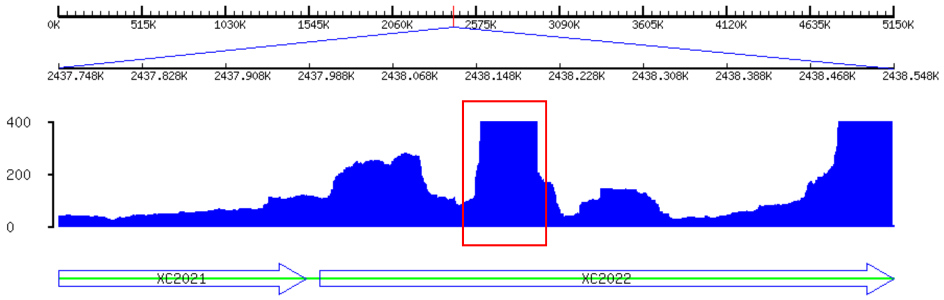

Transcript	Start	End	Length (bp)	RPKM	Inside gene	Overlap gene	Upstream gene	Downstream gene
TT291	2438490	2438555	65	99.8	XC2022	Null	XC2021	XC2023
TT292	2438665	2438733	69	275.8	Null	XC2023	XC2022	XC2024

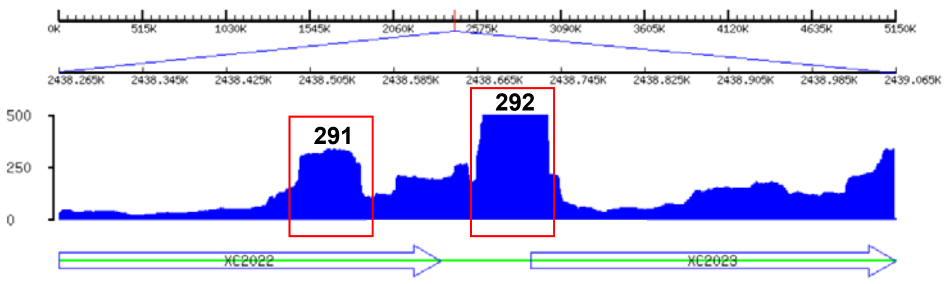

Transcript	Start	End	Length (bp)	RPKM	Inside gene	Overlap gene	Upstream gene	Downstream gene
TT293	2443942	2444096	154	95.3	XC2029	Null	XC2028	XC2030

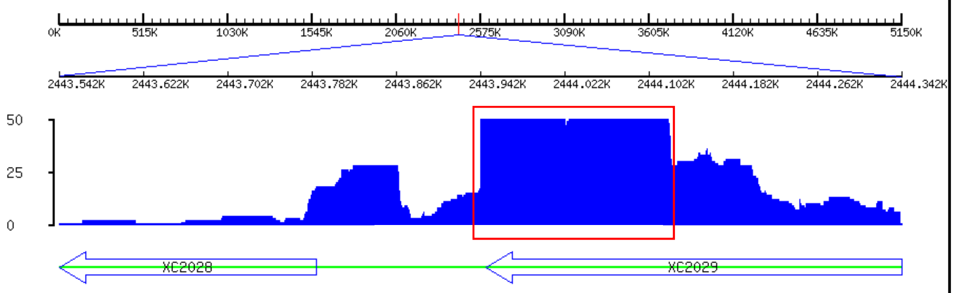

Transcript	Start	End	Length (bp)	RPKM	Inside gene	Overlap gene	Upstream gene	Downstream gene
TT294	2455991	2456046	56	114.9	XC2040	Null	XC2039	XC2041

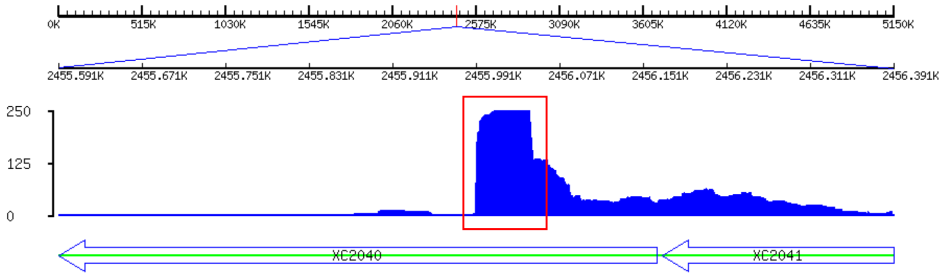

Transcript	Start	End	Length (bp)	RPKM	Inside gene	Overlap gene	Upstream gene	Downstream gene
TT295	2458026	2458115	90	85.7	Null	XC2044	XC2043	XC2045

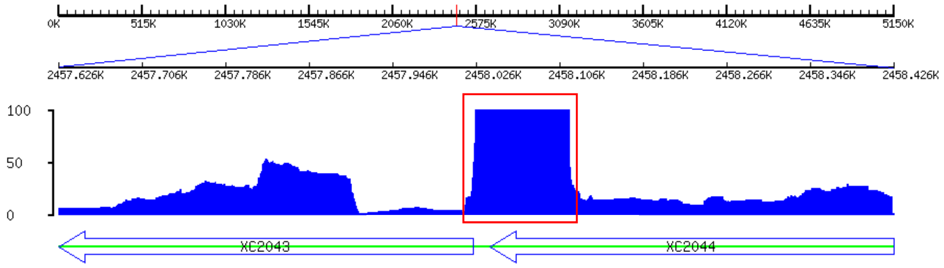

Transcript	Start	End	Length (bp)	RPKM	Inside gene	Overlap gene	Upstream gene	Downstream gene
TT296	2509851	2509912	62	89.1	XC2087	Null	XC2086	XC2088

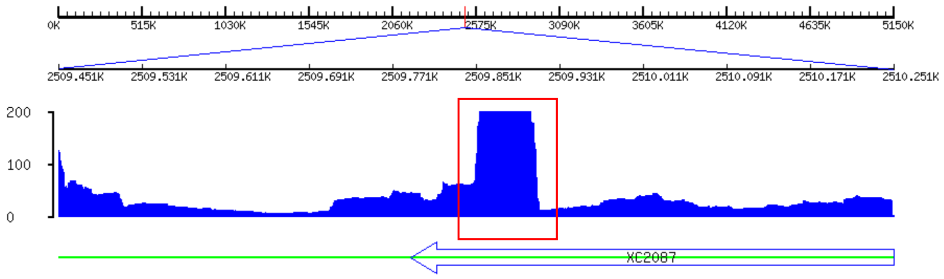

Transcript	Start	End	Length (bp)	RPKM	Inside gene	Overlap gene	Upstream gene	Downstream gene
TT297	2513401	2513472	72	411.7	Null	Null	XC2089	XC2090

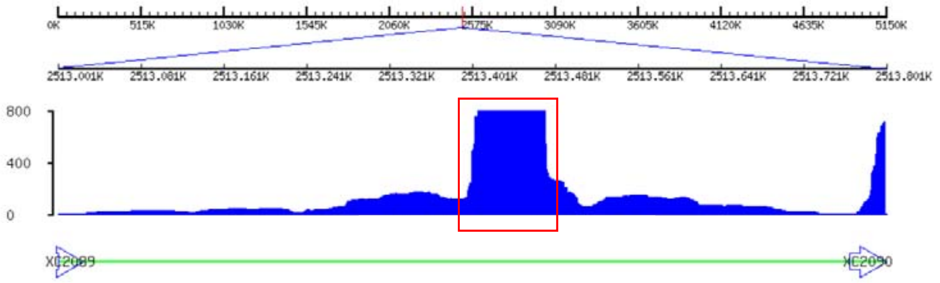

Transcript	Start	End	Length (bp)	RPKM	Inside gene	Overlap gene	Upstream gene	Downstream gene
TT298	2513787	2513873	87	164.7	XC2090	ULL	XC2089	XC4354

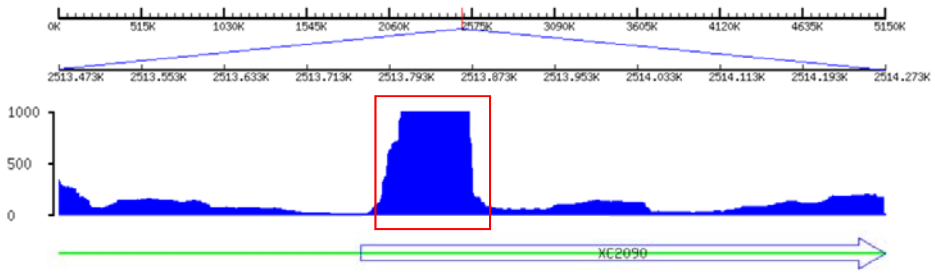

Transcript	Start	End	Length (bp)	RPKM	Inside gene	Overlap gene	Upstream gene	Downstream gene
TT299	2515196	2515268	72	7742.2	XC4354	Null	XC2089	XC2090

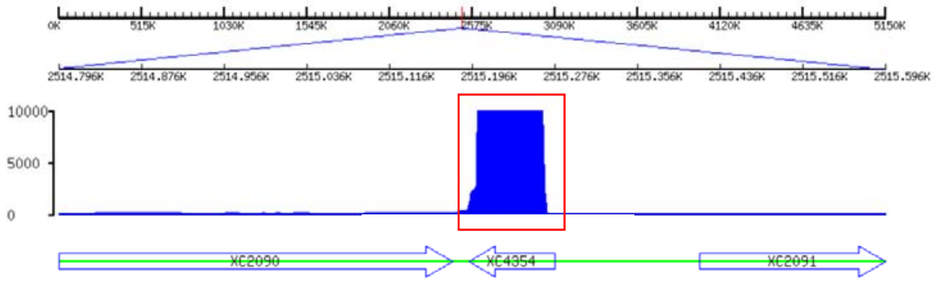

Transcript	Start	End	Length (bp)	RPKM	Inside gene	Overlap gene	Upstream gene	Downstream gene
TT300	2524765	2524854	90	471.1	Null	Null	XC2098	XC2099
TT301	2524870	2524968	98	1151.5	Null	Null	XC2098	XC2099

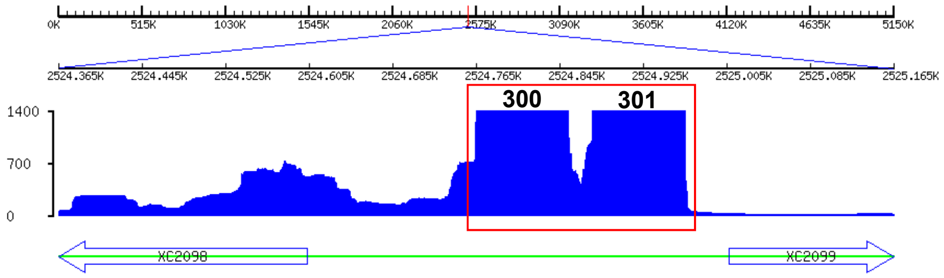

Transcript	Start	End	Length (bp)	RPKM	Inside gene	Overlap gene	Upstream gene	Downstream gene
TT302	2528080	2528154	75	69.3	Null	XC2102	XC2101	XC2103

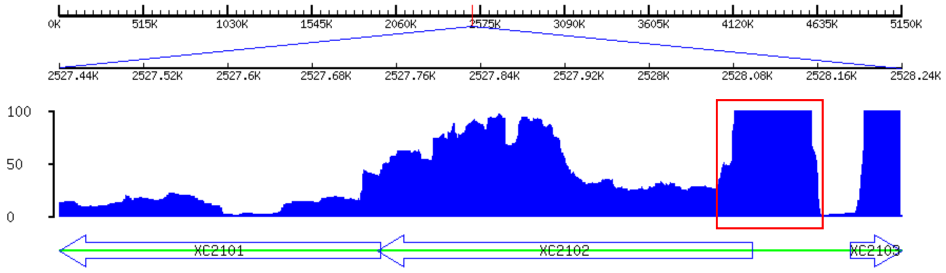

Transcript	Start	End	Length (bp)	RPKM	Inside gene	Overlap gene	Upstream gene	Downstream gene
TT303	2551037	2551103	67	44.3	XC2128	Null	XC2127	XC2129

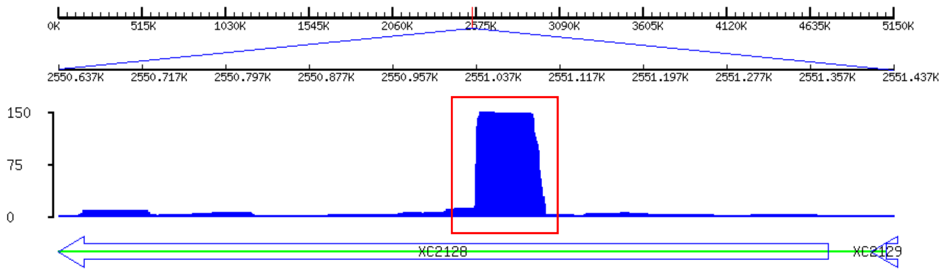

Transcript	Start	End	Length (bp)	RPKM	Inside gene	Overlap gene	Upstream gene	Downstream gene
TT304	2566429	2566739	311	93.3	Null	Null	XC2138	XC2139

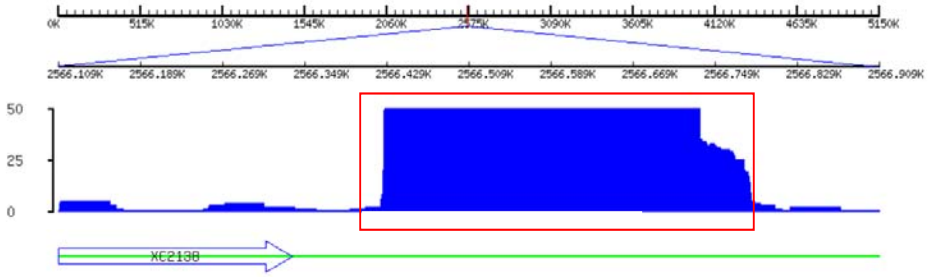

Transcript	Start	End	Length (bp)	RPKM	Inside gene	Overlap gene	Upstream gene	Downstream gene
TT305	2604812	2604903	92	50.4	Null	Null	XC2160	XC2161

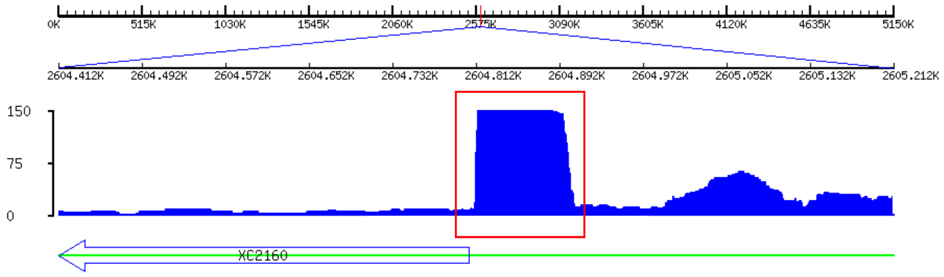

Transcript	Start	End	Length (bp)	RPKM	Inside gene	Overlap gene	Upstream gene	Downstream gene
TT306	2605473	2605544	72	112.4	XC2161	Null	XC2160	XC2162

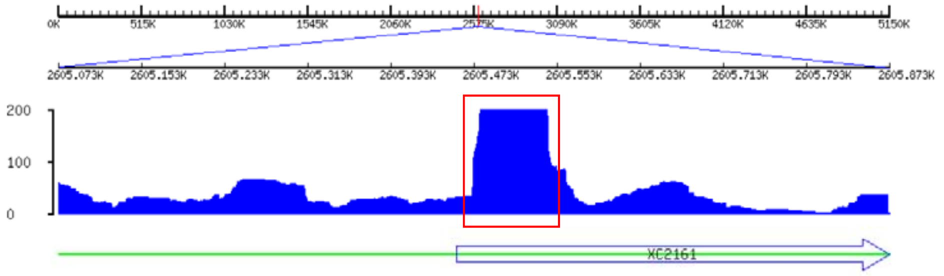

Transcript	Start	End	Length (bp)	RPKM	Inside gene	Overlap gene	Upstream gene	Downstream gene
TT307	2609805	2609908	104	279.9	Null	XC2164	XC2163	XC2165

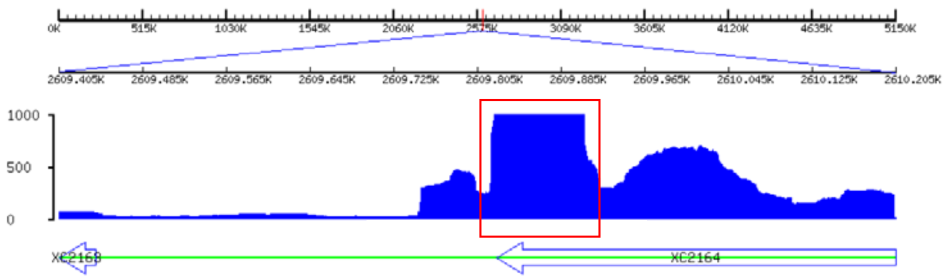

Transcript	Start	End	Length (bp)	RPKM	Inside gene	Overlap gene	Upstream gene	Downstream gene
TT308	2611091	2611380	290	390.1	XC2165	Null	XC2164	XC2166

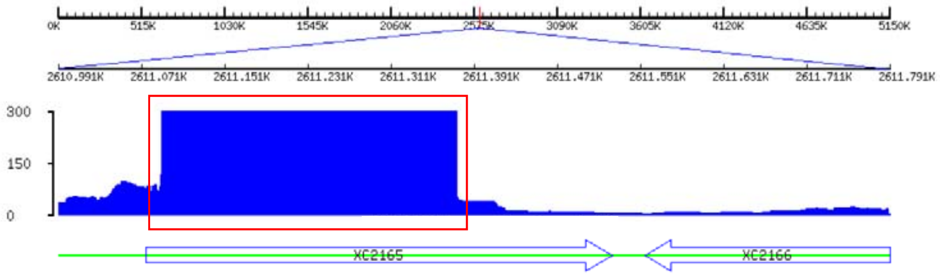

Transcript	Start	End	Length (bp)	RPKM	Inside gene	Overlap gene	Upstream gene	Downstream gene
TT309	2613155	2613216	62	82.6	Null	Null	XC2167	XC2168

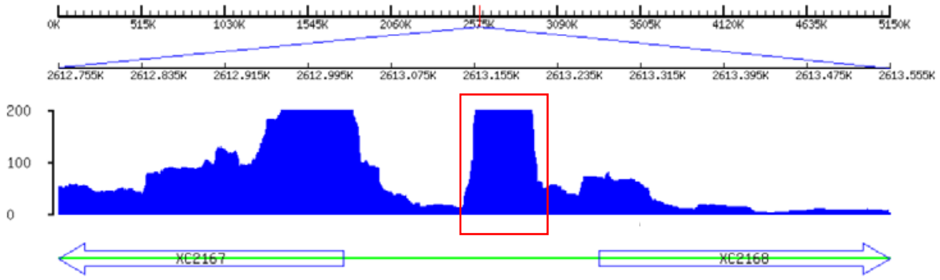

Transcript	Start	End	Length (bp)	RPKM	Inside gene	Overlap gene	Upstream gene	Downstream gene
TT310	2632909	2633006	97	109.1	XC2186	Null	XC2185	XC2187

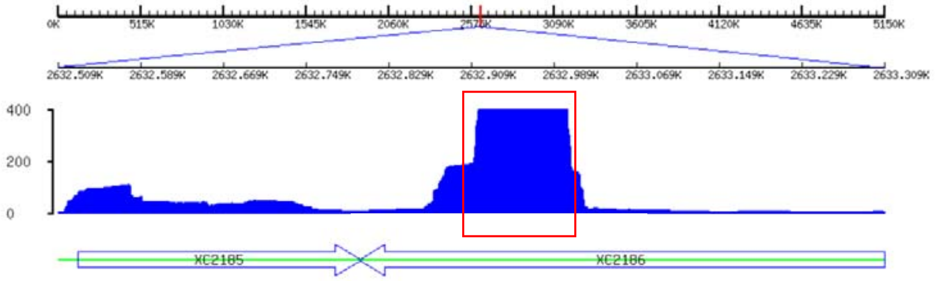

Transcript	Start	End	Length (bp)	RPKM	Inside gene	Overlap gene	Upstream gene	Downstream gene
TT311	2650241	2650294	54	89.3	XC2201	Null	XC2200	XC2202

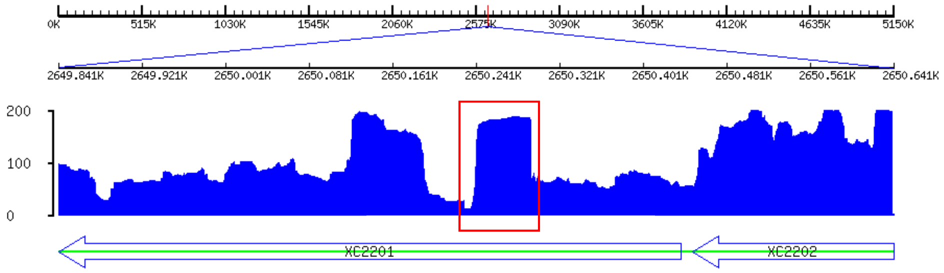

Transcript	Start	End	Length (bp)	RPKM	Inside gene	Overlap gene	Upstream gene	Downstream gene
TT312	2652059	2652116	58	286.5	Null	XC2203	XC2202	XC2204

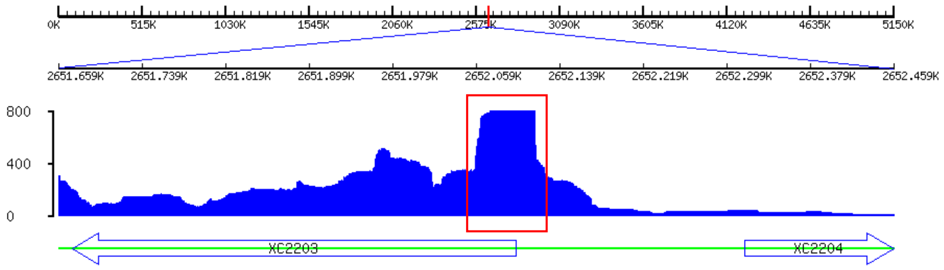

Transcript	Start	End	Length (bp)	RPKM	Inside gene	Overlap gene	Upstream gene	Downstream gene
TT313	2656886	2656937	108	444.9	Null	Null	XC2206	XC2208

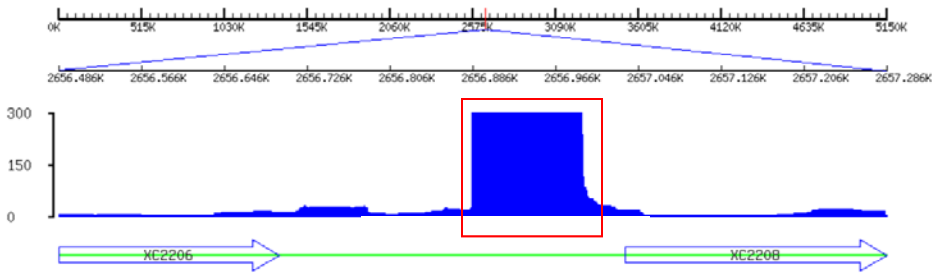

Transcript	Start	End	Length (bp)	RPKM	Inside gene	Overlap gene	Upstream gene	Downstream gene
TT314	2664818	2664928	110	33.9	Null	XC2211	XC2210	XC2212

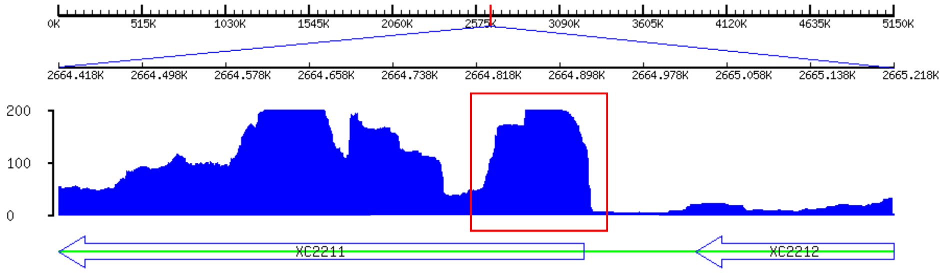

Transcript	Start	End	Length (bp)	RPKM	Inside gene	Overlap gene	Upstream gene	Downstream gene
TT315	2669660	2669738	79	109.2	Null	XC2214	XC2213	XC2215

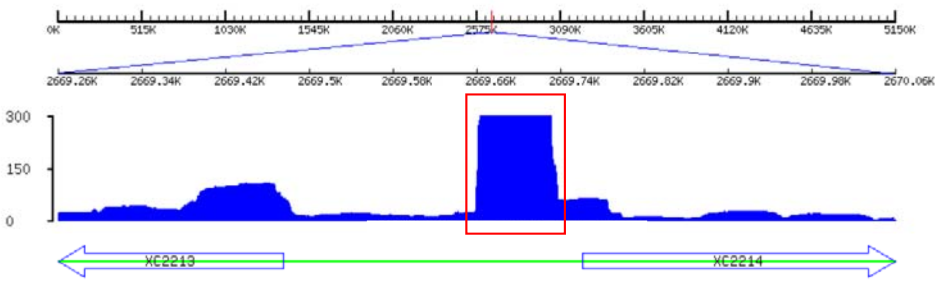

Transcript	Start	End	Length (bp)	RPKM	Inside gene	Overlap gene	Upstream gene	Downstream gene
TT316	2672746	2673016	270	138.6	Null	XC2217	XC2216	XC2218

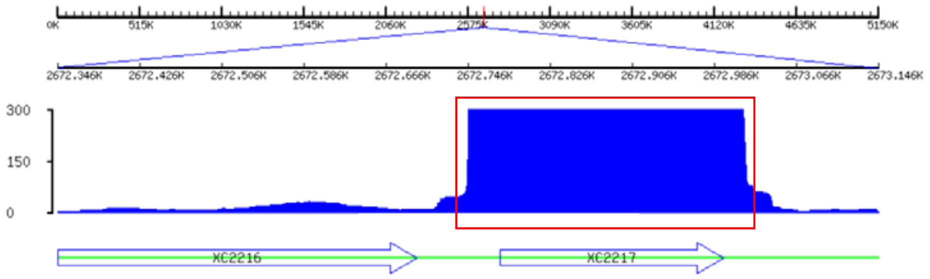

Transcript	Start	End	Length (bp)	RPKM	Inside gene	Overlap gene	Upstream gene	Downstream gene
TT317	2674533	2674591	59	157.6	XC2218	Null	XC2217	XC2219

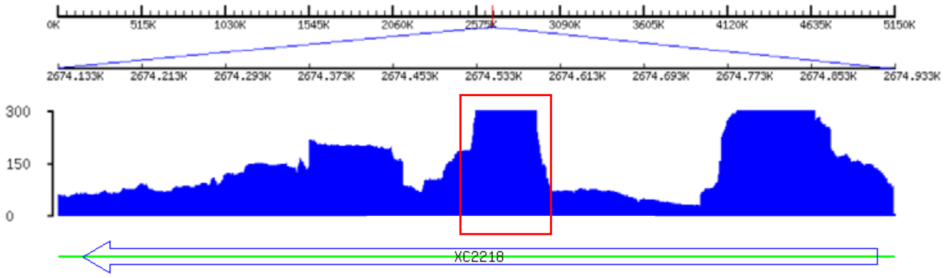

Transcript	Start	End	Length (bp)	RPKM	Inside gene	Overlap gene	Upstream gene	Downstream gene
TT318	2675156	2675268	113	154.6	XC2218	Null	XC2217	XC2219

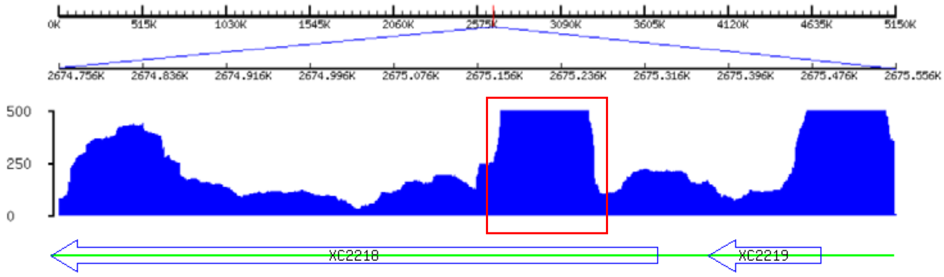

Transcript	Start	End	Length (bp)	RPKM	Inside gene	Overlap gene	Upstream gene	Downstream gene
TT319	2675595	2675684	90	686.3	XC2218	Null	XC2217	XC2219

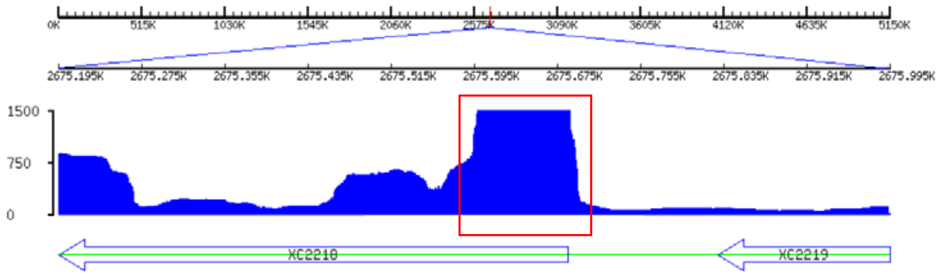

Transcript	Start	End	Length (bp)	RPKM	Inside gene	Overlap gene	Upstream gene	Downstream gene
TT320	2676130	2676194	65	118.7	Null	XC2219	XC2218	XC2220

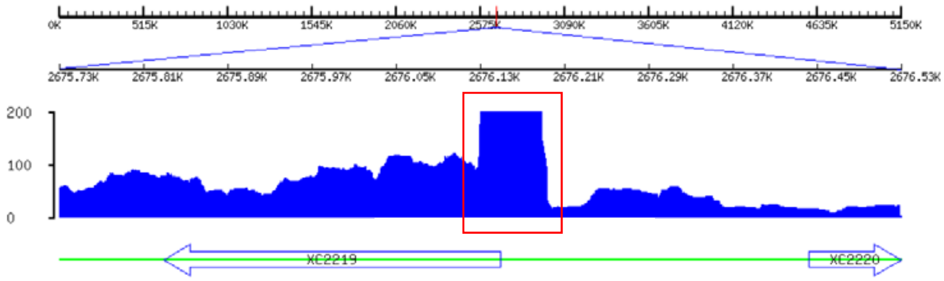

Transcript	Start	End	Length (bp)	RPKM	Inside gene	Overlap gene	Upstream gene	Downstream gene
TT321	2686474	2686558	85	143.8	Null	XC2227/XC2228	XC2226	XC2229

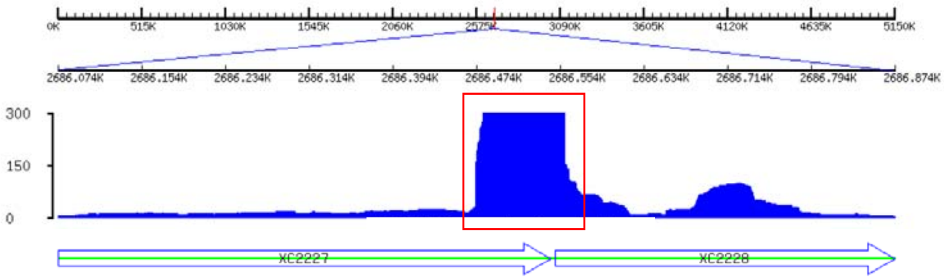

Transcript	Start	End	Length (bp)	RPKM	Inside gene	Overlap gene	Upstream gene	Downstream gene
TT322	2687002	2687059	58	37.4	XC2228	Null	XC2227	XC2229

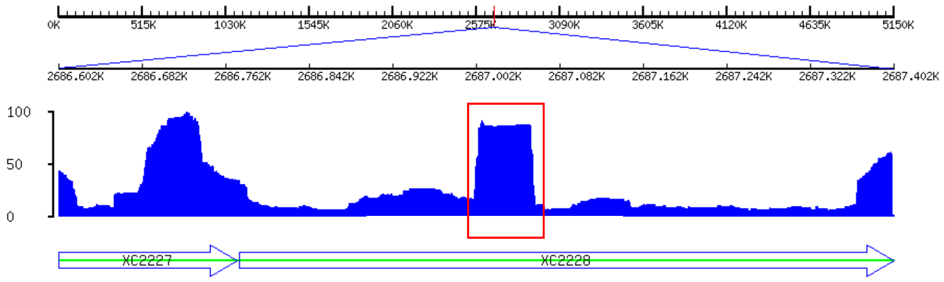

Transcript	Start	End	Length (bp)	RPKM	Inside gene	Overlap gene	Upstream gene	Downstream gene
TT323	2688285	2688345	61	133.6	Null	XC2228	XC2227	XC2229

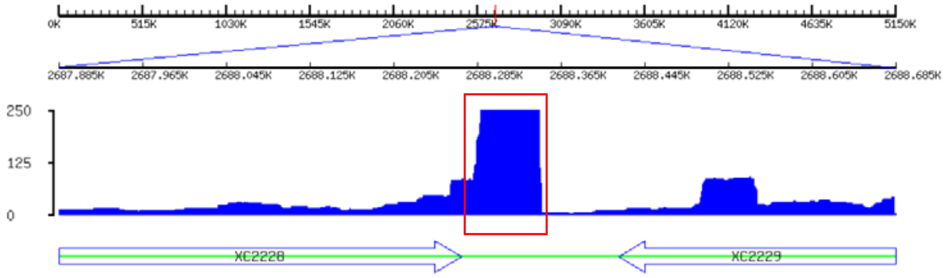

Transcript	Start	End	Length (bp)	RPKM	Inside gene	Overlap gene	Upstream gene	Downstream gene
TT324	2696762	2696842	81	437.1	Null	Null	XC2238	XC2239

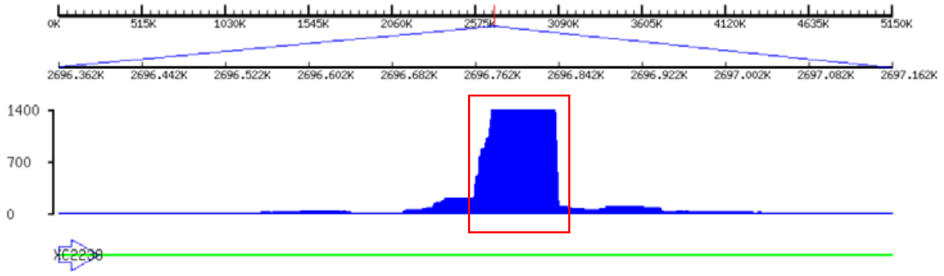

Transcript	Start	End	Length (bp)	RPKM	Inside gene	Overlap gene	Upstream gene	Downstream gene
TT325	2697318	2697449	132	37.7	Null	XC2239	XC2238	XC2240

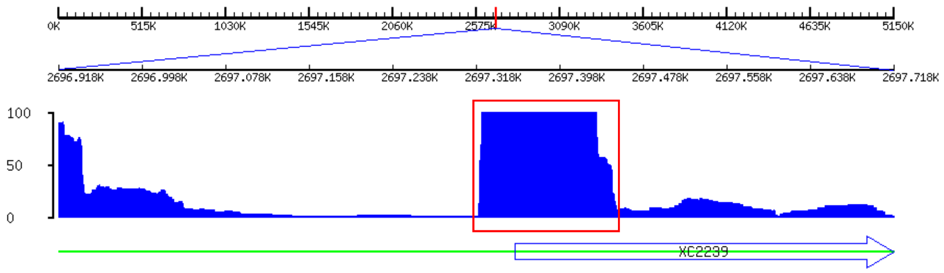

Transcript	Start	End	Length (bp)	RPKM	Inside gene	Overlap gene	Upstream gene	Downstream gene
TT326	2708703	2708768	66	346.8	XC2249	Null	XC2248	XC2250

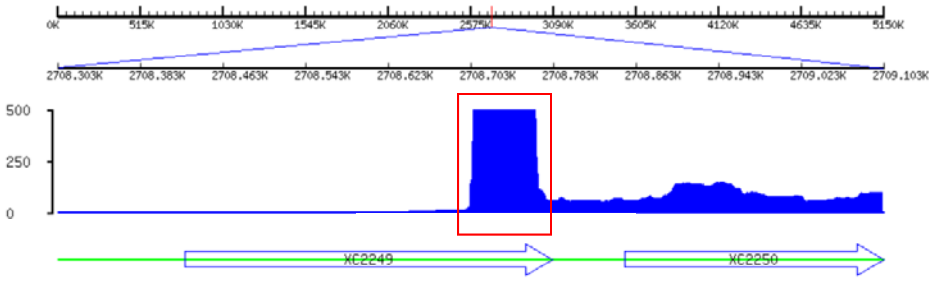

Transcript	Start	End	Length (bp)	RPKM	Inside gene	Overlap gene	Upstream gene	Downstream gene
TT327	2711028	2711082	55	122.8	XC2251	Null	XC2250	XC2252

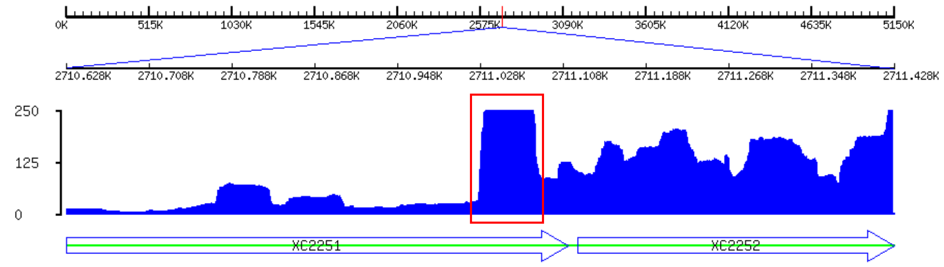

Transcript	Start	End	Length (bp)	RPKM	Inside gene	Overlap gene	Upstream gene	Downstream gene
TT328	2711448	2711512	64	795.6	2711448	2711512	64	795.6

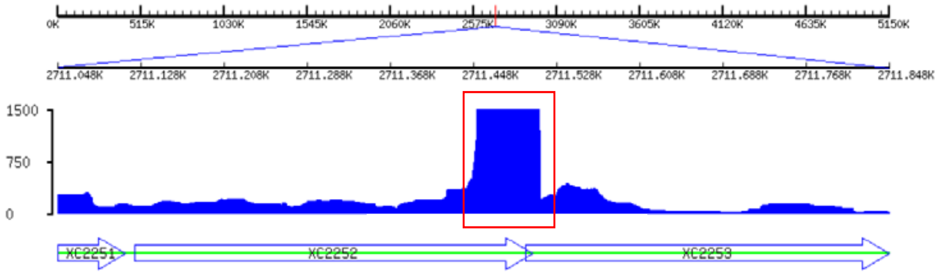

Transcript	Start	End	Length (bp)	RPKM	Inside gene	Overlap gene	Upstream gene	Downstream gene
TT329	2712357	2712419	63	95.3	XC2253	Null	XC2252	XC2254

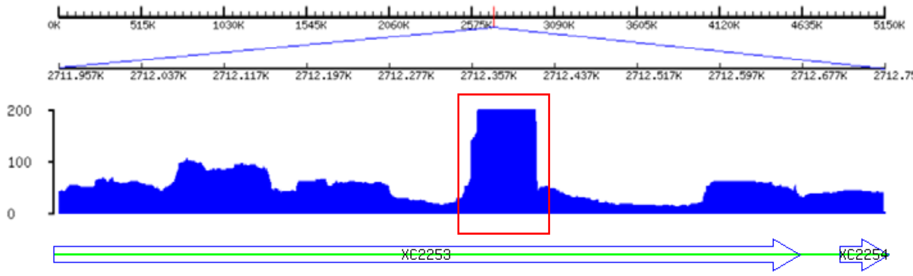

Transcript	Start	End	Length (bp)	RPKM	Inside gene	Overlap gene	Upstream gene	Downstream gene
TT330	2742223	2742276	54	47.1	Null	XC2277	XC2276	XC2278
TT331	2742505	2742577	72	21.6	XC2277	Null	XC2276	XC2278

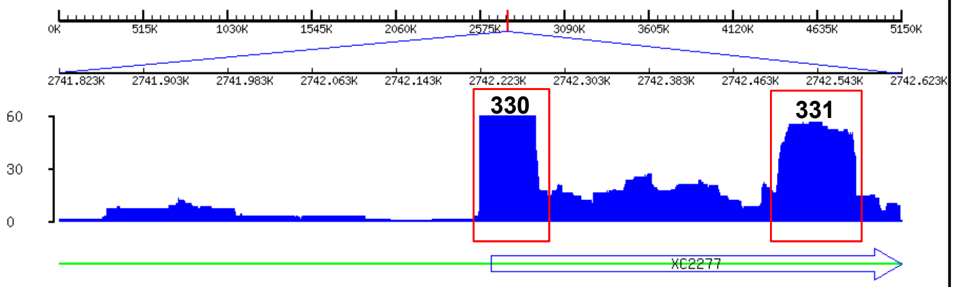

Transcript	Start	End	Length (bp)	RPKM	Inside gene	Overlap gene	Upstream gene	Downstream gene
TT332	2760980	2761064	84	395.3	Null	XC2293	XC2292	XC2294

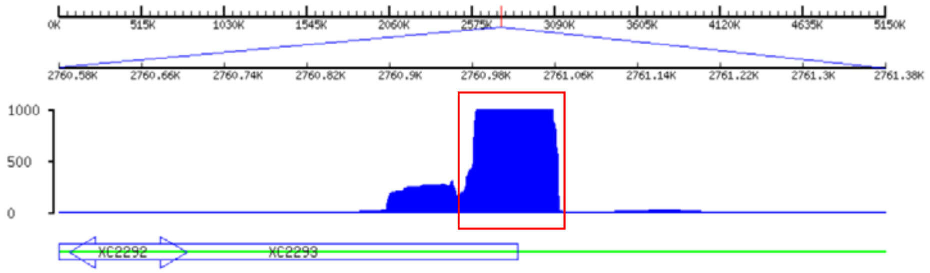

Transcript	Start	End	Length (bp)	RPKM	Inside gene	Overlap gene	Upstream gene	Downstream gene
TT333	2768652	2768730	79	34.9	Null	Null	XC2296	XC2297

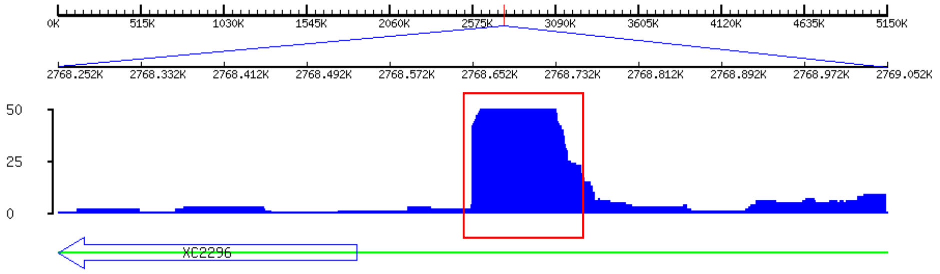

Transcript	Start	End	Length (bp)	RPKM	Inside gene	Overlap gene	Upstream gene	Downstream gene
TT334	2840642	2840754	113	35.5	Null	XC2348	XC2347	XC2349

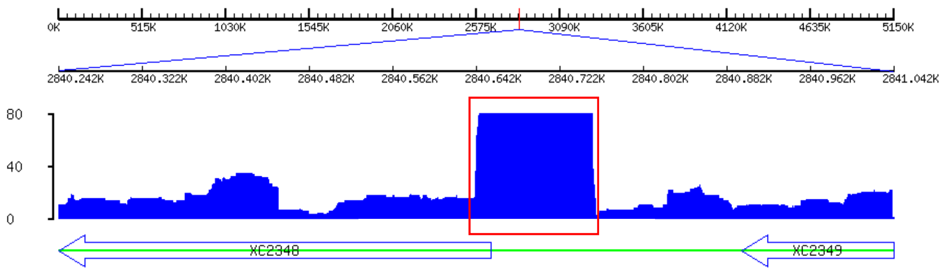

Transcript	Start	End	Length (bp)	RPKM	Inside gene	Overlap gene	Upstream gene	Downstream gene
TT335	2899441	2899500	60	3893.1	XC4355	Null	XC2396	XC2399

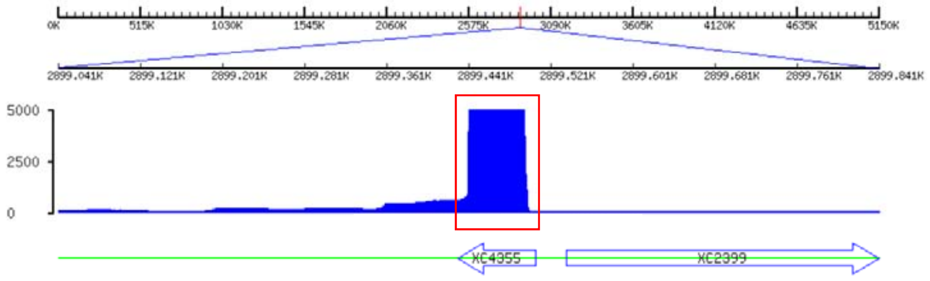

Transcript	Start	End	Length (bp)	RPKM	Inside gene	Overlap gene	Upstream gene	Downstream gene
TT336	2909838	2909913	76	64.2	Null	XC2403	XC2402	XC2404

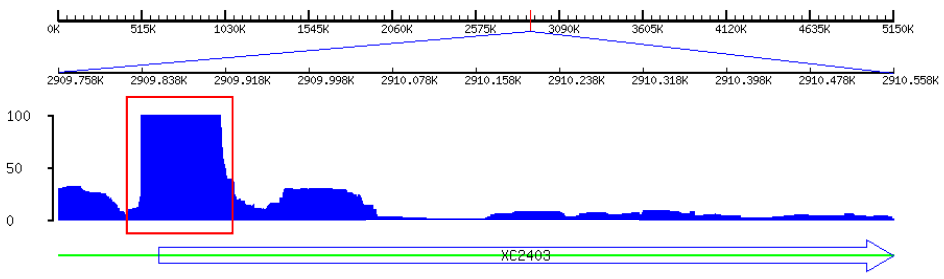

Transcript	Start	End	Length (bp)	RPKM	Inside gene	Overlap gene	Upstream gene	Downstream gene
TT337	2919083	2919303	220	244.1	Null	XC2410	XC2409	XC2411

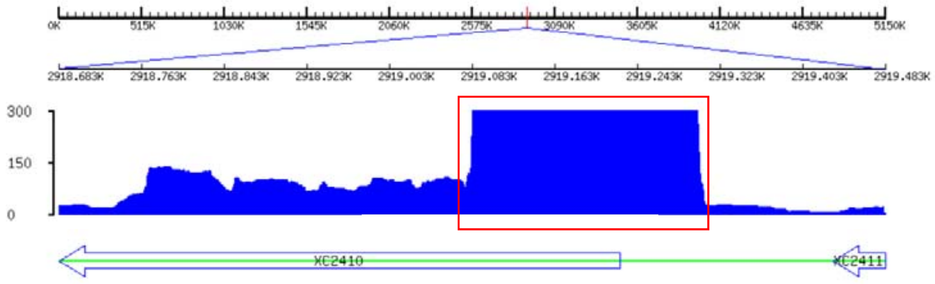

Transcript	Start	End	Length (bp)	RPKM	Inside gene	Overlap gene	Upstream gene	Downstream gene
TT338	2926371	2926572	201	255.2	Null	XC2414	XC2413	XC2415

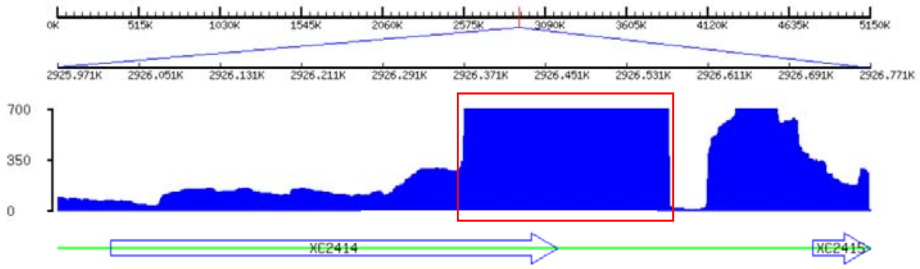

Transcript	Start	End	Length (bp)	RPKM	Inside gene	Overlap gene	Upstream gene	Downstream gene
TT339	2929115	2929355	240	657.2	XC2418	Null	XC2417	XC2419

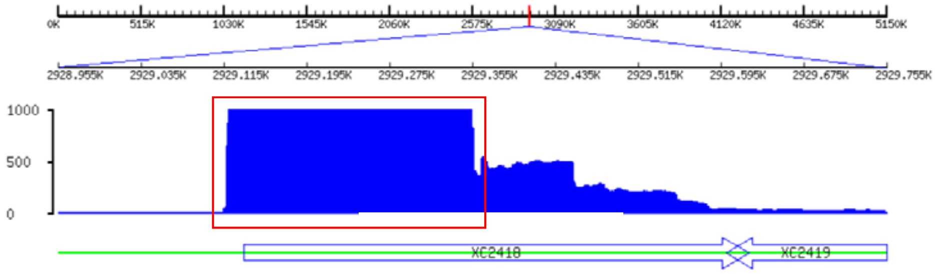

Transcript	Start	End	Length (bp)	RPKM	Inside gene	Overlap gene	Upstream gene	Downstream gene
TT340	2932972	2933220	248	196.1	Null	Null	XC2422	XC2423

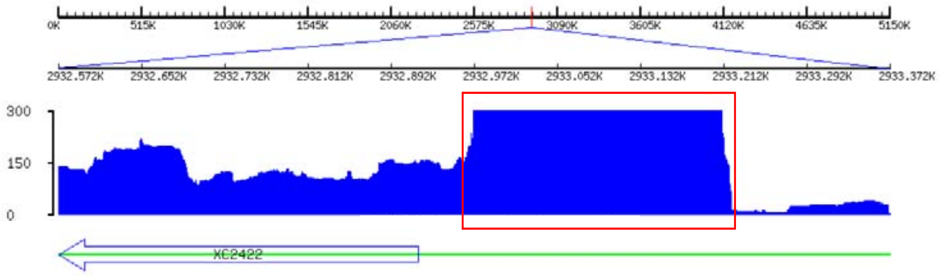

Transcript	Start	End	Length (bp)	RPKM	Inside gene	Overlap gene	Upstream gene	Downstream gene
TT341	2939567	2939752	185	8230	Null	XC2426	XC2425	XC2427

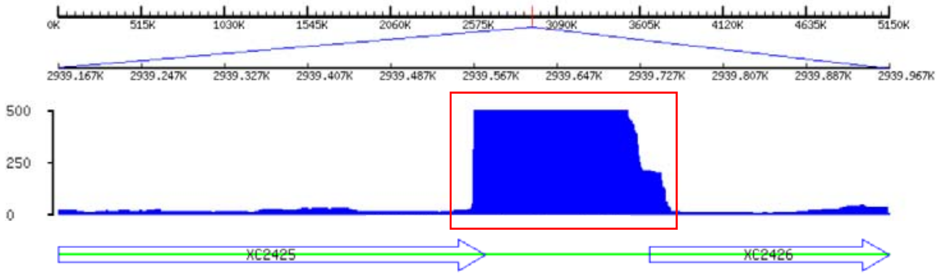

Transcript	Start	End	Length (bp)	RPKM	Inside gene	Overlap gene	Upstream gene	Downstream gene
TT342	2939567	2939638	71	2944.9	Null	XC2426	XC2425	XC2427

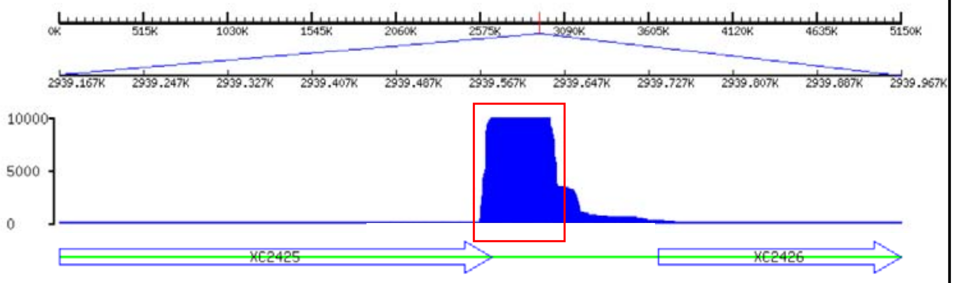

Transcript	Start	End	Length (bp)	RPKM	Inside gene	Overlap gene	Upstream gene	Downstream gene
TT343	2940875	2940934	60	151.9	Null	XC2426 /XC2427	XC2425	XC2428

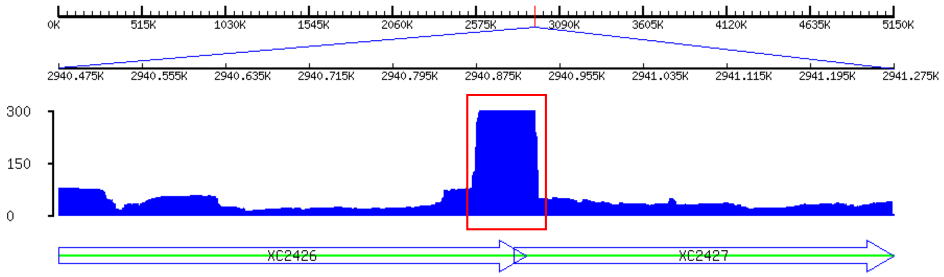

Transcript	Start	End	Length (bp)	RPKM	Inside gene	Overlap gene	Upstream gene	Downstream gene
TT344	2944151	2944205	55	924.9	Null	Null	XC2429	XC2430

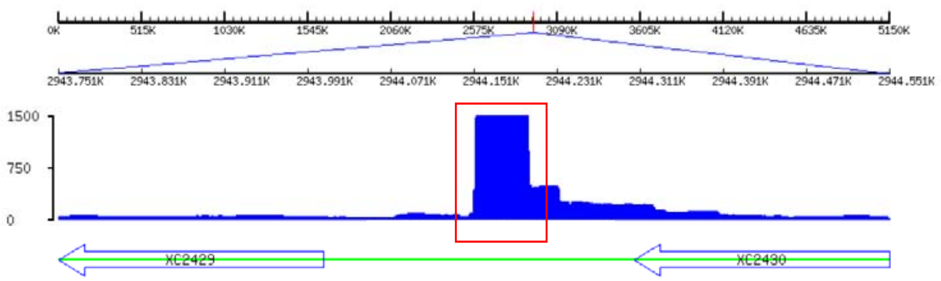

Transcript	Start	End	Length (bp)	RPKM	Inside gene	Overlap gene	Upstream gene	Downstream gene
TT345	2945183	2945246	64	76.6	Null	XC2430	XC2429	XC2431

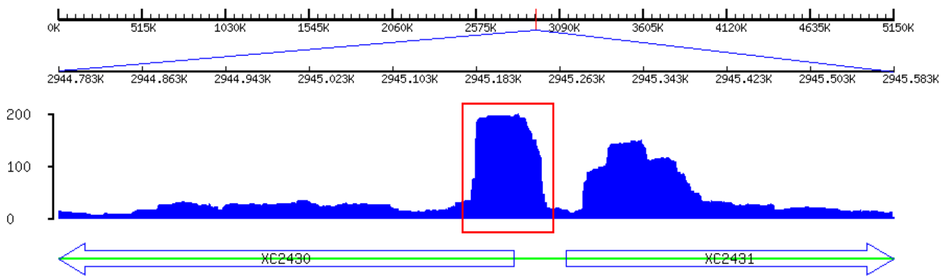

Transcript	Start	End	Length (bp)	RPKM	Inside gene	Overlap gene	Upstream gene	Downstream gene
TT346	2972679	2972743	65	230.1	Null	Null	XC2458	XC2459

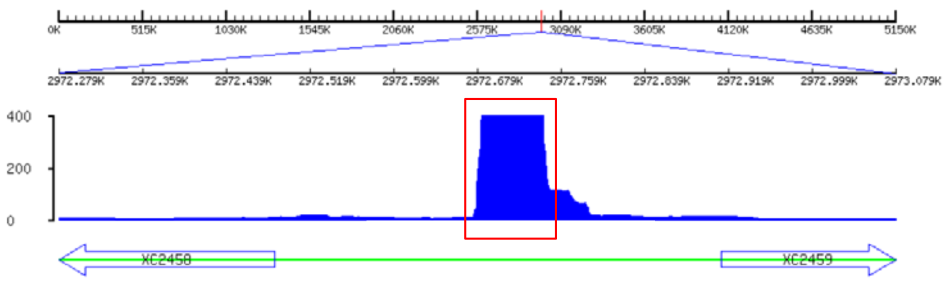

Transcript	Start	End	Length (bp)	RPKM	Inside gene	Overlap gene	Upstream gene	Downstream gene
TT347	2987874	2987928	55	190.1	XC2470	Null	XC2469	XC4356

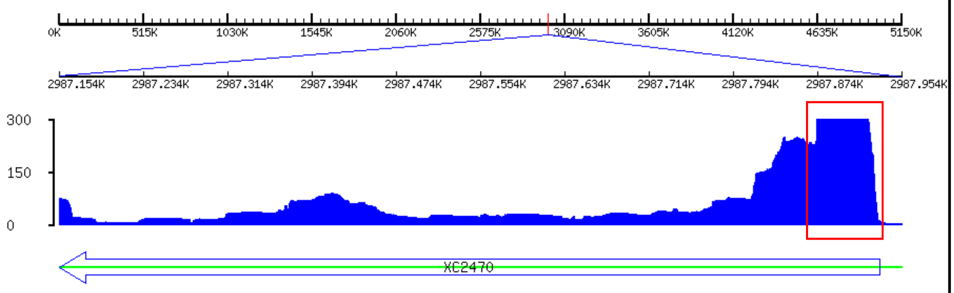

Transcript	Start	End	Length (bp)	RPKM	Inside gene	Overlap gene	Upstream gene	Downstream gene
TT348	2987988	2988049	61	2706.8	XC4356	Null	XC2469	XC2471

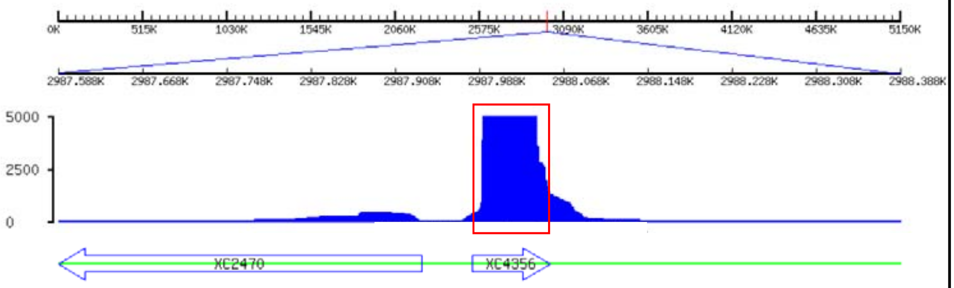

Transcript	Start	End	Length (bp)	RPKM	Inside gene	Overlap gene	Upstream gene	Downstream gene
TT349	3024007	3024079	73	51.1	Null	XC2495 /XC2496	XC2494	XC2497

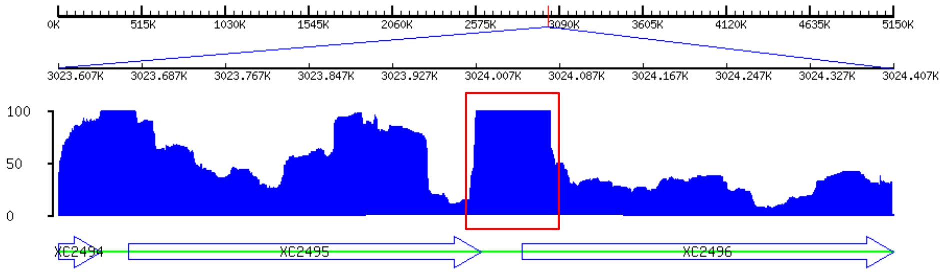

Transcript	Start	End	Length (bp)	RPKM	Inside gene	Overlap gene	Upstream gene	Downstream gene
TT350	3028347	3028440	93	752.9	Null	XC4358	XC4357	XC2501

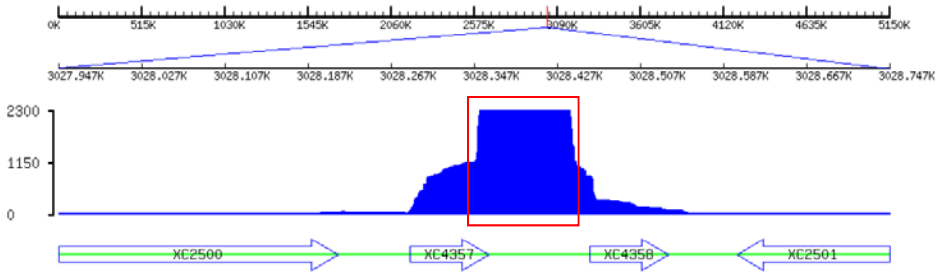

Transcript	Start	End	Length (bp)	RPKM	Inside gene	Overlap gene	Upstream gene	Downstream gene
TT351	3035112	3035198	86	1712.1	XC4359	Null	XC2505	XC2506

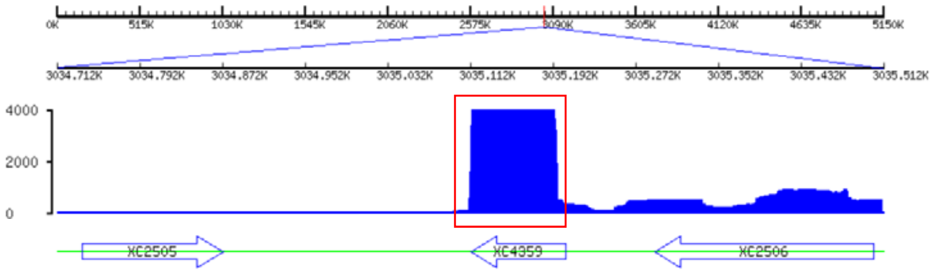

Transcript	Start	End	Length (bp)	RPKM	Inside gene	Overlap gene	Upstream gene	Downstream gene
TT352	3035652	3035743	92	556.7	XC2507	Null	XC2506	XC2508

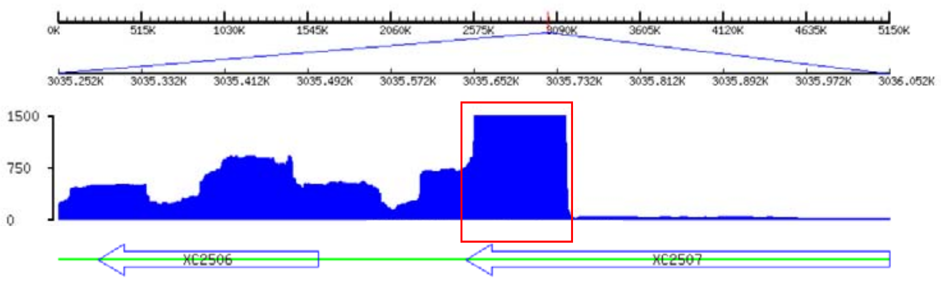

Transcript	Start	End	Length (bp)	RPKM	Inside gene	Overlap gene	Upstream gene	Downstream gene
TT353	3038601	3038661	61	84.4	XC2508	Null	XC2507	XC2509
TT354	3038922	3038984	63	709.1	Null	Null	XC2508	XC2509

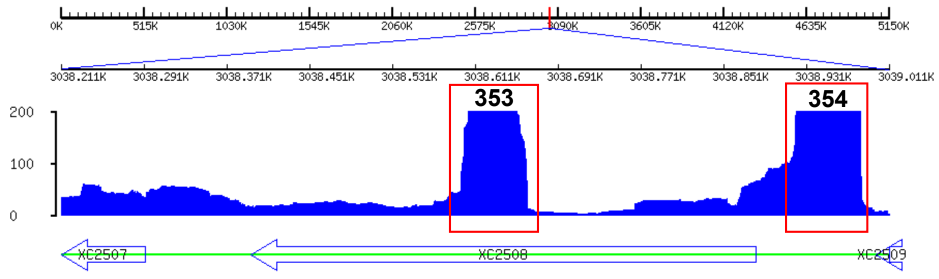

Transcript	Start	End	Length (bp)	RPKM	Inside gene	Overlap gene	Upstream gene	Downstream gene
TT355	3039995	3040047	53	133.5	Null	XC2509	XC2508	XC2510

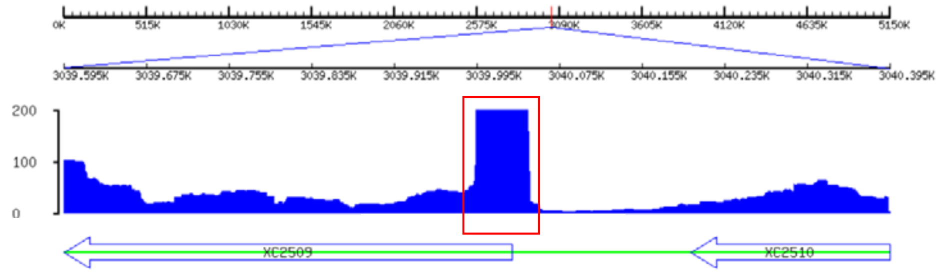

Transcript	Start	End	Length (bp)	RPKM	Inside gene	Overlap gene	Upstream gene	Downstream gene
TT356	3048398	3048450	53	232.1	XC2515	Null	XC2514	XC2516

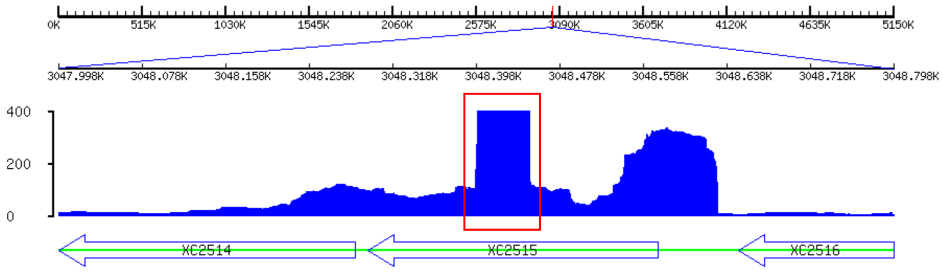

Transcript	Start	End	Length (bp)	RPKM	Inside gene	Overlap gene	Upstream gene	Downstream gene
TT357	3050859	3050915	57	183.4	XC2518	Null	XC2517	XC2519

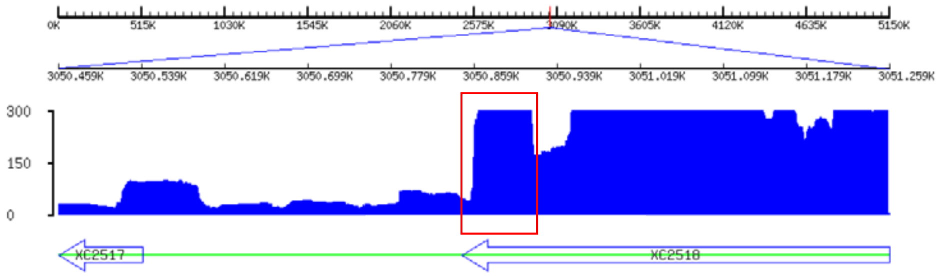

Transcript	Start	End	Length (bp)	RPKM	Inside gene	Overlap gene	Upstream gene	Downstream gene
TT358	3053031	3053085	55	305.5	XC2519	Null	XC2518	XC2520

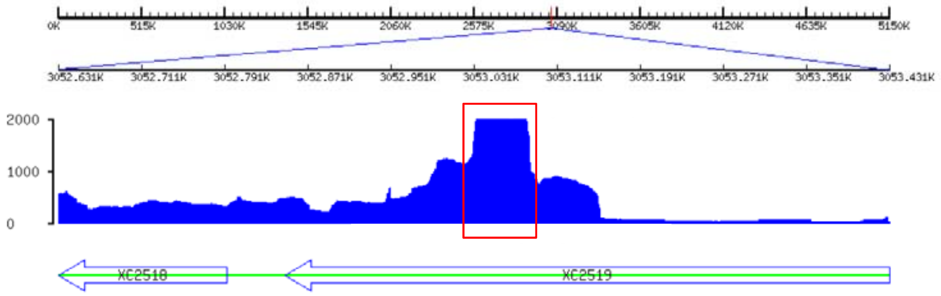

Transcript	Start	End	Length (bp)	RPKM	Inside gene	Overlap gene	Upstream gene	Downstream gene
TT359	3054244	3054427	184	39.6	Null	Null	XC2521	XC2522

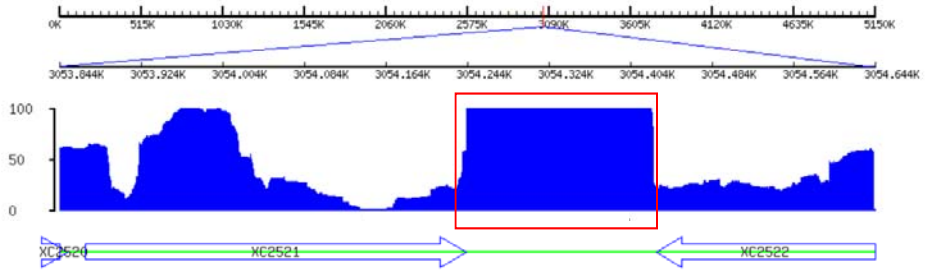

Transcript	Start	End	Length (bp)	RPKM	Inside gene	Overlap gene	Upstream gene	Downstream gene
TT360	3063812	3063867	56	96.2	Null	Null	XC2533	XC2534
TT361	3063918	3063995	78	110.6	Null	Null	XC2533	XC2534

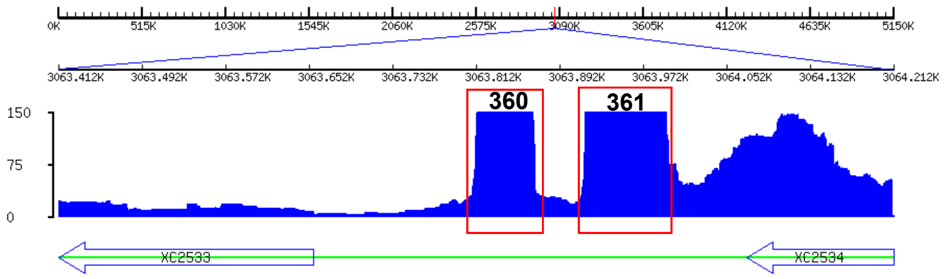

Transcript	Start	End	Length (bp)	RPKM	Inside gene	Overlap gene	Upstream gene	Downstream gene
TT362	3066078	3066187	110	534.3	XC2535	Null	XC2534	XC2536

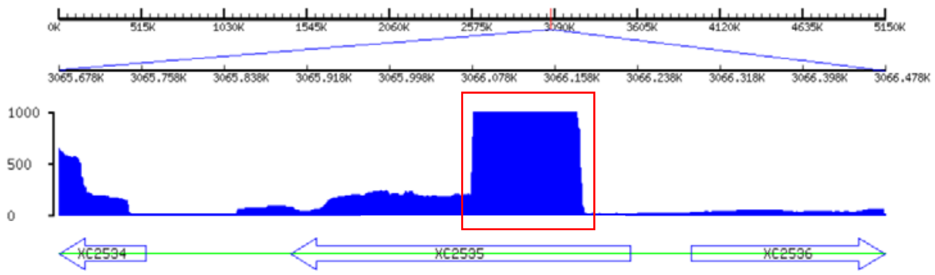

Transcript	Start	End	Length (bp)	RPKM	Inside gene	Overlap gene	Upstream gene	Downstream gene
TT363	3068395	3068473	79	39.7	Null	XC2537	XC2536	XC2538

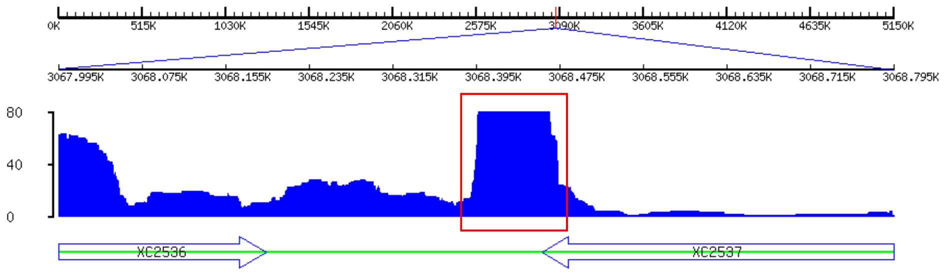

Transcript	Start	End	Length (bp)	RPKM	Inside gene	Overlap gene	Upstream gene	Downstream gene
TT364	3077469	3077539	71	348.1	XC2545	Null	XC2544	XC2546

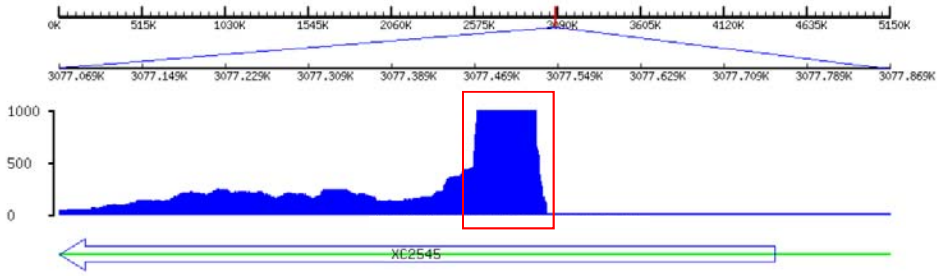

Transcript	Start	End	Length (bp)	RPKM	Inside gene	Overlap gene	Upstream gene	Downstream gene
TT365	3132991	3133083	93	38.4	Null	Null	XC2595	XC2596

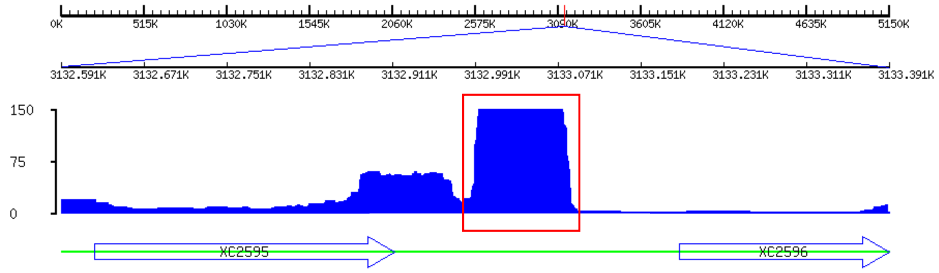

Transcript	Start	End	Length (bp)	RPKM	Inside gene	Overlap gene	Upstream gene	Downstream gene
TT366	3148184	3148415	231	203.4	Null	XC2607	XC2606	XC2608

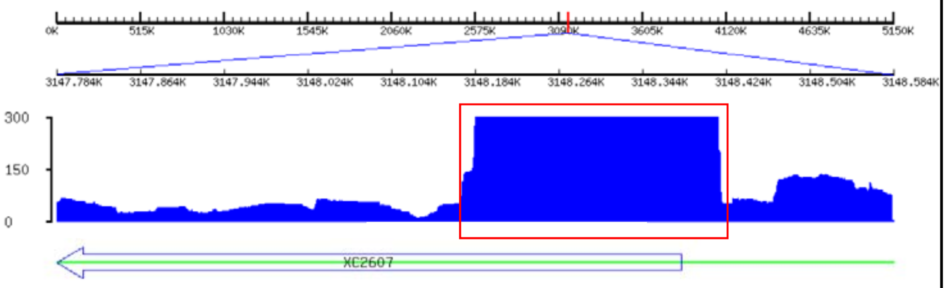

Transcript	Start	End	Length (bp)	RPKM	Inside gene	Overlap gene	Upstream gene	Downstream gene
TT367	3148927	3149006	90	629.5	Null	Null	XC2607	XC2608

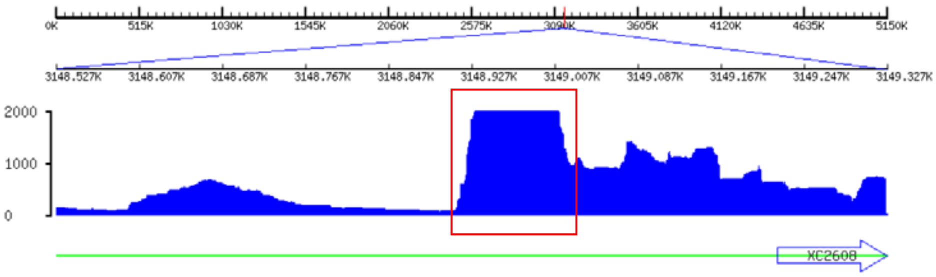

Transcript	Start	End	Length (bp)	RPKM	Inside gene	Overlap gene	Upstream gene	Downstream gene
TT368	3167243	3167332	90	161.7	Null	XC2630	XC2629	XC2631

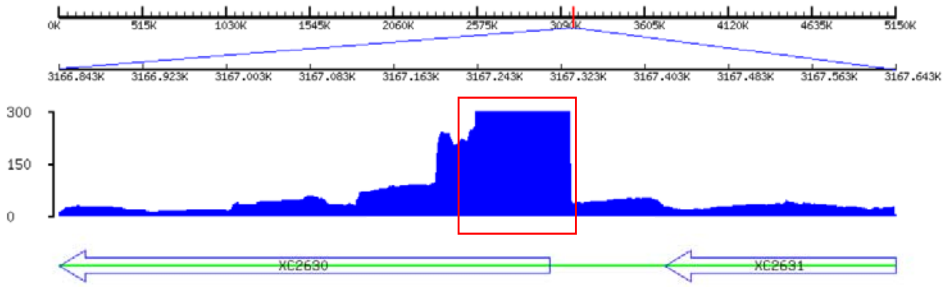

Transcript	Start	End	Length (bp)	RPKM	Inside gene	Overlap gene	Upstream gene	Downstream gene
TT369	3167739	3167812	74	242.2	Null	XC2631	XC2630	XC2632
TT370	3167857	3167944	88	184.6	XC2632	Null	XC2631	XC2633

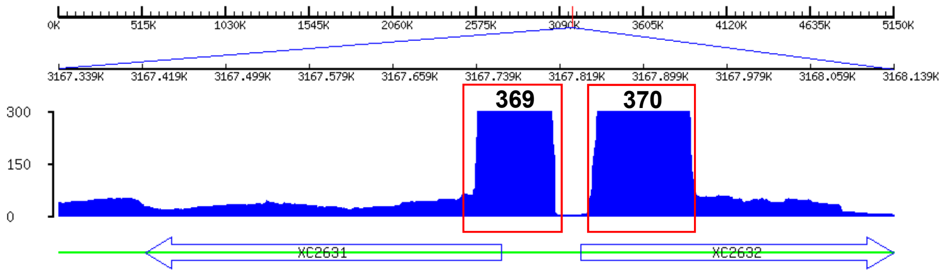

Transcript	Start	End	Length (bp)	RPKM	Inside gene	Overlap gene	Upstream gene	Downstream gene
TT371	3173234	3173295	62	153.9	XC2636	Null	XC2635	XC2637

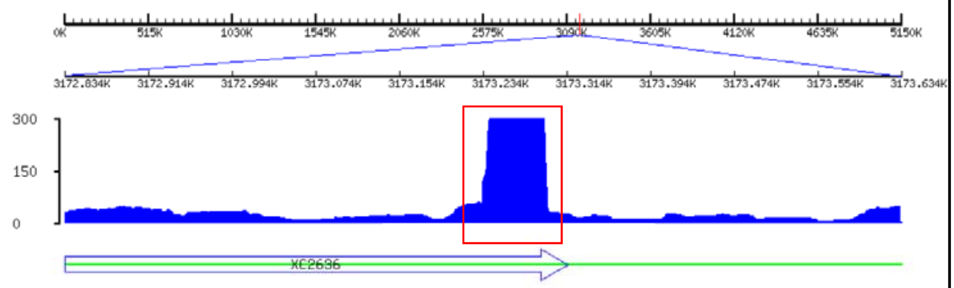

Transcript	Start	End	Length (bp)	RPKM	Inside gene	Overlap gene	Upstream gene	Downstream gene
TT372	3175544	3175610	67	171.9	XC2638	Null	XC2637	XC4360

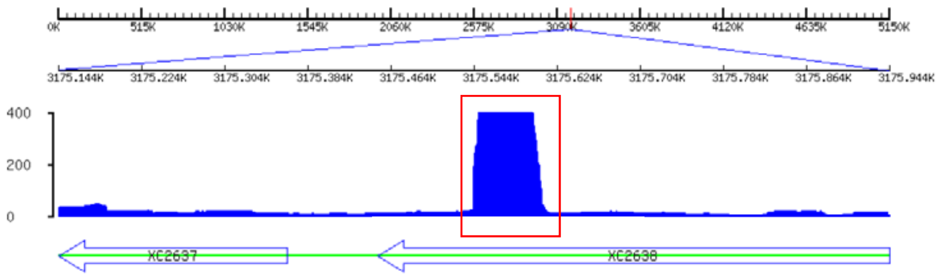

Transcript	Start	End	Length (bp)	RPKM	Inside gene	Overlap gene	Upstream gene	Downstream gene
TT373	3176577	3176723	147	398	XC2638	Null	XC2637	XC4360

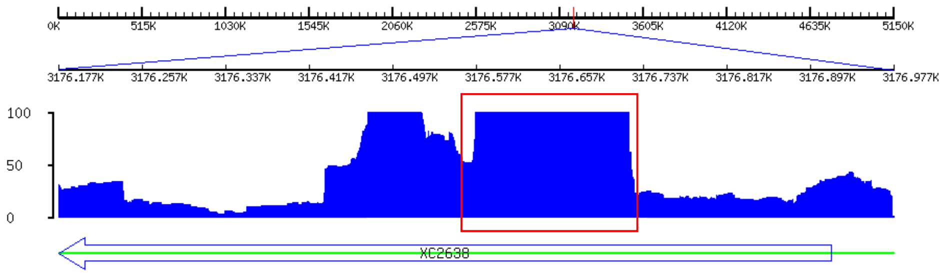

Transcript	Start	End	Length (bp)	RPKM	Inside gene	Overlap gene	Upstream gene	Downstream gene
TT374	3177001	3177086	90	411.5	XC4360	Null	XC2638	XC2639
TT375	3177097	3177203	107	191.1	Null	Null	XC4360	XC2639

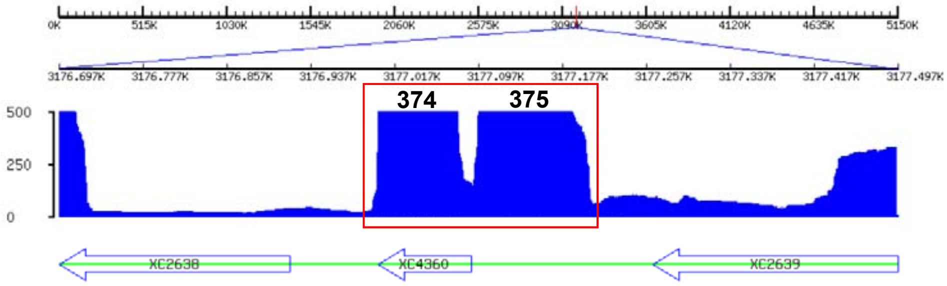

Transcript	Start	End	Length (bp)	RPKM	Inside gene	Overlap gene	Upstream gene	Downstream gene
TT376	3180693	3180759	67	351.2	XC2642	Null	XC2641	XC2643

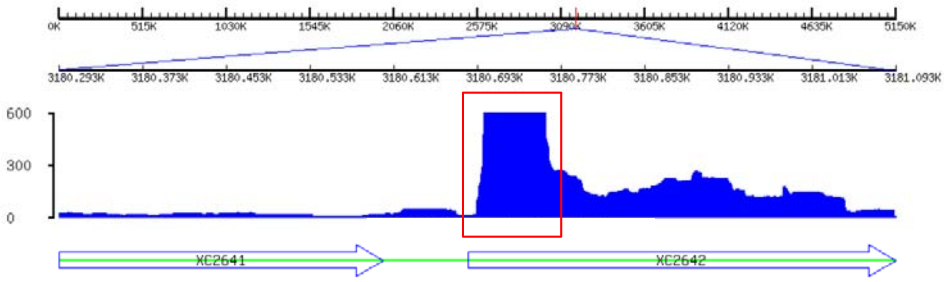

Transcript	Start	End	Length (bp)	RPKM	Inside gene	Overlap gene	Upstream gene	Downstream gene
TT377	3184370	3184450	81	34.4	XC2645	Null	XC2644	XC2646

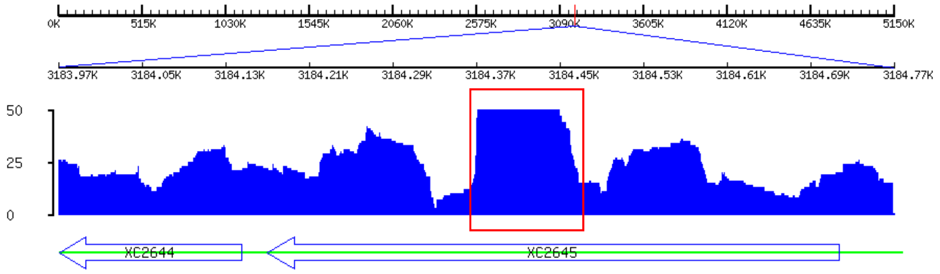

Transcript	Start	End	Length (bp)	RPKM	Inside gene	Overlap gene	Upstream gene	Downstream gene
TT378	3188220	3188336	117	167.4	Null	XC2650	XC2649	XC2651

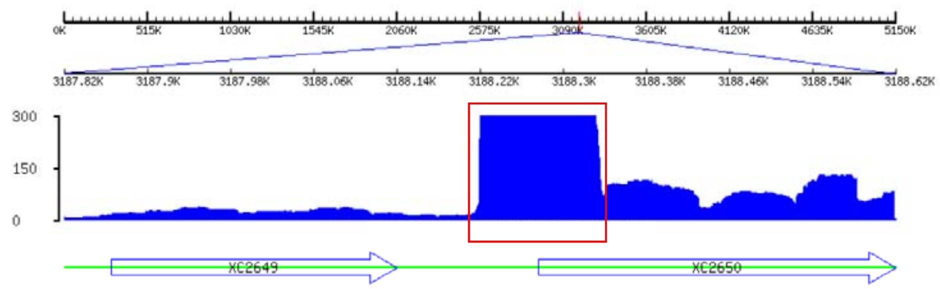

Transcript	Start	End	Length (bp)	RPKM	Inside gene	Overlap gene	Upstream gene	Downstream gene
TT379	3188900	3188954	55	1677.7	Null	Null	XC2650	XC2651

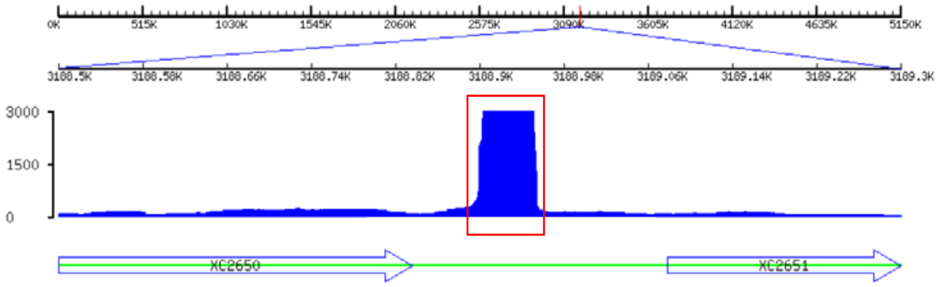

Transcript	Start	End	Length (bp)	RPKM	Inside gene	Overlap gene	Upstream gene	Downstream gene
TT380	3219243	3219349	107	147.1	Null	XC2672	XC2671	XC2673

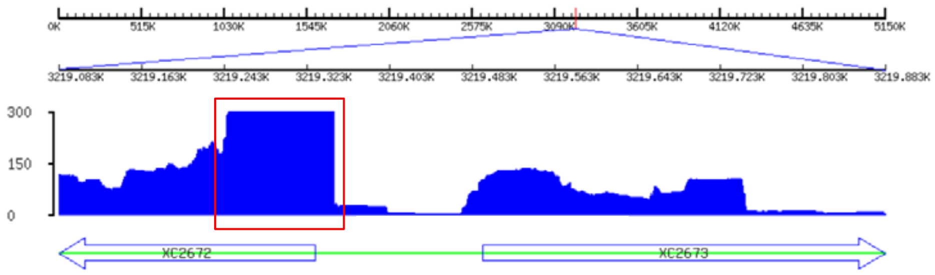

Transcript	Start	End	Length (bp)	RPKM	Inside gene	Overlap gene	Upstream gene	Downstream gene
TT381	3245880	3245946	67	843.6	Null	Null	XC2699	XC2700

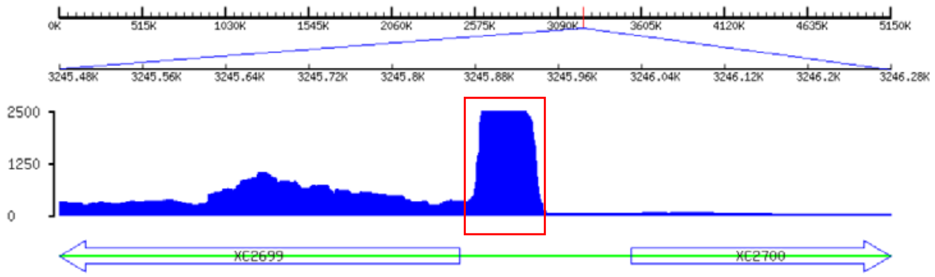

Transcript	Start	End	Length (bp)	RPKM	Inside gene	Overlap gene	Upstream gene	Downstream gene
TT382	3267573	3267649	77	5400.3	XC4361	Null	XC2718	XC2719

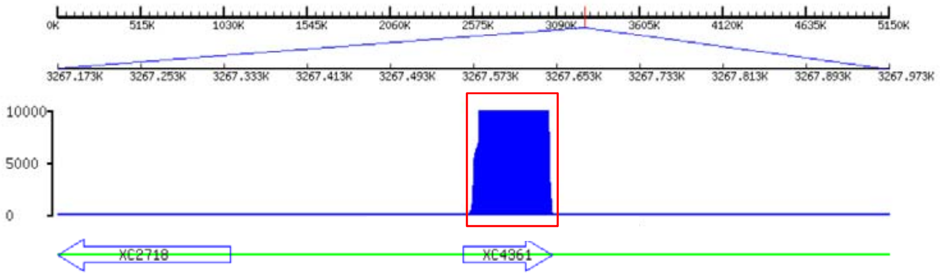

Transcript	Start	End	Length (bp)	RPKM	Inside gene	Overlap gene	Upstream gene	Downstream gene
TT383	3283476	3283530	55	86.2	Null	XC2731	XC2730	XC2732

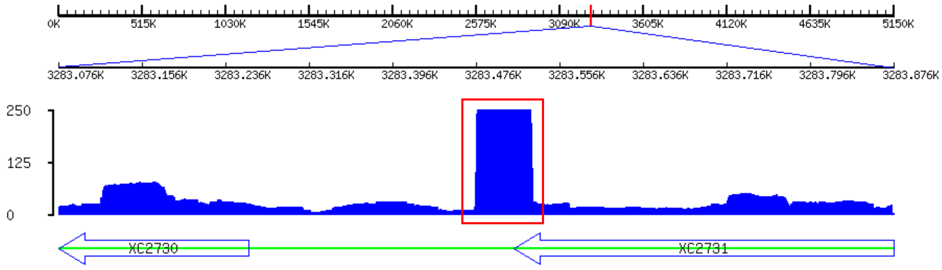

Transcript	Start	End	Length (bp)	RPKM	Inside gene	Overlap gene	Upstream gene	Downstream gene
TT384	3293595	3293674	80	37.5	Null	Null	XC2742	XC2743

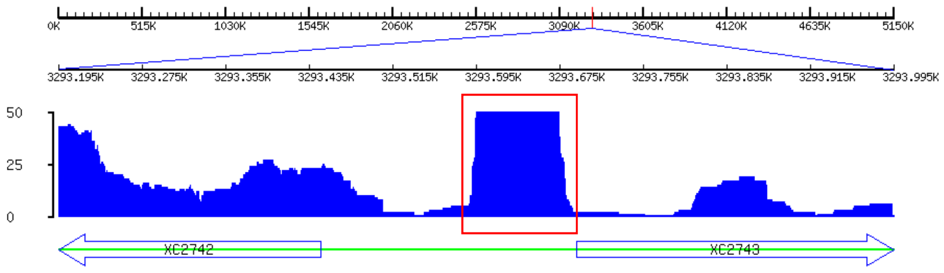

Transcript	Start	End	Length (bp)	RPKM	Inside gene	Overlap gene	Upstream gene	Downstream gene
TT385	3300714	3300975	262	362.8	XC2749	Null	XC2748	XC2750

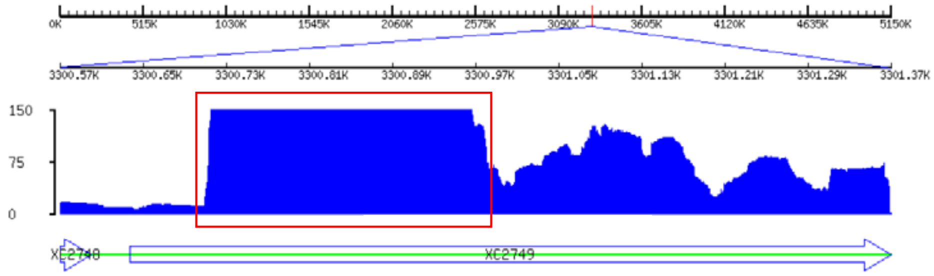

Transcript	Start	End	Length (bp)	RPKM	Inside gene	Overlap gene	Upstream gene	Downstream gene
TT386	3320361	3320415	55	147.6	XC2763	Null	XC2762	XC2764

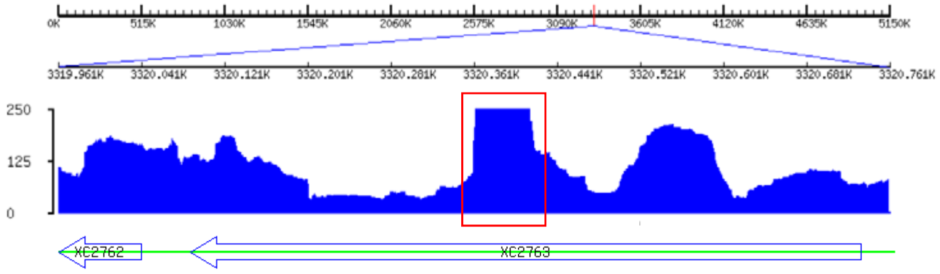

Transcript	Start	End	Length (bp)	RPKM	Inside gene	Overlap gene	Upstream gene	Downstream gene
TT387	3322074	3322155	82	319.6	XC2764	Null	XC2763	XC2765

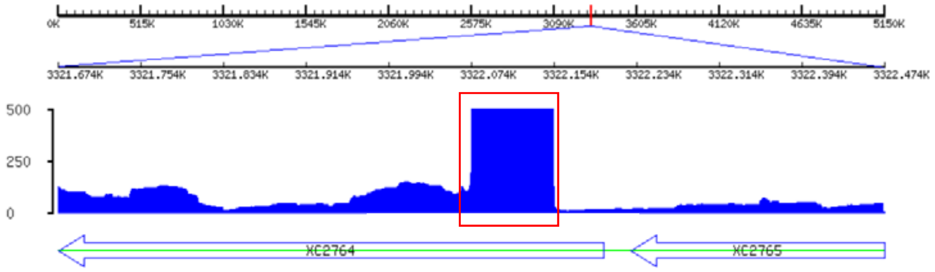

Transcript	Start	End	Length (bp)	RPKM	Inside gene	Overlap gene	Upstream gene	Downstream gene
TT388	3326115	3326169	55	292.9	Null	XC2767	XC2766	XC2768

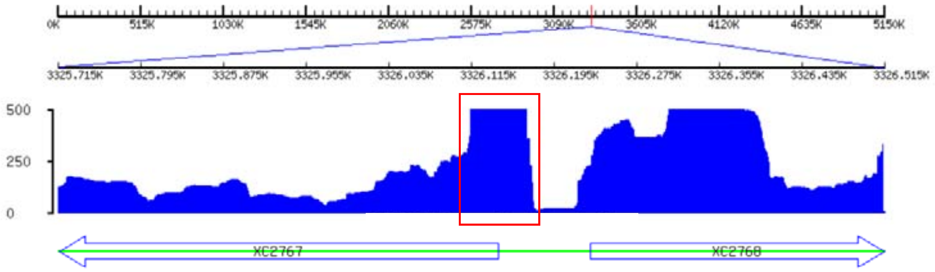

Transcript	Start	End	Length (bp)	RPKM	Inside gene	Overlap gene	Upstream gene	Downstream gene
TT389	3330944	3331397	454	195507.2	XC4362	Null	XC2772	XC2773

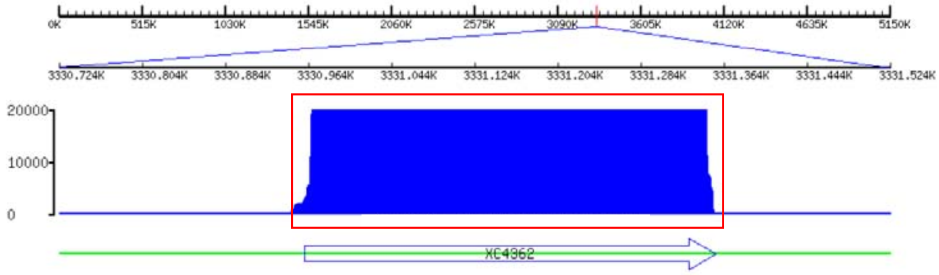

Transcript	Start	End	Length (bp)	RPKM	Inside gene	Overlap gene	Upstream gene	Downstream gene
TT390	3383191	3383473	282	709.1	XC2810	Null	XC2809	XC2811

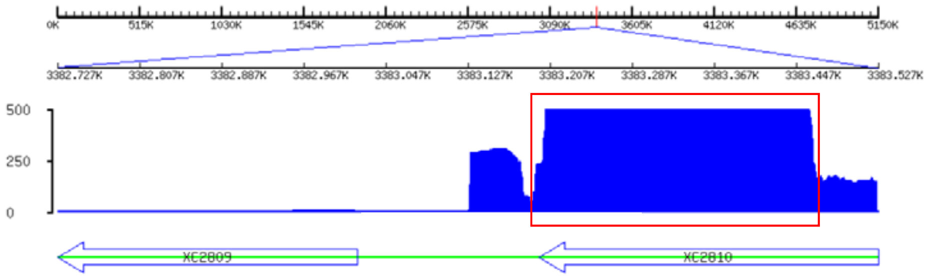

Transcript	Start	End	Length (bp)	RPKM	Inside gene	Overlap gene	Upstream gene	Downstream gene
TT391	3383686	3383997	311	383.5	Null	XC2810	XC2809	XC2811

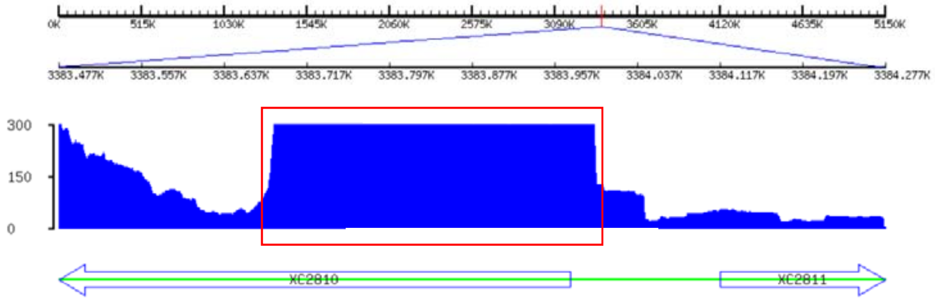

Transcript	Start	End	Length (bp)	RPKM	Inside gene	Overlap gene	Upstream gene	Downstream gene
TT392	3386925	3387070	146	424.8	Null	XC2815	XC2814	XC2816
TT393	3387271	3387359	89	139.1	Null	XC2816	XC2815	XC2817
TT394	3387384	3387473	90	367.5	Null	Null	XC2816	XC2817

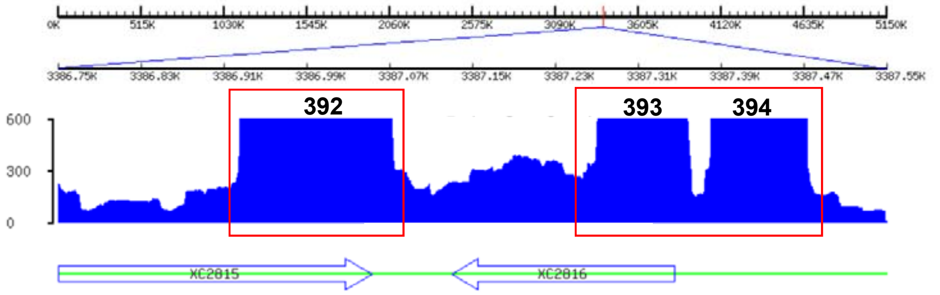

Transcript	Start	End	Length (bp)	RPKM	Inside gene	Overlap gene	Upstream gene	Downstream gene
TT395	3388879	3388930	52	166.9	XC2818	Null	XC2817	XC2819

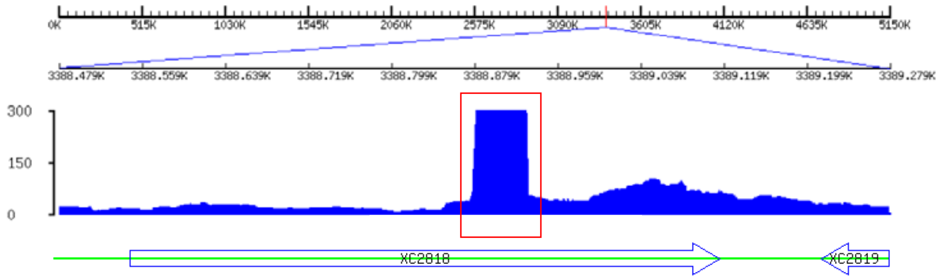

Transcript	Start	End	Length (bp)	RPKM	Inside gene	Overlap gene	Upstream gene	Downstream gene
TT396	3395492	3395587	96	201.1	Null	XC2824	XC2823	XC2825
TT397	3395646	3395735	90	90.8	XC2824	Null	XC2823	XC2825

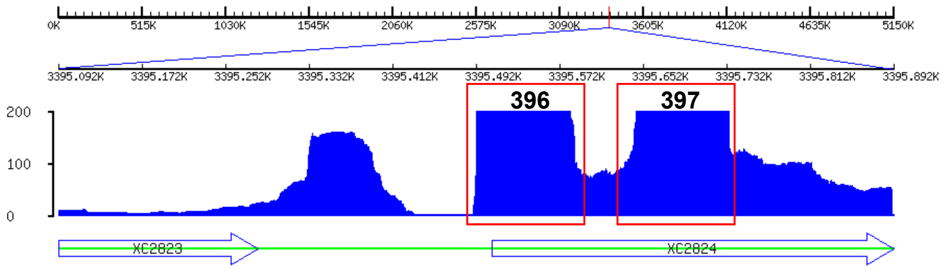

Transcript	Start	End	Length (bp)	RPKM	Inside gene	Overlap gene	Upstream gene	Downstream gene
TT398	3396012	3396079	68	137.1	XC2824	Null	XC2823	XC2825
TT399	3396261	3396372	112	74.1	Null	XC2824	XC2823	XC2825

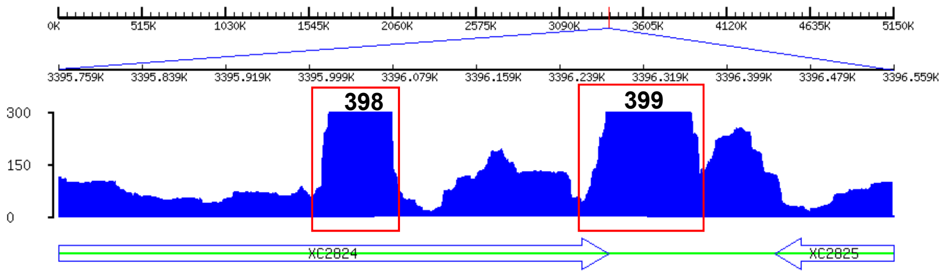

Transcript	Start	End	Length (bp)	RPKM	Inside gene	Overlap gene	Upstream gene	Downstream gene
TT400	3398279	3398362	84	24.9	XC2826	Null	XC2825	XC2827

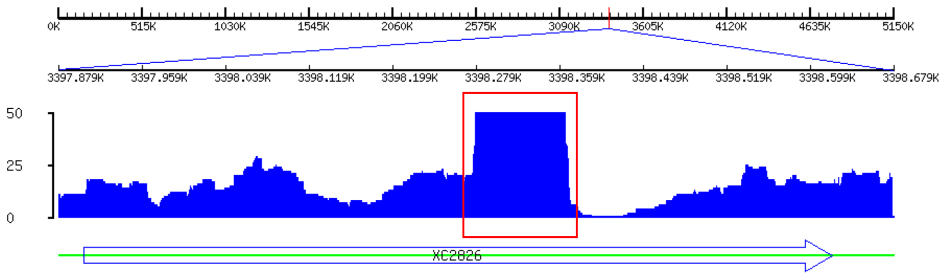

Transcript	Start	End	Length (bp)	RPKM	Inside gene	Overlap gene	Upstream gene	Downstream gene
TT401	3399972	3400047	76	136.1	Null	XC2827	XC2826	XC2828

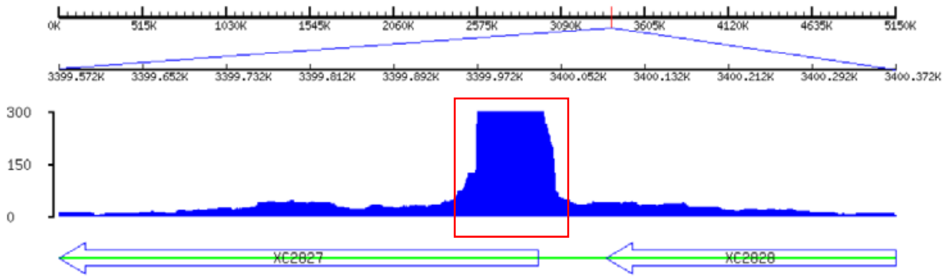

Transcript	Start	End	Length (bp)	RPKM	Inside gene	Overlap gene	Upstream gene	Downstream gene
TT402	3424761	3424838	78	25.8	XC2846	Null	XC2845	XC2847

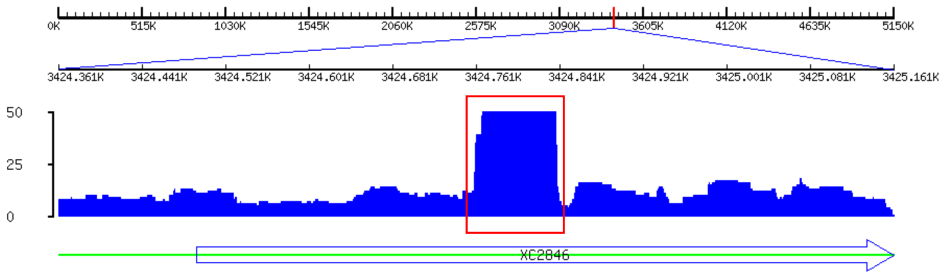

Transcript	Start	End	Length (bp)	RPKM	Inside gene	Overlap gene	Upstream gene	Downstream gene
TT403	3429490	3429577	88	150.1	Null	Null	XC2848	XC2850

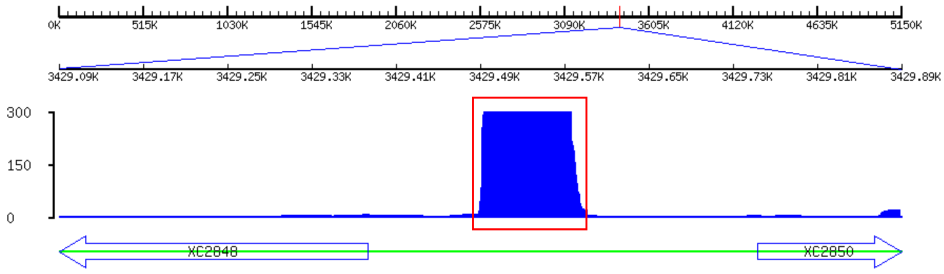

Transcript	Start	End	Length (bp)	RPKM	Inside gene	Overlap gene	Upstream gene	Downstream gene
TT404	3443612	3443735	124	446.3	Null	Null	XC2862	XC2863

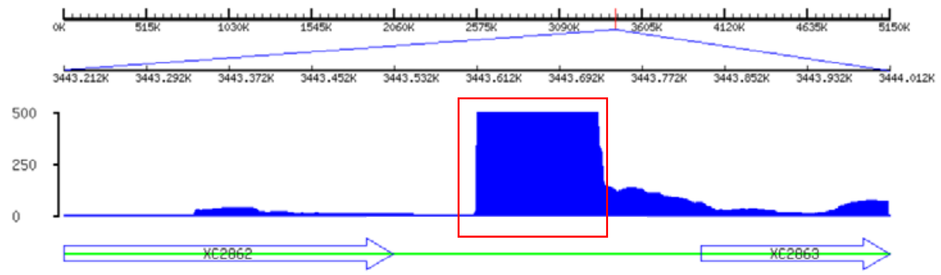

Transcript	Start	End	Length (bp)	RPKM	Inside gene	Overlap gene	Upstream gene	Downstream gene
TT405	3444555	3444617	63	172.3	XC2863	Null	XC2862	XC2864

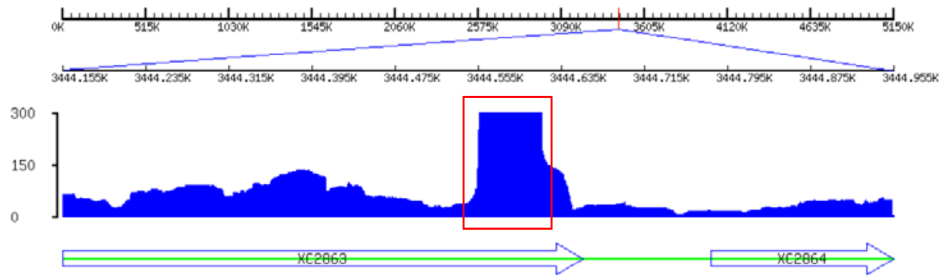

Transcript	Start	End	Length (bp)	RPKM	Inside gene	Overlap gene	Upstream gene	Downstream gene
TT406	3448563	3448651	89	150.3	Null	XC2868	XC2867	XC2869
TT407	3448741	3448795	55	71.6	XC2868	Null	XC2867	XC2869

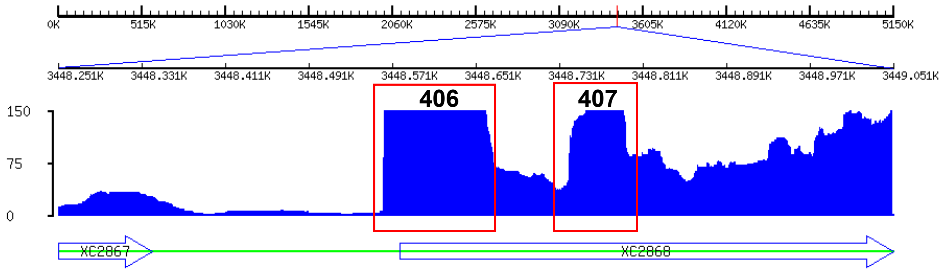

Transcript	Start	End	Length (bp)	RPKM	Inside gene	Overlap gene	Upstream gene	Downstream gene
TT408	3449931	3449984	54	51.1	XC2870	Null	XC2869	XC2871

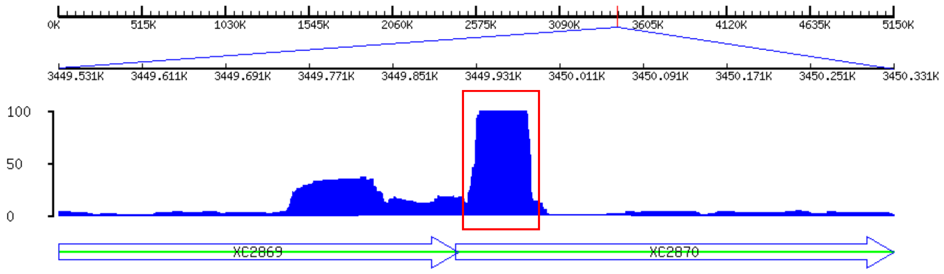

Transcript	Start	End	Length (bp)	RPKM	Inside gene	Overlap gene	Upstream gene	Downstream gene
TT409	3451808	3452033	225	92.3	Null	XC2871/XC2872	XC2870	XC2873

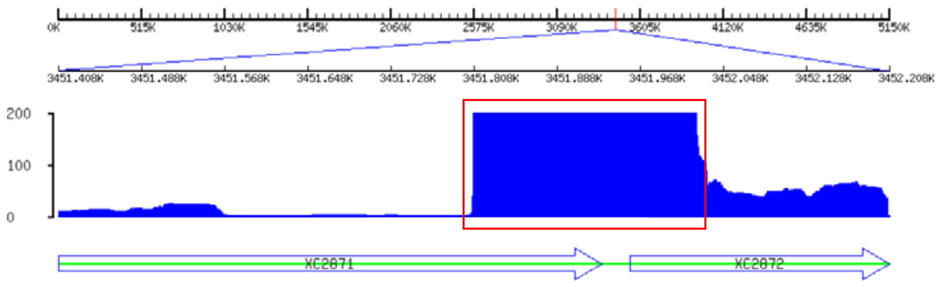

Transcript	Start	End	Length (bp)	RPKM	Inside gene	Overlap gene	Upstream gene	Downstream gene
TT410	3453636	3453711	76	81.5	XC2873	Null	XC2872	XC2874

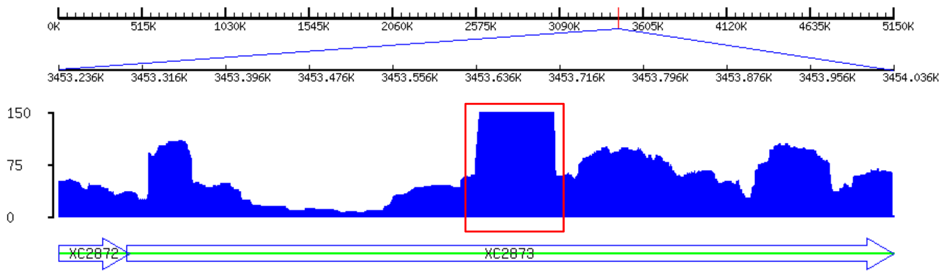

Transcript	Start	End	Length (bp)	RPKM	Inside gene	Overlap gene	Upstream gene	Downstream gene
TT411	3455442	3455508	67	78.8	XC2873	Null	XC2872	XC2874
TT412	3455703	3455769	67	130.8	Null	XC2873	XC2872	XC2874

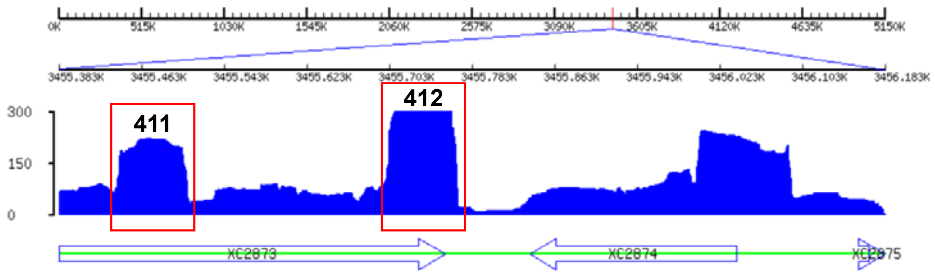

Transcript	Start	End	Length (bp)	RPKM	Inside gene	Overlap gene	Upstream gene	Downstream gene
TT413	3467621	3467730	110	819.6	XC2885	Null	XC2884	XC2886

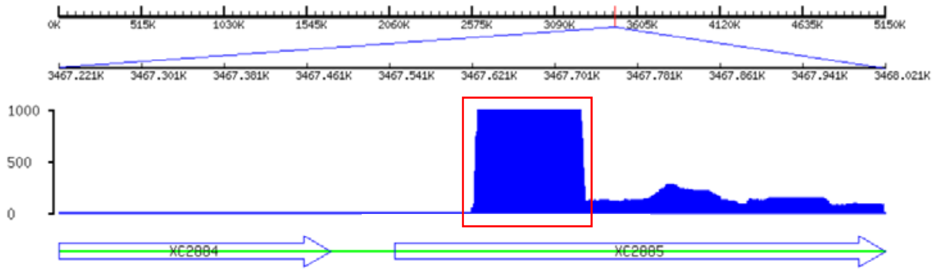

Transcript	Start	End	Length (bp)	RPKM	Inside gene	Overlap gene	Upstream gene	Downstream gene
TT414	3468615	3468951	336	287.4	Null	XC2885	XC2884	XC2886

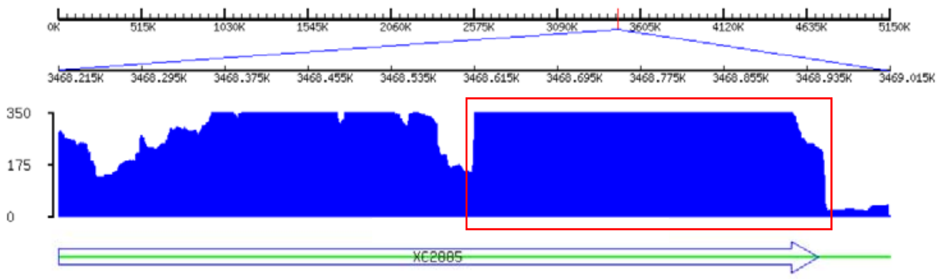

Transcript	Start	End	Length (bp)	RPKM	Inside gene	Overlap gene	Upstream gene	Downstream gene
TT415	3468827	3468901	74	574.8	XC2885	Null	XC2884	XC2886

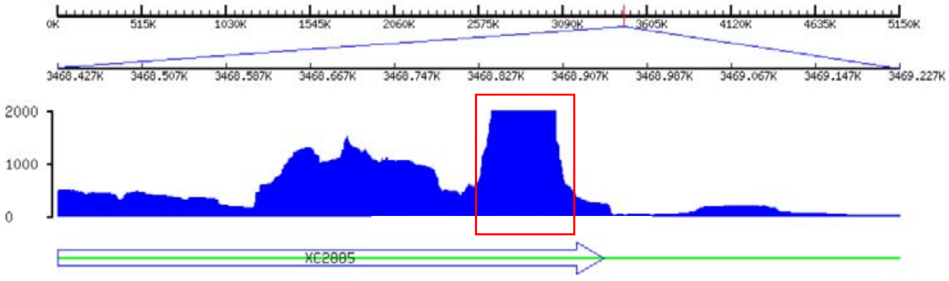

Transcript	Start	End	Length (bp)	RPKM	Inside gene	Overlap gene	Upstream gene	Downstream gene
TT416	3469025	3469110	85	43.2	Null	Null	XC2885	XC2886

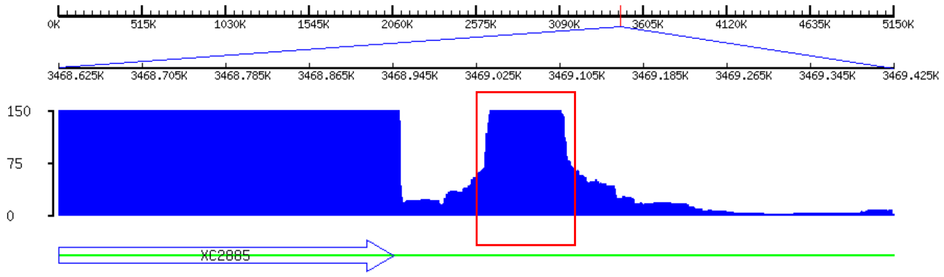

Transcript	Start	End	Length (bp)	RPKM	Inside gene	Overlap gene	Upstream gene	Downstream gene
TT417	3481118	3481187	70	338.4	Null	XC2897	XC2896	XC2898
TT418	3481288	3481348	60	91.1	Null	XC2898	XC2897	XC2899

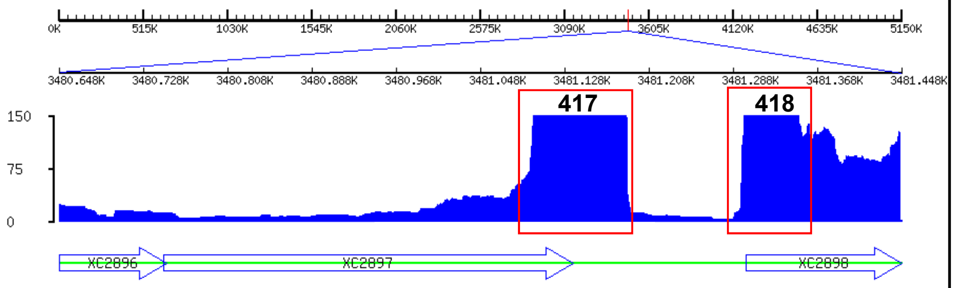

Transcript	Start	End	Length (bp)	RPKM	Inside gene	Overlap gene	Upstream gene	Downstream gene
TT419	3481945	3482023	78	349.8	Null	Null	XC2898	XC2899

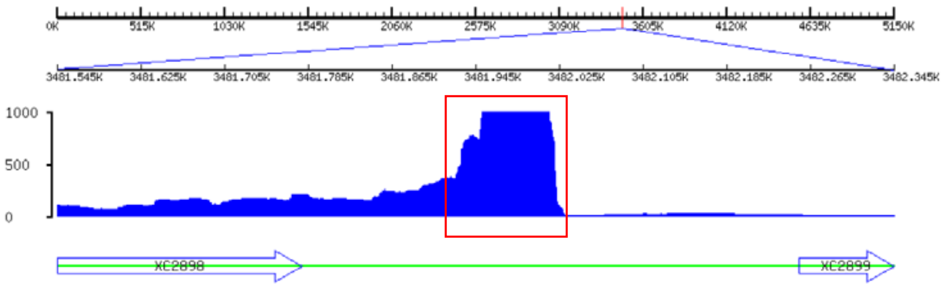

Transcript	Start	End	Length (bp)	RPKM	Inside gene	Overlap gene	Upstream gene	Downstream gene
TT420	3487609	3487715	107	105.9	Null	Null	XC2900	XC2901

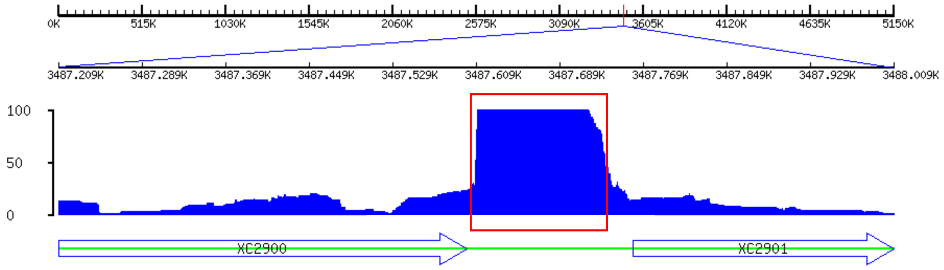

Transcript	Start	End	Length (bp)	RPKM	Inside gene	Overlap gene	Upstream gene	Downstream gene
TT421	3500373	3500433	61	67.7	Null	Null	XC2915	XC2916

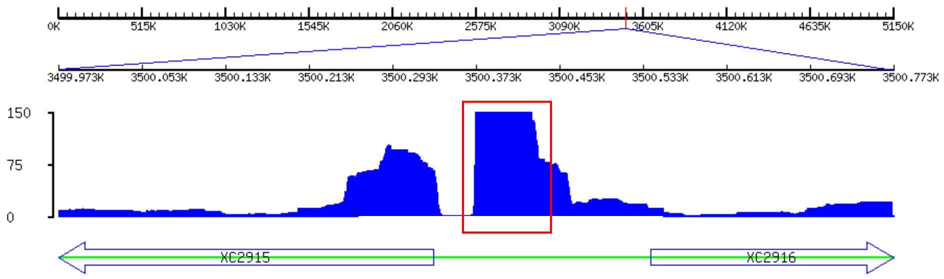

Transcript	Start	End	Length (bp)	RPKM	Inside gene	Overlap gene	Upstream gene	Downstream gene
TT422	3506655	3506749	95	1124.5	Null	XC2921	XC2920	XC2922

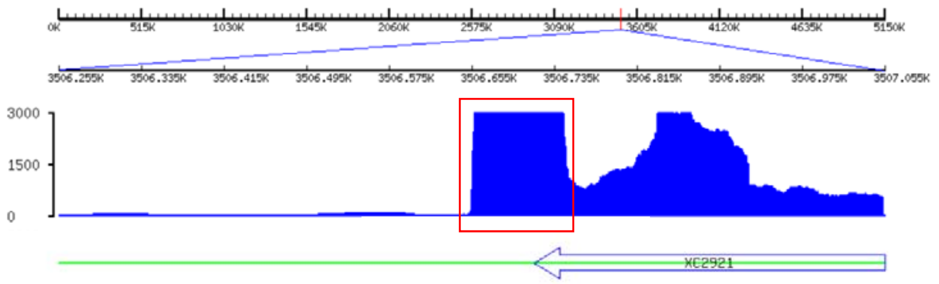

Transcript	Start	End	Length (bp)	RPKM	Inside gene	Overlap gene	Upstream gene	Downstream gene
TT423	3518444	3518523	79	125.5	XC2933	Null	XC2932	XC2934

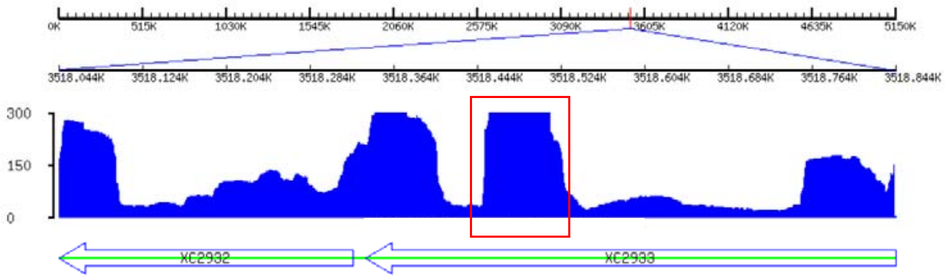

Transcript	Start	End	Length (bp)	RPKM	Inside gene	Overlap gene	Upstream gene	Downstream gene
TT424	3519275	3519326	52	213.4	XC2934	Null	XC2933	XC2935

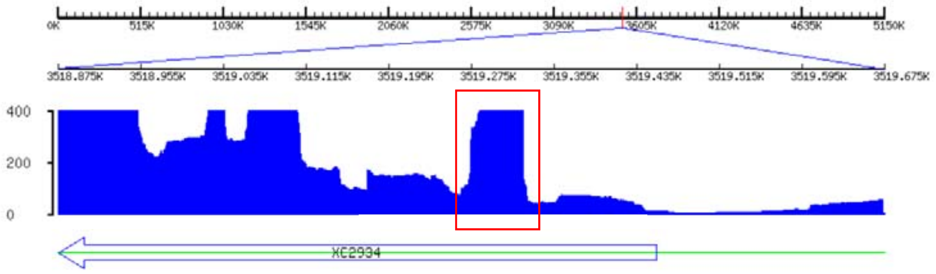

Transcript	Start	End	Length (bp)	RPKM	Inside gene	Overlap gene	Upstream gene	Downstream gene
TT425	3519856	3519935	80	118.2	Null	Null	XC2934	XC2935

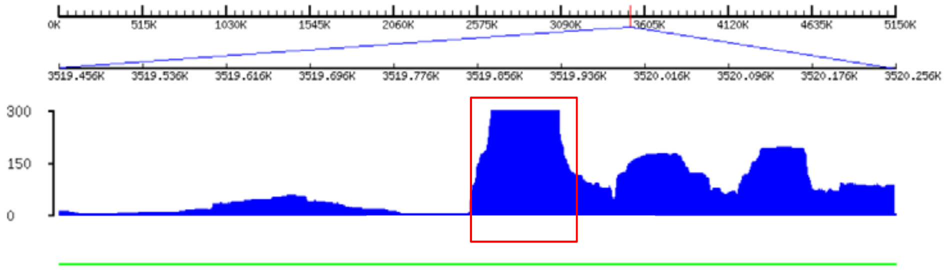

Transcript	Start	End	Length (bp)	RPKM	Inside gene	Overlap gene	Upstream gene	Downstream gene
TT426	3521764	3521855	91	65.2	Null	XC2937	XC2936	XC2938

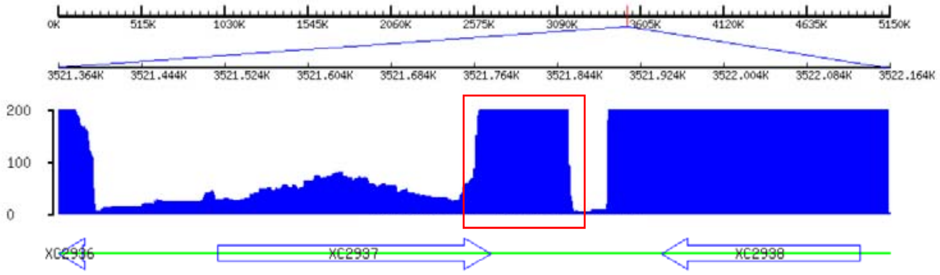

Transcript	Start	End	Length (bp)	RPKM	Inside gene	Overlap gene	Upstream gene	Downstream gene
TT427	3521892	3512207	316	494.9	XC2938	Null	XC2937	XC2939

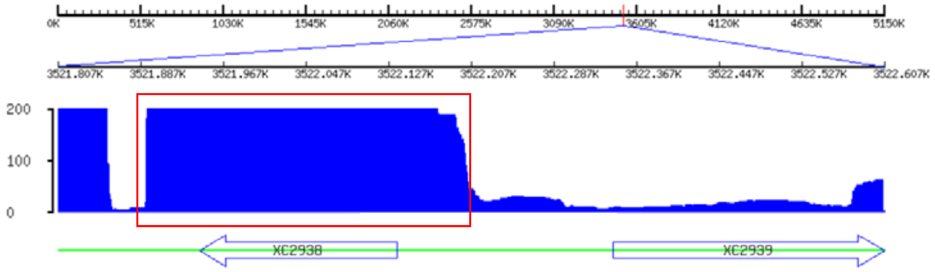

Transcript	Start	End	Length (bp)	RPKM	Inside gene	Overlap gene	Upstream gene	Downstream gene
TT428	3522100	3522168	68	1754.6	Null	XC2938	XC2937	XC2939

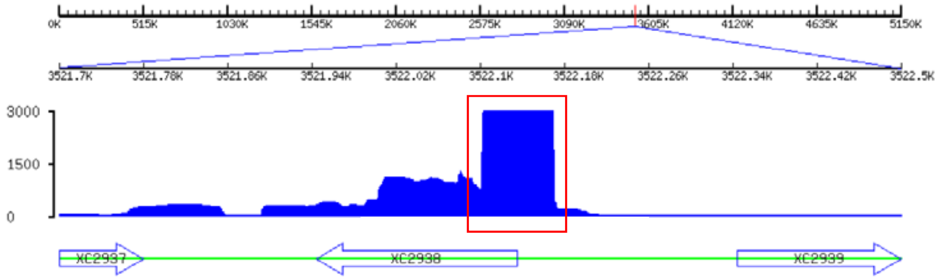

Transcript	Start	End	Length (bp)	RPKM	Inside gene	Overlap gene	Upstream gene	Downstream gene
TT429	3523176	3523265	90	72.1	Null	Null	XC2940	XC2941

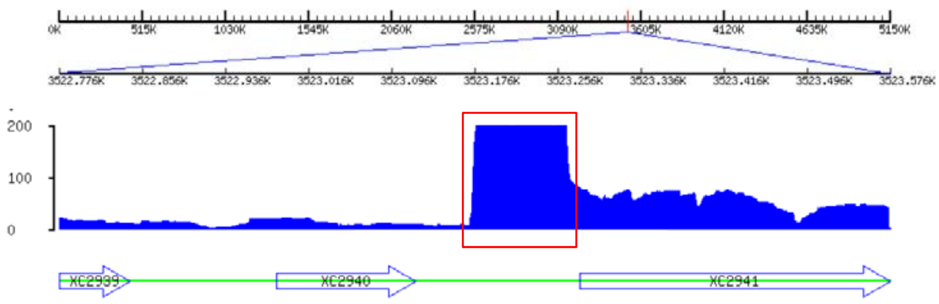

Transcript	Start	End	Length (bp)	RPKM	Inside gene	Overlap gene	Upstream gene	Downstream gene
TT430	3527193	3527272	80	158.2	XC2942	Null	XC2941	XC2943
TT431	3527492	3527576	85	71.3	Null	XC2943	XC2942	XC2944

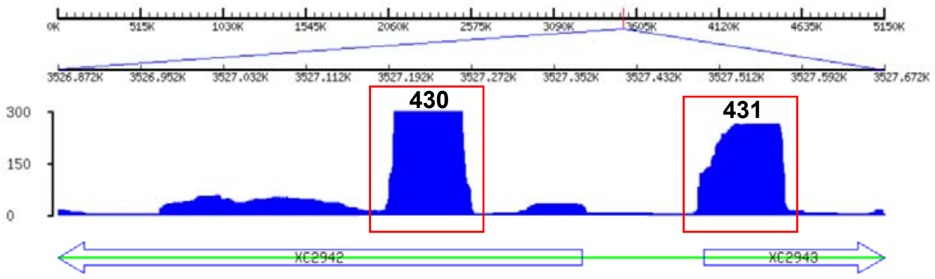

Transcript	Start	End	Length (bp)	RPKM	Inside gene	Overlap gene	Upstream gene	Downstream gene
TT432	3528755	3528811	57	360.2	Null	XC2944	XC2943	XC2945
TT433	3528827	3528940	114	100.4	XC2944	Null	XC2943	XC2945
TT434	3528983	3529097	115	49.2	XC2944	Null	XC2943	XC2945
TT435	3529123	3529194	71	147.9	Null	XC2944	XC2943	XC2945

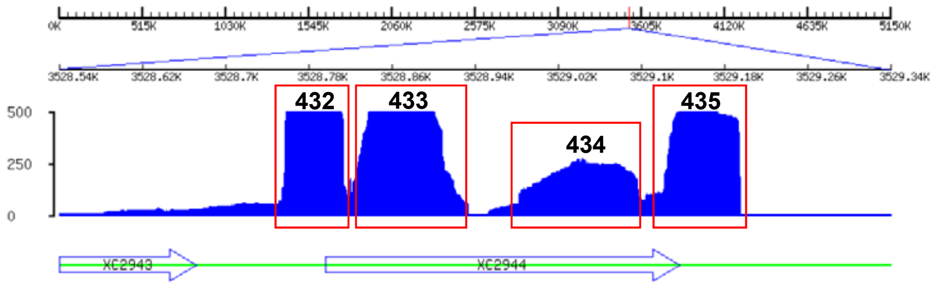

Transcript	Start	End	Length (bp)	RPKM	Inside gene	Overlap gene	Upstream gene	Downstream gene
TT436	3531660	3531777	118	31.8	Null	XC2947	XC2946	XC2948

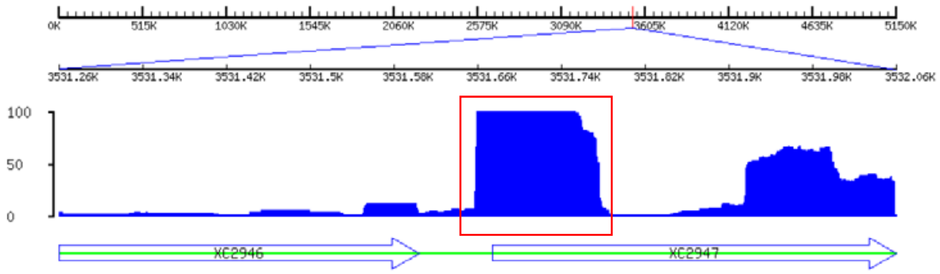

Transcript	Start	End	Length (bp)	RPKM	Inside gene	Overlap gene	Upstream gene	Downstream gene
TT437	3549990	3550063	74	834.1	Null	XC2965	XC2964	XC2966

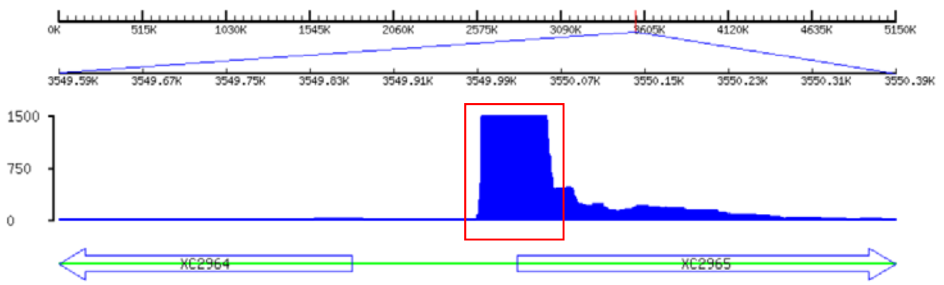

Transcript	Start	End	Length (bp)	RPKM	Inside gene	Overlap gene	Upstream gene	Downstream gene
TT438	3558182	3558251	70	116.7	Null	XC2972	XC2971	XC2973

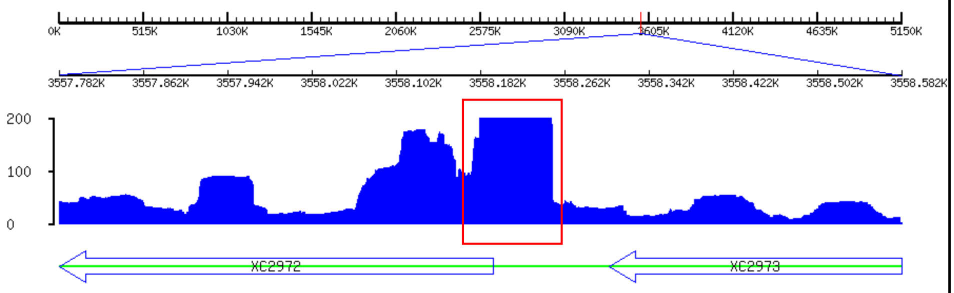

Transcript	Start	End	Length (bp)	RPKM	Inside gene	Overlap gene	Upstream gene	Downstream gene
TT439	3559134	3559223	90	293.9	Null	XC2973 XC2974	XC2972	XC2975
TT440	3559248	3559346	98	999.5	XC2974	Null	XC2973	XC2975

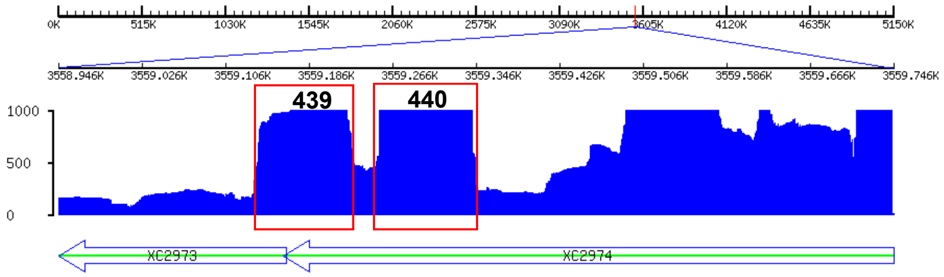

Transcript	Start	End	Length (bp)	RPKM	Inside gene	Overlap gene	Upstream gene	Downstream gene
TT441	3559708	3559804	97	1462.7	Null	XC2974	XC2973	XC2975

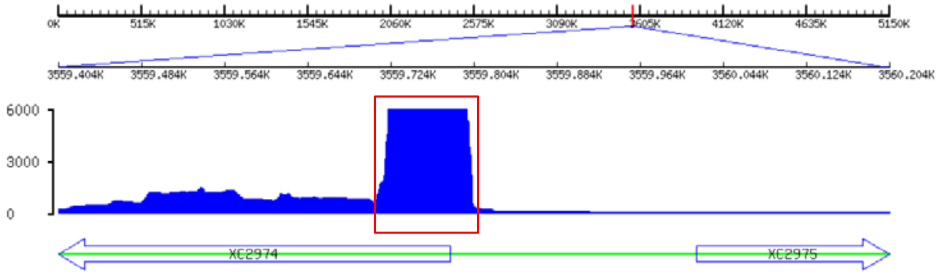

Transcript	Start	End	Length (bp)	RPKM	Inside gene	Overlap gene	Upstream gene	Downstream gene
TT442	3563159	3563274	116	28.6	Null	XC2976	XC2975	XC2977

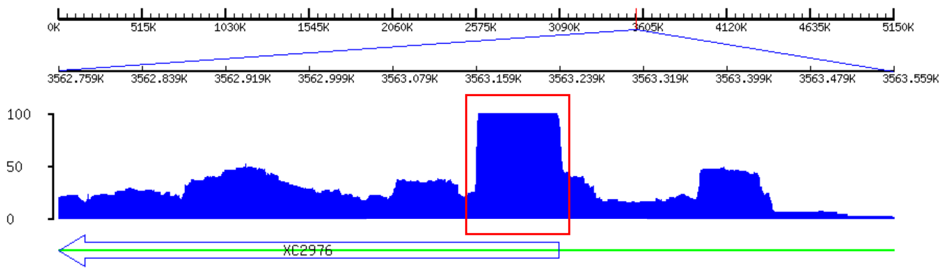

Transcript	Start	End	Length (bp)	RPKM	Inside gene	Overlap gene	Upstream gene	Downstream gene
TT443	3596895	3596986	91	145.2	Null	Null	XC3001	XC3002
TT444	3597210	3597299	90	168.8	Null	XC3002	XC3001	XC3003

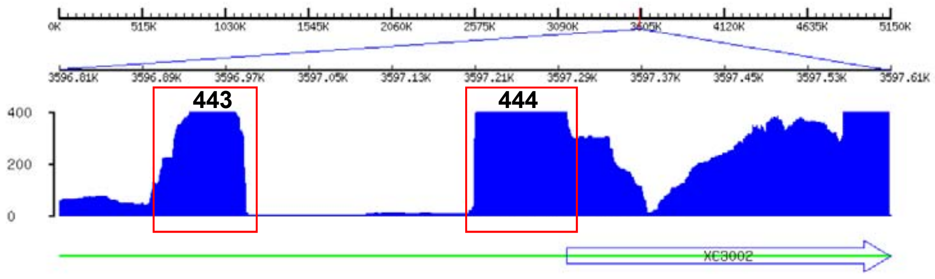

Transcript	Start	End	Length (bp)	RPKM	Inside gene	Overlap gene	Upstream gene	Downstream gene
TT445	3597565	3597654	90	174.5	XC3002	Null	XC3001	XC3003

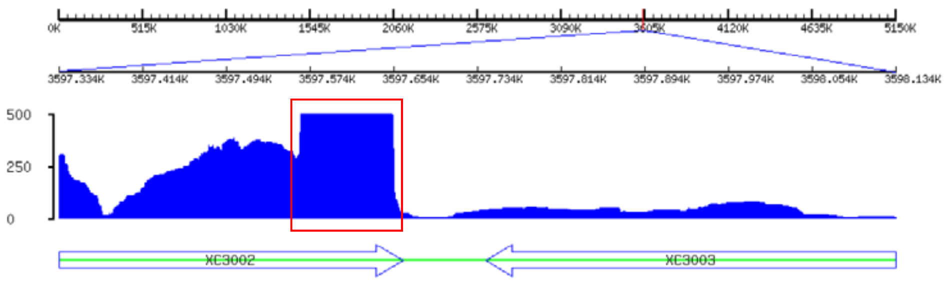

Transcript	Start	End	Length (bp)	RPKM	Inside gene	Overlap gene	Upstream gene	Downstream gene
TT446	3605262	3605378	117	75.6	Null	XC3011	XC3010	XC3012
TT447	3605549	3605652	104	303.1	Null	XC3012	XC3011	XC3013
TT448	3605682	3605743	61	268.6	XC3012	Null	XC3011	XC3013

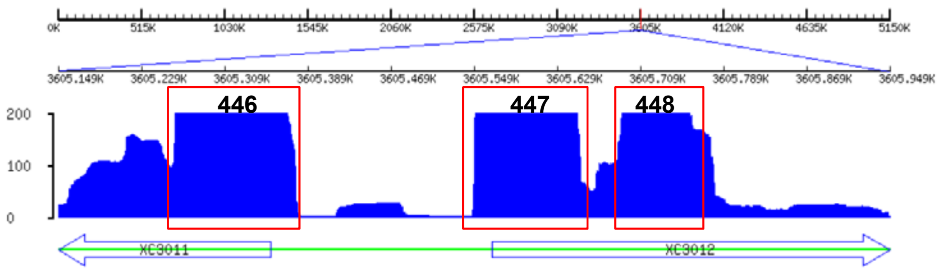

Transcript	Start	End	Length (bp)	RPKM	Inside gene	Overlap gene	Upstream gene	Downstream gene
TT449	3609150	3609243	94	400.3	Null	XC3014	XC3013	XC3015

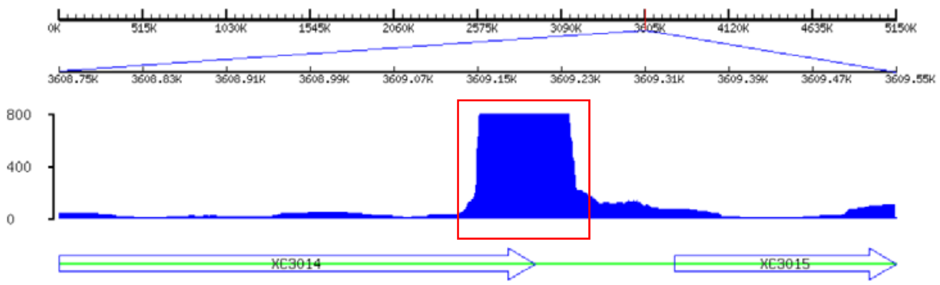

Transcript	Start	End	Length (bp)	RPKM	Inside gene	Overlap gene	Upstream gene	Downstream gene
TT450	3619443	3619520	78	210.6	Null	Null	XC3025	XC3026

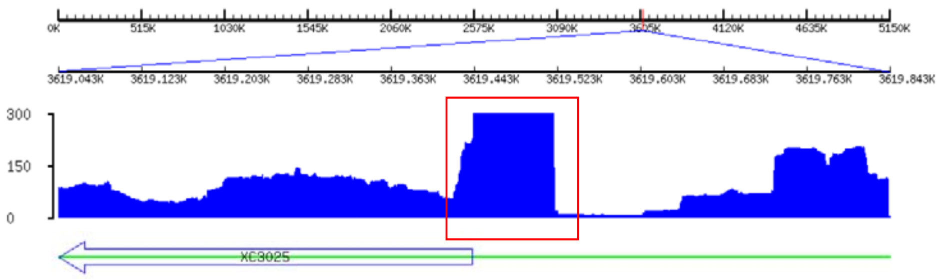

Transcript	Start	End	Length (bp)	RPKM	Inside gene	Overlap gene	Upstream gene	Downstream gene
TT451	3240766	3040810	44	36.2	Null	Null	XC3039	XC3040
TT452	3640864	3641322	459	88.5	XC3040	Null	XC3039	XC3041

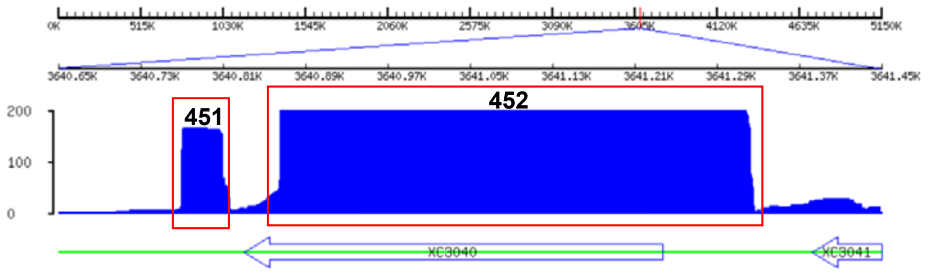

Transcript	Start	End	Length (bp)	RPKM	Inside gene	Overlap gene	Upstream gene	Downstream gene
TT453	3642707	3642822	116	247.7	Null	XC3042/XC3043	XC3041	XC3044
TT454	3642824	3642908	85	332.1	XC3043	Null	XC3042	XC3044
TT455	3642995	3643053	59	253.9	Null	XC3043	XC3042	XC3044

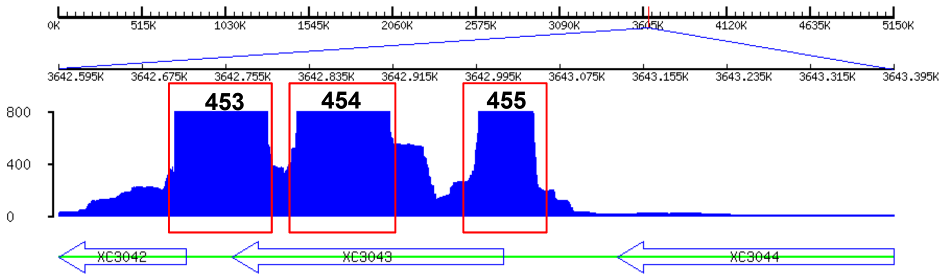

Transcript	Start	End	Length (bp)	RPKM	Inside gene	Overlap gene	Upstream gene	Downstream gene
TT456	3651162	3651256	95	30.2	XC3050	Null	XC3049	XC3051

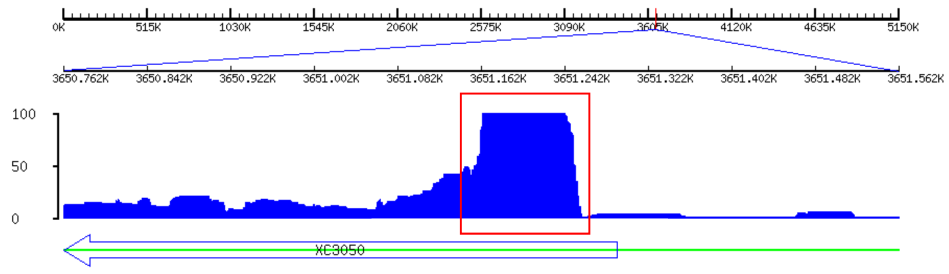

Transcript	Start	End	Length (bp)	RPKM	Inside gene	Overlap gene	Upstream gene	Downstream gene
TT457	3657767	3657856	90	81.6	XC3056	Null	XC3055	XC3057
TT458	3657890	3657983	93	76.7	XC3056	Null	XC3055	XC3057

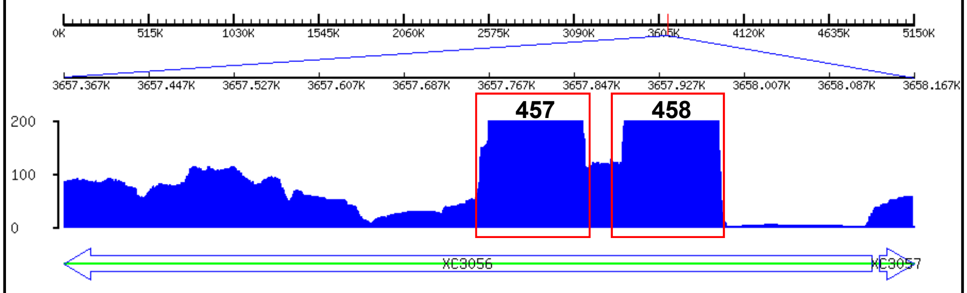

Transcript	Start	End	Length (bp)	RPKM	Inside gene	Overlap gene	Upstream gene	Downstream gene
TT459	3684840	3684929	90	126.3	Null	Null	XC3076	XC3077

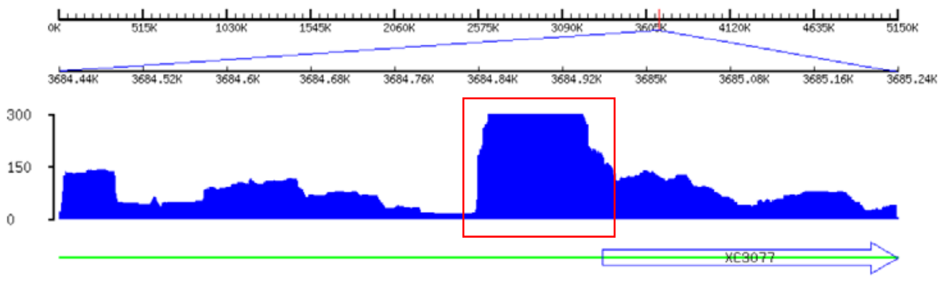

Transcript	Start	End	Length (bp)	RPKM	Inside gene	Overlap gene	Upstream gene	Downstream gene
TT460	3688259	3688335	77	161.1	Null	XC3079	XC3078	XC3080

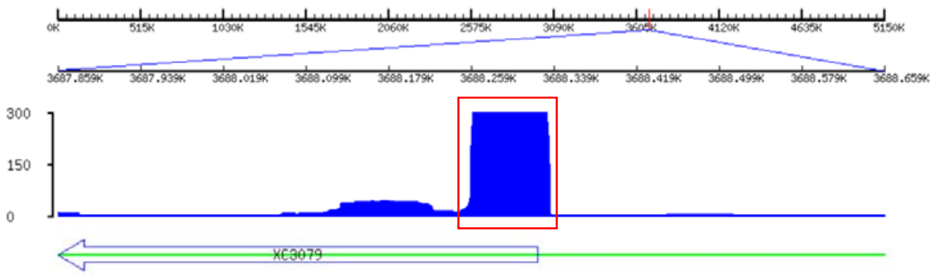

Transcript	Start	End	Length (bp)	RPKM	Inside gene	Overlap gene	Upstream gene	Downstream gene
TT461	3696407	3696464	58	128.5	Null	XC3085	XC4364	XC3086

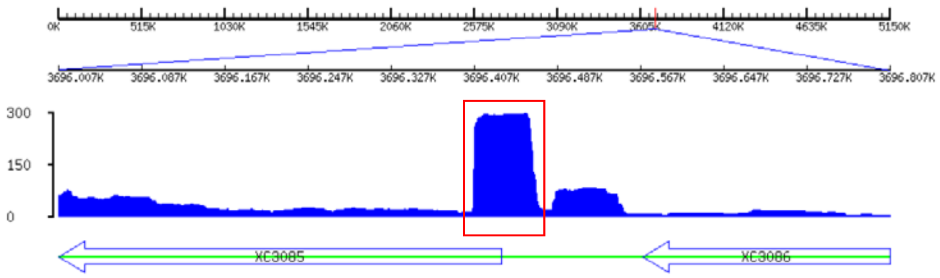

Transcript	Start	End	Length (bp)	RPKM	Inside gene	Overlap gene	Upstream gene	Downstream gene
TT462	3703180	3703535	356	1727	XC3090	Null	XC3089	XC3091

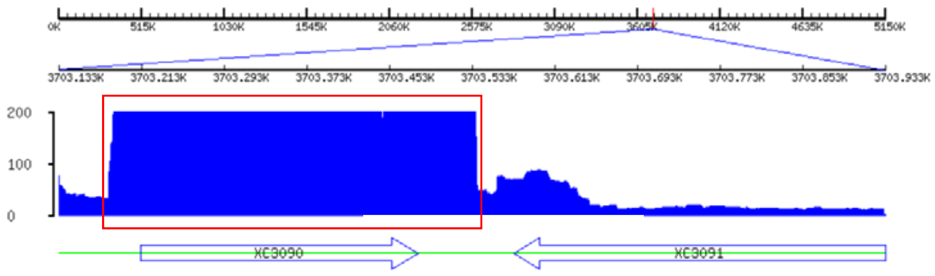

Transcript	Start	End	Length (bp)	RPKM	Inside gene	Overlap gene	Upstream gene	Downstream gene
TT463	3710523	3710605	83	38.7	XC3097	Null	XC3096	XC3098

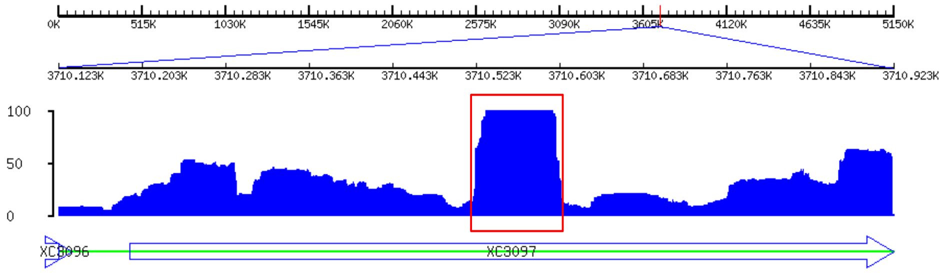

Transcript	Start	End	Length (bp)	RPKM	Inside gene	Overlap gene	Upstream gene	Downstream gene
TT464	3711936	3712009	74	490.7	Null	Null	XC3098	XC3099

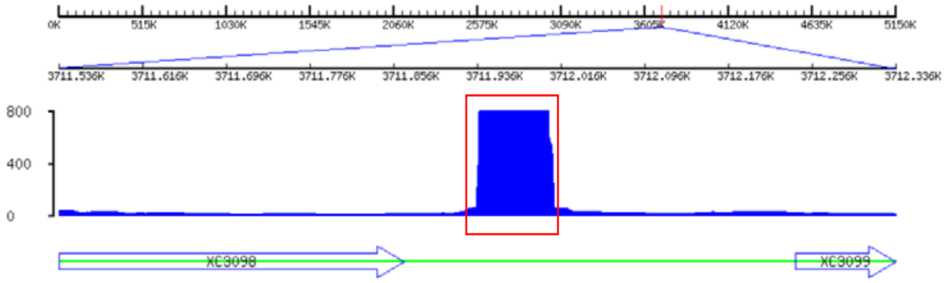

Transcript	Start	End	Length (bp)	RPKM	Inside gene	Overlap gene	Upstream gene	Downstream gene
TT465	3713524	3713638	114	1456.1	Null	XC3100	XC3099	XC3101
TT466	3713657	3713749	92	1404.7	XC3100	Null	XC3099	XC3101

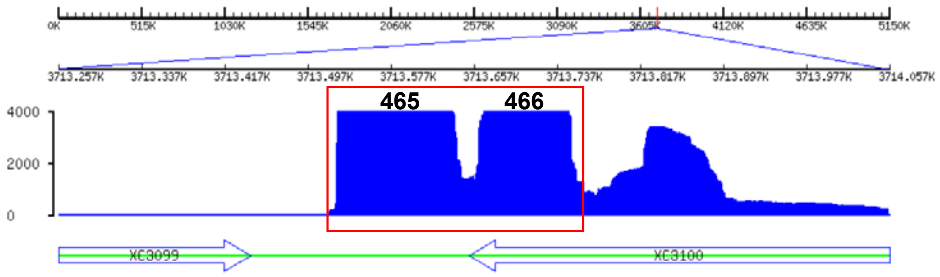

Transcript	Start	End	Length (bp)	RPKM	Inside gene	Overlap gene	Upstream gene	Downstream gene
TT467	3714229	3714329	100	869.9	Null	XC3100	XC3099	XC3101

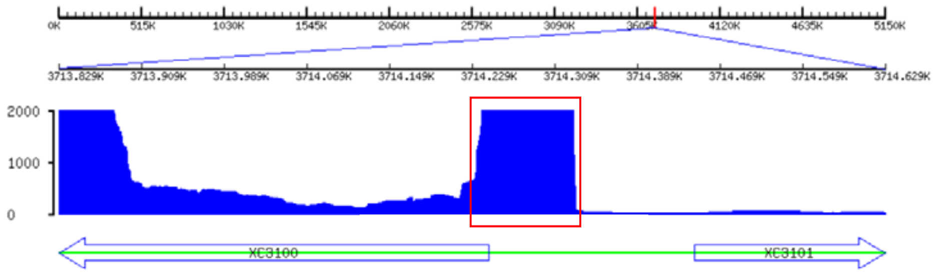

Transcript	Start	End	Length (bp)	RPKM	Inside gene	Overlap gene	Upstream gene	Downstream gene
TT468	3728975	3729058	84	136.2	XC3119	Null	XC3118	XC3121

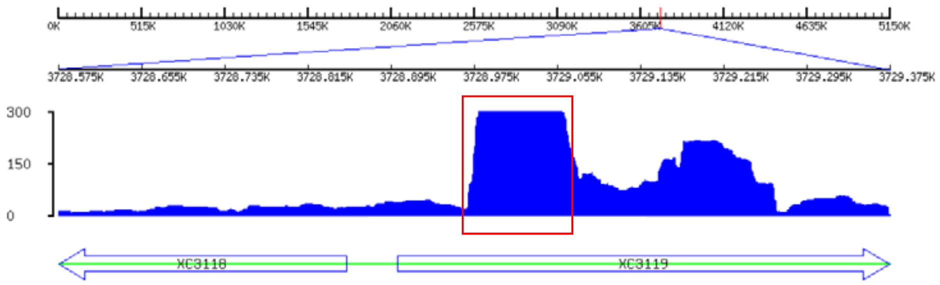

Transcript	Start	End	Length (bp)	RPKM	Inside gene	Overlap gene	Upstream gene	Downstream gene
TT469	3731165	3731244	80	53.3	Null	XC3122 XC3123	XC3121	XC3124
TT470	3731384	3731454	71	148.2	XC3123	Null	XC3122	XC3124

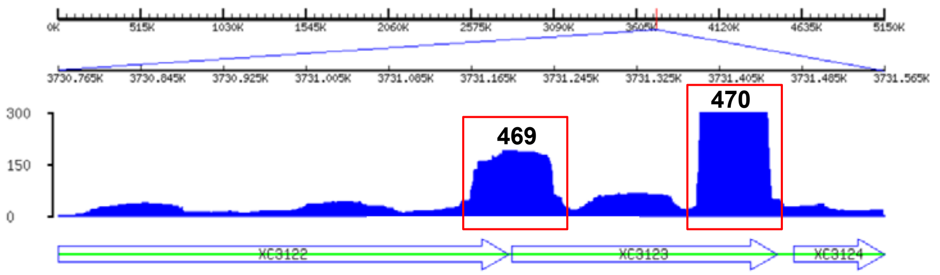

Transcript	Start	End	Length (bp)	RPKM	Inside gene	Overlap gene	Upstream gene	Downstream gene
TT471	3736036	3736086	51	349.9	Null	XC3128	XC3127	XC3129

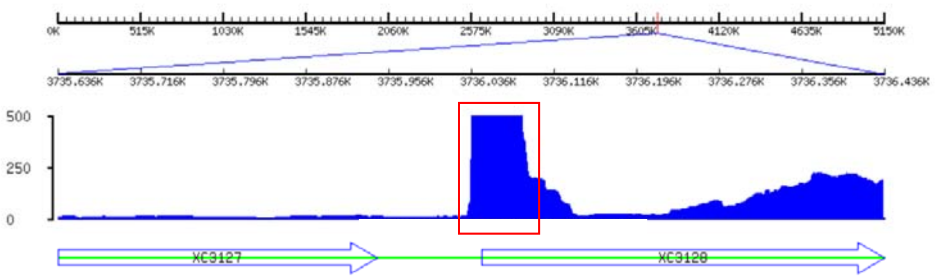

Transcript	Start	End	Length (bp)	RPKM	Inside gene	Overlap gene	Upstream gene	Downstream gene
TT472	3737338	3737417	80	133.3	XC3129	Null	XC3128	XC3130

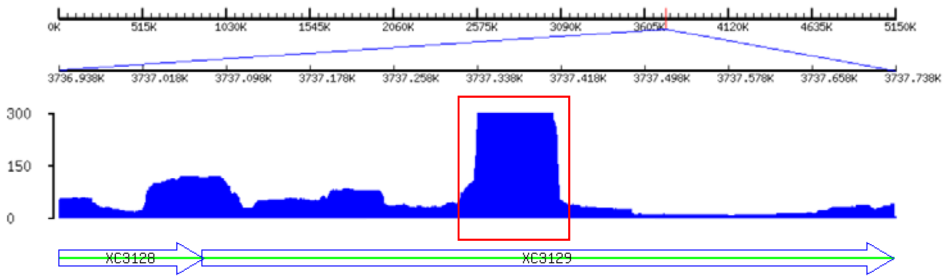

Transcript	Start	End	Length (bp)	RPKM	Inside gene	Overlap gene	Upstream gene	Downstream gene
TT473	3745231	3745408	177	261.5	Null	Null	XC3134	XC3135

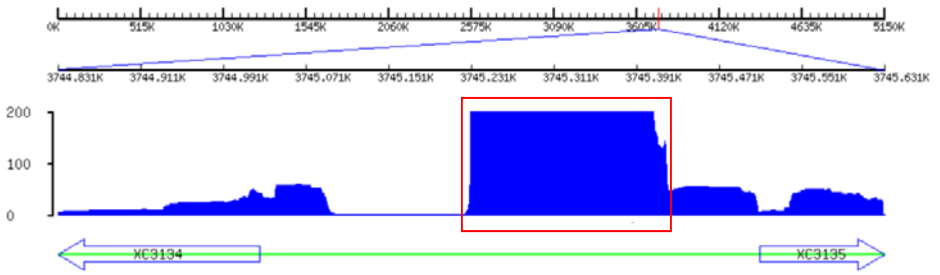

Transcript	Start	End	Length (bp)	RPKM	Inside gene	Overlap gene	Upstream gene	Downstream gene
TT474	3755519	3755604	86	77.9	Null	XC3142	XC3141	XC3143

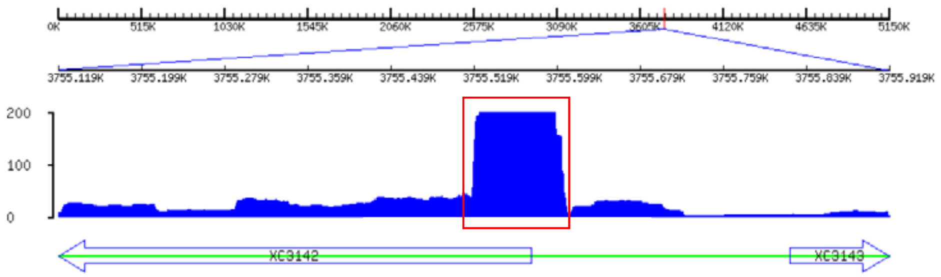

Transcript	Start	End	Length (bp)	RPKM	Inside gene	Overlap gene	Upstream gene	Downstream gene
TT475	3782877	3782977	101	551.2	XC3160	Null	XC3159	XC3161

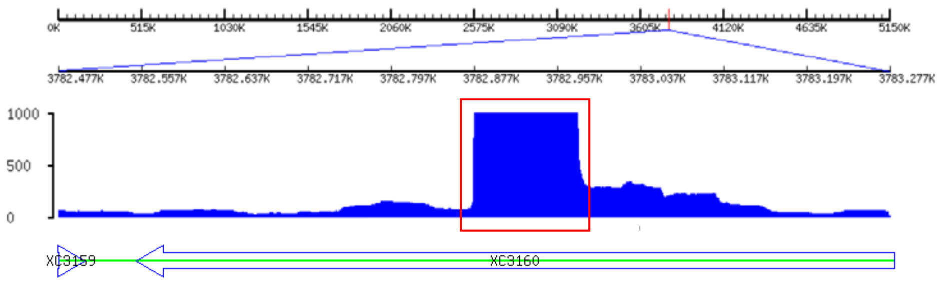

Transcript	Start	End	Length (bp)	RPKM	Inside gene	Overlap gene	Upstream gene	Downstream gene
TT476	3809409	3809576	168	1063.2	Null	Null	XC3179	XC3180

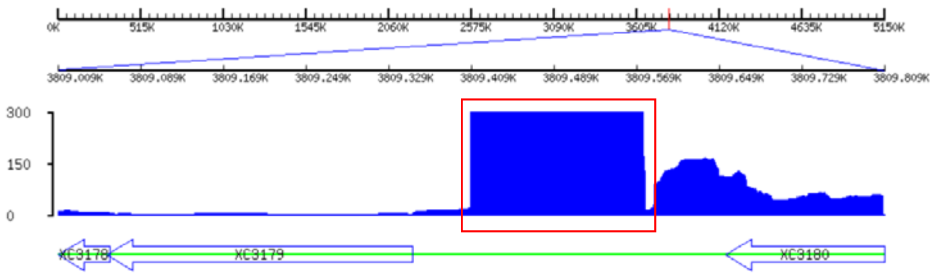

Transcript	Start	End	Length (bp)	RPKM	Inside gene	Overlap gene	Upstream gene	Downstream gene
TT477	3810025	3810150	126	100.2	XC3180	Null	XC3179	XC3181

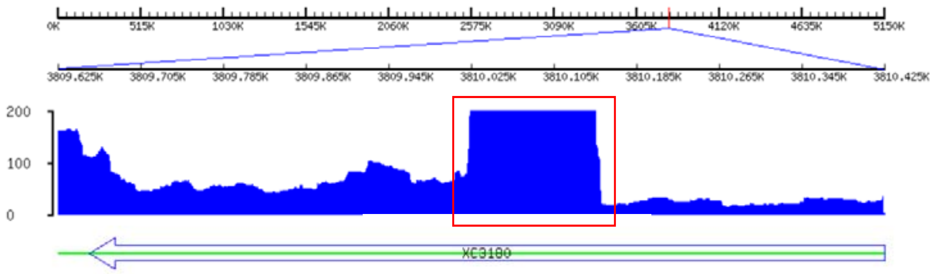

Transcript	Start	End	Length (bp)	RPKM	Inside gene	Overlap gene	Upstream gene	Downstream gene
TT478	3831134	3831207	74	333.5	Null	XC3196	XC3195	XC3197

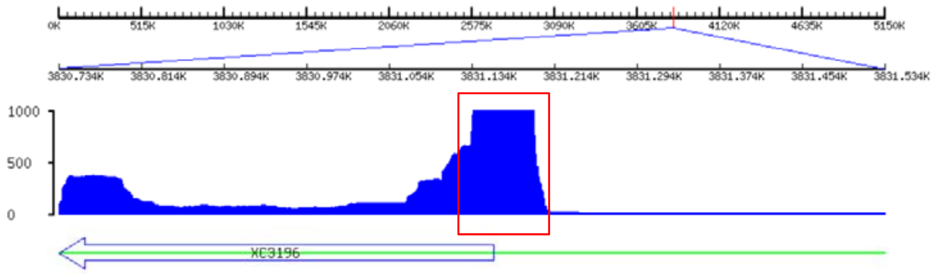

Transcript	Start	End	Length (bp)	RPKM	Inside gene	Overlap gene	Upstream gene	Downstream gene
TT479	3831853	3831910	58	181.6	Null	Null	XC3196	XC3197

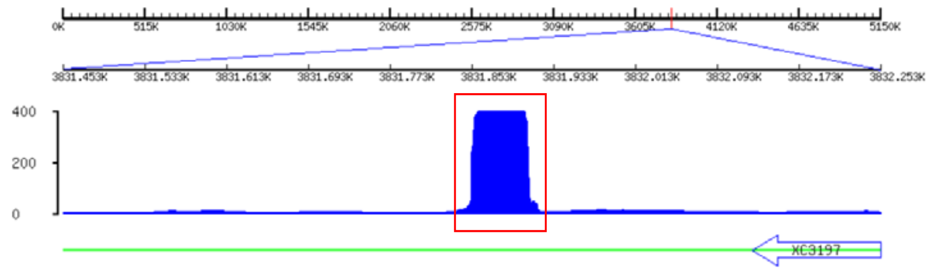

Transcript	Start	End	Length (bp)	RPKM	Inside gene	Overlap gene	Upstream gene	Downstream gene
TT480	3850534	3850594	61	169.5	Null	Null	XC3207	XC3208

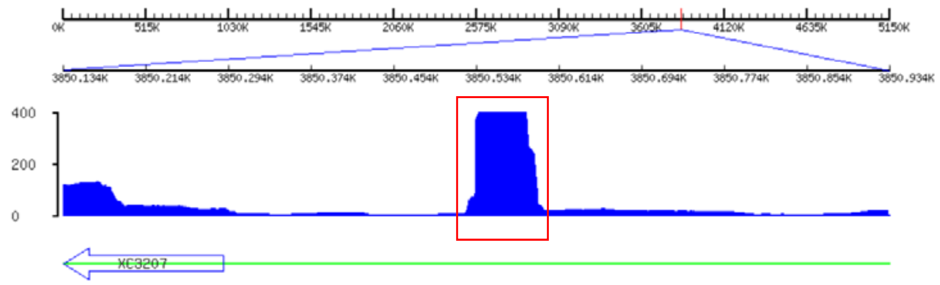

Transcript	Start	End	Length (bp)	RPKM	Inside gene	Overlap gene	Upstream gene	Downstream gene
TT481	3866289	3866358	70	853.8	XC4365	Null	XC3220	XC3221

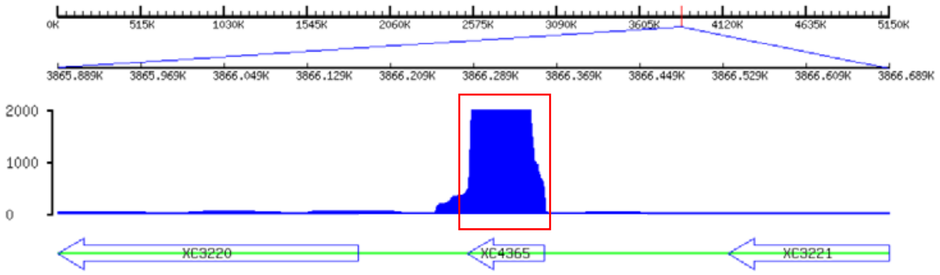

Transcript	Start	End	Length (bp)	RPKM	Inside gene	Overlap gene	Upstream gene	Downstream gene
TT482	3872958	3873047	90	53.6	Null	XC3227	XC3226	XC3228

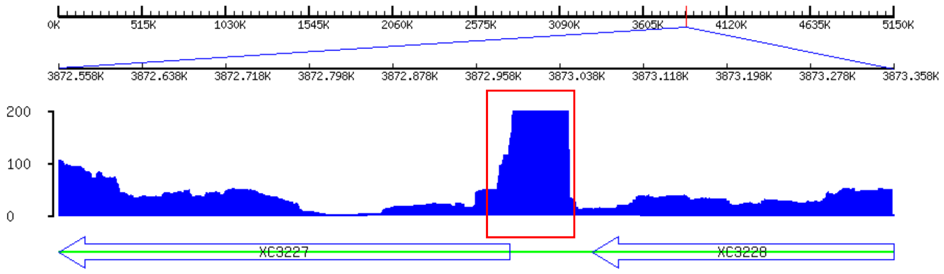

Transcript	Start	End	Length (bp)	RPKM	Inside gene	Overlap gene	Upstream gene	Downstream gene
TT483	3881882	3881942	61	347.5	Null	XC3236 /XC3237	XC3235	XC3238

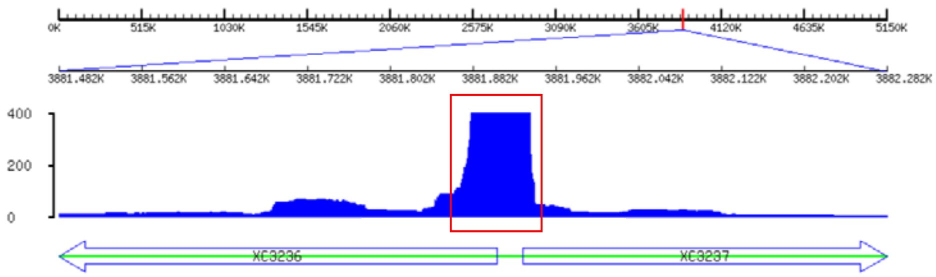

Transcript	Start	End	Length (bp)	RPKM	Inside gene	Overlap gene	Upstream gene	Downstream gene
TT484	3890025	3890130	106	5833.3	Null	Null	XC3244	XC4366
TT485	3890231	3890294	63	14395.5	XC4366	Null	XC3244	XC3245

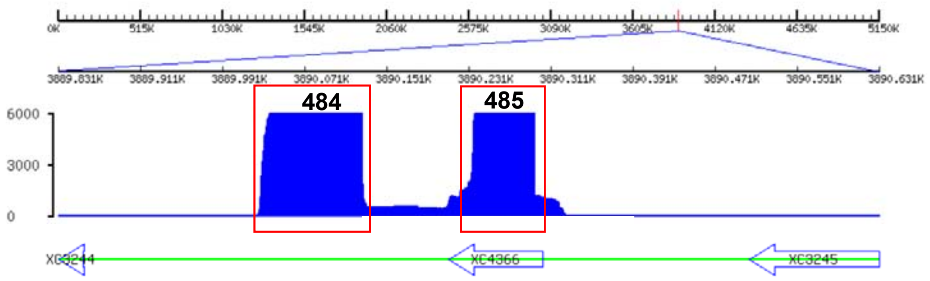

Transcript	Start	End	Length (bp)	RPKM	Inside gene	Overlap gene	Upstream gene	Downstream gene
TT486	3896282	3896544	263	2516.6	Null	XC4367	XC3253	XC3254

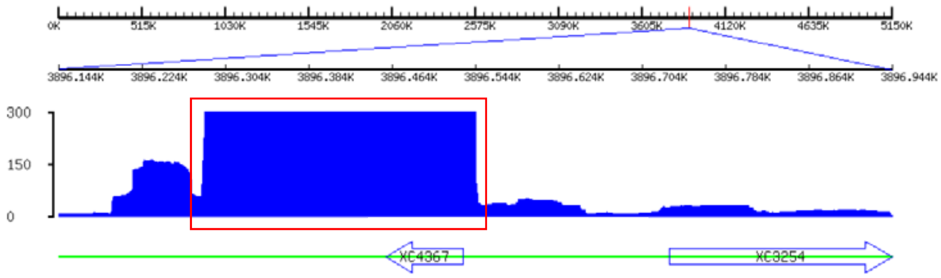

Transcript	Start	End	Length (bp)	RPKM	Inside gene	Overlap gene	Upstream gene	Downstream gene
TT487	3896462	3896525	63	8148.2	XC4367	Null	XC3253	XC3254

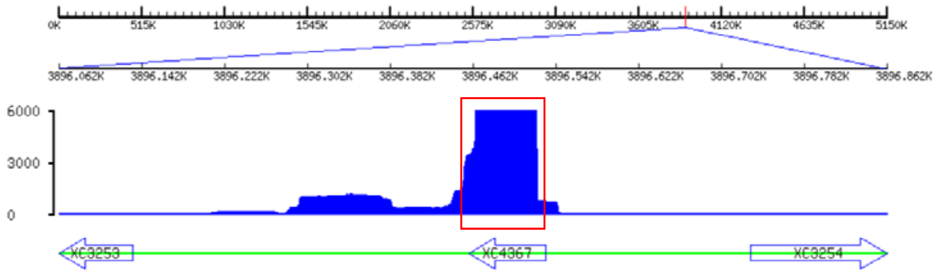

Transcript	Start	End	Length (bp)	RPKM	Inside gene	Overlap gene	Upstream gene	Downstream gene
TT488	3899791	3899900	109	158.6	XC3258	Null	XC3257	XC3259

Now ranger of the following image is from 3899231 bp **to** 3900030 bp , **Chromsome zoom value is** 1 : 1 , **Depth zoom value is 6 : 1**

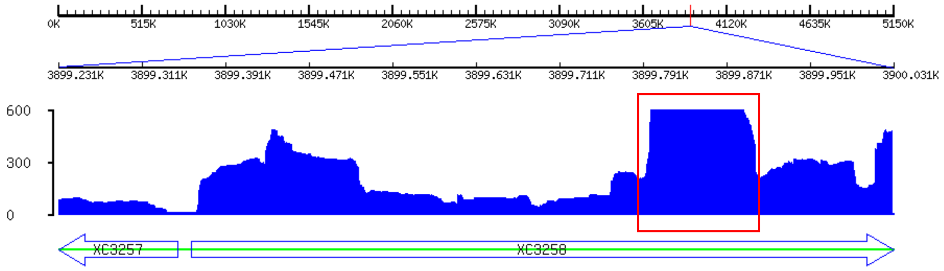

Transcript	Start	End	Length (bp)	RPKM	Inside gene	Overlap gene	Upstream gene	Downstream gene
TT489	3904927	2905249	323	303.1	XC4369	XC4368/XC4370	XC3261	XC3262

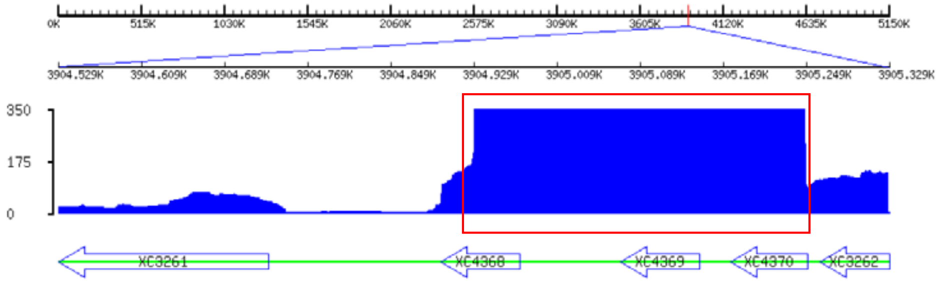

Transcript	Start	End	Length (bp)	RPKM	Inside gene	Overlap gene	Upstream gene	Downstream gene
TT490	3910473	3910593	121	516.5	XC3266	Null	XC3265	XC4371

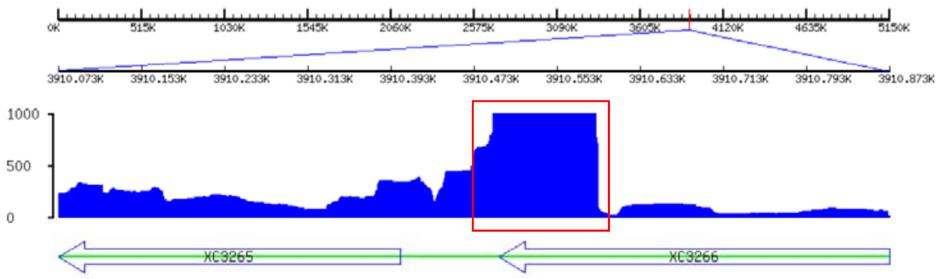

Transcript	Start	End	Length (bp)	RPKM	Inside gene	Overlap gene	Upstream gene	Downstream gene
TT491	3912154	3912219	66	9800.2	XC4372	Null	XC4371	XC4373
TT492	3912313	3912380	68	3377.2	XC4373	Null	XC4372	XC4374

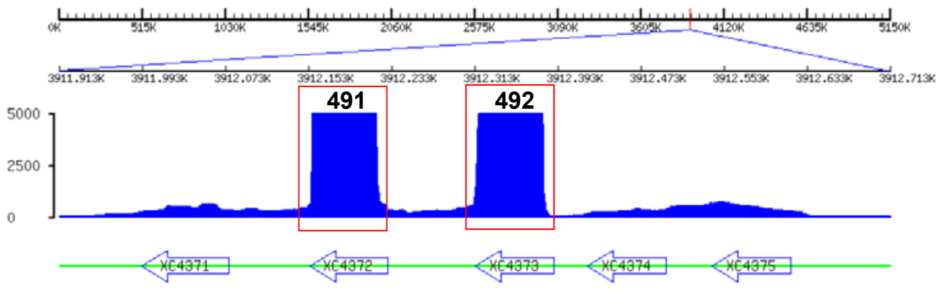

Transcript	Start	End	Length (bp)	RPKM	Inside gene	Overlap gene	Upstream gene	Downstream gene
TT493	3914010	3914101	92	232.5	XC3268	Null	XC3267	XC3269

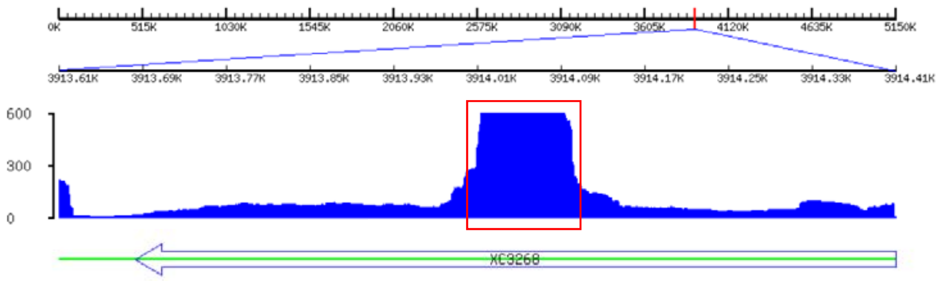

Transcript	Start	End	Length (bp)	RPKM	Inside gene	Overlap gene	Upstream gene	Downstream gene
TT494	3915254	3915308	55	160.3	XC3269	Null	XC3268	XC3270

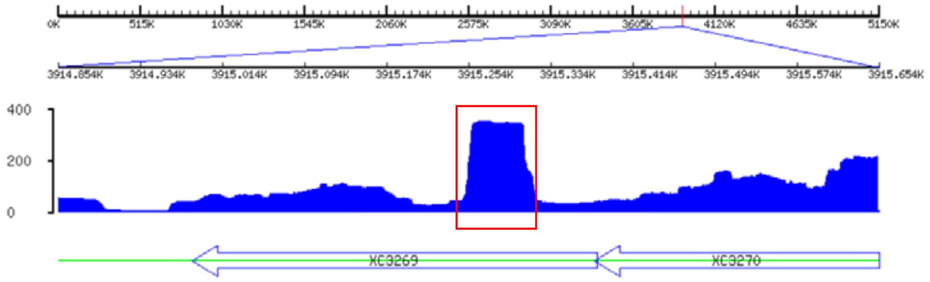

Transcript	Start	End	Length (bp)	RPKM	Inside gene	Overlap gene	Upstream gene	Downstream gene
TT495	3915732	3915793	62	321.2	XC3270	Null	XC3269	XC3271

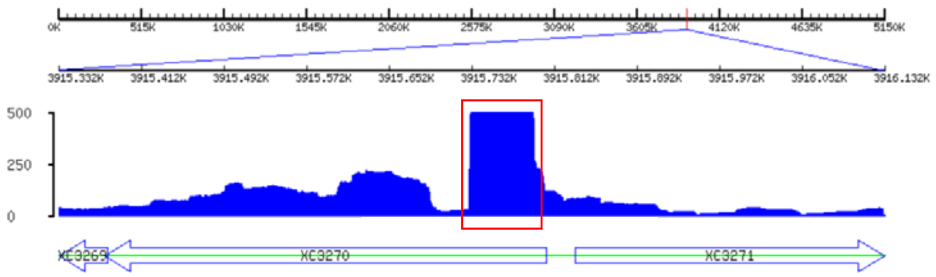

Transcript	Start	End	Length (bp)	RPKM	Inside gene	Overlap gene	Upstream gene	Downstream gene
TT496	3731672	3731762	91	40.3	XC3283	Null	XC3282	XC3284
TT497	3931796	3931888	92	138.1	Null	XC3283	XC3282	XC3284

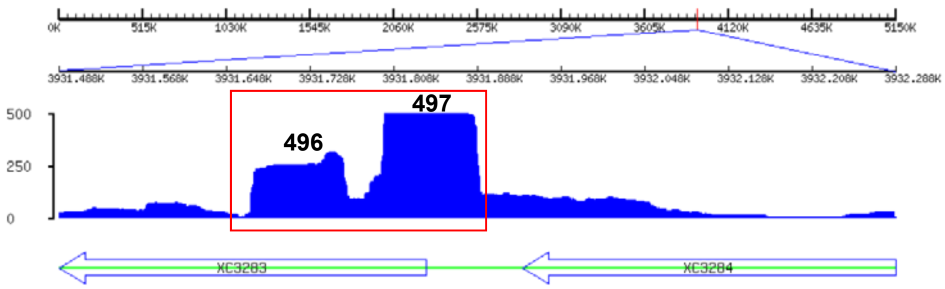

Transcript	Start	End	Length (bp)	RPKM	Inside gene	Overlap gene	Upstream gene	Downstream gene
TT498	3942597	3942687	91	115.2	XC3290	Null	XC3289	XC3291

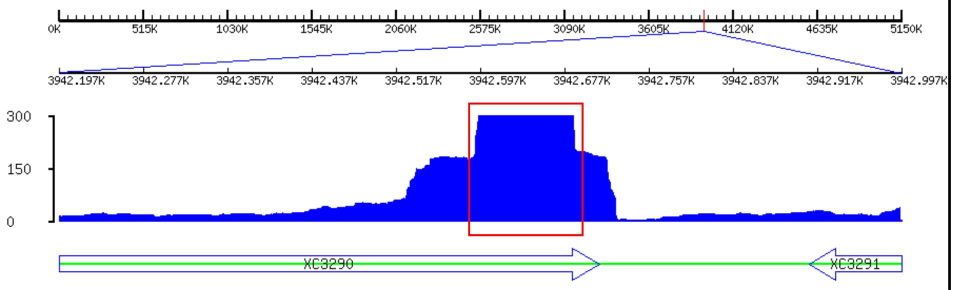

Transcript	Start	End	Length (bp)	RPKM	Inside gene	Overlap gene	Upstream gene	Downstream gene
TT499	3946746	3946806	61	598.8	Null	XC3294 XC3295	XC3293	XC3296

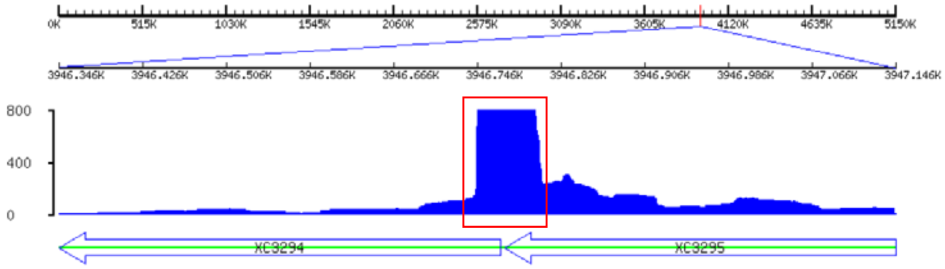

Transcript	Start	End	Length (bp)	RPKM	Inside gene	Overlap gene	Upstream gene	Downstream gene
TT500	3951120	3951214	95	810.8	XC3300	Null	XC3299	XC3301

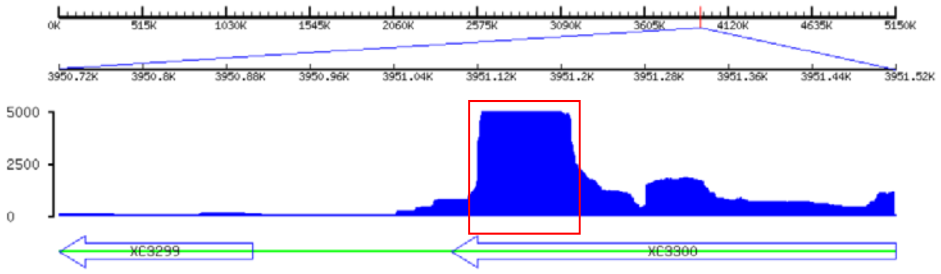

Transcript	Start	End	Length (bp)	RPKM	Inside gene	Overlap gene	Upstream gene	Downstream gene
TT501	3952166	3952255	90	953.2	Null	XC3300	XC3299	XC3301
TT502	3952270	3952344	74	964.4	Null	Null	XC3330	XC3301

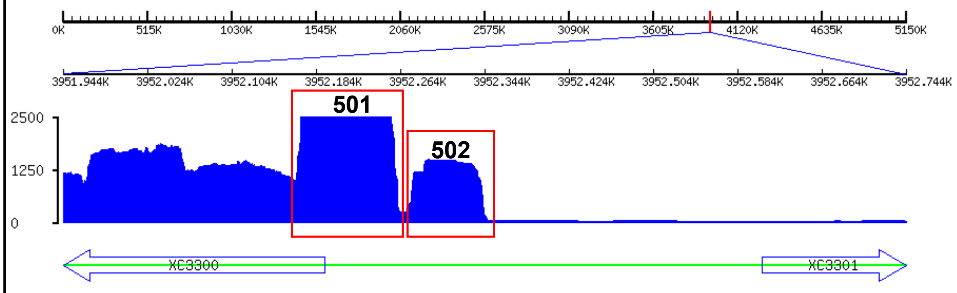

Transcript	Start	End	Length (bp)	RPKM	Inside gene	Overlap gene	Upstream gene	Downstream gene
TT503	3953842	3953969	128	615.9	Null	Null	XC3301	XC3302

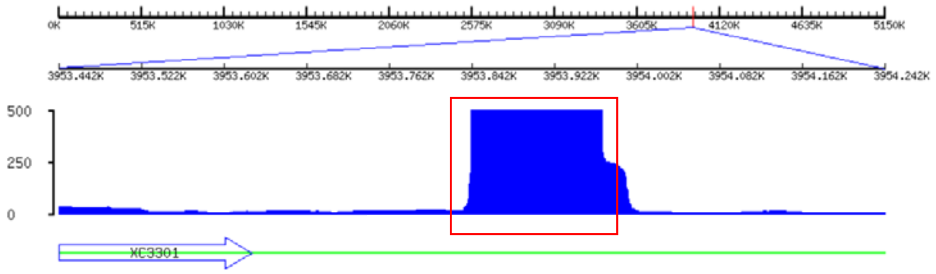

Transcript	Start	End	Length (bp)	RPKM	Inside gene	Overlap gene	Upstream gene	Downstream gene
TT504	3959367	3959450	84	320.9	Null	Null	XC3304	XC3305

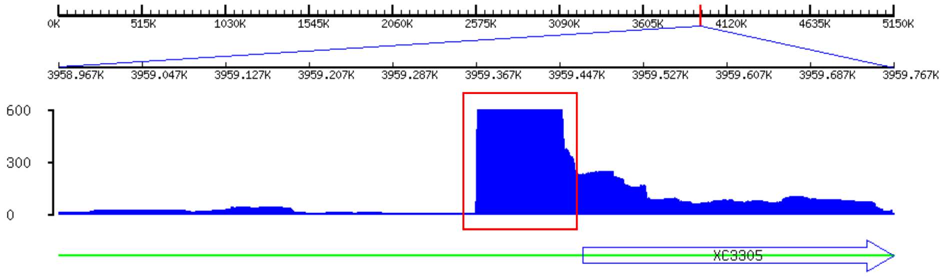

Transcript	Start	End	Length (bp)	RPKM	Inside gene	Overlap gene	Upstream gene	Downstream gene
TT505	3960669	3960726	58	68.4	Null	Null	XC3306	XC3307

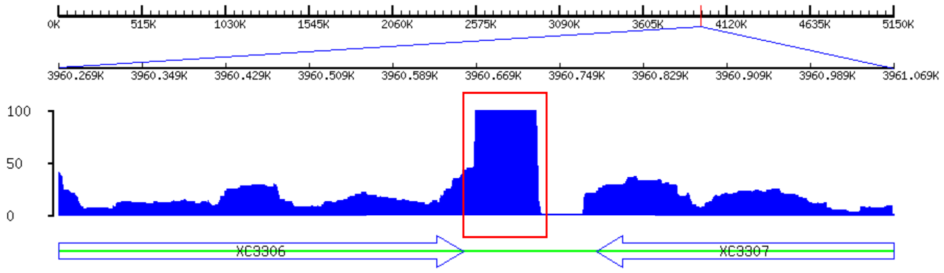

Transcript	Start	End	Length (bp)	RPKM	Inside gene	Overlap gene	Upstream gene	Downstream gene
TT506	3975385	3975452	68	210.9	Null	XC3320	XC3319	XC3321

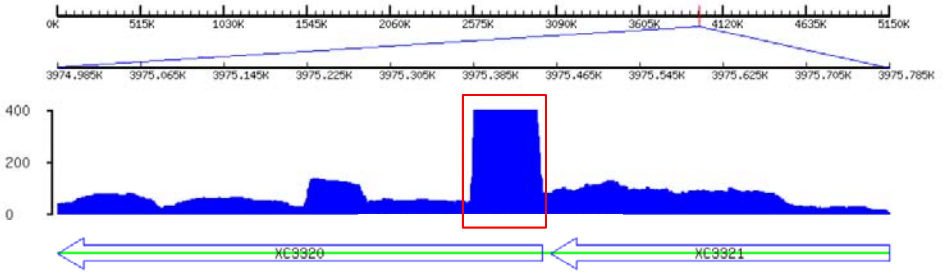

Transcript	Start	End	Length (bp)	RPKM	Inside gene	Overlap gene	Upstream gene	Downstream gene
TT507	3980310	3980391	82	127.5	XC3332	Null	XC3331	XC3333

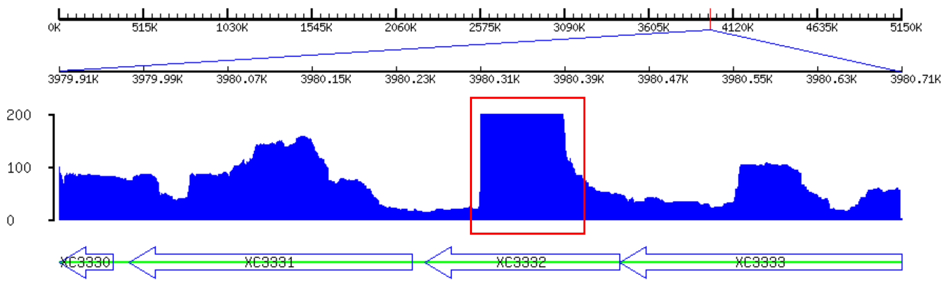

Transcript	Start	End	Length (bp)	RPKM	Inside gene	Overlap gene	Upstream gene	Downstream gene
TT508	3985177	3985256	80	516.2	Null	Null	XC3341	XC3342

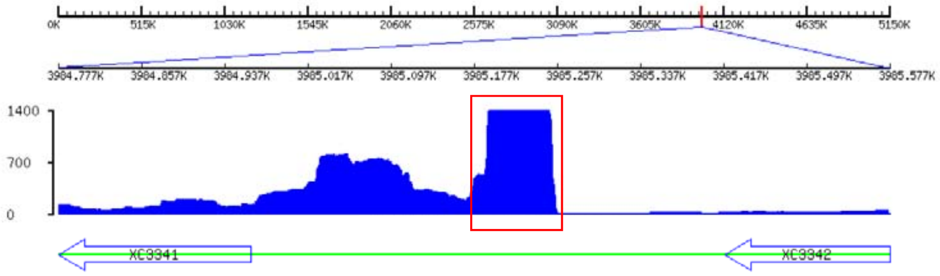

Transcript	Start	End	Length (bp)	RPKM	Inside gene	Overlap gene	Upstream gene	Downstream gene
TT509	3998705	3998872	167	234.6	Null	XC3348	XC3347	XC3349

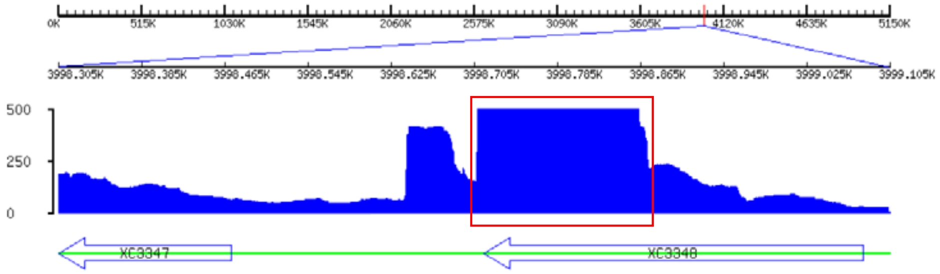

Transcript	Start	End	Length (bp)	RPKM	Inside gene	Overlap gene	Upstream gene	Downstream gene
TT510	4002507	4002557	51	112.5	Null	Null	XC3353	XC4376
TT511	4002575	4002641	67	172.4	XC4376	Null	XC3353	XC3354
TT512	4002749	4002839	91	97.5	Null	XC3354	XC4376	XC4377

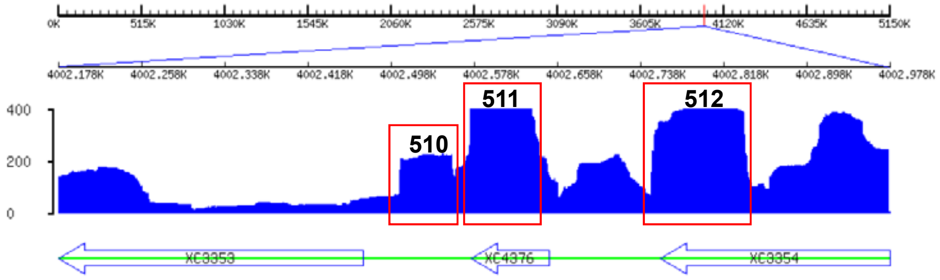

Transcript	Start	End	Length (bp)	RPKM	Inside gene	Overlap gene	Upstream gene	Downstream gene
TT513	4003949	4004046	98	354.4	Null	XC4377/XC4378	XC3354	XC4379
TT514	4004102	4004167	65	1468.2	XC4378	Null	XC4377	XC4379
TT515	4004215	4004327	112	470.9	Null	XC4378/XC4379	XC4377	XC3355

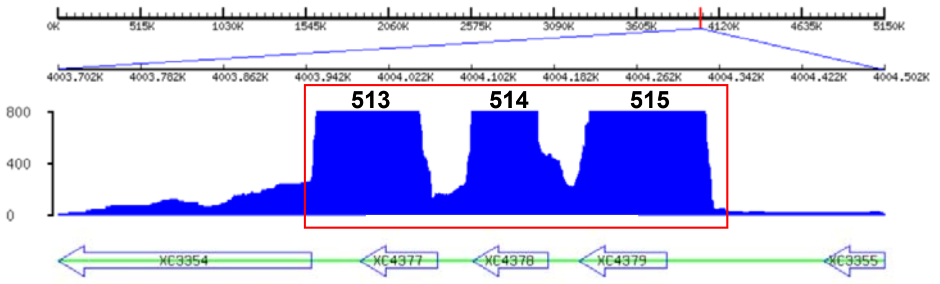

Transcript	Start	End	Length (bp)	RPKM	Inside gene	Overlap gene	Upstream gene	Downstream gene
TT516	4008125	4008194	69	8351.1	XC4380	Null	XC3358	XC3359

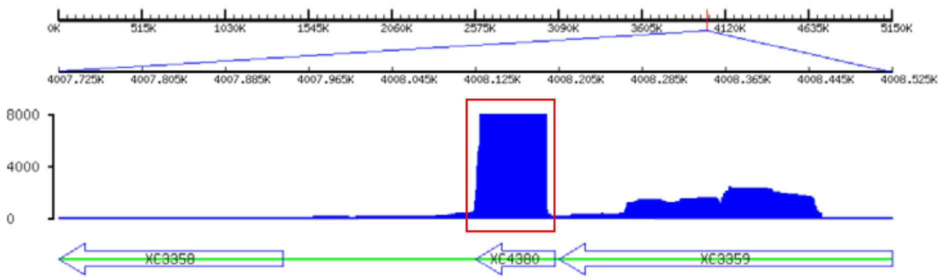

Transcript	Start	End	Length (bp)	RPKM	Inside gene	Overlap gene	Upstream gene	Downstream gene
TT517	4008216	4008458	242	341.5	XC3359	Null	XC4380	XC3360

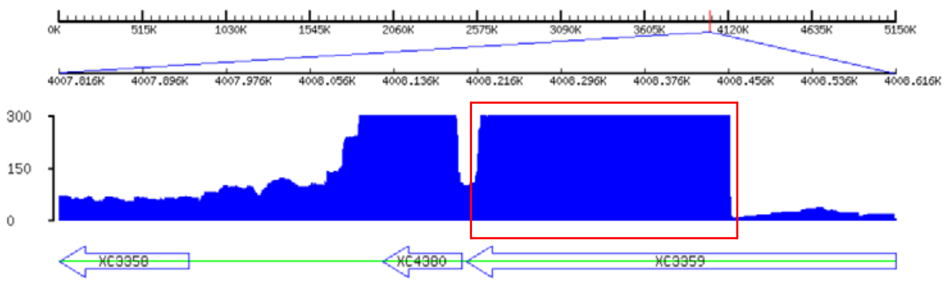

Transcript	Start	End	Length (bp)	RPKM	Inside gene	Overlap gene	Upstream gene	Downstream gene
TT518	4011324	4011438	115	62.2	Null	XC3361	XC3360	XC3362
TT519	4011451	4011543	93	184.7	Null	XC3362	XC3361	XC3363

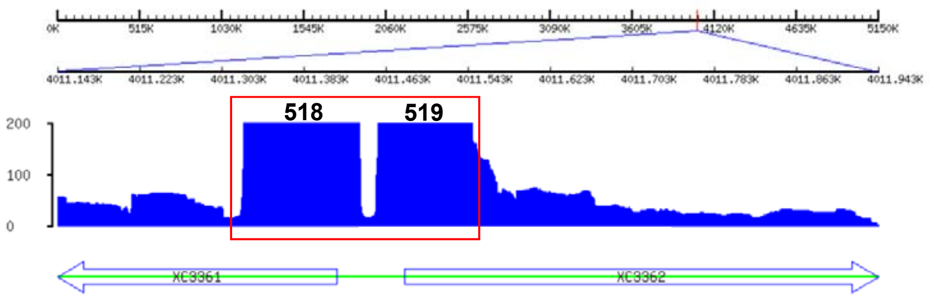

Transcript	Start	End	Length (bp)	RPKM	Inside gene	Overlap gene	Upstream gene	Downstream gene
TT520	4029376	4029439	64	458.5	XC3379	Null	XC3378	XC3380

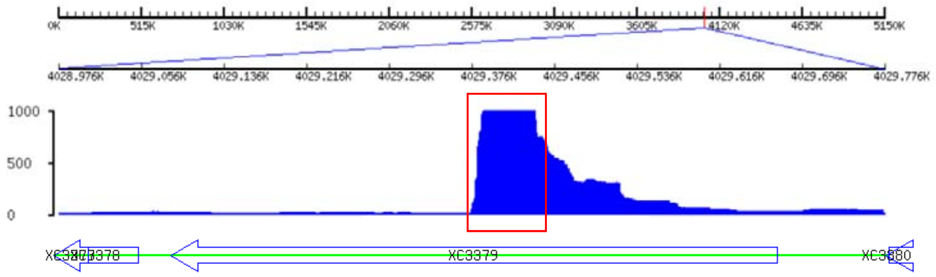

Transcript	Start	End	Length (bp)	RPKM	Inside gene	Overlap gene	Upstream gene	Downstream gene
TT521	4032184	4032259	76	99.1	XC3381	Null	XC3380	XC3382

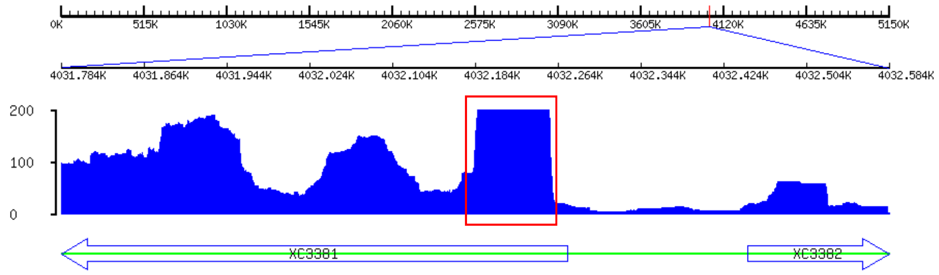

Transcript	Start	End	Length (bp)	RPKM	Inside gene	Overlap gene	Upstream gene	Downstream gene
TT522	4036140	4036208	69	97.9	XC3386	Null	XC3385	XC3387

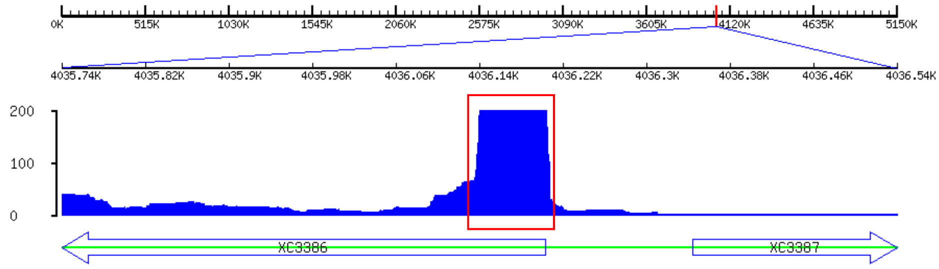

Transcript	Start	End	Length (bp)	RPKM	Inside gene	Overlap gene	Upstream gene	Downstream gene
TT523	4048247	4048323	77	218.6	XC3396	Null	XC3395	XC3397

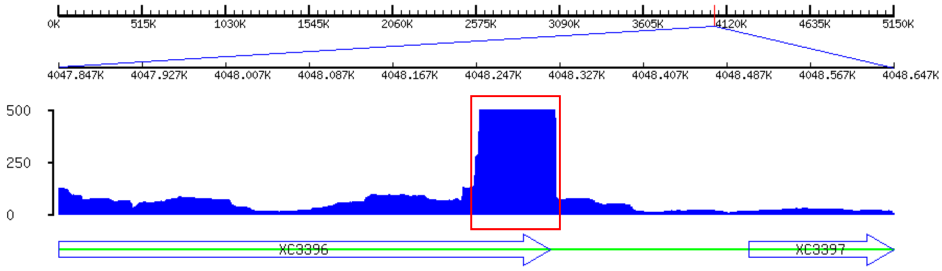

Transcript	Start	End	Length (bp)	RPKM	Inside gene	Overlap gene	Upstream gene	Downstream gene
TT524	4054079	4054210	132	152.7	Null	XC3402	XC3401	XC3403

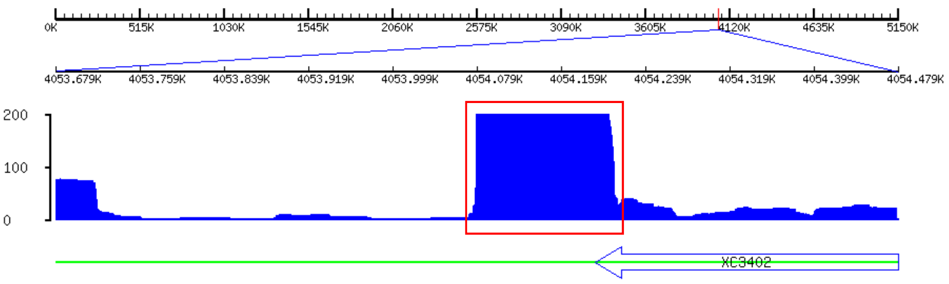

Transcript	Start	End	Length (bp)	RPKM	Inside gene	Overlap gene	Upstream gene	Downstream gene
TT525	4089013	4089106	93	59.6	XC3438	Null	XC3437	XC3439
TT526	4089148	4089234	87	67.5	XC3438	Null	XC3437	XC3439

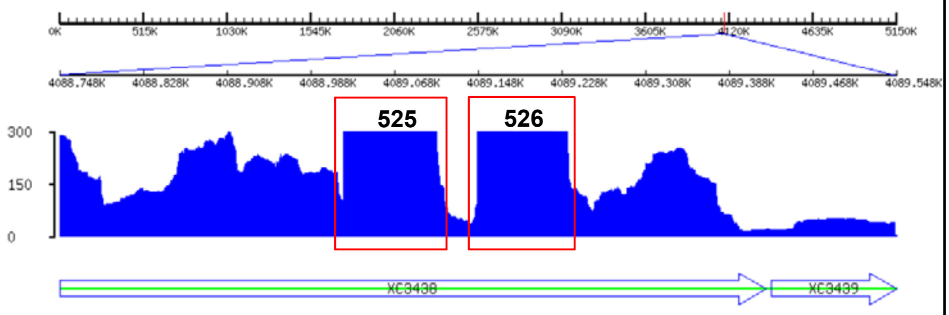

Transcript	Start	End	Length (bp)	RPKM	Inside gene	Overlap gene	Upstream gene	Downstream gene
TT527	4098603	4098678	76	27.9	Null	XC3450	XC3449	XC3451

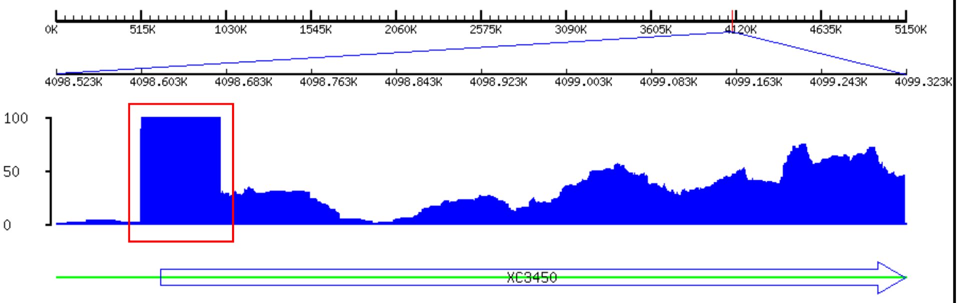

Transcript	Start	End	Length (bp)	RPKM	Inside gene	Overlap gene	Upstream gene	Downstream gene
TT528	4101774	4101839	66	547.8	XC3452	Null	XC3451	XC3453

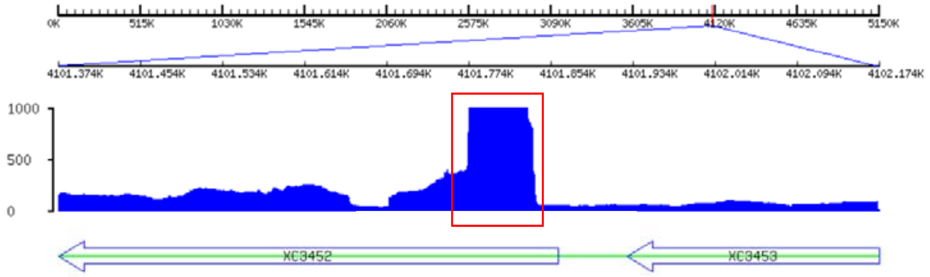

Transcript	Start	End	Length (bp)	RPKM	Inside gene	Overlap gene	Upstream gene	Downstream gene
TT529	4105917	4106041	125	300.6	Null	XC3456	XC3455	XC3457
TT530	4106078	4106161	113	374.3	XC3456	Null	XC3455	XC3457

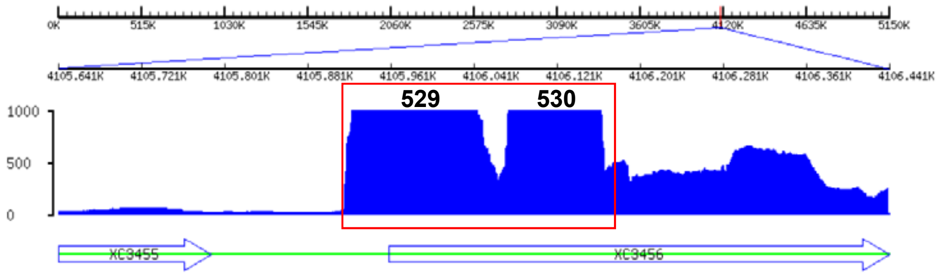

Transcript	Start	End	Length (bp)	RPKM	Inside gene	Overlap gene	Upstream gene	Downstream gene
TT531	4114612	4114817	205	243.4	XC3464	Null	XC3463	XC3465

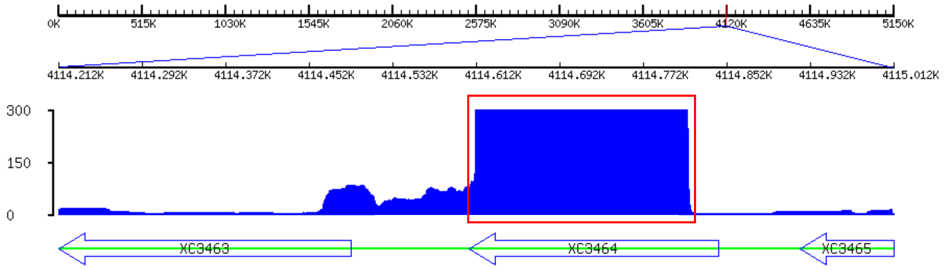

Transcript	Start	End	Length (bp)	RPKM	Inside gene	Overlap gene	Upstream gene	Downstream gene
TT532	4122300	4122374	75	447.4	Null	XC3471	XC3470	XC3472

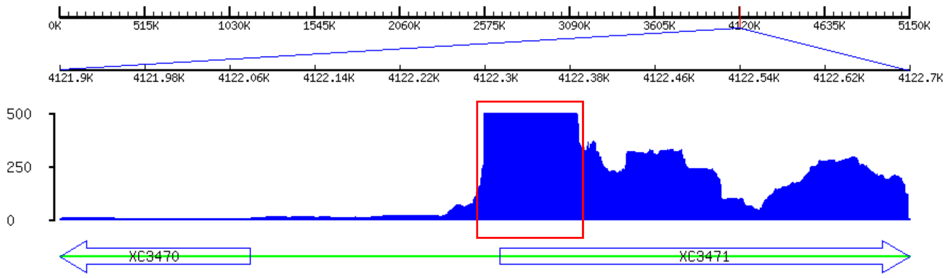

Transcript	Start	End	Length (bp)	RPKM	Inside gene	Overlap gene	Upstream gene	Downstream gene
TT533	4137310	4137368	59	122.6	Null	XC3479	XC3478	XC3481

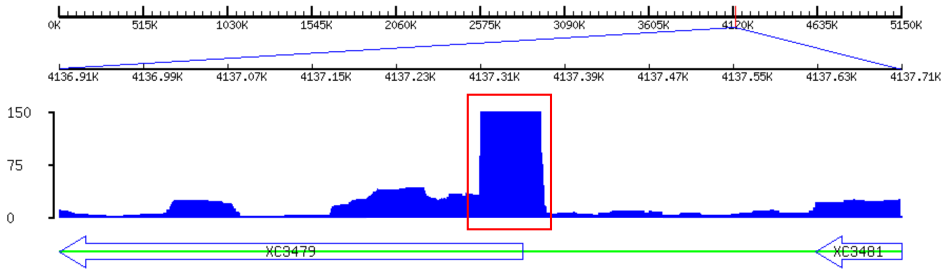

Transcript	Start	End	Length (bp)	RPKM	Inside gene	Overlap gene	Upstream gene	Downstream gene
TT534	4140268	4140451	183	413.3	Null	XC3482	XC3481	XC3483

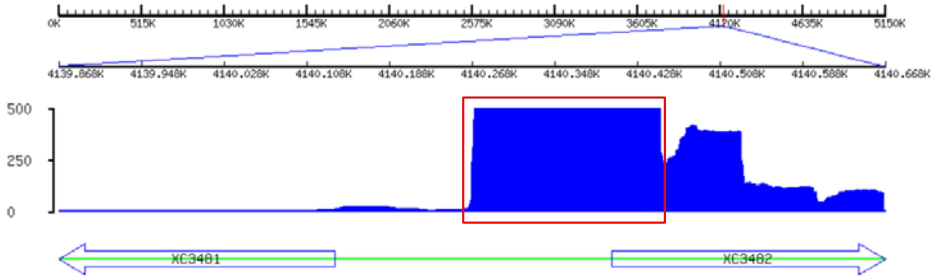

Transcript	Start	End	Length (bp)	RPKM	Inside gene	Overlap gene	Upstream gene	Downstream gene
TT535	4142107	4142181	75	50.7	Null	Null	XC3482	XC3483

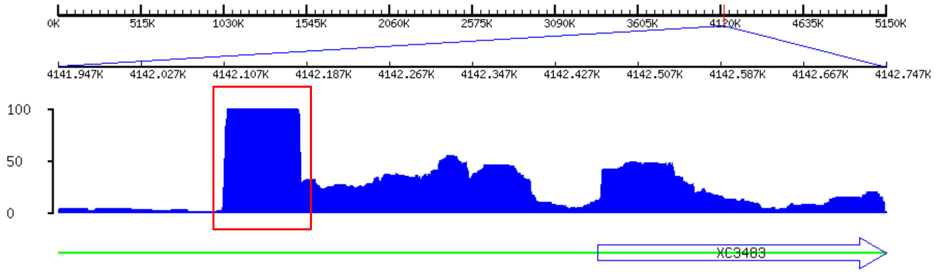

Transcript	Start	End	Length (bp)	RPKM	Inside gene	Overlap gene	Upstream gene	Downstream gene
TT536	4153845	4153937	93	147.3	Null	XC3496	XC3495	XC3497

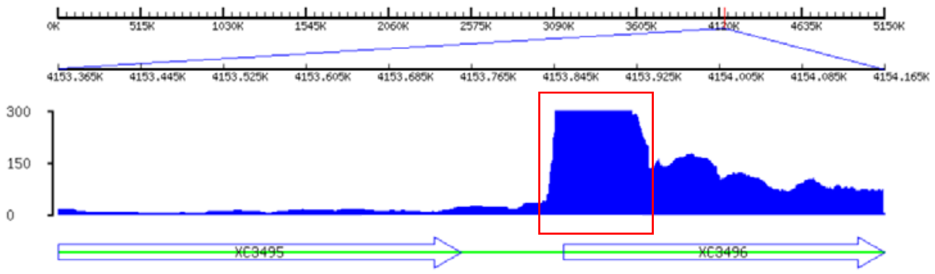

Transcript	Start	End	Length (bp)	RPKM	Inside gene	Overlap gene	Upstream gene	Downstream gene
TT537	4154212	4154341	130	100.2	XC3496	Null	XC3495	XC3497

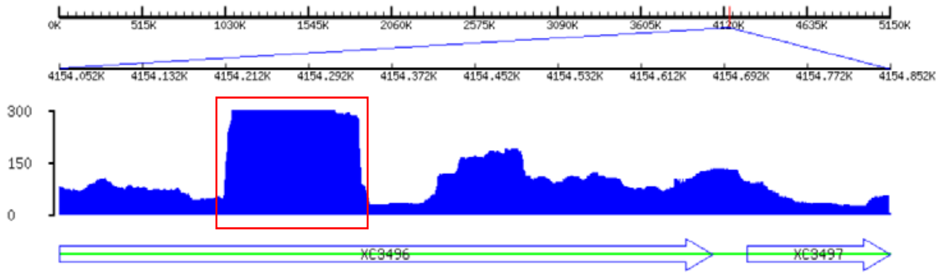

Transcript	Start	End	Length (bp)	RPKM	Inside gene	Overlap gene	Upstream gene	Downstream gene
TT538	4162147	4162217	71	389.2	XC3503	Null	XC3502	XC3504
TT539	4162383	4162445	63	89.8	XC3503	Null	XC3502	XC3504

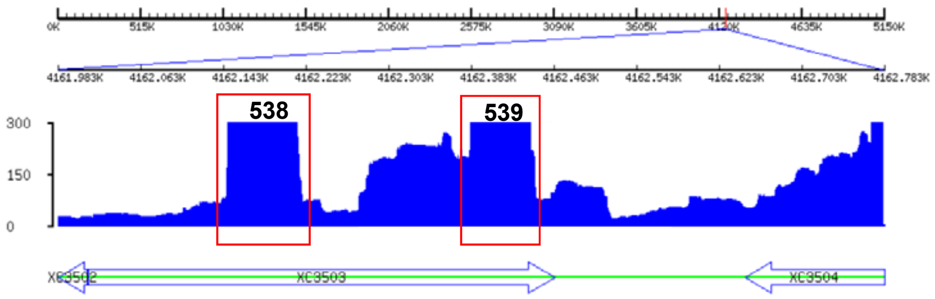

Transcript	Start	End	Length (bp)	RPKM	Inside gene	Overlap gene	Upstream gene	Downstream gene
TT540	4162878	4162954	77	211.9	XC3504	Null	XC3503	XC3505

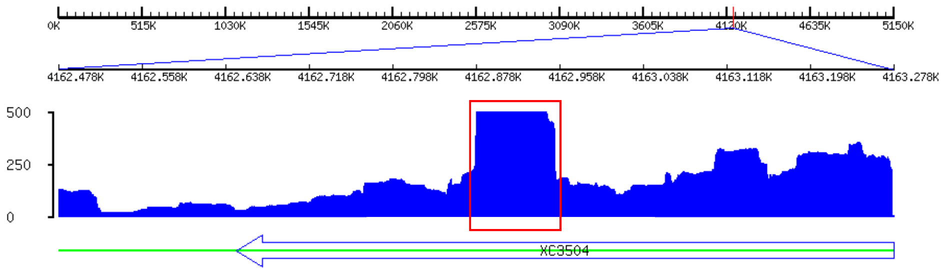

Transcript	Start	End	Length (bp)	RPKM	Inside gene	Overlap gene	Upstream gene	Downstream gene
TT541	4165010	4165095	86	76.7	Null	XC3505	XC3504	XC3506

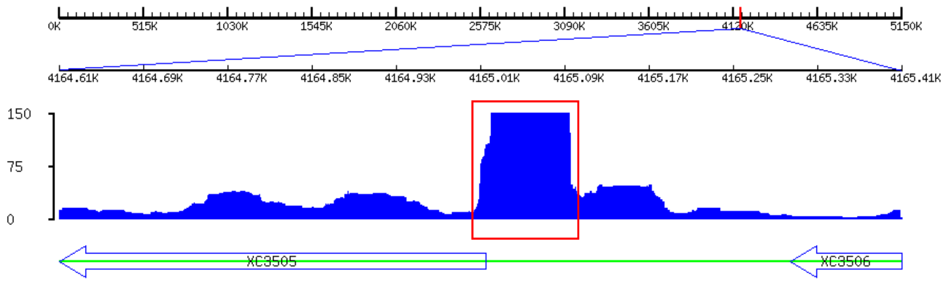

Transcript	Start	End	Length (bp)	RPKM	Inside gene	Overlap gene	Upstream gene	Downstream gene
TT542	4177501	4177573	73	46.9	XC3515	Null	XC3514	XC3516

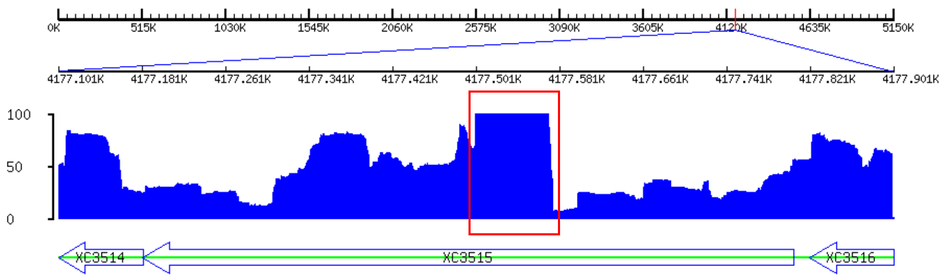

Transcript	Start	End	Length (bp)	RPKM	Inside gene	Overlap gene	Upstream gene	Downstream gene
TT543	4180020	4180304	285	86.6	Null	XC3518	XC3517	XC3519

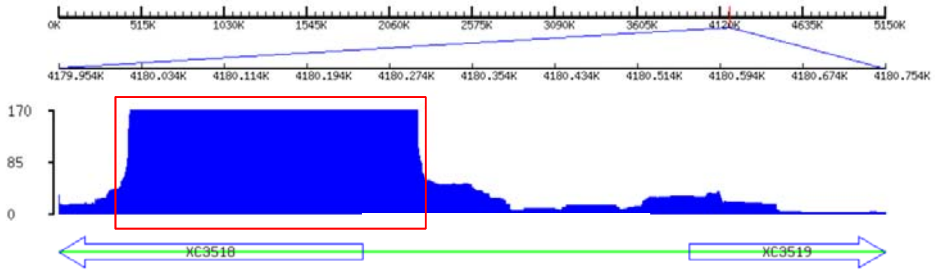

Transcript	Start	End	Length (bp)	RPKM	Inside gene	Overlap gene	Upstream gene	Downstream gene
TT544	4182260	4182605	345	20292.1	misc_RNA	Null	XC3521	XC3522

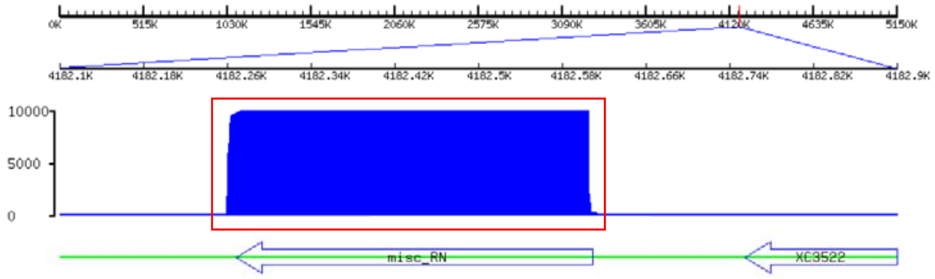

Transcript	Start	End	Length (bp)	RPKM	Inside gene	Overlap gene	Upstream gene	Downstream gene
TT545	4203279	4203464	186	307.1	Null	Null	XC3539	XC3540
TT546	4203604	4203672	69	76.9	Null	XC3540	XC3539	XC3541

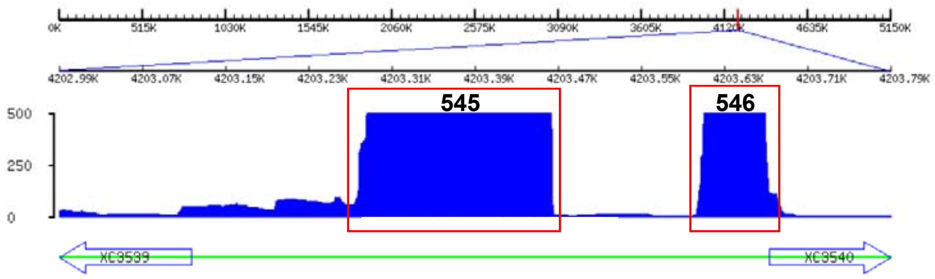

Transcript	Start	End	Length (bp)	RPKM	Inside gene	Overlap gene	Upstream gene	Downstream gene
TT547	4207781	4207841	61	216.2	Null	XC3544	XC3543	XC3545

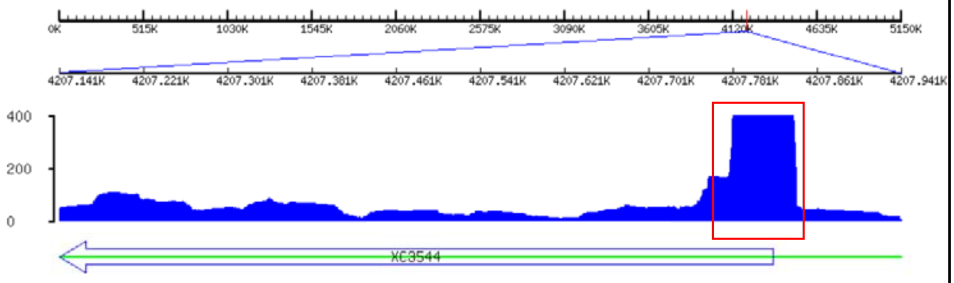

Transcript	Start	End	Length (bp)	RPKM	Inside gene	Overlap gene	Upstream gene	Downstream gene
TT548	4207977	4208207	231	591.7	Null	XC3545	XC3544	XC3546

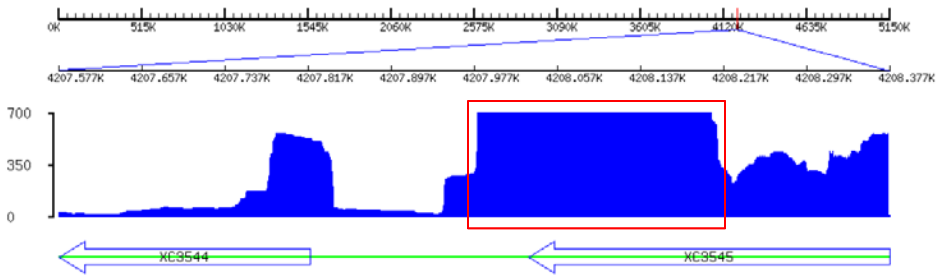

Transcript	Start	End	Length (bp)	RPKM	Inside gene	Overlap gene	Upstream gene	Downstream gene
TT549	4208448	4208572	125	1910.7	Null	XC3545	XC3544	XC3546

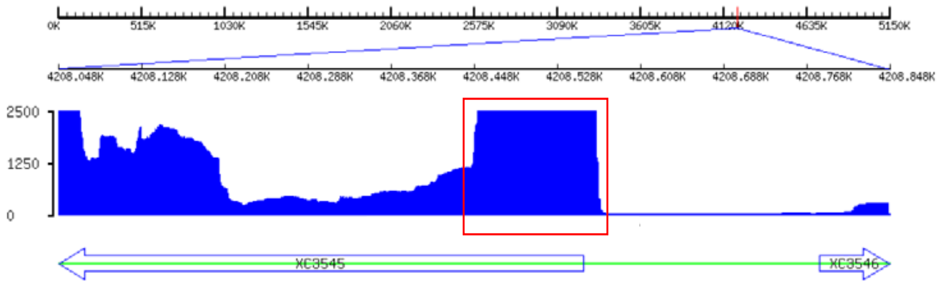

Transcript	Start	End	Length (bp)	RPKM	Inside gene	Overlap gene	Upstream gene	Downstream gene
TT550	4208814	4208875	62	106.3	XC3546	Null	XC3545	XC3547

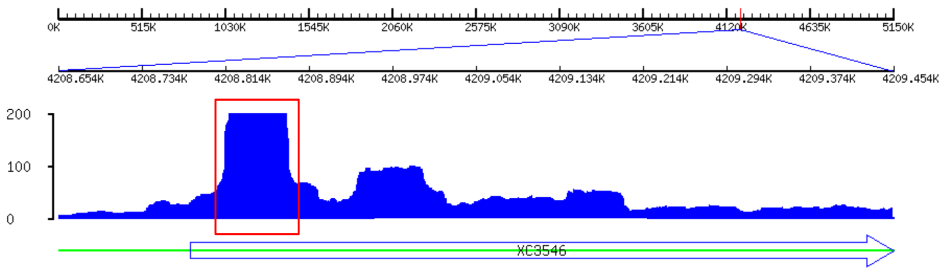

Transcript	Start	End	Length (bp)	RPKM	Inside gene	Overlap gene	Upstream gene	Downstream gene
TT551	4223588	4223655	68	83.9	Null	Null	XC3557	XC3558

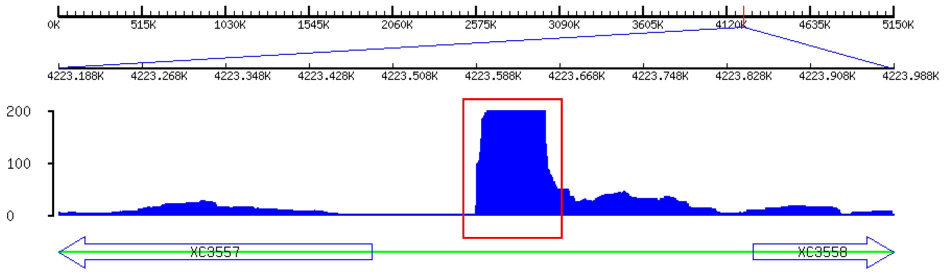

Transcript	Start	End	Length (bp)	RPKM	Inside gene	Overlap gene	Upstream gene	Downstream gene
TT552	4256839	4256912	74	340.1	XC3579	Null	XC3578	XC3580

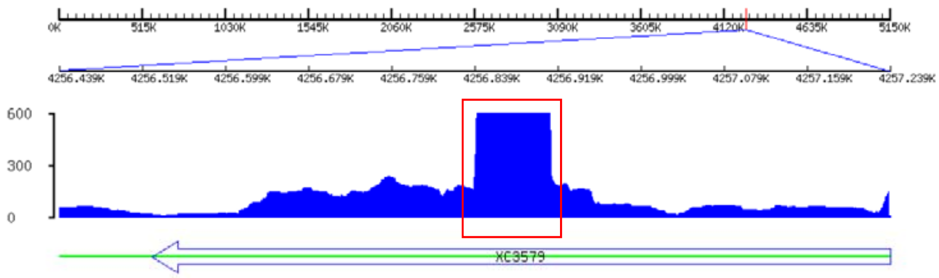

Transcript	Start	End	Length (bp)	RPKM	Inside gene	Overlap gene	Upstream gene	Downstream gene
TT553	4257233	4257332	100	56.5	Null	XC3579	XC3578	XC3580

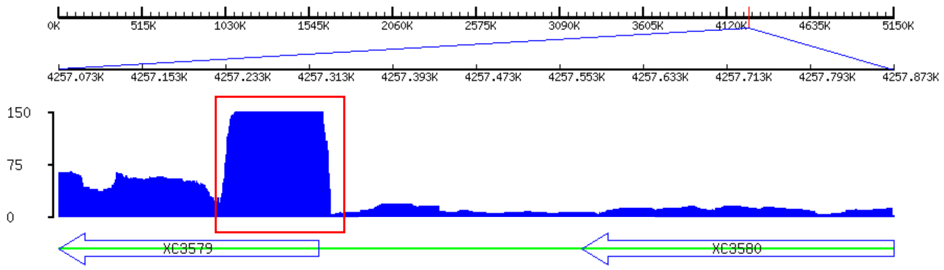

Transcript	Start	End	Length (bp)	RPKM	Inside gene	Overlap gene	Upstream gene	Downstream gene
TT554	4261827	4261905	73	487.2	Null	Null	XC3584	XC3585

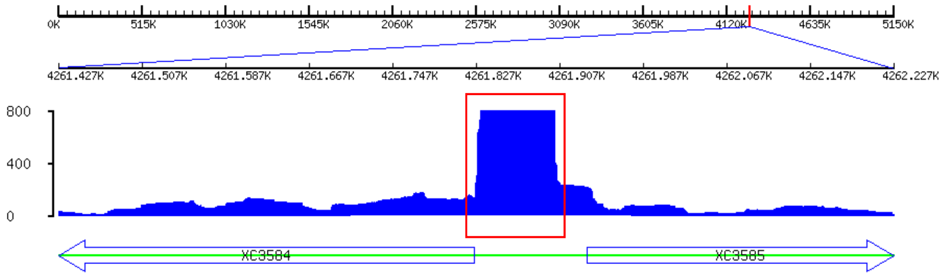

Transcript	Start	End	Length (bp)	RPKM	Inside gene	Overlap gene	Upstream gene	Downstream gene
TT555	4276767	4276830	64	725.7	Null	Null	XC3596	XC3597

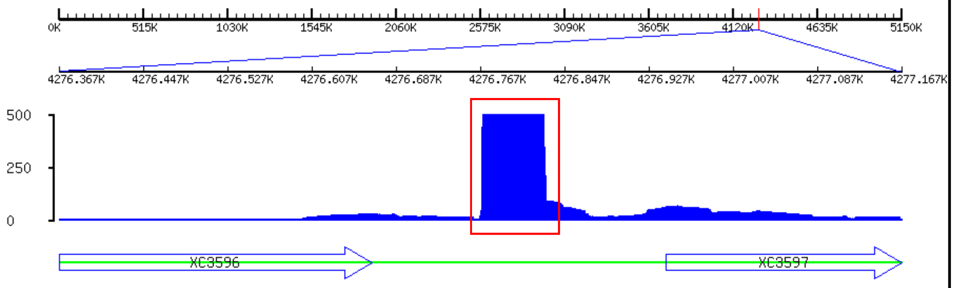

Transcript	Start	End	Length (bp)	RPKM	Inside gene	Overlap gene	Upstream gene	Downstream gene
TT556	4277484	4277545	62	167.7	XC3598	Null	XC3597	XC3599

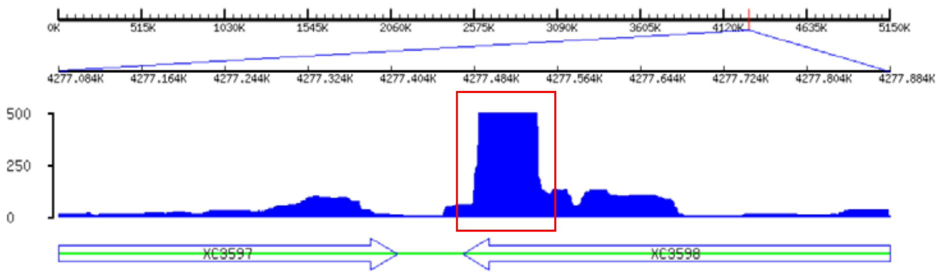

Transcript	Start	End	Length (bp)	RPKM	Inside gene	Overlap gene	Upstream gene	Downstream gene
TT557	4283435	4283495	61	9.5	XC3603	Null	XC3602	XC3604
TT558	4283660	4283955	296	56.3	Null	XC3604	XC3603	XC3605

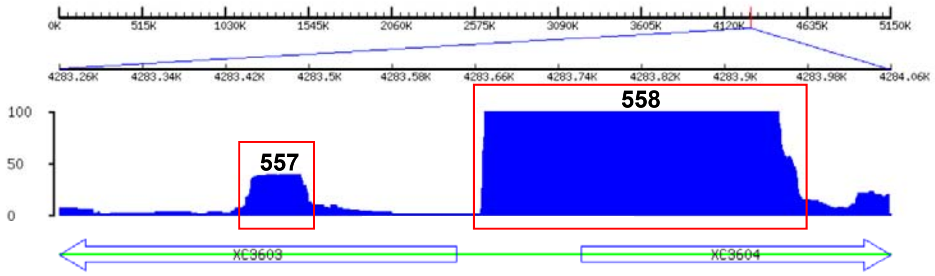

Transcript	Start	End	Length (bp)	RPKM	Inside gene	Overlap gene	Upstream gene	Downstream gene
TT559	4299726	4299807	92	82.3	XC3618	Null	XC3617	XC3619

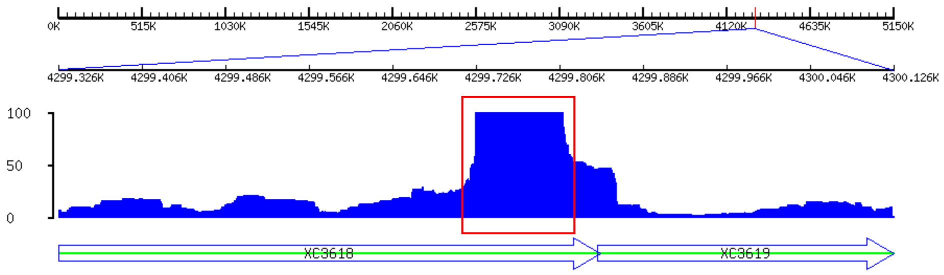

Transcript	Start	End	Length (bp)	RPKM	Inside gene	Overlap gene	Upstream gene	Downstream gene
TT560	4305698	4305751	54	71.5	Null	Null	XC3625	XC3626

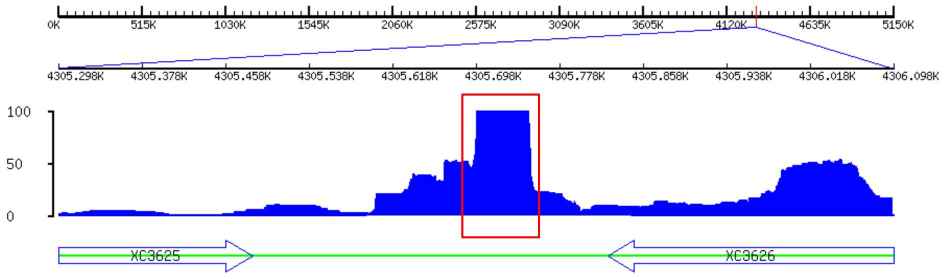

Transcript	Start	End	Length (bp)	RPKM	Inside gene	Overlap gene	Upstream gene	Downstream gene
TT561	4307826	4307891	66	73.9	Null	XC3628	XC3627	XC3629

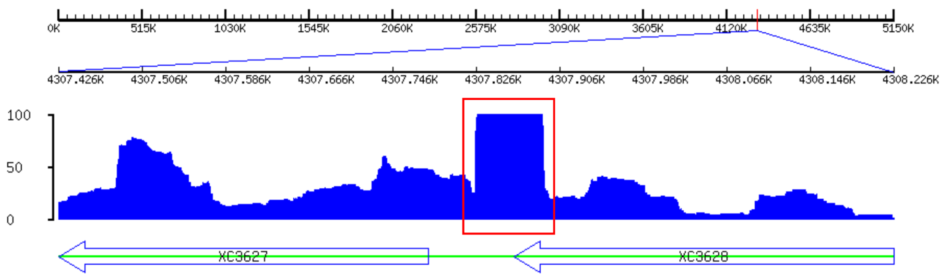

Transcript	Start	End	Length (bp)	RPKM	Inside gene	Overlap gene	Upstream gene	Downstream gene
TT562	4314352	4314420	69	103.8	XC3632	Null	XC3631	XC3633

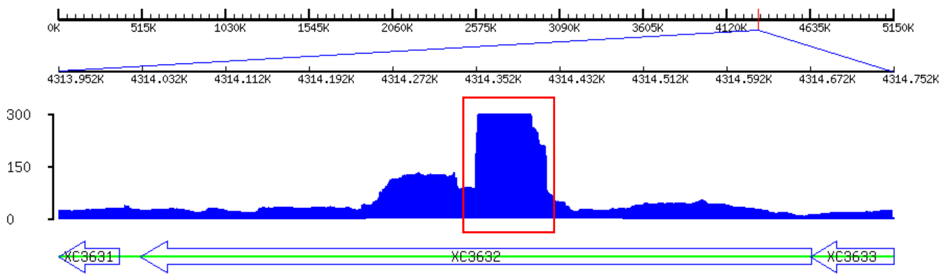

Transcript	Start	End	Length (bp)	RPKM	Inside gene	Overlap gene	Upstream gene	Downstream gene
TT563	4321220	4321278	59	30.9	XC3638	Null	XC3637	XC3639

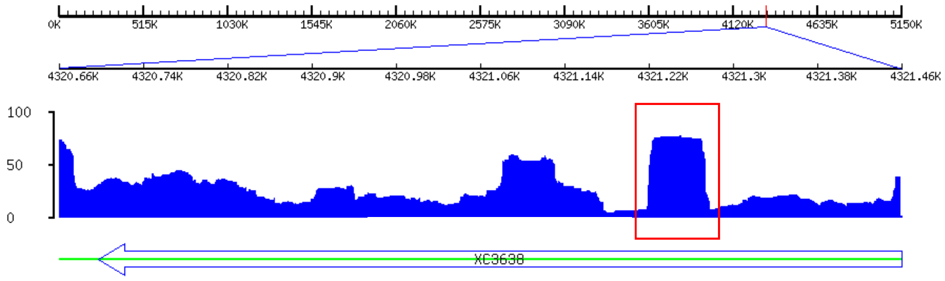

Transcript	Start	End	Length (bp)	RPKM	Inside gene	Overlap gene	Upstream gene	Downstream gene
TT564	4325572	4325730	158	527.7	Null	Null	XC3642	XC3643

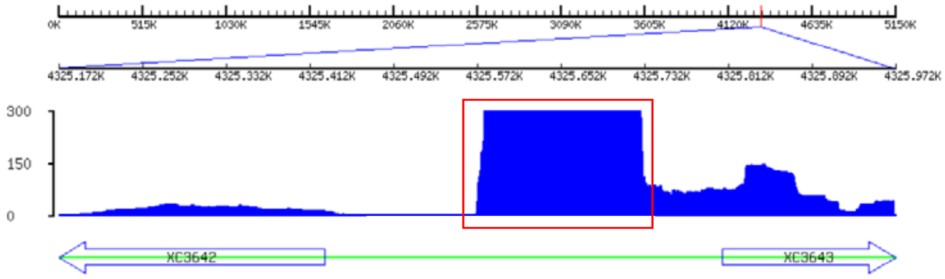

Transcript	Start	End	Length (bp)	RPKM	Inside gene	Overlap gene	Upstream gene	Downstream gene
TT565	4325578	4325645	67	1014.4	Null	Null	XC3642	XC3643

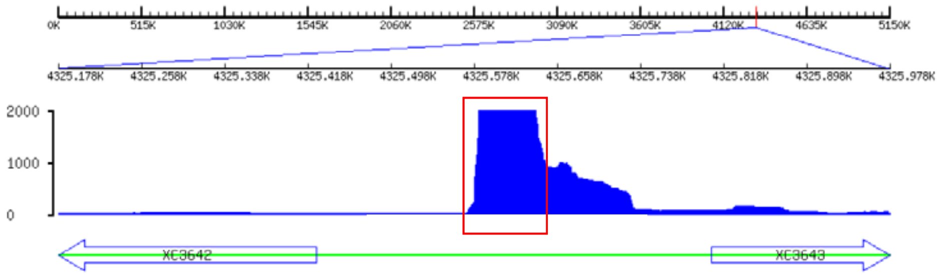

Transcript	Start	End	Length (bp)	RPKM	Inside gene	Overlap gene	Upstream gene	Downstream gene
TT566	4327830	4328260	431	1518.1	XC3645	Null	XC3644	XC3646

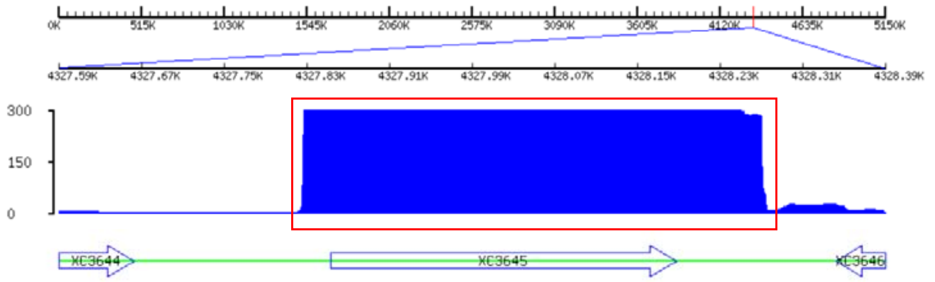

Transcript	Start	End	Length (bp)	RPKM	Inside gene	Overlap gene	Upstream gene	Downstream gene
TT567	4327830	4327882	53	5736.2	Null	XC3645	XC3644	XC3646
TT568	4328118	4328194	76	4608.9	Null	XC3645	XC3644	XC3646

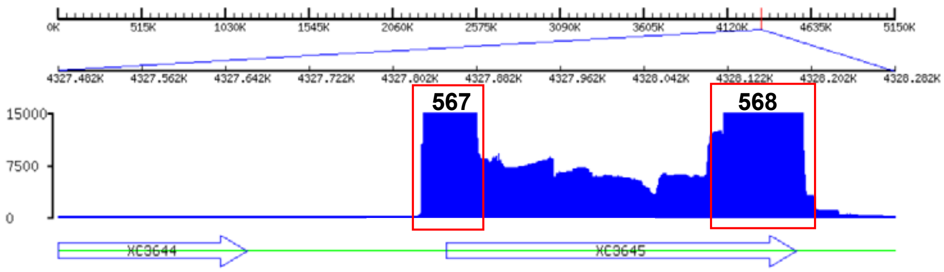

Transcript	Start	End	Length (bp)	RPKM	Inside gene	Overlap gene	Upstream gene	Downstream gene
TT569	4344293	4344482	190	221.9	Null	XC3659	XC3658	XC3660

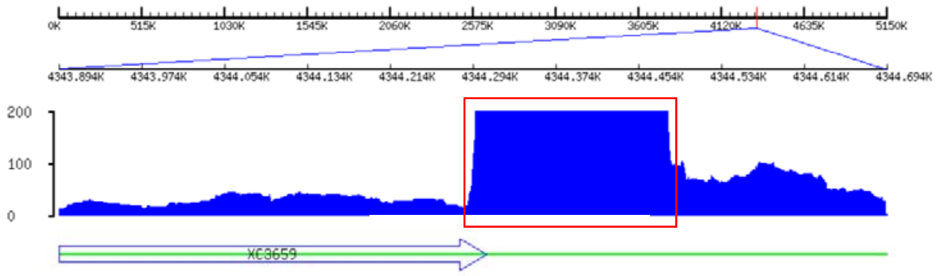

Transcript	Start	End	Length (bp)	RPKM	Inside gene	Overlap gene	Upstream gene	Downstream gene
TT570	4344297	4344393	97	324.4	Null	XC3659	XC3658	XC3660
TT571	4344414	4344482	69	154.6	Null	Null	XC3659	XC3660

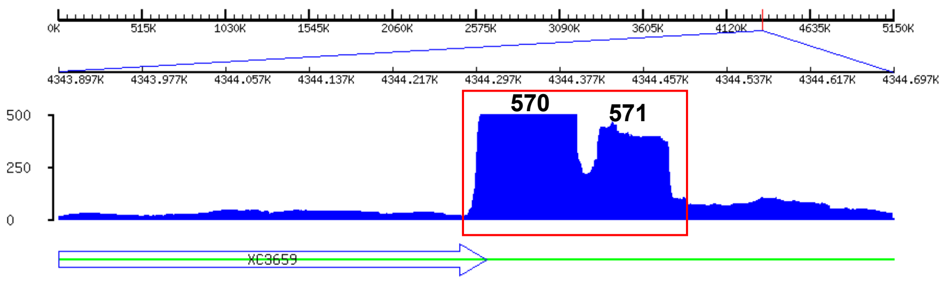

Transcript	Start	End	Length (bp)	RPKM	Inside gene	Overlap gene	Upstream gene	Downstream gene
TT572	4360151	4360233	83	104.3	Null	Null	XC3672	XC3673

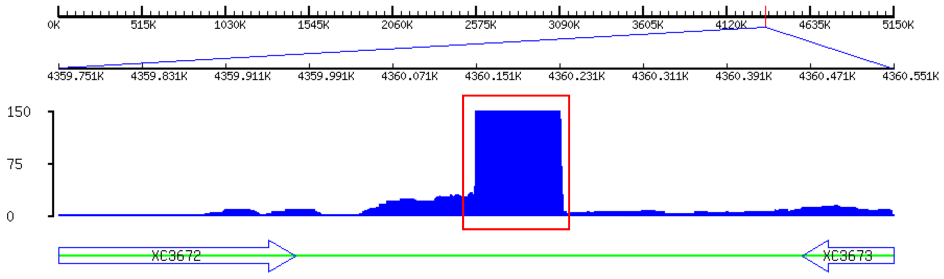

Transcript	Start	End	Length (bp)	RPKM	Inside gene	Overlap gene	Upstream gene	Downstream gene
TT573	4364450	4364513	64	40.9	Null	XC3676	XC3675	XC3677
TT574	4364624	4364711	88	173.3	Null	XC3677	XC3676	XC3678
TT575	4364742	4364839	97	112.2	XC3677	Null	XC3676	XC3678
TT576	4365101	4365191	91	802.2	Null	Null	XC3677	XC3678

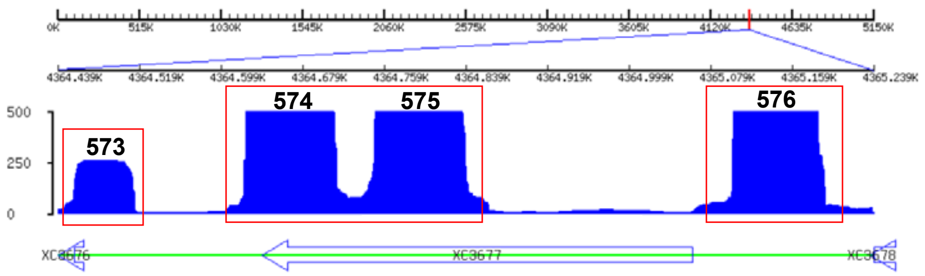

Transcript	Start	End	Length (bp)	RPKM	Inside gene	Overlap gene	Upstream gene	Downstream gene
TT577	4371817	4371917	101	106.4	XC3685	Null	XC3684	XC3686
TT578	4371971	4372060	90	126.1	Null	XC3685	XC3684	XC3686
TT579	4372202	4372293	92	322.8	Null	Null	XC3685	XC3686

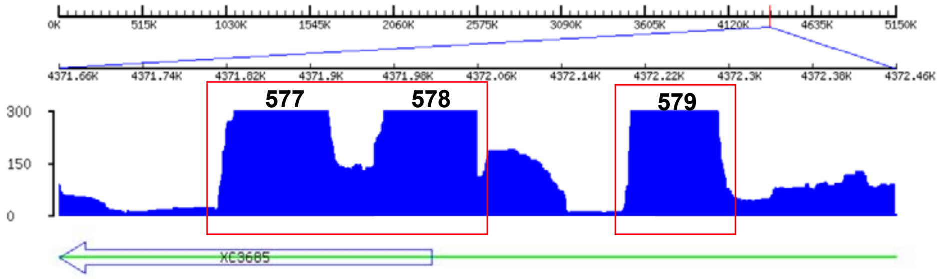

Transcript	Start	End	Length (bp)	RPKM	Inside gene	Overlap gene	Upstream gene	Downstream gene
TT580	4372499	4372665	167	437.6	Null	XC3686	XC3685	XC3687

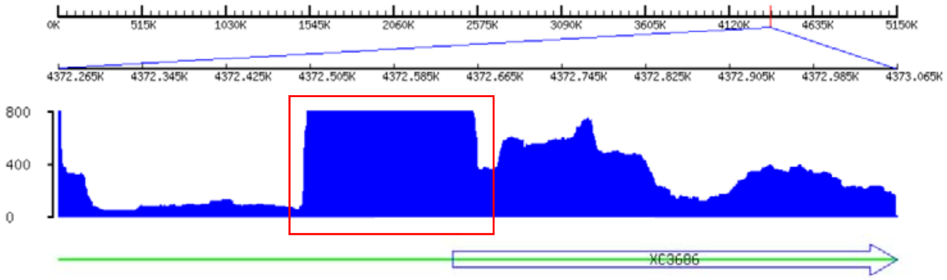

Transcript	Start	End	Length (bp)	RPKM	Inside gene	Overlap gene	Upstream gene	Downstream gene
TT581	4379005	4379095	91	43.3	XC3691	Null	XC3690	XC3692
TT582	4379127	4379216	90	66.1	XC3691	Null	XC3690	XC3692

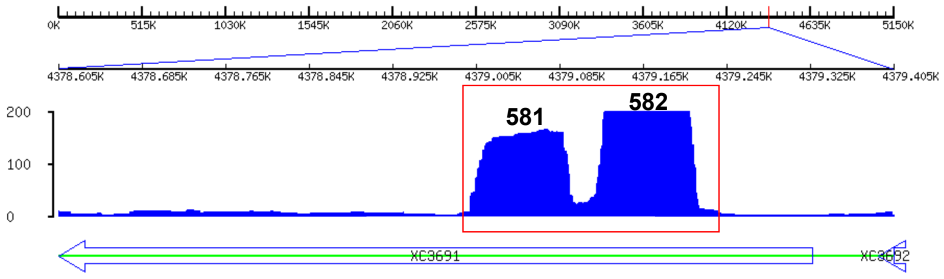

Transcript	Start	End	Length (bp)	RPKM	Inside gene	Overlap gene	Upstream gene	Downstream gene
TT583	4379743	4379816	74	51.1	Null	XC3692	XC3691	XC3693

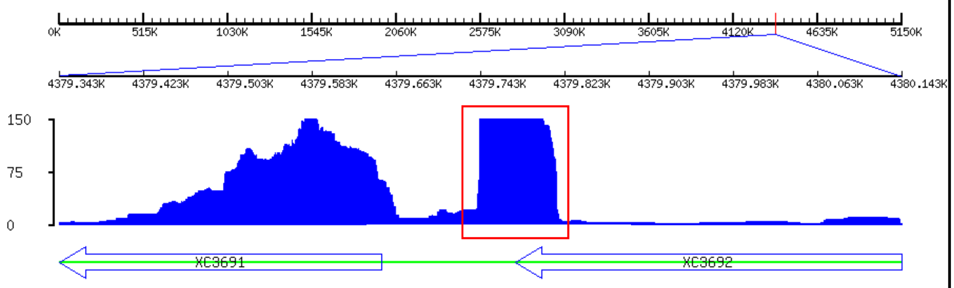

Transcript	Start	End	Length (bp)	RPKM	Inside gene	Overlap gene	Upstream gene	Downstream gene
TT584	4398939	4399013	75	791.9	Null	XC3711	XC3710	XC3712

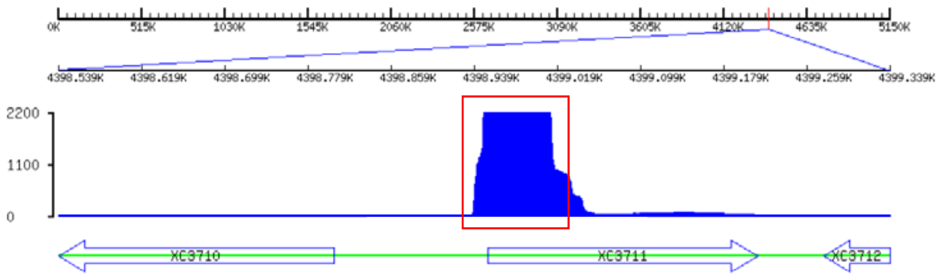

Transcript	Start	End	Length (bp)	RPKM	Inside gene	Overlap gene	Upstream gene	Downstream gene
TT585	4404218	4404294	77	314.9	XC3717	Null	XC3716	XC3718

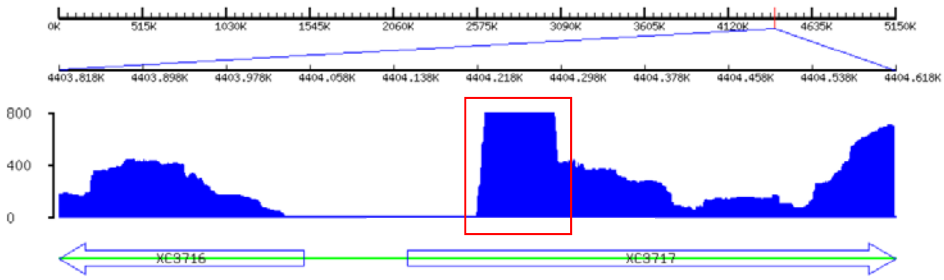

Transcript	Start	End	Length (bp)	RPKM	Inside gene	Overlap gene	Upstream gene	Downstream gene
TT586	4404650	4404744	94	708.2	XC3717	Null	XC3716	XC3718

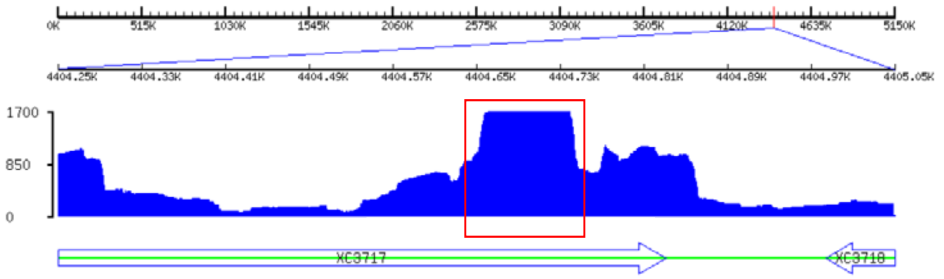

Transcript	Start	End	Length (bp)	RPKM	Inside gene	Overlap gene	Upstream gene	Downstream gene
TT587	4407445	4407503	59	174.4	Null	XC3720	XC3719	XC3721

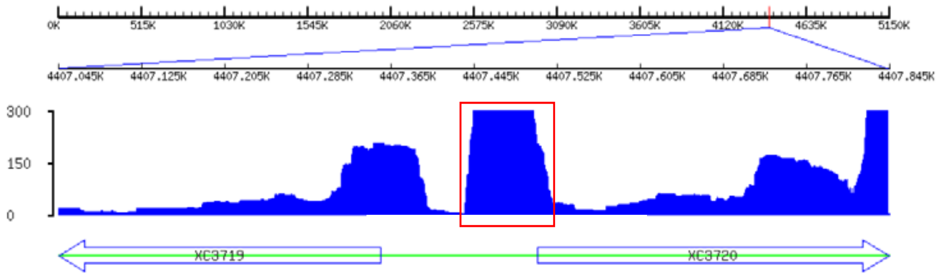

Transcript	Start	End	Length (bp)	RPKM	Inside gene	Overlap gene	Upstream gene	Downstream gene
TT588	4409083	4409150	68	106.1	Null	XC3722	XC3721	XC3723

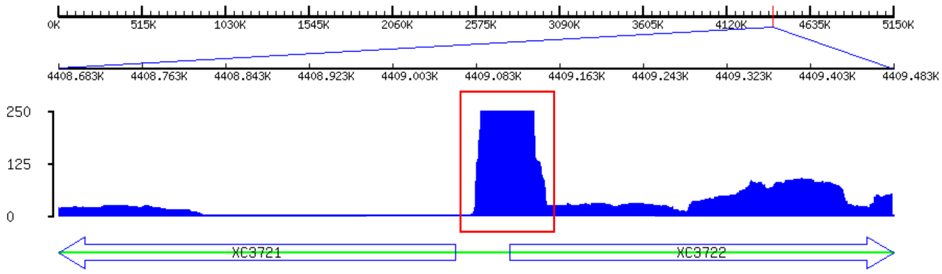

Transcript	Start	End	Length (bp)	RPKM	Inside gene	Overlap gene	Upstream gene	Downstream gene
TT589	4414867	4414937	71	30.6	Null	XC3729	XC3728	XC3730

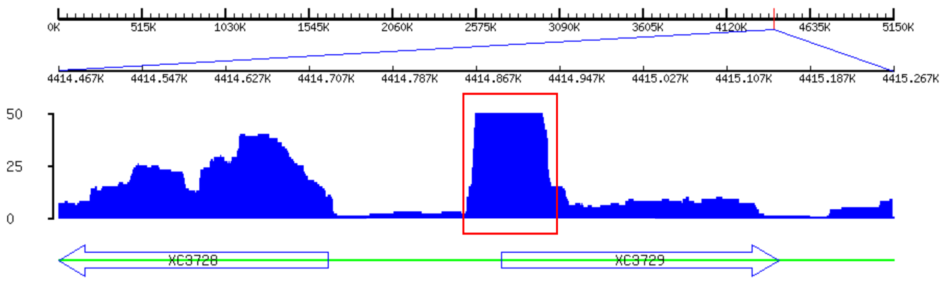

Transcript	Start	End	Length (bp)	RPKM	Inside gene	Overlap gene	Upstream gene	Downstream gene
TT590	4433479	4433579	101	54.9	XC3744	Null	XC3743	XC3745

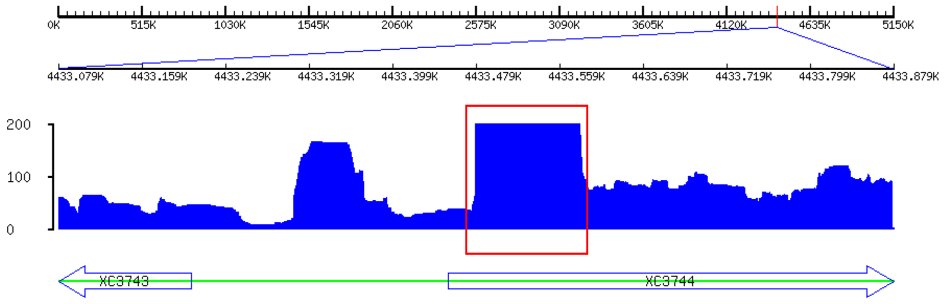

Transcript	Start	End	Length (bp)	RPKM	Inside gene	Overlap gene	Upstream gene	Downstream gene
TT591	4443157	4443419	263	227.3	Null	XC3754	XC3753	XC3755

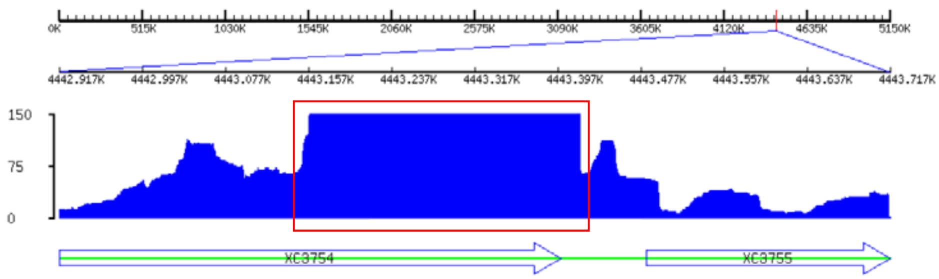

Transcript	Start	End	Length (bp)	RPKM	Inside gene	Overlap gene	Upstream gene	Downstream gene
TT592	4451453	4451532	80	559.8	XC3761	Null	XC3760	XC3762

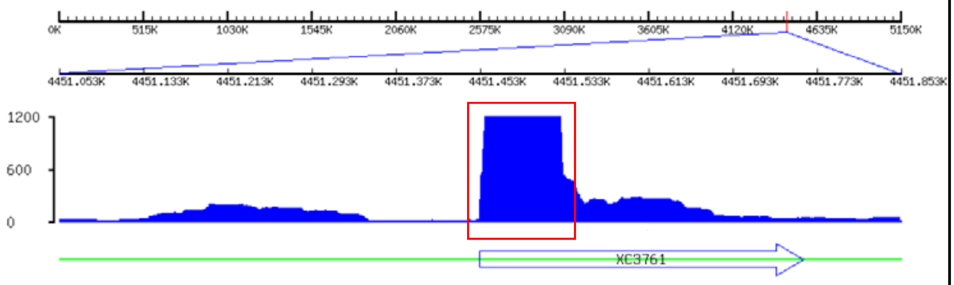

Transcript	Start	End	Length (bp)	RPKM	Inside gene	Overlap gene	Upstream gene	Downstream gene
TT593	4455684	4455754	71	80.8	XC3766	Null	XC3765	XC3767

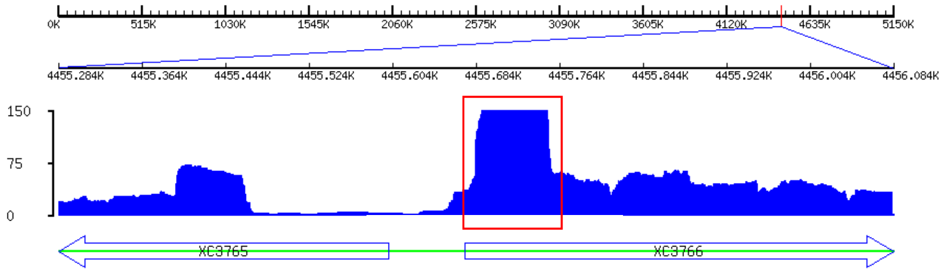

Transcript	Start	End	Length (bp)	RPKM	Inside gene	Overlap gene	Upstream gene	Downstream gene
TT594	4466873	4466962	90	104.5	Null	XC3775	XC3774	XC3776

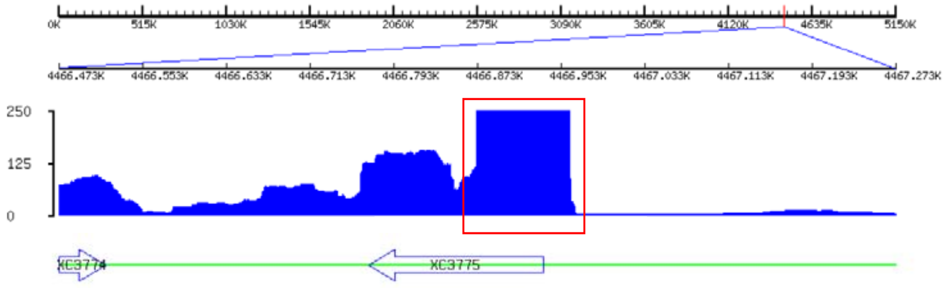

Transcript	Start	End	Length (bp)	RPKM	Inside gene	Overlap gene	Upstream gene	Downstream gene
TT595	4467352	4467466	115	118.6	XC3776	Null	XC3775	XC3777

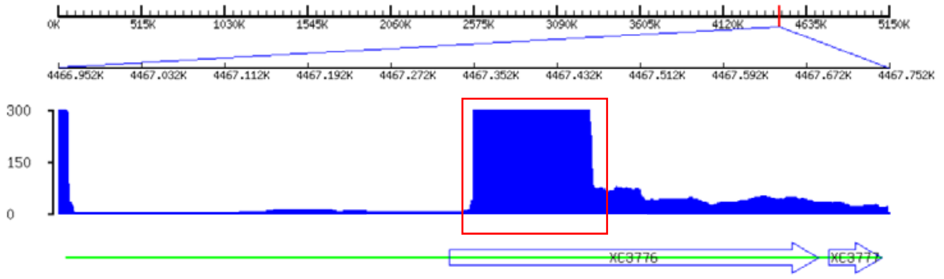

Transcript	Start	End	Length (bp)	RPKM	Inside gene	Overlap gene	Upstream gene	Downstream gene
TT596	4469094	4469207	114	98.9	Null	XC3779	XC3778	XC3780
TT597	4469312	4469372	61	94.9	Null	Null	XC3779	XC3780

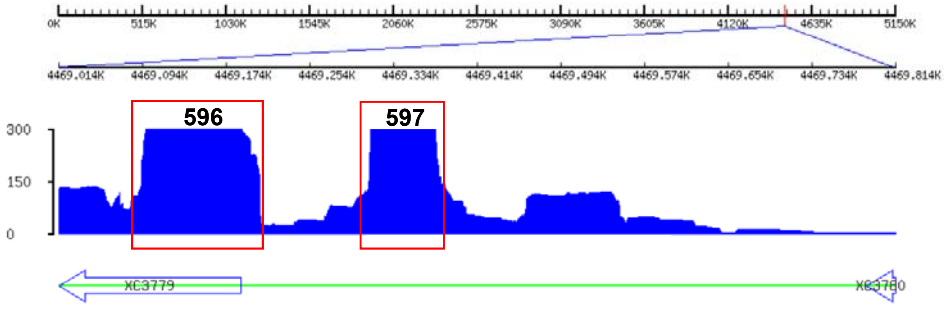

Transcript	Start	End	Length (bp)	RPKM	Inside gene	Overlap gene	Upstream gene	Downstream gene
TT598	4495726	4495826	102	305.6	Null	Null	XC3801	XC3802

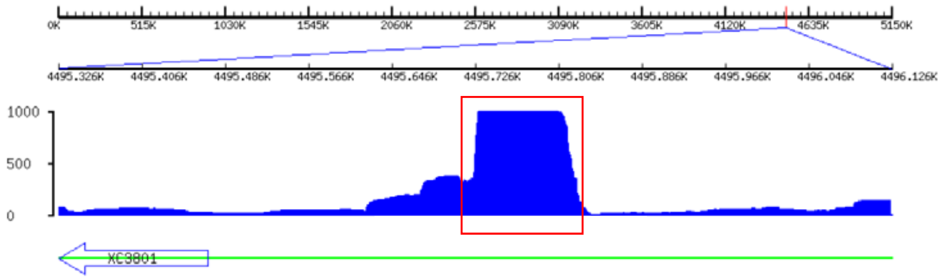

Transcript	Start	End	Length (bp)	RPKM	Inside gene	Overlap gene	Upstream gene	Downstream gene
TT599	4508950	4509041	92	332.6	XC3814	Null	XC3813	XC3815

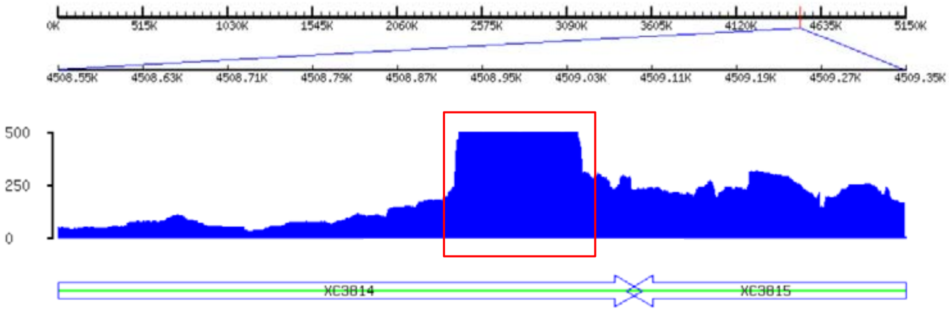

Transcript	Start	End	Length (bp)	RPKM	Inside gene	Overlap gene	Upstream gene	Downstream gene
TT600	4510347	4510416	70	52.8	Null	XC3815	XC3814	XC3816

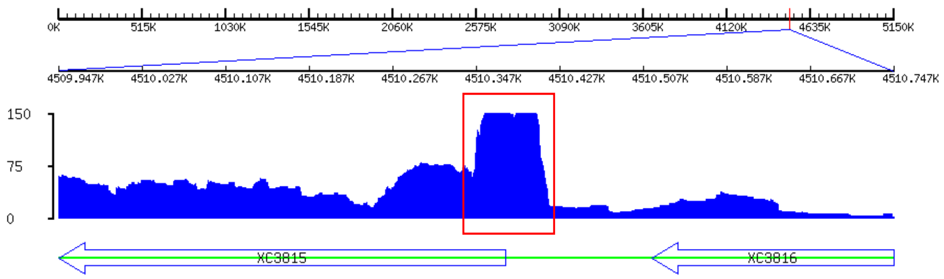

Transcript	Start	End	Length (bp)	RPKM	Inside gene	Overlap gene	Upstream gene	Downstream gene
TT601	4517683	4517754	72	113.5	XC3821	Null	XC3820	XC3822
TT602	4517831	4517924	94	330.7	Null	XC3821 XC3822	XC3820	XC3823
TT603	4517956	4518039	90	381.5	XC3822	Null	XC3821	XC3823

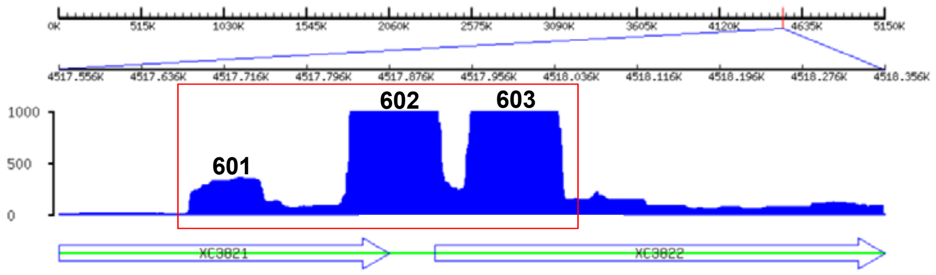

Transcript	Start	End	Length (bp)	RPKM	Inside gene	Overlap gene	Upstream gene	Downstream gene
TT604	4527317	4527368	52	159.3	Null	XC3830	XC3829	XC3831

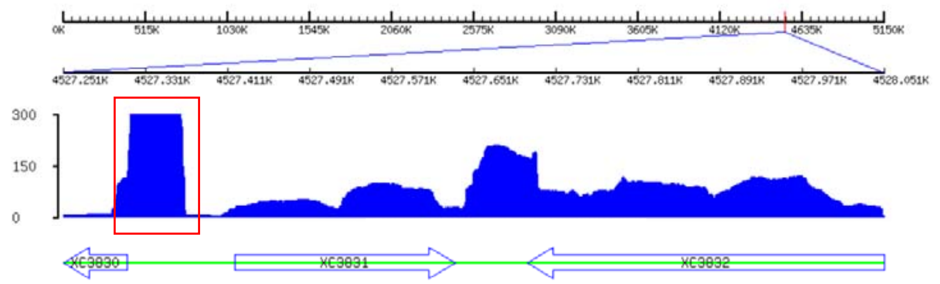

Transcript	Start	End	Length (bp)	RPKM	Inside gene	Overlap gene	Upstream gene	Downstream gene
TT605	4546818	4546871	54	500.3	Null	Null	XC3848	XC3849
TT606	4546883	4546990	107	329.1	Null	XC3849	XC3848	XC3850

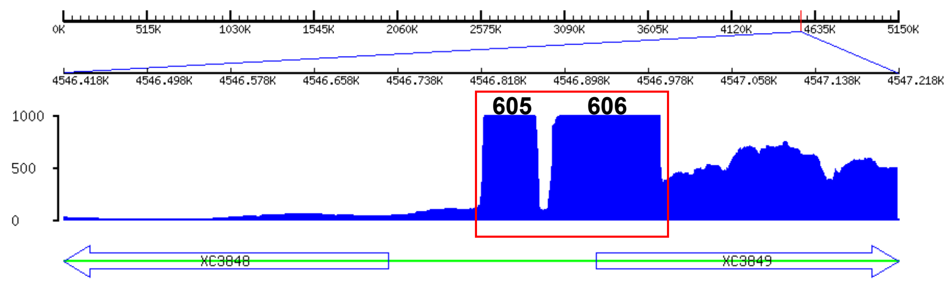

Transcript	Start	End	Length (bp)	RPKM	Inside gene	Overlap gene	Upstream gene	Downstream gene
TT607	4547381	4547437	57	716.5	Null	Null	XC3849	XC3850

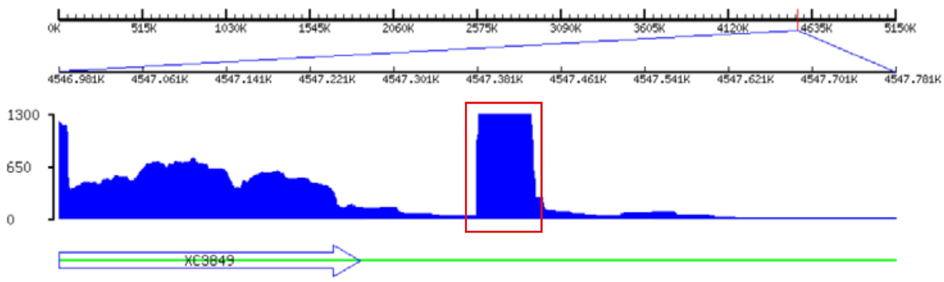

Transcript	Start	End	Length (bp)	RPKM	Inside gene	Overlap gene	Upstream gene	Downstream gene
TT608	4557051	4557138	87	407.9	Null	XC3856	XC3855	XC3857

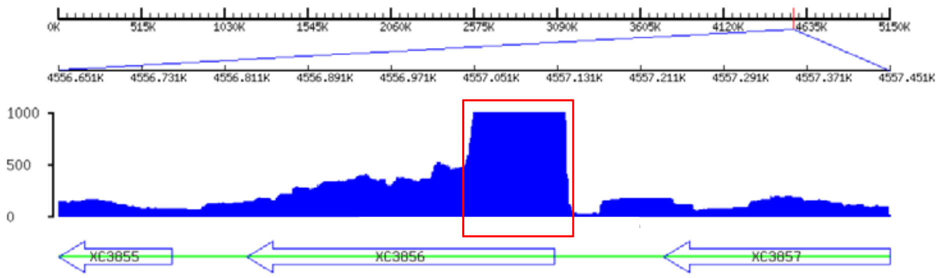

Transcript	Start	End	Length (bp)	RPKM	Inside gene	Overlap gene	Upstream gene	Downstream gene
TT609	4559479	4559533	55	14.2	Null	XC3585	XC3584	XC3586
TT610	4559652	4559742	91	311.9	Null	Null	XC3858	XC3859
TT611	4559818	4559884	67	92.3	XC3859	Null	XC3858	XC3860

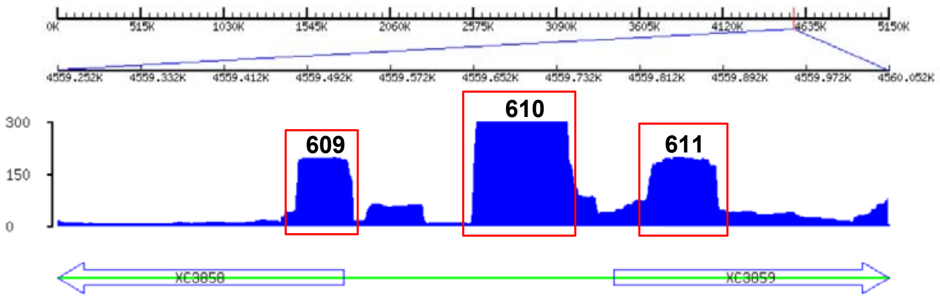

Transcript	Start	End	Length (bp)	RPKM	Inside gene	Overlap gene	Upstream gene	Downstream gene
TT612	4572028	4572079	52	114.4	Null	XC3865	XC3864	XC3866

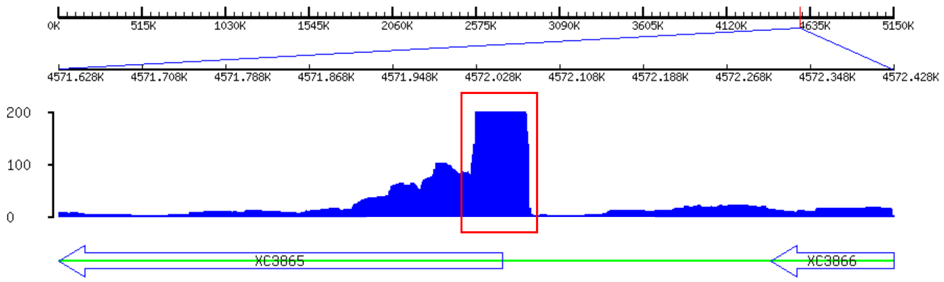

Transcript	Start	End	Length (bp)	RPKM	Inside gene	Overlap gene	Upstream gene	Downstream gene
TT613	4573570	4573631	62	108.5	Null	Null	XC3867	XC3868

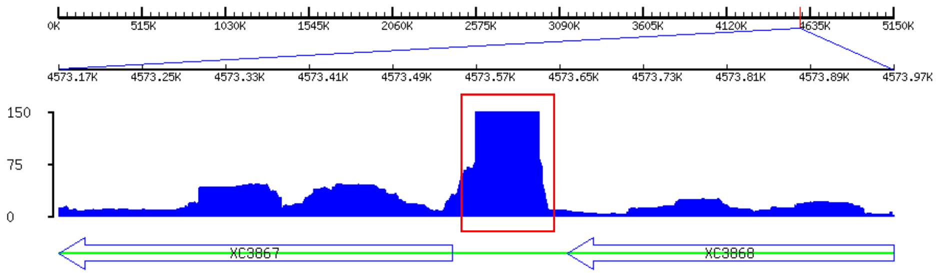

Transcript	Start	End	Length (bp)	RPKM	Inside gene	Overlap gene	Upstream gene	Downstream gene
TT614	4575097	4575235	139	231.9	Null	Null	XC3869	XC3870

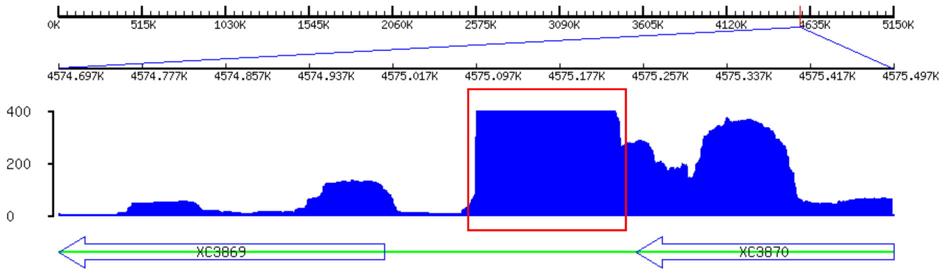

Transcript	Start	End	Length (bp)	RPKM	Inside gene	Overlap gene	Upstream gene	Downstream gene
TT615	4575554	4575632	79	139.4	Null	XC3870	XC3869	XC3871

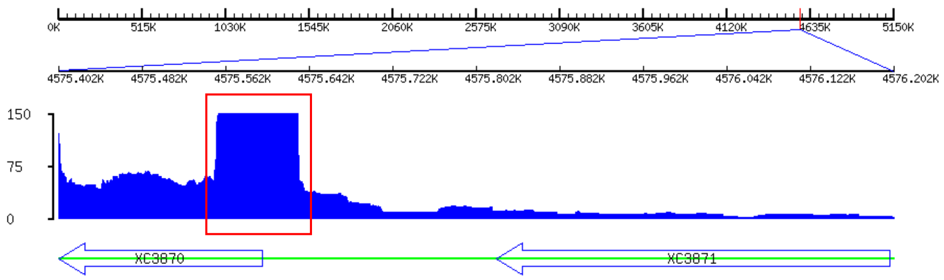

Transcript	Start	End	Length (bp)	RPKM	Inside gene	Overlap gene	Upstream gene	Downstream gene
TT616	4586683	4586871	189	118.4	XC3882	Null	XC3881	XC3883

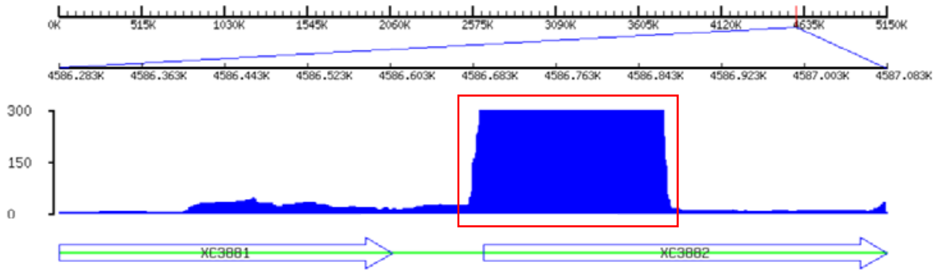

Transcript	Start	End	Length (bp)	RPKM	Inside gene	Overlap gene	Upstream gene	Downstream gene
TT617	4587350	4587674	325	204.4	XC3883	Null	XC3882	XC3884

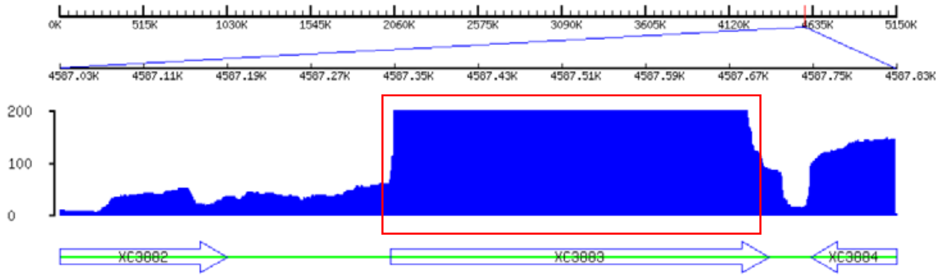

Transcript	Start	End	Length (bp)	RPKM	Inside gene	Overlap gene	Upstream gene	Downstream gene
TT618	4587350	4587408	59	545.9	XC3883	Null	XC3882	XC3884

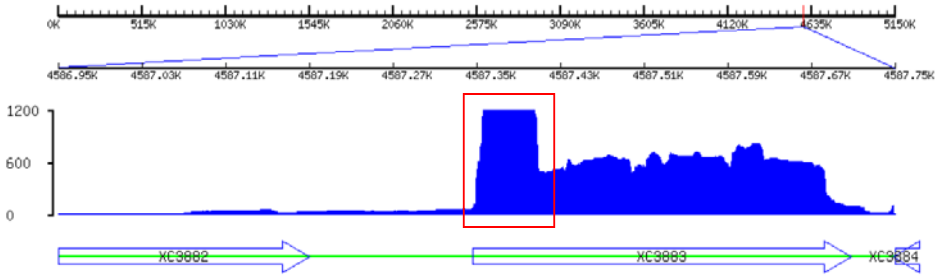

Transcript	Start	End	Length (bp)	RPKM	Inside gene	Overlap gene	Upstream gene	Downstream gene
TT619	4589554	4589608	55	478.1	Null	Null	XC3885	XC3886

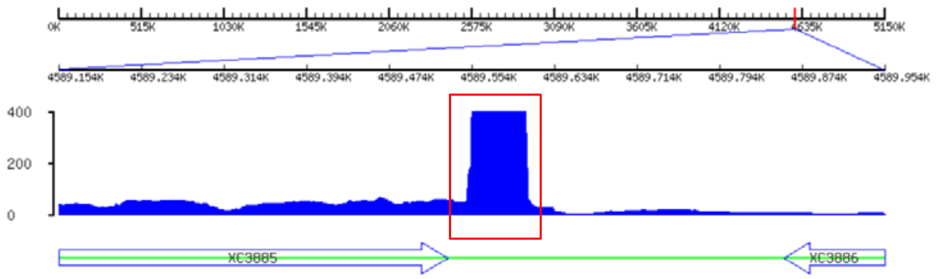

Transcript	Start	End	Length (bp)	RPKM	Inside gene	Overlap gene	Upstream gene	Downstream gene
TT620	4594306	4594362	57	179.1	Null	Null	XC3889	XC3890

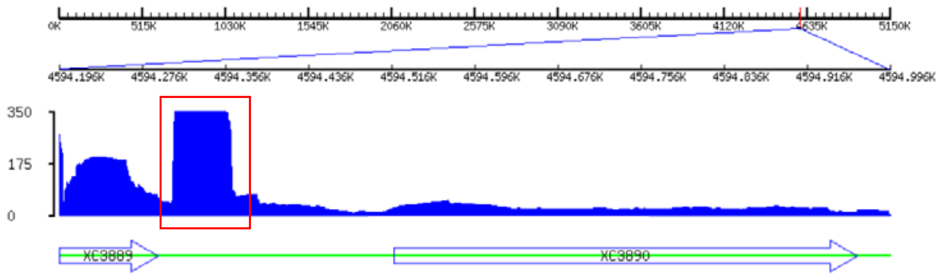

Transcript	Start	End	Length (bp)	RPKM	Inside gene	Overlap gene	Upstream gene	Downstream gene
TT621	4610834	4610889	56	22.5	XC3907	Null	XC3906	XC3908

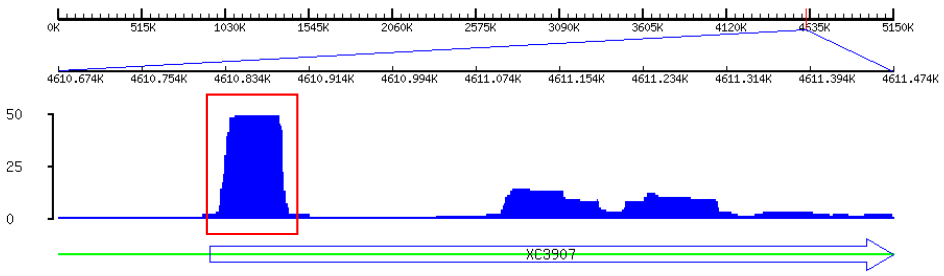

Transcript	Start	End	Length (bp)	RPKM	Inside gene	Overlap gene	Upstream gene	Downstream gene
TT622	4618882	4619047	165	1307.8	Null	Null	XC3912	XC3913

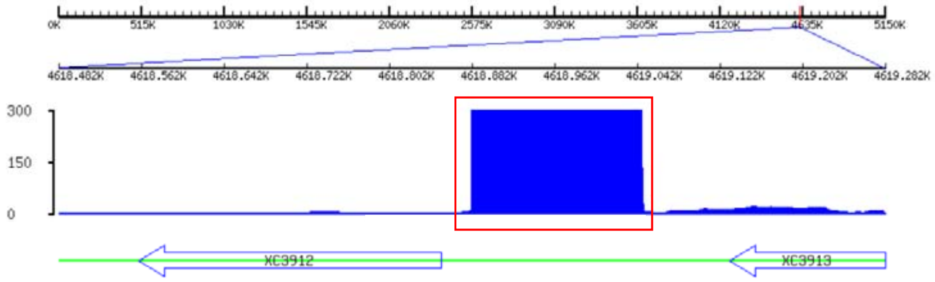

Transcript	Start	End	Length (bp)	RPKM	Inside gene	Overlap gene	Upstream gene	Downstream gene
TT623	4618882	4618947	65	2699.8	Null	Null	XC3912	XC3913

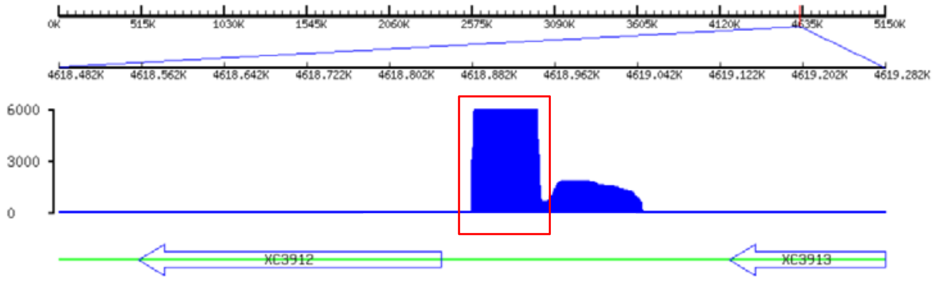

Transcript	Start	End	Length (bp)	RPKM	Inside gene	Overlap gene	Upstream gene	Downstream gene
TT624	4637937	4638023	87	3640.8	Null	Null	XC4386	XC3925

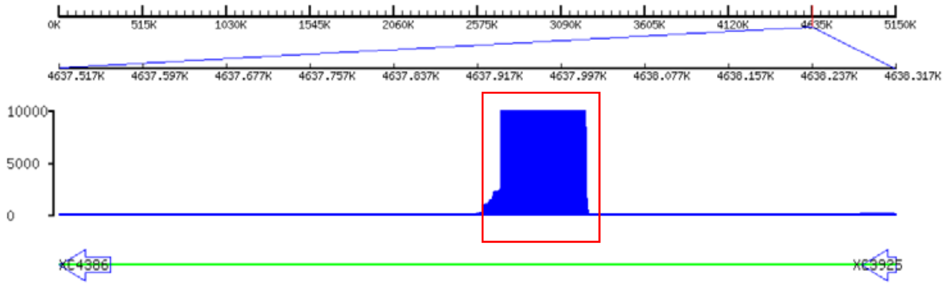

Transcript	Start	End	Length (bp)	RPKM	Inside gene	Overlap gene	Upstream gene	Downstream gene
TT625	4642808	4642902	95	99.1	Null	XC3928	XC3927	XC3929

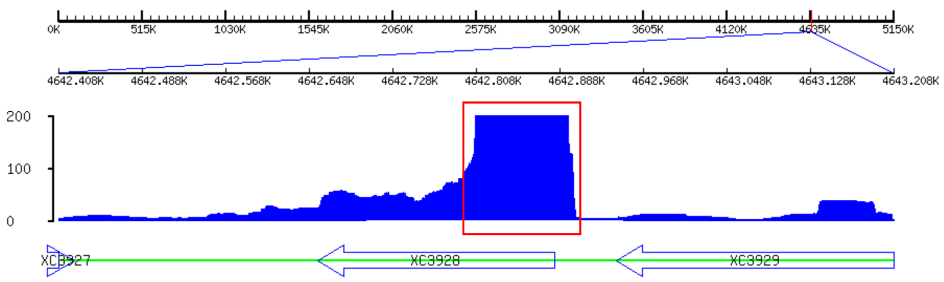

Transcript	Start	End	Length (bp)	RPKM	Inside gene	Overlap gene	Upstream gene	Downstream gene
TT626	4643585	4643661	77	29.6	XC3929	Null	XC3928	XC3930

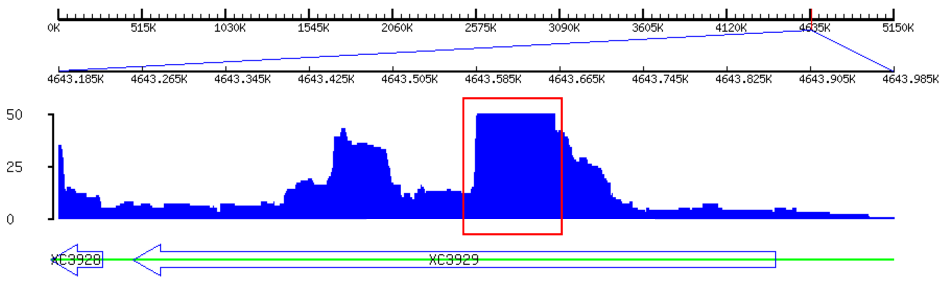

Transcript	Start	End	Length (bp)	RPKM	Inside gene	Overlap gene	Upstream gene	Downstream gene
TT627	4646038	4646102	65	474.6	Null	XC3931	XC3930	XC3932

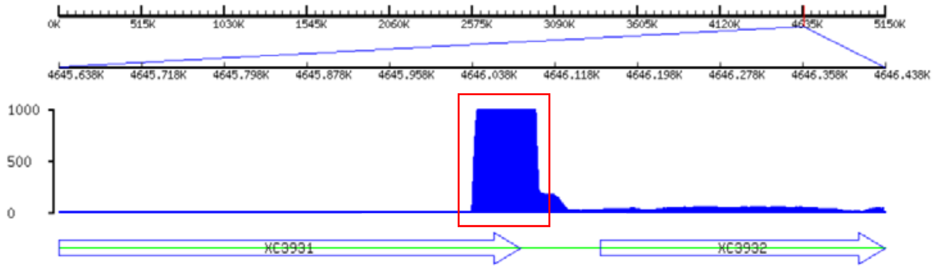

Transcript	Start	End	Length (bp)	RPKM	Inside gene	Overlap gene	Upstream gene	Downstream gene
TT628	4669328	4669448	121	47.4	Null	Null	XC3952	XC3953

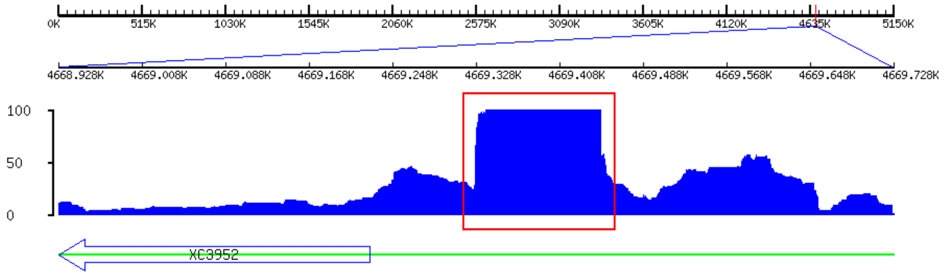

Transcript	Start	End	Length (bp)	RPKM	Inside gene	Overlap gene	Upstream gene	Downstream gene
TT629	4674462	4674571	110	24.1	Null	Null	XC3955	XC3956

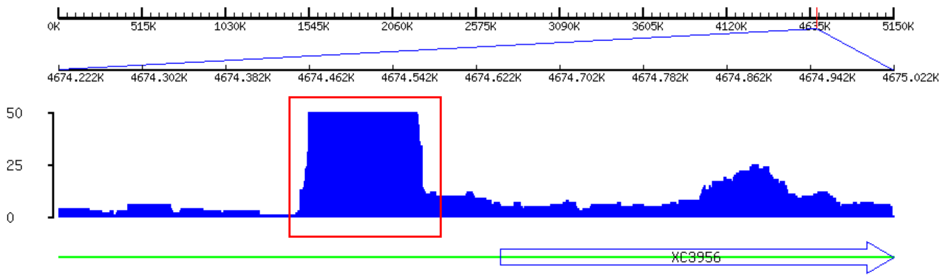

Transcript	Start	End	Length (bp)	RPKM	Inside gene	Overlap gene	Upstream gene	Downstream gene
TT630	4678700	4678943	244	2141.7	XC3961	XC3961	XC3960	XC3962

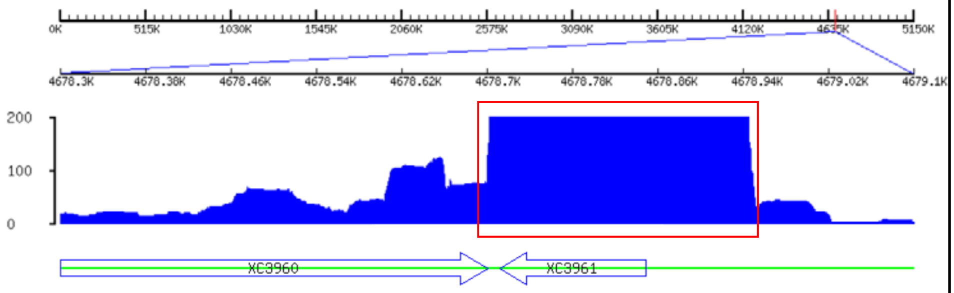

Transcript	Start	End	Length (bp)	RPKM	Inside gene	Overlap gene	Upstream gene	Downstream gene
TT631	4678850	4678945	105	4811.5	Null	XC3961	XC3960	XC3962

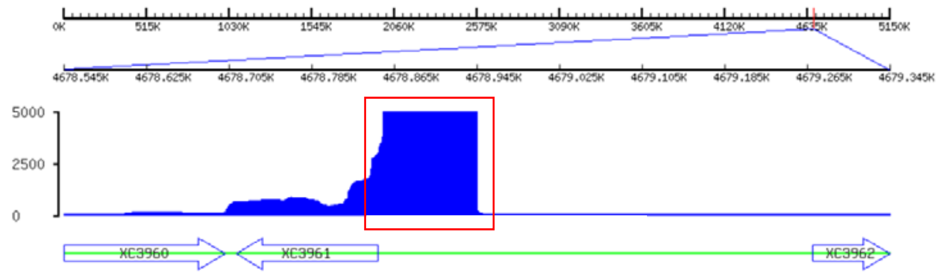

Transcript	Start	End	Length (bp)	RPKM	Inside gene	Overlap gene	Upstream gene	Downstream gene
TT632	4683269	4683337	69	174.8	XC3965	Null	XC3964	XC3966
TT633	4683418	4683578	160	160.6	XC3965	Null	XC3964	XC3966

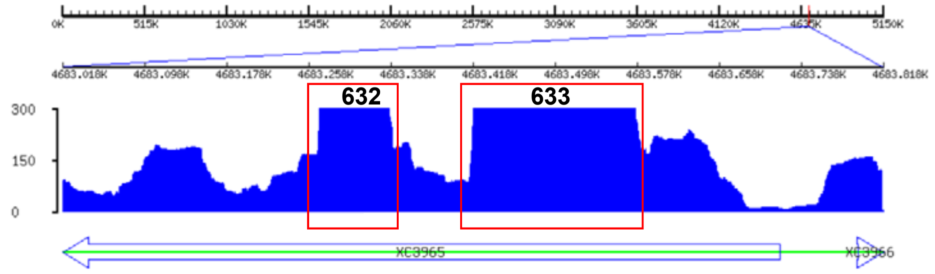

Transcript	Start	End	Length (bp)	RPKM	Inside gene	Overlap gene	Upstream gene	Downstream gene
TT634	4685557	4685687	131	612.3	XC3968	XC3968	XC3967	XC3969

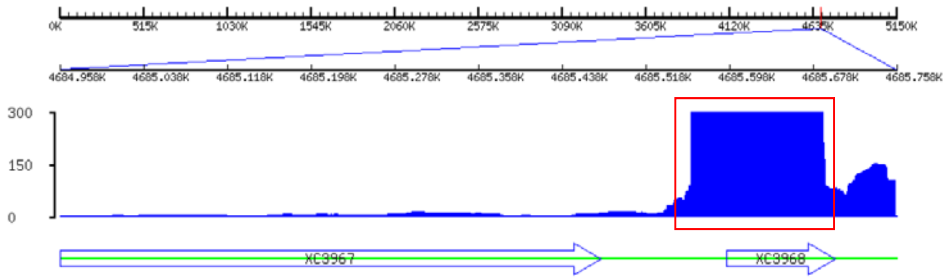

Transcript	Start	End	Length (bp)	RPKM	Inside gene	Overlap gene	Upstream gene	Downstream gene
TT635	4685562	4685622	61	996.8	Null	XC3968	XC3967	XC4387
TT636	4685823	4685908	86	484.3	Null	Null	XC3968	XC4387

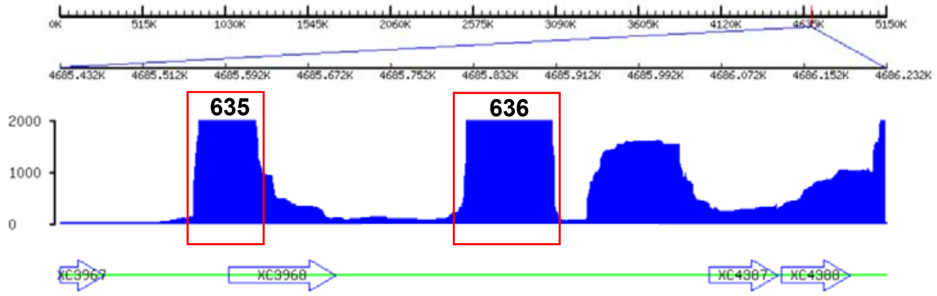

Transcript	Start	End	Length (bp)	RPKM	Inside gene	Overlap gene	Upstream gene	Downstream gene
TT637	4689116	4689212	97	321.3	Null	XC3971	XC3970	XC3972

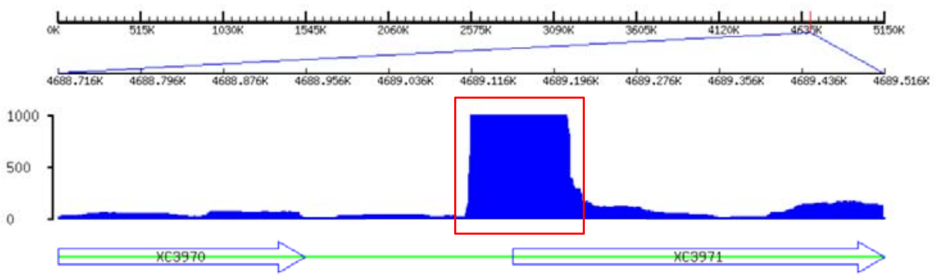

Transcript	Start	End	Length (bp)	RPKM	Inside gene	Overlap gene	Upstream gene	Downstream gene
TT638	4692392	4692484	92	297.7	XC3974	Null	XC3973	XC3975

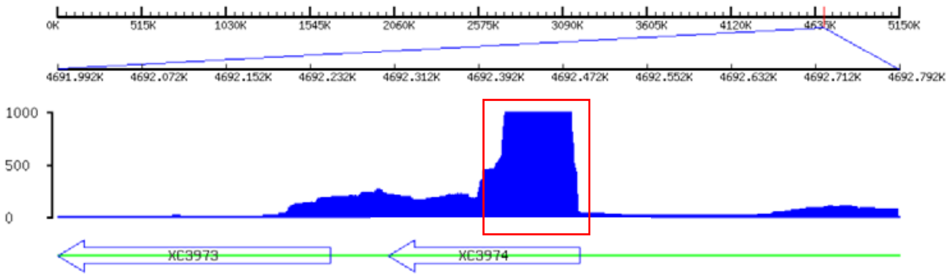

Transcript	Start	End	Length (bp)	RPKM	Inside gene	Overlap gene	Upstream gene	Downstream gene
TT639	4701609	4701678	70	81.5	Null	XC3986	XC3985	XC3987

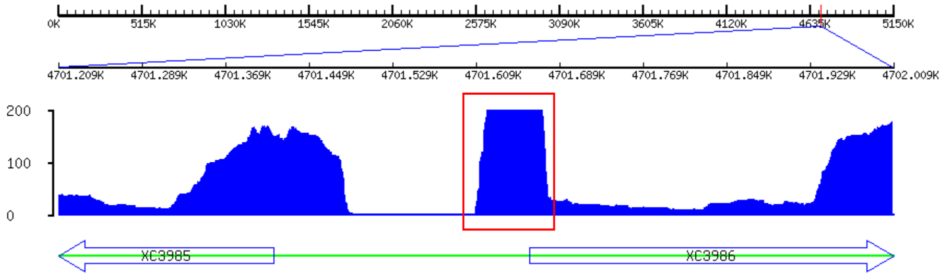

Transcript	Start	End	Length (bp)	RPKM	Inside gene	Overlap gene	Upstream gene	Downstream gene
TT640	4702331	4702421	91	88.4	XC3986	Null	XC3985	XC3987

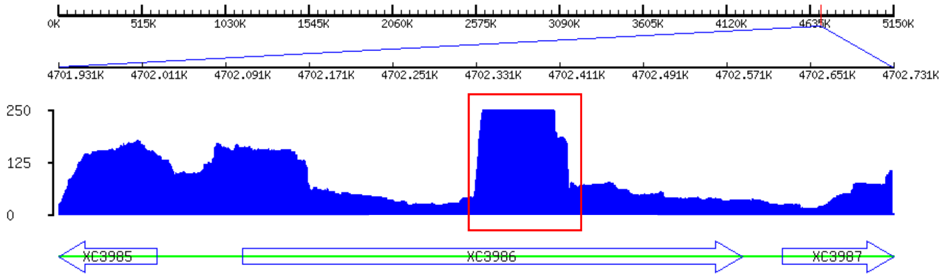

Transcript	Start	End	Length (bp)	RPKM	Inside gene	Overlap gene	Upstream gene	Downstream gene
TT641	4703172	4703262	91	270.1	Null	XC3987	XC3986	XC3988

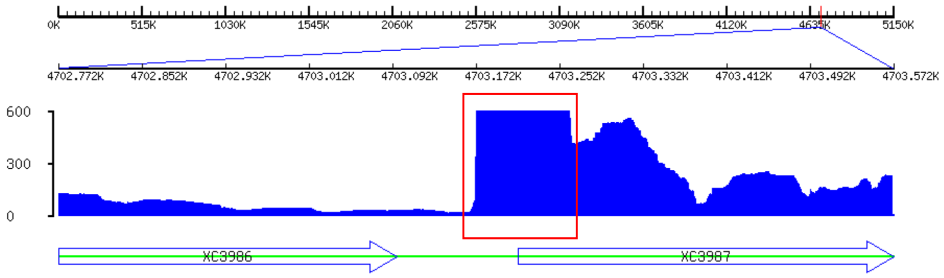

Transcript	Start	End	Length (bp)	RPKM	Inside gene	Overlap gene	Upstream gene	Downstream gene
TT642	4705783	4705854	72	262.8	Null	Null	XC3990	XC3991

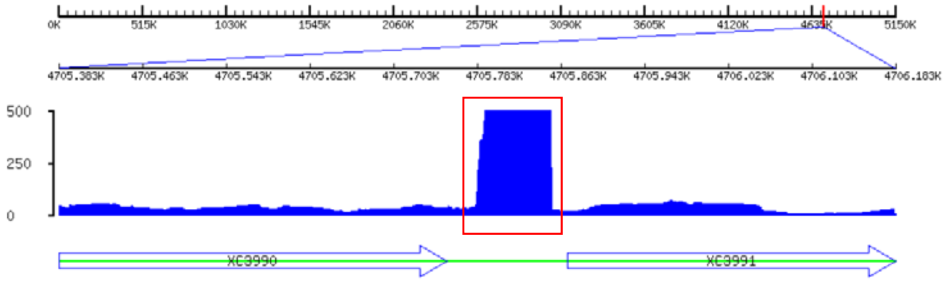

Transcript	Start	End	Length (bp)	RPKM	Inside gene	Overlap gene	Upstream gene	Downstream gene
TT643	4732595	4732653	59	189.4	Null	XC4016	XC4015	XC4017

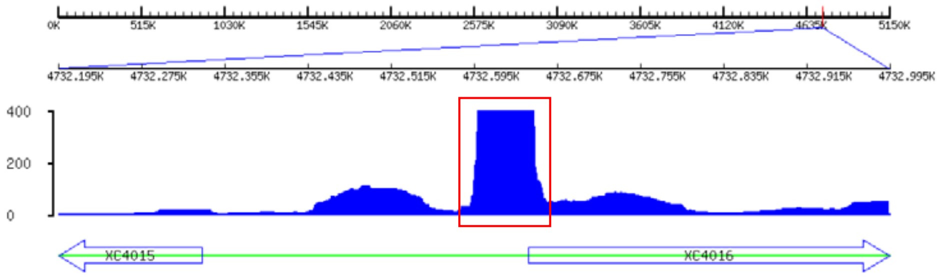

Transcript	Start	End	Length (bp)	RPKM	Inside gene	Overlap gene	Upstream gene	Downstream gene
TT644	4735193	4735282	90	156.9	Null	Null	XC4019	XC4020
TT645	4735457	4735513	56	26.5	XC4020	Null	XC4019	XC4021

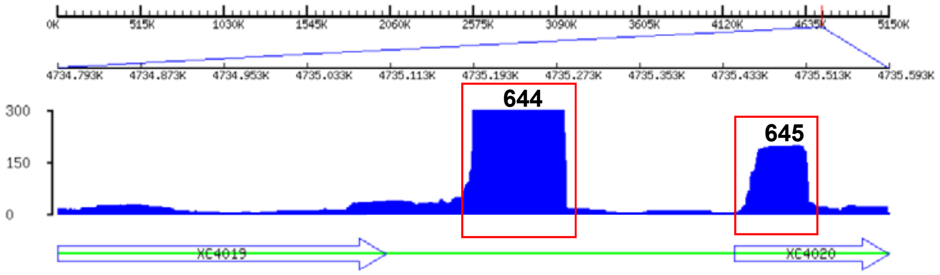

Transcript	Start	End	Length (bp)	RPKM	Inside gene	Overlap gene	Upstream gene	Downstream gene
TT646	4738459	4738522	63	89933.7	XC4389	Null	XC4023	XC4024

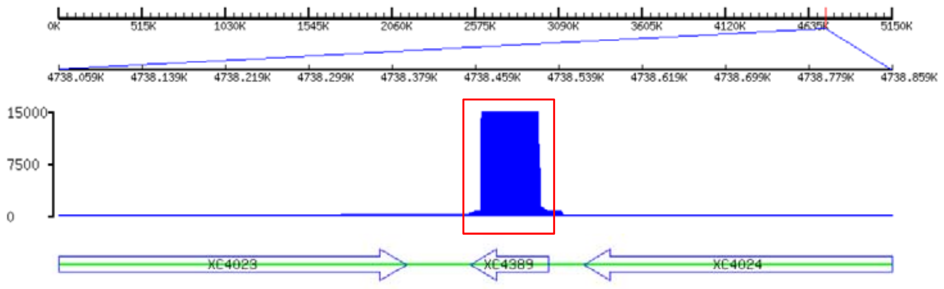

Transcript	Start	End	Length (bp)	RPKM	Inside gene	Overlap gene	Upstream gene	Downstream gene
TT647	4747532	4747590	85	920.7	XC4032	Null	XC4031	XC4033

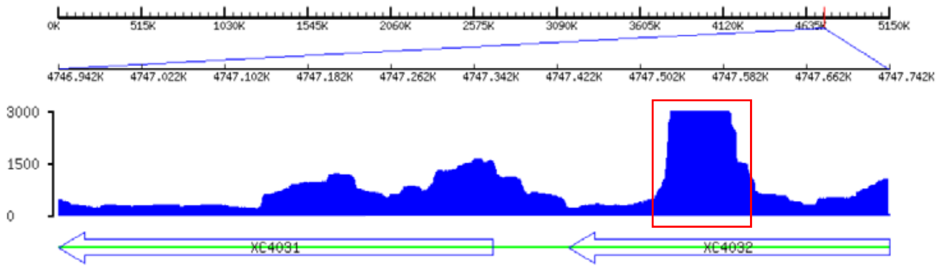

Transcript	Start	End	Length (bp)	RPKM	Inside gene	Overlap gene	Upstream gene	Downstream gene
TT648	4747790	4747883	94	3275.1	Null	XC4032	XC4031	XC4033

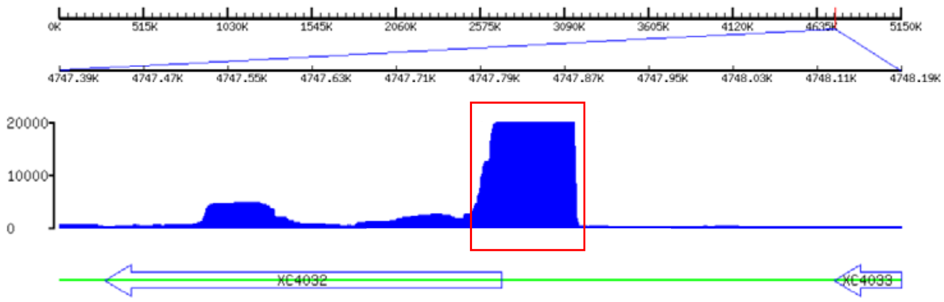

Transcript	Start	End	Length (bp)	RPKM	Inside gene	Overlap gene	Upstream gene	Downstream gene
TT649	4762773	4762863	90	81.6	XC4047	Null	XC4046	XC4048

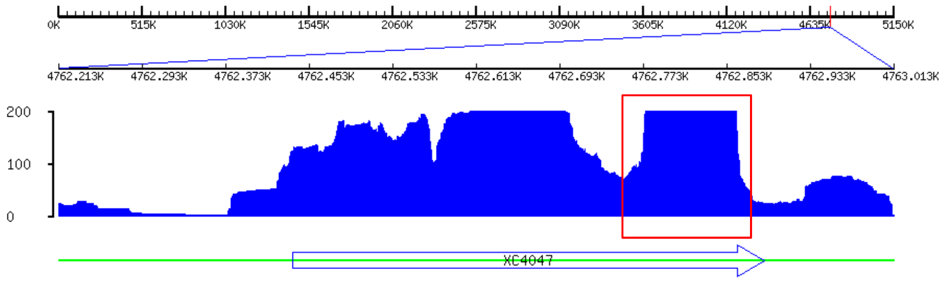

Transcript	Start	End	Length (bp)	RPKM	Inside gene	Overlap gene	Upstream gene	Downstream gene
TT650	4797114	4797205	92	790.3	Null	Null	XC4076	XC4077

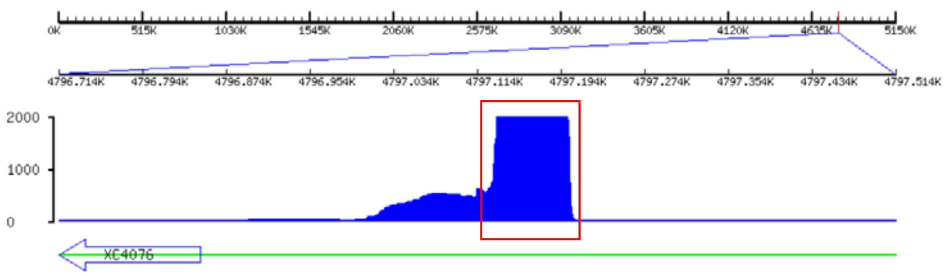

Transcript	Start	End	Length (bp)	RPKM	Inside gene	Overlap gene	Upstream gene	Downstream gene
TT651	4815362	4815422	61	150.3	Null	XC4087	XC4086	XC4088

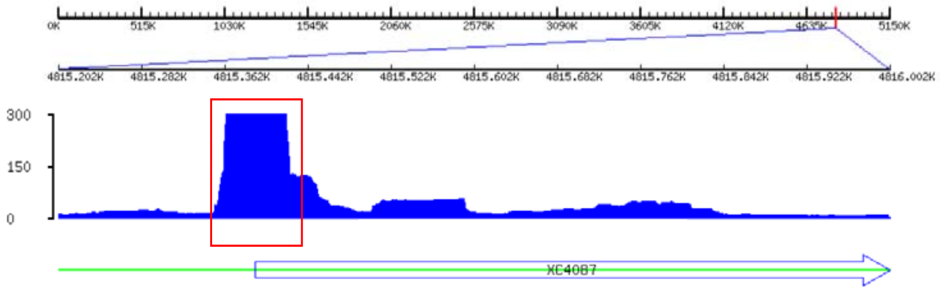

Transcript	Start	End	Length (bp)	RPKM	Inside gene	Overlap gene	Upstream gene	Downstream gene
TT652	4828507	4828590	84	112.9	Null	XC4100	XC4099	XC4101

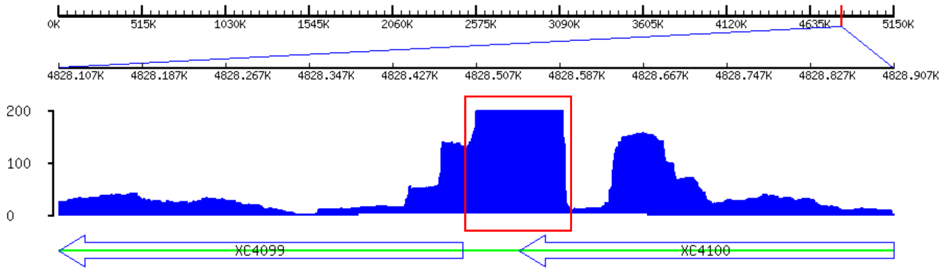

Transcript	Start	End	Length (bp)	RPKM	Inside gene	Overlap gene	Upstream gene	Downstream gene
TT653	4839865	4839956	92	17436.3	Null	Null	XC4108	XC4109

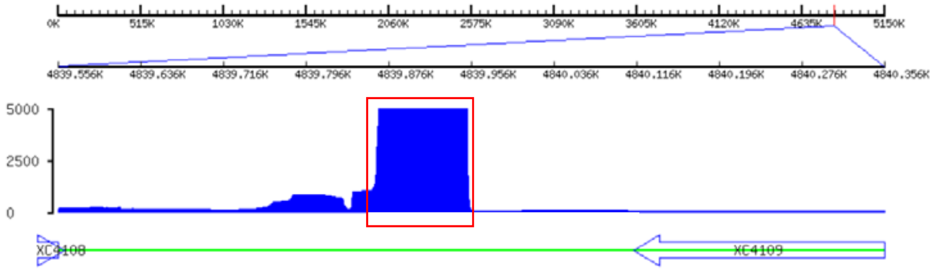

Transcript	Start	End	Length (bp)	RPKM	Inside gene	Overlap gene	Upstream gene	Downstream gene
TT654	4849090	4849154	65	154.2	Null	Null	XC4115	XC4116

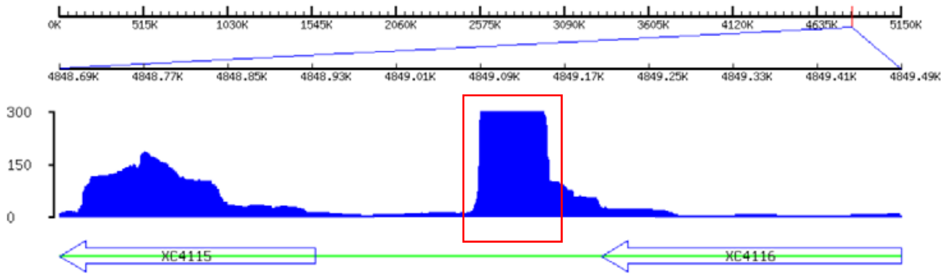

Transcript	Start	End	Length (bp)	RPKM	Inside gene	Overlap gene	Upstream gene	Downstream gene
TT655	4857835	4857887	53	89.5	Null	XC4122	XC4121	XC4123

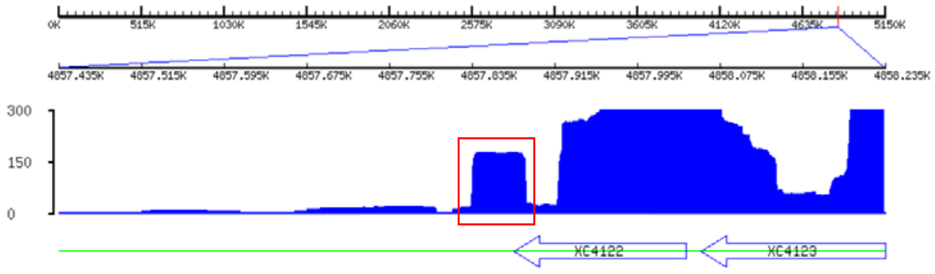

Transcript	Start	End	Length (bp)	RPKM	Inside gene	Overlap gene	Upstream gene	Downstream gene
TT656	4857835	4858443	610	58.7	Null	XC4122 /XC4123	XC4121	XC4124

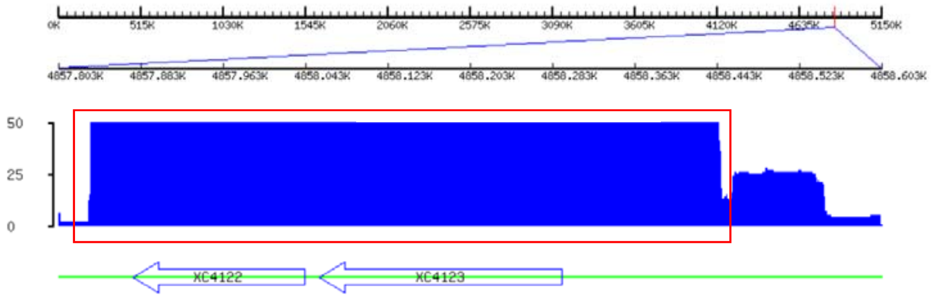

Transcript	Start	End	Length (bp)	RPKM	Inside gene	Overlap gene	Upstream gene	Downstream gene
TT657	4871721	4871920	200	85.8	Null	XC4133	XC4132	XC4134

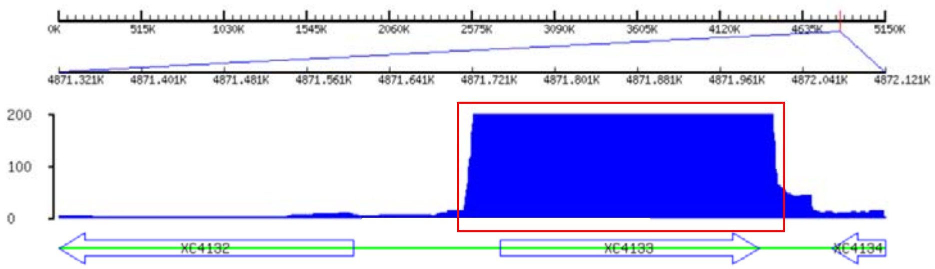

Transcript	Start	End	Length (bp)	RPKM	Inside gene	Overlap gene	Upstream gene	Downstream gene
TT658	4895422	4895595	174	18.3	Null	XC4152	XC4151	XC4153

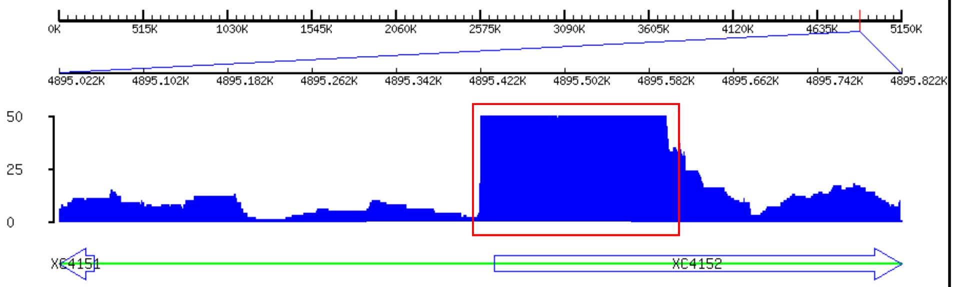

Transcript	Start	End	Length (bp)	RPKM	Inside gene	Overlap gene	Upstream gene	Downstream gene
TT659	4895947	4896240	294	473.9	Null	XC4152/ XC4153	XC4151	XC4154

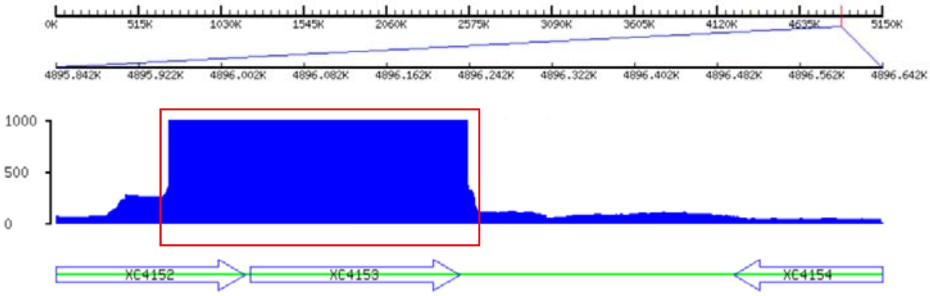

Transcript	Start	End	Length (bp)	RPKM	Inside gene	Overlap gene	Upstream gene	Downstream gene
TT660	4895947	4896120	174	699.7	Null	XC4152/ XC4153	XC4151	XC4154

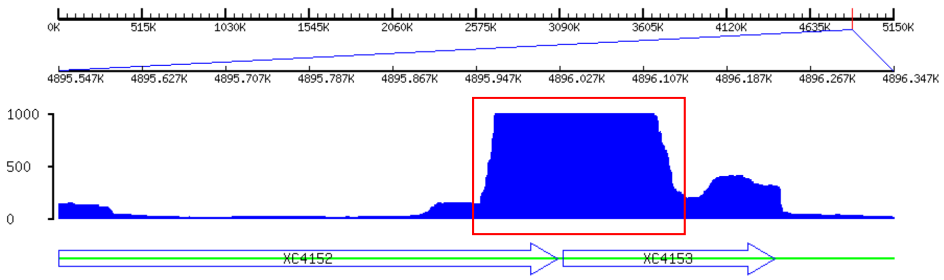

Transcript	Start	End	Length (bp)	RPKM	Inside gene	Overlap gene	Upstream gene	Downstream gene
TT661	4907646	4907735	90	67.1	XC4163	Null	XC4162	XC4164
TT662	4907779	4907870	92	64.1	XC4163	Null	XC4162	XC4164

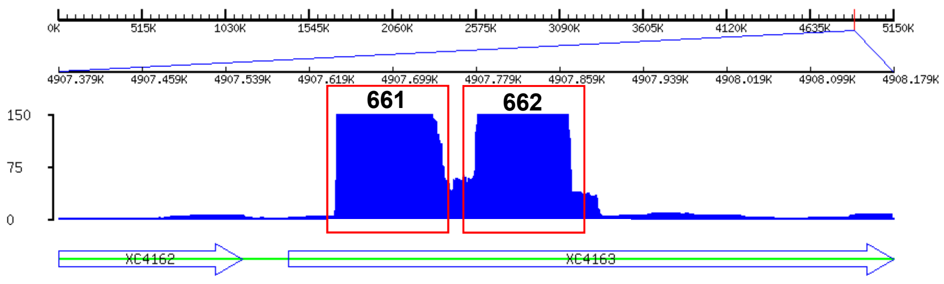

Transcript	Start	End	Length (bp)	RPKM	Inside gene	Overlap gene	Upstream gene	Downstream gene
TT663	4941041	4941255	215	161.8	XC4185	Null	XC4184	XC4186

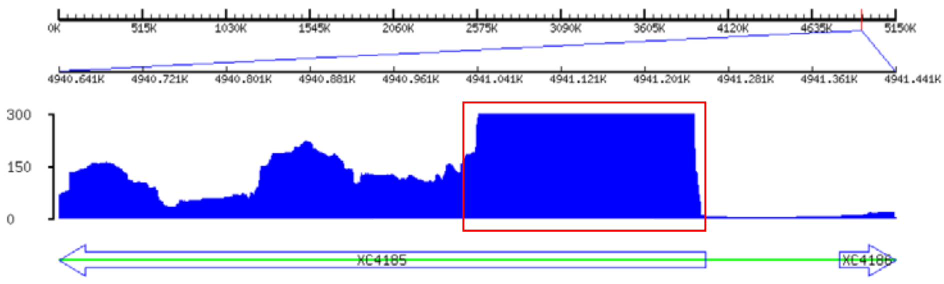

Transcript	Start	End	Length (bp)	RPKM	Inside gene	Overlap gene	Upstream gene	Downstream gene
TT664	4961230	4961296	67	130.4	Null	XC4199	XC4198	XC4200

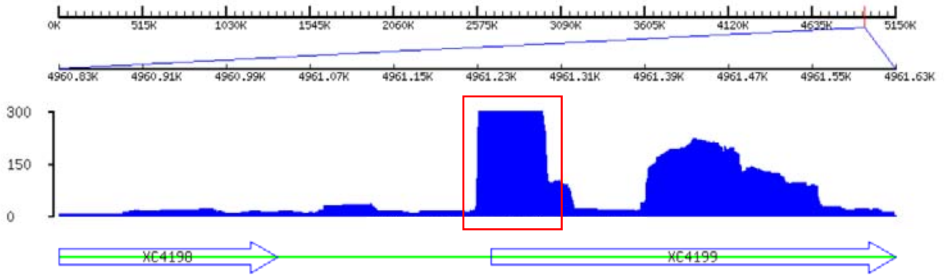

Transcript	Start	End	Length (bp)	RPKM	Inside gene	Overlap gene	Upstream gene	Downstream gene
TT665	4991506	4991585	80	13.1	Null	Null	XC4221	XC4222

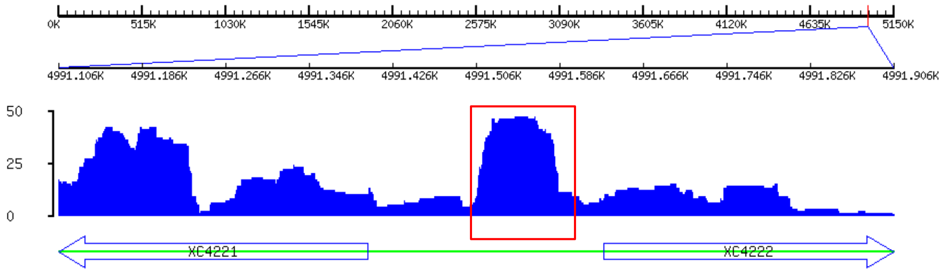

Transcript	Start	End	Length (bp)	RPKM	Inside gene	Overlap gene	Upstream gene	Downstream gene
TT666	4995813	4995868	56	101.5	XC4223	Null	XC4222	XC4224

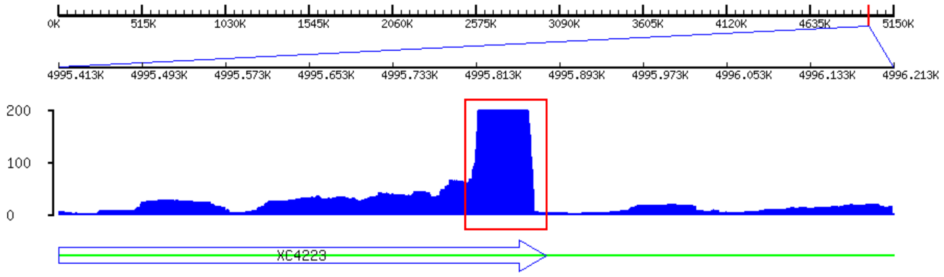

Transcript	Start	End	Length (bp)	RPKM	Inside gene	Overlap gene	Upstream gene	Downstream gene
TT667	5025143	5025415	272	1546.5	Null	Null	XC4393	XC4240

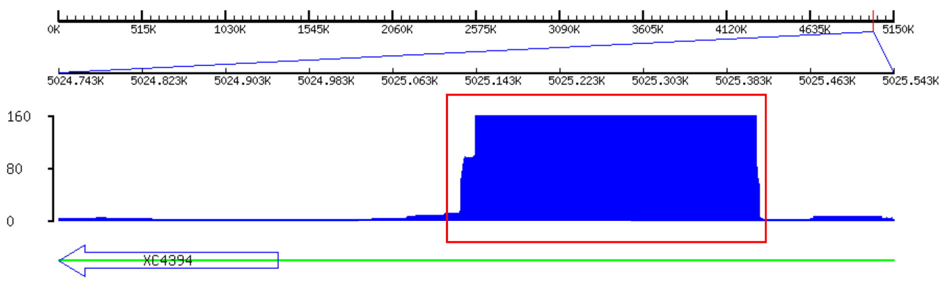

Transcript	Start	End	Length (bp)	RPKM	Inside gene	Overlap gene	Upstream gene	Downstream gene
TT668	5025323	5025415	93	4130.9	Null	Null	XC4393	XC4240

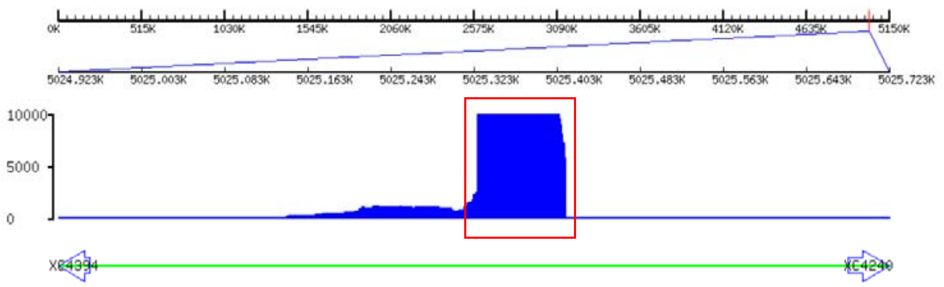

Transcript	Start	End	Length (bp)	RPKM	Inside gene	Overlap gene	Upstream gene	Downstream gene
TT669	5034040	5034162	123	74.7	Null	XC4245	XC4244	XC4246

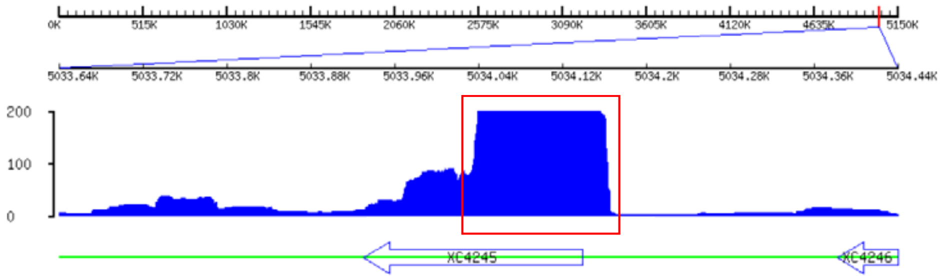

Transcript	Start	End	Length (bp)	RPKM	Inside gene	Overlap gene	Upstream gene	Downstream gene
TT670	5048477	5048580	104	93.1	XC4256	Null	XC4255	XC4257

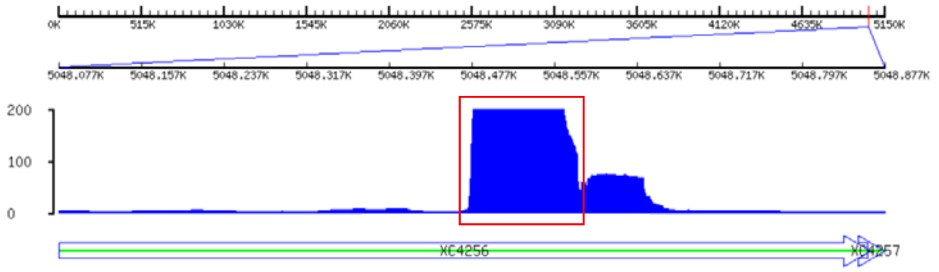

Transcript	Start	End	Length (bp)	RPKM	Inside gene	Overlap gene	Upstream gene	Downstream gene
TT671	5060296	5060361	66	46.3	XC4266	Null	XC4265	XC4267

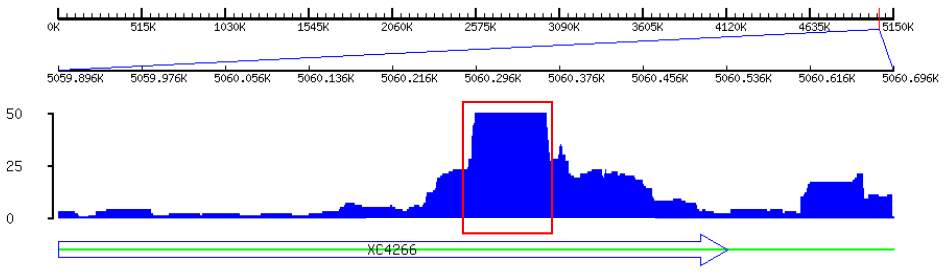

Transcript	Start	End	Length (bp)	RPKM	Inside gene	Overlap gene	Upstream gene	Downstream gene
TT672	5066546	5066635	90	577.7	XC4273	Null	XC4272	XC4274

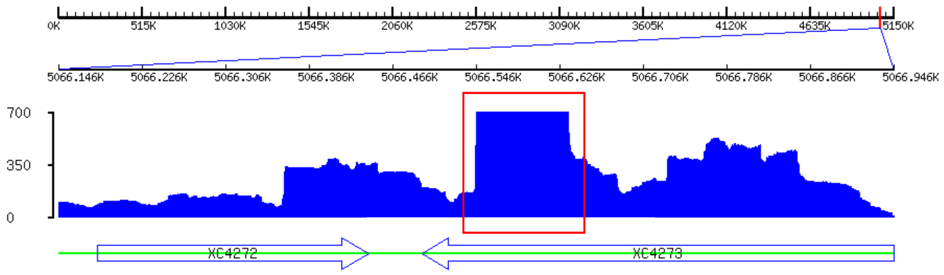

Transcript	Start	End	Length (bp)	RPKM	Inside gene	Overlap gene	Upstream gene	Downstream gene
TT673	5068506	5068612	107	110.9	Null	Null	XC4273	XC4274

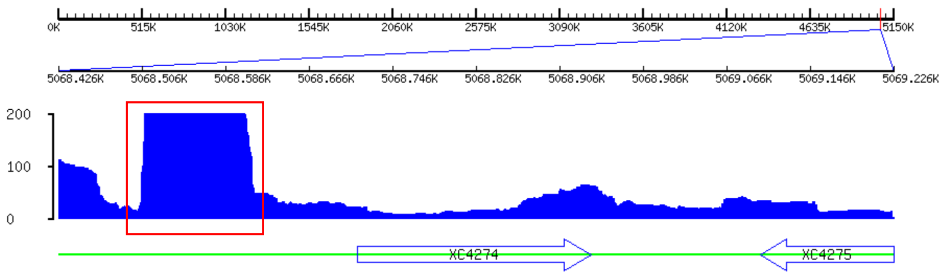

Transcript	Start	End	Length (bp)	RPKM	Inside gene	Overlap gene	Upstream gene	Downstream gene
TT674	5120450	5120510	61	137.9	Null	XC4313	XC4312	XC4314

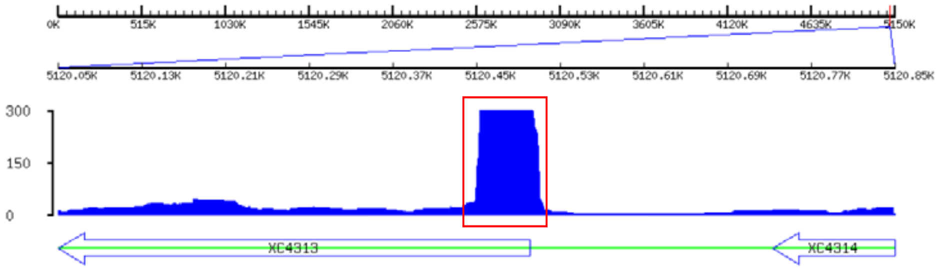

Transcript	Start	End	Length (bp)	RPKM	Inside gene	Overlap gene	Upstream gene	Downstream gene
TT675	5130309	5130381	73	109.8	Null	Null	XC4320	XC4321

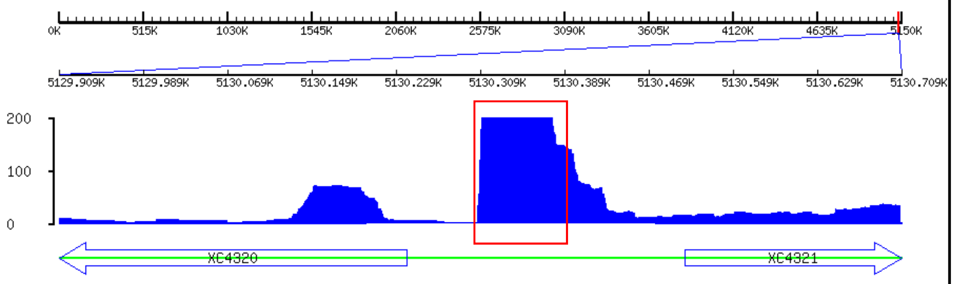

Transcript	Start	End	Length (bp)	RPKM	Inside gene	Overlap gene	Upstream gene	Downstream gene
TT676	5141724	5141917	194	33.3	Null	XC4327	XC4326	XC4328

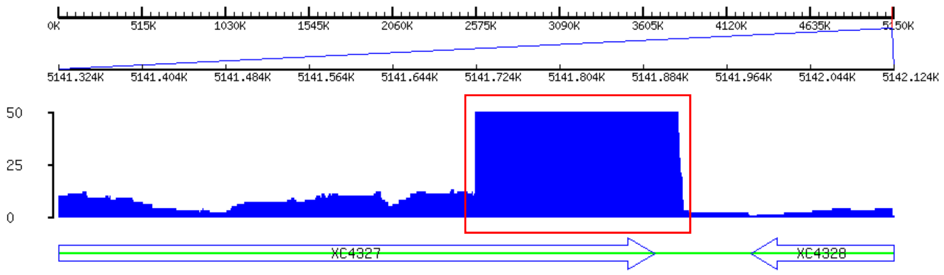

Supplement: Supplementary file 1 — FIGURE S1 The visualized mapping patterns of the 676 identified target transcripts (TTs). “Start” and “End” indicate the putative transcription start and end positions in the genome of Xcc strain 8,004. “Length” indicates the length of the TT. RPKM represents the value of the reads per kilobases per million reads. The vertical ordinate represents the number of mapped reads and the horizontal ordinate indicates the genome position and genetic organization of the mapped region [file MPP-21-1573-s001.pdf]
