# Supplementary material for: Genome‐wide screen and functional analysis in Xanthomonas reveal a large number of mRNA‐derived sRNAs, including the novel RsmA‐sequester RsmU
Source: Mol Plant Pathol. 2020 Sep 23;21(12):1573–90. doi: 10.1111/mpp.12997 (PMC7694677; doi:10.1111/mpp.12997)
Supplement: Supplementary file 2 — FIGURE S2 Sequence alignments of Xcc SRCs homologues using the Vector NTI program. The result reveals that the small RNAs SRC017/SRC434/SRC565 and SRC020/SRC291 are homologous [file MPP-21-1573-s002.pdf]

```
(1)   1        10         20         30         40         50         60         70              85
SRC017 (1) TGCCGGGAGGCTAGAAACCGCACACAGCCGGCGGGCTTATTCCCCTTGCGGGTGTTGTATTCGCCGC------------------
SRC343 (1) -GTCGGGAGGCTCGAAACCGCTAACAGCCGGCGGGCGTATTCCCCTTGCGGGTGTTGTATTAGCCGCCCTCCCGACGCAGAGCAT
SRC565 (1) TGTCGGGAGGTTCGAAACCGCAGAGAGCCGGCGGGCGTATTCCCCTTGCGGGTGTTGTATTAGCCG-------------------

(86)   86            100        110        120        130        140        150                169
SRC017 (68) ------------------------------------------------------------------------------------
SRC343 (85) CACGTCGGTTGCACCCACAAAAGTTGCAGGCACAAAAAACCCACCGGTTTGTCGGAGGTGGGTACCGCTGTTAGCGGAGTTTCG
SRC565 (67) ------------------------------------------------------------------------------------
```

```
(1)   1        10         20         30         40         50         60         70         80         90              107
SRC020 (1) ---------AGGTCCCGCGACGGTGAACGGGCAAGGACCAGTGGAGAGTGTCGGTTTGCGTGG-TGCAAGCAGTGCATGACGTGCGTTGTTCGGATAACGGCGCGTC
SRC291 (1) TGCCGCGTAAGGTCCCGCGACAGTGAGCGGGCAAGGACCAGTGGAGATGGTCGGTGTGCATGGCTATCAACAAAGCC-GCCA--------------------------

(108)  108         120        130        140        150        160        170        180        190        200          213
SRC020 (98) CGATCAGTGTCGCAGGGCAAACCGAGTAACACGCAGGCTTTCAACAAAGCAACCGACTCACTTTCTGGTGCGGTGTCCTCGCCGATTGCGGGACCGTGTGGCGGCA
SRC291 (82) ----------------------------------------------------------------------------------------------------------
```

Fig. S2. Sequence alignments of *Xcc* SRCs homologues using the Vector NTI program. The result reveals that the small RNAs SRC017/SRC434/SRC565, and SRC020/SRC291 are homologous, respectively.
