# Supplementary material for: Genome‐wide screen and functional analysis in Xanthomonas reveal a large number of mRNA‐derived sRNAs, including the novel RsmA‐sequester RsmU
Source: Mol Plant Pathol. 2020 Sep 23;21(12):1573–90. doi: 10.1111/mpp.12997 (PMC7694677; doi:10.1111/mpp.12997)

Fig. S3. Detection of the expression of sRNAs in *Xcc* by Northern blotting. The bacterial cells of *Xcc* wild type strain 8004 was cultured in the minimal medium MMX to mi-log phase and total RNAs were isolated from the cells. 3 μg total RNA was separated by PAGE and transferred to a positively charged nylon membrane. After UV-crosslinking, the membrane was hybridized with a DIG-labelled RNA probe at 68 ^{o}C for 8 hours and then signal bands were detected. To ensure the accuracy in evaluation of the size of signal bands, DIG-labeled RNA molecular weight marker (M) was loaded in each PAGE gel. Filled triangle inside the Northern blotting result picture indicates the position corresponding to the size of the target SRC predicted by RNA-seq. Above the Northern blotting result picture is the visualized mapping pattern of the corresponding SRC. Pink arrow indicates the transcriptional direction of the SRC. The lowermost arrow indicates protein-coding gene and its transcriptional orientation and the gene's ID is shown inside the arrow. The Y-axis represents the number of the mapped reads. RPKM, reads per kilo bases per million reads

sRNA name	sRNA candidate	Target Transcript	Start	End	Length (nt)	RPKM
sRX001	SRC001	TT001	1	115	115	148.1

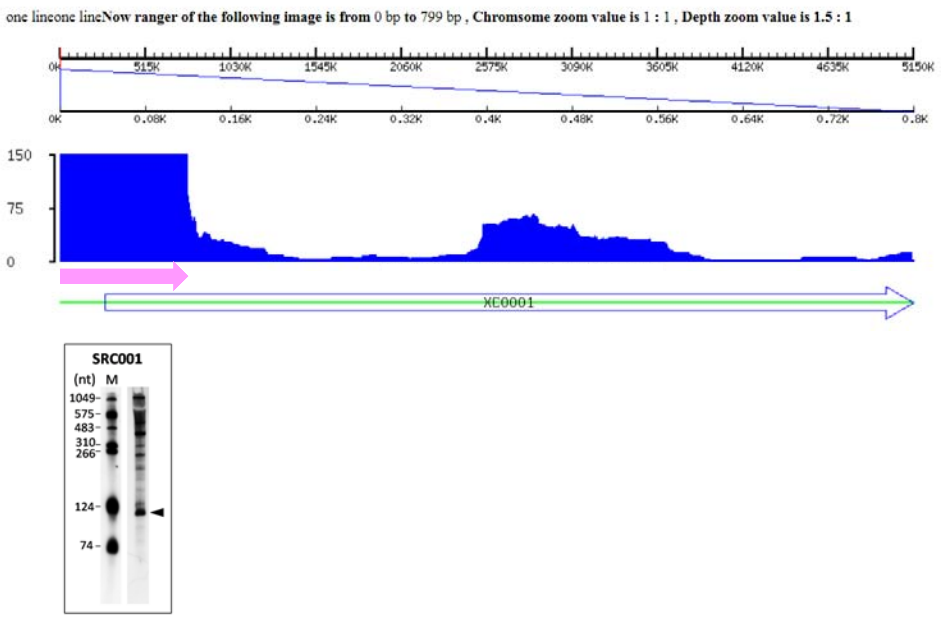

sRNA name	sRNA candidate	Target Transcript	Start	End	Length (nt)	RPKM
sRX002	SRC002	TT002	3605	3701	97	263.6

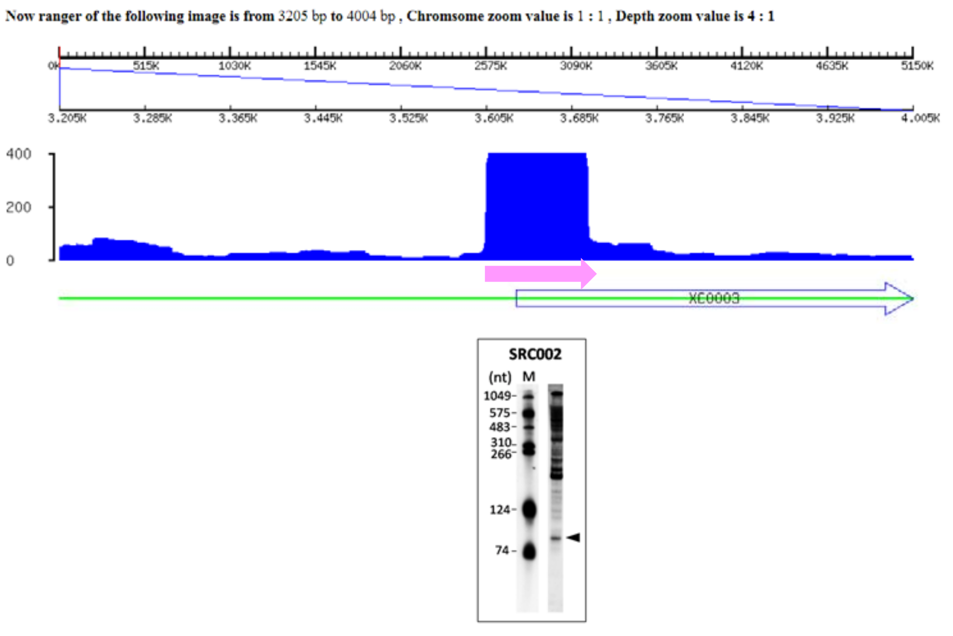

sRNA name	sRNA candidate	Target Transcript	Start	End	Length (nt)	RPKM
sRX003	SRC003	TT003	8254	8318	65	201

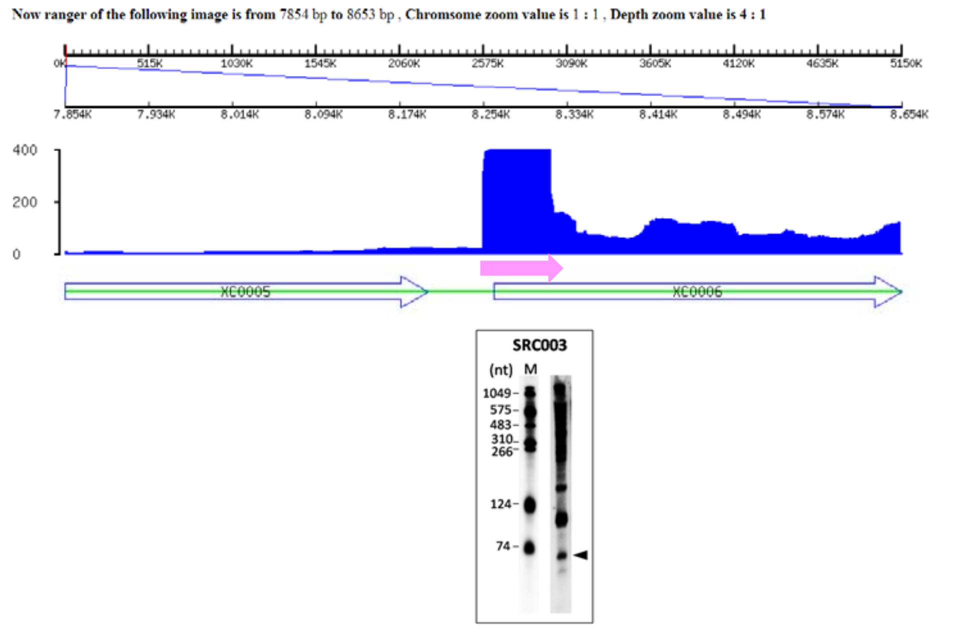

sRNA name	sRNA candidate	Target Transcript	Start	End	Length (nt)	RPKM
sRX004	SRC005	TT005	11540	11606	67	100.3

one lineone lineNow ranger of the following image is from 11140 bp to 11939 bp , Chromsome zoom value is 1 : 1 , Depth zoom value is 2.5 : 1

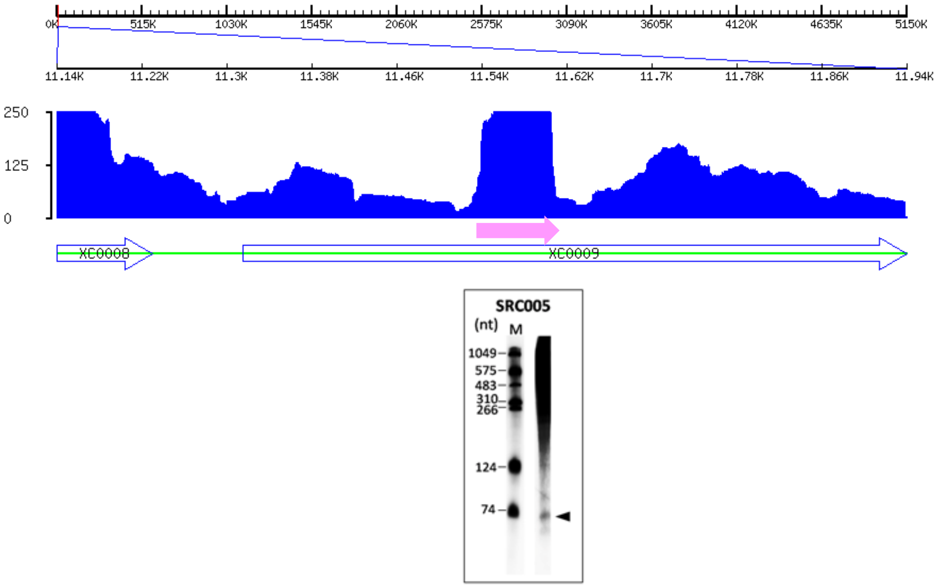

sRNA name	sRNA candidate	Target Transcript	Start	End	Length (nt)	RPKM
sRX005	SRC007	TT007	19739	19801	63	45.7
sRX006	SRC008	TT008	20127	20185	59	80.5

Now ranger of the following image is from 19419 bp to 20218 bp , Chromsome zoom value is 1 : 1 , Depth zoom value is 2 : 1

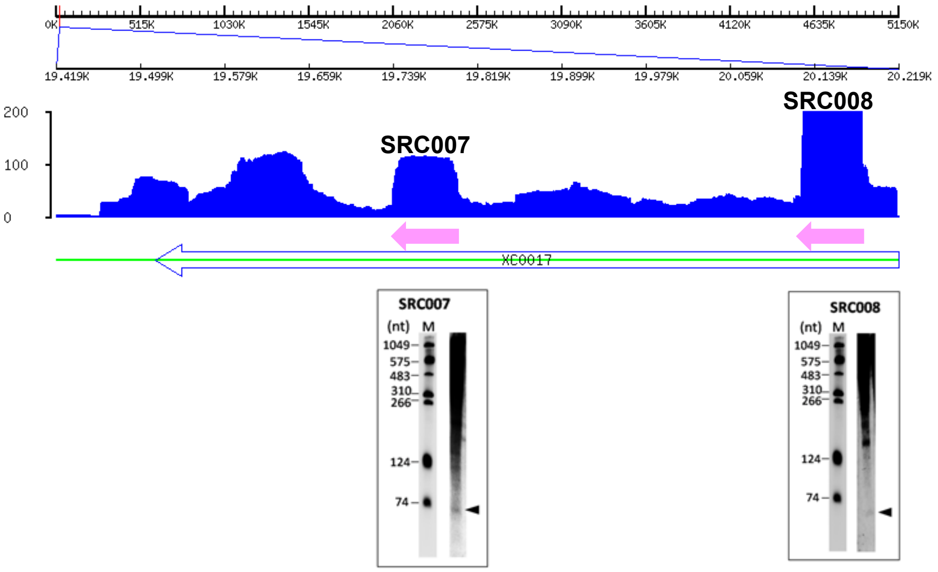

sRNA name	sRNA candidate	Target Transcript	Start	End	Length (nt)	RPKM
sRX007	SRC009	TT009	44065	44146	82	212.9

Now ranger of the following image is from 43665 bp to 44464 bp , Chromsome zoom value is 1 : 1 , Depth zoom value is 5 : 1

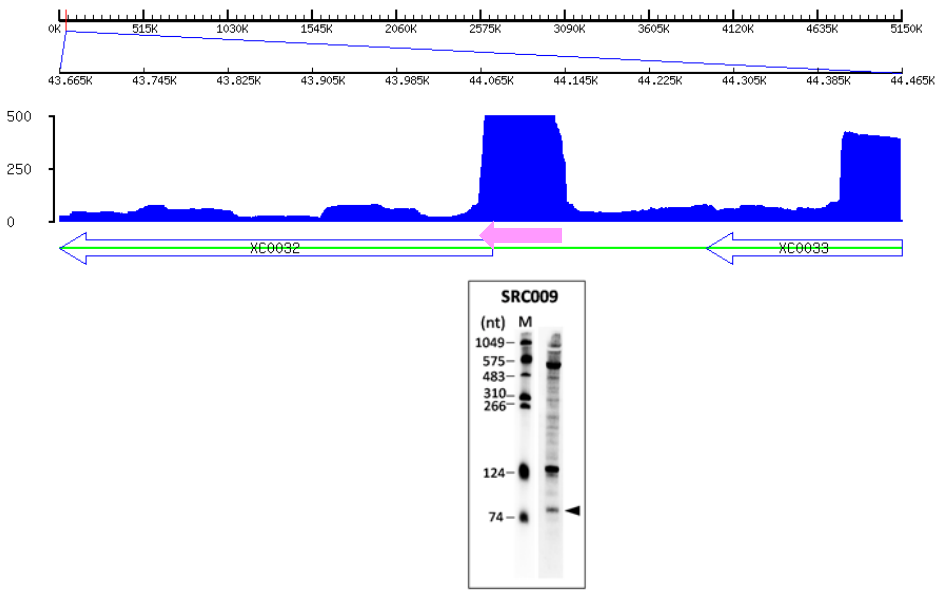

sRNA name	sRNA candidate	Target Transcript	Start	End	Length (nt)	RPKM
sRX008	SRC011	TT011	73643	73693	51	117.4

Now ranger of the following image is from 73243 bp to 74042 bp , Chromsome zoom value is 1 : 1 , Depth zoom value is 2 : 1

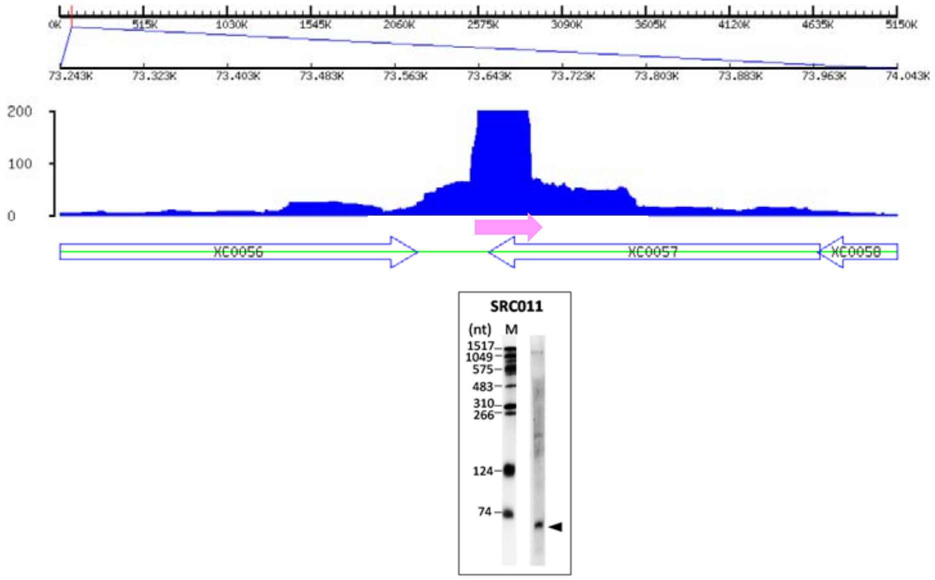

sRNA name	sRNA candidate	Target Transcript	Start	End	Length (nt)	RPKM
sRX009	SRC017	TT017	83883	83949	67	389.9

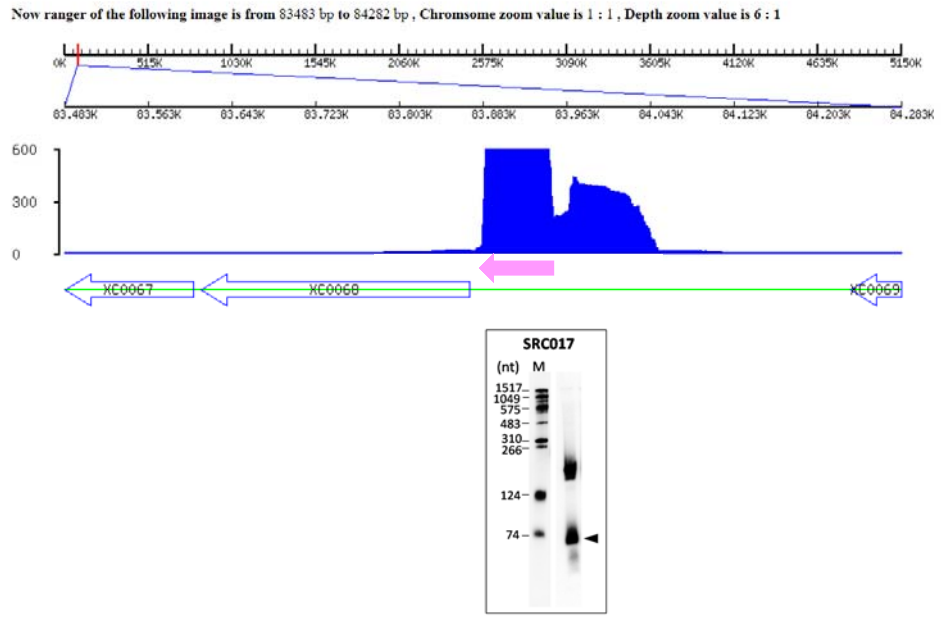

sRNA name	sRNA candidate	Target Transcript	Start	End	Length (nt)	RPKM
sRX010	SRC022	TT022	142449	142507	59	75.2

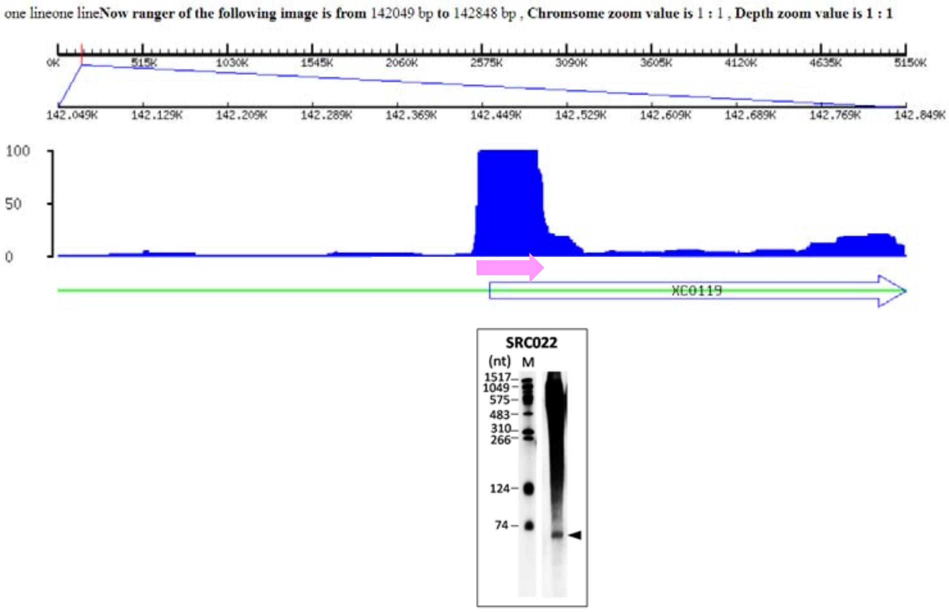

sRNA name	sRNA candidate	Target Transcript	Start	End	Length (nt)	RPKM
sRX011	SRC024	TT024	165210	165280	71	61.1

Now ranger of the following image is from 164810 bp to 165609 bp , Chromsome zoom value is 1 : 1 , Depth zoom value is 1 : 1

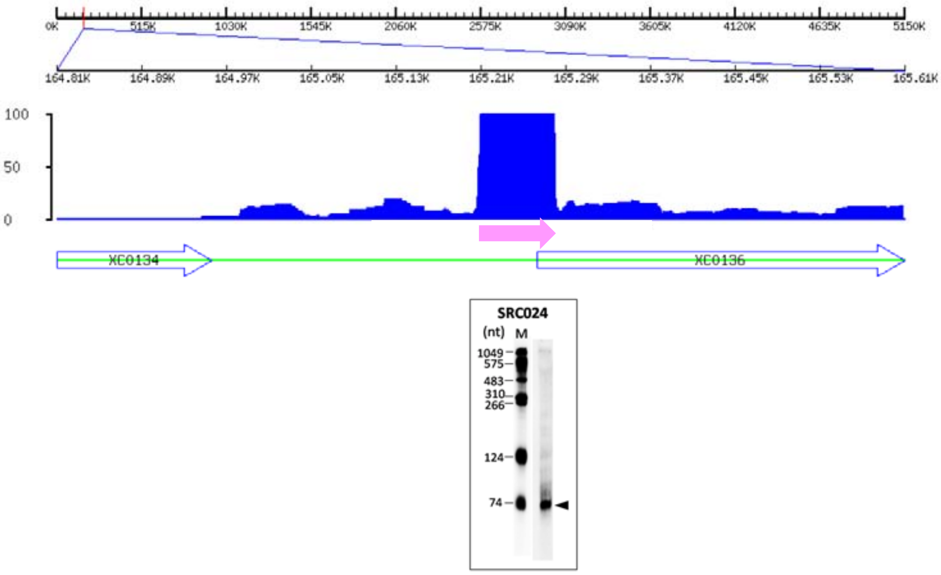

sRNA name	sRNA candidate	Target Transcript	Start	End	Length (nt)	RPKM
sRX012	SRC028	TT028	225413	225476	64	109.8

one lineone lineNow ranger of the following image is from 225013 bp to 225812 bp , Chromsome zoom value is 1 : 1 , Depth zoom value is 2.5 : 1

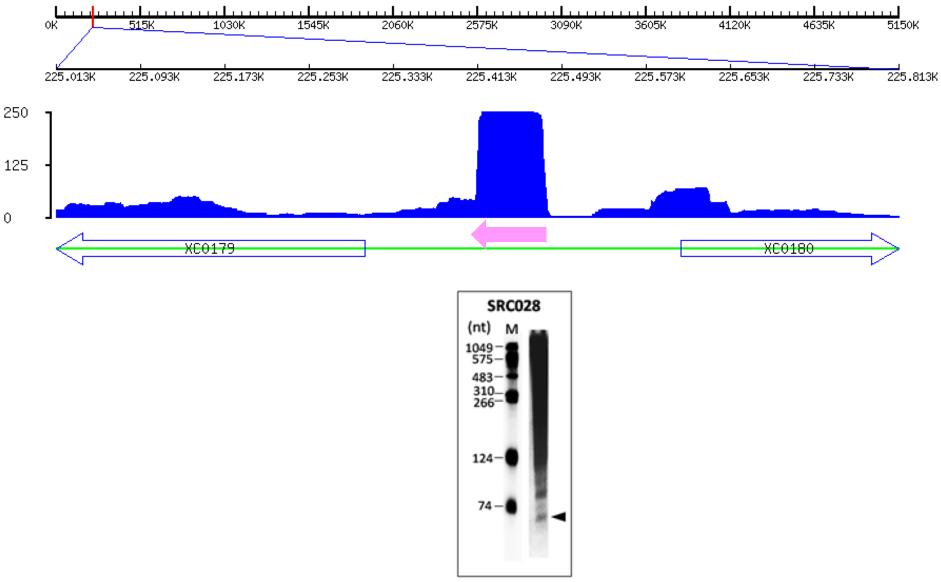

sRNA name	sRNA candidate	Target Transcript	Start	End	Length (nt)	RPKM
sRX013	SRC029	TT029	230567	230759	193	170.9

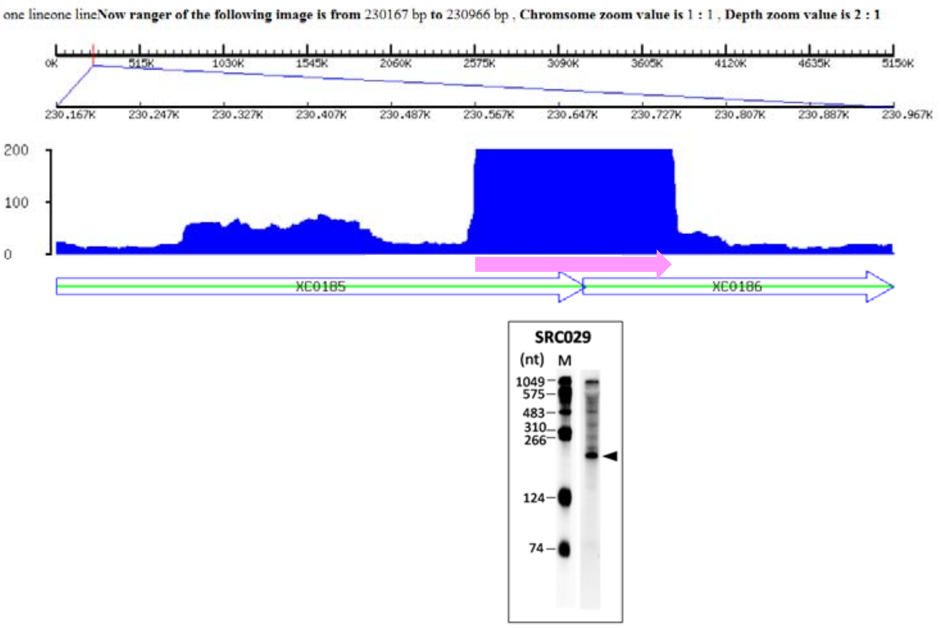

sRNA name	sRNA candidate	Target Transcript	Start	End	Length (nt)	RPKM
sRX014	SRC030	TT030	239805	239882	78	42.7

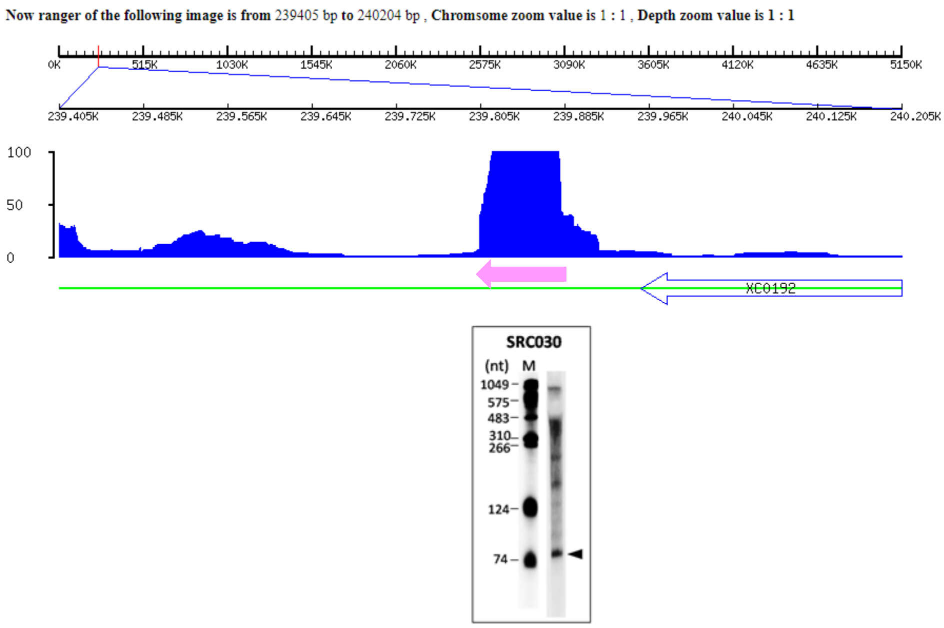

sRNA name	sRNA candidate	Target Transcript	Start	End	Length (nt)	RPKM
sRX015	SRC035	TT037	292444	292545	102	360.4

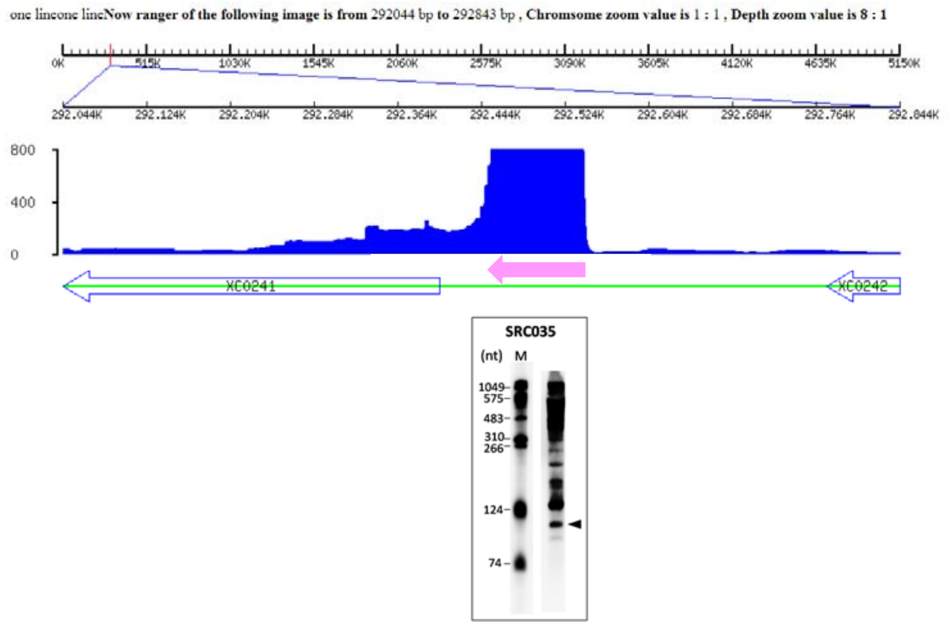

sRNA name	sRNA candidate	Target Transcript	Start	End	Length (nt)	RPKM
sRX016	SRC037	TT039	328623	328686	64	1243.9

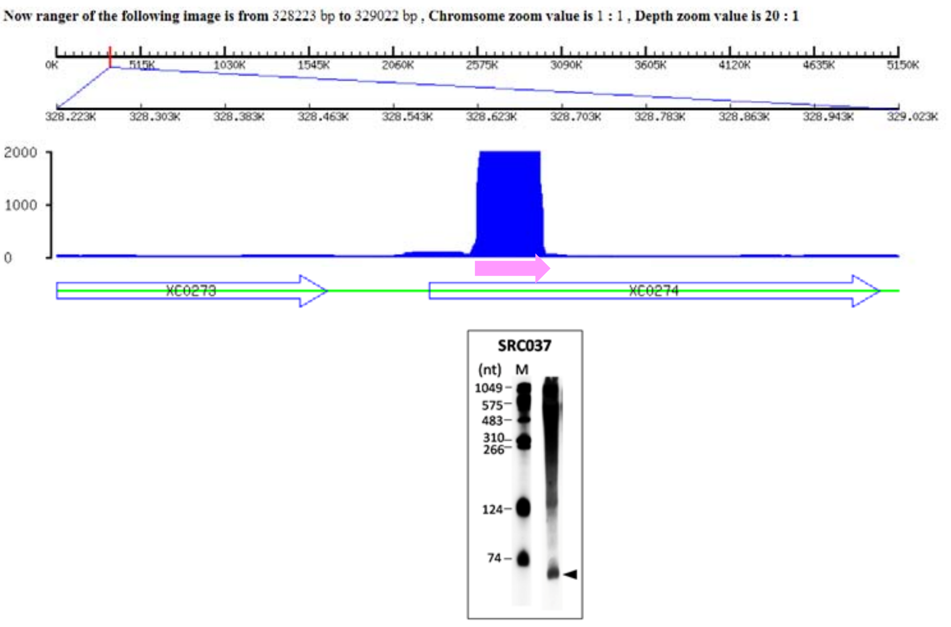

sRNA name	sRNA candidate	Target Transcript	Start	End	Length (nt)	RPKM
sRX017	SRC042	TT045	346558	346643	86	100.7

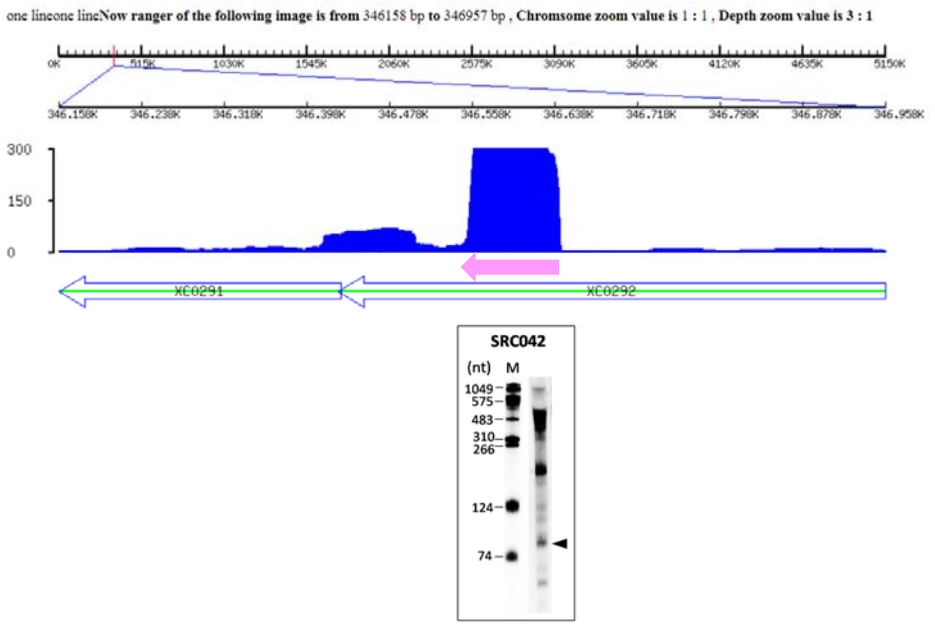

sRNA name	sRNA candidate	Target Transcript	Start	End	Length (nt)	RPKM
sRX018	SRC048	TT051	395405	395472	68	2288.1

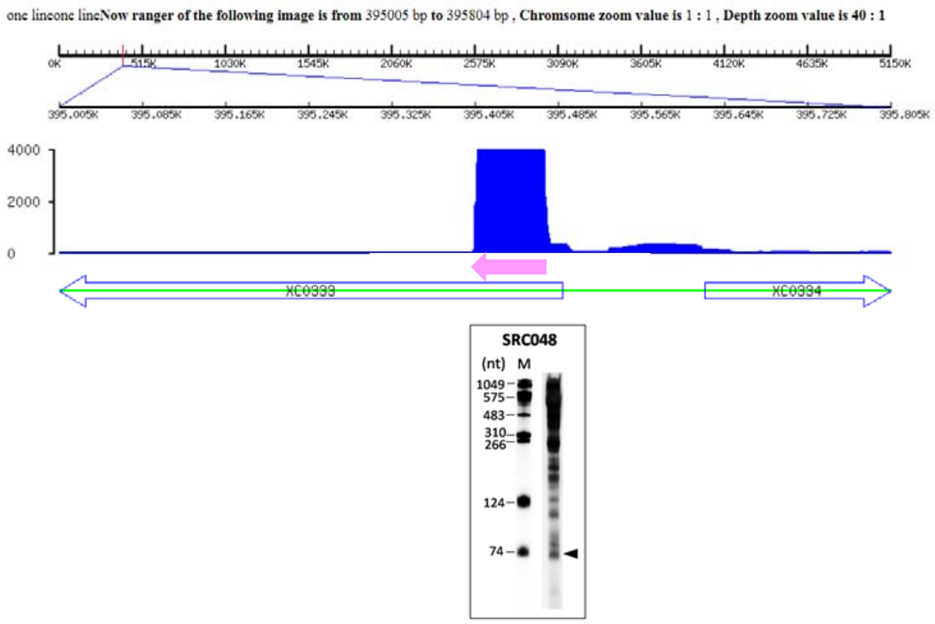

sRNA name	sRNA candidate	Target Transcript	Start	End	Length (nt)	RPKM
sRX019	SRC052	TT056	419488	419557	70	158.5

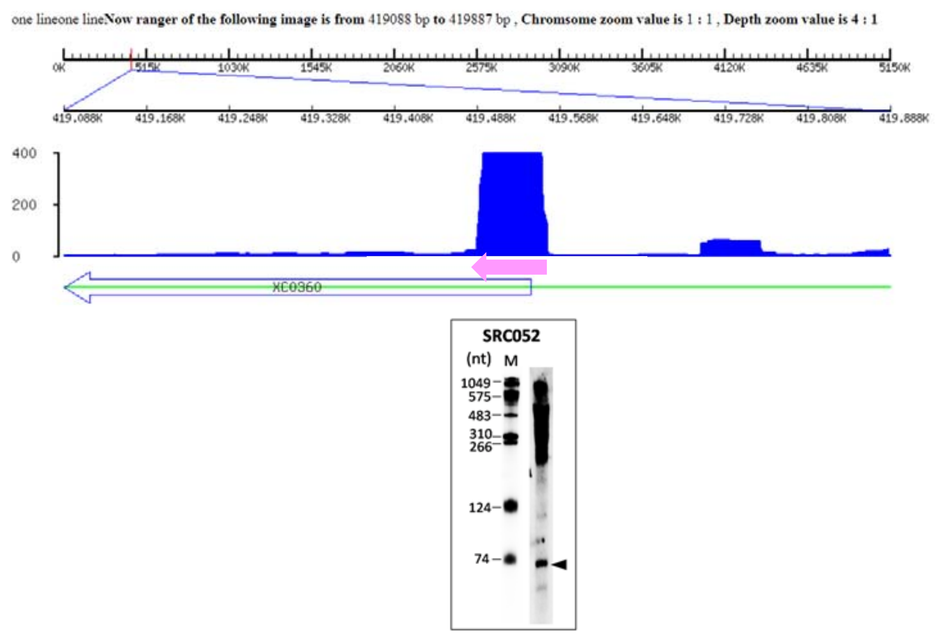

sRNA name	sRNA candidate	Target Transcript	Start	End	Length (nt)	RPKM
sRX020	SRC053	TT057	447714	447766	53	276.5

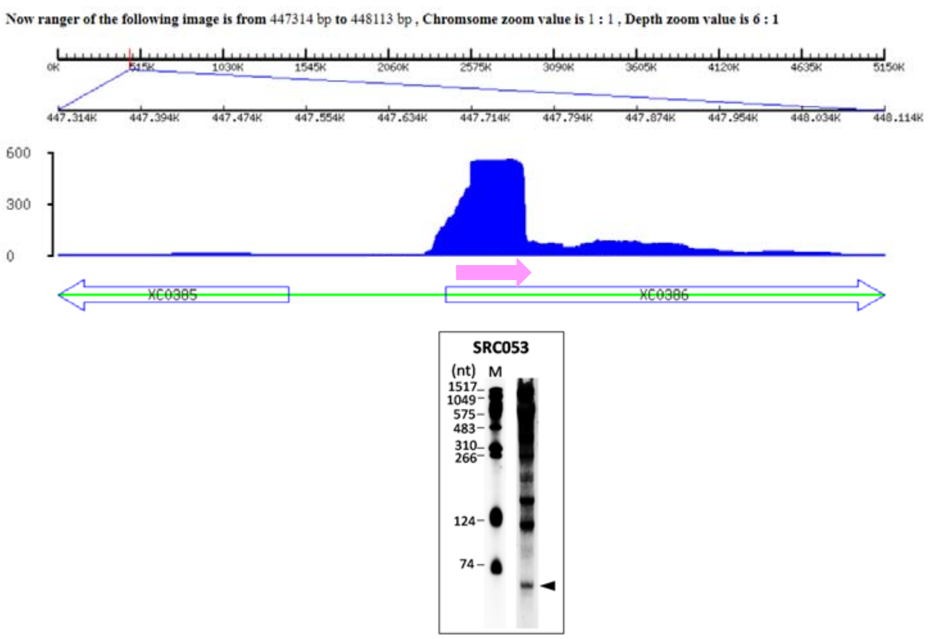

sRNA name	sRNA candidate	Target Transcript	Start	End	Length (nt)	RPKM
sRX021	SRC058	TT063	480582	480641	60	2317.6

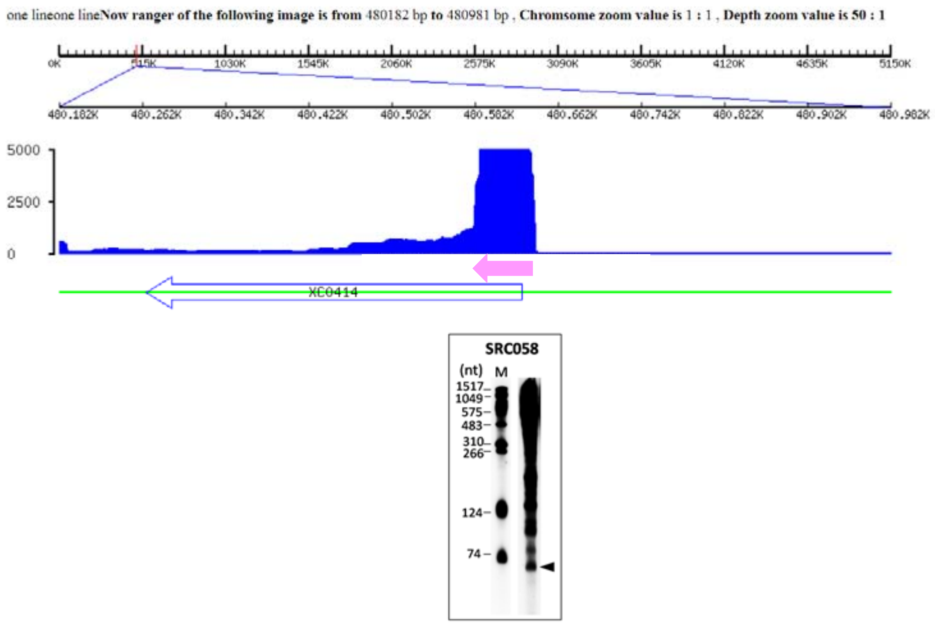

sRNA name	sRNA candidate	Target Transcript	Start	End	Length (nt)	RPKM
sRX022	SRC059	TT064	486775	486840	66	105.1

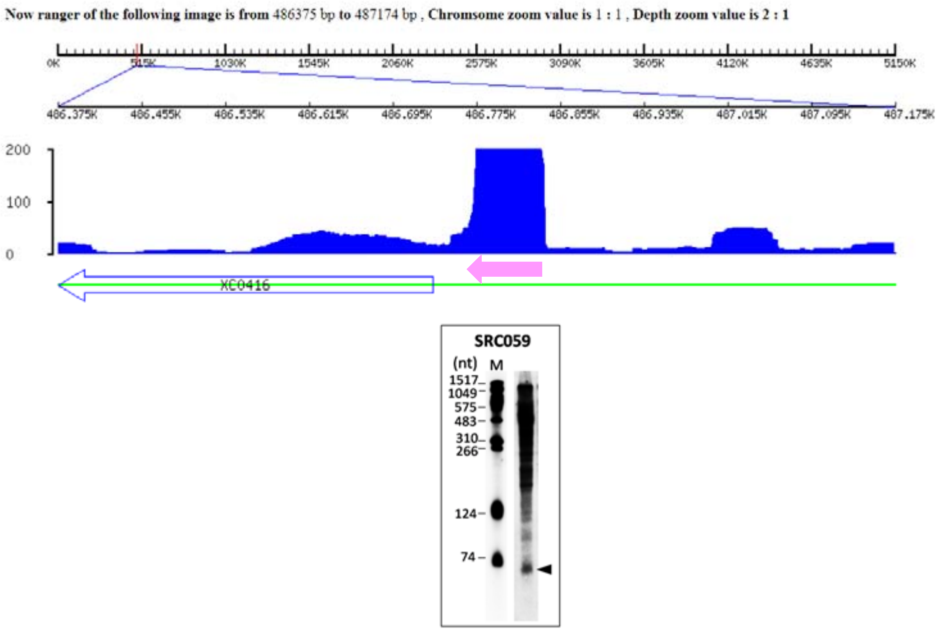

sRNA name	sRNA candidate	Target Transcript	Start	End	Length (nt)	RPKM
	SRC065	TT070	526278	526332	55	76.2

Now ranger of the following image is from 525878 bp to 526677 bp , Chromsome zoom value is 1 : 1 , Depth zoom value is 1 : 1

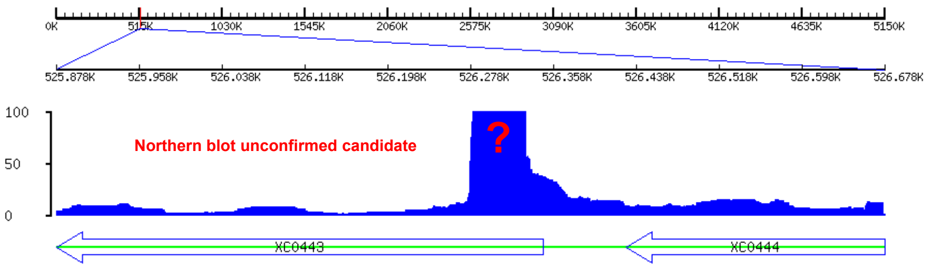

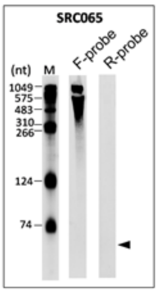

sRNA name	sRNA candidate	Target Transcript	Start	End	Length (nt)	RPKM
sRX023	SRC067	TT072	574674	574724	51	88.9

Now ranger of the following image is from 574274 bp to 575073 bp , Chromsome zoom value is 1 : 1 , Depth zoom value is 2 : 1

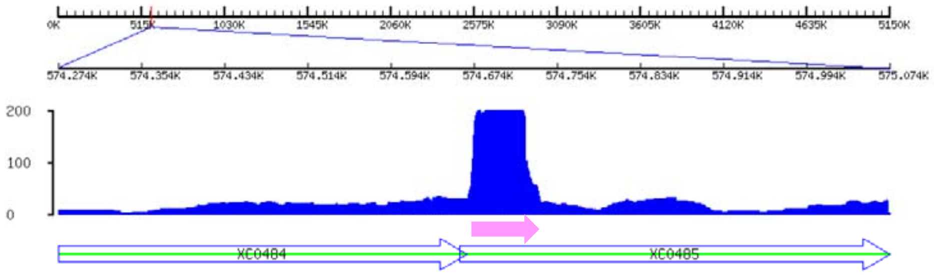

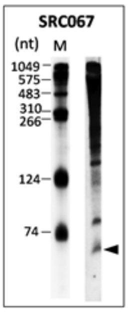

sRNA name	sRNA candidate	Target Transcript	Start	End	Length (nt)	RPKM
sRX024	SRC069	TT074	578891	578983	93	1286.2

Now ranger of the following image is from 578491 bp to 579290 bp , Chromsome zoom value is 1 : 1 , Depth zoom value is 30 : 1

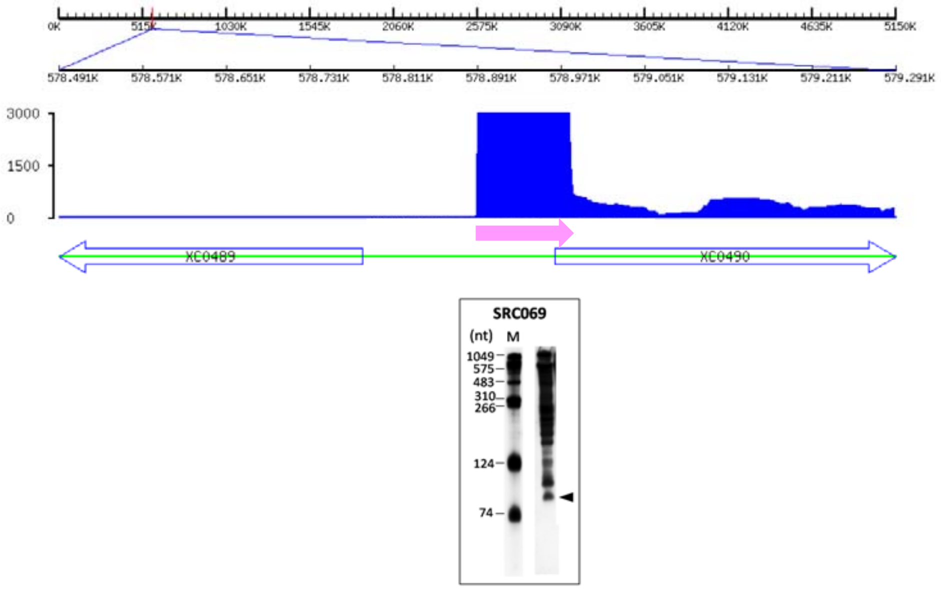

sRNA name	sRNA candidate	Target Transcript	Start	End	Length (nt)	RPKM
sRX025	SRC072	TT079	621291	621369	79	71.1

Now ranger of the following image is from 620891 bp to 621690 bp , Chromsome zoom value is 1 : 1 , Depth zoom value is 1.7 : 1

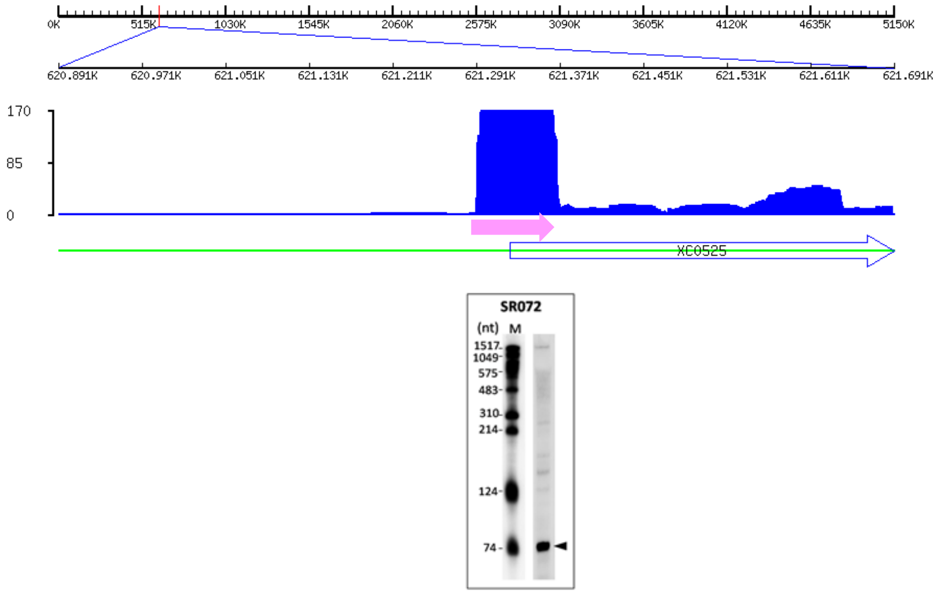

sRNA name	sRNA candidate	Target Transcript	Start	End	Length (nt)	RPKM
sRX026	SRC073	TT080	624944	625033	90	42.6

Now ranger of the following image is from 624544 bp to 625343 bp , Chromsome zoom value is 1 : 1 , Depth zoom value is 1 : 1

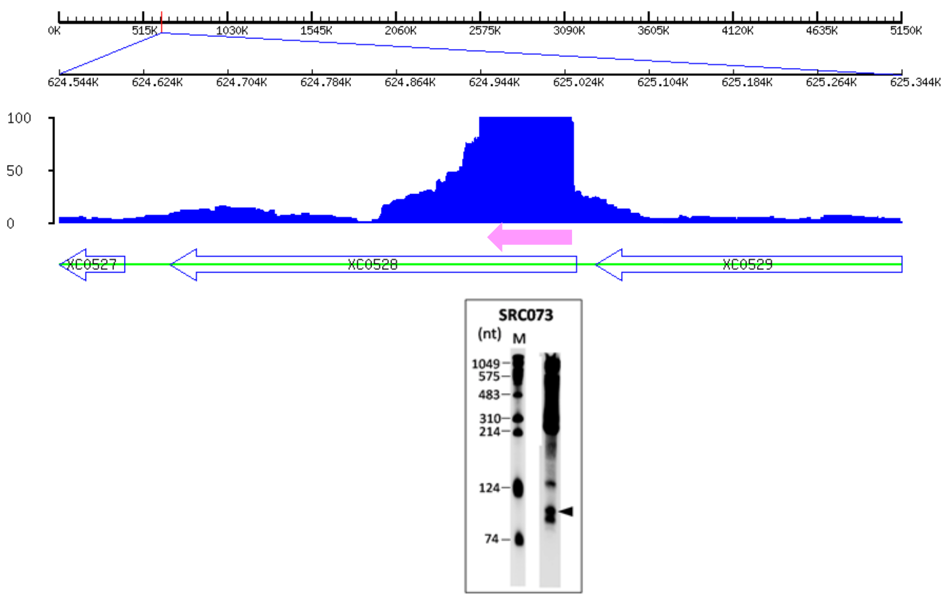

sRNA name	sRNA candidate	Target Transcript	Start	End	Length (nt)	RPKM
sRX027	SRC080	TT087	677097	677186	90	421.1

Now ranger of the following image is from 676697 bp to 677496 bp , Chromsome zoom value is 1 : 1 , Depth zoom value is 12 : 1

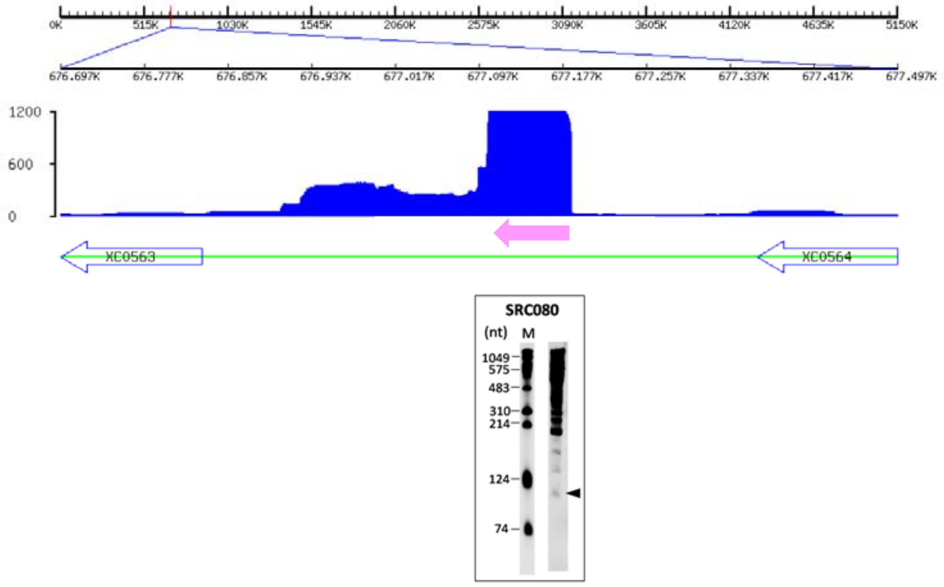

sRNA name	sRNA candidate	Target Transcript	Start	End	Length (nt)	RPKM
sRX028	SRC082	TT089	704129	704202	74	261.9

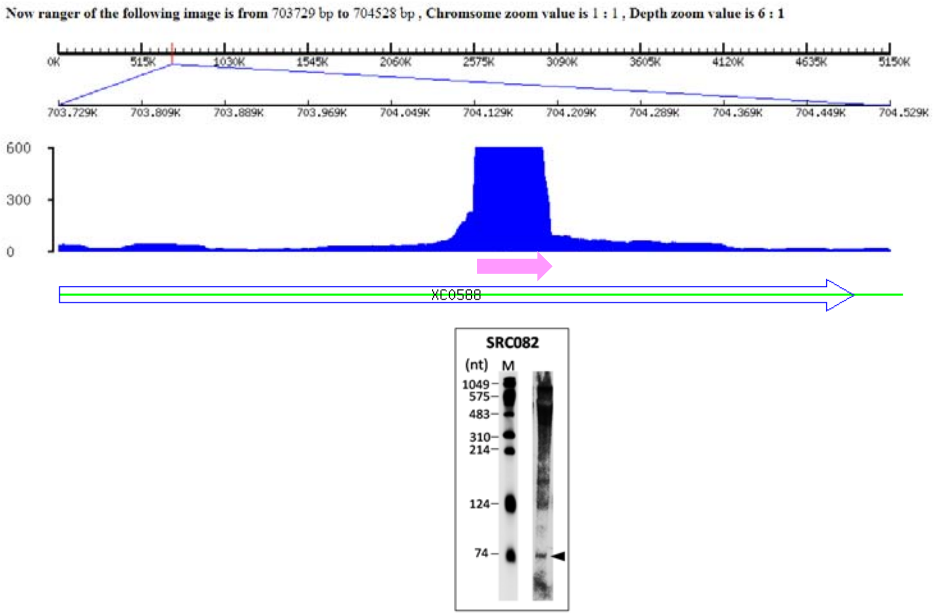

sRNA name	sRNA candidate	Target Transcript	Start	End	Length (nt)	RPKM
sRX029	SRC083	TT090	704921	705070	150	295.1

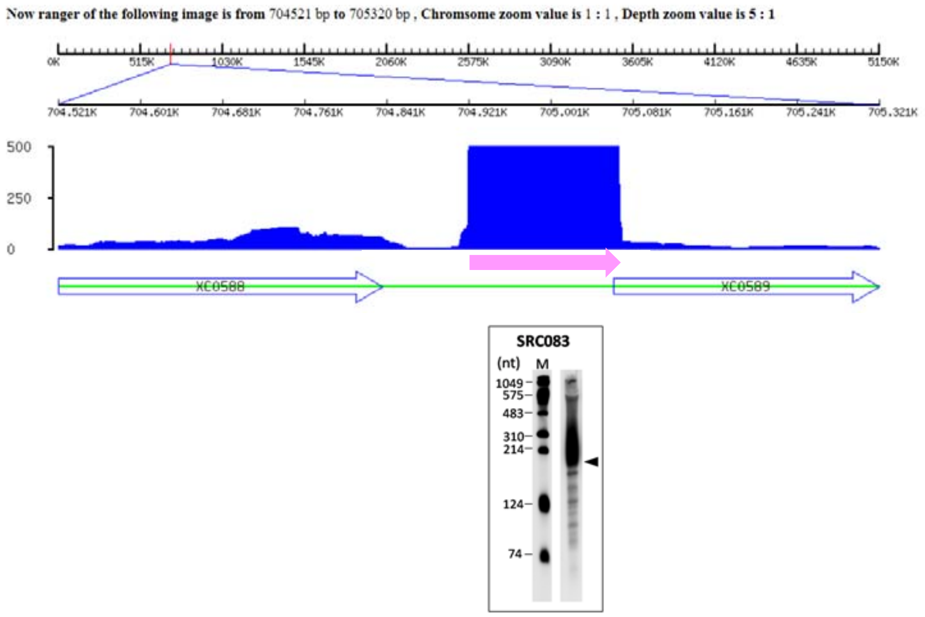

sRNA name	sRNA candidate	Target Transcript	Start	End	Length (nt)	RPKM
sRX030	SRC086	TT093	766606	766671	66	299.9

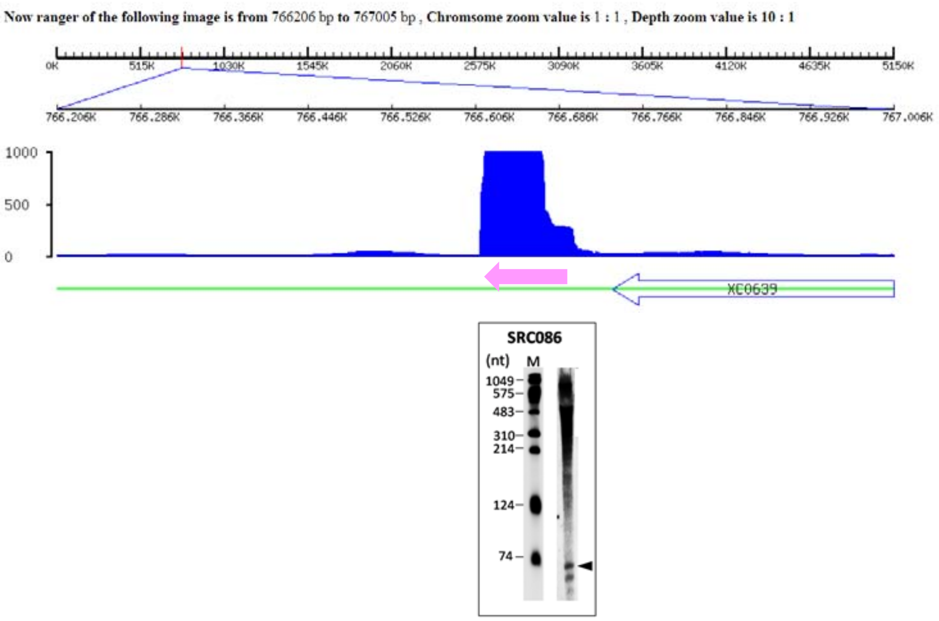

sRNA name	sRNA candidate	Target Transcript	Start	End	Length (nt)	RPKM
sRX031	SRC089	TT096	781908	781975	68	69.4

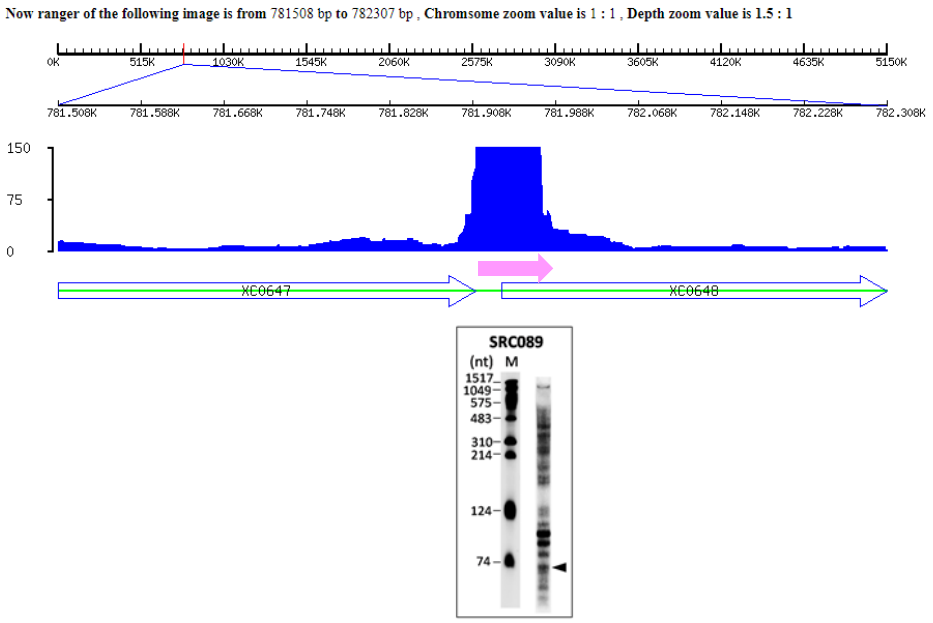

sRNA name	sRNA candidate	Target Transcript	Start	End	Length (nt)	RPKM
sRX032	SRC093	TT100	830789	830861	73	253.9

Now ranger of the following image is from 830389 bp to 831188 bp , Chromsome zoom value is 1 : 1 , Depth zoom value is 7 : 1

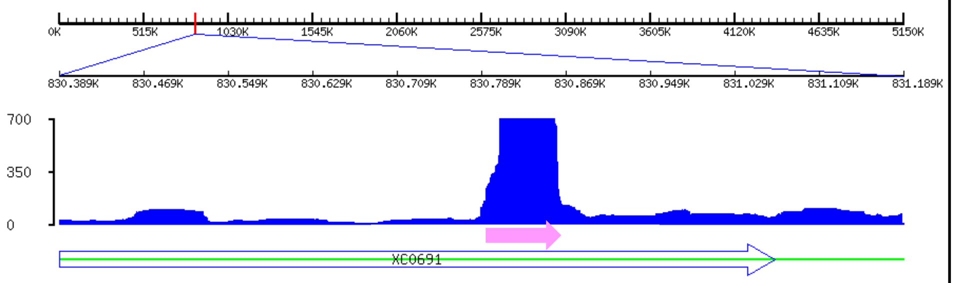

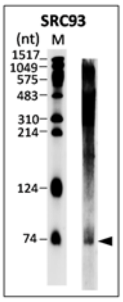

sRNA name	sRNA candidate	Target Transcript	Start	End	Length (nt)	RPKM
sRX033	SRC094	TT101	848204	848259	56	274.8

Now ranger of the following image is from 847804 bp to 848603 bp , Chromsome zoom value is 1 : 1 , Depth zoom value is 5 : 1

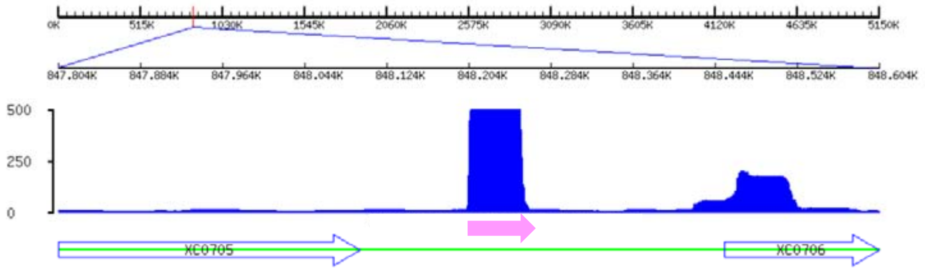

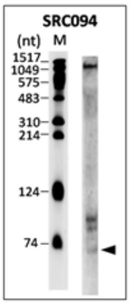

sRNA name	sRNA candidate	Target Transcript	Start	End	Length (nt)	RPKM
	SRC096	TT103	849532	849588	57	155.5

one lineone lineNow ranger of the following image is from 849132 bp to 849931 bp , Chromsome zoom value is 1 : 1 , Depth zoom value is 2.5 : 1

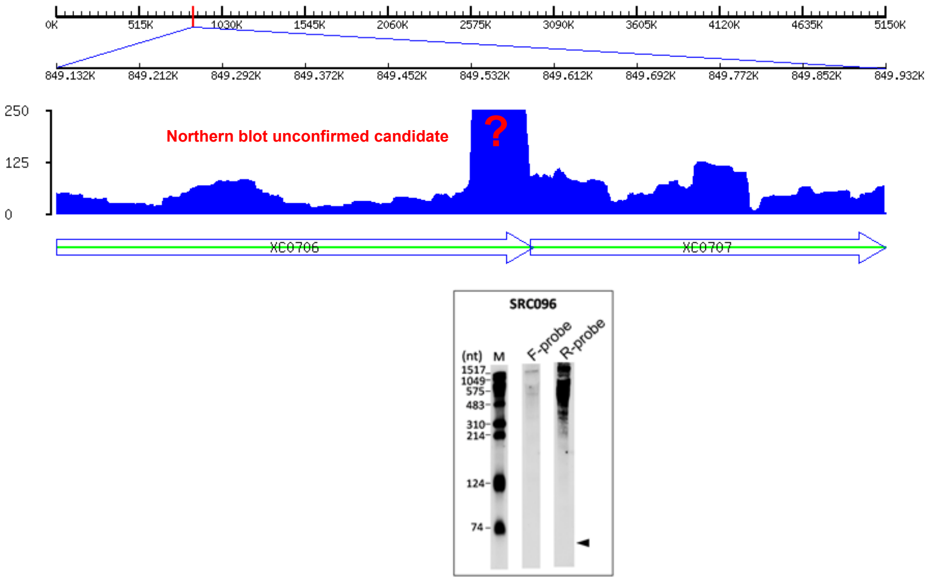

sRNA name	sRNA candidate	Target Transcript	Start	End	Length (nt)	RPKM
sRX034	SRC098	TT105	853703	853786	84	114.6

Now ranger of the following image is from 853251 bp to 854050 bp , Chromsome zoom value is 1 : 1 , Depth zoom value is 3 : 1

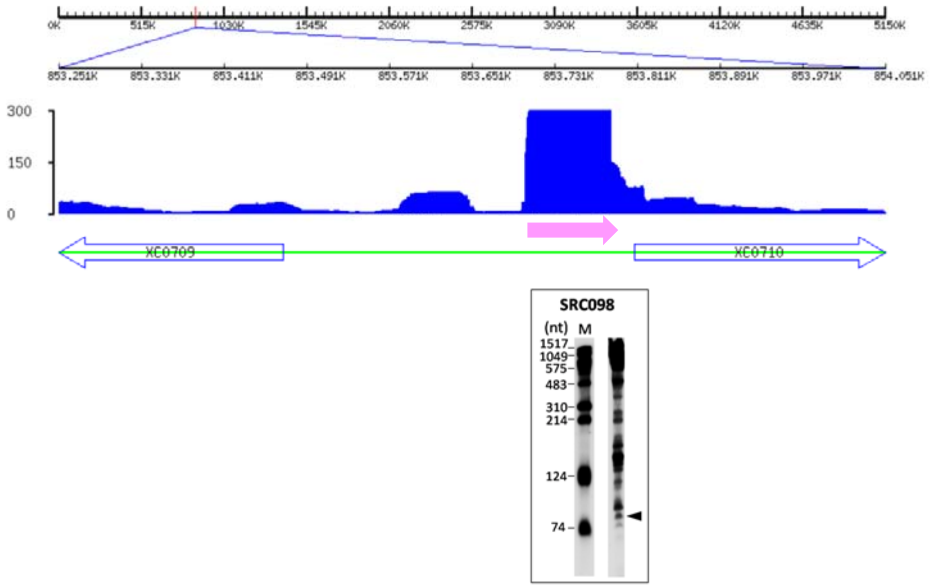

sRNA name	sRNA candidate	Target Transcript	Start	End	Length (nt)	RPKM
sRX035	SRC101	TT108	857025	857080	56	810.9

Now ranger of the following image is from 856625 bp to 857424 bp , Chromsome zoom value is 1 : 1 , Depth zoom value is 15 : 1

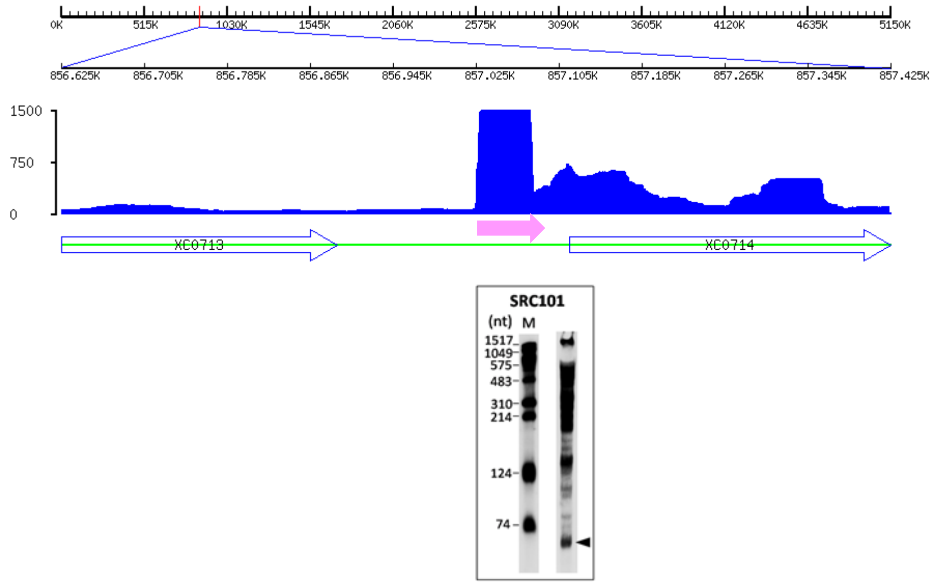

sRNA name	sRNA candidate	Target Transcript	Start	End	Length (nt)	RPKM
sRX036	SRC102	TT109	859744	859833	90	38.1

Now ranger of the following image is from 859344 bp to 860143 bp , Chromsome zoom value is 1 : 1 , Depth zoom value is 1 : 1

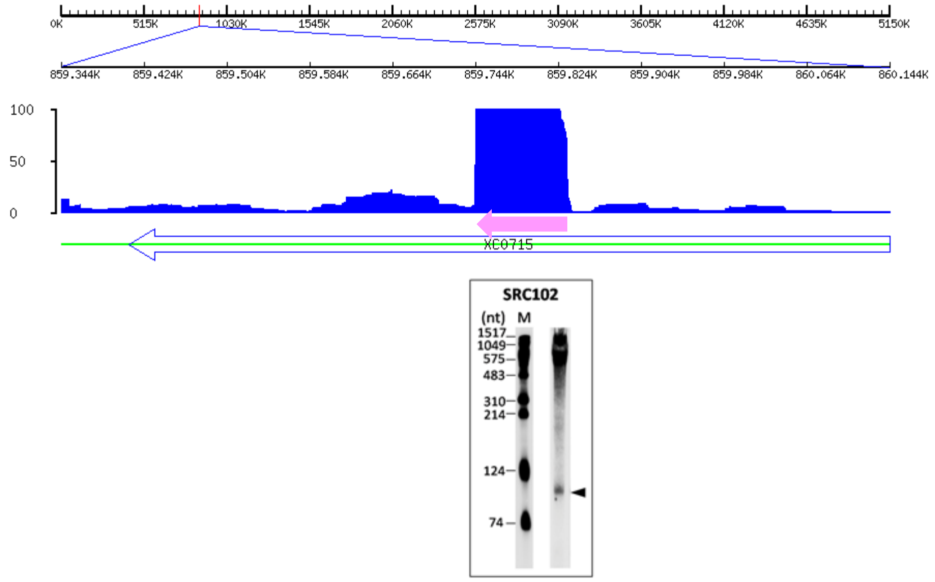

sRNA name	sRNA candidate	Target Transcript	Start	End	Length (nt)	RPKM
sRX037	SRC104	TT111	862234	862334	101	576.2

sRNA candidate	Target Transcript	Start	End	Length (nt)	RPKM
SRC104	TT111	862234	862334	101	576.2

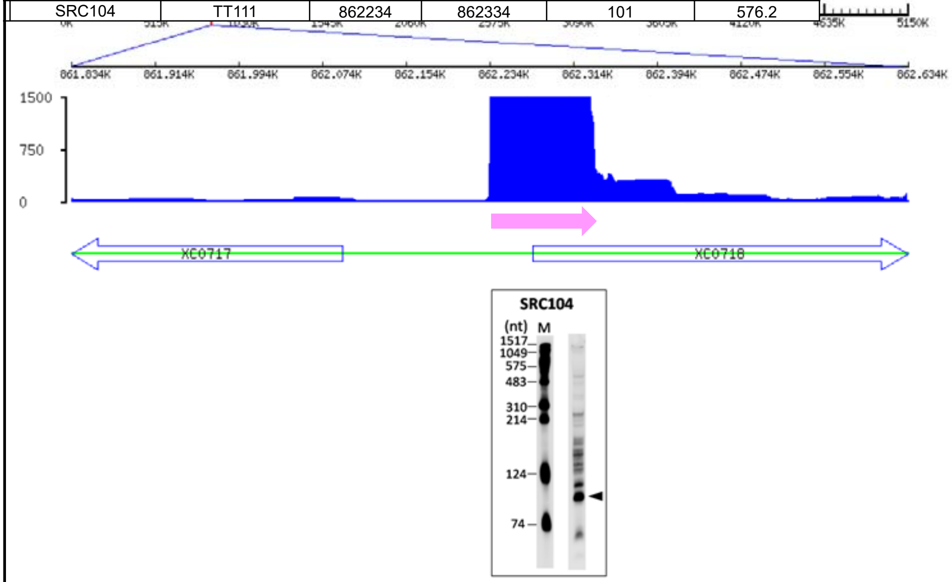

sRNA name	sRNA candidate	Target Transcript	Start	End	Length (nt)	RPKM
sRX038	SRC112	TT121	923871	923936	66	195.5

Now ranger of the following image is from 923471 **bp to** 924270 **bp , Chromsome zoom value is** 1 : 1 **, Depth zoom value is 5 : 1**

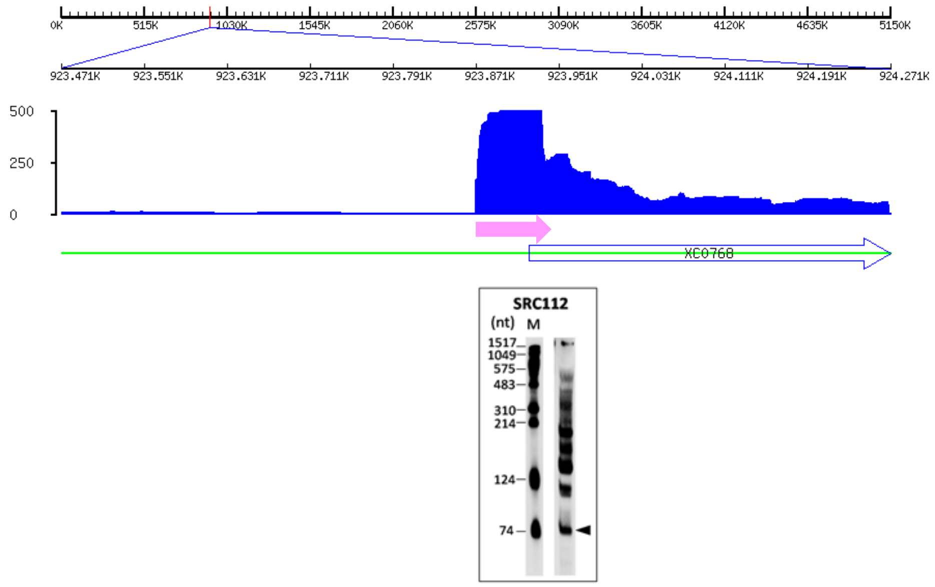

sRNA name	sRNA candidate	Target Transcript	Start	End	Length (nt)	RPKM
	SRC115	TT124	971506	971565	60	58.1

one lineone line**Now ranger of the following image is from** 971106 bp **to** 971905 bp , **Chromsome zoom value is** 1 : 1 , **Depth zoom value is 1 : 1**

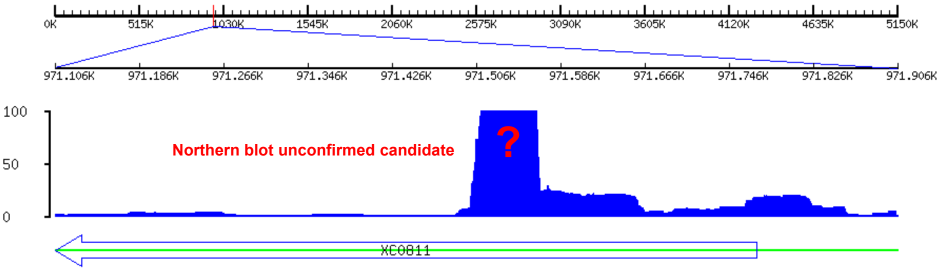

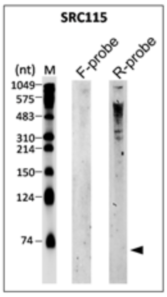

sRNA name	sRNA candidate	Target Transcript	Start	End	Length (nt)	RPKM
sRX039	SRC118	TT127	998027	998098	72	88.6

Now ranger of the following image is from 997386 bp **to** 998185 bp , **Chromsome zoom value is** 1 : 1 , **Depth zoom value is 1.5 : 1**

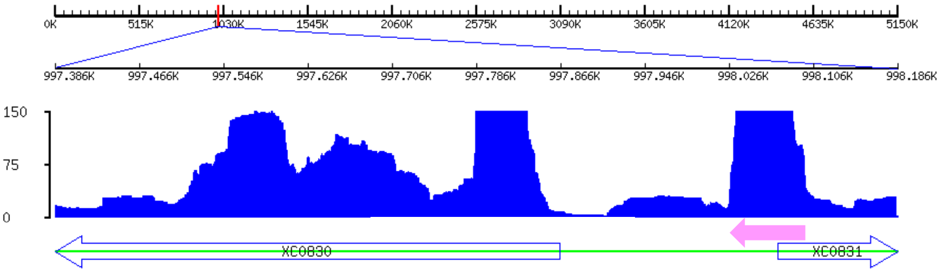

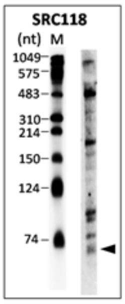

sRNA name	sRNA candidate	Target Transcript	Start	End	Length (nt)	RPKM
sRX040	SRC119	TT128	1009676	1009743	68	162.5

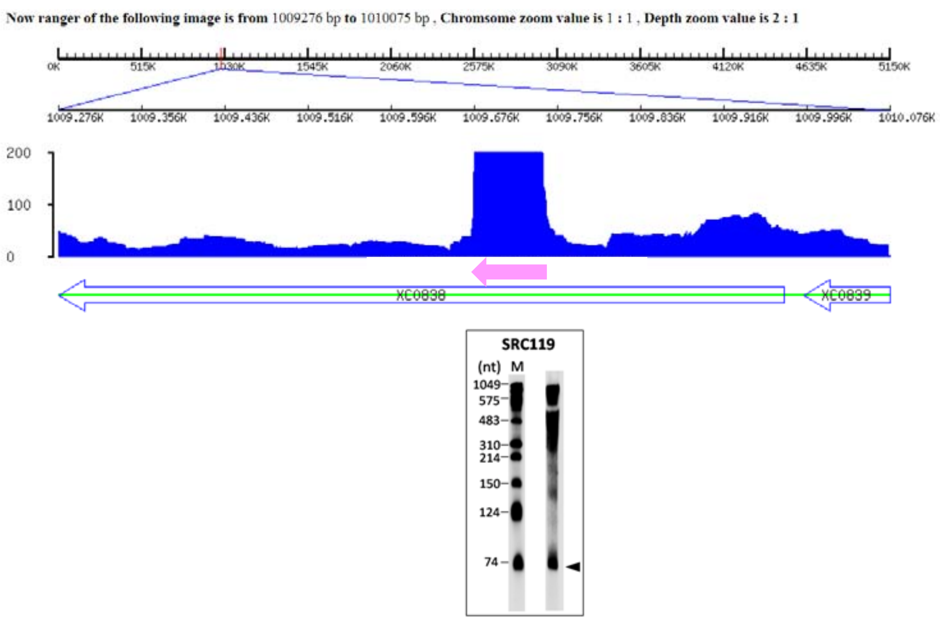

sRNA name	sRNA candidate	Target Transcript	Start	End	Length (nt)	RPKM
sRX041	SRC120	TT129	1013277	1013372	96	2433.3

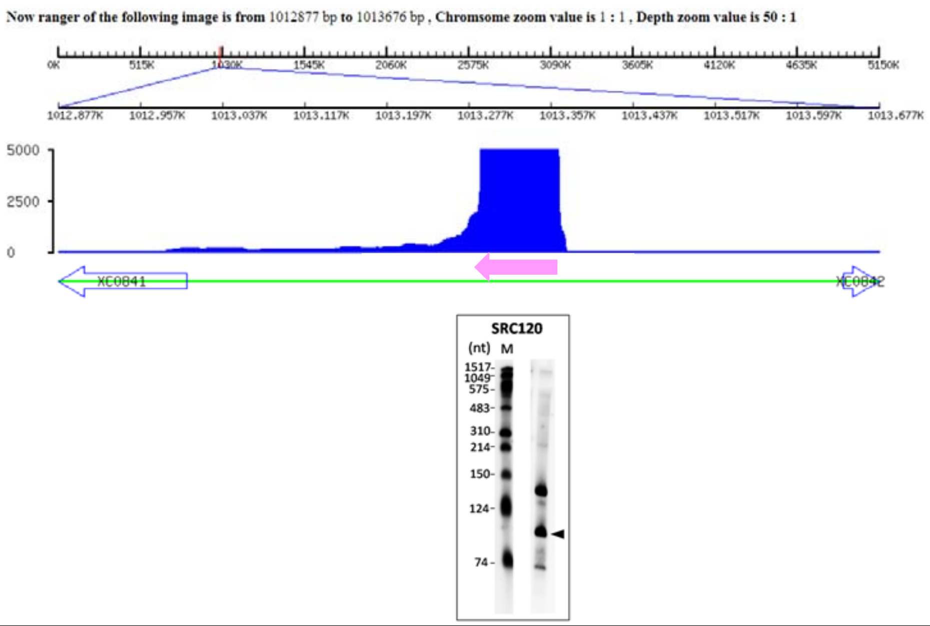

sRNA name	sRNA candidate	Target Transcript	Start	End	Length (nt)	RPKM
sRX042	SRC121	TT130	1014431	1014497	67	53.9

one lineone lineNow ranger of the following image is from 1014031 bp to 1014830 bp , Chromsome zoom value is 1 : 1 , Depth zoom value is 1.5 : 1

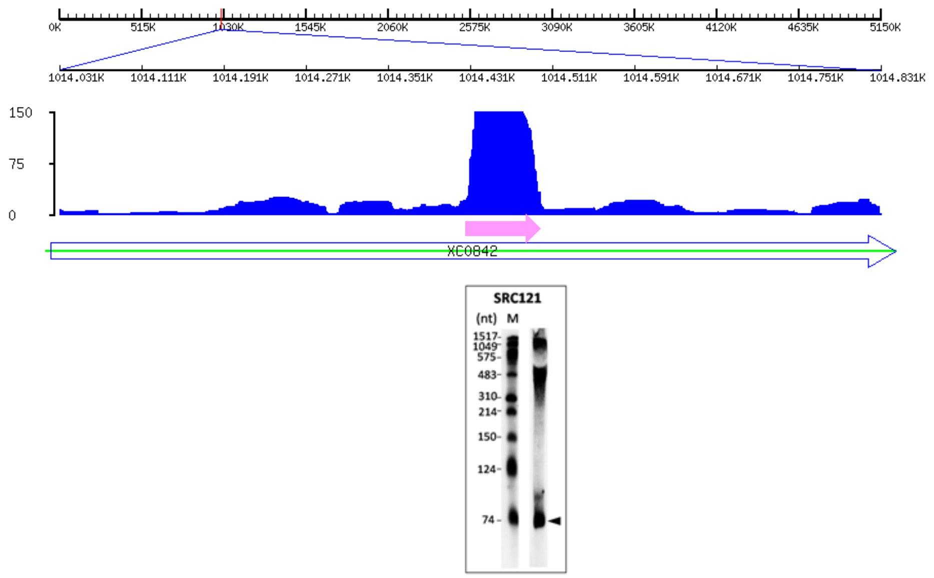

sRNA name	sRNA candidate	Target Transcript	Start	End	Length (nt)	RPKM
sRX043	SRC122	TT131	1019341	1019428	88	1519.9

Now ranger of the following image is from 1018941 bp to 1019740 bp , Chromsome zoom value is 1 : 1 , Depth zoom value is 10 : 1

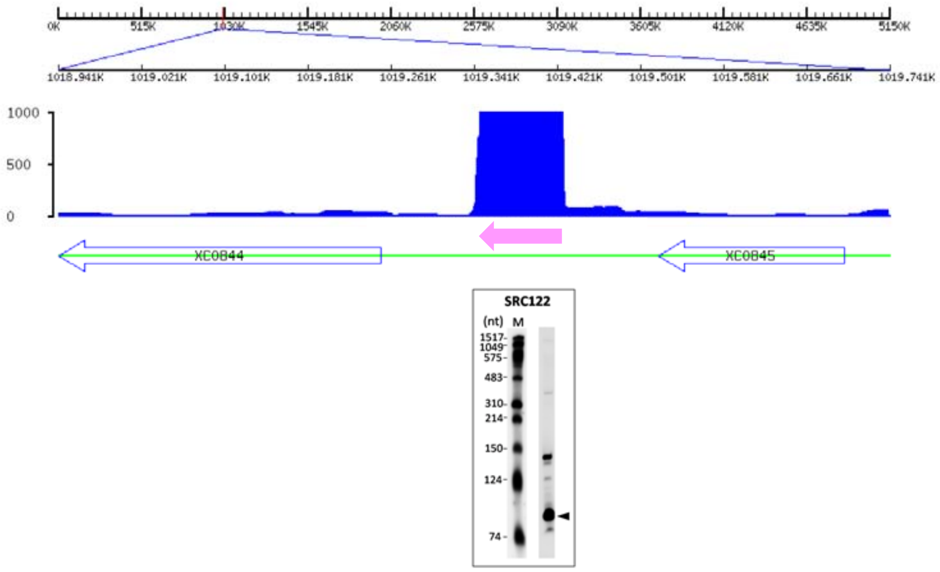

sRNA name	sRNA candidate	Target Transcript	Start	End	Length (nt)	RPKM
sRX044	SRC123	TT132	1019825	1020040	215	178.2

Now ranger of the following image is from 1019425 bp to 1020224 bp , Chromsome zoom value is 1 : 1 , Depth zoom value is 3 : 1

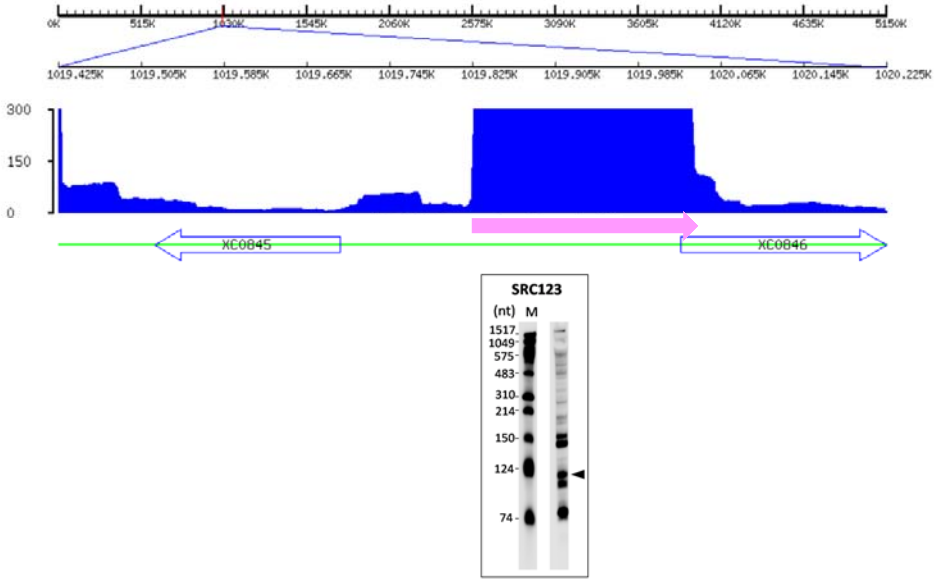

sRNA name	sRNA candidate	Target Transcript	Start	End	Length (nt)	RPKM
sRX045	SRC124	TT133	1022098	1022147	50	360.9

Now ranger of the following image is from 1021698 bp to 1022497 bp , Chromsome zoom value is 1 : 1 , Depth zoom value is 7 : 1

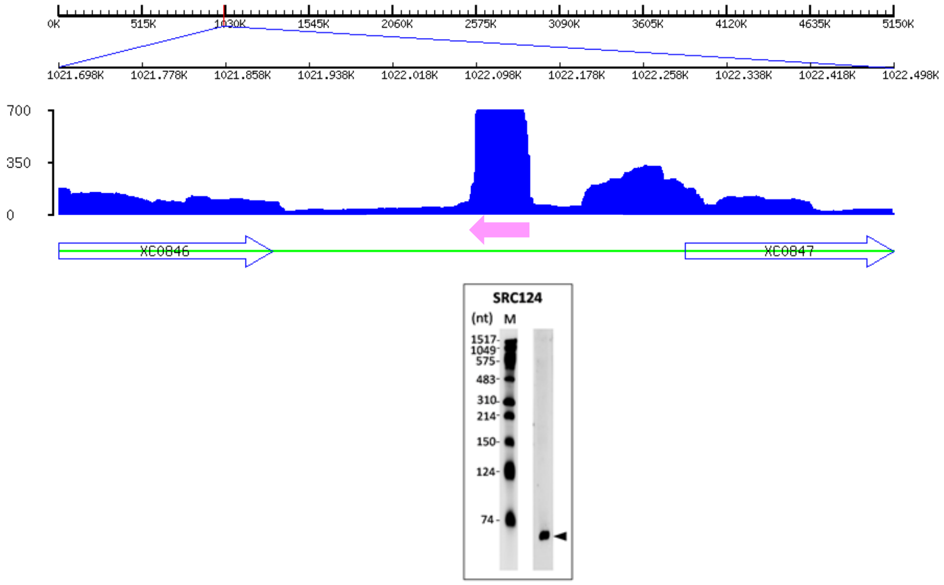

sRNA name	sRNA candidate	Target Transcript	Start	End	Length (nt)	RPKM
sRX046	SRC128	TT137	1035293	1035345	53	137.23

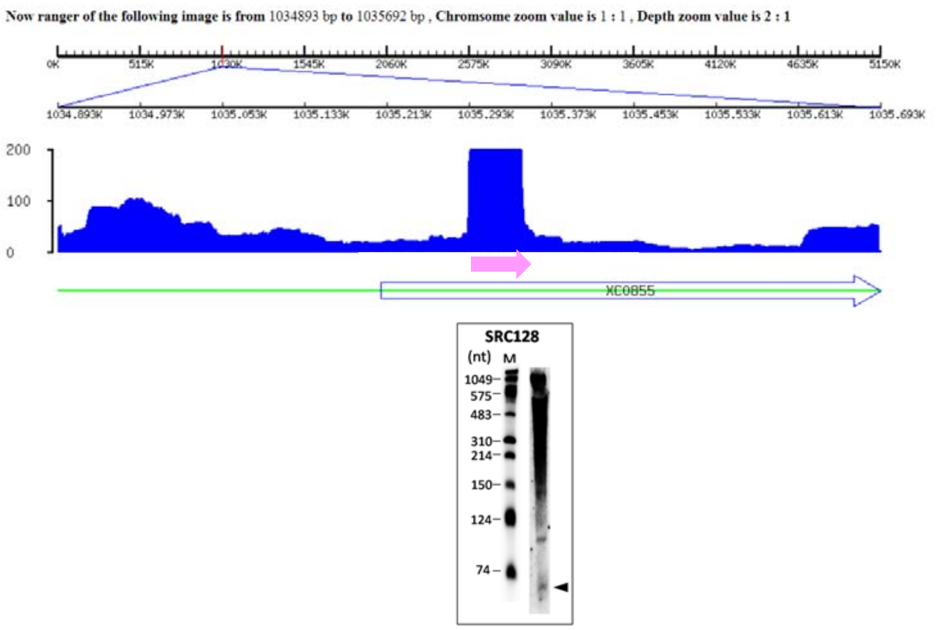

sRNA name	sRNA candidate	Target Transcript	Start	End	Length (nt)	RPKM
sRX047	SRC133	TT142	1052567	1052660	94	149.1

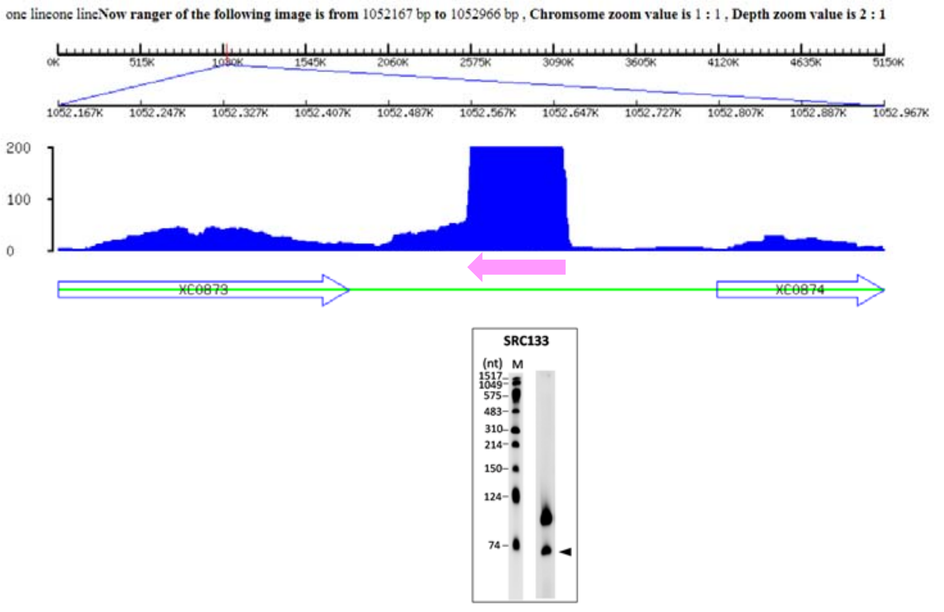

sRNA name	sRNA candidate	Target Transcript	Start	End	Length (nt)	RPKM
sRX048	SRC134	TT143	1074880	1075087	207	25.1

Now ranger of the following image is from 1074480 bp to 1075279 bp , Chromsome zoom value is 1 : 1 , Depth zoom value is 0.8 : 1

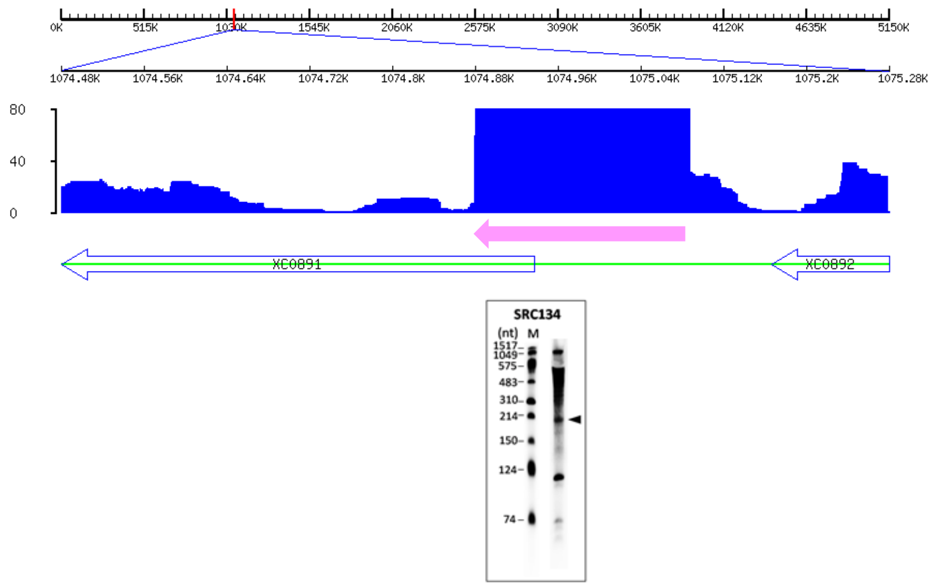

sRNA name	sRNA candidate	Target Transcript	Start	End	Length (nt)	RPKM
sRX049	SRC137	TT147	1097403	1097479	77	438.3

one lineone lineNow ranger of the following image is from 1097003 bp to 1097802 bp , Chromsome zoom value is 1 : 1 , Depth zoom value is 4 : 1

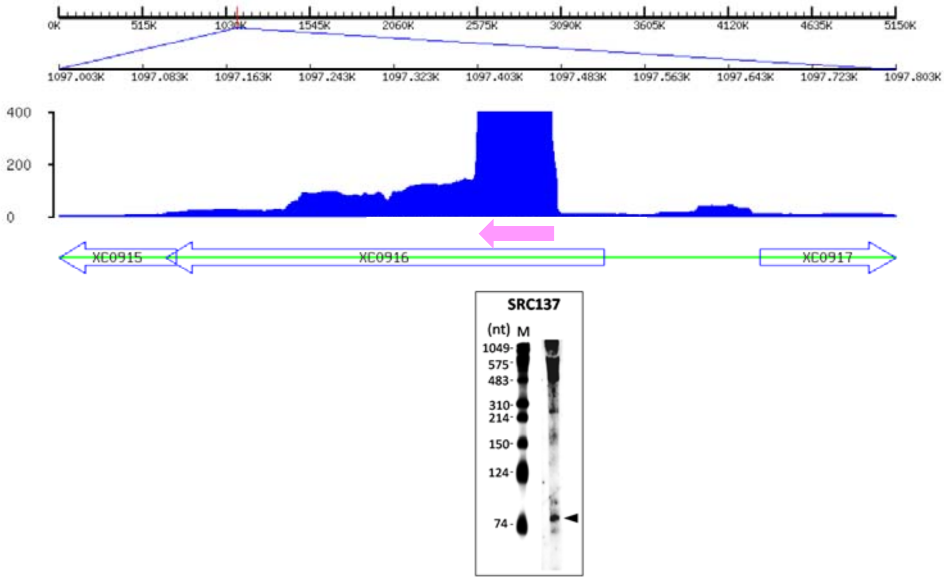

sRNA name	sRNA candidate	Target Transcript	Start	End	Length (nt)	RPKM
sRX050	SRC138	TT148	1108108	1108160	53	93.4

Now ranger of the following image is from 1107708 bp to 1108507 bp , Chromsome zoom value is 1 : 1 , Depth zoom value is 2 : 1

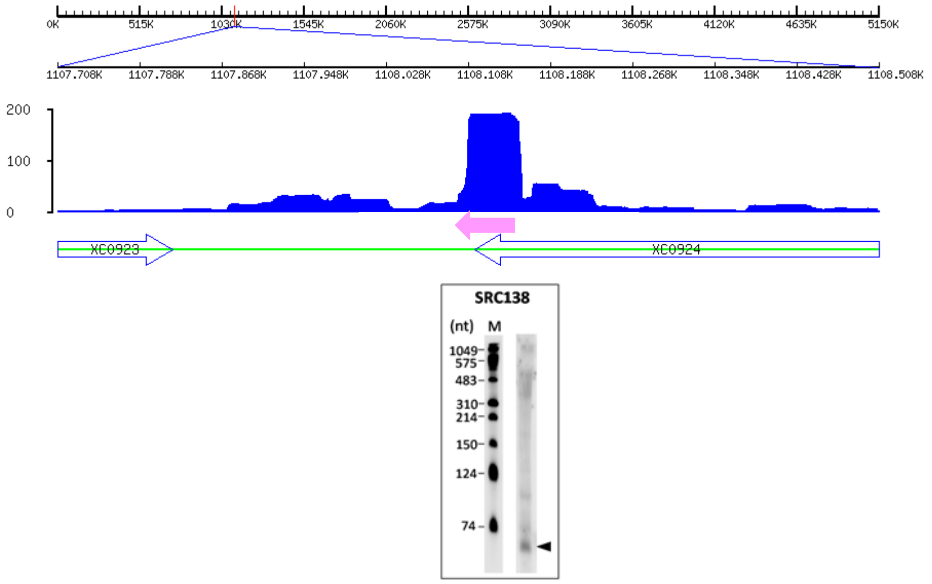

sRNA name	sRNA candidate	Target Transcript	Start	End	Length (nt)	RPKM
sRX051	SRC147	TT159	1231053	1231129	77	491.6

Now ranger of the following image is from 1230474 bp to 1231273 bp , Chromsome zoom value is 1 : 1 , Depth zoom value is 10 : 1

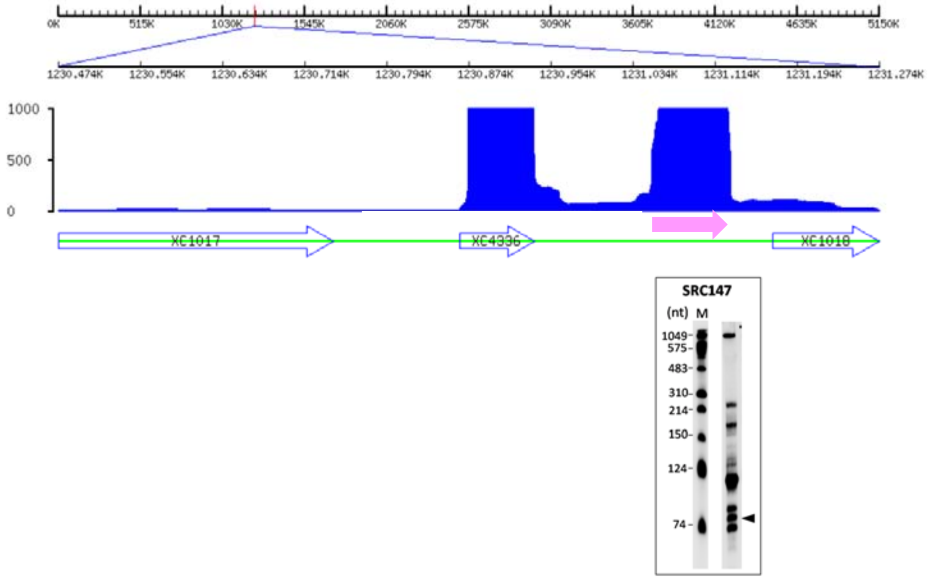

sRNA name	sRNA candidate	Target Transcript	Start	End	Length (nt)	RPKM
sRX052	SRC153	TT165	1269031	1269101	71	1206.7

Now ranger of the following image is from 1268631 bp **to** 1269430 bp , **Chromsome zoom value is** 1 : 1 , **Depth zoom value is 40 : 1**

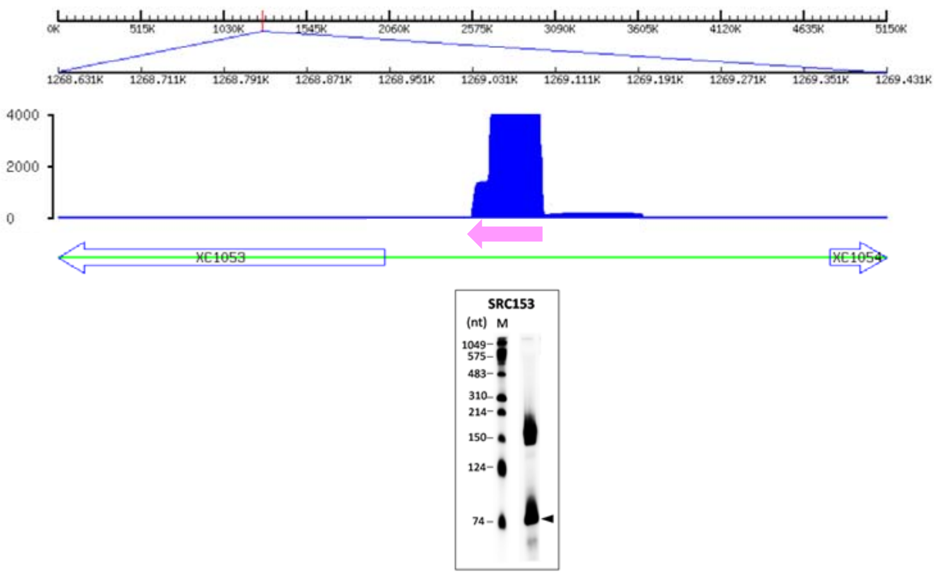

sRNA name	sRNA candidate	Target Transcript	Start	End	Length (nt)	RPKM
sRX053	SRC154	TT166	1295653	1295721	69	70.4

Now ranger of the following image is from 1295253 bp **to** 1296052 bp , **Chromsome zoom value is** 1 : 1 , **Depth zoom value is 1.5 : 1**

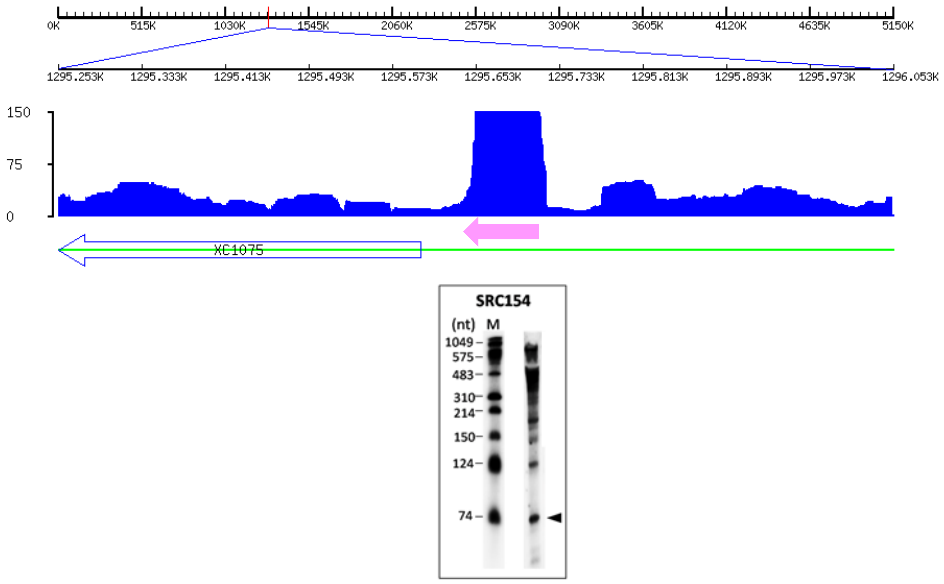

sRNA name	sRNA candidate	Target Transcript	Start	End	Length (nt)	RPKM
	SRC155	TT167	1296858	1296927	70	100.9

Now ranger of the following image is from 1296458 bp **to** 1297257 bp , **Chromsome zoom value is** 1 : 1 , **Depth zoom value is 2 : 1**

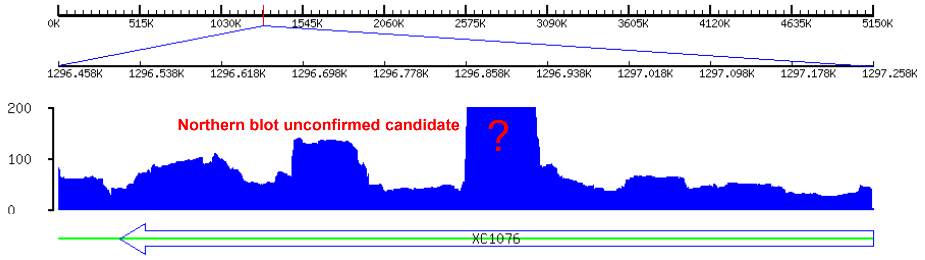

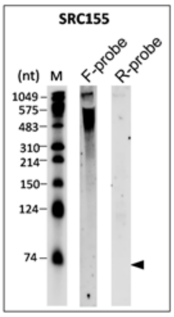

sRNA name	sRNA candidate	Target Transcript	Start	End	Length (nt)	RPKM
sRX054	SRC156	TT168	1316918	1317118	201	133.9

Now ranger of the following image is from 1316838 bp **to** 1317637 bp , **Chromsome zoom value is** 1 : 1 , **Depth zoom value is** 1.7 : 1

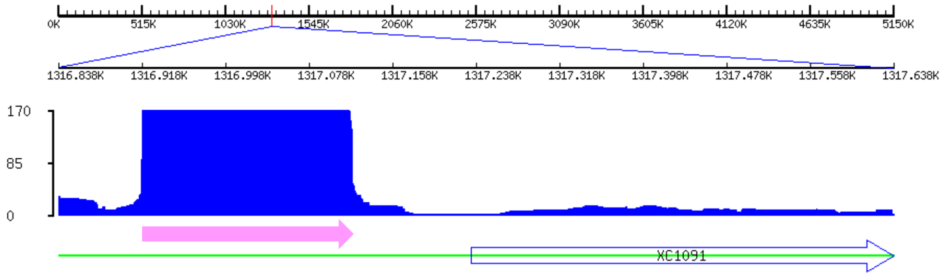

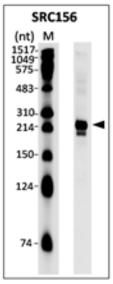

sRNA name	sRNA candidate	Target Transcript	Start	End	Length (nt)	RPKM
sRX055	SRC160	TT172	1381928	1382023	96	228.4

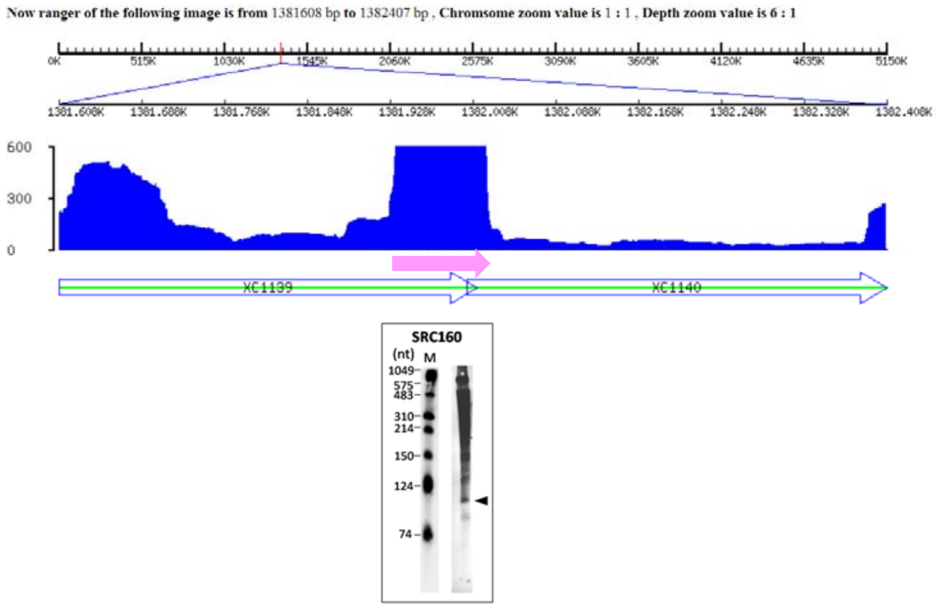

sRNA name	sRNA candidate	Target Transcript	Start	End	Length (nt)	RPKM
sRX056	SRC163	TT176	1412286	1412380	95	194.1

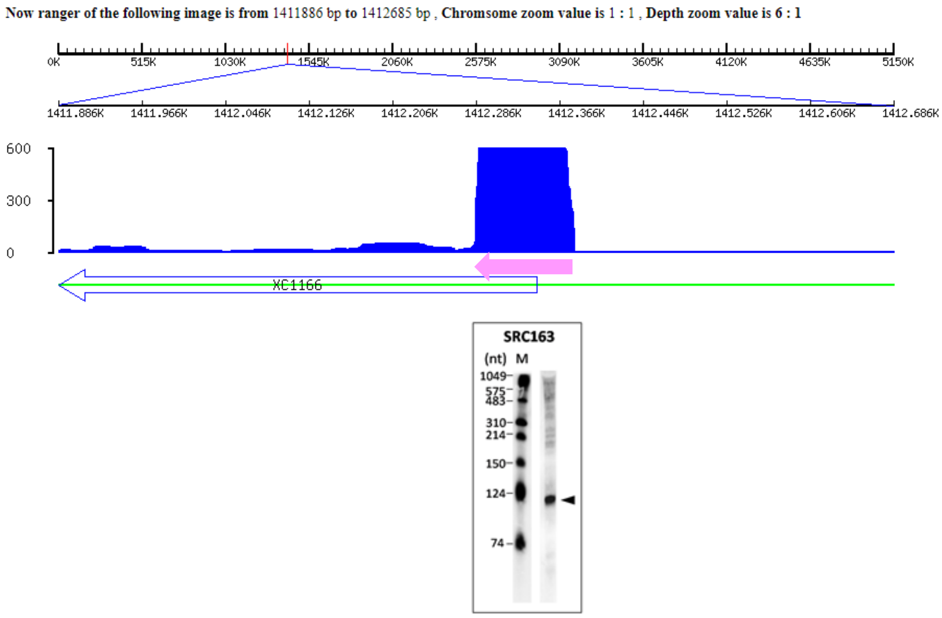

sRNA name	sRNA candidate	Target Transcript	Start	End	Length (nt)	RPKM
sRX057	SRC170	TT183	1511287	1511378	92	150.7

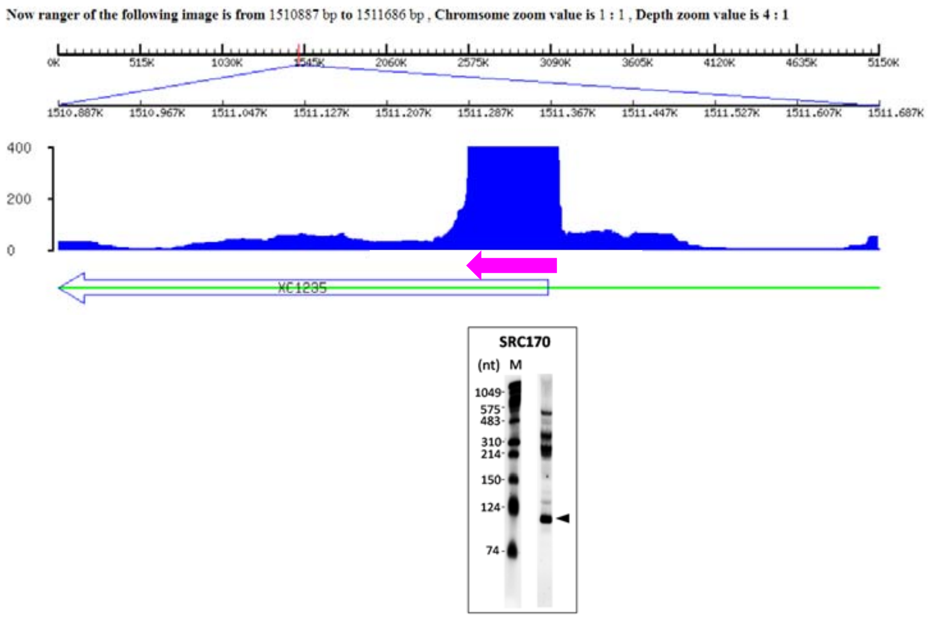

sRNA name	sRNA candidate	Target Transcript	Start	End	Length (nt)	RPKM
sRX058	SRC171	TT184	1531053	1531262	210	456.5

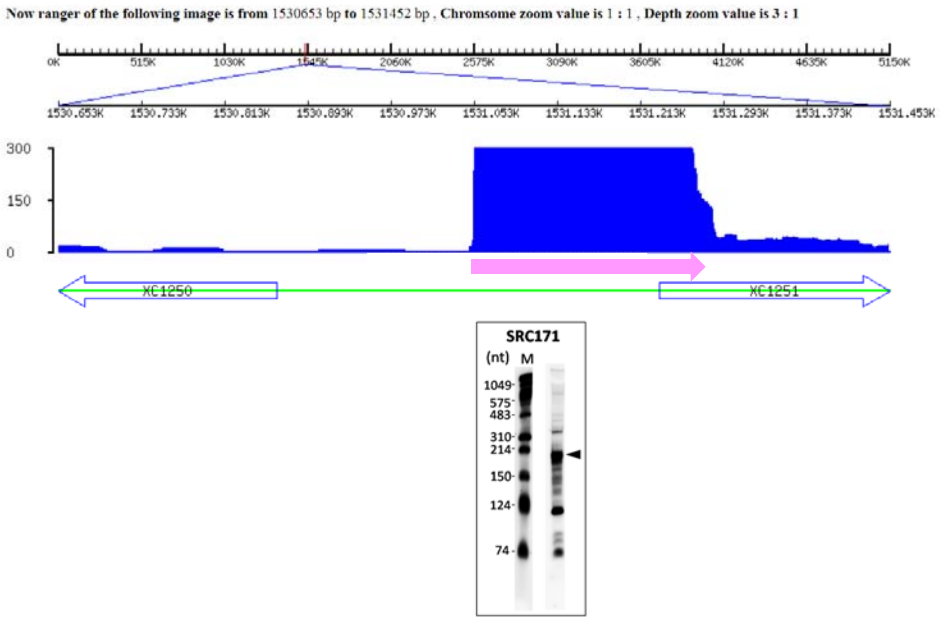

sRNA name	sRNA candidate	Target Transcript	Start	End	Length (nt)	RPKM
sRX059	SRC175	TT189	1594827	1594894	68	53.7

Now ranger of the following image is from 1594427 bp **to** 1595226 bp , **Chromsome zoom value is** 1 : 1 , **Depth zoom value is 1.5 : 1**

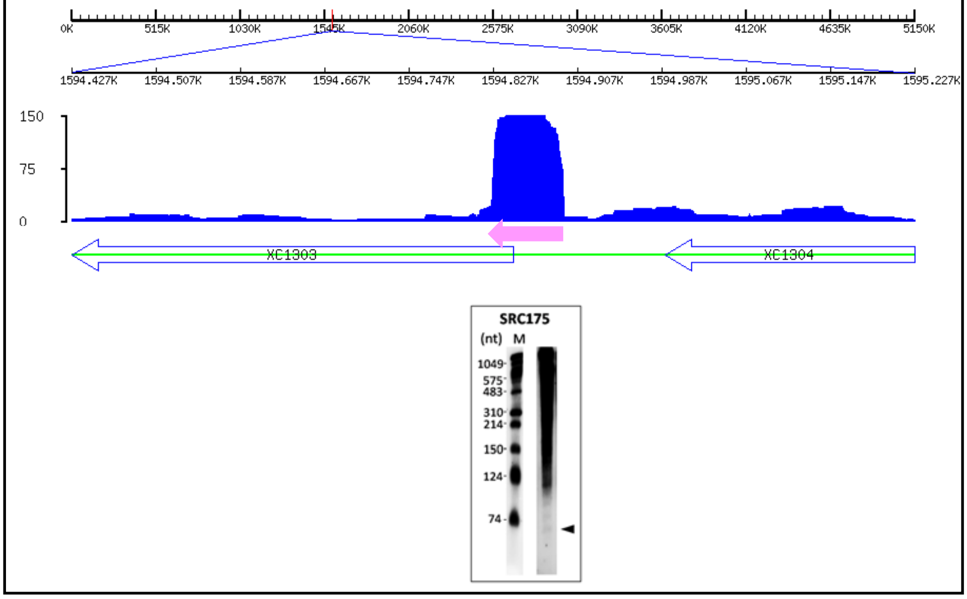

sRNA name	sRNA candidate	Target Transcript	Start	End	Length (nt)	RPKM
sRX060	SRC177	TT191	1600577	1600670	94	300.9

one lineone line**Now ranger of the following image is from** 1600177 bp **to** 1600976 bp , **Chromsome zoom value is** 1 : 1 , **Depth zoom value is 8 : 1**

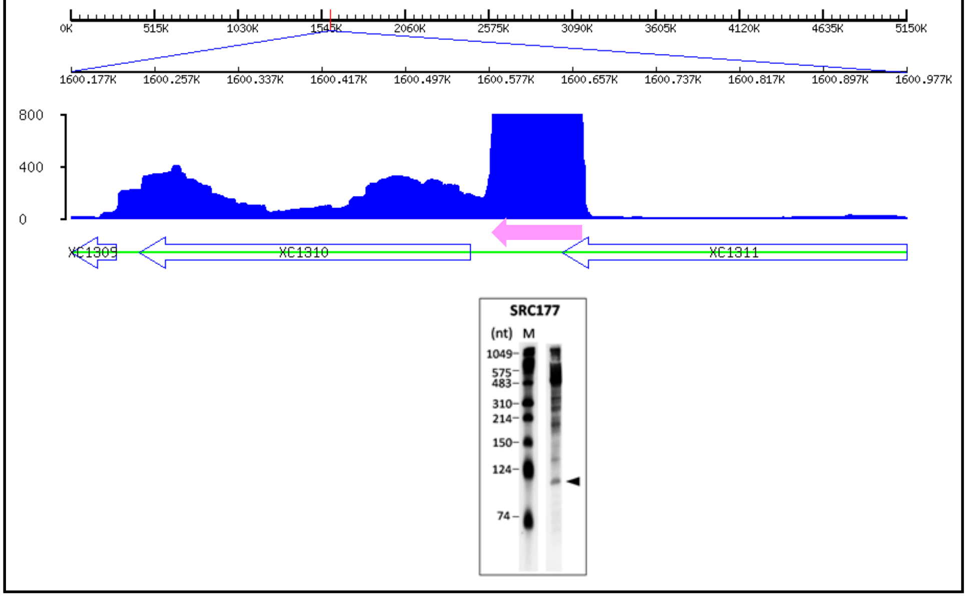

sRNA name	sRNA candidate	Target Transcript	Start	End	Length (nt)	RPKM
sRX061	SRC179	TT194	1618954	1619030	77	5188.6

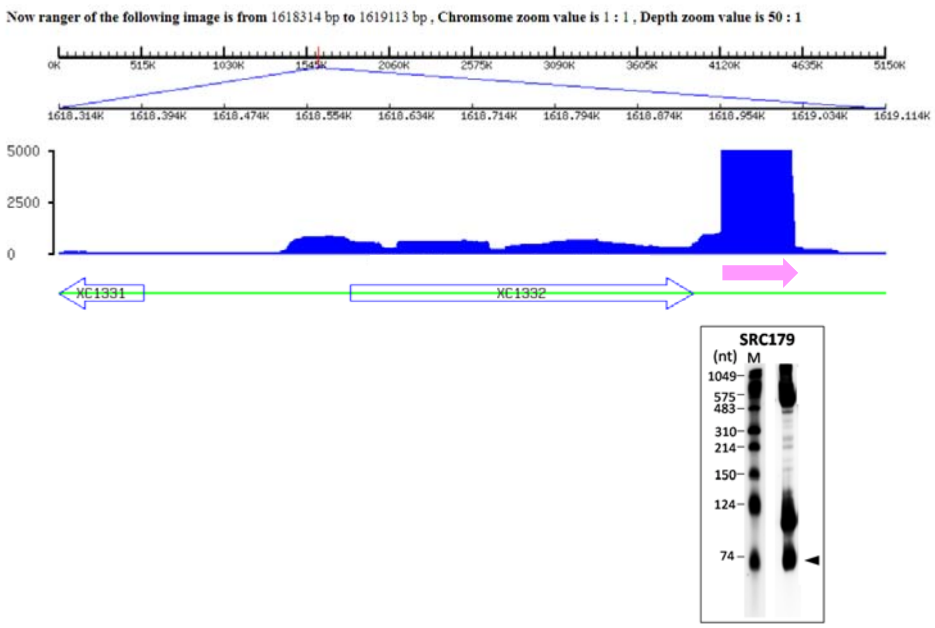

sRNA name	sRNA candidate	Target Transcript	Start	End	Length (nt)	RPKM
sRX062	SRC183	TT198	1634567	1634660	94	193.9

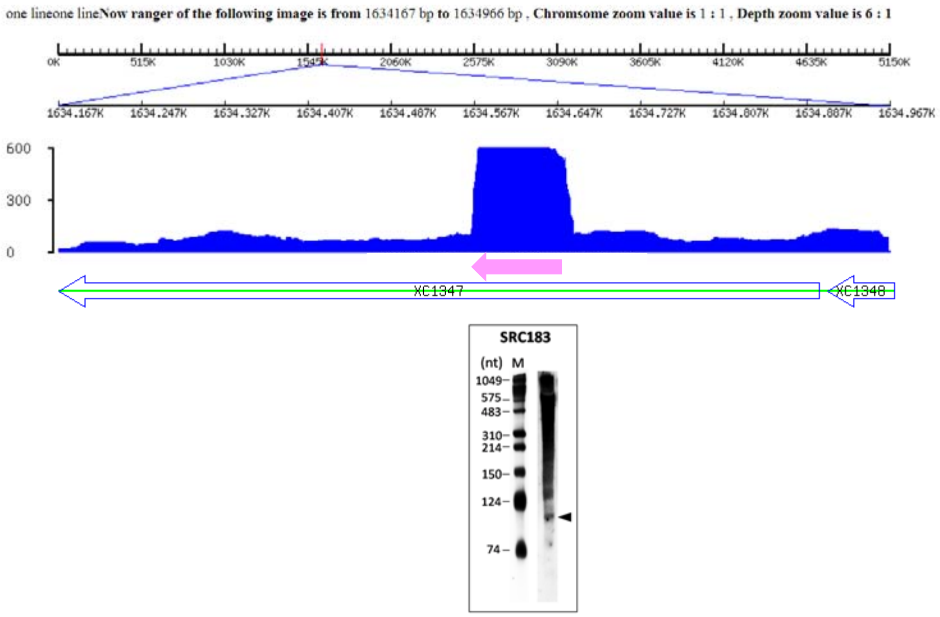

sRNA name	sRNA candidate	Target Transcript	Start	End	Length (nt)	RPKM
sRX063	SRC184	TT199	1638354	1638439	86	37.8

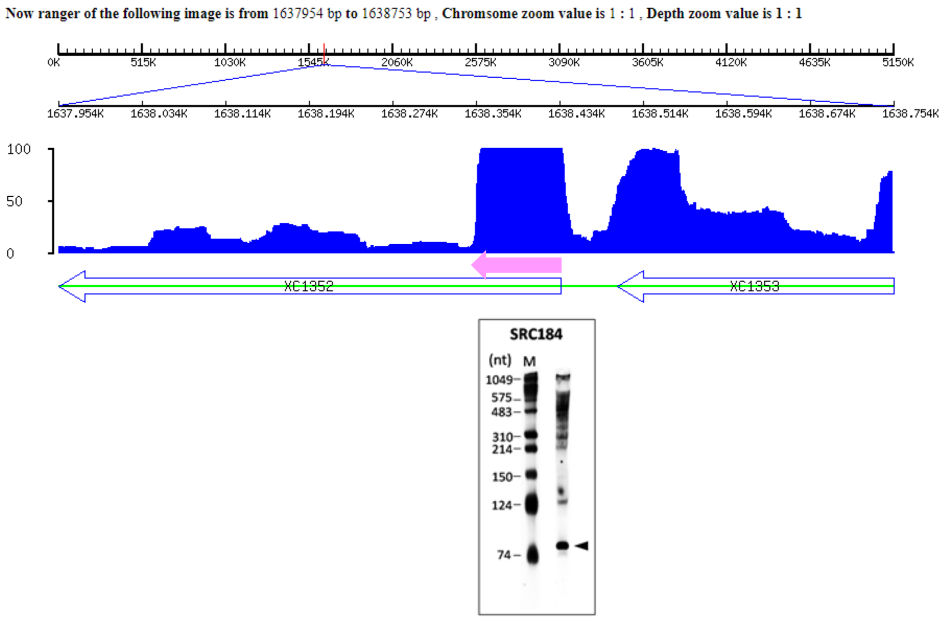

sRNA name	sRNA candidate	Target Transcript	Start	End	Length (nt)	RPKM
sRX064	SRC190	TT205	1671801	1671860	60	154.9

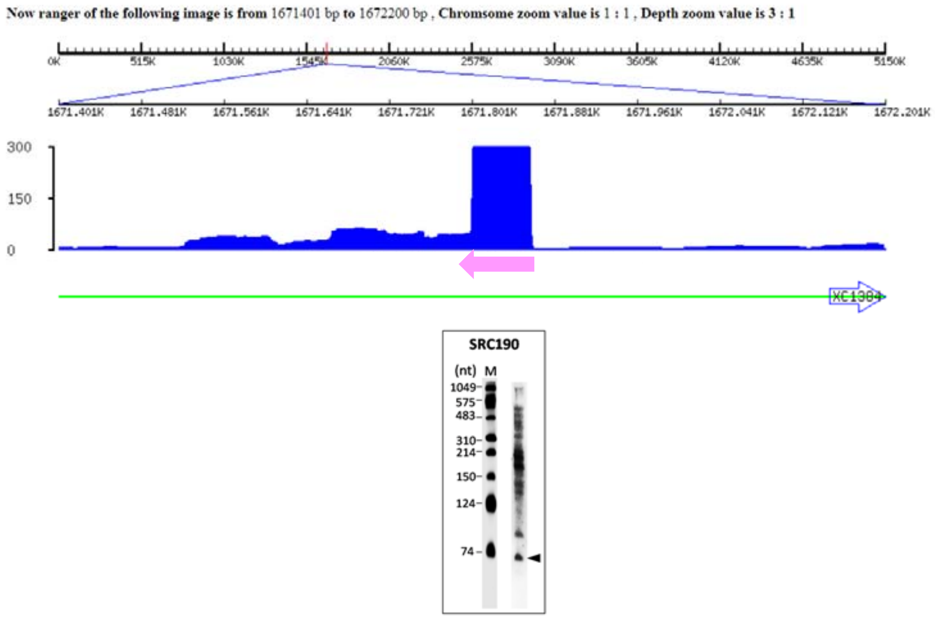

sRNA name	sRNA candidate	Target Transcript	Start	End	Length (nt)	RPKM
sRX065	SRC192	TT207	1690686	1690779	94	56.2

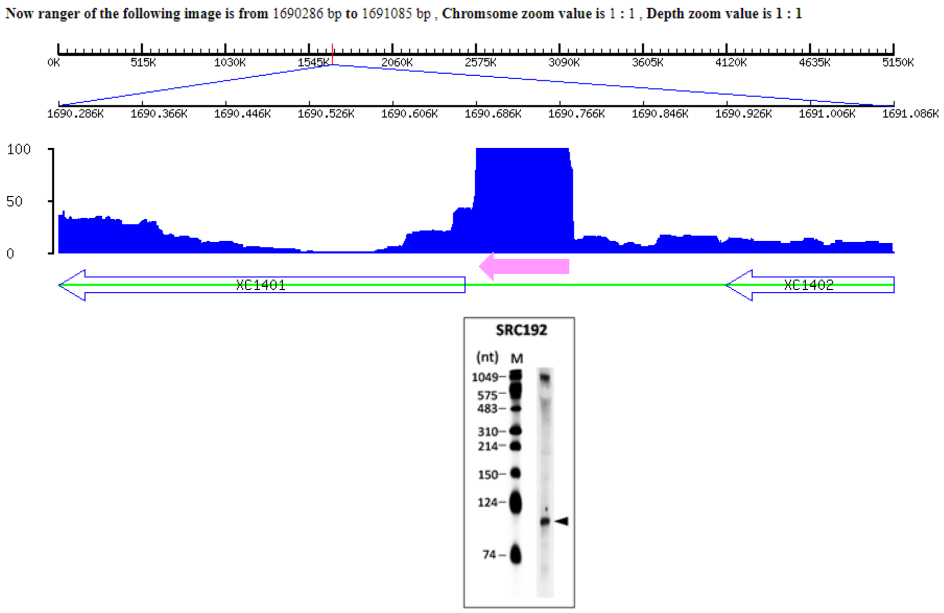

sRNA name	sRNA candidate	Target Transcript	Start	End	Length (nt)	RPKM
sRX066	SRC195	TT210	1747942	1748009	68	157.8

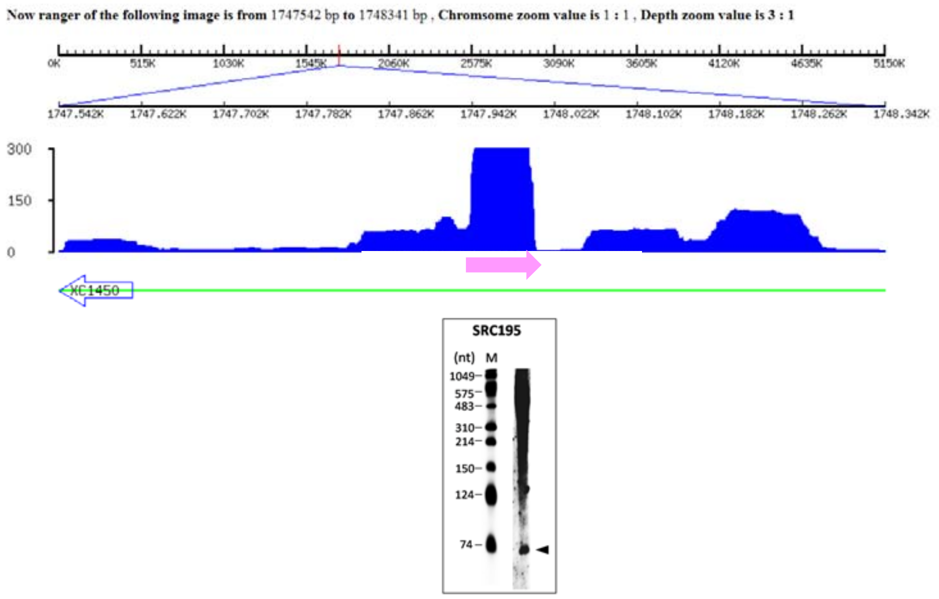

sRNA name	sRNA candidate	Target Transcript	Start	End	Length (nt)	RPKM
sRX067	SRC196	TT211	1750038	1750126	89	214.1

Now ranger of the following image is from 1749638 bp to 1750437 bp , Chromsome zoom value is 1 : 1 , Depth zoom value is 1.5 : 1

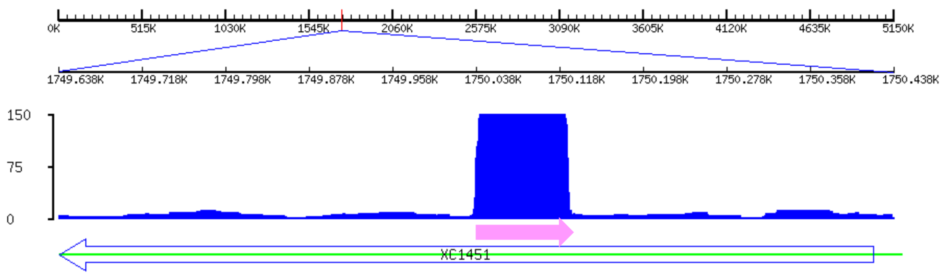

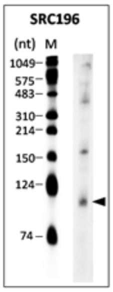

sRNA name	sRNA candidate	Target Transcript	Start	End	Length (nt)	RPKM
sRX068	SRC197	TT212	1751067	1751161	95	110.3

Now ranger of the following image is from 1750667 bp to 1751466 bp , Chromsome zoom value is 1 : 1 , Depth zoom value is 2 : 1

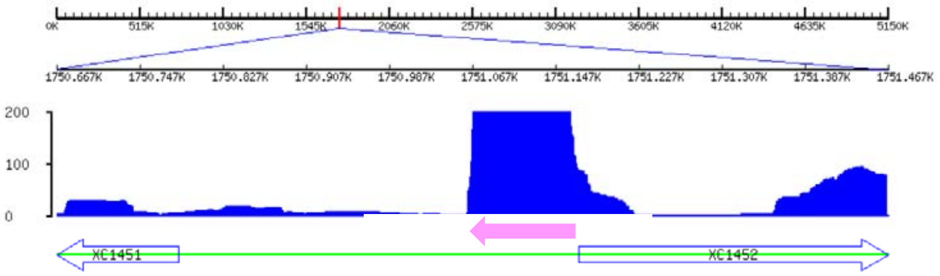

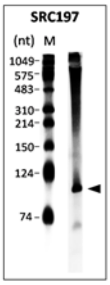

sRNA name	sRNA candidate	Target Transcript	Start	End	Length (nt)	RPKM
sRX069	SRC198	TT213	1791899	1791966	68	72.1

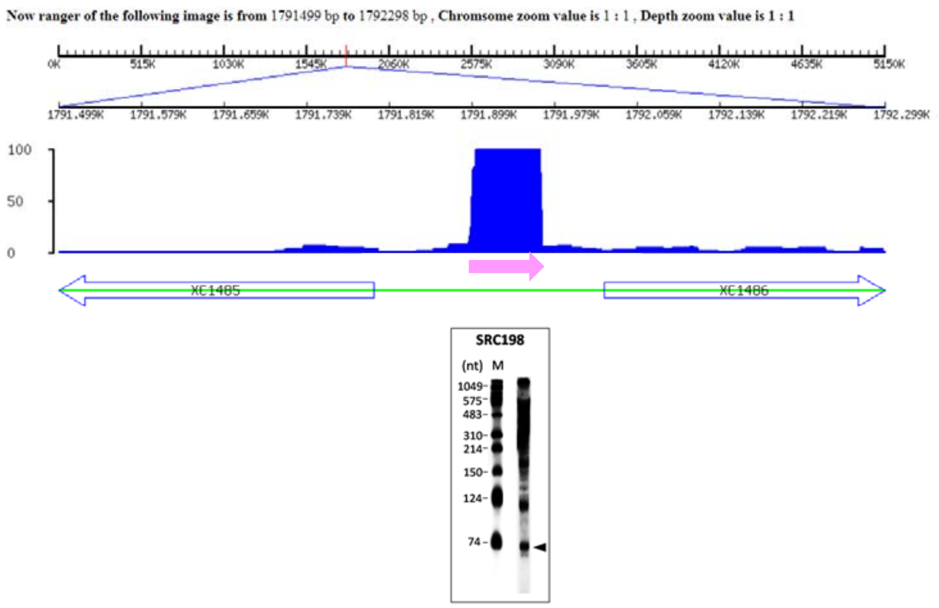

sRNA name	sRNA candidate	Target Transcript	Start	End	Length (nt)	RPKM
sRX070	SRC203	TT219	1807102	1807157	56	249.3

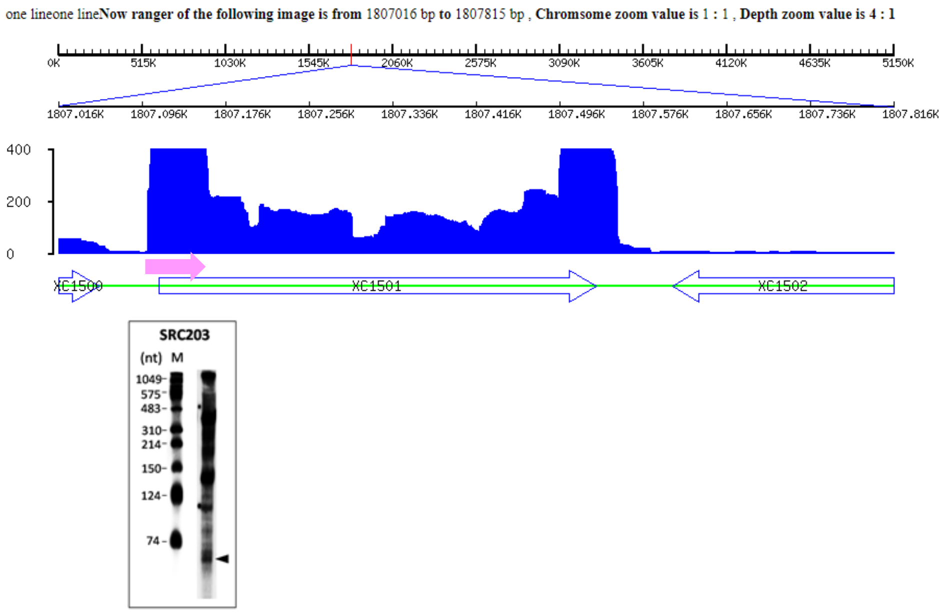

sRNA name	sRNA candidate	Target Transcript	Start	End	Length (nt)	RPKM
sRX071	SRC205	TT221	1814332	1814398	67	105.9

Now ranger of the following image is from 1813932 **bp to** 1814731 bp , **Chromsome zoom value is** 1 : 1 , **Depth zoom value is 2 : 1**

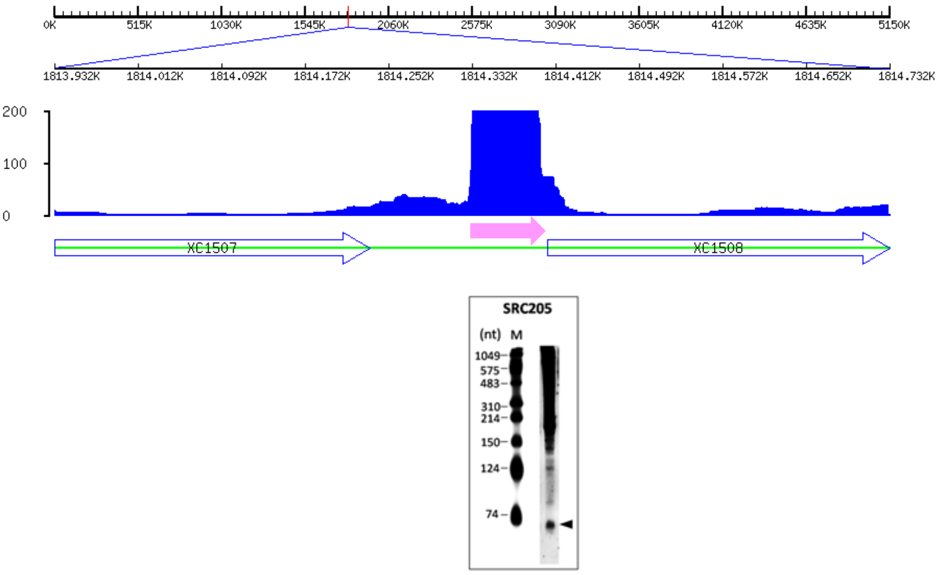

sRNA name	sRNA candidate	Target Transcript	Start	End	Length (nt)	RPKM
sRX072	SRC207	TT223	1843442	1843503	62	376.9

one lineone line**Now ranger of the following image is from** 1843042 **bp to** 1843841 bp , **Chromsome zoom value is** 1 : 1 , **Depth zoom value is 6 : 1**

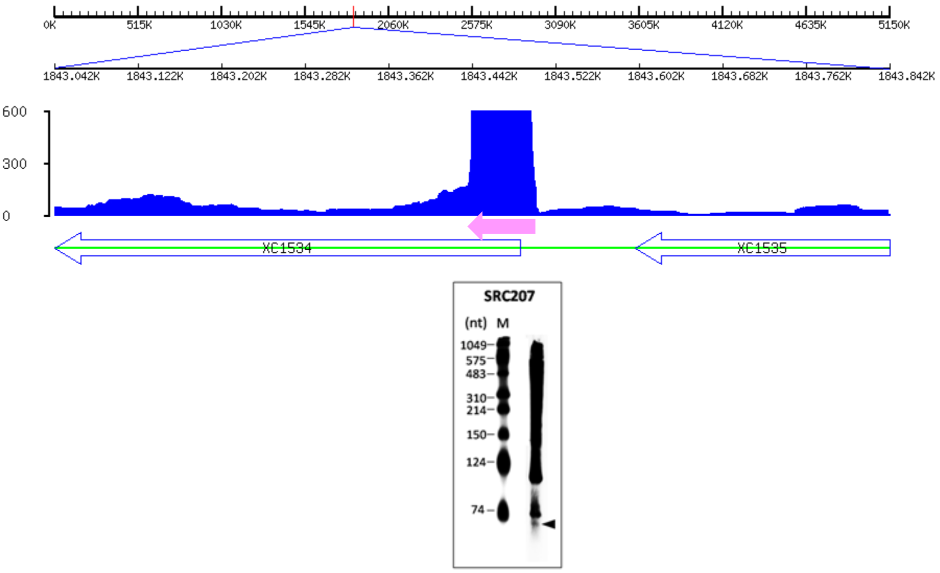

sRNA name	sRNA candidate	Target Transcript	Start	End	Length (nt)	RPKM
sRX073	SRC208	TT224	1844170	1844248	79	54.9

Now ranger of the following image is from 1843770 bp **to** 1844569 bp , **Chromsome zoom value is** 1 : 1 , **Depth zoom value is 4 : 1**

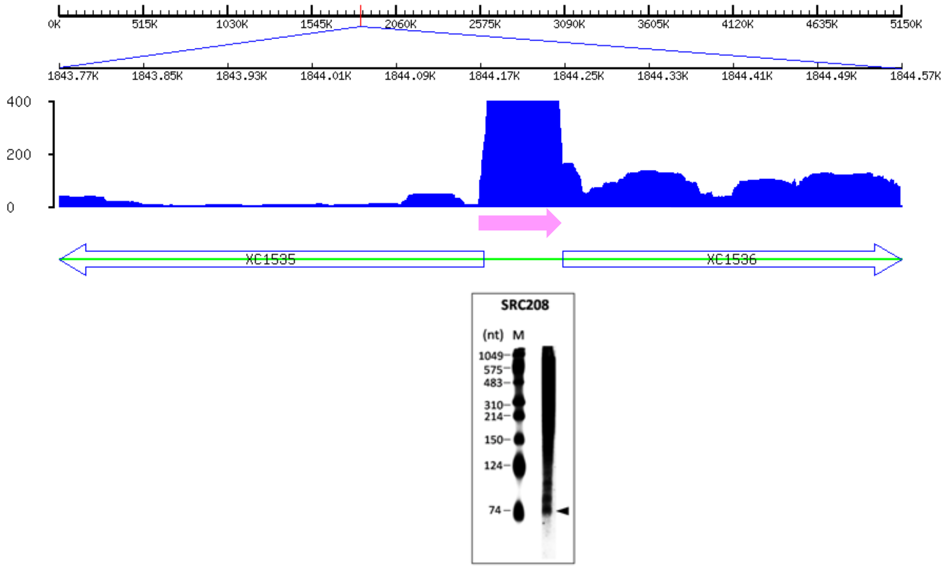

sRNA name	sRNA candidate	Target Transcript	Start	End	Length (nt)	RPKM
sRX074	SRC213	TT229	1896637	1896730	94	151.1

Now ranger of the following image is from 1896237 bp **to** 1897036 bp , **Chromsome zoom value is** 1 : 1 , **Depth zoom value is 5 : 1**

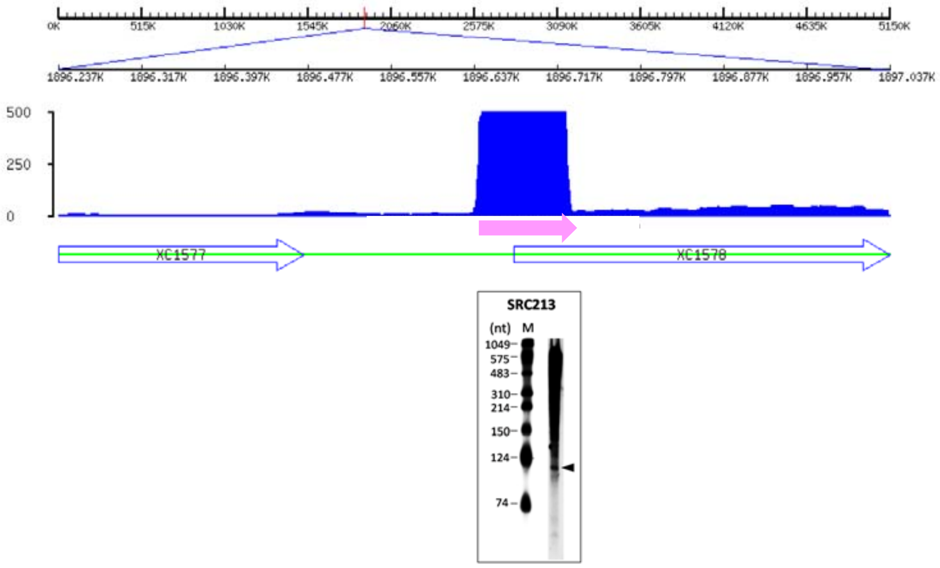

sRNA name	sRNA candidate	Target Transcript	Start	End	Length (nt)	RPKM
sRX075	SRC218	TT238	1955793	1955849	57	297.2

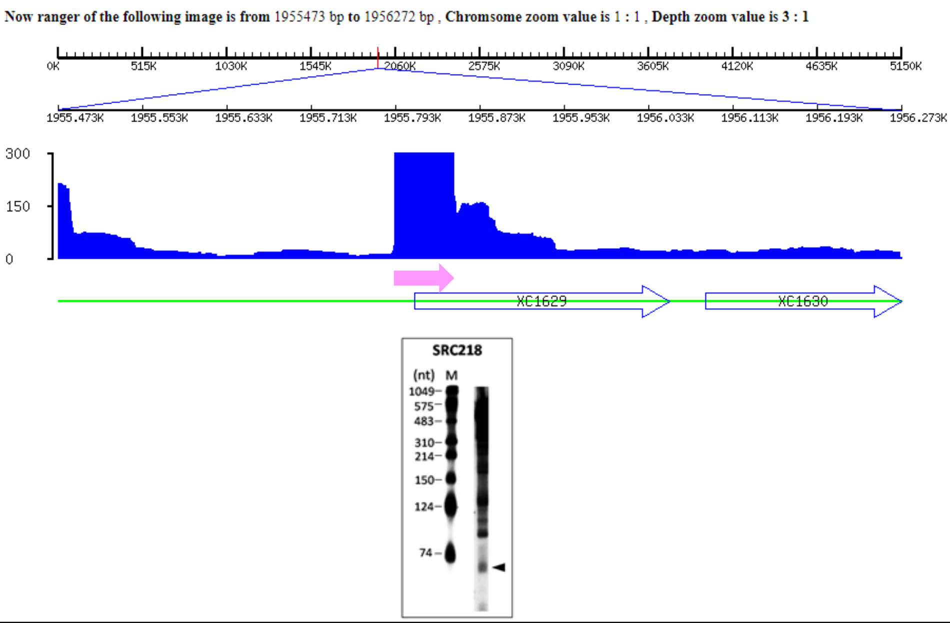

sRNA name	sRNA candidate	Target Transcript	Start	End	Length (nt)	RPKM
sRX076	SRC220	TT240	1964190	1964283	94	226.1

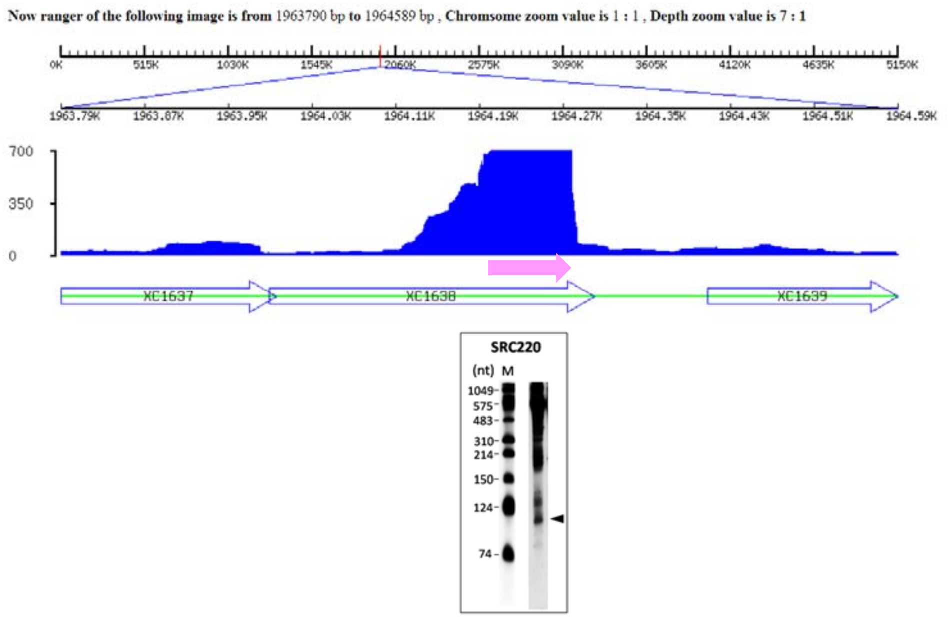

sRNA name	sRNA candidate	Target Transcript	Start	End	Length (nt)	RPKM
sRX077	SRC222	TT242	1987898	1988088	191	419.5

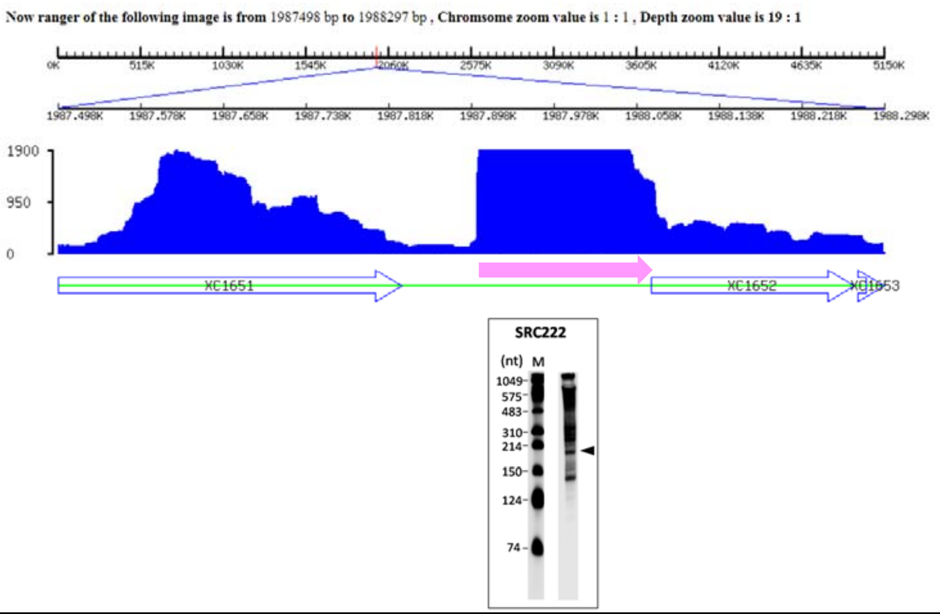

sRNA name	sRNA candidate	Target Transcript	Start	End	Length (nt)	RPKM
sRX078	SRC229	TT250	2018414	2018507	94	634.9

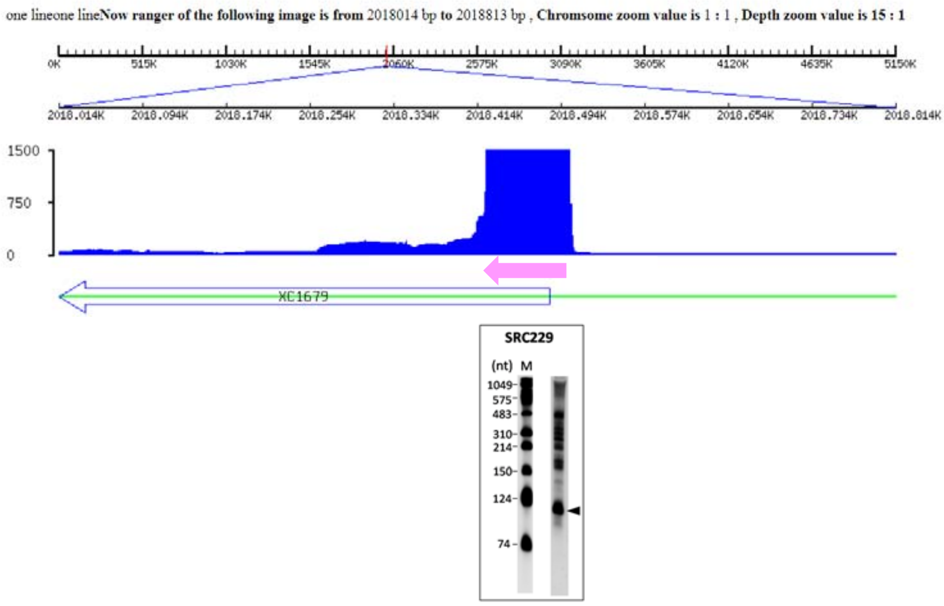

sRNA name	sRNA candidate	Target Transcript	Start	End	Length (nt)	RPKM
sRX079	SRC231	TT254	2117629	2117706	78	53.3

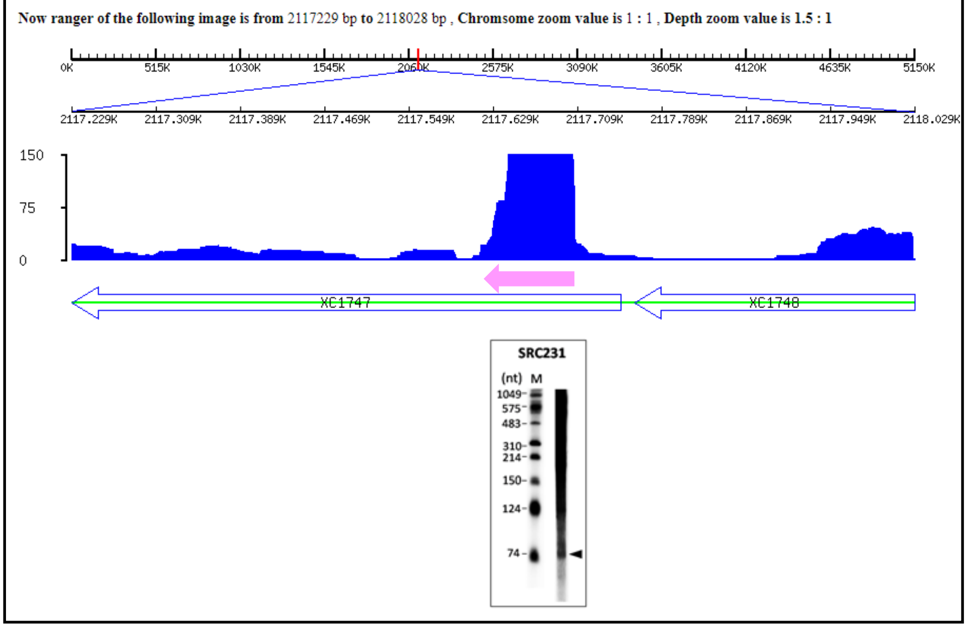

sRNA name	sRNA candidate	Target Transcript	Start	End	Length (nt)	RPKM
sRX080	SRC235	TT258	2170287	2170349	63	478.5

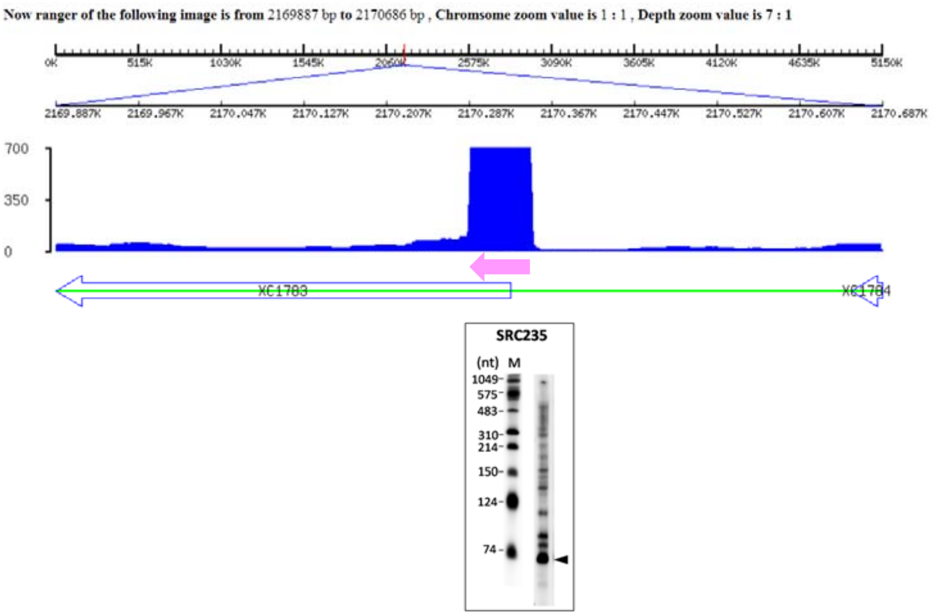

sRNA name	sRNA candidate	Target Transcript	Start	End	Length (nt)	RPKM
sRX081	SRC239	TT262	2221405	2221619	214	2615.9

one lineone lineone lineNow ranger of the following image is from 2221005 bp to 2221804 bp , Chromsome zoom value is 1 : 1 , Depth zoom value is 15 : 1

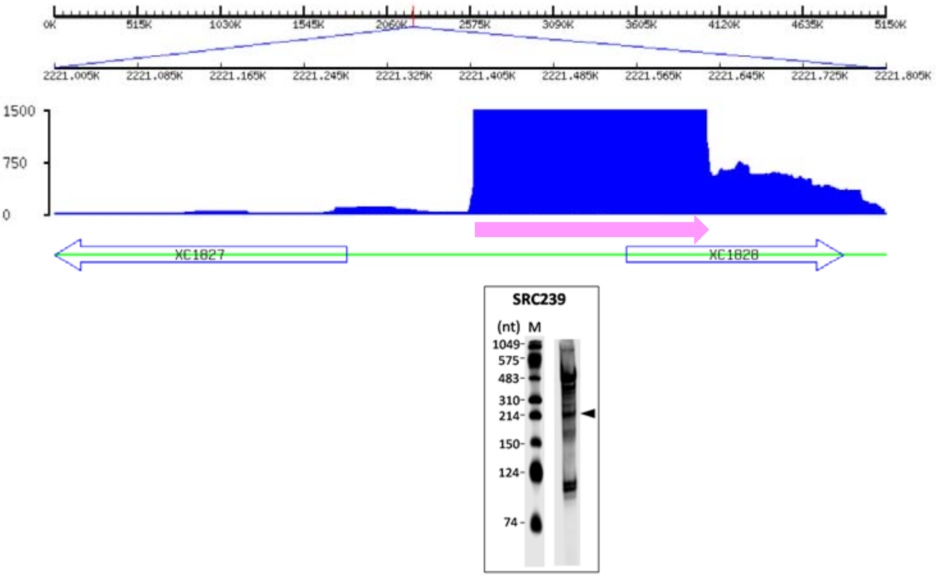

sRNA name	sRNA candidate	Target Transcript	Start	End	Length (nt)	RPKM
sRX082	SRC240	TT263	2223778	2223838	61	58.9

Now ranger of the following image is from 2223378 bp to 2224177 bp , Chromsome zoom value is 1 : 1 , Depth zoom value is 1 : 1

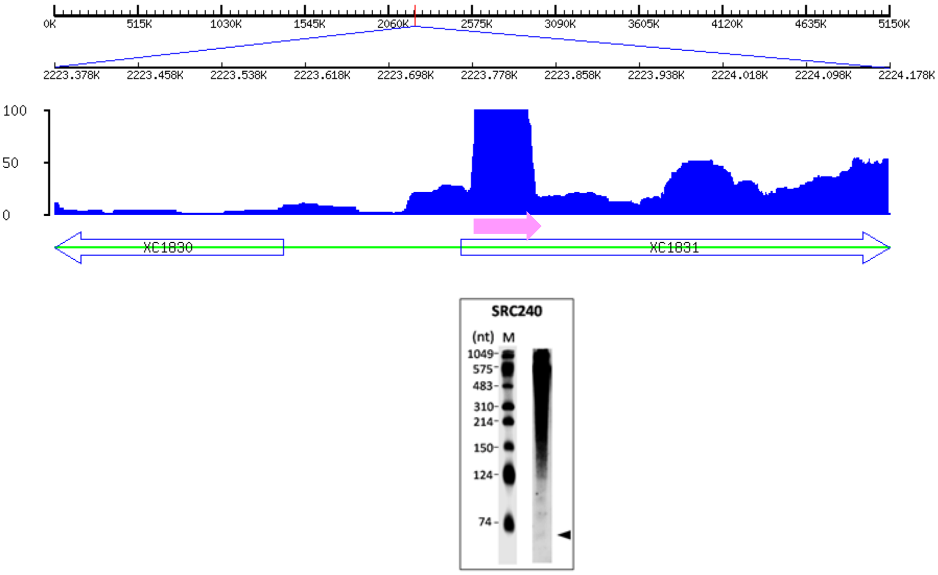

sRNA name	sRNA candidate	Target Transcript	Start	End	Length (nt)	RPKM
sRX083	SRC245	TT270	2281622	2281711	90	82.2

Now ranger of the following image is from 2281222 bp to 2282021 bp , Chromsome zoom value is 1 : 1 , Depth zoom value is 2 : 1

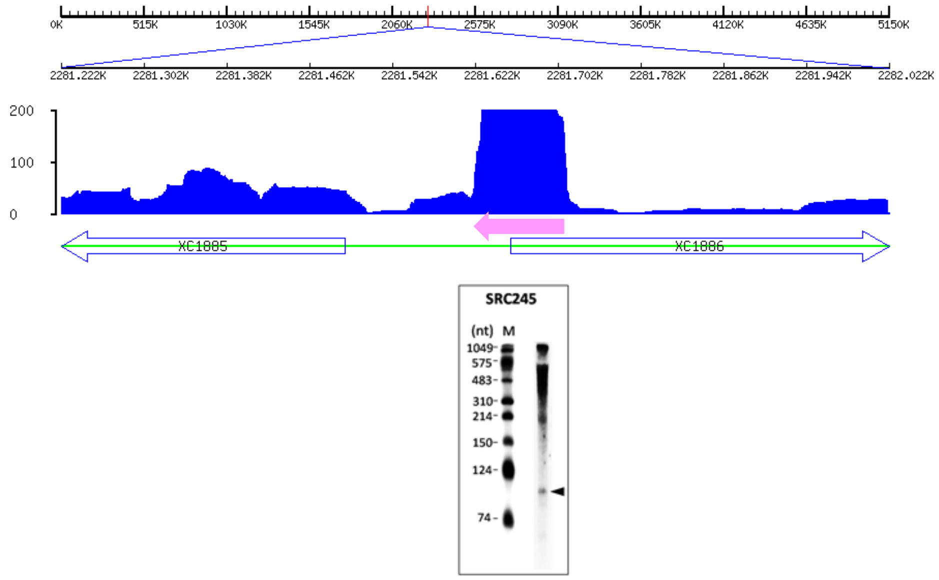

sRNA name	sRNA candidate	Target Transcript	Start	End	Length (nt)	RPKM
sRX084	SRC249	TT274	2303046	2303141	96	240.9

Now ranger of the following image is from 2302646 bp to 2303445 bp , Chromsome zoom value is 1 : 1 , Depth zoom value is 4 : 1

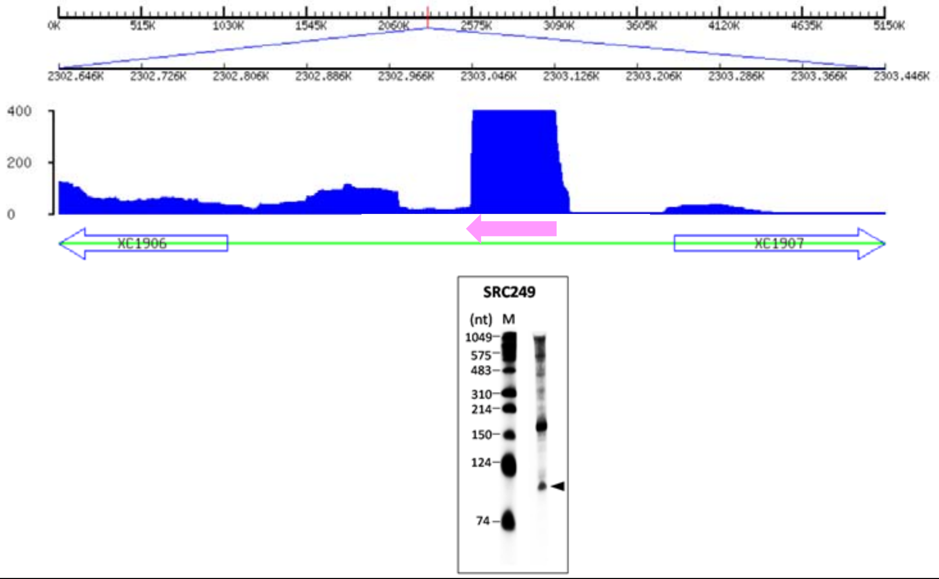

sRNA name	sRNA candidate	Target Transcript	Start	End	Length (nt)	RPKM
sRX085	SRC250	TT275	2334041	2334111	71	412.3
sRX086	SRC251	TT276	2334327	2334413	87	48.7

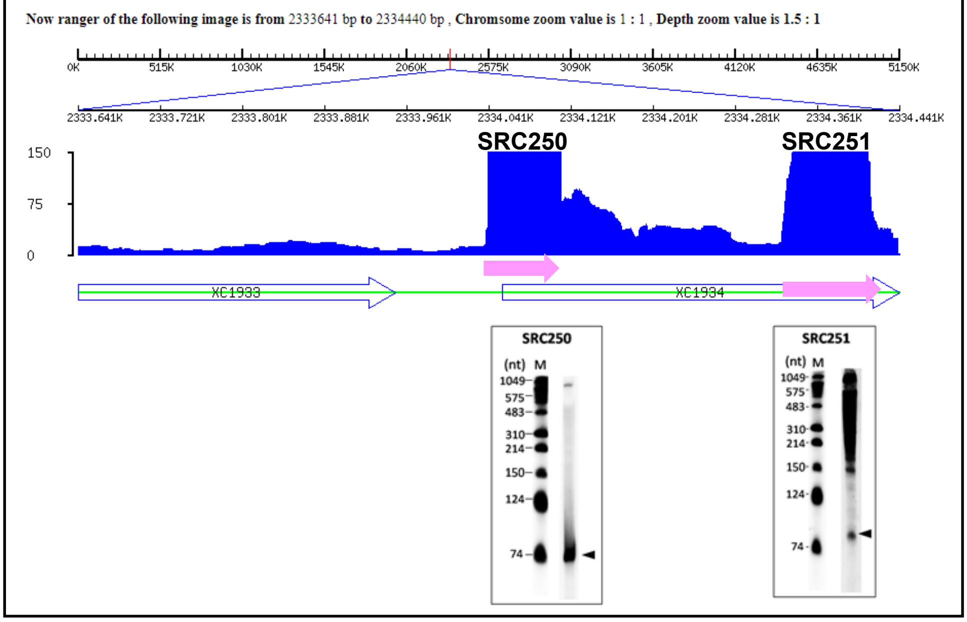

sRNA name	sRNA candidate	Target Transcript	Start	End	Length (nt)	RPKM
sRX087	SRC252	TT277	2358598	2358674	72	915.7

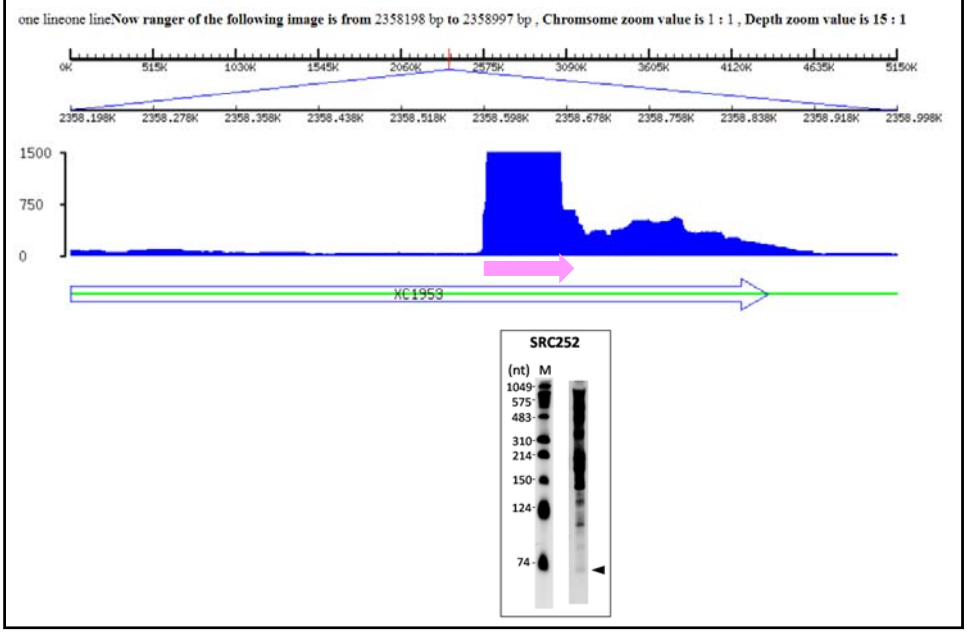

sRNA name	sRNA candidate	Target Transcript	Start	End	Length (nt)	RPKM
sRX088	SRC255	TT286	2418445	2418513	69	719.7

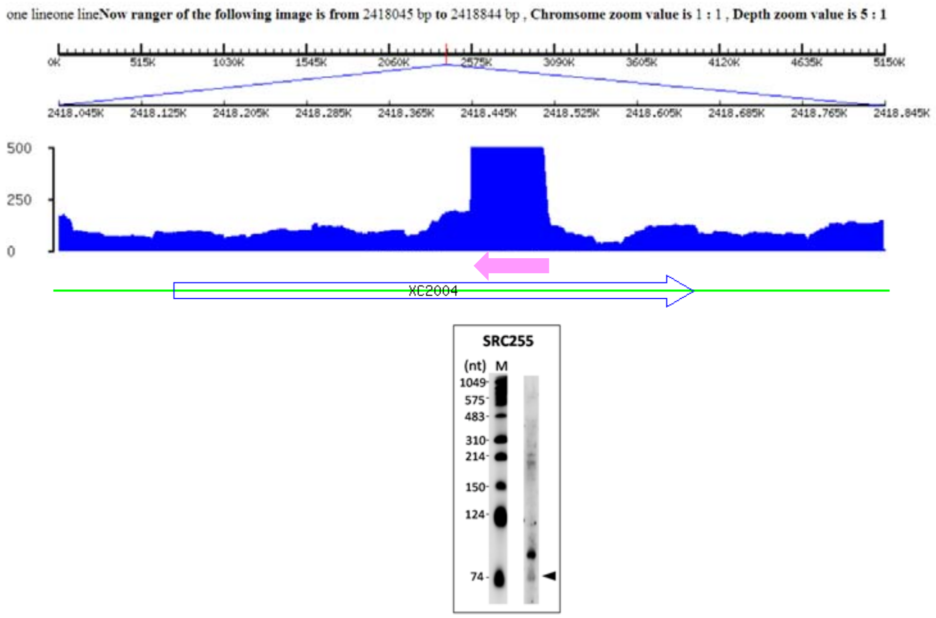

sRNA name	sRNA candidate	Target Transcript	Start	End	Length (nt)	RPKM
sRX089	SRC257	TT288	2428848	2428904	57	389.2

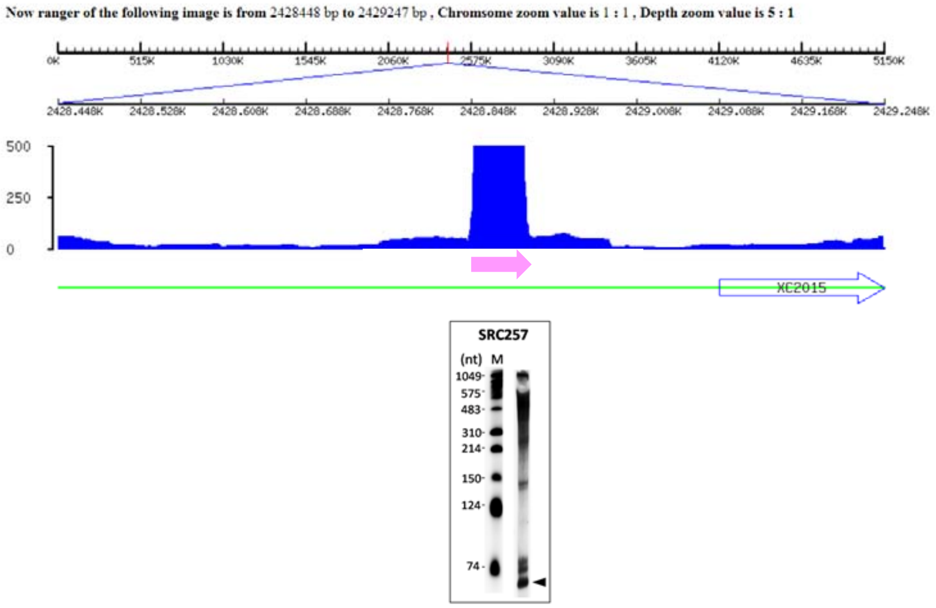

sRNA name	sRNA candidate	Target Transcript	Start	End	Length (nt)	RPKM
sRX090	SRC262	TT293	2443942	2444096	154	95.3

Now ranger of the following image is from 2443542 bp **to** 2444341 bp , **Chromsome zoom value is** 1 : 1 , **Depth zoom value is 0.5 : 1**

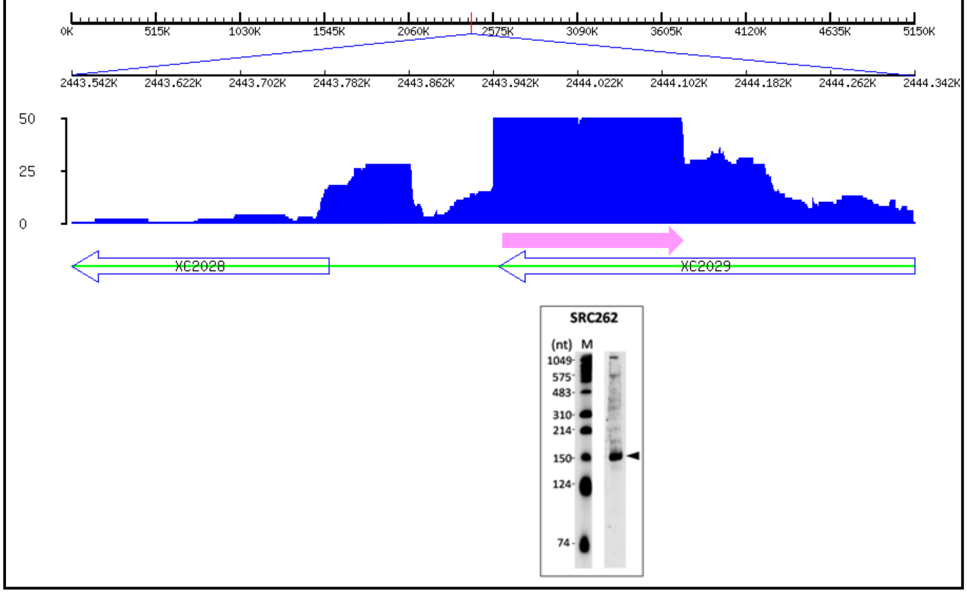

sRNA name	sRNA candidate	Target Transcript	Start	End	Length (nt)	RPKM
sRX091	SRC263	TT294	2455991	2456046	56	114.9

Now ranger of the following image is from 2455591 bp **to** 2456390 bp , **Chromsome zoom value is** 1 : 1 , **Depth zoom value is 2.5 : 1**

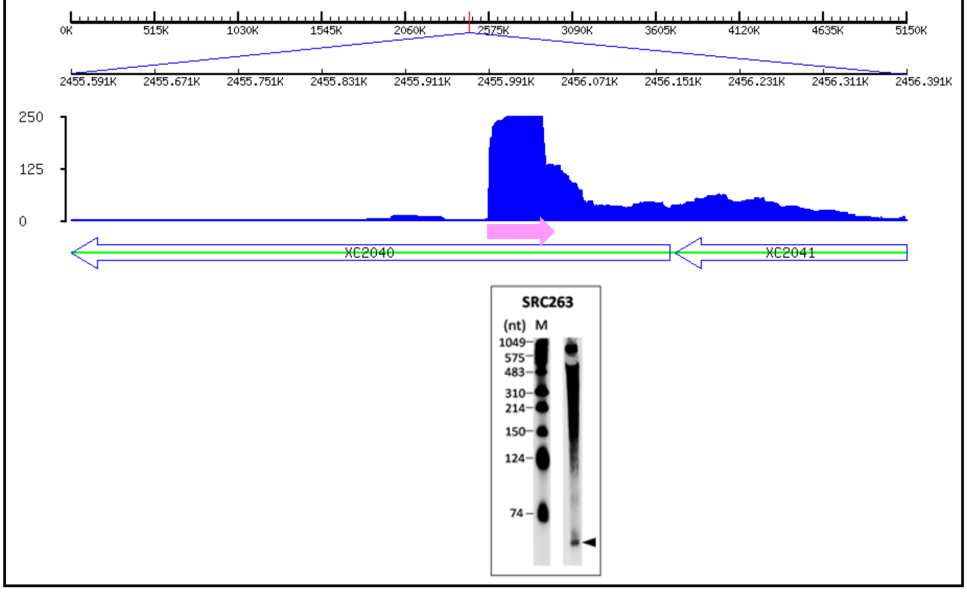

sRNA name	sRNA candidate	Target Transcript	Start	End	Length (nt)	RPKM
sRX092	SRC264	TT295	2458026	2458115	90	85.7

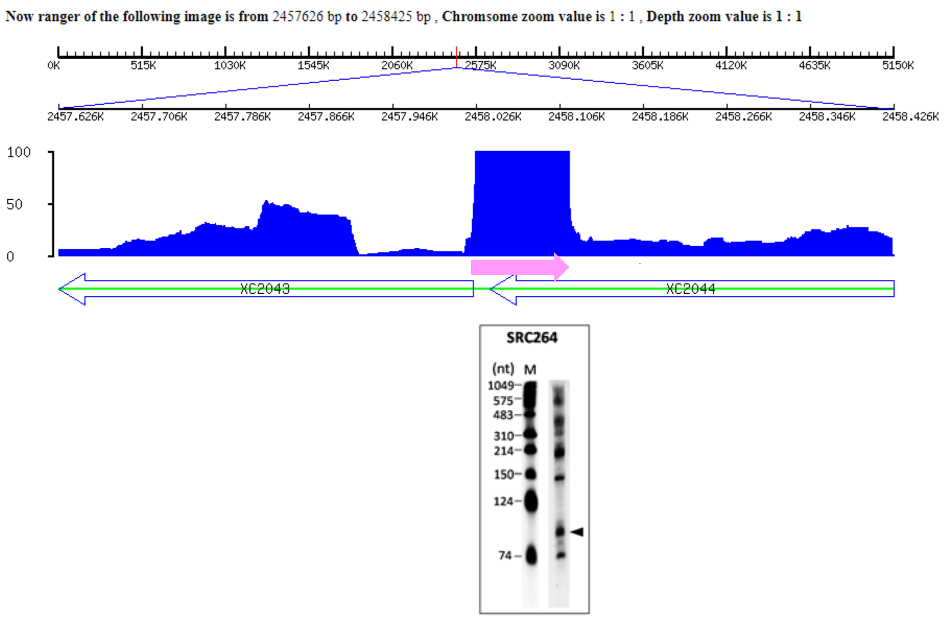

sRNA name	sRNA candidate	Target Transcript	Start	End	Length (nt)	RPKM
sRX093	SRC265	TT296	2509851	2509912	62	89.1

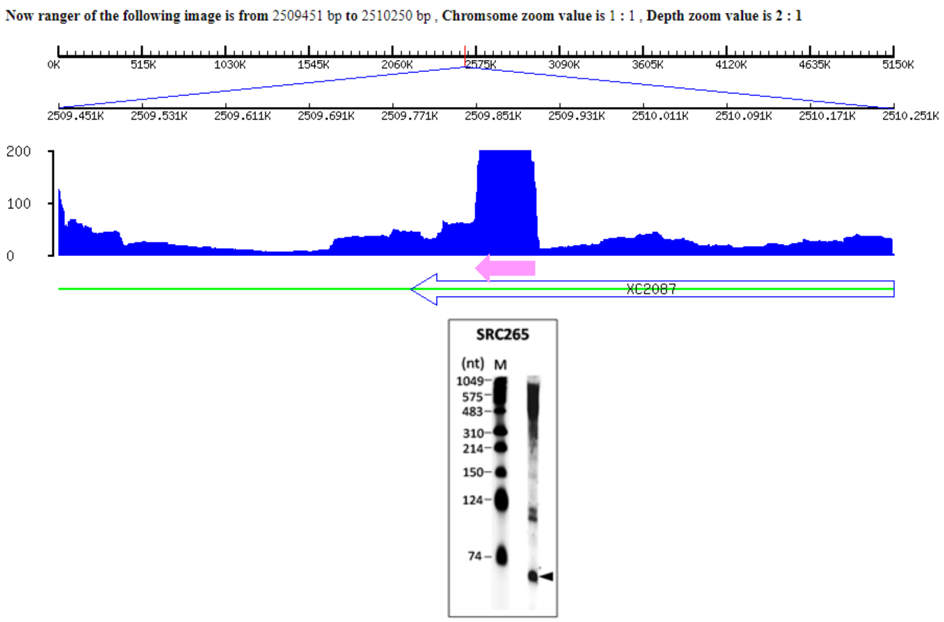

sRNA name	sRNA candidate	Target Transcript	Start	End	Length (nt)	RPKM
sRX094	SRC266	TT297	2513401	2513472	72	411.7

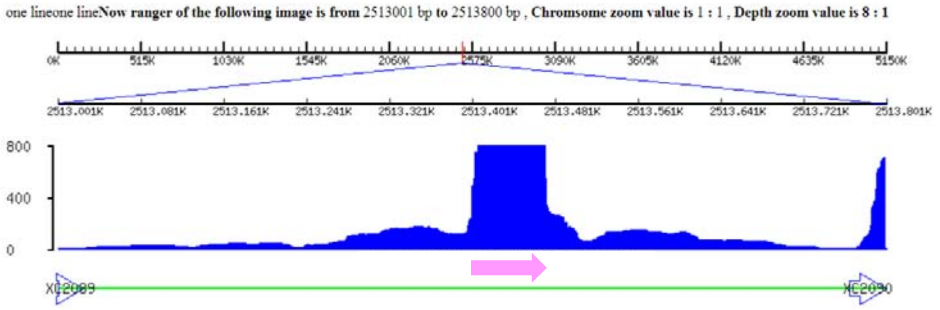

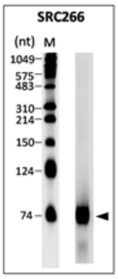

sRNA name	sRNA candidate	Target Transcript	Start	End	Length (nt)	RPKM
sRX095	SRC271	TT303	2551037	2551103	67	44.3

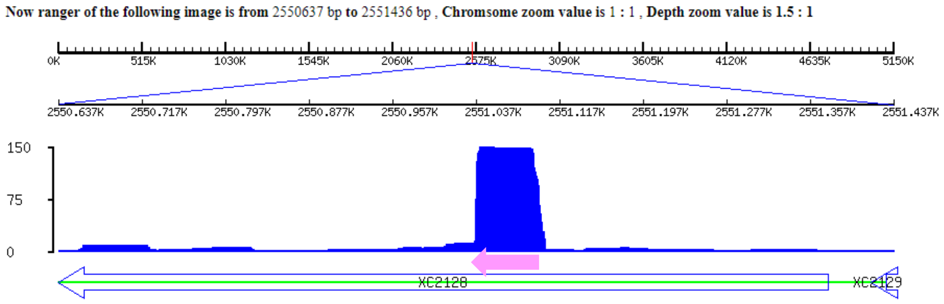

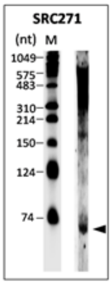

sRNA name	sRNA candidate	Target Transcript	Start	End	Length (nt)	RPKM
sRX096	SRC272	TT304	2566429	2566739	311	93.3

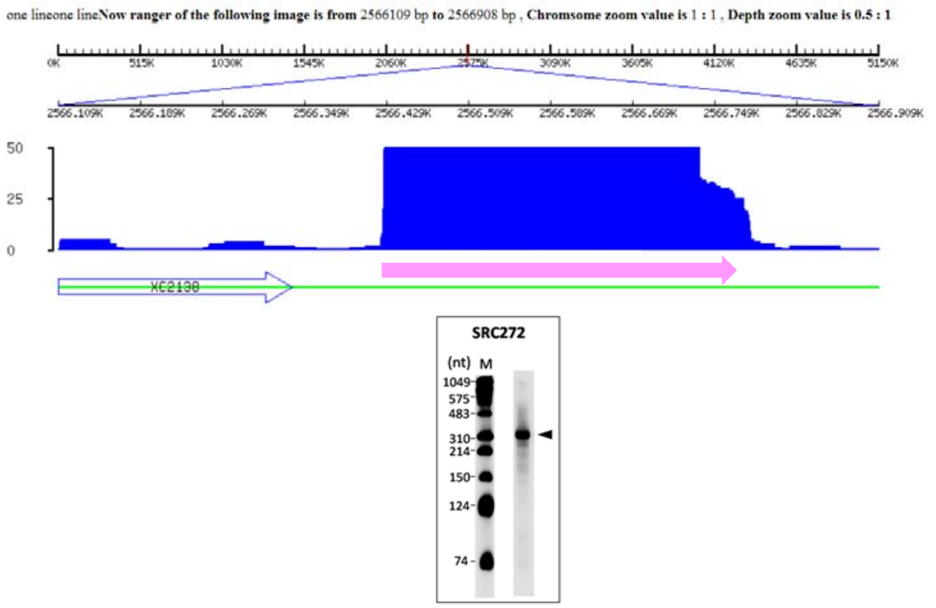

sRNA name	sRNA candidate	Target Transcript	Start	End	Length (nt)	RPKM
sRX097	SRC281	TT313	2656886	2656937	108	444.9

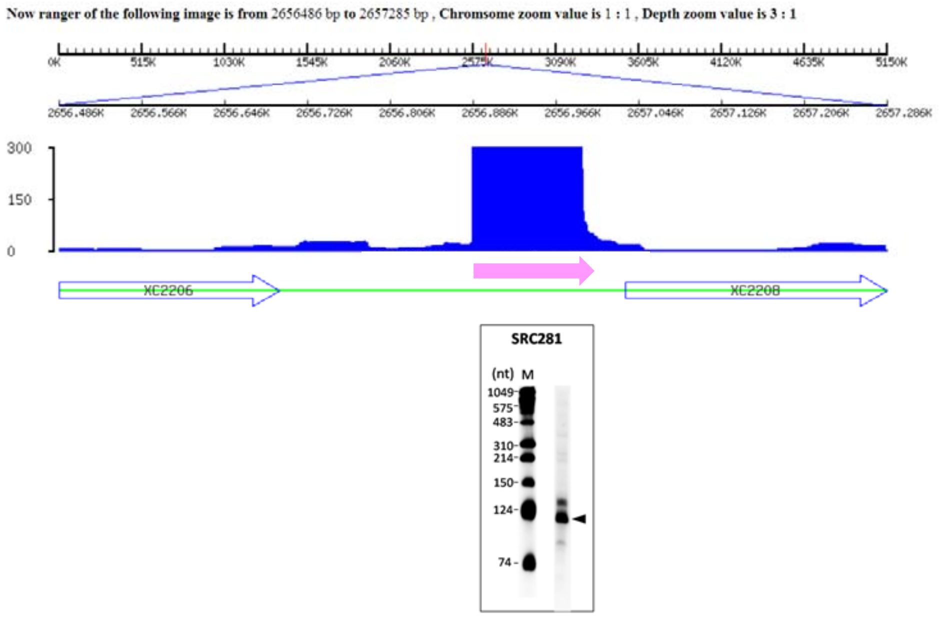

sRNA name	sRNA candidate	Target Transcript	Start	End	Length (nt)	RPKM
sRX098	SRC283	TT315	2669660	2669738	79	109.2

Now ranger of the following image is from 2669260 bp to 2670059 bp , Chromsome zoom value is 1 : 1 , Depth zoom value is 3 : 1

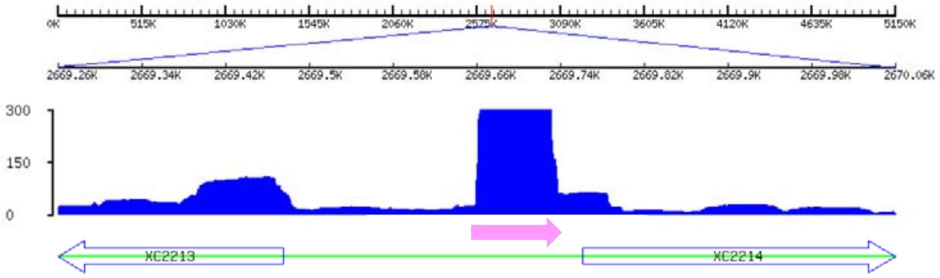

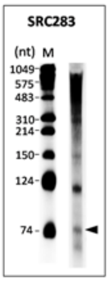

sRNA name	sRNA candidate	Target Transcript	Start	End	Length (nt)	RPKM
sRX099	SRC286	TT319	2675595	2675684	90	686.3

one lineone lineNow ranger of the following image is from 2675195 bp to 2675994 bp , Chromsome zoom value is 1 : 1 , Depth zoom value is 15 : 1

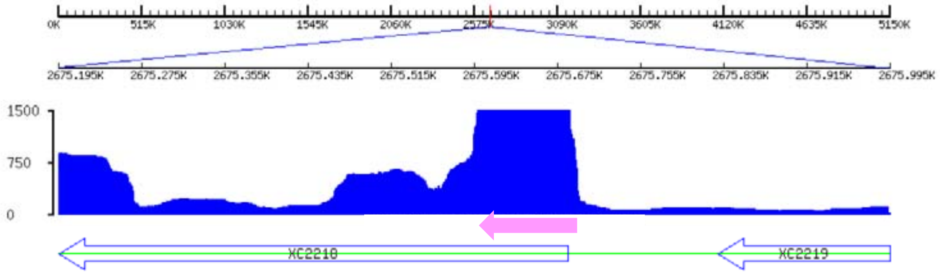

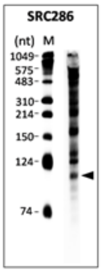

sRNA name	sRNA candidate	Target Transcript	Start	End	Length (nt)	RPKM
sRX100	SRC288	TT321	2686474	2686558	85	143.8

one lineone line**Now ranger of the following image is from** 2686074 bp **to** 2686873 bp , **Chromsome zoom value is** 1 : 1 , **Depth zoom value is 3 : 1**

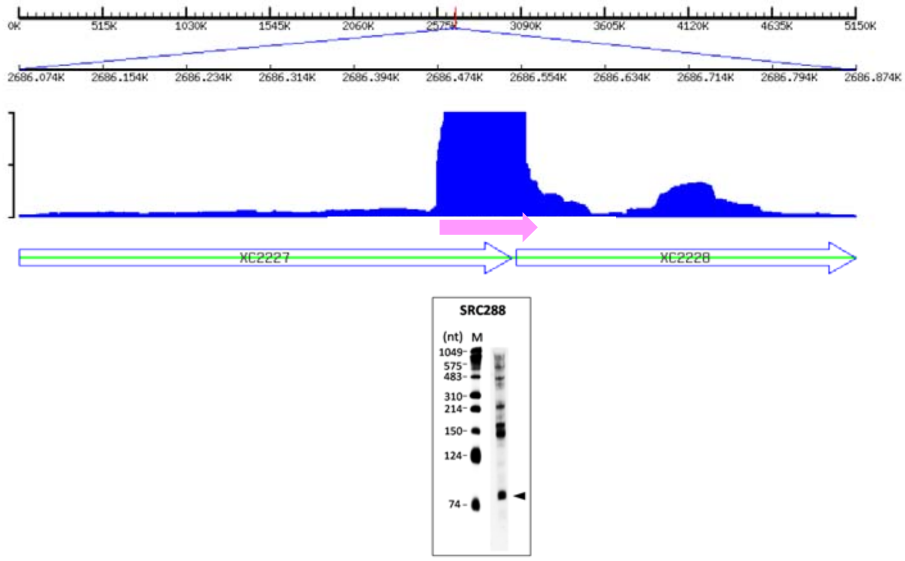

sRNA name	sRNA candidate	Target Transcript	Start	End	Length (nt)	RPKM
sRX101	SRC289	TT322	2687002	2687059	58	37.4

Now ranger of the following image is from 2686602 bp **to** 2687401 bp , **Chromsome zoom value is** 1 : 1 , **Depth zoom value is 1 : 1**

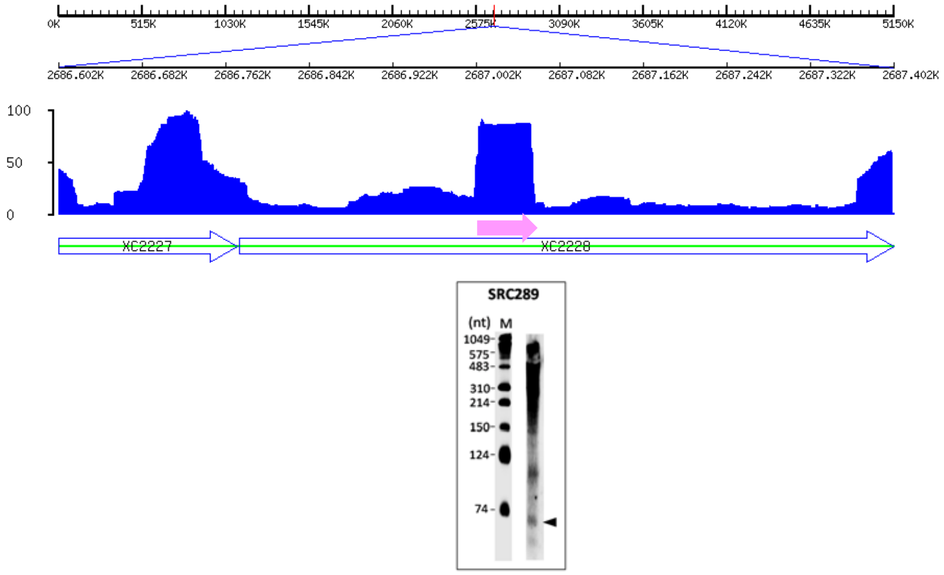

sRNA name	sRNA candidate	Target Transcript	Start	End	Length (nt)	RPKM
sRX102	SRC291	TT324	2696762	2696842	81	437.1

Now ranger of the following image is from 2696362 bp to 2697161 bp , Chromsome zoom value is 1 : 1 , Depth zoom value is 14 : 1

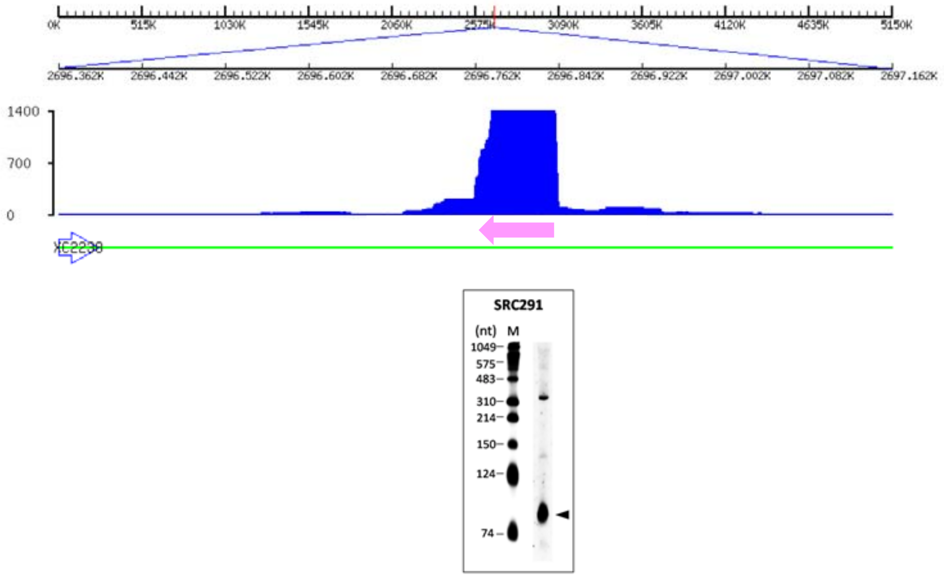

sRNA name	sRNA candidate	Target Transcript	Start	End	Length (nt)	RPKM
sRX103	SRC293	TT326	2708703	2708768	66	346.8

Now ranger of the following image is from 2708303 bp to 2709102 bp , Chromsome zoom value is 1 : 1 , Depth zoom value is 5 : 1

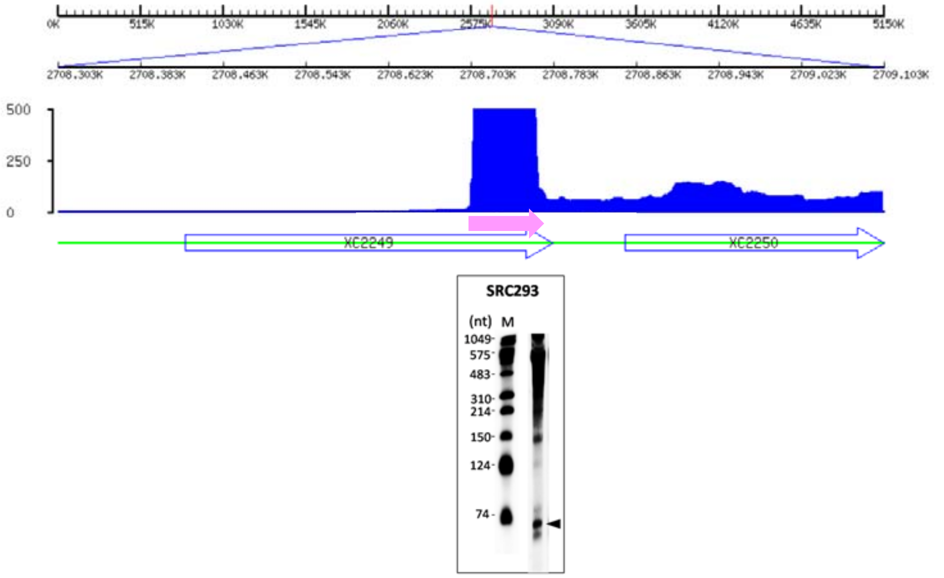

sRNA name	sRNA candidate	Target Transcript	Start	End	Length (nt)	RPKM
sRX104	SRC295	TT328	2711448	2711512	64	795.6

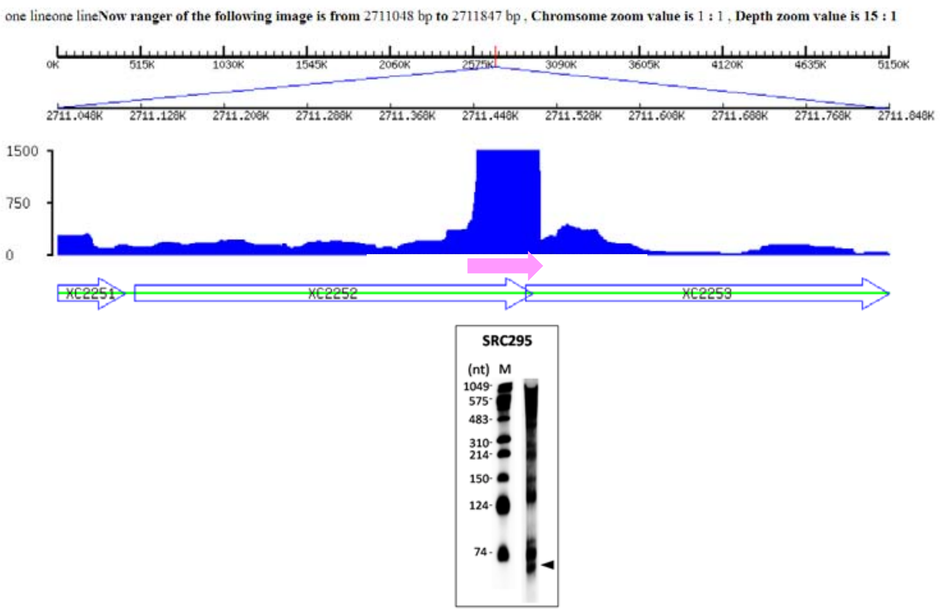

sRNA name	sRNA candidate	Target Transcript	Start	End	Length (nt)	RPKM
sRX105	SRC299	TT332	2760980	2761064	84	395.3

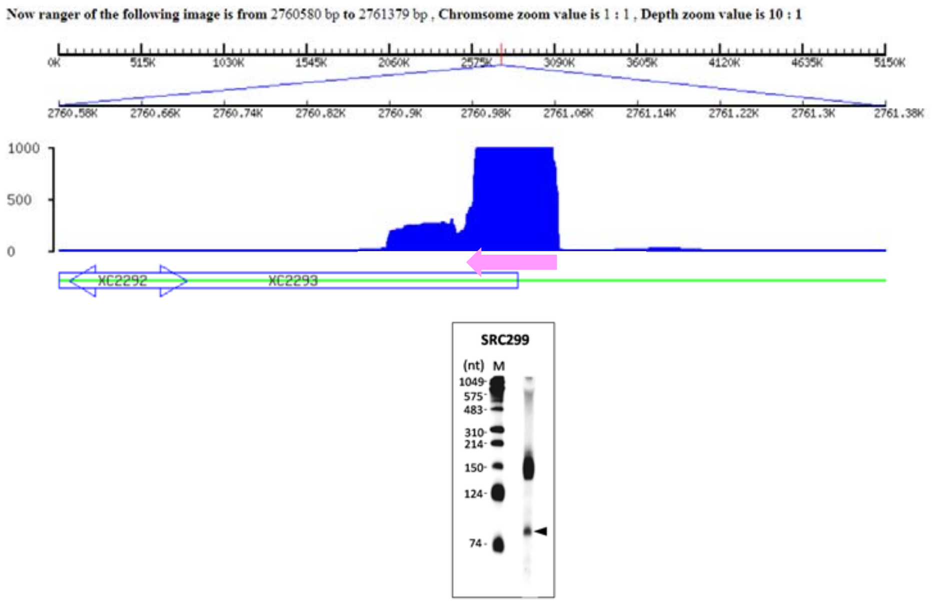

sRNA name	sRNA candidate	Target Transcript	Start	End	Length (nt)	RPKM
sRX106	SRC303	TT337	2919083	2919303	220	244.1

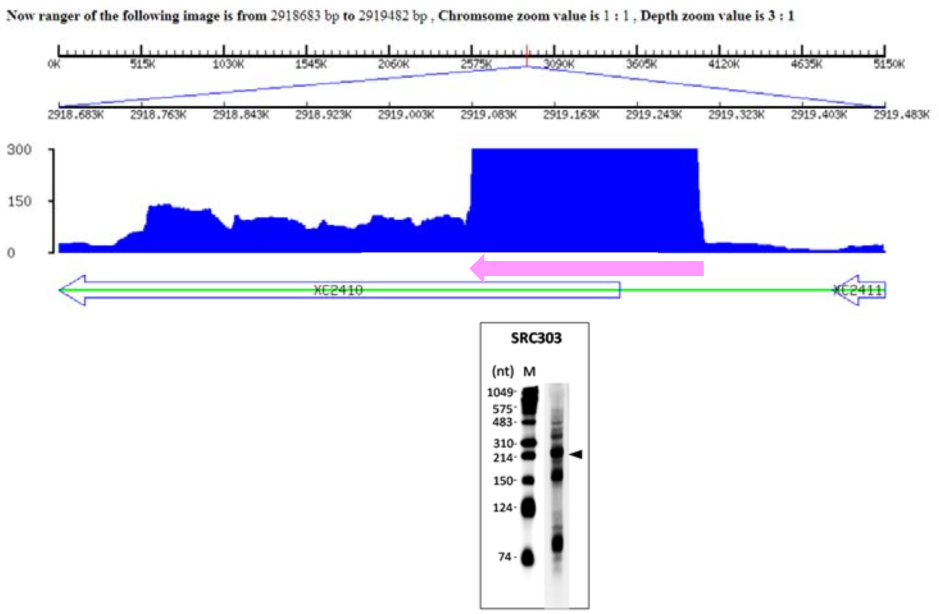

sRNA name	sRNA candidate	Target Transcript	Start	End	Length (nt)	RPKM
sRX107	SRC307	TT341	2939567	2939752	185	8230

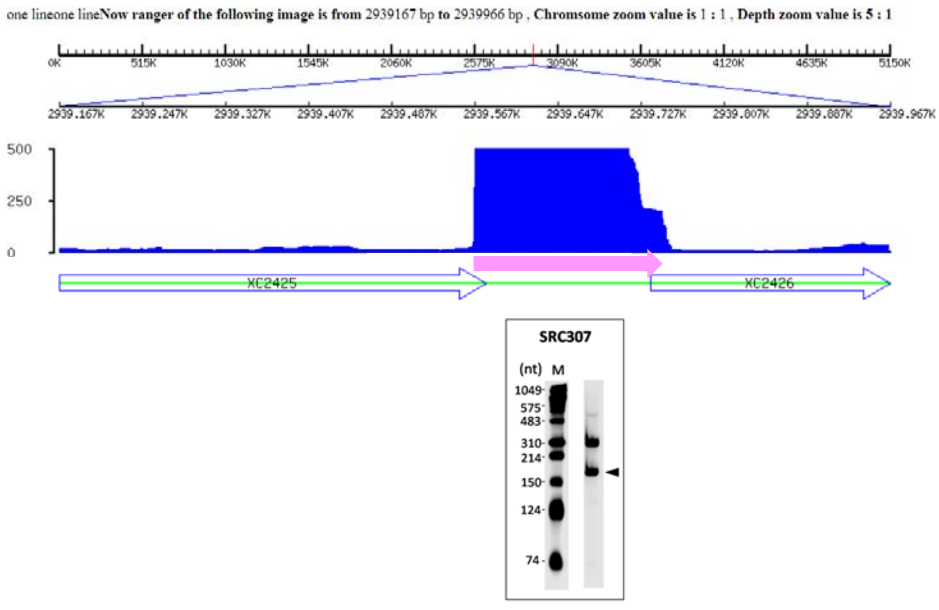

sRNA name	sRNA candidate	Target Transcript	Start	End	Length (nt)	RPKM
sRX108	SRC309	TT343	2940875	2940934	60	151.9

Now ranger of the following image is from 2940475 bp to 2941274 bp , Chromsome zoom value is 1 : 1 , Depth zoom value is 3 : 1

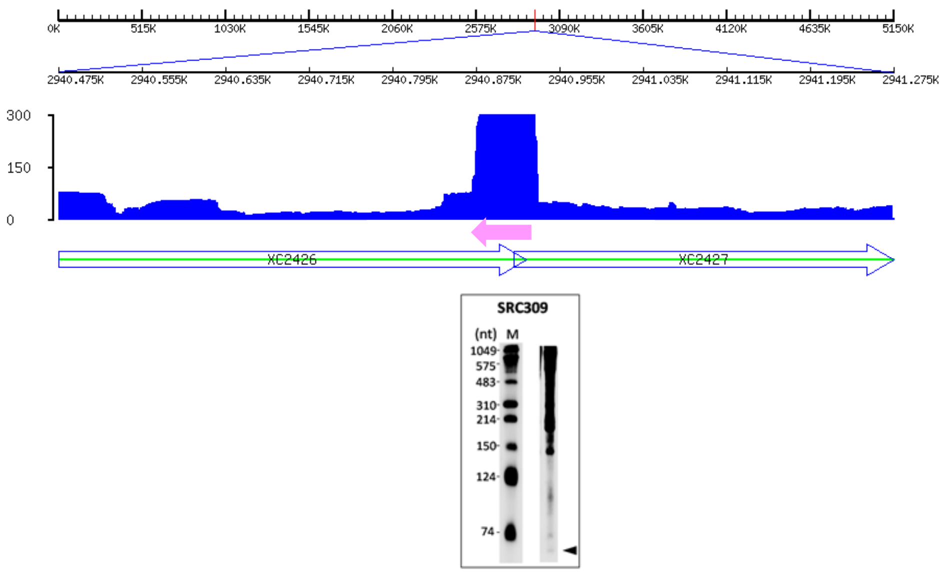

sRNA name	sRNA candidate	Target Transcript	Start	End	Length (nt)	RPKM
sRX109	SRC310	TT344	2944151	2944205	55	924.9

Now ranger of the following image is from 2943751 bp to 2944550 bp , Chromsome zoom value is 1 : 1 , Depth zoom value is 15 : 1

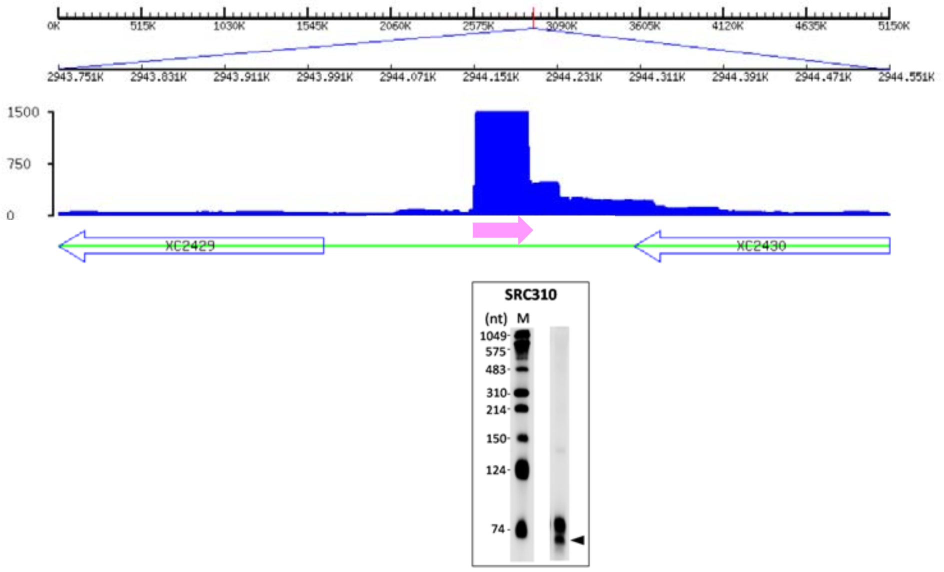

sRNA name	sRNA candidate	Target Transcript	Start	End	Length (nt)	RPKM
sRX110	SRC312	TT346	2972679	2972743	65	230.1

Now ranger of the following image is from 2972279 bp to 2973078 bp , Chromsome zoom value is 1 : 1 , Depth zoom value is 4 : 1

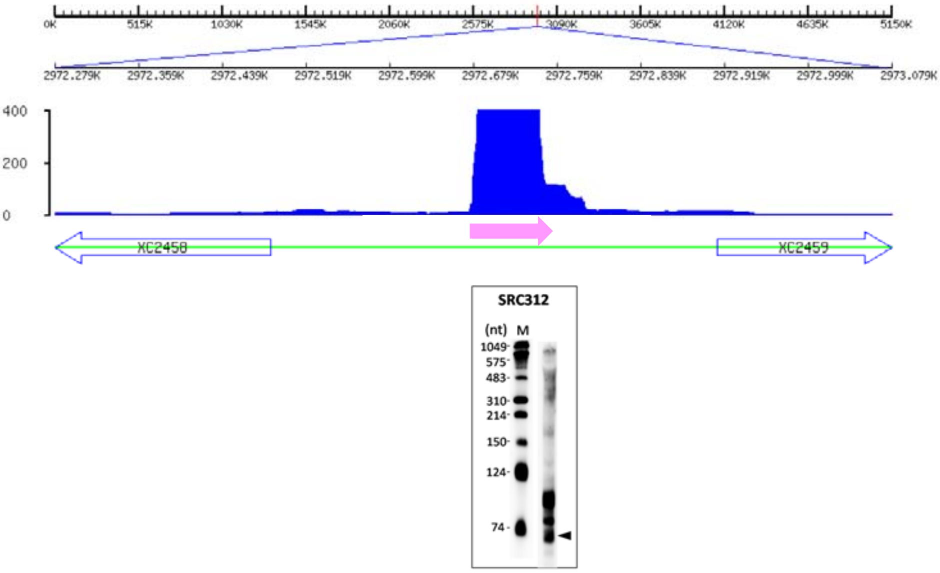

sRNA name	sRNA candidate	Target Transcript	Start	End	Length (nt)	RPKM
sRX111	SRC315	TT350	3028347	3028440	93	752.9

Now ranger of the following image is from 3027947 bp to 3028746 bp , Chromsome zoom value is 1 : 1 , Depth zoom value is 23 : 1

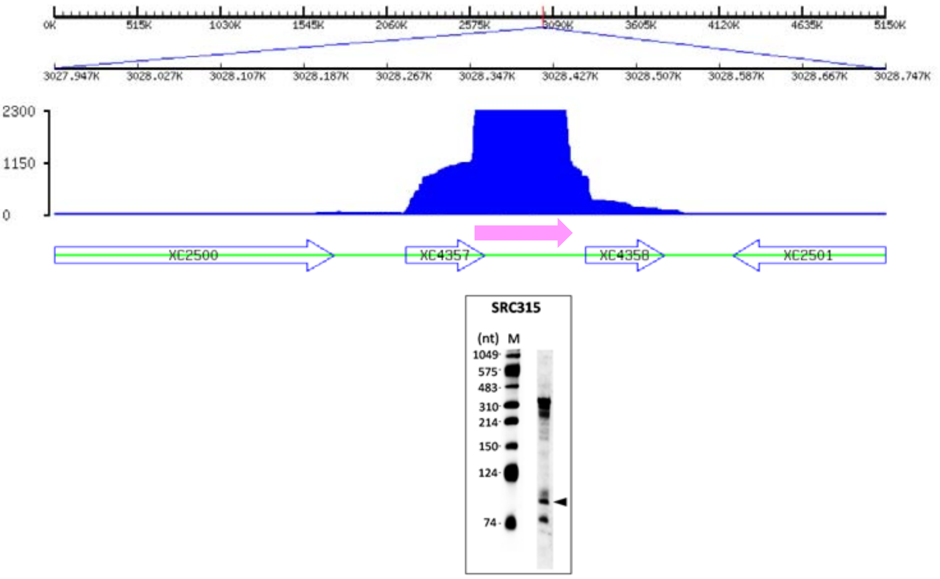

sRNA name	sRNA candidate	Target Transcript	Start	End	Length (nt)	RPKM
sRX112	SRC318	TT354	3038922	3038984	63	709.1

Now ranger of the following image is from 3038211 bp to 3039010 bp , Chromsome zoom value is 1 : 1 , Depth zoom value is 2 : 1

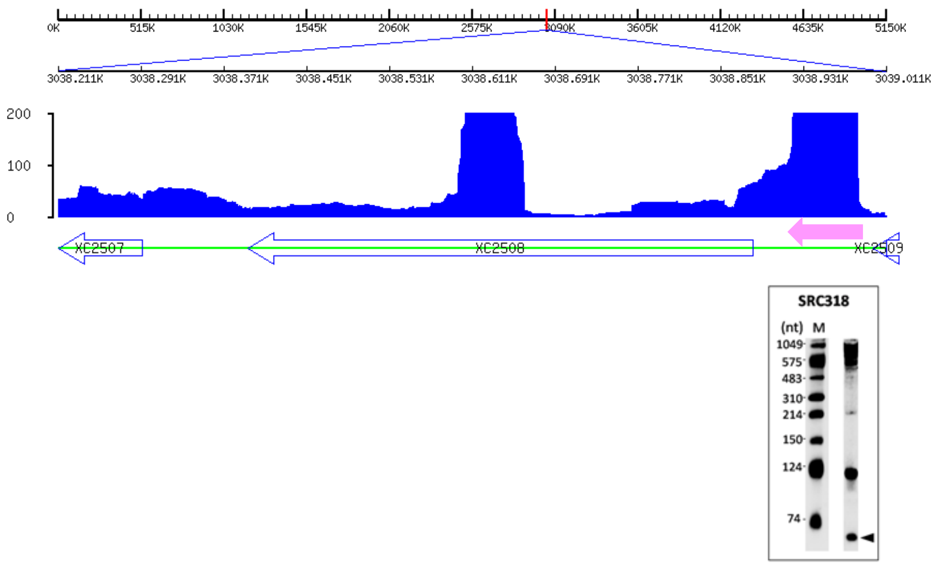

sRNA name	sRNA candidate	Target Transcript	Start	End	Length (nt)	RPKM
sRX113	SRC319	TT355	3039995	3040047	53	133.5

Now ranger of the following image is from 3039595 bp to 3040394 bp , Chromsome zoom value is 1 : 1 , Depth zoom value is 2 : 1

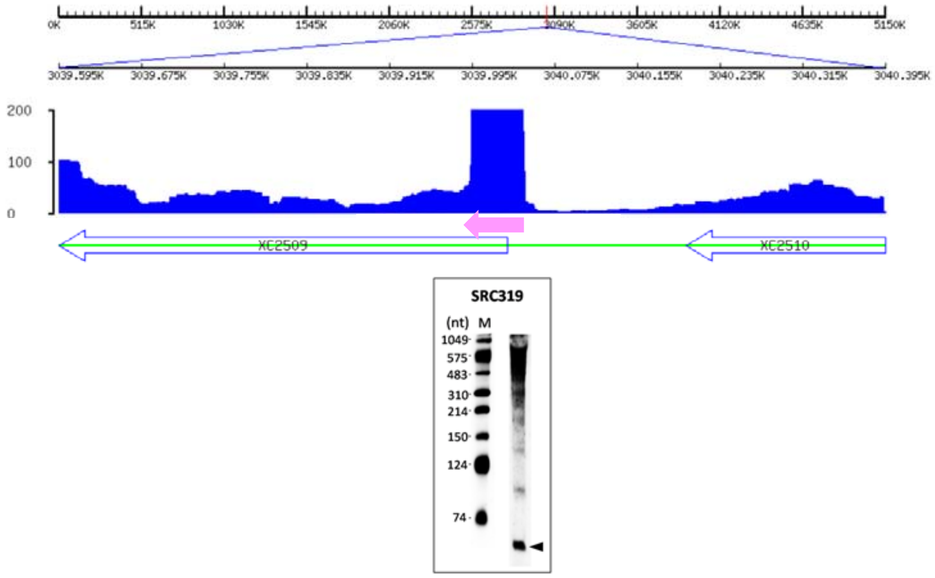

sRNA name	sRNA candidate	Target Transcript	Start	End	Length (nt)	RPKM
sRX114	SRC326	TT362	3066078	3066187	110	534.3

Now ranger of the following image is from 3065678 bp to 3066477 bp , Chromsome zoom value is 1 : 1 , Depth zoom value is 10 : 1

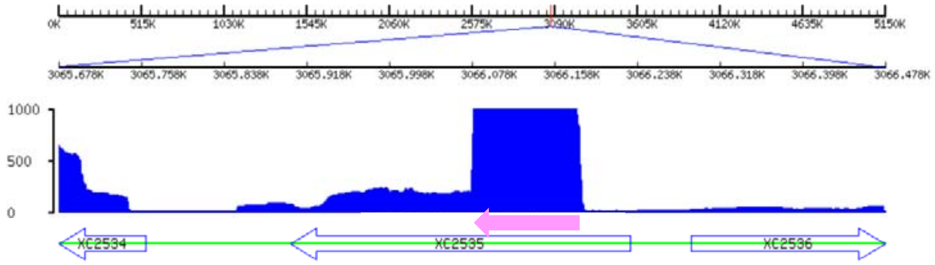

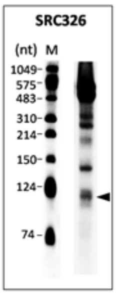

sRNA name	sRNA candidate	Target Transcript	Start	End	Length (nt)	RPKM
sRX115	SRC341	TT378	3188220	3188336	117	167.4

Now ranger of the following image is from 3187820 bp to 3188619 bp , Chromsome zoom value is 1 : 1 , Depth zoom value is 3 : 1

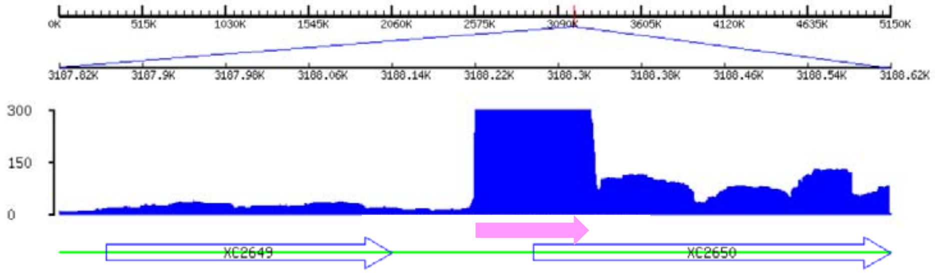

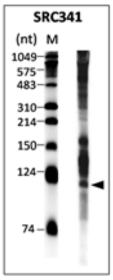

sRNA name	sRNA candidate	Target Transcript	Start	End	Length (nt)	RPKM
sRX116	SRC342	TT379	3188900	3188954	55	1677.7

Now ranger of the following image is from 3188500 bp to 3189299 bp , Chromsome zoom value is 1 : 1 , Depth zoom value is 30 : 1

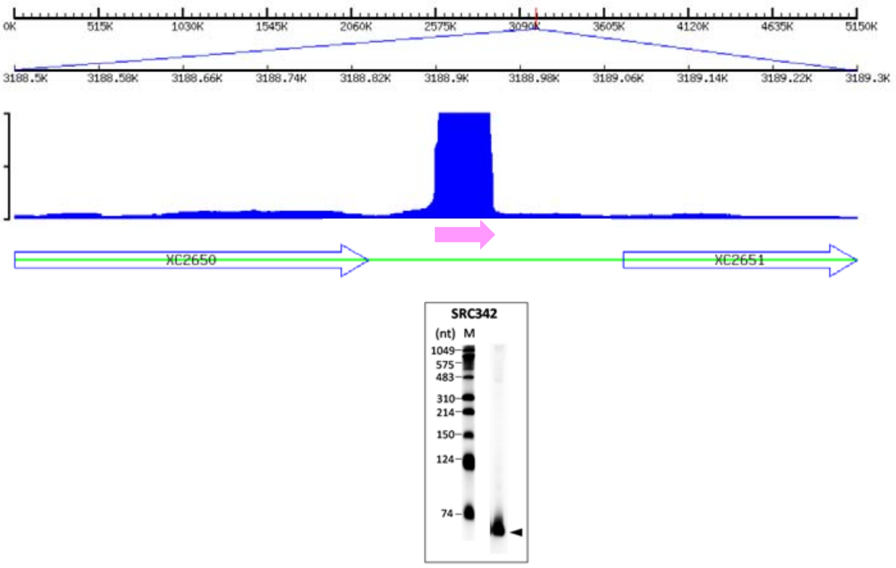

sRNA name	sRNA candidate	Target Transcript	Start	End	Length (nt)	RPKM
sRX117	SRC344	TT381	3245880	3245946	67	843.6

Now ranger of the following image is from 3245480 bp to 3246279 bp , Chromsome zoom value is 1 : 1 , Depth zoom value is 25 : 1

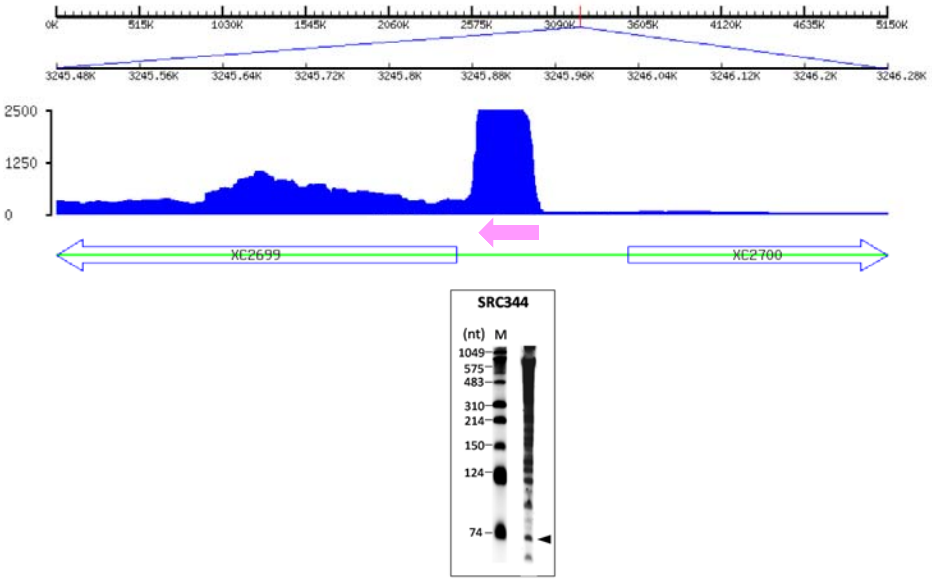

Supplement: Supplementary file 3 — FIGURE S3 Detection of the expression of sRNAs in Xcc by northern blotting. The bacterial cells of Xcc wild‐type strain 8,004 were cultured in the minimal medium MMX to mid‐log phase and total RNAs were isolated from the cells. Three micrograms of total RNA was separated by PAGE and transferred to a positively charged nylon membrane. After UV‐crosslinking, the membrane was hybridized with a DIG‐labelled RNA probe at 68 °C for 8 hr and then signal bands were detected. To ensure the accuracy in evaluation of the size of signal bands, DIG‐labelled RNA molecular weight marker (M) was loaded in each PAGE gel. The filled triangle inside the northern blotting result picture indicates the position corresponding to the size of the target SRC predicted by RNA‐Seq. Above the northern blotting result picture is the visualized mapping pattern of the corresponding SRC. The pink arrow indicates the transcriptional direction of the SRC. The lowermost arrow indicates the protein‐coding gene and its transcriptional orientation and the gene’s ID is shown inside the arrow. The y axis represents the number of the mapped reads. RPKM, reads per kilo bases per million reads [file MPP-21-1573-s003.pdf]
