# Supplementary material for: Genome‐wide screen and functional analysis in Xanthomonas reveal a large number of mRNA‐derived sRNAs, including the novel RsmA‐sequester RsmU
Source: Mol Plant Pathol. 2020 Sep 23;21(12):1573–90. doi: 10.1111/mpp.12997 (PMC7694677; doi:10.1111/mpp.12997)
Supplement: Supplementary file 4 — FIGURE S4 The predicted secondary structures of the sRNAs detectable by northern blotting. The secondary structures were predicted by using M‐fold online software (http://unafold.rna.albany.edu/?q=mfold/RNA‐Folding‐Form) (Zuker, 2003). One representative structure for each sRNA is shown [file MPP-21-1573-s004.pdf]

Fig. S4. The predicted secondary structures of the sRNAs detectable by Northern blotting. The secondary structures were predicted by using M-fold online software (http://unafold.rna.albany.edu/?q=mfold/RNA-Folding-Form) (Zuker, 2003). One representative structure for each sRNA was shown.

REFERENCE

Zuker, M. (2003) Mfold web server for nucleic acid folding and hybridization prediction. *Nucleic Acids Research*, 31, 3406-3415.

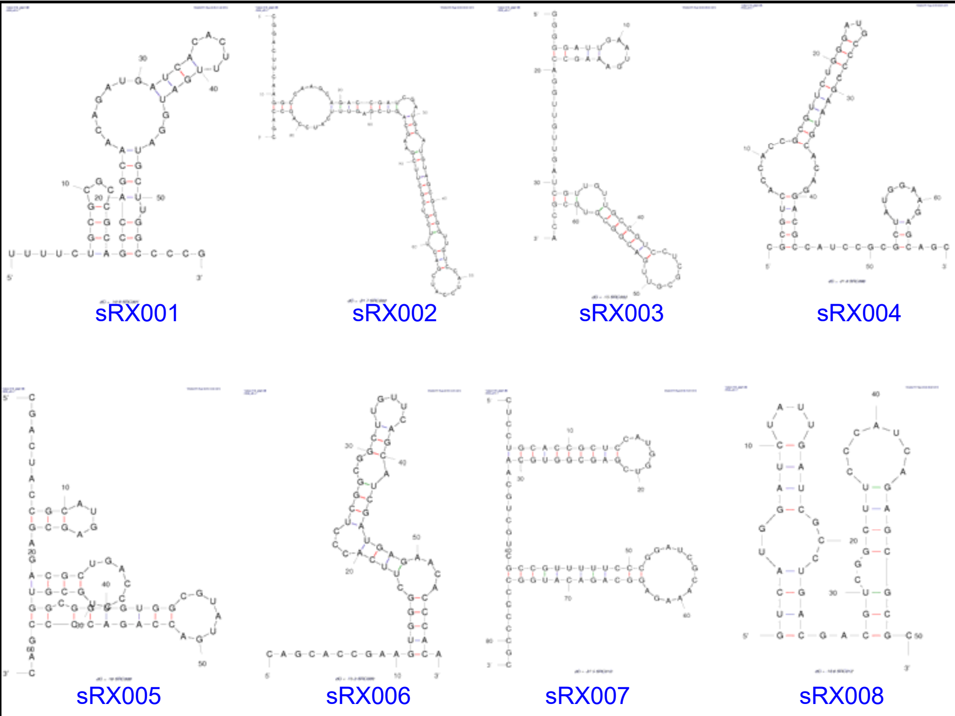

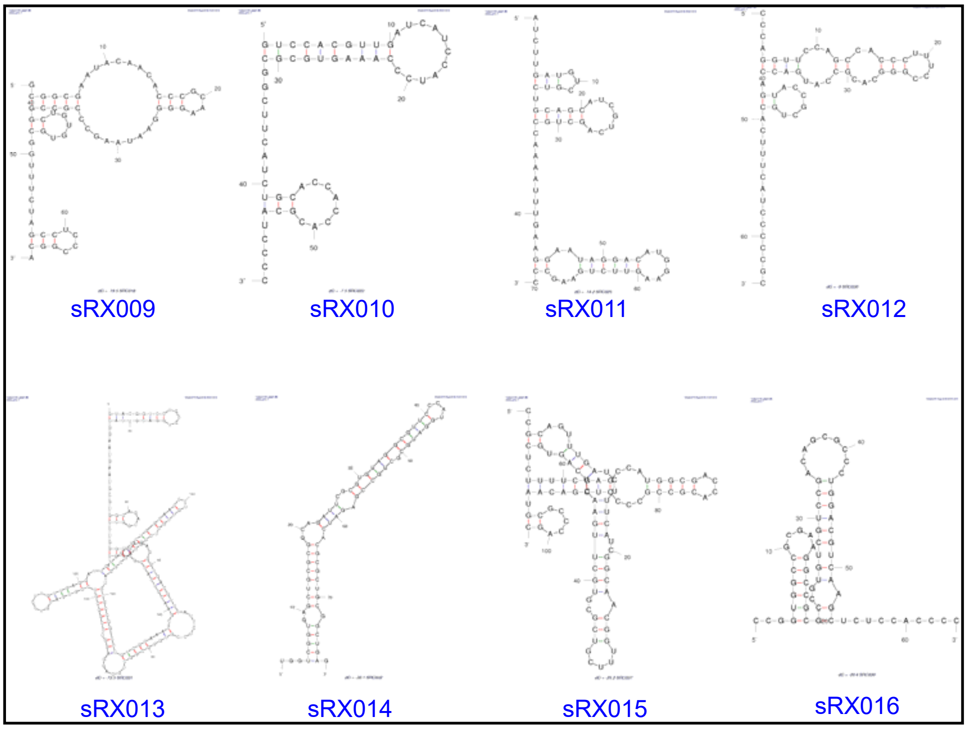
sRX009
sRX010
sRX011
sRX012
sRX013
sRX014
sRX015
sRX016

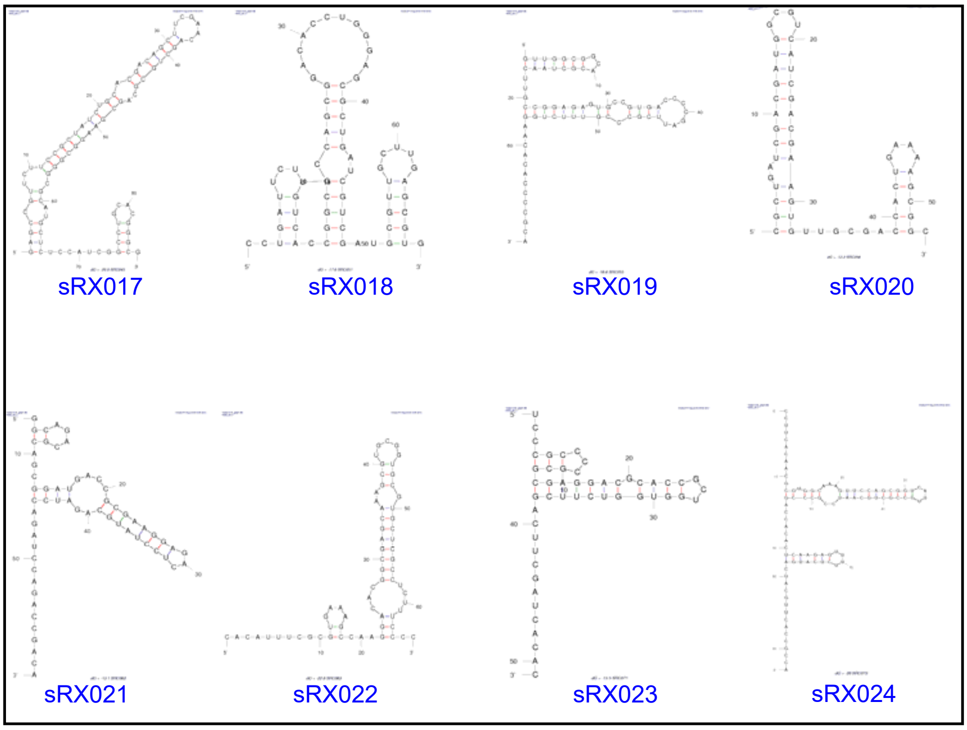
sRX017
sRX018
sRX019
sRX020
sRX021
sRX022
sRX023
sRX024

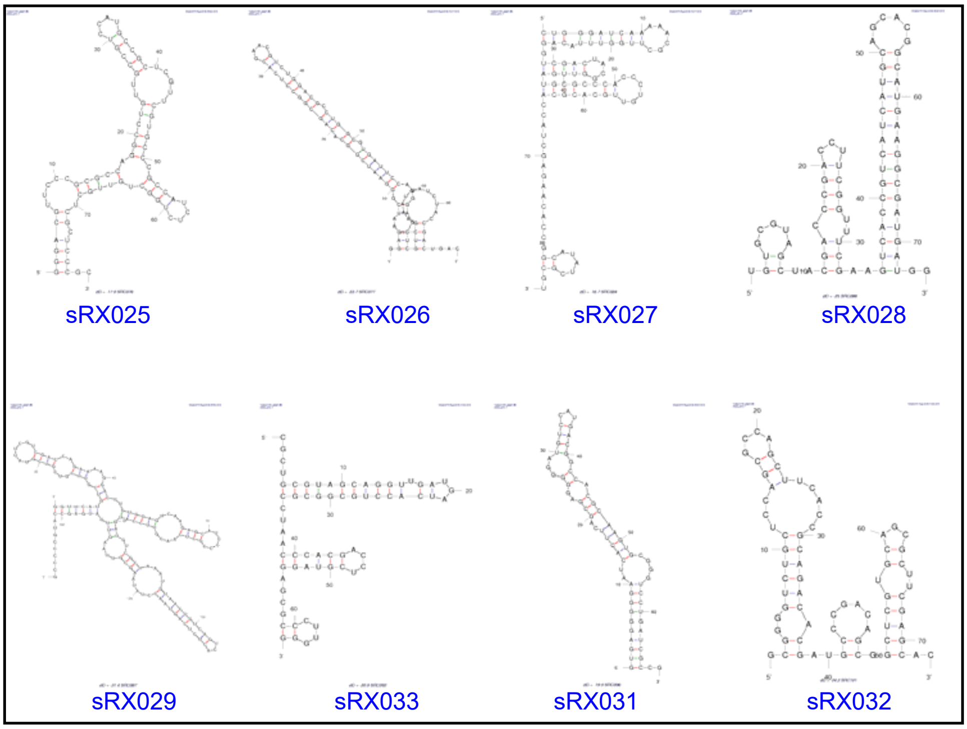
sRX025
sRX026
sRX027
sRX028
sRX029
sRX033
sRX031
sRX032

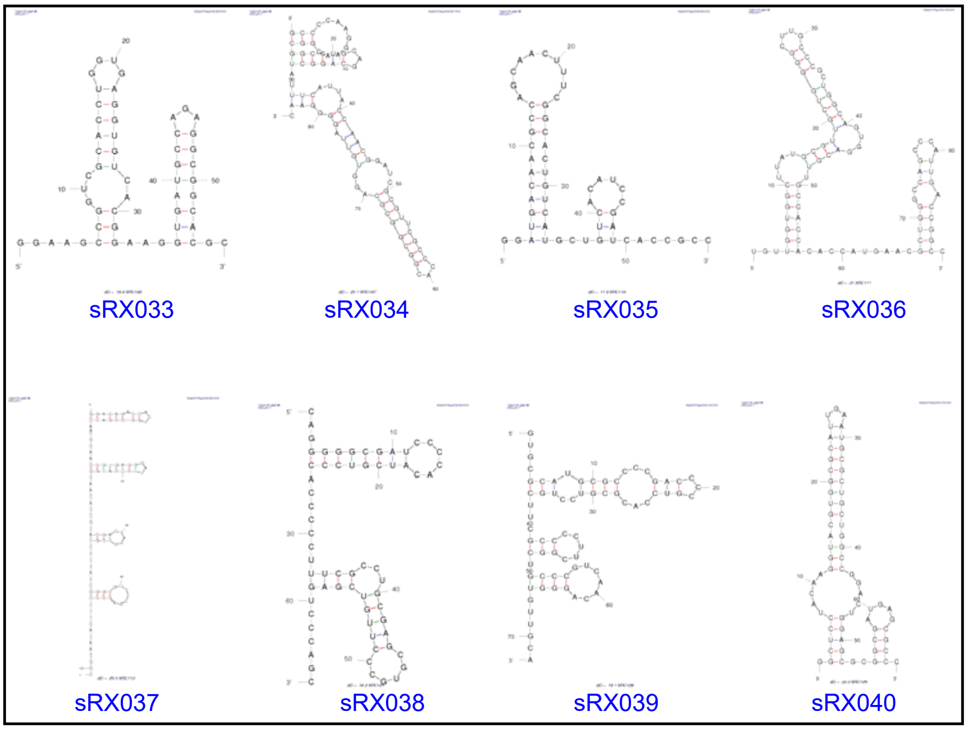
sRX033
sRX034
sRX035
sRX036
sRX037
sRX038
sRX039
sRX040

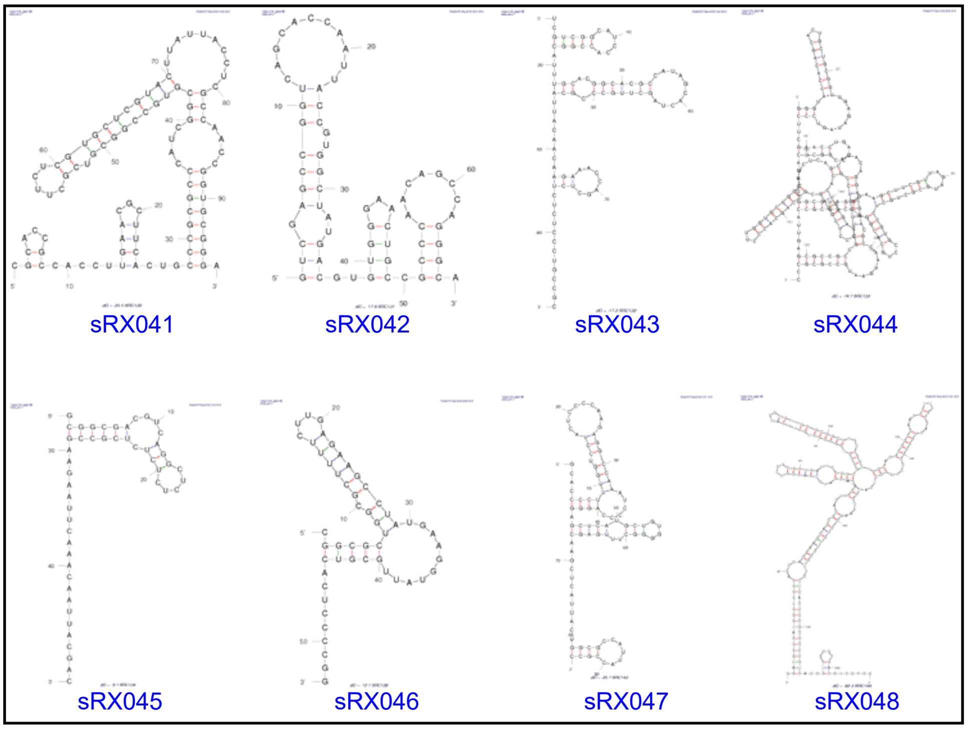
sRX041
sRX042
sRX043
sRX044
sRX045
sRX046
sRX047
sRX048

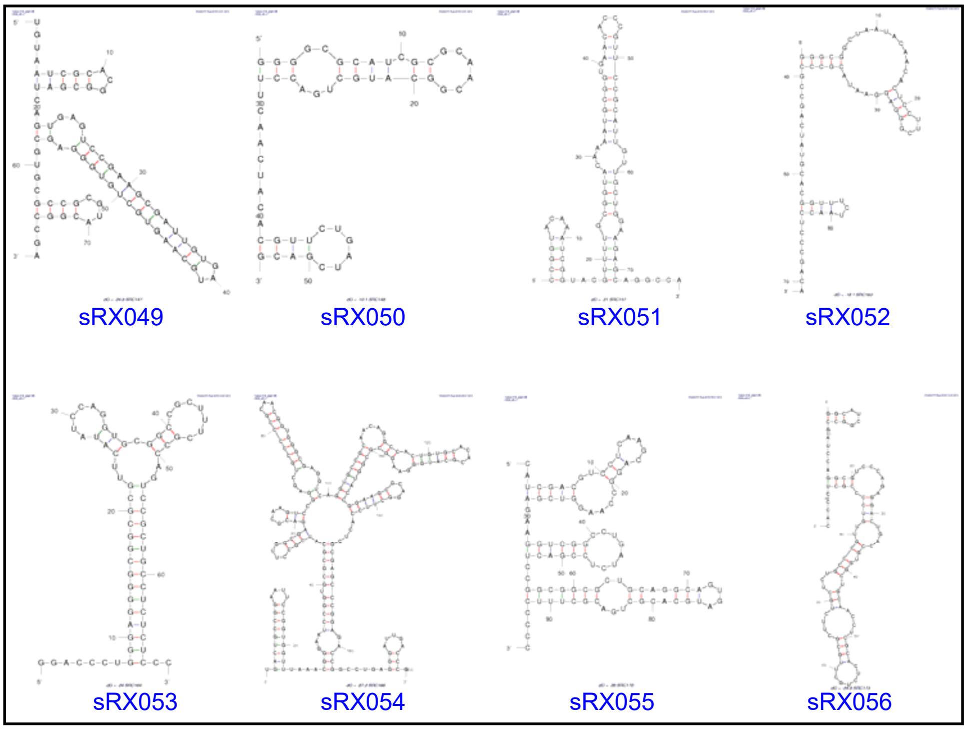
sRX049
sRX050
sRX051
sRX052
sRX053
sRX054
sRX055
sRX056

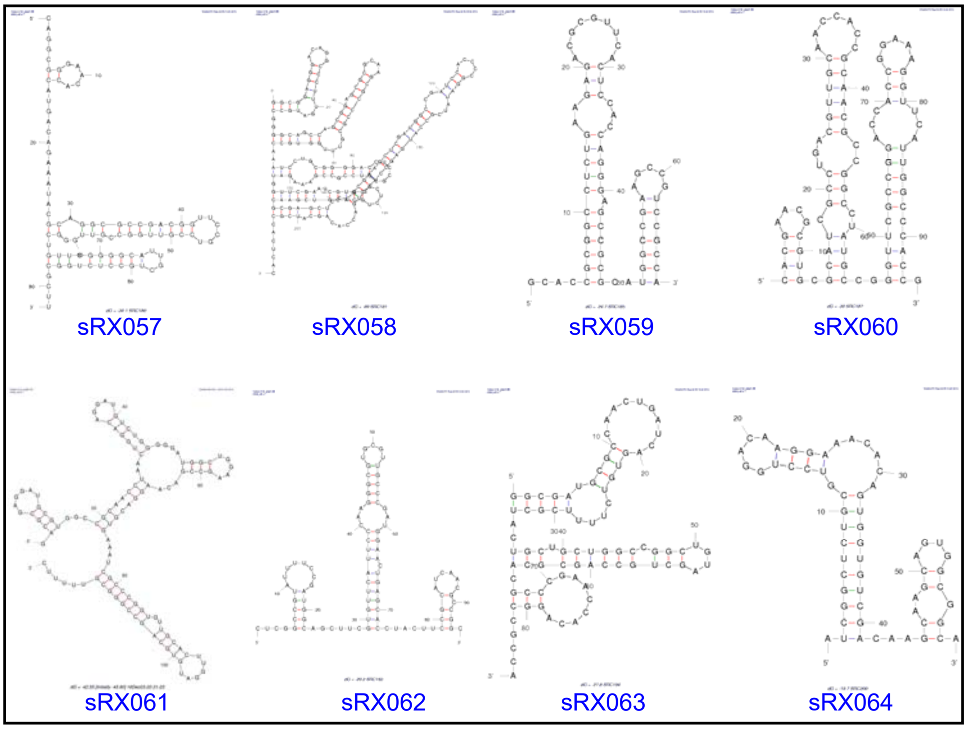
sRX057
sRX058
sRX059
sRX060
sRX061
sRX062
sRX063
sRX064

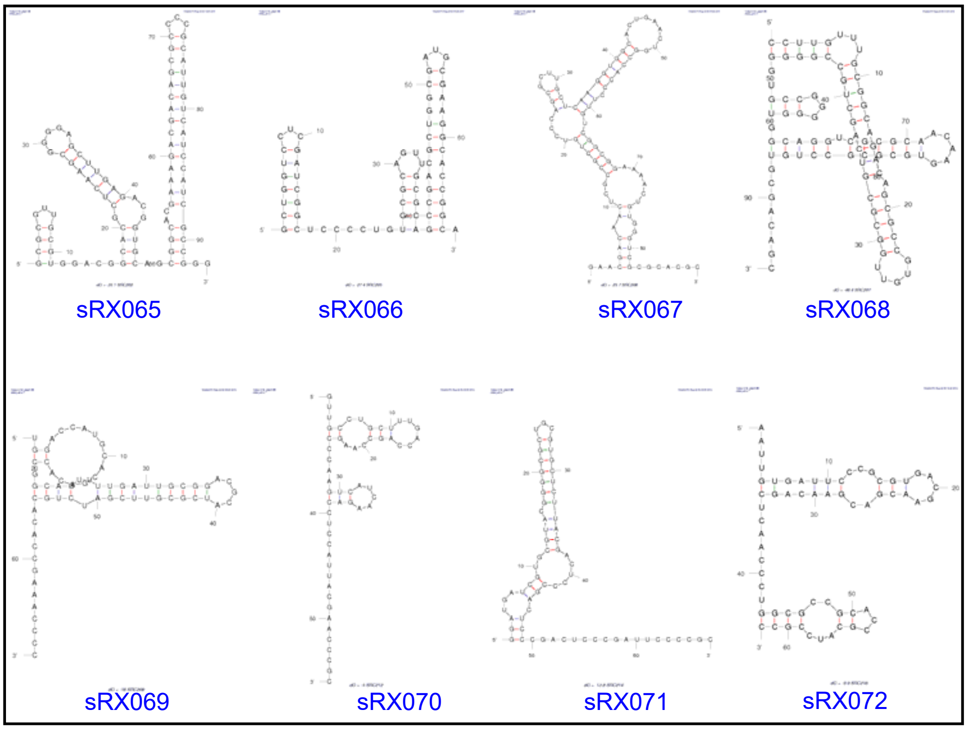
sRX065
sRX066
sRX067
sRX068
sRX069
sRX070
sRX071
sRX072

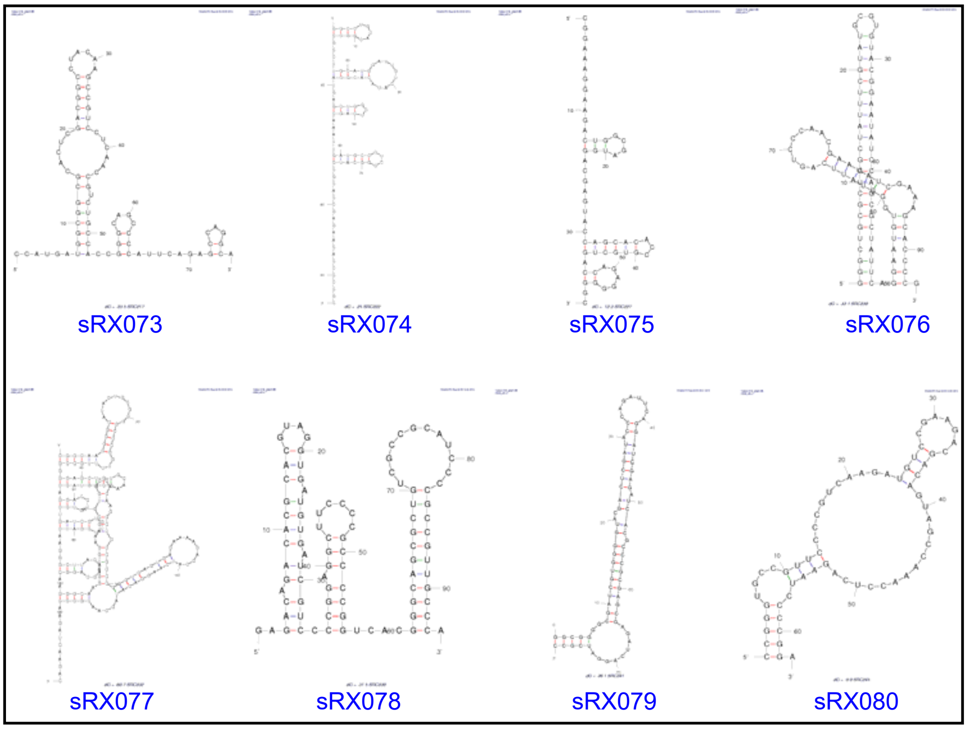
sRX073
sRX074
sRX075
sRX076
sRX077
sRX078
sRX079
sRX080

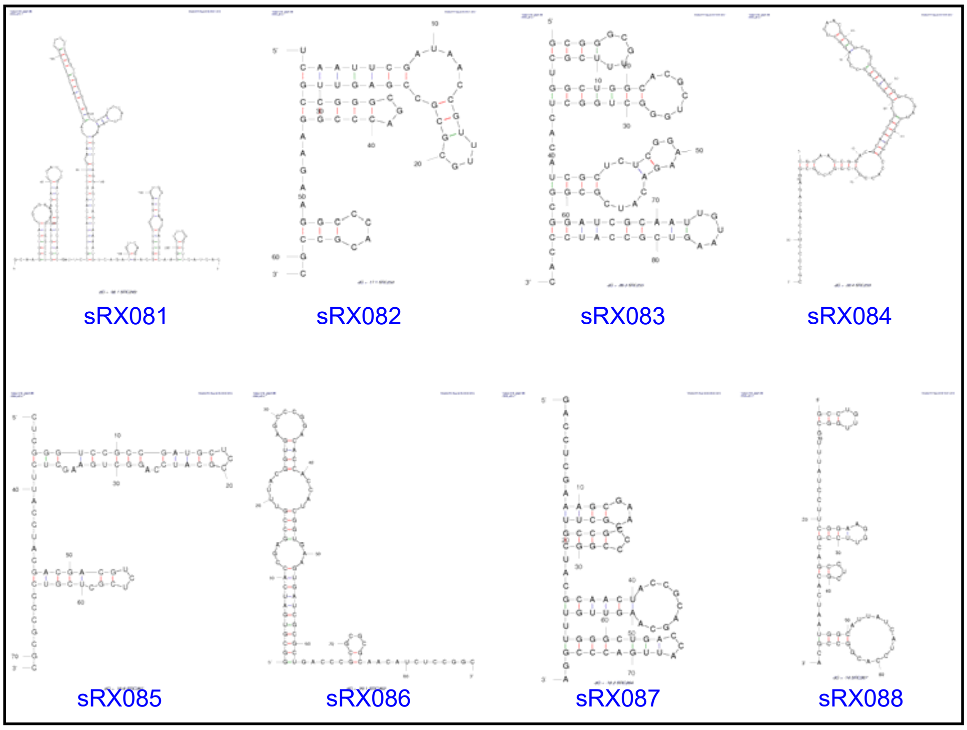
sRX081
sRX082
sRX083
sRX084
sRX085
sRX086
sRX087
sRX088

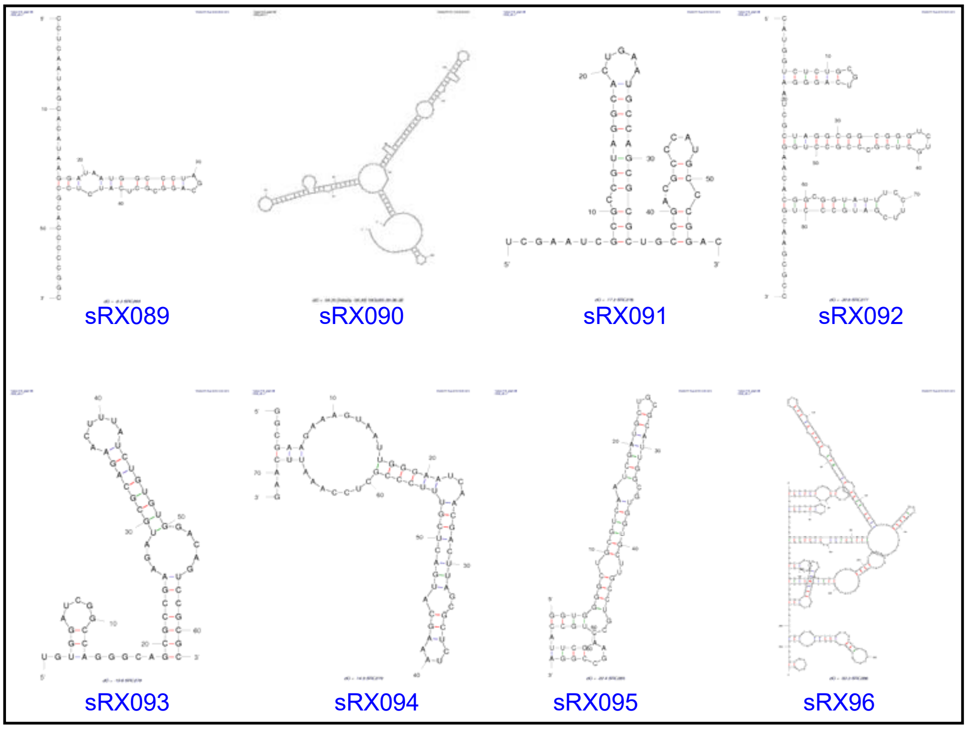
sRX089
sRX090
sRX091
sRX092
sRX093
sRX094
sRX095
sRX96

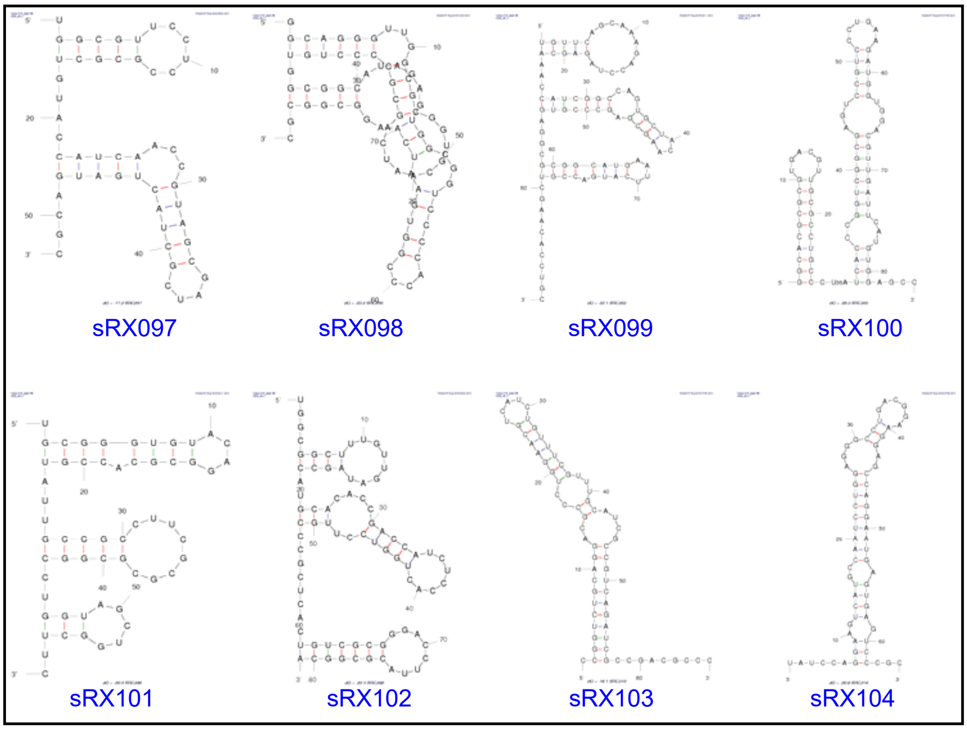
sRX097
sRX098
sRX099
sRX100
sRX101
sRX102
sRX103
sRX104

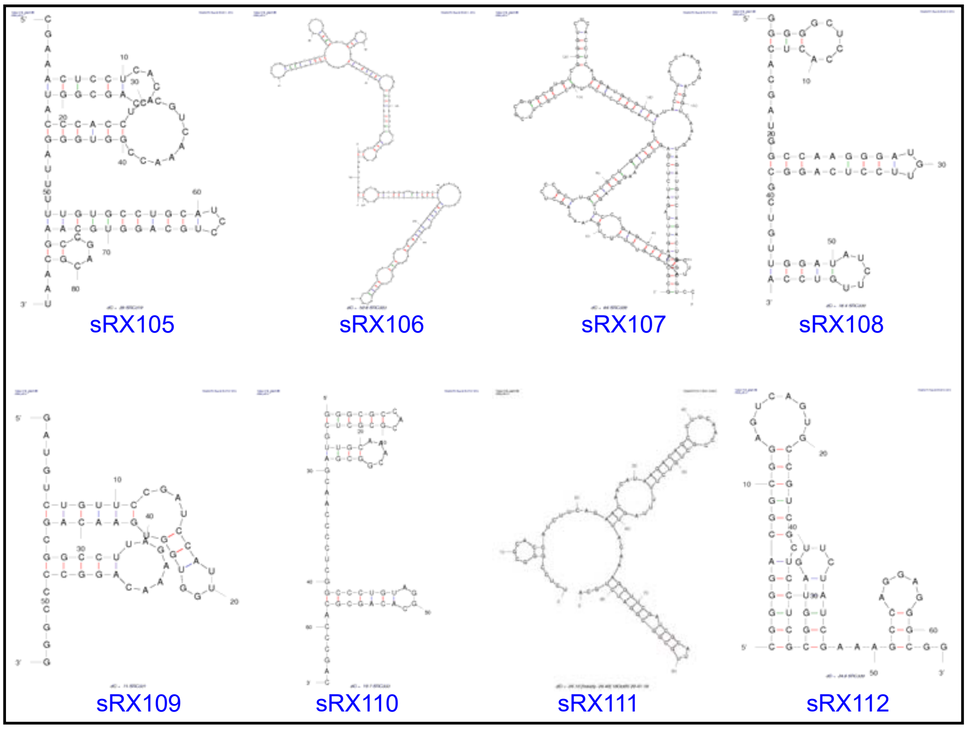
sRX105
sRX106
sRX107
sRX108
sRX109
sRX110
sRX111
sRX112

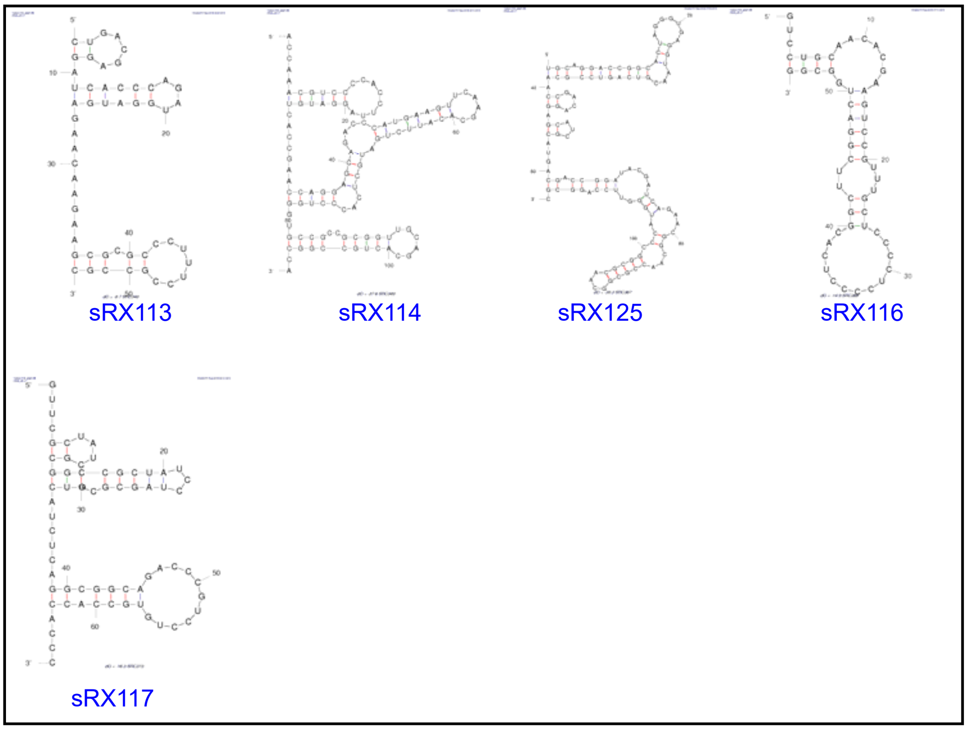
sRX113
sRX114
sRX125
sRX116
sRX117
