# Supplementary material for: Genome‐wide screen and functional analysis in Xanthomonas reveal a large number of mRNA‐derived sRNAs, including the novel RsmA‐sequester RsmU
Source: Mol Plant Pathol. 2020 Sep 23;21(12):1573–90. doi: 10.1111/mpp.12997 (PMC7694677; doi:10.1111/mpp.12997)
Supplement: Supplementary file 5 — FIGURE S5 Detection of the expression level of target sRNA in the pBBad‐carrying wild‐type strain (WT/pB) and its overexpression strain (OE‐SR) by semiquantitative RT‐PCR. Xcc cells were cultured in the minimal medium MMX to mid‐log phase. Total RNAs from the cells were isolated and contaminated DNA was removed. cDNAs were synthesized from the RNAs by reverse transcription and used as template for PCR amplification using the sRNA gene‐specific primers. The PCR was performed with a cycler using the following cycle parameters: 30 cycles of 94 °C for 15 s, 60 °C for 15 s, and 72 °C for 15 s. The amplification products were analysed in 1.2% agarose gels and signal bands were quantified using GelQuant.NET software provided by biochemlabsolutions.com. Values under each gel image are the relative signal density of the corresponding PCR product. The 16S rRNA gene was used as the internal control to verify absence of significant variation at cDNA level in the two RNA samples [file MPP-21-1573-s005.pdf]

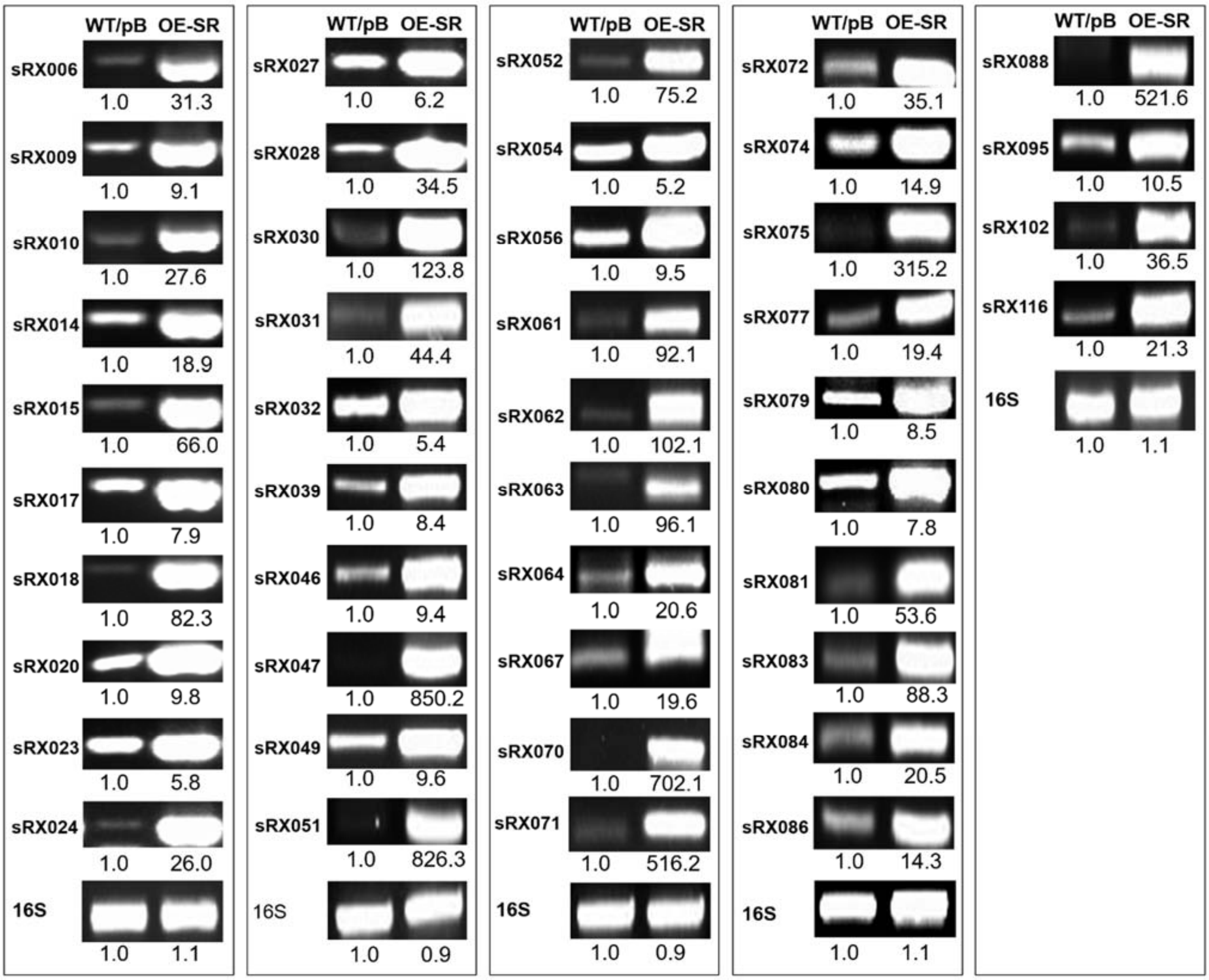

Fig. S5. Detection of the expression level of target sRNA in the pBBad-carrying wild-type strain (WT/pB) and its over-expression strain (OE-SR) by semi-quantitative RT-PCR. *Xcc* cells were cultured in the minimal medium MMX to mid-log phase. Total RNAs from the cells were isolated and contaminated DNA was removed. cDNAs were synthesized from the RNAs by reverse transcription and used as template for PCR amplification using the sRNA gene-specific primers. The PCR was performed with a cycler using the following cycle parameters: 30 cycles of 94 °C for 15 s, 60 °C for 15 s, and 72 °C for 15 s. The amplification products were analyzed in 1.2% agarose gels and signal bands were quantified using GelQuant.NET software provided by biochemlabsolutions.com. Values under each gel image are the relative signal density of the corresponding PCR product. The 16S rRNA gene was used as the internal control to verify absence of significant variation at cDNA level in the two RNA samples.
