# Supplementary material for: Genome‐wide screen and functional analysis in Xanthomonas reveal a large number of mRNA‐derived sRNAs, including the novel RsmA‐sequester RsmU
Source: Mol Plant Pathol. 2020 Sep 23;21(12):1573–90. doi: 10.1111/mpp.12997 (PMC7694677; doi:10.1111/mpp.12997)
Supplement: Supplementary file 6 — FIGURE S6 Comparison of the virulence of the sRNA overexpression strains and the wild‐type strain. The virulence of the Xcc strains was tested in the leaves of Chinese radish (Raphanus sativus var. radiculus) using the leaf‐clipping method (An et al., 2017). Xcc strains were grown in NYG medium at 28 °С with shaking at 200 rpm for 15 hr. Cell concentration was adjusted to OD600 = 0.001. Two to three fully expanded leaves per plant were inoculated by leaf clipping: the leaves were cut with scissors dipped in the bacterial suspensions. Lesion length was measured 10 days postinoculation. Fifty leaves were inoculated for each strain in each independent experiment. The experiment was repeated three times. Data are the mean ± SD from a representative experiment. Similar results were obtained in two other independent experiments. The asterisks above the column represent the significant difference (p = .01 by t test) versus the wild‐type strain [file MPP-21-1573-s006.pdf]

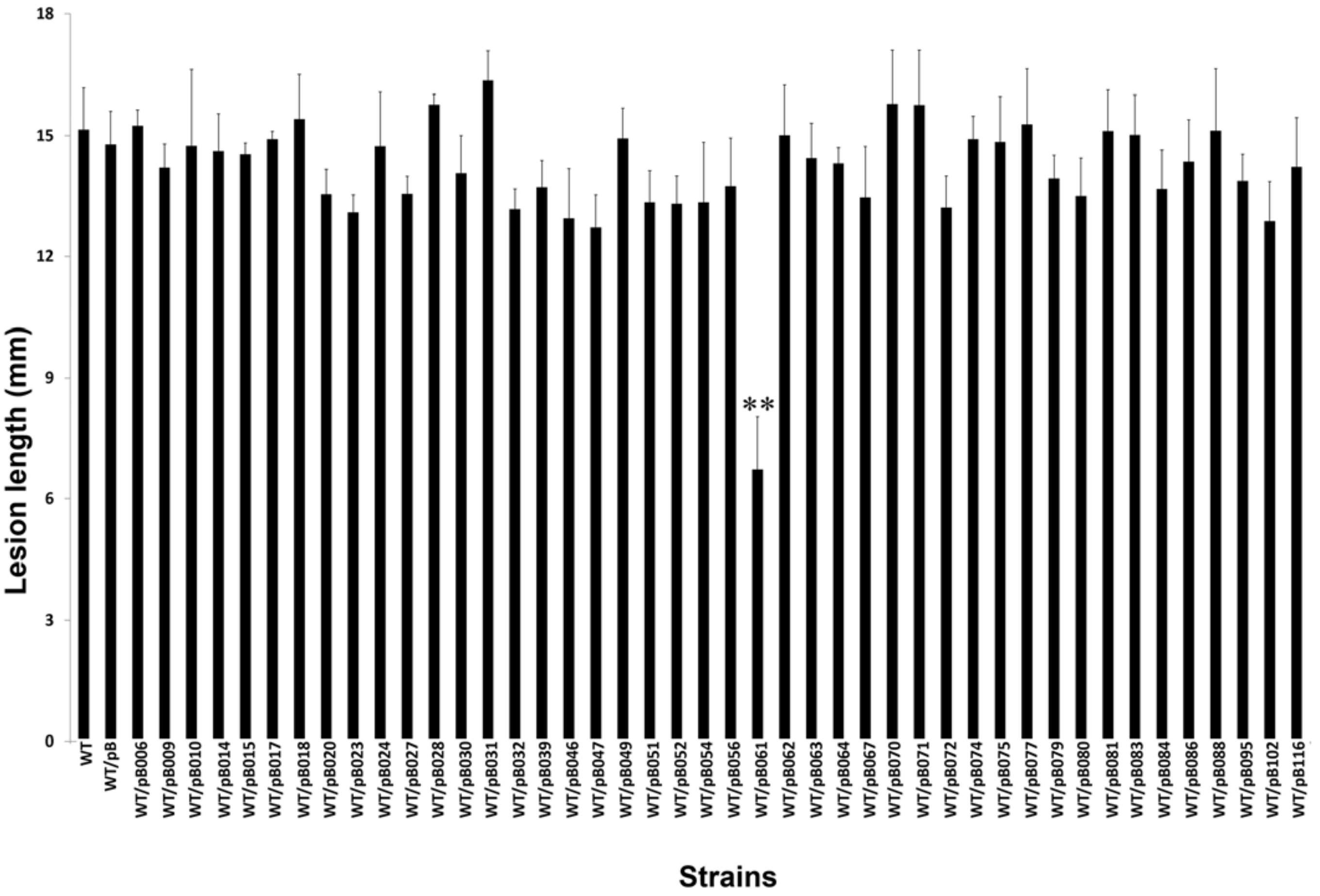

Fig. S6. Comparison of the virulence of the sRNA over-expression strains and the wild-type strain. The virulence of *Xcc* strains was tested in the leaves of Chinese radish (*Raphanus sativus* L. var. radiculus Pers.) using the leaf-clipping method (An *et al*., 2017). *Xcc* strains were grown in NYG medium at 28 °C with shaking at 200 rpm for 15 h. Cell concentration was adjusted to OD_{600}=0.001. Two to three full-expanded leaves per plant were inoculated by leaf clipping: the leaves were cut with scissors dipped in the bacterial suspensions. Lesion length was measured 10 days post-inoculation. Fifty leaves were inoculated for each strain in each independent experiment. The experiment was repeated three times. Data are the mean ± SD from a representative experiment. Similar results were obtained in two other independent experiments. The asterisks above the column represent the significant difference (P=0.01 by t-test) versus the wild-type strain.

REFERENCES

An, S.Q., Tang, J.L. and Dow, J.M. (2017) Probing the role of cyclic di-GMP signaling systems in disease using Chinese radish. *Methods in Molecular Biology*, 1657, 205-212.
