# Supplementary material for: Genome‐wide screen and functional analysis in Xanthomonas reveal a large number of mRNA‐derived sRNAs, including the novel RsmA‐sequester RsmU
Source: Mol Plant Pathol. 2020 Sep 23;21(12):1573–90. doi: 10.1111/mpp.12997 (PMC7694677; doi:10.1111/mpp.12997)
Supplement: Supplementary file 7 — FIGURE S7 Comparison of the growth of Xcc strains in the minimal medium MMX and the rich medium NYG. The pBBad‐carrying wild‐type strain WT/pB and the sRX061 overexpression strain WT/pB061 were separately inoculated in 5 ml of NYG medium and incubated overnight at 28 °С in a shaking incubator at 200 rpm. The cells were washed with water and resuspended to a concentration of OD600 = 1.0, and then 2 μl of the resuspension for each strain was spotted onto NYG and MMX plates, respectively, and incubated at 28 °С. Results were observed at 48 hr (for NYG plate) or 96 hr (for MMX plate) of incubation. Each experiment was repeated three times and similar results were obtained [file MPP-21-1573-s007.pdf]

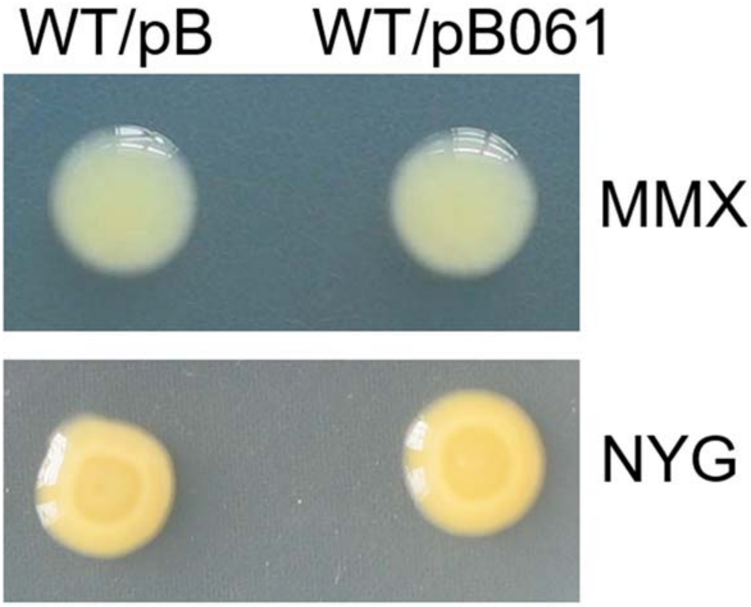

Fig. S7. Comparison of the growth of *Xcc* strains in the minimal medium MMX and the rich medium NYG. The pBBad-carrying wild-type strain WT/pB and the sRX061 over-expression strain WT/pB061 were separately inoculated in 5 ml of NYG medium and incubated overnight at 28 °C in a shaking incubator at 200 rpm. The cells were washed with water and resuspended to a concentration of OD_{600} = 1.0, and then 2 μl of the resuspension for each strain was respectively spotted onto NYG and MMX plates and incubated at 28 °C. Results were observed at 48 h (for NYG plate) or 96 h (for MMX plate) post-incubation. Each experiment was repeated three times and similar results were obtained.
