# Supplementary material for: Genome‐wide screen and functional analysis in Xanthomonas reveal a large number of mRNA‐derived sRNAs, including the novel RsmA‐sequester RsmU
Source: Mol Plant Pathol. 2020 Sep 23;21(12):1573–90. doi: 10.1111/mpp.12997 (PMC7694677; doi:10.1111/mpp.12997)
Supplement: Supplementary file 8 — FIGURE S8 Detection of sRX061S and sRX061L levels in the Xcc wild‐type strain 8,004 (WT), WT carrying an empty pBBad (WT/pB), WT carrying a pBBad containing sRX061S‐coding sequence (WT/pB061S) or sRX061L‐coding sequence (WT/pB061L). The strains were cultured in the minimal medium MMX at 28 °C with shaking at 200 rpm for 24 hr. Total RNAs were isolated and 3 μg of the total RNAs were separated by 6% denaturing (8 M urea) polyacrylamide gel electrophoresis and transferred to a positively charged nylon membrane. After UV‐crosslinking, the membrane was hybridized with the DIG‐labelled sR061 probe. The hybridization was performed at 68 °C for 8 hr. Signal bands were detected using a DIG‐Northern Starter Kit, visualized with an ImageQuant LAS 500 imager, and quantified using GelQuant.NET software provided by biochemlabsolutions.com. 5S rRNA was probed as a loading control [file MPP-21-1573-s008.pdf]

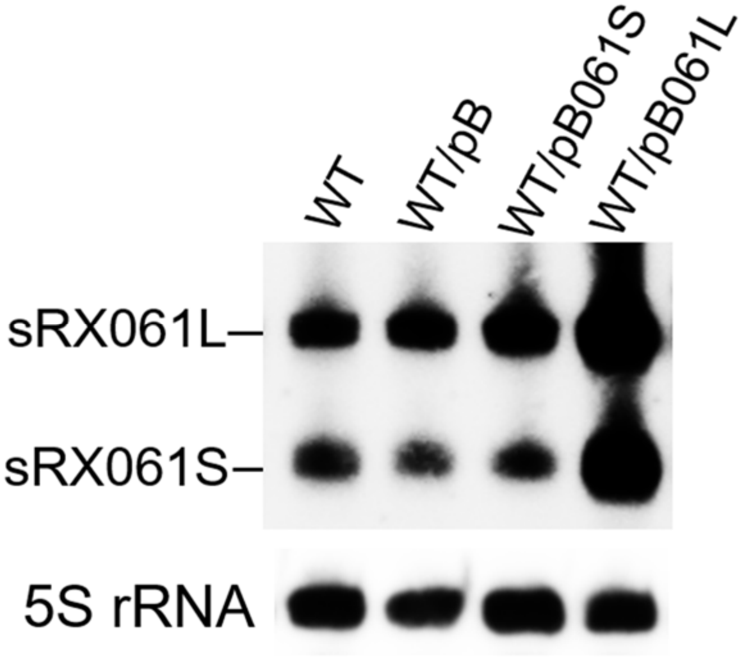

Fig. S8. Detection of sRX061S and sRX061L levels in the *Xcc* wild-type strain 8004 (WT), WT carrying an empty pBBad (WT/pB), WT carrying a pBBad containing sRX061S-coding sequence (WT/pB061S) or sRX061L-coding sequence (WT/pB061L). The strains were cultured in the minimal medium MMX at 28 °C with shaking at 200 rpm for 24 h. Total RNAs were isolated and 3 μg of the total RNAs were separated by 6% denature (8M urea) polyacrylamide gel electrophoresis and transferred to a positively charged nylon membrane. After UV-crosslinking, the membrane was hybridized with the DIG-labelled sR061 probe. The hybridization was performed at 68 °C for 8 h. Signal bands were detected by using a DIG-Northern Starter Kit, visualized with an ImageQuant LAS 500 imager and quantified using GelQuant.NET software provided by biochemlabsolutions.com. 5S rRNA was probed as a loading control.
