# Supplementary material for: Genome‐wide screen and functional analysis in Xanthomonas reveal a large number of mRNA‐derived sRNAs, including the novel RsmA‐sequester RsmU
Source: Mol Plant Pathol. 2020 Sep 23;21(12):1573–90. doi: 10.1111/mpp.12997 (PMC7694677; doi:10.1111/mpp.12997)
Supplement: Supplementary file 9 — FIGURE S9 Identification of target transcripts (TTs) based on the visualized mapping pattern. The two representative graphical diagrams show the mapping details of the RNA‐Seq reads obtained from the 50–500 nt RNA libraries in the genome of Xcc strain 8,004. (a) A typical graphical diagram of a mapped region containing a TT. (b) A typical graphical diagram of a mapped region without a significant TT. The vertical ordinate represents the number of mapped reads and the horizontal ordinate indicates the genome position and genetic organization of the mapped region [file MPP-21-1573-s009.pdf]

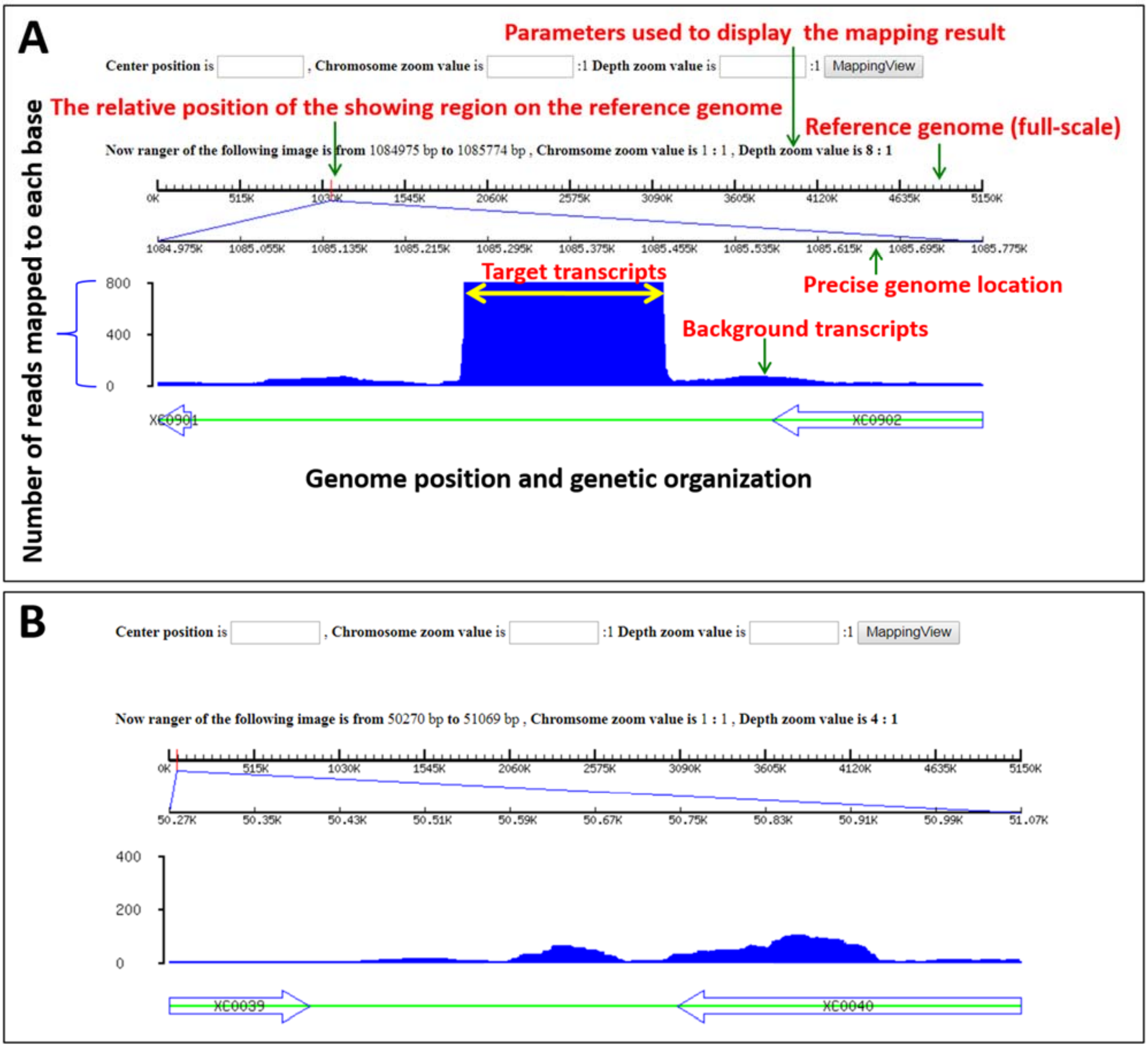

Fig. S9. Identification of target transcripts (TTs) based on the visualized mapping pattern. The figure consists of two representative graphical diagrams showing the mapping details of the RNA-seq reads obtained from the 50-500-nt RNA libraries in the genome of *Xcc* strain 8004. **(A)** A typical graphical diagram of a mapped region containing a TT. **(B)** A typical graphical diagram of a mapped region without a significant TT. The vertical ordinate represents the number of mapped reads and the horizontal ordinate indicates genome position and genetic organization of the mapped region.
