# Supplementary material for: Genome‐wide screen and functional analysis in Xanthomonas reveal a large number of mRNA‐derived sRNAs, including the novel RsmA‐sequester RsmU
Source: Mol Plant Pathol. 2020 Sep 23;21(12):1573–90. doi: 10.1111/mpp.12997 (PMC7694677; doi:10.1111/mpp.12997)
Supplement: Supplementary file 10 — FIGURE S10 The effect of XC1332 3′‐UTR deletion on its mRNA accumulation. (a) The genetic organization of the XC1332 3′‐UTR deletion strain. (b) Detection of the XC1332 mRNA level in wild‐type strain (WT) and the XC1332 3′‐UTR deletion strain Δ3′UTR by northern blotting using the P1332+061 probe. Strains were cultured in NYG medium at 28 °C with shaking at 200 rpm for 24 hr. Total RNAs were isolated and 3 μg was used for northern blotting with a DIG‐labelled RNA probe. The positions of the P1332+061 probe are shown as a thick red line in (a). 5S rRNA was probed as a loading control [file MPP-21-1573-s010.pdf]

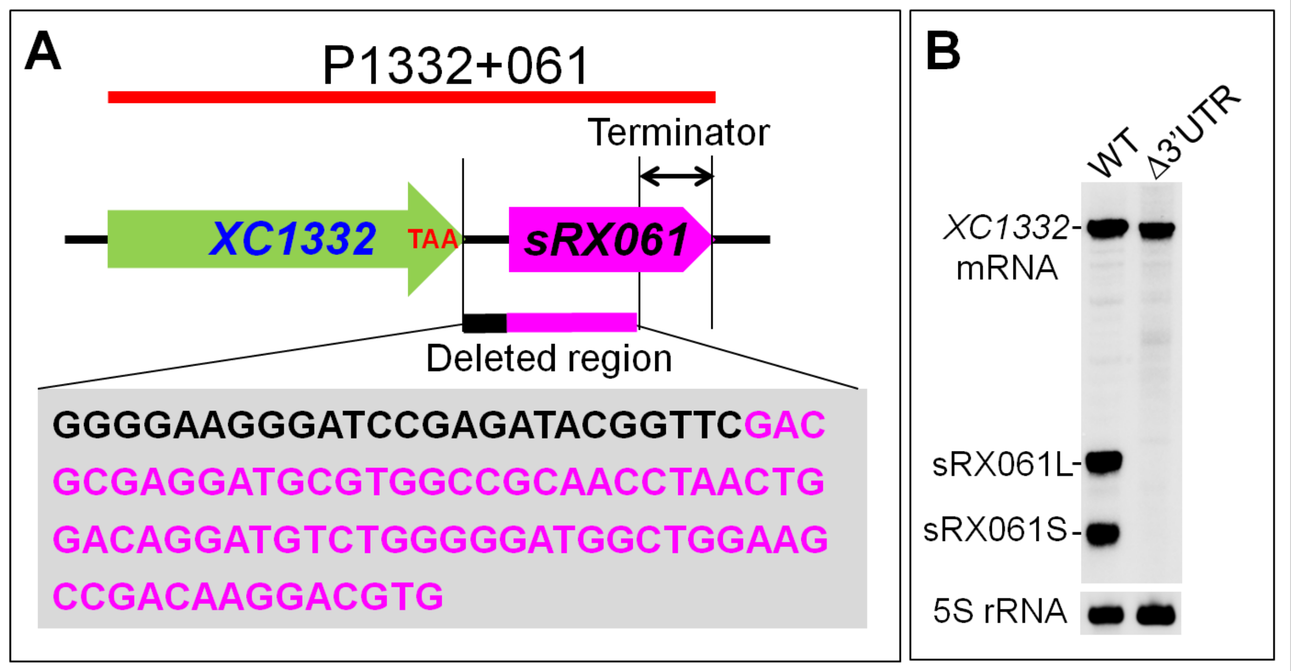

Fig. S10. The effect of *XC1332* 3'UTR deletion on its mRNA accumulation. (A) The genetic organization of the *XC1332* 3'UTR deletion strain. **(B)** Detection of *XC1332* mRNA level in wild-type strain (WT) and the *XC1332* 3'UTR deletion strain Δ3'UTR by Northern blotting using P1332+061 probe. *S*trains were cultured in NYG medium at 28 °C with shaking at 200 rpm for 24 h. Total RNAs were isolated and 3 μg of them were used for Northern blotting with DIG-labelled RNA probe. The positions of P1332+061 probe are shown as a thick red line in panel A. 5S rRNA was probed as a loading control.
