# Supplementary material for: Genome‐wide screen and functional analysis in Xanthomonas reveal a large number of mRNA‐derived sRNAs, including the novel RsmA‐sequester RsmU
Source: Mol Plant Pathol. 2020 Sep 23;21(12):1573–90. doi: 10.1111/mpp.12997 (PMC7694677; doi:10.1111/mpp.12997)
Supplement: Supplementary file 11 — FIGURE S11 Phenotype analysis of ΔP1332 and CΔP1332 strains. (a) Black rot symptoms and the average lesion lengths (in mm) caused by wild‐type strain (WT), ΔP1332 and CΔP1332 on inoculated leaves of Chinese radish. Images were taken at 10 days postinoculation. Values under each leaf are the average lesion lengths (mean ± SD) from three repeats, each with 50 leaves. The same letter after the values of the average lesion lengths indicates no significant difference (t test, p < .01). (b) The hypersensitive response (HR) induced on nonhost plant pepper leaves (Capsicum annuum “ECW‐10R”) by Xcc strains. The photograph was taken 24 hr after inoculation. The result presented is from a representative experiment and similar results were obtained in two other independent experiments. The type III‐deficient mutant strain (ΔhrcV) was used as a negative control. (c) The result of a swarming motility assay. Two microlitres of overnight culture (OD600 = 1.0) of each strain was spotted onto the swarming assay plate, and results were observed and photographed after incubation at 28 °C for 3 days [file MPP-21-1573-s011.pdf]

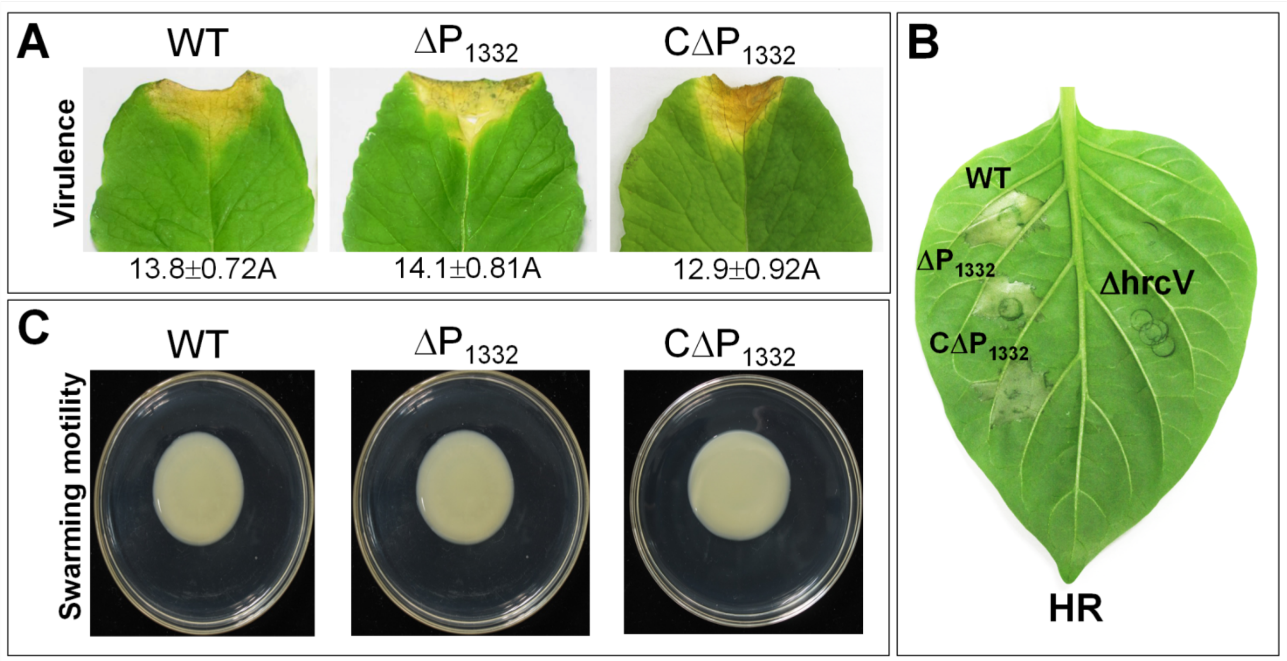

Fig. S11. Phenotype analysis of ΔP_{1332} and CΔP_{1332} strains. (A) Black rot symptoms and the average lesion lengths (in mm) caused by wild-type strain (WT), ΔP_{1332} and CΔP_{1332} on inoculated leaves of Chinese radish. Images were taken at day 10 post-inoculation. Values under each leaf are the average lesion lengths (mean ± SD) from three repeats, each with 50 leaves. The same letter after the values of the average lesion lengths indicate no significant difference (*t*-test, $P < 0.01$). **(B)** The HR induced on non-host plant pepper leave (*Capsicumannuum* cv. ECW-10R) by *Xcc* strains. The photograph was taken 24 h after inoculation. The result presented is from a representative experiment and similar results were obtained in other two independent experiments. The type III-deficient mutant strain (ΔhrcV) was used as a negative control. **(C)** The result of swarming motility assay. 2μl overnight culture (OD_{600} = 1.0) of each strain was respectively spotted onto the swarming assay plate, and results were observed and photographed after incubation at 28 °C for 3 days.
