# Supplementary material for: Genome‐wide screen and functional analysis in Xanthomonas reveal a large number of mRNA‐derived sRNAs, including the novel RsmA‐sequester RsmU
Source: Mol Plant Pathol. 2020 Sep 23;21(12):1573–90. doi: 10.1111/mpp.12997 (PMC7694677; doi:10.1111/mpp.12997)
Supplement: Supplementary file 12 — FIGURE S12 The effect of rsmA deletion on the accumulation of sRX061 and XC1332 mRNA. (a) The genetic organization of sRX061 locus and the specific probes used in this northern blotting. (b) Detection of the sRX061 and XC1332 mRNA transcripts in wild‐type strain (WT) and rsmA deletion mutant (ΔrsmA) by northern blotting using a probe specific for the coding region of XC1332 (P1332) and the sRX061‐specific probe (P061). Strains were cultured in NYG medium at 28 °C with shaking at 200 rpm for 24 hr. Total RNAs were isolated and 3 μg of these was used for northern blotting with a DIG‐labelled RNA probe. The positions of P1332 and P061 are shown as a thick red line in (a). 5S rRNA was probed as a loading control [file MPP-21-1573-s012.pdf]

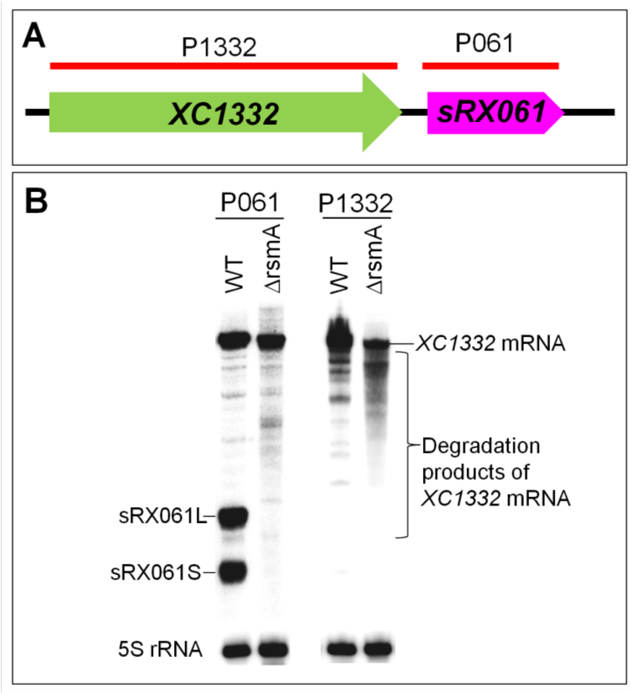

Fig. S12. The effect of *rsmA* deletion on the accumulation of sRX061 and *XC1332* mRNA. (A) The genetic organization of *sRX061* locus and the specific probes used in this Northern blotting. **(B)** Detection of the sRX061 and *XC1332* mRNA transcripts in wild-type strain (WT) and *rsmA* deletion mutant (ΔrsmA) by Northern blotting using probe specific for the coding region of *XC1332* (P1332) and the sRX061-specific probe (P061). *S*trains were cultured in NYG medium at 28 °C with shaking at 200 rpm for 24 h. Total RNAs were isolated and 3 μg of them were used for Northern blotting with DIG-labelled RNA probe. The positions of P1332 and P061 are shown as a thick red line in panel A. 5S rRNA was probed as a loading control.
