# Supplementary material for: Genome‐wide screen and functional analysis in Xanthomonas reveal a large number of mRNA‐derived sRNAs, including the novel RsmA‐sequester RsmU
Source: Mol Plant Pathol. 2020 Sep 23;21(12):1573–90. doi: 10.1111/mpp.12997 (PMC7694677; doi:10.1111/mpp.12997)
Supplement: Supplementary file 13 — TABLE S1 Bacterial strains and plasmids used in this work [file MPP-21-1573-s013.pdf]

Table S1. Bacterial strains and plasmids used in this work

Strains and plasmids	Relevant characteristics*	Source or reference
***E. coli* strains**		
ED8767	*RecA*56, *metB, hsdS, supEsupF,* containing pRK2073, Spc^r	Our Lab's collection
DH5α	F-*recA*1Φ80d *lacZ* ⊿M15	Our Lab's collection
BL21(DE3)	F –*ompT hsdS$_B$* (r_B –m_B –) *gal dcm* (DE3) pLysS	Novagen, Germany
JM109	*RecA*1, *endA*1, *gyrA*96, *thi*, *supE*44, *relA*1⊿ (*lac-proAB*)/F' [*traD*36, *lacI*^{q}, *lacZ*⊿M15]	Yanisch-Perron *et al.*, 1985
BL2506	BL21(DE3) containing pET2506, Kan^r	Our Lab's collection
***Xcc* strains**		
8004	Wild-type strain, Rif^r	Daniels *et al.*, 1984
ΔP$_{1332}$	As 8004, but the promoter region of *XC1332* has been deleted, Rif^r	This study
Δ3'UTR	As 8004, but the 3'UTR of *XC1332* has been deleted, Rif^r	This study
CΔP$_{1332}$	ΔP$_{1332}$ carrying the plasmid pL1332	This study
ΔhrcV	As 8004, but the coding region of *hrcV* (*XC3013*) has been deleted, Rif^r	Our Lab's collection
ΔrsmA	As 8004, but the coding region of *rsmA* (*XC2506*) has been deleted, Rif^r	Our Lab's collection
Hfq3F	Also known as 8004Hfq3F. As 8004, but the 3'-end of *hfq* coding region with an in-frame insertion of 3xFLAG-coding sequence, Rif^r	Lai *et al.*, 2018
RsmA3F	As 8004, but the 3'-end of *rsmA* coding region with an in-frame insertion of 3xFLAG-coding sequence, Rif^r	Our Lab's collection
RsmA3F/pB	RsmA3F containing PBBad, Rif^r, Kan^r	This study
RsmA3F/ PBsRX061L	RsmA3F containing PBsRX061L, Rif^r, Kan^r	This study
WT/pB	8004 containing pBBad, Rif^r, Kan^r	Our Lab's collection
WT/pB006	8004 containing pBsRX006, Rif^r, Kan^r	This study
WT/pB009	8004 containing pBsRX009, Rif^r, Kan^r	This study
WT/pB010	8004 containing pBsRX010, Rif^r, Kan^r	This study
WT/pB014	8004 containing pBsRX014, Rif^r, Kan^r	This study
WT/pB015	8004 containing pBsRX015, Rif^r, Kan^r	This study
WT/pB017	8004 containing pBsRX017, Rif^r, Kan^r	This study
WT/pB018	8004 containing pBsRX018, Rif^r, Kan^r	This study
WT/pB020	8004 containing pBsRX020, Rif^r, Kan^r	This study
WT/pB023	8004 containing pBsRX023, Rif^r, Kan^r	This study
WT/pB024	8004 containing pBsRX024, Rif^r, Kan^r	This study

WT/pB027	8004 containing pBsRX027, Rif^r, Kan^r	This study
WT/pB028	8004 containing pBsRX028, Rif^r, Kan^r	This study
WT/pB030	8004 containing pBsRX030, Rif^r, Kan^r	This study
WT/pB031	8004 containing pBsRX031, Rif^r, Kan^r	This study
WT/pB032	8004 containing pBsRX032, Rif^r, Kan^r	This study
WT/pB039	8004 containing pBsRX039, Rif^r, Kan^r	This study
WT/pB046	8004 containing pBsRX046, Rif^r, Kan^r	This study
WT/pB047	8004 containing pBsRX047, Rif^r, Kan^r	This study
WT/pB049	8004 containing pBsRX049, Rif^r, Kan^r	This study
WT/pB051	8004 containing pBsRX051, Rif^r, Kan^r	This study
WT/pB052	8004 containing pBsRX052, Rif^r, Kan^r	This study
WT/pB054	8004 containing pBsRX054, Rif^r, Kan^r	This study
WT/pB056	8004 containing pBsRX056, Rif^r, Kan^r	This study
WT/pB061	8004 containing pBsRX061, Rif^r, Kan^r	This study
WT/pB061L	8004 containing pBsRX061L, Rif^r, Kan^r	This study
WT/pB061S	8004 containing pBsRX061S, Rif^r, Kan^r	This study
WT/pB061LM	8004 containing pBsRX061LM, Rif^r, Kan^r	This study
WT/pB062	8004 containing pBsRX062, Rif^r, Kan^r	This study
WT/pB063	8004 containing pBsRX063, Rif^r, Kan^r	This study
WT/pB064	8004 containing pBsRX064, Rif^r, Kan^r	This study
WT/pB067	8004 containing pBsRX067, Rif^r, Kan^r	This study
WT/pB070	8004 containing pBsRX070, Rif^r, Kan^r	This study
WT/pB071	8004 containing pBsRX071, Rif^r, Kan^r	This study
WT/pB072	8004 containing pBsRX072, Rif^r, Kan^r	This study
WT/pB074	8004 containing pBsRX074, Rif^r, Kan^r	This study
WT/pB075	8004 containing pBsRX075, Rif^r, Kan^r	This study
WT/pB077	8004 containing pBsRX077, Rif^r, Kan^r	This study
WT/pB079	8004 containing pBsRX079, Rif^r, Kan^r	This study
WT/pB080	8004 containing pBsRX080, Rif^r, Kan^r	This study
WT/pB081	8004 containing pBsRX081, Rif^r, Kan^r	This study

WT/pB083	8004 containing pBsRX083, Rif^r, Kan^r	This study
WT/pB084	8004 containing pBsRX084, Rif^r, Kan^r	This study
WT/pB086	8004 containing pBsRX086, Rif^r, Kan^r	This study
WT/pB088	8004 containing pBsRX088, Rif^r, Kan^r	This study
WT/pB095	8004 containing pBsRX095, Rif^r, Kan^r	This study
WT/pB102	8004 containing pBsRX102, Rif^r, Kan^r	This study
WT/pB116	8004 containing pBsRX116, Rif^r, Kan^r	This study
**Plasmids**		
pRK2073	Helper plasmid, Tra+,Mob+,ColE1,Spc^r	Leong *et al.*, 1982
pLAFR6	Broad host range cloning vector, Tc^r	Huynh *et al.*, 1989
pK18mobsacB	The suicide plasmid pK18mob containing a *sacB* gene, Kan^r	Our Lab's collection
pET-30a-c(+)	Expression vector, allowing the production of fusion protein containing amino terminal 6xHis-tagged sequences, Kan^r	Novagen, Germany
pET2506	pET-30a-c(+) containing the coding sequence of $rsmA_{Xcc}$, Kan^r	Our Lab's collection
pBBad	Arabinose-inducible broad host range expression vector, Kan^r	Sukchawalit *et al.*, 1999
pBsRX006	pBBad containing sRX006 gene, Kan^r	This study
pBsRX009	pBBad containing sRX009 gene, Kan^r	This study
pBsRX010	pBBad containing sRX010 gene, Kan^r	This study
pBsRX014	pBBad containing sRX014 gene, Kan^r	This study
pBsRX015	pBBad containing sRX015 gene, Kan^r	This study
pBsRX017	pBBad containing sRX017 gene, Kan^r	This study
pBsRX018	pBBad containing sRX018 gene, Kan^r	This study
pBsRX020	pBBad containing sRX020 gene, Kan^r	This study
pBsRX023	pBBad containing sRX023 gene, Kan^r	This study
pBsRX024	pBBad containing sRX024 gene, Kan^r	This study
pBsRX027	pBBad containing sRX027 gene, Kan^r	This study
pBsRX028	pBBad containing sRX028 gene, Kan^r	This study
pBsRX030	pBBad containing sRX030 gene, Kan^r	This study
pBsRX031	pBBad containing sRX031 gene, Kan^r	This study
pBsRX032	pBBad containing sRX032 gene, Kan^r	This study
pBsRX039	pBBad containing sRX039 gene, Kan^r	This study

pBsRX046	pBBad containing sRX046 gene, Kan^r	This study
pBsRX047	pBBad containing sRX047 gene, Kan^r	This study
pBsRX049	pBBad containing sRX049 gene, Kan^r	This study
pBsRX051	pBBad containing sRX051 gene, Kan^r	This study
pBsRX052	pBBad containing sRX052 gene, Kan^r	This study
pBsRX054	pBBad containing sRX054 gene, Kan^r	This study
pBsRX056	pBBad containing sRX056 gene, Kan^r	This study
pBsRX061	pBBad containing sRX061 gene, Kan^r	This study
pBsRX061L	pBBad containing the sRX061 isoform sRX061L, Kan^r	This study
pBsRX061LM	pBBad containing the mutated (GGA→AAA) sRX061L , Kan^r	This study
pBsRX061S	pBBad containing the sRX061 isoform sRX061S, Kan^r	This study
pBsRX062	pBBad containing sRX062 gene, Kan^r	This study
pBsRX063	pBBad containing sRX063 gene, Kan^r	This study
pBsRX064	pBBad containing sRX064 gene, Kan^r	This study
pBsRX067	pBBad containing sRX067 gene, Kan^r	This study
pBsRX070	pBBad containing sRX070 gene, Kan^r	This study
pBsRX071	pBBad containing sRX071 gene, Kan^r	This study
pBsRX072	pBBad containing sRX072 gene, Kan^r	This study
pBsRX074	pBBad containing sRX074 gene, Kan^r	This study
pBsRX075	pBBad containing sRX075 gene, Kan^r	This study
pBsRX077	pBBad containing sRX077 gene, Kan^r	This study
pBsRX079	pBBad containing sRX079 gene, Kan^r	This study
pBsRX080	pBBad containing sRX080 gene, Kan^r	This study
pBsRX081	pBBad containing sRX081 gene, Kan^r	This study
pBsRX083	pBBad containing sRX083 gene, Kan^r	This study
pBsRX084	pBBad containing sRX084 gene, Kan^r	This study
pBsRX086	pBBad containing sRX086 gene, Kan^r	This study
pBsRX088	pBBad containing sRX088 gene, Kan^r	This study
pBsRX095	pBBad containing sRX095 gene, Kan^r	This study
pBsRX102	pBBad containing sRX102 gene, Kan^r	This study

pBsRX116	pBBad containing sRX116 gene, Kan^r	This study
pKP1332UD	pK18mobsacB containing the ~1.3 kb upstream and downstream flanking sequences of *XC1332* promoter, Kan^r	This study
pK3'UTRUD	pK18mobsacB containing the ~1.5 kb upstream and downstream flanking sequences of the 3'UTR of *XC1332*, Kan^r	This study
pL1332	pLAFR6 containing the entire *XC1332* gene, Tc^r	This study

*Rif^r, rifampicin-resistant; Kan^r, kanamycin-resistant; Spc^r, spectinomycin-resistant; Tc^r, tetracycline-resistant.

REFERENCE

Daniels, M.J., Barber, C.E., Turner, P.C., Sawczyc, M.K., Byrde, R.J.W. and Fielding, A.H. (1984) Cloning of genes involved in pathogenicity of *Xanthomonas campestris* pv. *campestris* using the broad-host-range cosmid pLAFR1. *EMBO Journal*, 3, 3323-3328.

Huynh, T.V., Dahlbeck, D, and Staskawicz, B.J. (1989) Bacterial blight of soybean: regulation of a pathogen gene determining host cultivar specificity. *Science*, 245, 1374-1377.

Lai, J.-L. Tang, D.-J. Liang, Y.-W. Zhang, R. Chen, Q. Qin, Z.-P. Ming, Z.-H. and Tang J.-L. (2018) The RNA chaperone Hfq is important for the virulence, motility and stress tolerance in the phytopathogen *Xanthomonas campestris*. *Environmental Microbiology Reports*, 10(5), 542-554.

Leong, S.A., Ditta, G.S. and Helinski, D.R. (1982) Heme biosynthesis in *Rhizobium*. Identification of a cloned gene coding for delta-aminolevulinic acid synthetase from *Rhizobium meliloti*. *The Journal of Biological Chemistry*, 257, 8724-8730.

Sukchawalit, R., Vattanaviboon, P., Sallabhan, R. and Mongkolsuk, S. (1999) Construction and characterization of regulated L-arabinose-inducible broad host range expression vectors in *Xanthomonas*. *FEMS Microbiology Letters*, 181, 217-223.

Yanisch-Perron, C., Vieira, J. and Messing, J. (1985) Improved M13 phage cloning vectors and host strains: nucleotide sequences of the M13mp18 and pUC19 vectors. *Gene*, 33, 103-119.
