# Supplementary material for: Genome‐wide screen and functional analysis in Xanthomonas reveal a large number of mRNA‐derived sRNAs, including the novel RsmA‐sequester RsmU
Source: Mol Plant Pathol. 2020 Sep 23;21(12):1573–90. doi: 10.1111/mpp.12997 (PMC7694677; doi:10.1111/mpp.12997)
Supplement: Supplementary file 14 — TABLE S2 Distribution of the RNA‐Seq reads mapped on the genome of Xcc strain 8,004 [file MPP-21-1573-s014.pdf]

Table S2. Distribution of the RNA-seq reads mapped on the genome of *Xcc* strain 8004[a]

Location	No.	Total length (bp)	Mapped reads	Coverage
genome	1	5,148,708	37,304,364 (100%)	652
ORFs	4,332	4,392,669	6,333,254 (16.9%)	130
IGRs[b]	2,344	742,000	7,350,108 (19.85%)	892
23S rRNA	2	5,766	1,815,389 (4.87%)	28,336
16S rRNA	2	3,078	907,694 (2.43%)	26,541
5S rRNA	2	238	19,530,642 (52.35%)	7,385,536
tRNAs	54	4,218	1,288,951 (3.45%)	27,503
tmRNA	1	397	48,203 (0.13%)	10,928
rnpB RNA	1	342	30,123 (0.008%)	9,727

[a]*Xcc* strain 8004 genome information comes from GenBank data (accession no. CP000050).
[b]Only the space between two annotated protein-coding or structural RNA genes equally to and longer than 50 base pair (bp) be considered as an IGR.
