# Supplementary material for: Genome‐wide screen and functional analysis in Xanthomonas reveal a large number of mRNA‐derived sRNAs, including the novel RsmA‐sequester RsmU
Source: Mol Plant Pathol. 2020 Sep 23;21(12):1573–90. doi: 10.1111/mpp.12997 (PMC7694677; doi:10.1111/mpp.12997)
Supplement: Supplementary file 15 — TABLE S3 A summary of the identified target transcripts (TTs) (676 in total) [file MPP-21-1573-s015.pdf]

Table S3 A summary of the identified target transcripts (TTs) (676 in total)*

TTs name	Start	End	Length (bp)	PRKM	Inside gene	Overlap gene(s)	Upstream gene	Downstream gene	tRNA, mRNA or known sRNA
TT001	1	115	115	148.1	N	XC0001	N	XC0002	
TT002	3605	3701	97	263.6	N	XC0003	XC0002	XC0004	
TT003	8254	8318	65	201	N	XC0006	XC0005	XC0007	
TT004	10489	10568	80	223.4	N	XC0008	XC0007	XC0009	
TT005	11540	11606	67	100.3	XC0009	N	XC0008	XC0010	
TT006	12884	12975	92	221.4	N	XC0011	XC0010	XC0012	
TT007	19739	19801	63	45.7	XC0017	N	XC0016	XC0018	
TT008	20127	20185	59	80.5	XC0017	N	XC0016	XC0018	
TT009	44065	44146	82	212.9	N	XC0032	XC0031	XC0033	
TT010	44407	44484	77	95.1	XC0033	N	XC0032	XC0034	
TT011	73643	73693	51	117.4	N	XC0057	XC0056	XC0058	
TT012	77120	77175	56	69.7	N	N	XC0062	XC0063	
TT013	77385	77474	90	107.1	XC0063	N	XC0062	XC0064	
TT014	79055	79114	60	40.4	N	N	XC0063	XC0064	
TT015	79664	79718	55	75.8	N	N	XC0064	XC0065	
TT016	81165	81218	54	59.7	N	N	XC0065	XC0066	
TT017	83883	83949	67	389.9	N	N	XC0068	XC0069	
TT018	96245	96331	87	112.2	N	XC0082	XC0081	XC0083	
TT019	96345	96430	86	109.3	XC0082	N	XC0081	XC0083	
TT020	113628	113830	203	261.8	N	N	XC0096	XC0097	
TT021	141901	141975	75	59.5	N	XC0118	XC0117	XC0119	
TT022	142449	142507	59	75.2	N	XC0119	XC0118	XC0120	
TT023	146692	146747	56	88.6	N	N	XC0122	XC0123	
TT024	165210	165280	71	61.1	N	XC0136	XC0134	XC0137	
TT025	174860	174916	57	177.8	N	N	XC0140	XC0141	
TT026	214031	214120	90	108.8	N	XC0171	XC0170	XC0172	
TT027	214270	214333	64	224.9	XC0171	N	XC0170	XC0172	
TT028	225413	225476	64	109.8	N	N	XC0179	XC0180	
TT029	230567	230759	193	170.9	N	XC185<br>XC186	XC0184	XC0187	
TT030	239805	239882	78	42.7	N	N	XC0191	XC0192	
TT031	257175	257244	70	126.5	N	XC0207	XC0206	XC0208	
TT032	262750	262802	53	287.8	N	N	XC0214	XC0215	

TT033	262819	263474	655	302.1	XC0215	N	XC0214	XC0216	XC0215 mRNA
TT034	263377	263474	98	1235.9	N	XC0215	XC0214	XC0216	
TT035	278860	279124	264	203.7	XC0230	N	XC0229	XC0231	XC0230 mRNA
TT036	278860	278923	64	639.9	N	XC0230	XC0229	XC0231	
TT037	292444	292545	102	360.4	N	N	XC0241	XC0242	
TT038	318332	318488	157	41.6	N	XC0264	XC0263	XC0265	
TT039	328623	328686	64	1243.9	XC0274	N	XC0273	XC0275	
TT040	330632	330722	90	1594.6	XC0277	N	XC0275	XC0278	
TT041	330757	330846	89	109.31	XC0278	N	XC0275	XC0278	
TT042	337275	337739	464	457.1	XC0284	N	XC0283	XC0285	XC0284 mRNA
TT043	337275	337504	230	711.6	N	XC0284	XC0283	XC0285	
TT044	337655	337739	85	377.7	N	XC0284	XC0283	XC0285	
TT045	346558	346643	86	100.7	XC0292	N	XC0291	XC0293	
TT046	387979	388077	99	393.8	N	XC0328	XC0327	XC0329	
TT047	388414	388470	57	140.9	XC0328	N	XC0327	XC0329	
TT048	391153	391204	52	159.1	N	XC0331	XC0330	XC0332	
TT049	391686	391747	62	74.1	XC0331	N	XC0330	XC0332	
TT050	392000	392058	59	77.1	XC0331	N	XC0330	XC0332	
TT051	395405	395472	68	2288.1	XC0333	N	XC0332	XC0334	
TT052	410078	410152	74	17009.9	N	N	XC0350	XC0351	sRNA-Xcc1
TT053	413078	413167	90	38.7	XC0353	N	XC0352	XC0354	
TT054	413186	413278	93	29.3	XC0353	N	XC0352	XC0354	
TT055	413571	413631	61	91.3	N	XC0354	XC0353	XC0355	
TT056	419488	419557	70	158.5	N	XC0360	XC0359	XC0361	
TT057	447714	447766	53	276.5	XC0386	N	XC0385	XC0387	
TT058	453239	453295	57	627.5	N	N	XC0389	XC0390	
TT059	453784	453855	72	197.5	XC0390	N	XC0389	XC0391	
TT060	456160	456216	78	64.1	N	XC0394	XC0393	XC0395	
TT061	465779	465848	88	555.1	N	XC4333	XC0402	XC0403	tRNA (XC4333)
TT062	471914	471986	73	102.1	N	N	XC0408	XC0409	
TT063	480582	480641	60	2317.6	N	XC0414	XC0413	XC0415	
TT064	486775	486840	66	105.1	N	N	XC0416	XC0417	
TT065	488417	488478	62	180.9	XC0417	N	XC0416	XC0418	
TT066	491031	491085	55	112.3	XC0419	N	XC0418	XC0420	
TT067	491321	491385	65	53.1	XC0419	N	XC0418	XC0420	
TT068	522064	522154	91	137.8	N	N	XC0438	XC0439	

TT069	522210	522633	423	301.9	N	N	XC0438	XC0439	
TT070	526278	526332	55	76.2	XC0443	N	XC0442	XC0444	
TT071	551604	551667	64	190.7	N	XC0463	XC0462	XC0464	
TT072	574674	574724	51	88.9	XC0485	N	XC0484	XC0486	
TT073	576332	576410	79	200.4	XC0486	N	XC0485	XC0487	
TT074	578891	578983	93	1286.2	N	XC0490	XC0489	XC0491	
TT075	580611	580674	63	2280.7	XC4334	N	XC0491	XC0492	tRNA (XC4334)
TT076	580741	580805	64	1500.3	XC4335	N	XC0491	XC0492	tRNA (XC4335)
TT077	581608	581674	67	107.4	N	XC0493	XC0492	XC0494	
TT078	587862	587940	79	42.3	N	N	XC0497	XC0498	
TT079	621291	621369	79	71.1	N	XC0525	XC0524	XC0526	
TT080	624944	625033	90	42.6	XC0528	N	XC0527	XC0529	
TT081	626223	626282	60	146.7	N	N	XC0530	XC0531	
TT082	632099	632168	70	79.7	N	N	XC0533	XC0534	
TT083	633720	633783	64	64.9	XC0535	N	XC0534	XC0536	
TT084	633817	633876	60	46.2	XC0535	N	XC0534	XC0536	
TT085	639092	639163	72	56.2	XC0538	N	XC0537	XC0539	
TT086	639326	639384	59	134.2	XC0538	N	XC0537	XC0539	
TT087	677097	677186	90	421.1	N	N	XC0563	XC0564	
TT088	697976	698036	61	201.1	XC0584	N	XC0583	XC0585	
TT089	704129	704202	74	261.9	XC0588	N	XC0587	XC0589	
TT090	704921	705070	150	295.1	N	XC0589	XC0588	XC0590	
TT091	752527	752580	54	33.4	N	N	XC0631	XC0632	
TT092	753719	753782	64	115.3	XC0633	N	XC0632	XC0634	
TT093	766606	766671	66	299.9	N	N	XC0638	XC0639	
TT094	767128	767235	108	57.6	XC0639	N	XC0638	XC0640	
TT095	768166	768225	60	297.1	N	XC0639	XC0638	XC0640	
TT096	781908	781975	68	69.4	N	XC0647<br>XC0648	XC0646	XC0649	
TT097	791697	791786	90	38.4	N	XC0658	XC0657	XC0659	
TT098	793036	793100	65	70.2	N	XC0659	XC0658	XC0660	
TT099	793576	793633	58	80.9	XC0659	N	XC0658	XC0660	
TT100	830789	830861	73	253.9	XC0691	N	XC0690	XC0692	
TT101	848204	848259	56	274.8	N	N	XC0705	XC0706	
TT102	848460	848519	60	54.7	XC0706	N	XC0705	XC0707	
TT103	849532	849588	57	155.5	XC0706	N	XC0705	XC0707	
TT104	853570	853651	81	26.5	N	N	XC0709	XC0711	

TT105	853703	853786	84	114.6	N	XC0710	XC0709	XC0711	
TT106	854937	855002	65	158.4	XC0711	N	XC0710	XC0712	
TT107	855059	855156	97	72.7	XC0711	N	XC0710	XC0712	
TT108	857025	857080	56	810.9	N	N	XC0713	XC0714	
TT109	859744	859833	90	38.1	XC0715	N	XC0714	XC0716	
TT110	861445	861551	107	32.9	XC0717	N	XC0716	XC0718	
TT111	862234	862334	101	576.2	N	XC0718	XC0717	XC0719	
TT112	867931	868360	429	1168.1	XC0723	N	XC0722	XC0724	XC0723 mRNA
TT113	867931	868054	124	1111.6	N	XC0723	XC0722	XC0724	
TT114	868058	868137	80	807.7	XC0723	N	XC0722	XC0724	
TT115	868138	868230	93	1962.1	XC0723	N	XC0722	XC0724	
TT116	868271	868360	90	1376.4	N	XC0723	XC0722	XC0724	
TT117	872102	872531	429	494.1	XC0727	N	XC0726	XC0728	XC0727 mRNA
TT118	872436	872531	116	771.7	N	XC0727	XC0726	XC0728	
TT119	872797	872880	84	50.8	N	N	XC0727	XC0728	
TT120	872917	873019	103	304.7	N	XC0728	XC0727	XC0729	
TT121	923871	923936	66	195.5	N	XC0768	XC0767	XC0769	
TT122	951277	951358	82	68.4	N	XC0796	XC0795	XC0797	
TT123	951867	951935	69	194.5	XC0796	N	XC0795	XC0797	
TT124	971506	971565	60	58.1	XC0811	N	XC0810	XC0812	
TT125	997549	997605	56	42.3	XC0830	N	XC0829	XC0831	
TT126	997786	997835	50	102.4	XC0830	N	XC0829	XC0831	
TT127	998027	998098	72	88.6	N	XC0831	XC0830	XC0832	
TT128	1009676	1009743	68	162.5	XC0838	N	XC0837	XC0839	
TT129	1013277	1013372	96	2433.3	N	N	XC0841	XC0842	
TT130	1014431	1014497	67	53.9	XC0842	N	XC0841	XC0843	
TT131	1019341	1019428	88	1519.9	N	N	XC0844	XC0845	
TT132	1019825	1020040	215	178.2	N	XC0846	XC0845	XC0847	
TT133	1022098	1022147	50	360.9	N	N	XC0846	XC0847	
TT134	1025928	1026043	116	207.8	XC0849	N	XC0848	XC0850	
TT135	1034180	1034253	73	43.7	N	N	XC0853	XC0854	
TT136	1034363	1034451	89	100.5	N	XC0854	XC0853	XC0855	
TT137	1035293	1035345	53	137.23	XC0855	N	XC0854	XC0856	
TT138	1036765	1036832	68	13.1	XC0857	N	XC0856	XC0858	
TT139	1037060	1037130	71	79.9	XC0857	N	XC0856	XC0858	
TT140	1051759	1051813	55	52.7	N	N	XC0872	XC0873	

TT141	1051878	1051967	90	140.9	XC0873	N	XC0872	XC0874	
TT142	1052567	1052660	94	149.1	N	N	XC0873	XC0874	
TT143	1074880	1075087	207	25.1	N	XC0891	XC0890	XC0892	
TT144	1080412	1080475	63	195.9	N	N	XC0894	XC0895	
TT145	1085288	1085468	181	49972.9	N	N	XC0901	XC0902	6SRNA (sRNA-*Xcc2*)
TT146	1088492	1088680	188	167.3	N	N	XC0905	XC0906	
TT147	1097403	1097479	77	438.3	XC0916	N	XC0915	XC0917	
TT148	1108108	1108160	53	93.4	N	XC0924	XC0923	XC0925	
TT149	1110302	1110379	78	11.9	N	XC0925	XC0924	XC0926	
TT150	1135264	1135538	274	130.6	XC0945	N	XC0944	XC0946	XC0945 mRNA
TT151	1162926	1163013	88	39.6	N	XC0969	XC0968	XC0970	
TT152	1170965	1171020	56	20.2	N	N	XC0975	XC0976	
TT153	1176661	1176720	59	962.6	XC0980	N	XC0979	XC0981	
TT154	1189861	1189937	77	147.4	XC0991	N	XC0990	XC0992	
TT155	1190631	1190685	55	2095.7	N	N	XC0990	XC0992	
TT156	1222132	1222192	61	195.9	N	XC1009	XC1008	XC1010	
TT157	1222577	1222649	73	155.9	N	XC1009	XC1008	XC1010	
TT158	1230874	1230938	65	493.9	N	XC4336	XC1017	XC1018	tRNA (XC4336)
TT159	1231053	1231129	77	491.6	N	N	XC4336	XC1018	
TT160	1233305	1233394	90	99.5	N	N	XC1018	XC1019	
TT161	1241514	1241570	57	308.1	N	N	XC1023	XC1024	
TT162	1259596	1259664	69	85.2	N	XC1044	XC1043	XC1045	
TT163	1260414	1260464	50	98.6	N	XC1046	XC1045	XC1047	
TT164	1260784	1260859	76	128.1	N	XC1046	XC1045	XC1047	
TT165	1269031	1269101	71	1206.7	N	N	XC1053	XC1054	
TT166	1295653	1295721	69	70.4	N	N	XC1075	XC1076	
TT167	1296858	1296927	70	100.9	XC1076	N	XC1075	XC1077	
TT168	1316918	1317118	201	133.9	N	N	XC1090	XC1091	
TT169	1341918	1341977	60	123.5	N	N	XC1110	XC1111	
TT170	1348210	1348271	62	61.9	XC1113	N	XC1112	XC1114	
TT171	1379140	1379198	59	82.9	XC1136	N	XC1135	XC1137	
TT172	1381928	1382023	96	228.4	N	XC1139<br>XC1140	XC1138	XC1141	
TT173	1383289	1383342	54	96.9	XC1140	N	XC1139	XC1141	
TT174	1387682	1387742	60	308.7	XC4337	N	XC1145	XC1147	tRNA (XC4337)
TT175	1397137	1397310	173	17.2	N	XC1156	XC1155	XC1157	
TT176	1412286	1412380	95	194.1	N	XC1166	XC1165	XC1167	

TT177	1417998	1418254	256	1185.6	N	XC1172	XC1171	XC1173	
TT178	1418118	1418254	136	1567.7	N	N	XC1172	XC1173	
TT179	1427477	1427565	89	32.9	N	XC1177	XC1176	XC1178	
TT180	1427619	1427690	71	29.1	N	N	XC1176	XC1178	
TT181	1469473	1469563	91	354.5	N	XC1210	XC1209	XC1211	
TT182	1508410	1508509	100	150.2	N	XC1234	XC1233	XC1235	
TT183	1511287	1511378	92	150.7	N	XC1235	XC1234	XC1236	
TT184	1531053	1531262	210	456.5	N	XC1251	XC1250	XC1252	
TT185	1558850	1558916	66	16.2	N	N	XC1270	XC1271	
TT186	1577666	1577921	256	92.3	XC1289	N	XC1288	XC1290	XC1289 mRNA
TT187	1578022	1578382	360	60.6	N	XC1290	XC1289	XC1291	
TT188	1578816	1578872	57	71.5	XC1290	N	XC1289	XC1291	
TT189	1594827	1594894	68	53.7	N	XC1303	XC1302	XC1304	
TT190	1596957	1597013	57	45.4	XC1305	N	XC1304	XC1306	
TT191	1600577	1600670	94	300.9	N	XC1311	XC1310	XC1312	
TT192	2109208	1609364	157	171.8	N	XC1321	XC1320	XC1322	
TT193	1618520	1619034	514	997.4	XC1332	N	XC1331	XC1333	XC1332 mRNA
TT194	1618954	1619030	77	5188.6	N	N	XC1332	XC1333	
TT195	1629893	1629982	90	80.3	XC1343	N	XC1342	XC1344	
TT196	1630002	1630092	91	38.6	XC1344	N	XC1342	XC1344	
TT197	1633658	1633717	60	596.1	N	N	XC1346	XC1347	
TT198	1634567	1634660	94	193.9	XC1347	N	XC1346	XC1348	
TT199	1638354	1638439	86	37.8	N	XC1352	XC1351	XC1353	
TT200	1653515	1653608	94	186.7	N	XC1369	XC1368	XC1370	
TT201	1653608	1653737	58	584.9	XC1369	N	XC1368	XC1370	
TT202	1654243	1654307	64	826.2	N	N	XC1369	XC1370	
TT203	1664428	1664494	66	14.3	XC1377	N	XC1376	XC1378	
TT204	1666368	1666457	90	59.7	XC1380	N	XC1379	XC1381	
TT205	1671801	1671860	60	154.9	N	N	XC1383	XC1384	
TT206	1673650	1673706	57	202.4	XC1385	N	XC1384	XC1386	
TT207	1690686	1690779	94	56.2	N	N	XC1401	XC1402	
TT208	1725118	1725256	139	96.3	N	XC1430	XC1428	XC1431	
TT209	1725291	1725380	90	97.8	XC1430	N	XC1429	XC1431	
TT210	1747942	1748009	68	157.8	N	N	XC1450	XC1451	
TT211	1750038	1750126	89	214.1	XC1451	N	XC1450	XC1452	
TT212	1751067	1751161	95	110.3	N	N	XC1451	XC1452	

TT213	1791899	1791966	68	72.1	N	N	XC1485	XC1486	
TT214	1794789	1794847	59	23.4	N	N	XC1488	XC1489	
TT215	1795129	1795191	63	1795129	N	XC1489	XC1488	XC1490	
TT216	1798597	1798657	61	206.9	N	N	XC1492	XC1493	
TT217	1798594	1798658	65	151.2	N	N	XC1492	XC1493	
TT218	1798734	1798971	237	160.1	XC1493	N	XC1492	XC1494	XC1493 mRNA
TT219	1807102	1807157	56	249.3	N	XC1501	XC1500	XC1502	
TT220	1807496	1807551	56	211.5	N	XC1501	XC1500	XC1502	
TT221	1814332	1814398	67	105.9	N	N	XC1507	XC1508	
TT222	1830452	1830516	65	71.7	XC1520	N	XC1519	XC1521	
TT223	1843442	1843503	62	376.9	N	XC1534	XC1533	XC1535	
TT224	1844170	1844248	79	54.9	N	XC1535 XC1536	XC1534	XC1537	
TT225	1857558	1857611	54	90.3	XC1545	N	XC1544	XC1546	
TT226	1878565	1878635	70	15.2	XC1560	N	XC1559	XC1561	
TT227	1878860	1878944	85	32.8	N	N	XC1560	XC1561	
TT228	1891848	1892048	200	179.9	N	XC1572 XC1573	XC1571	XC1574	
TT229	1896637	1896730	94	151.1	N	XC1578	XC1577	XC1579	
TT230	1908312	1908365	53	2282.1	XC4338	N	XC1588	XC1589	tRNA (XC4338)
TT231	1923549	1923619	70	4672.8	XC4339	N	XC1602	XC1603	tRNA (XC4339)
TT232	1925066	1925156	91	93.1	XC1604	N	XC1603	XC1605	
TT233	1925180	1925269	90	134.1	XC1604	N	XC1603	XC1605	
TT234	1925989	1926039	51	152.9	XC1605	N	XC1604	XC1606	
TT235	1935900	1935956	57	51.7	N	XC1612	XC1611	XC1613	
TT236	1952411	1952494	83	3231.1	N	XC4340	XC1626	XC1627	tRNA (XC4340)
TT237	1955313	1955402	90	2951.2	XC4341	N	XC1628	XC1629	tRNA (XC4341)
TT238	1955793	1955849	57	297.2	N	XC1629	XC4341	XC1630	
TT239	1958154	1958316	162	365.4	N	XC1631	XC1630	XC1632	
TT240	1964190	1964283	94	226.1	XC1638	N	XC1637	XC1639	
TT241	1987096	1987333	237	924.1	N	XC1650	XC1649	XC1651	
TT242	1987898	1988088	191	419.5	N	XC1652	XC1651	XC1653	
TT243	1988338	1988392	55	333.3	XC1653	N	XC1652	XC1654	
TT244	1992387	1992456	69	74.6	XC1655	N	XC1654	XC1656	
TT245	1992504	1992592	88	62.9	XC1656	N	XC1655	XC1657	
TT246	1993177	1993237	60	29595.6	XC4342	N	XC1657	XC1658	tRNA (XC4342)

TT247	1993596	1993830	235	639.4	N	XC1658	XC4342	XC1659	
TT248	1996966	1997054	89	87.1	XC1660	N	XC1659	XC1661	
TT249	2017621	2017680	60	193.4	XC1678	N	XC1677	XC1679	
TT250	2018414	2018507	94	634.9	N	XC1679	XC1678	XC1680	
TT251	2022475	2022552	77	215.1	XC4343	N	XC1682	XC1683	tRNA (XC4343)
TT252	2027555	2027808	254	98.9	XC1687	N	XC1686	XC1688	XC1687 mRNA
TT253	2099001	2099060	60	56.7	N	XC1735	XC1734	XC1736	
TT254	2117629	2117706	78	53.3	XC1747	N	XC1746	XC1748	
TT255	2134040	2134267	228	146.5	N	XC1756<br>XC1757	XC1755	XC1758	
TT256	2146561	2146651	90	38.4	XC1765	N	XC1764	XC1766	
TT257	2146707	2146801	95	79.6	N	XC1765	XC1764	XC1766	
TT258	2170287	2170349	63	478.5	N	XC1783	XC1782	XC1784	
TT259	2195687	2195760	74	302.4	N	XC1807	XC1806	XC1808	
TT260	2213815	2213865	51	77.2	XC1820	N	XC1819	XC1821	
TT261	2218048	2218110	63	18.3	XC1824	N	XC1823	XC1825	
TT262	2221405	2221619	214	2615.9	N	XC1828	XC1827	XC1829	
TT263	2223778	2223838	61	58.9	XC1831	N	XC1830	XC1832	
TT264	2230280	2230330	50	9.2	XC1836	N	XC1835	XC1837	
TT265	2230540	2230624	85	272.1	N	XC1836	XC1835	XC1837	
TT266	2248862	2249165	304	79.8	XC1853	N	XC1852	XC1854	XC1853 mRNA
TT267	2256215	2256330	116	72.1	N	XC1860	XC1859	XC1861	
TT268	2278165	2278223	58	9.2	XC1882	N	XC1881	XC1883	
TT269	2278395	2278480	86	356.7	XC4345	N	XC1882	XC1883	tRNA (XC4345)
TT270	2281622	2281711	90	82.2	N	XC1886	XC1885	XC1887	
TT271	2292655	2292725	70	36.2	N	XC1896	XC1895	XC1897	
TT272	2292876	2292945	70	43.6	XC1897	N	XC1896	XC1898	
TT273	2298646	2298706	61	348.8	N	N	XC1903	XC1904	
TT274	2303046	2303141	96	240.9	N	N	XC1906	XC1907	
TT275	2334041	2334111	71	412.3	N	XC1934	XC1933	XC1935	
TT276	2334327	2334413	87	48.7	XC1934	N	XC1933	XC1935	
TT277	2358598	2358674	72	915.7	XC1953	N	XC1952	XC1954	
TT278	2359088	2359178	91	89.2	XC1954	N	XC1953	XC1955	
TT279	2372820	2372917	97	70.2	N	XC1966	XC1965	XC4346	
TT280	2373053	2373118	65	11464.2	XC4346	N	XC1966	XC1967	tRNA (XC4346)
TT281	2373869	2374083	214	90.1	N	XC4347<br>XC4348	XC1967	XC4349	tRNA (XC4347+XC4348)

TT282	2374836	2375033	197	398.1	XC4349 XC4350	N	XC4348	XC1968	tRNA (XC4349+XC4350)
TT283	2414123	2414194	71	452.2	XC4351	N	XC2001	XC4352	tRNA (XC4351)
TT284	2414283	2414352	70	35981.3	XC4352	N	XC4351	XC4353	tRNA (XC4352)
TT285	2414412	2414492	81	1081.9	XC4353	N	XC4352	XC2002	tRNA (XC4353)
TT286	2418445	2418513	69	719.7	XC2004	N	XC2003	XC2005	
TT287	2426223	2426278	56	57.9	N	N	XC2012	XC2013	
TT288	2428848	2428904	57	389.2	N	N	XC2014	XC2015	
TT289	2431128	2431185	58	105.8	N	N	XC2016	XC2017	
TT290	2438148	2438207	60	87.9	XC2022	N	XC2021	XC2023	
TT291	2438490	2438555	65	99.8	XC2022	N	XC2021	XC2023	
TT292	2438665	2438733	69	275.8	N	XC2023	XC2022	XC2024	
TT293	2443942	2444096	154	95.3	XC2029	N	XC2028	XC2030	
TT294	2455991	2456046	56	114.9	XC2040	N	XC2039	XC2041	
TT295	2458026	2458115	90	85.7	N	XC2044	XC2043	XC2045	
TT296	2509851	2509912	62	89.1	XC2087	N	XC2086	XC2088	
TT297	2513401	2513472	72	411.7	N	N	XC2089	XC2090	
TT298	2513787	2513873	87	164.7	XC2090	ULL	XC2089	XC4354	
TT299	2515196	2515268	72	7742.2	XC4354	N	XC2089	XC2090	tRNA (XC4354)
TT300	2524765	2524854	90	471.1	N	N	XC2098	XC2099	
TT301	2524870	2524968	98	1151.5	N	N	XC2098	XC2099	
TT302	2528080	2528154	75	69.3	N	XC2102	XC2101	XC2103	
TT303	2551037	2551103	67	44.3	XC2128	N	XC2127	XC2129	
TT304	2566429	2566739	311	93.3	N	N	XC2138	XC2139	
TT305	2604812	2604903	92	50.4	N	N	XC2160	XC2161	
TT306	2605473	2605544	72	112.4	XC2161	N	XC2160	XC2162	
TT307	2609805	2609908	104	279.9	N	XC2164	XC2163	XC2165	
TT308	2611091	2611380	290	390.1	XC2165	N	XC2164	XC2166	
TT309	2613155	2613216	62	82.6	N	N	XC2167	XC2168	
TT310	2632909	2633006	97	109.1	XC2186	N	XC2185	XC2187	
TT311	2650241	2650294	54	89.3	XC2201	N	XC2200	XC2202	
TT312	2652059	2652116	58	286.5	N	XC2203	XC2202	XC2204	
TT313	2656886	2656937	108	444.9	N	N	XC2206	XC2208	
TT314	2664818	2664928	110	33.9	N	XC2211	XC2210	XC2212	
TT315	2669660	2669738	79	109.2	N	XC2214	XC2213	XC2215	
TT316	2672746	2673016	270	138.6	N	XC2217	XC2216	XC2218	XC2217 mRNA
TT317	2674533	2674591	59	157.6	XC2218	N	XC2217	XC2219	

TT318	2675156	2675268	113	154.6	XC2218	N	XC2217	XC2219	
TT319	2675595	2675684	90	686.3	XC2218	N	XC2217	XC2219	
TT320	2676130	2676194	65	118.7	N	XC2219	XC2218	XC2220	
TT321	2686474	2686558	85	143.8	N	XC2227<br>XC2228	XC2226	XC2229	
TT322	2687002	2687059	58	37.4	XC2228	N	XC2227	XC2229	
TT323	2688285	2688345	61	133.6	N	XC2228	XC2227	XC2229	
TT324	2696762	2696842	81	437.1	N	N	XC2238	XC2239	
TT325	2697318	2697449	132	37.7	N	XC2239	XC2238	XC2240	
TT326	2708703	2708768	66	346.8	XC2249	N	XC2248	XC2250	
TT327	2711028	2711082	55	122.8	XC2251	N	XC2250	XC2252	
TT328	2711448	2711512	64	795.6	2711448	2711512	64	795.6	
TT329	2712357	2712419	63	95.3	XC2253	N	XC2252	XC2254	
TT330	2742223	2742276	54	47.1	N	XC2277	XC2276	XC2278	
TT331	2742505	2742577	72	21.6	XC2277	N	XC2276	XC2278	
TT332	2760980	2761064	84	395.3	N	XC2293	XC2292	XC2294	
TT333	2768652	2768730	79	34.9	N	N	XC2296	XC2297	
TT334	2840642	2840754	113	35.5	N	XC2348	XC2347	XC2349	
TT335	2899441	2899500	60	3893.1	XC4355	N	XC2396	XC2399	tRNA (XC4355)
TT336	2909838	2909913	76	64.2	N	XC2403	XC2402	XC2404	
TT337	2919083	2919303	220	244.1	N	XC2410	XC2409	XC2411	
TT338	2926371	2926572	201	255.2	N	XC2414	XC2413	XC2415	
TT339	2929115	2929355	240	657.2	XC2418	N	XC2417	XC2419	
TT340	2932972	2933220	248	196.1	N	N	XC2422	XC2423	
TT341	2939567	2939752	185	8230	N	XC2426	XC2425	XC2427	
TT342	2939567	2939638	71	2944.9	N	XC2426	XC2425	XC2427	
TT343	2940875	2940934	60	151.9	N	XC2426<br>XC2427	XC2425	XC2428	
TT344	2944151	2944205	55	924.9	N	N	XC2429	XC2430	
TT345	2945183	2945246	64	76.6	N	XC2430	XC2429	XC2431	
TT346	2972679	2972743	65	230.1	N	N	XC2458	XC2459	
TT347	2987874	2987928	55	190.1	XC2470	N	XC2469	XC4356	
TT348	2987988	2988049	61	2706.8	XC4356	N	XC2469	XC2471	tRNA (XC4356)
TT349	3024007	3024079	73	51.1	N	XC2495<br>XC2496	XC2494	XC2497	
TT350	3028347	3028440	93	752.9	N	XC4358	XC4357	XC2501	
TT351	3035112	3035198	86	1712.1	XC4359	N	XC2505	XC2506	tRNA (XC4359)

TT352	3035652	3035743	92	556.7	XC2507	N	XC2506	XC2508	
TT353	3038601	3038661	61	84.4	XC2508	N	XC2507	XC2509	
TT354	3038922	3038984	63	709.1	N	N	XC2508	XC2509	
TT355	3039995	3040047	53	133.5	N	XC2509	XC2508	XC2510	
TT356	3048398	3048450	53	232.1	XC2515	N	XC2514	XC2516	
TT357	3050859	3050915	57	183.4	XC2518	N	XC2517	XC2519	
TT358	3053031	3053085	55	305.5	XC2519	N	XC2518	XC2520	
TT359	3054244	3054427	184	39.6	N	N	XC2521	XC2522	
TT360	3063812	3063867	56	96.2	N	N	XC2533	XC2534	
TT361	3063918	3063995	78	110.6	N	N	XC2533	XC2534	
TT362	3066078	3066187	110	534.3	XC2535	N	XC2534	XC2536	
TT363	3068395	3068473	79	39.7	N	XC2537	XC2536	XC2538	
TT364	3077469	3077539	71	348.1	XC2545	N	XC2544	XC2546	
TT365	3132991	3133083	93	38.4	N	N	XC2595	XC2596	
TT366	3148184	3148415	231	203.4	N	XC2607	XC2606	XC2608	
TT367	3148927	3149006	90	629.5	N	N	XC2607	XC2608	
TT368	3167243	3167332	90	161.7	N	XC2630	XC2629	XC2631	
TT369	3167739	3167812	74	242.2	N	XC2631	XC2630	XC2632	
TT370	3167857	3167944	88	184.6	XC2632	N	XC2631	XC2633	
TT371	3173234	3173295	62	153.9	XC2636	N	XC2635	XC2637	
TT372	3175544	3175610	67	171.9	XC2638	N	XC2637	XC4360	
TT373	3176577	3176723	147	398	XC2638	N	XC2637	XC4360	
TT374	3177001	3177086	90	411.5	XC4360	N	XC2638	XC2639	tRNA (XC4360)
TT375	3177097	3177203	107	191.1	N	N	XC4360	XC2639	
TT376	3180693	3180759	67	351.2	XC2642	N	XC2641	XC2643	
TT377	3184370	3184450	81	34.4	XC2645	N	XC2644	XC2646	
TT378	3188220	3188336	117	167.4	N	XC2650	XC2649	XC2651	
TT379	3188900	3188954	55	1677.7	N	N	XC2650	XC2651	
TT380	3219243	3219349	107	147.1	N	XC2672	XC2671	XC2673	
TT381	3245880	3245946	67	843.6	N	N	XC2699	XC2700	
TT382	3267573	3267649	77	5400.3	XC4361	N	XC2718	XC2719	tRNA (XC4361)
TT383	3283476	3283530	55	86.2	N	XC2731	XC2730	XC2732	
TT384	3293595	3293674	80	37.5	N	N	XC2742	XC2743	
TT385	3300714	3300975	262	362.8	XC2749	N	XC2748	XC2750	
TT386	3320361	3320415	55	147.6	XC2763	N	XC2762	XC2764	
TT387	3322074	3322155	82	319.6	XC2764	N	XC2763	XC2765	

TT388	3326115	3326169	55	292.9	N	XC2767	XC2766	XC2768	
TT389	3330944	3331397	454	195507.2	XC4362	N	XC2772	XC2773	tmRNA
TT390	3383191	3383473	282	709.1	XC2810	N	XC2809	XC2811	
TT391	3383686	3383997	311	383.5	N	XC2810	XC2809	XC2811	
TT392	3386925	3387070	146	424.8	N	XC2815	XC2814	XC2816	
TT393	3387271	3387359	89	139.1	N	XC2816	XC2815	XC2817	
TT394	3387384	3387473	90	367.5	N	N	XC2816	XC2817	
TT395	3388879	3388930	52	166.9	XC2818	N	XC2817	XC2819	
TT396	3395492	3395587	96	201.1	N	XC2824	XC2823	XC2825	
TT397	3395646	3395735	90	90.8	XC2824	N	XC2823	XC2825	
TT399	3396012	3396079	68	137.1	XC2824	N	XC2823	XC2825	
TT399	3396261	3396372	112	74.1	N	XC2824	XC2823	XC2825	
TT400	3398279	3398362	84	24.9	XC2826	N	XC2825	XC2827	
TT401	3399972	3400047	76	136.1	N	XC2827	XC2826	XC2828	
TT402	3424761	3424838	78	25.8	XC2846	N	XC2845	XC2847	
TT403	3429490	3429577	88	150.1	N	N	XC2848	XC2850	
TT404	3443612	3443735	124	446.3	N	N	XC2862	XC2863	
TT405	3444555	3444617	63	172.3	XC2863	N	XC2862	XC2864	
TT406	3448563	3448651	89	150.3	N	XC2868	XC2867	XC2869	
TT407	3448741	3448795	55	71.6	XC2868	N	XC2867	XC2869	
TT408	3449931	3449984	54	51.1	XC2870	N	XC2869	XC2871	
TT409	3451808	3452033	225	92.3	N	XC2871 XC2872	XC2870	XC2873	
TT410	3453636	3453711	76	81.5	XC2873	N	XC2872	XC2874	
TT411	3455442	3455508	67	78.8	XC2873	N	XC2872	XC2874	
TT412	3455703	3455769	67	130.8	N	XC2873	XC2872	XC2874	
TT413	3467621	3467730	110	819.6	XC2885	N	XC2884	XC2886	
TT414	3468615	3468951	336	287.4	N	XC2885	XC2884	XC2886	
TT415	3468827	3468901	74	574.8	XC2885	N	XC2884	XC2886	
TT416	3469025	3469110	85	43.2	N	N	XC2885	XC2886	
TT417	3481118	3481187	70	338.4	N	XC2897	XC2896	XC2898	
TT418	3481288	3481348	60	91.1	N	XC2898	XC2897	XC2899	
TT419	3481945	3482023	78	349.8	N	N	XC2898	XC2899	
TT420	3487609	3487715	107	105.9	N	N	XC2900	XC2901	
TT421	3500373	3500433	61	67.7	N	N	XC2915	XC2916	
TT422	3506655	3506749	95	1124.5	N	XC2921	XC2920	XC2922	

TT423	3518444	3518523	79	125.5	XC2933	N	XC2932	XC2934	
TT424	3519275	3519326	52	213.4	XC2934	N	XC2933	XC2935	
TT425	3519856	3519935	80	118.2	N	N	XC2934	XC2935	
TT426	3521764	3521855	91	65.2	N	XC2937	XC2936	XC2938	
TT427	3521892	3512207	316	494.9	XC2938	N	XC2937	XC2939	XC2938 mRNA
TT428	3522100	3522168	68	1754.6	N	XC2938	XC2937	XC2939	
TT429	3523176	3523265	90	72.1	N	N	XC2940	XC2941	
TT430	3527193	3527272	80	158.2	XC2942	N	XC2941	XC2943	
TT431	3527492	3527576	85	71.3	N	XC2943	XC2942	XC2944	
TT432	3528755	3528811	57	360.2	N	XC2944	XC2943	XC2945	
TT433	3528827	3528940	114	100.4	XC2944	N	XC2943	XC2945	
TT434	3528983	3529097	115	49.2	XC2944	N	XC2943	XC2945	
TT435	3529123	3529194	71	147.9	N	XC2944	XC2943	XC2945	
TT436	3531660	3531777	118	31.8	N	XC2947	XC2946	XC2948	
TT437	3549990	3550063	74	834.1	N	XC2965	XC2964	XC2966	
TT438	3558182	3558251	70	116.7	N	XC2972	XC2971	XC2973	
TT439	3559134	3559223	90	293.9	N	XC2973<br>XC2974	XC2972	XC2975	
TT440	3559248	3559346	98	999.5	XC2974	N	XC2973	XC2975	
TT441	3559708	3559804	97	1462.7	N	XC2974	XC2973	XC2975	
TT442	3563159	3563274	116	28.6	N	XC2976	XC2975	XC2977	
TT443	3596895	3596986	91	145.2	N	N	XC3001	XC3002	
TT444	3597210	3597299	90	168.8	N	XC3002	XC3001	XC3003	
TT445	3597565	3597654	90	174.5	XC3002	N	XC3001	XC3003	
TT446	3605262	3605378	117	75.6	N	XC3011	XC3010	XC3012	
TT447	3605549	3605652	104	303.1	N	XC3012	XC3011	XC3013	
TT448	3605682	3605743	61	268.6	XC3012	N	XC3011	XC3013	
TT449	3609150	3609243	94	400.3	N	XC3014	XC3013	XC3015	
TT450	3619443	3619520	78	210.6	N	N	XC3025	XC3026	
TT451	3240766	3040810	44	36.2	N	N	XC3039	XC3040	
TT452	3640864	3641322	459	88.5	XC3040	N	XC3039	XC3041	XC3040 mRNA
TT453	3642707	3642822	116	247.7	N	XC3042<br>XC3043	XC3041	XC3044	
TT454	3642824	3642908	85	332.1	XC3043	N	XC3042	XC3044	
TT455	3642995	3643053	59	253.9	N	XC3043	XC3042	XC3044	
TT456	3651162	3651256	95	30.2	XC3050	N	XC3049	XC3051	
TT457	3657767	3657856	90	81.6	XC3056	N	XC3055	XC3057	

TT458	3657890	3657983	93	76.7	XC3056	N	XC3055	XC3057	
TT459	3684840	3684929	90	126.3	N	N	XC3076	XC3077	
TT460	3688259	3688335	77	161.1	N	XC3079	XC3078	XC3080	
TT461	3696407	3696464	58	128.5	N	XC3085	XC_4364	XC3086	
TT462	3703180	3703535	356	1727	XC3090	N	XC3089	XC3091	XC3090 mRNA
TT463	3710523	3710605	83	38.7	XC3097	N	XC3096	XC3098	
TT464	3711936	3712009	74	490.7	N	N	XC3098	XC3099	
TT465	3713524	3713638	114	1456.1	N	XC3100	XC3099	XC3101	
TT466	3713657	3713749	92	1404.7	XC3100	N	XC3099	XC3101	
TT467	3714229	3714329	100	869.9	N	XC3100	XC3099	XC3101	
TT468	3728975	3729058	84	136.2	XC3119	N	XC3118	XC3121	
TT469	3731165	3731244	80	53.3	N	XC3122<br>XC3123	XC3121	XC3124	
TT470	3731384	3731454	71	148.2	XC3123	N	XC3122	XC3124	
TT471	3736036	3736086	51	349.9	N	XC3128	XC3127	XC3129	
TT472	3737338	3737417	80	133.3	XC3129	N	XC3128	XC3130	
TT473	3745231	3745408	177	261.5	N	N	XC3134	XC3135	
TT474	3755519	3755604	86	77.9	N	XC3142	XC3141	XC3143	
TT475	3782877	3782977	101	551.2	XC3160	N	XC3159	XC3161	
TT476	3809409	3809576	168	1063.2	N	N	XC3179	XC3180	
TT477	3810025	3810150	126	100.2	XC3180	N	XC3179	XC3181	
TT478	3831134	3831207	74	333.5	N	XC3196	XC3195	XC3197	
TT479	3831853	3831910	58	181.6	N	N	XC3196	XC3197	
TT480	3850534	3850594	61	169.5	N	N	XC3207	XC3208	
TT481	3866289	3866358	70	853.8	XC4365	N	XC3220	XC3221	tRNA (XC4365)
TT482	3872958	3873047	90	53.6	N	XC3227	XC3226	XC3228	
TT483	3881882	3881942	61	347.5	N	XC3236<br>XC3237	XC3235	XC3238	
TT484	3890025	3890130	106	5833.3	N	N	XC3244	XC4366	sRNA-Xcc3
TT485	3890231	3890294	63	14395.5	XC4366	N	XC3244	XC3245	tRNA (XC4366)
TT486	3896282	3896544	263	2516.6	N	XC4367	XC3253	XC3254	
TT487	3896462	3896525	63	8148.2	XC4367	N	XC3253	XC3254	tRNA (XC4367)
TT488	3899791	3899900	109	158.6	XC3258	N	XC3257	XC3259	
TT489	3904927	2905249	323	303.1	XC_4369	XC4368<br>XC4370	XC3261	XC3262	tRNA<br>(XC4368/XC4369/XC4370)
TT490	3910473	3910593	121	516.5	XC3266	N	XC3265	XC4371	
TT491	3912154	3912219	66	9800.2	XC4372	N	XC4371	XC4373	tRNA (XC4372)

TT492	3912313	3912380	68	3377.2	XC4373	N	XC4372	XC4374	tRNA (XC4373)
TT493	3914010	3914101	92	232.5	XC3268	N	XC3267	XC3269	
TT494	3915254	3915308	55	160.3	XC3269	N	XC3268	XC3270	
TT495	3915732	3915793	62	321.2	XC3270	N	XC3269	XC3271	
TT496	3731672	3731762	91	40.3	XC3283	N	XC3282	XC3284	
TT497	3931796	3931888	92	138.1	N	XC3283	XC3282	XC3284	
TT498	3942597	3942687	91	115.2	XC3290	N	XC3289	XC3291	
TT499	3946746	3946806	61	598.8	N	XC3294<br>XC3295	XC3293	XC3296	
TT500	3951120	3951214	95	810.8	XC3300	N	XC3299	XC3301	
TT501	3952166	3952255	90	953.2	N	XC3300	XC3299	XC3301	
TT502	3952270	3952344	74	964.4	N	N	XC3330	XC3301	
TT503	3953842	3953969	128	615.9	N	N	XC3301	XC3302	
TT504	3959367	3959450	84	320.9	N	N	XC3304	XC3305	
TT505	3960669	3960726	58	68.4	N	N	XC3306	XC3307	
TT506	3975385	3975452	68	210.9	N	XC3320	XC3319	XC3321	
TT507	3980310	3980391	82	127.5	XC3332	N	XC3331	XC3333	
TT508	3985177	3985256	80	516.2	N	N	XC3341	XC3342	
TT509	3998705	3998872	167	234.6	N	XC3348	XC3347	XC3349	
TT510	4002507	4002557	51	112.5	N	N	XC3353	XC4376	
TT511	4002575	4002641	67	172.4	XC4376	N	XC3353	XC3354	tRNA (XC4376)
TT512	4002749	4002839	91	97.5	N	XC3354	XC4376	XC4377	
TT513	4003949	4004046	98	354.4	N	XC4377<br>XC4378	XC3354	XC4379	tRNA (XC4377)
TT514	4004102	4004167	65	1468.2	XC4378	N	XC4377	XC4379	tRNA (XC4378)
TT515	4004215	4004327	112	470.9	N	XC4378<br>XC4379	XC4377	XC3355	tRNA (XC4379)
TT516	4008125	4008194	69	8351.1	XC4380	N	XC3358	XC3359	tRNA (XC4380)
TT517	4008216	4008458	242	341.5	XC3359	N	XC4380	XC3360	
TT518	4011324	4011438	115	62.2	N	XC3361	XC3360	XC3362	
TT519	4011451	4011543	93	184.7	N	XC3362	XC3361	XC3363	
TT520	4029376	4029439	64	458.5	XC3379	N	XC3378	XC3380	
TT521	4032184	4032259	76	99.1	XC3381	N	XC3380	XC3382	
TT522	4036140	4036208	69	97.9	XC3386	N	XC3385	XC3387	
TT523	4048247	4048323	77	218.6	XC3396	N	XC3395	XC3397	
TT524	4054079	4054210	132	152.7	N	XC3402	XC3401	XC3403	
TT525	4089013	4089106	93	59.6	XC3438	N	XC3437	XC3439	

TT526	4089148	4089234	87	67.5	XC3438	N	XC3437	XC3439	
TT527	4098603	4098678	76	27.9	N	XC3450	XC3449	XC3451	
TT528	4101774	4101839	66	547.8	XC3452	N	XC3451	XC3453	
TT529	4105917	4106041	125	300.6	N	XC3456	XC3455	XC3457	
TT530	4106078	4106161	113	374.3	XC3456	N	XC3455	XC3457	
TT531	4114612	4114817	205	243.4	XC3464	N	XC3463	XC3465	XC3464 mRNA
TT532	4122300	4122374	75	447.4	N	XC3471	XC3470	XC3472	
TT533	4137310	4137368	59	122.6	N	XC3479	XC3478	XC3481	
TT534	4140268	4140451	183	413.3	N	XC3482	XC3481	XC3483	
TT535	4142107	4142181	75	50.7	N	N	XC3482	XC3483	
TT536	4153845	4153937	93	147.3	N	XC3496	XC3495	XC3497	
TT537	4154212	4154341	130	100.2	XC3496	N	XC3495	XC3497	
TT538	4162147	4162217	71	389.2	XC3503	N	XC3502	XC3504	
TT539	4162383	4162445	63	89.8	XC3503	N	XC3502	XC3504	
TT540	4162878	4162954	77	211.9	XC3504	N	XC3503	XC3505	
TT541	4165010	4165095	86	76.7	N	XC3505	XC3504	XC3506	
TT542	4177501	4177573	73	46.9	XC3515	N	XC3514	XC3516	
TT543	4180020	4180304	285	86.6	N	XC3518	XC3517	XC3519	
TT544	4182260	4182605	345	20292.1	misc_RNA	N	XC3521	XC3522	RnpB RNA
TT545	4203279	4203464	186	307.1	N	N	XC3539	XC3540	
TT546	4203604	4203672	69	76.9	N	XC3540	XC3539	XC3541	
TT547	4207781	4207841	61	216.2	N	XC3544	XC3543	XC3545	
TT548	4207977	4208207	231	591.7	N	XC3545	XC3544	XC3546	
TT549	4208448	4208572	125	1910.7	N	XC3545	XC3544	XC3546	
TT550	4208814	4208875	62	106.3	XC3546	N	XC3545	XC3547	
TT551	4223588	4223655	68	83.9	N	N	XC3557	XC3558	
TT552	4256839	4256912	74	340.1	XC3579	N	XC3578	XC3580	
TT553	4257233	4257332	100	56.5	N	XC3579	XC3578	XC3580	
TT554	4261827	4261905	73	487.2	N	N	XC3584	XC3585	
TT555	4276767	4276830	64	725.7	N	N	XC3596	XC3597	
TT556	4277484	4277545	62	167.7	XC3598	N	XC3597	XC3599	
TT557	4283435	4283495	61	9.5	XC3603	N	XC3602	XC3604	
TT558	4283660	4283955	296	56.3	N	XC3604	XC3603	XC3605	
TT559	4299726	4299807	92	82.3	XC3618	N	XC3617	XC3619	
TT560	4305698	4305751	54	71.5	N	N	XC3625	XC3626	
TT561	4307826	4307891	66	73.9	N	XC3628	XC3627	XC3629	

TT562	4314352	4314420	69	103.8	XC3632	N	XC3631	XC3633	
TT563	4321220	4321278	59	30.9	XC3638	N	XC3637	XC3639	
TT564	4325572	4325730	158	527.7	N	N	XC3642	XC3643	
TT565	4325578	4325645	67	1014.4	N	N	XC3642	XC3643	
TT566	4327830	4328260	431	1518.1	XC3645	N	XC3644	XC3646	XC3645 mRNA
TT567	4327830	4327882	53	5736.2	N	XC3645	XC3644	XC3646	
TT568	4328118	4328194	76	4608.9	N	XC3645	XC3644	XC3646	
TT569	4344293	4344482	190	221.9	N	XC3659	XC3658	XC3660	
TT570	4344297	4344393	97	324.4	N	XC3659	XC3658	XC3660	
TT571	4344414	4344482	69	154.6	N	N	XC3659	XC3660	
TT572	4360151	4360233	83	104.3	N	N	XC3672	XC3673	
TT573	4364450	4364513	64	40.9	N	XC3676	XC3675	XC3677	
TT574	4364624	4364711	88	173.3	N	XC3677	XC3676	XC3678	
TT575	4364742	4364839	97	112.2	XC3677	N	XC3676	XC3678	
TT576	4365101	4365191	91	802.2	N	N	XC3677	XC3678	
TT577	4371817	4371917	101	106.4	XC3685	N	XC3684	XC3686	
TT578	4371971	4372060	90	126.1	N	XC3685	XC3684	XC3686	
TT579	4372202	4372293	92	322.8	N	N	XC3685	XC3686	
TT580	4372499	4372665	167	437.6	N	XC3686	XC3685	XC3687	
TT581	4379005	4379095	91	43.3	XC3691	N	XC3690	XC3692	
TT582	4379127	4379216	90	66.1	XC3691	N	XC3690	XC3692	
TT583	4379743	4379816	74	51.1	N	XC3692	XC3691	XC3693	
TT584	4398939	4399013	75	791.9	N	XC3711	XC3710	XC3712	
TT585	4404218	4404294	77	314.9	XC3717	N	XC3716	XC3718	
TT586	4404650	4404744	94	708.2	XC3717	N	XC3716	XC3718	
TT587	4407445	4407503	59	174.4	N	XC3720	XC3719	XC3721	
TT588	4409083	4409150	68	106.1	N	XC3722	XC3721	XC3723	
TT589	4414867	4414937	71	30.6	N	XC3729	XC3728	XC3730	
TT590	4433479	4433579	101	54.9	XC3744	N	XC3743	XC3745	
TT591	4443157	4443419	263	227.3	N	XC3754	XC3753	XC3755	
TT592	4451453	4451532	80	559.8	XC3761	N	XC3760	XC3762	
TT593	4455684	4455754	71	80.8	XC3766	N	XC3765	XC3767	
TT594	4466873	4466962	90	104.5	N	XC3775	XC3774	XC3776	
TT595	4467352	4467466	115	118.6	XC3776	N	XC3775	XC3777	
TT596	4469094	4469207	114	98.9	N	XC3779	XC3778	XC3780	
TT597	4469312	4469372	61	94.9	N	N	XC3779	XC3780	

TT598	4495726	4495826	102	305.6	N	N	XC3801	XC3802	
TT599	4508950	4509041	92	332.6	XC3814	N	XC3813	XC3815	
TT600	4510347	4510416	70	52.8	N	XC3815	XC3814	XC3816	
TT601	4517683	4517754	72	113.5	XC3821	N	XC3820	XC3822	
TT602	4517831	4517924	94	330.7	N	XC3821<br>XC3822	XC3820	XC3823	
TT603	4517956	4518039	90	381.5	XC3822	N	XC3821	XC3823	
TT604	4527317	4527368	52	159.3	N	XC3830	XC3829	XC3831	
TT605	4546818	4546871	54	500.3	N	N	XC3848	XC3849	
TT606	4546883	4546990	107	329.1	N	XC3849	XC3848	XC3850	
TT607	4547381	4547437	57	716.5	N	N	XC3849	XC3850	
TT608	4557051	4557138	87	407.9	N	XC3856	XC3855	XC3857	
TT609	4559479	4559533	55	14.2	N	XC3585	XC3584	XC3586	
TT610	4559652	4559742	91	311.9	N	N	XC3858	XC3859	
TT611	4559818	4559884	67	92.3	XC3859	N	XC3858	XC3860	
TT612	4572028	4572079	52	114.4	N	XC3865	XC3864	XC3866	
TT613	4573570	4573631	62	108.5	N	N	XC3867	XC3868	
TT614	4575097	4575235	139	231.9	N	N	XC3869	XC3870	
TT615	4575554	4575632	79	139.4	N	XC3870	XC3869	XC3871	
TT616	4586683	4586871	189	118.4	XC3882	N	XC3881	XC3883	
TT617	4587350	4587674	325	204.4	XC3883	N	XC3882	XC3884	XC3883 mRNA
TT618	4587350	4587408	59	545.9	XC3883	N	XC3882	XC3884	
TT619	4589554	4589608	55	478.1	N	N	XC3885	XC3886	
TT620	4594306	4594362	57	179.1	N	N	XC3889	XC3890	
TT621	4610834	4610889	56	22.5	XC3907	N	XC3906	XC3908	
TT622	4618882	4619047	165	1307.8	N	N	XC3912	XC3913	
TT623	4618882	4618947	65	2699.8	N	N	XC3912	XC3913	
TT624	4637937	4638023	87	3640.8	N	N	XC4386	XC3925	
TT625	4642808	4642902	95	99.1	N	XC3928	XC3927	XC3929	
TT626	4643585	4643661	77	29.6	XC3929	N	XC3928	XC3930	
TT627	4646038	4646102	65	474.6	N	XC3931	XC3930	XC3932	
TT628	4669328	4669448	121	47.4	N	N	XC3952	XC3953	
TT629	4674462	4674571	110	24.1	N	N	XC3955	XC3956	
TT630	4678700	4678943	244	2141.7	XC3961	XC3961	XC3960	XC3962	XC3961 mRNA
TT631	4678850	4678945	105	4811.5	N	XC3961	XC3960	XC3962	
TT632	4683269	4683337	69	174.8	XC3965	N	XC3964	XC3966	
TT633	4683418	4683578	160	160.6	XC3965	N	XC3964	XC3966	

TT634	4685557	4685687	131	612.3	XC3968	XC3968	XC3967	XC3969	XC3968 mRNA
TT635	4685562	4685622	61	996.8	N	XC3968	XC3967	XC4387	
TT636	4685823	4685908	86	484.3	N	N	XC3968	XC4387	
TT637	4689116	4689212	97	321.3	N	XC3971	XC3970	XC3972	
TT638	4692392	4692484	92	297.7	XC3974	N	XC3973	XC3975	
TT639	4701609	4701678	70	81.5	N	XC3986	XC3985	XC3987	
TT640	4702331	4702421	91	88.4	XC3986	N	XC3985	XC3987	
TT641	4703172	4703262	91	270.1	N	XC3987	XC3986	XC3988	
TT642	4705783	4705854	72	262.8	N	N	XC3990	XC3991	
TT643	4732595	4732653	59	189.4	N	XC4016	XC4015	XC4017	
TT644	4735193	4735282	90	156.9	N	N	XC4019	XC4020	
TT645	4735457	4735513	56	26.5	XC4020	N	XC4019	XC4021	
TT646	4738459	4738522	63	89933.7	XC4389	N	XC4023	XC4024	tRNA (XC4389)
TT647	4747532	4747590	85	920.7	XC4032	N	XC4031	XC4033	
TT648	4747790	4747883	94	3275.1	N	XC4032	XC4031	XC4033	
TT649	4762773	4762863	90	81.6	XC4047	N	XC4046	XC4048	
TT650	4797114	4797205	92	790.3	N	N	XC4076	XC4077	
TT651	4815362	4815422	61	150.3	N	XC4087	XC4086	XC4088	
TT652	4828507	4828590	84	112.9	N	XC4100	XC4099	XC4101	
TT653	4839865	4839956	92	17436.3	N	N	XC4108	XC4109	
TT654	4849090	4849154	65	154.2	N	N	XC4115	XC4116	
TT655	4857835	4857887	53	89.5	N	XC4122	XC4121	XC4123	
TT656	4857835	4858443	610	58.7	N	XC4122 XC4123	XC4121	XC4124	XC4122+XC4123 mRNA
TT657	4871721	4871920	200	85.8	N	XC4133	XC4132	XC4134	XC4133 mRNA
TT658	4895422	4895595	174	18.3	N	XC4152	XC4151	XC4153	
TT659	4895947	4896240	294	473.9	N	XC4152 XC4153	XC4151	XC4154	XC4153 mRNA
TT660	4895947	4896120	174	699.7	N	XC4152 XC4153	XC4151	XC4154	
TT661	4907646	4907735	90	67.1	XC4163	N	XC4162	XC4164	
TT662	4907779	4907870	92	64.1	XC4163	N	XC4162	XC4164	
TT663	4941041	4941255	215	161.8	XC4185	N	XC4184	XC4186	
TT664	4961230	4961296	67	130.4	N	XC4199	XC4198	XC4200	
TT665	4991506	4991585	80	13.1	N	N	XC4221	XC4222	
TT666	4995813	4995868	56	101.5	XC4223	N	XC4222	XC4224	
TT667	5025143	5025415	272	1546.5	N	N	XC_4393	XC4240	

TT668	5025323	5025415	93	4130.9	N	N	XC4393	XC4240	
TT669	5034040	5034162	123	74.7	N	XC4245	XC4244	XC4246	
TT670	5048477	5048580	104	93.1	XC4256	N	XC4255	XC4257	
TT671	5060296	5060361	66	46.3	XC4266	N	XC4265	XC4267	
TT672	5066546	5066635	90	577.7	XC4273	N	XC4272	XC4274	
TT673	5068506	5068612	107	110.9	N	N	XC4273	XC4274	
TT674	5120450	5120510	61	137.9	N	XC4313	XC4312	XC4314	
TT675	5130309	5130381	73	109.8	N	N	XC4320	XC4321	
TT676	5141724	5141917	194	33.3	N	XC4327	XC4326	XC4328	

*N, Null.
