# Supplementary material for: Genome‐wide screen and functional analysis in Xanthomonas reveal a large number of mRNA‐derived sRNAs, including the novel RsmA‐sequester RsmU
Source: Mol Plant Pathol. 2020 Sep 23;21(12):1573–90. doi: 10.1111/mpp.12997 (PMC7694677; doi:10.1111/mpp.12997)
Supplement: Supplementary file 16 — TABLE S4 A summary of the 612 identified sRNA candidates (SRCs) [file MPP-21-1573-s016.pdf]

Table S4. A summary of the 612 identified sRNA candidates (SRCs)#

SRC name	TT name	Length (bp)	RPKM value	Location
SRC001	TT001	115	148.1	5'UTR
SRC002	TT002	97	263.6	5'UTR
SRC003	TT003	65	201	5'UTR
SRC004	TT004	80	223.4	5'UTR
SRC005	TT005	67	100.3	Inside
SRC006	TT006	92	221.4	3'UTR
SRC007	TT007	63	45.7	Inside
SRC008	TT008	59	80.5	Inside
SRC009	TT009	82	212.9	5'UTR
SRC010	TT010	77	95.1	Inside
SRC011	TT011	51	117.4	3'UTR
SRC012	TT012	56	69.7	IGR
SRC013	TT013	90	107.1	Inside
SRC014	TT014	60	40.4	IGR
SRC015	TT015	55	75.8	IGR
SRC016	TT016	54	59.7	IGR
SRC017	TT017	67	389.9	IGR
SRC018	TT018	87	112.2	5'UTR
SRC019	TT019	86	109.3	Inside
SRC020	TT020	203	261.8	IGR
SRC021	TT021	75	59.5	5'UTR
SRC022	TT022	59	75.2	5'UTR
SRC023	TT023	56	88.6	IGR
SRC024	TT024	71	61.1	5'UTR
SRC025	TT025	57	177.8	IGR
SRC026	TT026	90	108.8	5'UTR
SRC027	TT027	64	224.9	Inside
SRC028	TT028	64	109.8	IGR
SRC029	TT029	193	170.9	Inside
SRC030	TT030	78	42.7	IGR
SRC031	TT031	70	126.5	5'UTR
SRC032	TT032	53	287.8	3'UTR
SRC033	TT034	98	1235.9	5'UTR
SRC034	TT036	64	639.9	5'UTR
SRC035	TT037	102	360.4	5'UTR
SRC036	TT038	157	41.6	5'UTR
SRC037	TT039	64	1243.9	Inside
SRC038	TT040	90	1594.6	Inside
SRC039	TT041	89	109.31	Inside
SRC040	TT043	230	711.6	3'UTR
SRC041	TT044	85	377.7	5'UTR
SRC042	TT045	86	100.7	Inside
SRC043	TT046	99	393.8	5'UTR

SRC044	TT047	57	140.9	Inside
SRC045	TT048	52	159.1	IGR
SRC046	TT049	62	74.1	Inside
SRC047	TT050	59	77.1	Inside
SRC048	TT051	68	2288.1	Inside
SRC049	TT053	90	38.7	Inside
SRC050	TT054	93	29.3	Inside
SRC051	TT055	61	91.3	5’UTR
SRC052	TT056	70	158.5	5’UTR
SRC053	TT057	53	276.5	Inside
SRC054	TT058	57	627.5	IGR
SRC055	TT059	72	197.5	Inside
SRC056	TT060	78	64.1	3’UTR
SRC057	TT062	73	102.1	IGR
SRC058	TT063	60	2317.6	5’UTR
SRC059	TT064	66	105.1	5’UTR
SRC060	TT065	62	180.9	Inside
SRC061	TT066	55	112.3	Inside
SRC062	TT067	65	53.1	Inside
SRC063	TT068	91	137.8	IGR
SRC064	TT069	423	301.9	IGR
SRC065	TT070	55	76.2	Inside
SRC066	TT071	64	190.7	5’UTR
SRC067	TT072	51	88.9	Inside
SRC068	TT073	79	200.4	Inside
SRC069	TT074	93	1286.2	5’UTR
SRC070	TT077	67	107.4	5’UTR
SRC071	TT078	79	42.3	IGR
SRC072	TT079	79	71.1	5’UTR
SRC073	TT080	90	42.6	Inside
SRC074	TT081	60	146.7	IGR
SRC075	TT082	70	79.7	5’UTR
SRC076	TT083	64	64.9	Inside
SRC077	TT084	60	46.2	Inside
SRC078	TT085	72	56.2	Inside
SRC079	TT086	59	134.2	Inside
SRC080	TT087	90	421.1	IGR
SRC081	TT088	61	201.1	Inside
SRC082	TT089	74	261.9	Inside
SRC083	TT090	150	295.1	5’UTR
SRC084	TT091	54	33.4	IGR
SRC085	TT092	64	115.3	Inside
SRC086	TT093	66	299.9	IGR
SRC087	TT094	108	57.6	Inside
SRC088	TT095	60	297.1	5’UTR

SRC089	TT096	68	69.4	Inside
SRC090	TT097	90	38.4	3'UTR
SRC091	TT098	65	70.2	5'UTR
SRC092	TT099	58	80.9	Inside
SRC093	TT100	73	253.9	Inside
SRC094	TT101	56	274.8	IGR
SRC095	TT102	60	54.7	Inside
SRC096	TT103	57	155.5	Inside
SRC097	TT104	81	26.5	IGR
SRC098	TT105	84	114.6	5'UTR
SRC099	TT106	65	158.4	Inside
SRC100	TT107	97	72.7	Inside
SRC101	TT108	56	810.9	5'UTR
SRC102	TT109	90	38.1	Inside
SRC103	TT110	107	32.9	Inside
SRC104	TT111	101	576.2	5'UTR
SRC105	TT113	124	1111.6	5'UTR
SRC106	TT114	80	807.7	Inside
SRC107	TT115	93	1962.1	Inside
SRC108	TT116	90	1376.4	3'UTR
SRC109	TT118	116	771.7	3'UTR
SRC110	TT119	84	50.8	IGR
SRC111	TT120	103	304.7	5'UTR
SRC112	TT121	66	195.5	5'UTR
SRC113	TT122	82	68.4	3'UTR
SRC114	TT123	69	194.5	Inside
SRC115	TT124	60	58.1	Inside
SRC116	TT125	56	42.3	Inside
SRC117	TT126	50	102.4	Inside
SRC118	TT127	72	88.6	5'UTR
SRC119	TT128	68	162.5	Inside
SRC120	TT129	96	2433.3	IGR
SRC121	TT130	67	53.9	Inside
SRC122	TT131	88	1519.9	IGR
SRC123	TT132	215	178.2	5'UTR
SRC124	TT133	50	360.9	IGR
SRC125	TT134	116	207.8	Inside
SRC126	TT135	73	43.7	5'UTR
SRC127	TT136	89	100.5	5'UTR
SRC128	TT137	53	137.23	Inside
SRC129	TT138	68	13.1	Inside
SRC130	TT139	71	79.9	Inside
SRC131	TT140	55	52.7	IGR
SRC132	TT141	90	140.9	Inside
SRC133	TT142	94	149.1	IGR

SRC134	TT143	207	25.1	5'UTR
SRC135	TT144	63	195.9	IGR
SRC136	TT146	188	167.3	IGR
SRC137	TT147	77	438.3	Inside
SRC138	TT148	53	93.4	3'UTR
SRC139	TT149	78	11.9	5'UTR
SRC140	TT151	88	39.6	3'UTR
SRC141	TT152	56	20.2	IGR
SRC142	TT153	59	962.6	Inside
SRC143	TT154	77	147.4	Inside
SRC144	TT155	55	2095.7	IGR
SRC145	TT156	61	195.9	3'UTR
SRC146	TT157	73	155.9	5'UTR
SRC147	TT159	77	491.6	IGR
SRC148	TT160	90	99.5	IGR
SRC149	TT161	57	308.1	IGR
SRC150	TT162	69	85.2	5'UTR
SRC151	TT163	50	98.6	5'UTR
SRC152	TT164	76	128.1	3'UTR
SRC153	TT165	71	1206.7	IGR
SRC154	TT166	69	70.4	IGR
SRC155	TT167	70	100.9	Inside
SRC156	TT168	201	133.9	IGR
SRC157	TT169	60	123.5	5'UTR
SRC158	TT170	62	61.9	Inside
SRC159	TT171	59	82.9	Inside
SRC160	TT172	96	228.4	Inside
SRC161	TT173	54	96.9	Inside
SRC162	TT175	173	17.2	5'UTR
SRC163	TT176	95	194.1	5'UTR
SRC164	TT177	256	1185.6	5'UTR
SRC165	TT178	136	1567.7	IGR
SRC166	TT179	89	32.9	3'UTR
SRC167	TT180	71	29.1	IGR
SRC168	TT181	91	354.5	5'UTR
SRC169	TT182	100	150.2	3'UTR
SRC170	TT183	92	150.7	5'UTR
SRC171	TT184	210	456.5	5'UTR
SRC172	TT185	66	16.2	IGR
SRC173	TT187	360	60.6	5'UTR
SRC174	TT188	57	71.5	Inside
SRC175	TT189	68	53.7	5'UTR
SRC176	TT190	57	45.4	Inside
SRC177	TT191	94	300.9	5'UTR
SRC178	TT192	157	171.8	5'UTR

SRC179	TT194	77	5188.6	3'UTR
SRC180	TT195	90	80.3	Inside
SRC181	TT196	91	38.6	Inside
SRC182	TT197	60	596.1	IGR
SRC183	TT198	94	193.9	Inside
SRC184	TT199	86	37.8	5'UTR
SRC185	TT200	94	186.7	5'UTR
SRC186	TT201	58	584.9	Inside
SRC187	TT202	64	826.2	3'UTR
SRC188	TT203	66	14.3	Inside
SRC189	TT204	90	59.7	Inside
SRC190	TT205	60	154.9	IGR
SRC191	TT206	57	202.4	Inside
SRC192	TT207	94	56.2	5'UTR
SRC193	TT208	139	96.3	5'UTR
SRC194	TT209	90	97.8	Inside
SRC195	TT210	68	157.8	IGR
SRC196	TT211	89	214.1	Inside
SRC197	TT212	95	110.3	5'UTR
SRC198	TT213	68	72.1	IGR
SRC199	TT214	59	23.4	3'UTR
SRC200	TT215	63	1795129	5'UTR
SRC201	TT216	61	206.9	3'UTR
SRC202	TT217	65	151.2	3'UTR
SRC203	TT219	56	249.3	3'UTR
SRC204	TT220	56	211.5	5'UTR
SRC205	TT221	67	105.9	5'UTR
SRC206	TT222	65	71.7	Inside
SRC207	TT223	62	376.9	5'UTR
SRC208	TT224	79	54.9	5'UTR
SRC209	TT225	54	90.3	Inside
SRC210	TT226	70	15.2	Inside
SRC211	TT227	85	32.8	5'UTR
SRC212	TT228	200	179.9	Inside
SRC213	TT229	94	151.1	5'UTR
SRC214	TT232	91	93.1	Inside
SRC215	TT233	90	134.1	Inside
SRC216	TT234	51	152.9	Inside
SRC217	TT235	57	51.7	5'UTR
SRC218	TT238	57	297.2	5'UTR
SRC219	TT239	162	365.4	5'UTR
SRC220	TT240	94	226.1	Inside
SRC221	TT241	237	924.1	3'UTR
SRC222	TT242	191	419.5	5'UTR
SRC223	TT243	55	333.3	Inside

SRC224	TT244	69	74.6	Inside
SRC225	TT245	88	62.9	Inside
SRC226	TT247	235	639.4	5'UTR
SRC227	TT248	89	87.1	Inside
SRC228	TT249	60	193.4	Inside
SRC229	TT250	94	634.9	5'UTR
SRC230	TT253	60	56.7	3'UTR
SRC231	TT254	78	53.3	Inside
SRC232	TT255	228	146.5	Inside
SRC233	TT256	90	38.4	Inside
SRC234	TT257	95	79.6	5'UTR
SRC235	TT258	63	478.5	5'UTR
SRC236	TT259	74	302.4	5'UTR
SRC237	TT260	51	77.2	Inside
SRC238	TT261	63	18.3	Inside
SRC239	TT262	214	2615.9	5'UTR
SRC240	TT263	61	58.9	Inside
SRC241	TT264	50	9.2	Inside
SRC242	TT265	85	272.1	5'UTR
SRC243	TT267	116	72.1	5'UTR
SRC244	TT268	58	9.2	Inside
SRC245	TT270	90	82.2	5'UTR
SRC246	TT271	70	36.2	5'UTR
SRC247	TT272	70	43.6	Inside
SRC248	TT273	61	348.8	IGR
SRC249	TT274	96	240.9	IGR
SRC250	TT275	71	412.3	5'UTR
SRC251	TT276	87	48.7	Inside
SRC252	TT277	72	915.7	Inside
SRC253	TT278	91	89.2	Inside
SRC254	TT279	97	70.2	5'UTR
SRC255	TT286	69	719.7	Inside
SRC256	TT287	56	57.9	Inside
SRC257	TT288	57	389.2	IGR
SRC258	TT289	58	105.8	3'UTR
SRC259	TT290	60	87.9	Inside
SRC260	TT291	65	99.8	Inside
SRC261	TT292	69	275.8	5'UTR
SRC262	TT293	154	95.3	Inside
SRC263	TT294	56	114.9	Inside
SRC264	TT295	90	85.7	3'UTR
SRC265	TT296	62	89.1	Inside
SRC266	TT297	72	411.7	IGR
SRC267	TT298	87	164.7	Inside
SRC268	TT300	90	471.1	IGR

SRC269	TT301	98	1151.5	IGR
SRC270	TT302	75	69.3	5'UTR
SRC271	TT303	67	44.3	Inside
SRC272	TT304	311	93.3	IGR
SRC273	TT305	92	50.4	5'UTR
SRC274	TT306	72	112.4	Inside
SRC275	TT307	104	279.9	Inside
SRC276	TT308	290	390.1	Inside
SRC277	TT309	62	82.6	IGR
SRC278	TT310	97	109.1	Inside
SRC279	TT311	54	89.3	Inside
SRC280	TT312	58	286.5	5'UTR
SRC281	TT313	108	444.9	IGR
SRC282	TT314	110	33.9	Inside
SRC283	TT315	79	109.2	5'UTR
SRC284	TT317	59	157.6	Inside
SRC285	TT318	113	154.6	Inside
SRC286	TT319	90	686.3	Inside
SRC287	TT320	65	118.7	5'UTR
SRC288	TT321	85	143.8	Inside
SRC289	TT322	58	37.4	Inside
SRC290	TT323	61	133.6	3'UTR
SRC291	TT324	81	437.1	IGR
SRC292	TT325	132	37.7	5'UTR
SRC293	TT326	66	346.8	Inside
SRC294	TT327	55	122.8	Inside
SRC295	TT328	64	795.6	Inside
SRC296	TT329	63	95.3	Inside
SRC297	TT330	54	47.1	5'UTR
SRC298	TT331	72	21.6	Inside
SRC299	TT332	84	395.3	5'UTR
SRC300	TT333	79	34.9	IGR
SRC301	TT334	113	35.5	5'UTR
SRC302	TT336	76	64.2	5'UTR
SRC303	TT337	220	244.1	5'UTR
SRC304	TT338	201	255.2	3'UTR
SRC305	TT339	240	657.2	5'UTR
SRC306	TT340	248	196.1	5'UTR
SRC307	TT341	185	8230	5'UTR
SRC308	TT342	71	2944.9	IGR
SRC309	TT343	60	151.9	Inside
SRC310	TT344	55	924.9	IGR
SRC311	TT345	64	76.6	5'UTR
SRC312	TT346	65	230.1	IGR
SRC313	TT347	55	190.1	Inside

SRC314	TT349	73	51.1	Inside
SRC315	TT350	93	752.9	IGR
SRC316	TT352	92	556.7	Inside
SRC317	TT353	61	84.4	Inside
SRC318	TT354	63	709.1	5'UTR
SRC319	TT355	53	133.5	5'UTR
SRC320	TT356	53	232.1	Inside
SRC321	TT357	57	183.4	Inside
SRC322	TT358	55	305.5	Inside
SRC323	TT359	184	39.6	IGR
SRC324	TT360	56	96.2	IGR
SRC325	TT361	78	110.6	3'UTR
SRC326	TT362	110	534.3	Inside
SRC327	TT363	79	39.7	3'UTR
SRC328	TT364	71	348.1	Inside
SRC329	TT365	93	38.4	IGR
SRC330	TT366	231	203.4	5'UTR
SRC331	TT367	90	629.5	IGR
SRC332	TT368	90	161.7	5'UTR
SRC333	TT369	74	242.2	5'UTR
SRC334	TT370	88	184.6	Inside
SRC335	TT371	62	153.9	Inside
SRC336	TT372	67	171.9	Inside
SRC337	TT373	147	398	Inside
SRC338	TT375	107	191.1	IGR
SRC339	TT376	67	351.2	Inside
SRC340	TT377	81	34.4	Inside
SRC341	TT378	117	167.4	5'UTR
SRC342	TT379	55	1677.7	IGR
SRC343	TT380	107	147.1	5'UTR
SRC344	TT381	67	843.6	5'UTR
SRC345	TT383	55	86.2	3'UTR
SRC346	TT384	80	37.5	5'UTR
SRC347	TT385	262	362.8	Inside
SRC348	TT386	55	147.6	Inside
SRC349	TT387	82	319.6	Inside
SRC350	TT388	55	292.9	5'UTR
SRC351	TT390	282	709.1	Inside
SRC352	TT391	311	383.5	5'UTR
SRC353	TT392	146	424.8	3'UTR
SRC354	TT393	89	139.1	5'UTR
SRC355	TT394	90	367.5	5'UTR
SRC356	TT395	52	166.9	Inside
SRC357	TT396	96	201.1	5'UTR
SRC358	TT397	90	90.8	Inside

SRC359	TT399	68	137.1	Inside
SRC360	TT399	112	74.1	3'UTR
SRC361	TT400	84	24.9	Inside
SRC362	TT401	76	136.1	5'UTR
SRC363	TT402	78	25.8	Inside
SRC364	TT403	88	150.1	IGR
SRC365	TT404	124	446.3	5'UTR
SRC366	TT405	63	172.3	Inside
SRC367	TT406	89	150.3	5'UTR
SRC368	TT407	55	71.6	Inside
SRC369	TT408	54	51.1	Inside
SRC370	TT409	225	92.3	Inside
SRC371	TT410	76	81.5	Inside
SRC372	TT411	67	78.8	Inside
SRC373	TT412	67	130.8	3'UTR
SRC374	TT413	110	819.6	Inside
SRC375	TT414	336	287.4	Inside
SRC376	TT415	74	574.8	Inside
SRC377	TT416	85	43.2	IGR
SRC378	TT417	70	338.4	3'UTR
SRC379	TT418	60	91.1	Inside
SRC380	TT419	78	349.8	IGR
SRC381	TT420	107	105.9	IGR
SRC382	TT421	61	67.7	IGR
SRC383	TT422	95	1124.5	3'UTR
SRC384	TT423	79	125.5	Inside
SRC385	TT424	52	213.4	Inside
SRC386	TT425	80	118.2	IGR
SRC387	TT426	91	65.2	3'UTR
SRC388	TT428	68	1754.6	5'UTR
SRC389	TT429	90	72.1	5'UTR
SRC390	TT430	80	158.2	Inside
SRC391	TT431	85	71.3	Inside
SRC392	TT432	57	360.2	5'UTR
SRC393	TT433	114	100.4	Inside
SRC394	TT434	115	49.2	Inside
SRC395	TT435	71	147.9	3'UTR
SRC396	TT436	118	31.8	5'UTR
SRC397	TT437	74	834.1	5'UTR
SRC398	TT438	70	116.7	5'UTR
SRC399	TT439	90	293.9	Inside
SRC400	TT440	98	999.5	Inside
SRC401	TT441	97	1462.7	5'UTR
SRC402	TT442	116	28.6	Inside
SRC403	TT443	91	145.2	IGR

SRC404	TT444	90	168.8	5'UTR
SRC405	TT445	90	174.5	Inside
SRC406	TT446	117	75.6	5'UTR
SRC407	TT447	104	303.1	5'UTR
SRC408	TT448	61	268.6	Inside
SRC409	TT449	94	400.3	3'UTR
SRC410	TT450	78	210.6	5'UTR
SRC411	TT451	44	36.2	IGR
SRC412	TT453	116	247.7	5'UTR
SRC413	TT454	85	332.1	Inside
SRC414	TT455	59	253.9	5'UTR
SRC415	TT456	95	30.2	Inside
SRC416	TT457	90	81.6	Inside
SRC417	TT458	93	76.7	Inside
SRC418	TT459	90	126.3	5'UTR
SRC419	TT460	77	161.1	5'UTR
SRC420	TT461	58	128.5	5'UTR
SRC421	TT463	83	38.7	Inside
SRC422	TT464	74	490.7	IGR
SRC423	TT465	114	1456.1	3'UTR
SRC424	TT466	92	1404.7	Inside
SRC425	TT467	100	869.9	3'UTR
SRC426	TT468	84	136.2	Inside
SRC427	TT469	80	53.3	Inside
SRC428	TT470	71	148.2	Inside
SRC429	TT471	51	349.9	5'UTR
SRC430	TT472	80	133.3	Inside
SRC431	TT473	177	261.5	IGR
SRC432	TT474	86	77.9	5'UTR
SRC433	TT475	101	551.2	Inside
SRC434	TT476	168	1063.2	IGR
SRC435	TT477	126	100.2	Inside
SRC436	TT478	74	333.5	5'UTR
SRC437	TT479	58	181.6	IGR
SRC438	TT480	61	169.5	IGR
SRC439	TT482	90	53.6	5'UTR
SRC440	TT483	61	347.5	Inside
SRC441	TT486	263	2516.6	Inside
SRC442	TT488	109	158.6	Inside
SRC443	TT490	121	516.5	3'UTR
SRC444	TT493	92	232.5	Inside
SRC445	TT494	55	160.3	Inside
SRC446	TT495	62	321.2	Inside
SRC447	TT496	91	40.3	Inside
SRC448	TT497	92	138.1	5'UTR

SRC449	TT498	91	115.2	Inside
SRC450	TT499	61	598.8	Inside
SRC451	TT500	95	810.8	Inside
SRC452	TT501	90	953.2	5'UTR
SRC453	TT502	74	964.4	5'UTR
SRC454	TT503	128	615.9	IGR
SRC455	TT504	84	320.9	5'UTR
SRC456	TT505	58	68.4	3'UTR
SRC457	TT506	68	210.9	Inside
SRC458	TT507	82	127.5	Inside
SRC459	TT508	80	516.2	IGR
SRC460	TT509	167	234.6	Inside
SRC461	TT510	51	112.5	IGR
SRC462	TT512	91	97.5	Inside
SRC463	TT517	242	341.5	Inside
SRC464	TT518	115	62.2	5'UTR
SRC465	TT519	93	184.7	5'UTR
SRC466	TT520	64	458.5	Inside
SRC467	TT521	76	99.1	Inside
SRC468	TT522	69	97.9	Inside
SRC469	TT523	77	218.6	Inside
SRC470	TT524	132	152.7	3'UTR
SRC471	TT525	93	59.6	Inside
SRC472	TT526	87	67.5	Inside
SRC473	TT527	76	27.9	5'UTR
SRC474	TT528	66	547.8	Inside
SRC475	TT529	125	300.6	5'UTR
SRC476	TT530	113	374.3	Inside
SRC477	TT532	75	447.4	5'UTR
SRC478	TT533	59	122.6	5'UTR
SRC479	TT534	183	413.3	5'UTR
SRC480	TT535	75	50.7	IGR
SRC481	TT536	93	147.3	5''TR
SRC482	TT537	130	100.2	Inside
SRC483	TT538	71	389.2	Inside
SRC484	TT539	63	89.8	Inside
SRC485	TT540	77	211.9	Inside
SRC486	TT541	86	76.7	5'UTR
SRC487	TT542	73	46.9	Inside
SRC488	TT543	285	86.6	Inside
SRC489	TT545	186	307.1	IGR
SRC490	TT546	69	76.9	5'UTR
SRC491	TT547	61	216.2	5'UTR
SRC492	TT548	231	591.7	3'UTR
SRC493	TT549	125	1910.7	5'UTR

SRC494	TT550	62	106.3	Inside
SRC495	TT551	68	83.9	IGR
SRC496	TT552	74	340.1	Inside
SRC497	TT553	100	56.5	5'UTR
SRC498	TT554	73	487.2	5'UTR
SRC499	TT555	64	725.7	IGR
SRC500	TT556	62	167.7	Inside
SRC501	TT557	61	9.5	Inside
SRC502	TT558	296	56.3	5'UTR
SRC503	TT559	92	82.3	Inside
SRC504	TT560	54	71.5	IGR
SRC505	TT561	66	73.9	3'UTR
SRC506	TT562	69	103.8	Inside
SRC507	TT563	59	30.9	Inside
SRC508	TT564	158	527.7	5'UTR
SRC509	TT565	67	1014.4	IGR
SRC510	TT567	53	5736.2	5'UTR
SRC511	TT568	76	4608.9	3'UTR
SRC512	TT569	190	221.9	3'UTR
SRC513	TT570	97	324.4	3'UTR
SRC514	TT571	69	154.6	3'UTR
SRC515	TT572	83	104.3	IGR
SRC516	TT573	64	40.9	5'UTR
SRC517	TT574	88	173.3	3'UTR
SRC518	TT575	97	112.2	Inside
SRC519	TT576	91	802.2	IGR
SRC520	TT577	101	106.4	Inside
SRC521	TT578	90	126.1	5'UTR
SRC522	TT579	92	322.8	IGR
SRC523	TT580	167	437.6	5'UTR
SRC524	TT581	91	43.3	Inside
SRC525	TT582	90	66.1	Inside
SRC526	TT583	74	51.1	3'UTR
SRC527	TT584	75	791.9	5'UTR
SRC528	TT585	77	314.9	Inside
SRC529	TT586	94	708.2	Inside
SRC530	TT587	59	174.4	5'UTR
SRC531	TT588	68	106.1	5'UTR
SRC532	TT589	71	30.6	5'UTR
SRC533	TT590	101	54.9	Inside
SRC534	TT591	263	227.3	3'UTR
SRC535	TT592	80	559.8	Inside
SRC536	TT593	71	80.8	Inside
SRC537	TT594	90	104.5	5'UTR
SRC538	TT595	115	118.6	Inside

SRC539	TT596	114	98.9	5’UTR
SRC540	TT597	61	94.9	IGR
SRC541	TT598	102	305.6	IGR
SRC542	TT599	92	332.6	Inside
SRC543	TT600	70	52.8	5’UTR
SRC544	TT601	72	113.5	Inside
SRC545	TT602	94	330.7	Inside
SRC546	TT603	90	381.5	Inside
SRC547	TT604	52	159.3	5’UTR
SRC548	TT605	54	500.3	IGR
SRC549	TT606	107	329.1	5’UTR
SRC550	TT607	57	716.5	IGR
SRC551	TT608	87	407.9	5’UTR
SRC552	TT609	55	14.2	Inside
SRC553	TT610	91	311.9	5’UTR
SRC554	TT611	67	92.3	Inside
SRC555	TT612	52	114.4	5’UTR
SRC556	TT613	62	108.5	5’UTR
SRC557	TT614	139	231.9	3’UTR
SRC558	TT615	79	139.4	5’UTR
SRC559	TT616	189	118.4	5’UTR
SRC560	TT618	59	545.9	Inside
SRC561	TT619	55	478.1	3’UTR
SRC562	TT620	57	179.1	3’UTR
SRC563	TT621	56	22.5	Inside
SRC564	TT622	165	1307.8	IGR
SRC565	TT623	65	2699.8	IGR
SRC566	TT624	87	3640.8	IGR
SRC567	TT625	95	99.1	5’UTR
SRC568	TT626	77	29.6	Inside
SRC569	TT627	65	474.6	3’UTR
SRC570	TT628	121	47.4	IGR
SRC571	TT629	110	24.1	5’UTR
SRC572	TT631	105	4811.5	5’UTR
SRC573	TT632	69	174.8	Inside
SRC574	TT633	160	160.6	Inside
SRC575	TT635	61	996.8	5’UTR
SRC576	TT636	86	484.3	IGR
SRC577	TT637	97	321.3	5’UTR
SRC578	TT638	92	297.7	Inside
SRC579	TT639	70	81.5	5’UTR
SRC580	TT640	91	88.4	Inside
SRC581	TT641	91	270.1	5’UTR
SRC582	TT642	72	262.8	3’UTR
SRC583	TT643	59	189.4	5’UTR

SRC584	TT644	90	156.9	IGR
SRC585	TT645	56	26.5	Inside
SRC586	TT647	85	920.7	Inside
SRC587	TT648	94	3275.1	5’UTR
SRC588	TT649	90	81.6	Inside
SRC589	TT650	92	790.3	IGR
SRC590	TT651	61	150.3	5’UTR
SRC591	TT652	84	112.9	Inside
SRC592	TT653	92	17436.3	IGR
SRC593	TT654	65	154.2	IGR
SRC594	TT655	53	89.5	3’UTR
SRC595	TT658	174	18.3	5’UTR
SRC596	TT660	174	699.7	Inside
SRC597	TT661	90	67.1	Inside
SRC598	TT662	92	64.1	Inside
SRC599	TT663	215	161.8	Inside
SRC600	TT664	67	130.4	5’UTR
SRC601	TT665	80	13.1	5’UTR
SRC602	TT666	56	101.5	Inside
SRC603	TT667	272	1546.5	IGR
SRC604	TT668	93	4130.9	IGR
SRC605	TT669	123	74.7	5’UTR
SRC606	TT670	104	93.1	Inside
SRC607	TT671	66	46.3	Inside
SRC608	TT672	90	577.7	Inside
SRC609	TT673	107	110.9	IGR
SRC610	TT674	61	137.9	5’UTR
SRC611	TT675	73	109.8	IGR
SRC612	TT676	194	33.3	3’UTR

#RPKM represents reads per kilo bases per million reads. “5’UTR”, “3’UTR”, “IGR” and “Inside” represent the SRC is located in an ORF’s 5’-untranslated region, 3’-untranslated region, intergenic region and inside an ORF coding region, respectively. TT, target transcript.
