# Supplementary material for: Genome‐wide screen and functional analysis in Xanthomonas reveal a large number of mRNA‐derived sRNAs, including the novel RsmA‐sequester RsmU
Source: Mol Plant Pathol. 2020 Sep 23;21(12):1573–90. doi: 10.1111/mpp.12997 (PMC7694677; doi:10.1111/mpp.12997)
Supplement: Supplementary file 17 — TABLE S5 The identified seven putative riboswitches [file MPP-21-1573-s017.pdf]

Table S5. The identified 7 putative riboswitches[#]

Riboswitch name	SRC name	TT name	Length (bp)	Overlap gene	Upstream gene	Downstream gene
TPP (RF00059)	SRC123	TT132	215	XC0846	XC0845	XC0847
Cobalamin (RF00174)	SRC156	TT168	201	Null	XC1090	XC1091
SAM (RF00162)	SRC171	TT184	210	XC1251	XC1250	XC1252
Glycine (RF00504)	SRC431	TT473	177	Null	XC3134	XC3135
SAH (FR01057)	SRC479	TT534	183	XC3482	XC3481	XC3483
FMN (FR0050)	SRC489	TT545	186	Null	XC3539	XC3540
yybP-ykoY (FR0080)	SRC611	TT675	73	Null	XC4320	XC4321

[#]SRC, sRNA candidate; TT, target transcript.
