# Supplementary material for: Genome‐wide screen and functional analysis in Xanthomonas reveal a large number of mRNA‐derived sRNAs, including the novel RsmA‐sequester RsmU
Source: Mol Plant Pathol. 2020 Sep 23;21(12):1573–90. doi: 10.1111/mpp.12997 (PMC7694677; doi:10.1111/mpp.12997)
Supplement: Supplementary file 18 — TABLE S6 A summary of the 117 detectable sRNAs from 121 SRCs by northern blotting [file MPP-21-1573-s018.pdf]

Table S6. A summary of the 117 detectable sRNAs from 121 SRCs by Northern blotting*

sRNA name	SRC name	TT name	Start	End	Length (bp)	RPKM value	Detected by Northern blotting
sRX001	SRC001	TT001	1	115	115	148.1	Yes
sRX002	SRC002	TT002	3605	3701	97	263.6	Yes
sRX003	SRC003	TT003	8254	8318	65	201	Yes
sRX004	SRC005	TT005	11540	11606	67	100.3	Yes
sRX005	SRC007	TT007	19739	19801	63	45.7	Yes
sRX006	SRC008	TT008	20127	20185	59	80.5	Yes
sRX007	SRC009	TT009	44065	44146	82	212.9	Yes
sRX008	SRC011	TT011	73643	73693	51	117.4	Yes
sRX009	SRC017	TT017	83883	83949	67	389.9	Yes
sRX010	SRC022	TT022	142449	142507	59	75.2	Yes
sRX011	SRC024	TT024	165210	165280	71	61.1	Yes
sRX012	SRC028	TT028	225413	225476	64	109.8	Yes
sRX013	SRC029	TT029	230567	230759	193	170.9	Yes
sRX014	SRC030	TT030	239805	239882	78	42.7	Yes
sRX015	SRC035	TT037	292444	292545	102	360.4	Yes
sRX016	SRC037	TT039	328623	328686	64	1243.9	Yes
sRX017	SRC042	TT045	346558	346643	86	100.7	Yes
sRX018	SRC048	TT051	395405	395472	68	2288.1	Yes
sRX019	SRC052	TT056	419488	419557	70	158.5	Yes
sRX020	SRC053	TT057	447714	447766	53	276.5	Yes
sRX021	SRC058	TT063	480582	480641	60	2317.6	Yes
sRX022	SRC059	TT064	486775	486840	66	105.1	Yes
	SRC065	TT070	526278	526332	55	76.2	Not
sRX023	SRC067	TT072	574674	574724	51	88.9	Yes
sRX024	SRC069	TT074	578891	578983	93	1286.2	Yes
sRX025	SRC072	TT079	621291	621369	79	71.1	Yes
sRX026	SRC073	TT080	624944	625033	90	42.6	Yes
sRX027	SRC080	TT087	677097	677186	90	421.1	Yes
sRX028	SRC082	TT089	704129	704202	74	261.9	Yes
sRX029	SRC083	TT090	704921	705070	150	295.1	Yes
sRX030	SRC086	TT093	766606	766671	66	299.9	Yes
sRX031	SRC089	TT096	781908	781975	68	69.4	Yes
sRX032	SRC093	TT100	830789	830861	73	253.9	Yes
sRX033	SRC094	TT101	848204	848259	56	274.8	Yes
	SRC096	TT103	849532	849588	57	155.5	Not
sRX034	SRC098	TT105	853703	853786	84	114.6	Yes
sRX035	SRC101	TT108	857025	857080	56	810.9	Yes
sRX036	SRC102	TT109	859744	859833	90	38.1	Yes
sRX037	SRC104	TT111	862234	862334	101	576.2	Yes
sRX038	SRC112	TT121	923871	923936	66	195.5	Yes
	SRC115	TT124	971506	971565	60	58.1	Not
sRX039	SRC118	TT127	998027	998098	72	88.6	Yes

sRX040	SRC119	TT128	1009676	1009743	68	162.5	Yes
sRX041	SRC120	TT129	1013277	1013372	96	2433.3	Yes
sRX042	SRC121	TT130	1014431	1014497	67	53.9	Yes
sRX043	SRC122	TT131	1019341	1019428	88	1519.9	Yes
sRX044	SRC123	TT132	1019825	1020040	215	178.2	Yes
sRX045	SRC124	TT133	1022098	1022147	50	360.9	Yes
sRX046	SRC128	TT137	1035293	1035345	53	137.23	Yes
sRX047	SRC133	TT142	1052567	1052660	94	149.1	Yes
sRX048	SRC134	TT143	1074880	1075087	207	25.1	Yes
sRX049	SRC137	TT147	1097403	1097479	77	438.3	Yes
sRX050	SRC138	TT148	1108108	1108160	53	93.4	Yes
sRX051	SRC147	TT159	1231053	1231129	77	491.6	Yes
sRX052	SRC153	TT165	1269031	1269101	71	1206.7	Yes
sRX053	SRC154	TT166	1295653	1295721	69	70.4	Yes
	SRC155	TT167	1296858	1296927	70	100.9	Not
sRX054	SRC156	TT168	1316918	1317118	201	133.9	Yes
sRX055	SRC160	TT172	1381928	1382023	96	228.4	Yes
sRX056	SRC163	TT176	1412286	1412380	95	194.1	Yes
sRX057	SRC170	TT183	1511287	1511378	92	150.7	Yes
sRX058	SRC171	TT184	1531053	1531262	210	456.5	Yes
sRX059	SRC175	TT189	1594827	1594894	68	53.7	Yes
sRX060	SRC177	TT191	1600577	1600670	94	300.9	Yes
sRX061	SRC179	TT194	1618954	1619030	77	5188.6	Yes
sRX062	SRC183	TT198	1634567	1634660	94	193.9	Yes
sRX063	SRC184	TT199	1638354	1638439	86	37.8	Yes
sRX064	SRC190	TT205	1671801	1671860	60	154.9	Yes
sRX065	SRC192	TT207	1690686	1690779	94	56.2	Yes
sRX066	SRC195	TT210	1747942	1748009	68	157.8	Yes
sRX067	SRC196	TT211	1750038	1750126	89	214.1	Yes
sRX068	SRC197	TT212	1751067	1751161	95	110.3	Yes
sRX069	SRC198	TT213	1791899	1791966	68	72.1	Yes
sRX070	SRC203	TT219	1807102	1807157	56	249.3	Yes
sRX071	SRC205	TT221	1814332	1814398	67	105.9	Yes
sRX072	SRC207	TT223	1843442	1843503	62	376.9	Yes
sRX073	SRC208	TT224	1844170	1844248	79	54.9	Yes
sRX074	SRC213	TT229	1896637	1896730	94	151.1	Yes
sRX075	SRC218	TT238	1955793	1955849	57	297.2	Yes
sRX076	SRC220	TT240	1964190	1964283	94	226.1	Yes
sRX077	SRC222	TT242	1987898	1988088	191	419.5	Yes
sRX078	SRC229	TT250	2018414	2018507	94	634.9	Yes
sRX079	SRC231	TT254	2117629	2117706	78	53.3	Yes
sRX080	SRC235	TT258	2170287	2170349	63	478.5	Yes
sRX081	SRC239	TT262	2221405	2221619	214	2615.9	Yes
sRX082	SRC240	TT263	2223778	2223838	61	58.9	Yes
sRX083	SRC245	TT270	2281622	2281711	90	82.2	Yes

sRX084	SRC249	TT274	2303046	2303141	96	240.9	Yes
sRX085	SRC250	TT275	2334041	2334111	71	412.3	Yes
sRX086	SRC251	TT276	2334327	2334413	87	48.7	Yes
sRX087	SRC252	TT277	2358598	2358674	72	915.7	Yes
sRX088	SRC255	TT286	2418445	2418513	69	719.7	Yes
sRX089	SRC257	TT288	2428848	2428904	57	389.2	Yes
sRX090	SRC262	TT293	2443942	2444096	154	95.3	Yes
sRX091	SRC263	TT294	2455991	2456046	56	114.9	Yes
sRX092	SRC264	TT295	2458026	2458115	90	85.7	Yes
sRX093	SRC265	TT296	2509851	2509912	62	89.1	Yes
sRX094	SRC266	TT297	2513401	2513472	72	411.7	Yes
sRX095	SRC271	TT303	2551037	2551103	67	44.3	Yes
sRX096	SRC272	TT304	2566429	2566739	311	93.3	Yes
sRX097	SRC281	TT313	2656886	2656937	108	444.9	Yes
sRX098	SRC283	TT315	2669660	2669738	79	109.2	Yes
sRX099	SRC286	TT319	2675595	2675684	90	686.3	Yes
sRX100	SRC288	TT321	2686474	2686558	85	143.8	Yes
sRX101	SRC289	TT322	2687002	2687059	58	37.4	Yes
sRX102	SRC291	TT324	2696762	2696842	81	437.1	Yes
sRX103	SRC293	TT326	2708703	2708768	66	346.8	Yes
sRX104	SRC295	TT328	2711448	2711512	64	795.6	Yes
sRX105	SRC299	TT332	2760980	2761064	84	395.3	Yes
sRX106	SRC303	TT337	2919083	2919303	220	244.1	Yes
sRX107	SRC307	TT341	2939567	2939752	185	8230	Yes
sRX108	SRC309	TT343	2940875	2940934	60	151.9	Yes
sRX109	SRC310	TT344	2944151	2944205	55	924.9	Yes
sRX110	SRC312	TT346	2972679	2972743	65	230.1	Yes
sRX111	SRC315	TT350	3028347	3028440	93	752.9	Yes
sRX112	SRC318	TT354	3038922	3038984	63	709.1	Yes
sRX113	SRC319	TT355	3039995	3040047	53	133.5	Yes
sRX114	SRC326	TT362	3066078	3066187	110	534.3	Yes
sRX115	SRC341	TT378	3188220	3188336	117	167.4	Yes
sRX116	SRC342	TT379	3188900	3188954	55	1677.7	Yes
sRX117	SRC344	TT381	3245880	3245946	67	843.6	Yes

*SRC, sRNA candidate. TT, target transcript. RPKM, reads per kilo bases per million reads.
