# Supplementary material for: Genome‐wide screen and functional analysis in Xanthomonas reveal a large number of mRNA‐derived sRNAs, including the novel RsmA‐sequester RsmU
Source: Mol Plant Pathol. 2020 Sep 23;21(12):1573–90. doi: 10.1111/mpp.12997 (PMC7694677; doi:10.1111/mpp.12997)
Supplement: Supplementary file 19 — TABLE S7 Primers and DNA oligonucleotides used in this work [file MPP-21-1573-s019.pdf]

Table S7. Primers and DNA Oligos used in this work

Primers or Oligos	Sequence (5' to 3')*	Purpose
**Primer sets**		
NP-Xcc5S-F/<br>NP-Xcc5S-R	TAATACGACTCACTATAGGGCGAGTTCGGGATGGGATCGGG/<br>ACGTCACAAGTTAACTATGCGAG	Used for PCR amplification of the DNA templates for Northern RNA probe preparations.
NP-SRC001F-F/<br>NP-SRC001F-R	TAATACGACTCACTATAGGGTTTTCTGCGCGCCGCAGCC/<br>CGGGGCCAAGCATCC	
NP-SRC001R-F/<br>NP-SRC001R-R	TAATACGACTCACTATAGGGCGGGGCCAAGCATCCATC/<br>TTTTCTGCGCGCCGCAGCC	
NP-SRC002F-F/<br>NP-SRC002F-R	TAATACGACTCACTATAGGGCGGACTTCAAGGCCAAGCAGACC/<br>GCTGGCTGGATGAAACTC	
NP-SRC002R-F/<br>NP-SRC002R-R	TAATACGACTCACTATAGGGGCTGGCTGGATGAAACTCGAC/<br>CGGACTTCAAGGCCAAG	
NP-SRC003F-F/<br>NP-SRC003F-R	TAATACGACTCACTATAGGGGGGGGATTGAATGAAAGCCAGG/<br>TGGCGCACGCCGTCAAC	
NP-SRC003R-F/<br>NP-SRC003R-R	TAATACGACTCACTATAGGGTGGCGCACGCCGTCAACGCGAGG/<br>GGGGGATTGAATGAAAGCC	
NP-SRC005F-F/<br>NP-SRC005F-R	TAATACGACTCACTATAGGGCGCGTCACCACCGCGTTCTGGG/<br>GCTGCTCTTCCATAGCGC	
NP-SRC005R-F/<br>NP-SRC005R-R	TAATACGACTCACTATAGGGGCTGCTCTTCCATAGCGCGGATGG/<br>CGCGTCACCACCGCGTTC	
NP-SRC007F-F/<br>NP-SRC007F-R	TAATACGACTCACTATAGGGGTCGGGGTCTGGTCATACGCC/<br>CGACTACCGCATGAGCGAG	
NP-SRC007R-F/<br>NP-SRC007R-R	TAATACGACTCACTATAGGGCGACTACCGCATGAGCGAGACGC/<br>GTCGGGGTCTGGTCATAC	
NP-SRC008F-F/<br>NP-SRC008F-R	TAATACGACTCACTATAGGGTGTGGGTGTTCTCATCGATG/<br>CAGCACCGAAGTGGGCTTC	
NP-SRC008R-F/<br>NP-SRC008R-R	TAATACGACTCACTATAGGGCAGCACCGAAGTGGGCTTCACCC/<br>TGTGGGTGTTCTCATCG	
NP-SRC009F-F/<br>NP-SRC009F-R	TAATACGACTCACTATAGGGGCGGGGGGCCATGTCTGCCTC/<br>CTCCTGCACCGCTCCATGG	
NP-SRC009R-F/<br>NP-SRC009R-R	TAATACGACTCACTATAGGGCTCCTGCACCGCTCCATGGTCGAG/<br>GCGGGGGGCCATGTCTGCC	
NP-SRC011F-F/<br>NP-SRC011F-R	TAATACGACTCACTATAGGGGTCATGGATCTATTGATCGCC/<br>GCGCGCTCTGATGGG	
NP-SRC011R-F/<br>NP-SRC011R-R	TAATACGACTCACTATAGGGGCGCGCTCTGATGGGAAGCC/<br>GTCATGGATCTATTGATC	
NP-SRC017F-F/<br>NP-SRC017F-R	TAATACGACTCACTATAGGGTGCCGGGAGGCTAGAAACCGC/<br>GCGGCGAATACAACACCC	
NP-SRC017R-F/<br>NP-SRC017R-R	TAATACGACTCACTATAGGGGCGGCGAATACAACACCCGCAAGG/<br>TGCCGGGAGGCTAGAAACC	
NP-SRC022F-F/<br>NP-SRC022F-R	TAATACGACTCACTATAGGGGTCCACGTTGATCATCCATCCC/<br>GGGGATGCGTGGTGGTG	
NP-SRC022R-F/<br>NP-SRC022R-R	TAATACGACTCACTATAGGGGGGGATGCGTGGTGGTGCAGATG/<br>GTCCACGTTGATCATCC	
NP-SRC024F-F/<br>NP-SRC024F-R	TAATACGACTCACTATAGGGATCTTGATGTCGTCTGCAGCATC/<br>GGGCTTCAGAACTTCCATG	
NP-SRC024R-F/<br>NP-SRC024R-R	TAATACGACTCACTATAGGGGGGCTTCAGAACTTCCATGTCC/<br>ATCTTGATGTCGTCTGC	
NP-SRC028F-F/<br>NP-SRC028F-R	TAATACGACTCACTATAGGGGCGGGGGATGAAAGTGCAGCGG/<br>CCCAGGTTCCAGCCACCC	
NP-SRC028R-F/<br>NP-SRC028R-R	TAATACGACTCACTATAGGGCCCAGGTTCCAGCCACCCTTTCCGGG/<br>GCGGGGGATGAAAGTG	
NP-SRC029F-F/<br>NP-SRC029F-R	TAATACGACTCACTATAGGGGTACGGTCCTGCGTGGACGTTACGG/<br>GGCGCGGTGACGTTCGG	

NP-SRC029R-F/<br>NP-SRC029R-R	**TAATACGACTCACTATAGGG**GGCGCGGTGACGTTCGGTTTGCGAC/<br>GTACGGTCCTGCGTGGACG	
NP-SRC030F-F/<br>NP-SRC030F-R	**TAATACGACTCACTATAGGG**CTCAGCCGCAGCGCGTGATC/<br>TGGTCGGTGAGCTGCGCGG	
NP-SRC030R-F/<br>NP-SRC030R-R	**TAATACGACTCACTATAGGG**TGGTCGGTGAGCTGCGCGGC/<br>CTCAGCCGCAGCGCGTG	
NP-SRC037F-F/<br>NP-SRC037F-R	**TAATACGACTCACTATAGGG**GCTGGGCGCATTGTCGAGGGG/<br>CCGCAGTTTGATCTTTCATCGG	
NP-SRC037R-F/<br>NP-SRC037R-R	**TAATACGACTCACTATAGGG**CCGCAGTTTGATCTTTCATCGGC<br>GCTGGGCGCATTGTCGAG	
NP-SRC042F-F/<br>NP-SRC042F-R	**TAATACGACTCACTATAGGG**CGCCCGTGCAGGCCGATGG/<br>GAGCCGTTCTTCCGCTATC	
NP-SRC042R-F/<br>NP-SRC042R-R	**TAATACGACTCACTATAGGG**GAGCCGTTCTTCCGCTATCTGCACG/<br>CGCCCGTGCAGGCCG	
NP-SRC048F-F/<br>NP-SRC048F-R	**TAATACGACTCACTATAGGG**CACGCTCAAGCAACGCATCGACG/<br>CCTGATTCTTGTCACCGG	
NP-SRC048R-F/<br>NP-SRC048R-R	**TAATACGACTCACTATAGGG**CCTGATTCTTGTCACCGGCGCC/<br>CACGCTCAAGCAACGC	
NP-SRC052F-F/<br>NP-SRC052F-R	**TAATACGACTCACTATAGGG**TGCGGGGTGTGTTCCAGAAACGGG/<br>GTTGGCGGCACGCTAACTTG	
NP-SRC052R-F/<br>NP-SRC052R-R	**TAATACGACTCACTATAGGG**GTTGGCGGCACGCTAACTTGCCGG/<br>TGCGGGGTGTGTTCCAG	
NP-SRC053F-F/<br>NP-SRC053F-R	**TAATACGACTCACTATAGGG**CGCTGATCGACGATGGCGTC/<br>GCCGCTTTTCAGTGG	
NP-SRC053R-F/<br>NP-SRC053R-R	**TAATACGACTCACTATAGGG**GCCGCTTTTCAGTGGTCGCAACAC/<br>CGCTGATCGACGATGG	
NP-SRC058F-F/<br>NP-SRC058F-R	**TAATACGACTCACTATAGGG**TGTCGGTCTGGATCTGGATCTG/<br>GGCAGACGCAGCGG	
NP-SRC058R-F/<br>NP-SRC058R-R	**TAATACGACTCACTATAGGG**GGCAGACGCAGCGGATGACC/<br>TGTCGGTCTGGATCTGG	
NP-SRC059F-F/<br>NP-SRC059F-R	**TAATACGACTCACTATAGGG**GGGGAAAGAGGCGAGCACGC/<br>CACATTTCGCGTGAAAGCC	
NP-SRC059R-F/<br>NP-SRC059R-R	**TAATACGACTCACTATAGGG**CACATTTCGCGTGAAAGCCAAGG/<br>GGGGAAAGAGGCGAG	
NP-SRC065F-F/<br>NP-SRC065F-R	**TAATACGACTCACTATAGGG**GCGGGGTCGTGTTCCAGCTCCC/<br>CCCTGATCGAAGCCATC	
NP-SRC065R-F/<br>NP-SRC065R-R	**TAATACGACTCACTATAGGG**CCCTGATCGAAGCCATCACCC/<br>GCGGGGTCGTGTTCC	
NP-SRC067F-F/<br>NP-SRC067F-R	**TAATACGACTCACTATAGGG**TCCCGCCCCGCGCGAGGAC/<br>GTGTGATCGAAGTCGAAG	
NP-SRC067R-F/<br>NP-SRC067R-R	**TAATACGACTCACTATAGGG**GTGTGATCGAAGTCGAAGACC/<br>TCCCGCCCCGCGCG	
NP-SRC069F-F/<br>NP-SRC069F-R	**TAATACGACTCACTATAGGG**CCTTCACAACGCGTGGCAAAG/<br>TGGCGGTGAACGTAGTC	
NP-SRC069R-F/<br>NP-SRC069R-R	**TAATACGACTCACTATAGGG**TGGCGGTGAACGTAGTCATGAG/<br>CCTTCACAACGCGTGG	
NP-SRC072F-F/<br>NP-SRC072F-R	**TAATACGACTCACTATAGGG**GGGACGTTCCCGCGCCAGGCC/<br>GCGGGAGCGAGCAAC	
NP-SRC072R-F/<br>NP-SRC072R-R	**TAATACGACTCACTATAGGG**GCGGGAGCGAGCAACAGCCAGAG/<br>GGGACGTTCCCGCGCC	
NP-SRC073F-F/<br>NP-SRC073F-R	**TAATACGACTCACTATAGGG**GTCAGTCGGTAGATCATTTCGAC/<br>GGAGAATCGGGAATCGG	
NP-SRC073R-F/<br>NP-SRC073R-R	**TAATACGACTCACTATAGGG**GGAGAATCGGGAATCGGCACAGCGG/<br>GTCAGTCGGTAGATC	
NP-SRC080F-F/<br>NP-SRC080F-R	**TAATACGACTCACTATAGGG**ACGCGATATGCCGGTGTTCTC/<br>CTGGGATCAAAAACGCTTGG	
NP-SRC080R-F/<br>NP-SRC080R-R	**TAATACGACTCACTATAGGG**CTGGGATCAAAAACGCTTGG/<br>ACGCGATATGCCGGTG	

NP-SRC082F-F/<br>NP-SRC082F-R	TAATACGACTCACTATAGGGTGTGCGTAGCTACGACCCGACC/<br>CCATCATCGCCTTCATGCC	
NP-SRC082R-F/<br>NP-SRC082R-R	TAATACGACTCACTATAGGGCCATCATCGCCTTCATGCCGTGCTG/<br>TGTGCGTAGCTACGACCC	
NP-SRC083F-F/<br>NP-SRC083F-R	TAATACGACTCACTATAGGGGGTTCATGTCGTTCGTCGTCGTATTC/<br>CGGGGGCATGGCTCATC	
NP-SRC083R-F/<br>NP-SRC083R-R	TAATACGACTCACTATAGGGCGGGGGCATGGCTCATCAACTTAAC/<br>GGTTCATGTCGTTCGTC	
NP-SRC086F-F/<br>NP-SRC086F-R	TAATACGACTCACTATAGGGCCCAAGGGCGCTCCTACGAGG/<br>CGCTGCGTAGCAGGTTG	
NP-SRC086R-F/<br>NP-SRC086R-R	TAATACGACTCACTATAGGGCGCTGCGTAGCAGGTTGATGATC/<br>CCCAAGGGCGCTCC	
NP-SRC089F-F/<br>NP-SRC089F-R	TAATACGACTCACTATAGGGGTGAGGGGGAATCACTTCAG/<br>CGGCGATCAGGACCCGC	
NP-SRC089R-F/<br>NP-SRC089R-R	TAATACGACTCACTATAGGGCGGCGATCAGGACCCGCACTTG/<br>GTGAGGGGGAATCAC	
NP-SRC093F-F/<br>NP-SRC093F-R	TAATACGACTCACTATAGGGGTGCTCGAAGCGCTGCACGAG/<br>GCGGGGTCTGCTCCAG	
NP-SRC093R-F/<br>NP-SRC093R-R	TAATACGACTCACTATAGGGGCGGGGTCTGCTCCAGCGCC/<br>GTGCTCGAAGCGCTG	
NP-SRC094F-F/<br>NP-SRC094F-R	TAATACGACTCACTATAGGGGGAAGCCGGTCGCACCTGG/<br>GCGTGCCGCCTCTGG	
NP-SRC094R-F/<br>NP-SRC094R-R	TAATACGACTCACTATAGGGGCGTGCCGCCTCTGGCATCACC/<br>GGAAGCCGGTCGCACC	
NP-SRC096F-F/<br>NP-SRC096F-R	TAATACGACTCACTATAGGGTGCGATGGCCGTGTTCCAGCTG/<br>GCCGGGGGTAACTCC	
NP-SRC096R-F/<br>NP-SRC096R-R	TAATACGACTCACTATAGGGGCCGGGGGTAACTCCTTGCC/<br>TGCGATGGCCGTGTTCC	
NP-SRC098F-F/<br>NP-SRC098F-R	TAATACGACTCACTATAGGGGCCCCAAGGAACGGCGGCC/<br>GTTCCCCTAACACCTG	
NP-SRC098R-F/<br>NP-SRC098R-R	TAATACGACTCACTATAGGGGTTCCCCTAACACCTGCGCC/<br>GCCCCAAGGAACGG	
NP-SRC101F-F/<br>NP-SRC101F-R	TAATACGACTCACTATAGGGGGATGACAACGCCAGCAAC/<br>GGCGGTGATCGGATG	
NP-SRC101R-F/<br>NP-SRC101R-R	TAATACGACTCACTATAGGGGGCGGTGATCGGATGTG/<br>GGATGACAACGCC	
NP-SRC102F-F/<br>NP-SRC102F-R	TAATACGACTCACTATAGGGGGCCGGTCAATGGGCTGGCCC/<br>TGTTGGTGGCTTATGCGTTG	
NP-SRC102R-F/<br>NP-SRC102R-R	TAATACGACTCACTATAGGGTGTTGGTGGCTTATGCGTTGCTGGGG/<br>GGCCGGTCAATGGG	
NP-SRC104F-F/<br>NP-SRC104F-R	TAATACGACTCACTATAGGGGGACAGACCACTGGCTGACC/<br>GCCGTTATGAAAGGG	
NP-SRC104R-F/<br>NP-SRC104R-R	TAATACGACTCACTATAGGGGCCGTTATGAAAGGGCTCGAAAC/<br>GGACAGACCACTGGCTG	
NP-SRC112F-F/<br>NP-SRC112F-R	TAATACGACTCACTATAGGGCAGGGGGCGATCCCCACATCGTCCC/<br>GCTGGGACTCGACAAGGG	
NP-SRC112R-F/<br>NP-SRC112R-R	TAATACGACTCACTATAGGGGCTGGGACTCGACAAGGGCAC/<br>CAGGGGGCGATCCCC	
NP-SRC115F-F/<br>NP-SRC115F-R	TAATACGACTCACTATAGGGGTACTGCAGGCCGGCGAACTCC/<br>GCCGGCAAGTTCTACGGG	
NP-SRC115R-F/<br>NP-SRC115R-R	TAATACGACTCACTATAGGGGCCGGCAAGTTCTACGGGTTCAAC/<br>GTACTGCAGGCCGGCG	
NP-SRC118F-F/<br>NP-SRC118F-R	TAATACGACTCACTATAGGGTGCAACACCCTGTTGACGGG/<br>GTGCGCATGCGCCCC	
NP-SRC118R-F/<br>NP-SRC118R-R	TAATACGACTCACTATAGGGGTGCGCATGCGCCCCGACCCC/<br>TGCAACACCCTGTTGAC	
NP-SRC119F-F/<br>NP-SRC119F-R	TAATACGACTCACTATAGGGGGGCGCTCATCGCCGCGCTCC/<br>GTGCGCATGCGCCCC	

NP-SRC119R-F/<br>NP-SRC119R-R	**TAATACGACTCACTATAGGG**GTGCGCATGCGCCCCGACCCC/<br>GGGCGCTCATCGCCGCGC	
NP-SRC120F-F/<br>NP-SRC120F-R	**TAATACGACTCACTATAGGG**TCCCGCACCGGTTGGCGAGG/<br>CGCACCGCCACCTTGAAC	
NP-SRC120R-F/<br>NP-SRC120R-R	**TAATACGACTCACTATAGGG**CGCACCGCCACCTTGAACGCTTCAC/<br>TCCCGCACCGGTTGGCGAGG	
NP-SRC121F-F/<br>NP-SRC121F-R	**TAATACGACTCACTATAGGG**GTCGAGCCGGTCAGCACC/<br>TGCCCTGGCTGTTGGG	
NP-SRC121R-F/<br>NP-SRC121R-R	**TAATACGACTCACTATAGGG**TGCCCTGGCTGTTGGGCGGCAG/<br>GTCGAGCCGGTCAGCACC	
NP-SRC122F-F/<br>NP-SRC122F-R	**TAATACGACTCACTATAGGG**GCGGCAGGGAGAGAGAGCTGG/<br>TCGTCGGCATCCACCGG	
NP-SRC122R-F/<br>NP-SRC122R-R	**TAATACGACTCACTATAGGG**TCGTCGGCATCCACCGGCATTTG/<br>GCGGCAGGGAGAGAGAG	
NP-SRC123F-F/<br>NP-SRC123F-R	**TAATACGACTCACTATAGGG**GGGTACCACAGCACTGCTGCGG/<br>GGGCGCGGCATTCATTGG	
NP-SRC123R-F/<br>NP-SRC123R-R	**TAATACGACTCACTATAGGG**GGGCGCGGCATTCATTGGCGTTC/<br>GGGTACCACAGCACTG	
NP-SRC124F-F/<br>NP-SRC124F-R	**TAATACGACTCACTATAGGG**GTCGTAATTGTTTGAATTC/<br>CGGCGACGTCAGG	
NP-SRC124R-F/<br>NP-SRC124R-R	**TAATACGACTCACTATAGGG**GCGGCGACGTCAGGCTCTC/<br>GTCGTAATTGTTTG	
NP-SRC128F-F/<br>NP-SRC128F-R	**TAATACGACTCACTATAGGG**CGGCGCTGGCGCTTTTCTTG/<br>CCGGGGAGTGACGC	
NP-SRC128R-F/<br>NP-SRC128R-R	**TAATACGACTCACTATAGGG**CCGGGGAGTGACGCAATACC/<br>CGGCGCTGGCGC	
NP-SRC133F-F/<br>NP-SRC133F-R	**TAATACGACTCACTATAGGG**GGCGGTAATGGCGCCAGTAATG/<br>GCACCCCTTTGGTCCTAC	
NP-SRC133R-F/<br>NP-SRC133R-R	**TAATACGACTCACTATAGGG**GCACCCCTTTGGTCCTACTCCCC/<br>GGCGGTAATGGCGCC	
NP-SRC134F-F/<br>NP-SRC134F-R	**TAATACGACTCACTATAGGG**GGGGGAGGTCGCGCGACGGTG/<br>GGATGGGCATGGGTGGGG	
NP-SRC134R-F/<br>NP-SRC134R-R	**TAATACGACTCACTATAGGG**GGATGGGCATGGGTGGGGACCC/<br>GGGGGAGGTCGCGCGACGG	
NP-SRC137F-F/<br>NP-SRC137F-R	**TAATACGACTCACTATAGGG**TCGGCCGTACGCGGCGCAC/<br>TGTAATCGCACGGCGATC	
NP-SRC137R-F/<br>NP-SRC137R-R	**TAATACGACTCACTATAGGG**TGTAATCGCACGGCGATCAGTGAG/<br>TCGGCCGTACGCGGCGC	
NP-SRC138F-F/<br>NP-SRC138F-R	**TAATACGACTCACTATAGGG**CGTCGATCAGAACGTGTAG/<br>GGGGCGCATCGCGC	
NP-SRC138R-F/<br>NP-SRC138R-R	**TAATACGACTCACTATAGGG**GGGGCGCATCGCGCAACGG/<br>CGTCGATCAGAACGTG	
NP-SRC147F-F/<br>NP-SRC147F-R	**TAATACGACTCACTATAGGG**CCGGTACAAATCGGTACGTTTTG/<br>TGGCCTGCTCTTCCAGCAAC	
NP-SRC147R-F/<br>NP-SRC147R-R	**TAATACGACTCACTATAGGG**TGGCCTGCTCTTCCAGCAACAATGCGG<br>/CCGGTACAAATCGGTAC	
NP-SRC153F-F/<br>NP-SRC153F-R	**TAATACGACTCACTATAGGG**TGTCGGGAGGTTAGAAACC/<br>GGGCGGCTAATACAAC	
NP-SRC153R-F/<br>NP-SRC153R-R	**TAATACGACTCACTATAGGG**GGGCGGCTAATACAACACTCC/<br>TGTCGGGAGGTTAG	
NP-SRC154F-F/<br>NP-SRC154F-R	**TAATACGACTCACTATAGGG**GGGAGAGAGGCAGCGGACTGG/<br>GGACCCTGGGAGGGG	
NP-SRC154R-F/<br>NP-SRC154R-R	**TAATACGACTCACTATAGGG**GGACCCTGGGAGGGGCGG/<br>GGGAGAGAGGCAGCGG	
NP-SRC155F-F/<br>NP-SRC155F-R	**TAATACGACTCACTATAGGG**TGGCTGAAGTTCTGGTTGAC/<br>GCCACATCAACGGTGG	
NP-SRC155R-F/<br>NP-SRC155R-R	**TAATACGACTCACTATAGGG**GCCACATCAACGGTGGTCATG/<br>TGGCTGAAGTTCTGG	

NP-SRC156F-F/<br>NP-SRC156F-R	TAATACGACTCACTATAGGGTGACTGCCGGAATTCCGGTGG/<br>CGGGTCAATCCCTCAGG	
NP-SRC156R-F/<br>NP-SRC156R-R	TAATACGACTCACTATAGGGCGGGTCAATCCCTCAGGCCGGTC/<br>TGACTGCCGGAATTCC	
NP-SRC160F-F/<br>NP-SRC160F-R	TAATACGACTCACTATAGGGCATCGACGTCCTCAAGCAGG/<br>GGGGGAAAGCGTCAG	
NP-SRC160R-F/<br>NP-SRC160R-R	TAATACGACTCACTATAGGGGGGGGAAAGCGTCAGCGTGCATC/<br>CATCGACGTCCTCAAG	
NP-SRC163F-F/<br>NP-SRC163F-R	TAATACGACTCACTATAGGGGTGGGAGCGGGGACAACGGG/<br>GGCATCGGCCGATCC	
NP-SRC163R-F/<br>NP-SRC163R-R	TAATACGACTCACTATAGGGGGCATCGGCCGATCCAGGCGTCCC/<br>GTGGGAGCGGGGAC	
NP-SRC170F-F/<br>NP-SRC170F-R	TAATACGACTCACTATAGGGAAGCGCCAGAGGCAGCAATGCCCC/<br>CAGGCGGGAACACC	
NP-SRC170R-F/<br>NP-SRC170R-R	TAATACGACTCACTATAGGGCAGGCGGGAACACCATGACAG/<br>AAGCGCCAGAGGCAG	
NP-SRC171F-F/<br>NP-SRC171F-R	TAATACGACTCACTATAGGGGGCGGAGGGACAGGCCCTTTGATG/<br>GTGAGTGGCGATGCTGTGG	
NP-SRC171R-F/<br>NP-SRC171R-R	TAATACGACTCACTATAGGGGTGAGTGGCGATGCTGTGGTCACGAGG<br>/GGCGGAGGGACAGGCCC	
NP-SRC175F-F/<br>NP-SRC175F-R	TAATACGACTCACTATAGGGTGGCGGACGGCTTCGGCCATG/<br>GCACCGCGGCCTCTGAAG	
NP-SRC175R-F/<br>NP-SRC175R-R	TAATACGACTCACTATAGGGGCACCGCGGCCTCTGAAGAG/<br>TGGCGGACGGCTTCGGCC	
NP-SRC177F-F/<br>NP-SRC177F-R	TAATACGACTCACTATAGGGCGGTGGGCCAATGAACCTTTCC/<br>CACGAACGCGTGCGCATC	
NP-SRC177R-F/<br>NP-SRC177R-R	TAATACGACTCACTATAGGGCACGAACGCGTGCGCATCGCC/<br>CGGTGGGCCAATGAACC	
NP-SRC179F-F/<br>NP-SRC179F-R	TAATACGACTCACTATAGGGGACGCGAGGATGCGTGGCC/<br>GGCGATTTCACGTCC	
NP-SRC179R-F/<br>NP-SRC179R-R	TAATACGACTCACTATAGGGGGCGATTTCACGTCCTTGTCGG/<br>GACGCGAGGATGCGTGG	
NP-SRC183F-F/<br>NP-SRC183F-R	TAATACGACTCACTATAGGGGCCGGCGTTGATGCGGAAGTAGG/<br>CTCGGCCGTATTTTCC	
NP-SRC183R-F/<br>NP-SRC183R-R	TAATACGACTCACTATAGGGCTCGGCCGTATTTTCCGATGG/<br>GCCGGCGTTGATGCGG	
NP-SRC184F-F/<br>NP-SRC184F-R	TAATACGACTCACTATAGGGTGGCGGCCTGTGGTTCGGG<br>GGCGATGCGCCAACTG/	
NP-SRC184R-F/<br>NP-SRC184R-R	TAATACGACTCACTATAGGGGGCGATGCGCCAACTGATCAGTG/<br>TGGCGGCCTGTGG	
NP-SRC190F-F/<br>NP-SRC190F-R	TAATACGACTCACTATAGGGTGCCGCCACTGCTTGCTTG/<br>ATCGGCTCTGCGTCC	
NP-SRC190R-F/<br>NP-SRC190R-R	TAATACGACTCACTATAGGGATCGGCTCTGCGTCCTGG/<br>TGCCGCCACTGCTTG	
NP-SRC192F-F/<br>NP-SRC192F-R	TAATACGACTCACTATAGGGCCCGGCGATGGATGACAATGCGGGG/<br>GCGCGTTGCGTGGACGG	
NP-SRC192R-F/<br>NP-SRC192R-R	TAATACGACTCACTATAGGGGCGCGTTGCGTGGACGGCACGCTC/<br>CCCGGCGATGGATGAC	
NP-SRC195F-F/<br>NP-SRC195F-R	TAATACGACTCACTATAGGGGCTGGTCCTCGATCGGCTCCCC/<br>TGCCGGTGCCTTCGCATC	
NP-SRC195R-F/<br>NP-SRC195R-R	TAATACGACTCACTATAGGGTGCCGGTGCCTTCGCATCGCC/<br>GCTGGTCCTCGATCGG	
NP-SRC196F-F/<br>NP-SRC196F-R	TAATACGACTCACTATAGGGGAACGACAACTCGCCGCTGTCCC/<br>GCGTGCGCGACCCACAG	
NP-SRC196R-F/<br>NP-SRC196R-R	TAATACGACTCACTATAGGGGCGTGCGCGACCCACAGTTTTCC/<br>GAACGACAACTCGCC	
NP-SRC197F-F/<br>NP-SRC197F-R	TAATACGACTCACTATAGGGGCTGGTCCTCGATCGGCTCCCC/<br>TGCCGGTGCCTTCGCATC	

NP-SRC197R-F/<br>NP-SRC197R-R	TAATACGACTCACTATAGGGTGCCGGTGCCTTCGCATCGCC/<br>GCTGGTCCTCGATCGG	
NP-SRC198F-F/<br>NP-SRC198F-R	TAATACGACTCACTATAGGGTGGACCATGCACGACACCGGC/<br>GGGGTTTCGGTGTG	
NP-SRC198R-F/<br>NP-SRC198R-R	TAATACGACTCACTATAGGGGGGGTTTCGGTGTGCAGATC/<br>TGGACCATGCACGACACC	
NP-SRC203F-F/<br>NP-SRC203F-R	TAATACGACTCACTATAGGGGTTGCCTGCTTTGACCAGCC/<br>GCGGGTTCGTAATGG	
NP-SRC203R-F/<br>NP-SRC203R-R	TAATACGACTCACTATAGGGGCGGGTTCGTAATGGAGG/<br>GTTGCCTGCTTTGACC	
NP-SRC205F-F/<br>NP-SRC205F-R	TAATACGACTCACTATAGGGGGATGATCGTGCGTACGGGG<br>GCGGGGAATCGGG/	
NP-SRC205R-F/<br>NP-SRC205R-R	TAATACGACTCACTATAGGGGCGGGGAATCGGGAGTCGGG/<br>GGATGATCGTGCGTAC	
NP-SRC207F-F/<br>NP-SRC207F-R	TAATACGACTCACTATAGGGGGCGGATGCGGTGCGGCGCC/<br>AATTGTGATTCCCGCG	
NP-SRC207R-F/<br>NP-SRC207R-R	TAATACGACTCACTATAGGGAATTGTGATTCCCGCGTGAC/<br>GGCGGATGCGGTG	
NP-SRC208F-F/<br>NP-SRC208F-R	TAATACGACTCACTATAGGGCCATGATGGCGGCGCACTC/<br>TGCCTGGCTCTGAATGGGG	
NP-SRC208R-F/<br>NP-SRC208R-R	TAATACGACTCACTATAGGGTGCCTGGCTCTGAATGGGG/<br>CCATGATGGCGGCGCAC	
NP-SRC213F-F/<br>NP-SRC213F-R	TAATACGACTCACTATAGGGGGCCTGCACGGCCGCC/<br>GCGGGGGTGTTCTCGG	
NP-SRC213R-F/<br>NP-SRC213R-R	TAATACGACTCACTATAGGGGCGGGGGTGTTCTCGGTGCGG/<br>GGCCTGCACGGCCGCC	
NP-SRC218F-F/<br>NP-SRC218F-R	TAATACGACTCACTATAGGGCGGAAAGGAAGACTGGCGATGG/<br>GCCCCCTCTGGTCAGCACGG	
NP-SRC218R-F/<br>NP-SRC218R-R	TAATACGACTCACTATAGGGGCCCCCTCTGGTCAGCACGG/<br>CGGAAAGGAAGACTGG	
NP-SRC220F-F/<br>NP-SRC220F-R	TAATACGACTCACTATAGGGGGGCTGCGCTGGCTATTTC/<br>CGGGGTGCTTTCG	
NP-SRC220R-F/<br>NP-SRC220R-R	TAATACGACTCACTATAGGGCGGGGTGCTTTCGATATTC/<br>GGGCTGCGCTGG	
NP-SRC222F-F/<br>NP-SRC222F-R	TAATACGACTCACTATAGGGCGGCAACGGGCATACTGCGCGG/<br>GTCTTGATCTTGGGCATTG	
NP-SRC222R-F/<br>NP-SRC222R-R	TAATACGACTCACTATAGGGGTCTTGATCTTGGGCATTGCTATGTCC/<br>CGGCAACGGGCATACTG	
NP-SRC229F-F/<br>NP-SRC229F-R	TAATACGACTCACTATAGGGTGGGCAACGGCGGGGATG/<br>GAGACAGACACGCAC	
NP-SRC229R-F/<br>NP-SRC229R-R	TAATACGACTCACTATAGGGGAGACAGACACGCACGTAGG/<br>TGGGCAACGGCGGGG	
NP-SRC231F-F/<br>NP-SRC231F-R	TAATACGACTCACTATAGGGGGCGGGGCGATCGTGGGG/<br>GGCGATCCTGATCTC	
NP-SRC231R-F/<br>NP-SRC231R-R	TAATACGACTCACTATAGGGGGCGATCCTGATCTCGCTCGCGGG/<br>GGCGGGGCGATCGTGG	
NP-SRC235F-F/<br>NP-SRC235F-R	TAATACGACTCACTATAGGGTCCGGGGATTCTGAGGTTTGG/<br>CCGGGTGCCGTTCCCC	
NP-SRC235R-F/<br>NP-SRC235R-R	TAATACGACTCACTATAGGGCCGGGTGCCGTTCCCCGTCAAG/<br>TCCGGGGATTCTGAGG	
NP-SRC239F-F/<br>NP-SRC239F-R	TAATACGACTCACTATAGGGGCAAGGGTCACCGTGAATCGTGG/<br>GTGATGAAGCCGAAGCCC	
NP-SRC239R-F/<br>NP-SRC239R-R	TAATACGACTCACTATAGGGGTGATGAAGCCGAAGCCCTTGG/<br>GCAAGGGTCACCGTG	
NP-SRC240F-F/<br>NP-SRC240F-R	TAATACGACTCACTATAGGGTCAATTCGATAACCGTTTGCGCGCC/<br>GCGGCGTGGGCCTTCTTCC	
NP-SRC240R-F/<br>NP-SRC240R-R	TAATACGACTCACTATAGGGGCGGCGTGGGCCTTCTTCCGGG/<br>TCAATTCGATAACCGTTTG	

NP-SRC245F-F/<br>NP-SRC245F-R	**TAATACGACTCACTATAGGG**GTGGGATGGCGACTTAC/<br>GCGGGCGTTTCGCTGG	
NP-SRC245R-F/<br>NP-SRC245R-R	**TAATACGACTCACTATAGGG**GCGGGCGTTTCGCTGGCTGG/<br>GTGGGATGGCGAC	
NP-SRC249F-F/<br>NP-SRC249F-R	**TAATACGACTCACTATAGGG**GCGGGGAGGTCGTCTAC/<br>CGCAACCGGACGCAG	
NP-SRC249R-F/<br>NP-SRC249R-R	**TAATACGACTCACTATAGGG**CGCAACCGGACGCAGCGGCCC/<br>GCGGGGAGGTCGTC	
NP-SRC250F-F/<br>NP-SRC250F-R	**TAATACGACTCACTATAGGG**CTCGGGTCCGCCGATGCTCC/<br>GCGCGGGGACGAGCGAG	
NP-SRC250R-F/<br>NP-SRC250R-R	**TAATACGACTCACTATAGGG**GCGCGGGGACGAGCGAGAC/<br>CTCGGGTCCGCCGATG	
NP-SRC251F-F/<br>NP-SRC251F-R	**TAATACGACTCACTATAGGG**GGCGTGATCACCGAGCC/<br>GCCGGAGATGTTGCG	
NP-SRC251R-F/<br>NP-SRC251R-R	**TAATACGACTCACTATAGGG**GCCGGAGATGTTGCGCGCG/<br>GGCGTGATCACCG	
NP-SRC252F-F/<br>NP-SRC252F-R	**TAATACGACTCACTATAGGG**GACCTCGAAAGCGAACGCTTGCC/<br>TCCGGGTCAATGG	
NP-SRC252R-F/<br>NP-SRC252R-R	**TAATACGACTCACTATAGGG**TCCGGGTCAATGGTCAGCCC/<br>GACCTCGAAAGCGAAC	
NP-SRC255F-F/<br>NP-SRC255F-R	**TAATACGACTCACTATAGGG**TGCGGCCGTGGATGATAATG/<br>GCCTGTTGGCGTTTATCC	
NP-SRC255R-F/<br>NP-SRC255R-R	**TAATACGACTCACTATAGGG**GCCTGTTGGCGTTTATCCTTCGG/<br>TGCGGCCGTGGATG	
NP-SRC257F-F/<br>NP-SRC257F-R	**TAATACGACTCACTATAGGG**CCTCAATAGCACATAAGG/<br>GCCGGGGGTGCGG	
NP-SRC257R-F/<br>NP-SRC257R-R	**TAATACGACTCACTATAGGG**GCCGGGGGTGCGGAGATG/<br>CCTCAATAGCACATAAGG	
NP-SRC262F-F/<br>NP-SRC262F-R	**TAATACGACTCACTATAGGG**ATGGGCTACAGCAGGCCGCGCTC/<br>CCCTCGGGGGCCACTGAACC	
NP-SRC262R-F/<br>NP-SRC262R-R	**TAATACGACTCACTATAGGG**CCCTCGGGGGCCACTGAACC/<br>ATGGGCTACAGCAGGCC	
NP-SRC263F-F/<br>NP-SRC263F-R	**TAATACGACTCACTATAGGG**TCGAATCGCGCCGTAGG/<br>GTCCGGGCATGGG	
NP-SRC263R-F/<br>NP-SRC263R-R	**TAATACGACTCACTATAGGG**GTCCGGGCATGGGCGTCGG/<br>TCGAATCGCGCCGTAGG	
NP-SRC264F-F/<br>NP-SRC264F-R	**TAATACGACTCACTATAGGG**CATGGTCTCTGCGTCAGGG/<br>GGCGCTTGCAGGG	
NP-SRC264R-F/<br>NP-SRC264R-R	**TAATACGACTCACTATAGGG**GGCGCTTGCAGGGCATCGAAGG/<br>CATGGTCTCTGCGTCAGG	
NP-SRC265F-F/<br>NP-SRC265F-R	**TAATACGACTCACTATAGGG**GCGCGGACTGTCCACACAG/<br>TGTGGATCGGCCAGGG	
NP-SRC265R-F/<br>NP-SRC265R-R	**TAATACGACTCACTATAGGG**TGTGGATCGGCCAGGGCAGCGCC/<br>GCGCGGACTGTCCACAC	
NP-SRC266F-F/<br>NP-SRC266F-R	**TAATACGACTCACTATAGGG**GGCGAAGAAAGTAATTGGG/<br>CTTGAATTTGGAGCGGG	
NP-SRC266R-F/<br>NP-SRC266R-R	**TAATACGACTCACTATAGGG**CTTGAATTTGGAGCGGGAAAC/<br>GGCGAAGAAAGTAATTGGG	
NP-SRC271F-F/<br>NP-SRC271F-R	**TAATACGACTCACTATAGGG**TCCGGCTTCGAATGGCAGG/<br>GGTGGGGCTGCGTCAAT	
NP-SRC271R-F/<br>NP-SRC271R-R	**TAATACGACTCACTATAGGG**GGTGGGGCTGCGTCAATC/<br>TCCGGCTTCGAATGG	
NP-SRC272F-F/<br>NP-SRC272F-R	**TAATACGACTCACTATAGGG**GCAGTGCAACGCATTCACGG/<br>GCACGTCGCATTTGAG	
NP-SRC272R-F/<br>NP-SRC272R-R	**TAATACGACTCACTATAGGG**GCACGTCGCATTTGAGTTTTCC/<br>GCAGTGCAACGCATTC	
NP-SRC281F-F/<br>NP-SRC281F-R	**TAATACGACTCACTATAGGG**TGGCGTTCCTCCGCGC/<br>GCGTCATCAGTAGCG	

NP-SRC281R-F/<br>NP-SRC281R-R	TAATACGACTCACTATAGGGGCGTCATCAGTAGCGATCGC/<br>TGGCGTTCCTCCGCGC	
NP-SRC283F-F/<br>NP-SRC283F-R	TAATACGACTCACTATAGGGGCGCCGCCTTGATTCACCGGG/<br>GGCAGGGTTGGCGCGG	
NP-SRC283R-F/<br>NP-SRC283R-R	TAATACGACTCACTATAGGGGGCAGGGTTGGCGCGGTG/<br>GCGCCGCCTTGATTCACC	
NP-SRC286F-F/<br>NP-SRC286F-R	TAATACGACTCACTATAGGGGCAGGTGTTCGACGGTCATG/<br>TGTTCAGCAAAGACC	
NP-SRC286R-F/<br>NP-SRC286R-R	TAATACGACTCACTATAGGGTGTTCAGCAAAGACCTAGAG/<br>GCAGGTGTTCGACGG	
NP-SRC288F-F/<br>NP-SRC288F-R	TAATACGACTCACTATAGGGGGCACGCGCGTGACGTTGCGCC/<br>GGCTCCACATGAATCAAC	
NP-SRC288R-F/<br>NP-SRC288R-R	TAATACGACTCACTATAGGGGGCTCCACATGAATCAACGTCC/<br>GGCACGCGCGTGACG	
NP-SRC289F-F/<br>NP-SRC289F-R	TAATACGACTCACTATAGGGGAAGCCAGCTACCAGGCC/<br>TGCGGGTGTACAGG	
NP-SRC289R-F/<br>NP-SRC289R-R	TAATACGACTCACTATAGGGTGCGGGTGTACAGGCGCACC/<br>GAAGCCAGCTACC	
NP-SRC291F-F/<br>NP-SRC291F-R	TAATACGACTCACTATAGGGTGCCGCGTAAGGTCCC/<br>TGGCGGCTTTGTTG	
NP-SRC291R-F/<br>NP-SRC291R-R	TAATACGACTCACTATAGGGTGGCGGCTTTGTTGATAGCC/<br>TGCCGCGTAAGGTCCC	
NP-SRC293F-F/<br>NP-SRC293F-R	TAATACGACTCACTATAGGGCCGGTCTGCAGGACGCCC/<br>GGGCGTCGGCGATCTG	
NP-SRC293R-F/<br>NP-SRC293R-R	TAATACGACTCACTATAGGGGGGCGTCGGCGATCTGACGG/<br>CCGGTCTGCAGGACGCCC	
NP-SRC295F-F/<br>NP-SRC295F-R	TAATACGACTCACTATAGGGTATCCAGGAAGTCATGCC/<br>GCGGGACTCACTCATTCC	
NP-SRC295R-F/<br>NP-SRC295R-R	TAATACGACTCACTATAGGGGCGGGACTCACTCATTCCTGG/<br>TATCCAGGAAGTCATGCC	
NP-SRC299F-F/<br>NP-SRC299F-R	TAATACGACTCACTATAGGGATTGCGTCGGCTTGCACC/<br>CGAAACTCCTCACAC	
NP-SRC299R-F/<br>NP-SRC299R-R	TAATACGACTCACTATAGGGCGAAACTCCTCACACAGCGG/<br>ATTGCGTCGGCTTGCACC	
NP-SRC303F-F/<br>NP-SRC303F-R	TAATACGACTCACTATAGGGGGACGAGGGAAACGCATC/<br>GCGCCAATGCCAACGCC	
NP-SRC303R-F/<br>NP-SRC303R-R	TAATACGACTCACTATAGGGGCGCCAATGCCAACGCCAGATC/<br>GGACGAGGGAAACGC	
NP-SRC307F-F/<br>NP-SRC307F-R	TAATACGACTCACTATAGGGGCGCCAGTTTAGATCTCTAG/<br>GGACGCCAGTCTGAC	
NP-SRC307R-F/<br>NP-SRC307R-R	TAATACGACTCACTATAGGGGGACGCCAGTCTGACATCTAC/<br>GCGCCAGTTTAGATCTC	
NP-SRC309F-F/<br>NP-SRC309F-R	TAATACGACTCACTATAGGGTGGACAAGATATCCAACAG/<br>GGGGGCTCCACTC	
NP-SRC309R-F/<br>NP-SRC309R-R	TAATACGACTCACTATAGGGGGGGGCTCCACTCACGATGG/<br>TGGACAAGATATCC	
NP-SRC310F-F/<br>NP-SRC310F-R	TAATACGACTCACTATAGGGGATGTCTGTTCCGATCC/<br>CCCGGGGCCTGTTTC	
NP-SRC310R-F/<br>NP-SRC310R-R	TAATACGACTCACTATAGGGCCCGGGGCCTGTTTCTAAGG/<br>GATGTCTGTTCCGATCC	
NP-SRC312F-F/<br>NP-SRC312F-R	TAATACGACTCACTATAGGGGGGCGCCACGCGCTCGTTG/<br>GTCGGGTGCGCTGTGCC	
NP-SRC312R-F/<br>NP-SRC312R-R	TAATACGACTCACTATAGGGGTCGGGTGCGCTGTGCCTACAGGG/<br>GGGCGCCACGCGCTCG	
NP-SRC315F-F/<br>NP-SRC315F-R	AATACGACTCACTATAGGGTCTCCGGGCGCACCATCTTCAG/<br>TGCAGATCCGCCGCAATG	
NP-SRC315R-F/<br>NP-SRC315R-R	TAATACGACTCACTATAGGGTGCAGATCCGCCGCAATGCGATC/<br>TCTCCGGGCGCACCATC	

NP-SRC318F-F/ NP-SRC318F-R	**TAATACGACTCACTATAGGG**CCGCCCTCCTGGCTTTCGATAG/ CGGGGACGGCGGAGTCAG	
NP-SRC318R-F/ NP-SRC318R-R	**TAATACGACTCACTATAGGG**CGGGGACGGCGGAGTCAGTGCC/ CCGCCCTCCTGGCTTTC	
NP-SRC319F-F/ NP-SRC319F-R	**TAATACGACTCACTATAGGG**GCGGCGGAAAGGGCGCGCTTC/ CTGACGAGGATCACCC	
NP-SRC319R-F/ NP-SRC319R-R	**TAATACGACTCACTATAGGG**CTGACGAGGATCACCCAGATGG/ GCGGCGGAAAGGGCGCGC	
NP-SRC326F-F/ NP-SRC326F-R	**TAATACGACTCACTATAGGG**TGGCCGGCAGTGCTGCAACC/ ACCAAACGTCCCCACC	
NP-SRC326R-F/ NP-SRC326R-R	**TAATACGACTCACTATAGGG**ACCAAACGTCCCCACCTTAGG/ TGGCCGGCAGTGCTGCAACC	
NP-SRC341F-F/ NP-SRC341F-R	**TAATACGACTCACTATAGGG**TGCAGGACCGGCACTAGGG/ GCGCCTGGAACCCATGGG	
NP-SRC341R-F/ NP-SRC341R-R	**TAATACGACTCACTATAGGG**GCGCCTGGAACCCATGGGCC/ GCAGGACCGGCACTAGGG	
NP-SRC342F-F/ NP-SRC342F-R	**TAATACGACTCACTATAGGG**GTCCTGCAACACGAAGTCC/ CCGCCAGTCCGAAGCC	
NP-SRC342R-F/ NP-SRC342R-R	**TAATACGACTCACTATAGGG**CCGCCAGTCCGAAGCCGTG/ GTCCTGCAACACGAAG	
NP-SRC344F-F/ NP-SRC344F-R	**TAATACGACTCACTATAGGG**GGGTGGTGGCACAGGACGGG/ GTTCGCTATCGCGGCCGC	
NP-SRC344R-F/ NP-SRC344R-R	**TAATACGACTCACTATAGGG**GTTCGCTATCGCGGCCGCTATCC/ GGGTGGTGGCACAGGACGGG	
NP-sRX061L-F/ NP-sRX061L-R	**TAATACGACTCACTATAGGG**GAAAAACGCCCGGCTGCACATCC/ GACGCGAGGATGCGTGGCC	
NP-1332-F/ NP-1332-R	**TAATACGACTCACTATAGGG**TTAACGCCGTCCCACCGG/ ATGAAGTCATTTACCGTAGTCC	
OEsRX006F/ OEsRX006R	CCCGAATTCCAGCACCGAAGTGGG/ CCCAAGCTTTGTGGGTGTTCTCATC	Used for sRNA over-expression strain construction.
OEsRX009F/ OEsRX009R	CCCGAATTCGCGGCGAATACAAC/ CCCAAGCTTTGCCGGGAGGCTAG	
OEsRX010F/ OEsRX010R	CCCGAATTCGTCCACGTTGATCATCC/ CCCAAGCTTGGGGATGCGTGGTGGTG	
OEsRX014F/ OEsRX014R	CCCGAATTCTGGTCGGTGAGCTGC/ CCCAAGCTTCTCAGCCGCAGCGCG	
OEsRX015F/ OEsRX015R	CCCGAATTCCCGCAGTTTGATCTTTC/ CCCAAGCTTGCTGGGCGCATTGTC	
OEsRX017F/ OEsRX017R	CCCGAATTCGAGCCGTTCTTCCGC/ CCCAAGCTTCGCCCGTGCAGGCCG	
OEsRX018F/ OEsRX018R	CCCGAATTCCCTGATTCTTGTCACC/ CCCAAGCTTCACGCTCAAGCAACG	
OEsRX020F/ OEsRX020R	CCCGAATTCCGCTGATCGACGATG/ CCCAAGCTTGCCGCTTTTCAGTGG	
OEsRX023F/ OEsRX023R	CCCGAATTCTCCCGCCCCGCGCG/ CCCAAGCTTGTGTGATCGAAGTCG	
OEsRX024F/ OEsRX024R	CCCGAATTCCCTTCACAACGCGTGG/ CCCAAGCTTTGGCGGTGAACGTAG	
OEsRX027F/ OEsRX027R	CCCGAATTCCTGGGATCAAAAACGC/ CCCAAGCTTACGCGATATGCCGGTG	
OEsRX028F/ OEsRX028R	CCCGAATTCTGTGCGTAGCTACGACCC/ CCCAAGCTTCCATCATCGCCTTCATG	
OEsRX030F/ OEsRX030R	CCCGAATTCCGCTGCGTAGCAGGTTG/ CCCAAGCTTCCCAAGGGCGCTCCTAC	
OEsRX031F/ OEsRX031R	CCCGAATTCGTGAGGGGGAATCACTTC/ CCCAAGCTTCGGCGATCAGGACCC	
OEsRX032F/ OEsRX032R	CCCGAATTCGCGGGGTCTGCTCCAGCGCC/ CCCAAGCTTGTGCTCGAAGCGCTG	

OEsRX039F/<br>OEsRX039R	CCCGAATTCGTGCGCATGCGCCCC/<br>CCCAAGCTTTGCAACACCCTGTTG	
OEsRX046F/<br>OEsRX046R	CCCGAATTCCGGCGCTGGCGCTTTTC/<br>CCCAAGCTTCCGGGGAGTGACGCAATACC	
OEsRX047F/<br>OEsRX047R	CCCGAATTCGCACCCCTTTGGTCCTAC/<br>CCCAAGCTTGGCGGTAATGGCGCC	
OEsRX049F/<br>OEsRX049R	CCCGAATTCTGTAATCGCACGGCG/<br>CCCAAGCTTTCGGCCGTACGCGG	
OEsRX051F/<br>OEsRX051R	CCCGAATTCCCGGTACAAATCGG/<br>CCCAAGCTTTGGCCTGCTCTTCCAG	
OEsRX052F/<br>OEsRX052R	CCCGAATTCGGGCGGCTAATACAACAC/<br>CCCAAGCTTTGTCGGGAGGTTAG	
OEsRX054F/<br>OEsRX054R	CCCGGATCCTGACTGCCGGAATTCC/<br>CCCAAGCTTCGGGTCAATCCCTCAGG	
OEsRX056F/<br>OEsRX056R	CCCGAATTCGGCATCGGCCGATCC/<br>CCCAAGCTTGTGGGAGCGGGGAC	
OEsRX061SF/<br>OEsRX061SR	CCCGAATTCGACGCGAGGATGCGTG/<br>CCCAAGCTTGGCGATTTCACGTCC	
OEsRX061LF/<br>OEsRX061LR	CCCGAATTCGACGCGAGGATGCGTG/<br>CCCAAGCTTGAAAAACGCCCGGC	
OEsRX061LMF/<br><br>OEsRX061LMR	CCCGAATTCGACGCGAAAATGCGTGGCCGCAACCTAACTGGACAAAATGTC<br>TGGGAAATGGCTAAAAGCCGACAAGGACGTG/<br>CCCAAGCTTGAAAAACGCCCGGCTGCACATTTAAGTGCAACACC	
OEsRX062F/<br>OEsRX062R	CCCGAATTCCTCGGCCGTATTTTCC/<br>CCCAAGCTTGCCGGCGTTGATGCGG	
OEsRX063F/<br>OEsRX063R	CCCGAATTCGGCGATGCGCCAACTG/<br>CCCAAGCTTTGGCGGCCTGTGGTTC	
OEsRX064F/<br>OEsRX064R	CCCGAATTCATCGGCTCTGCGTCC/<br>CCCAAGCTTTGCCGCCACTGCTTG	
OEsRX067F/<br>OEsRX067R	CCCGAATTCGAACGACAACTCGCC/<br>CCCAAGCTTGCGTGCGCGACCCAC	
OEsRX070F/<br>OEsRX070R	CCCGAATTCGTTGCCTGCTTTGACC/<br>CCCAAGCTTGCGGGTTCGTAATGG	
OEsRX071F/<br>OEsRX071R	CCCGAATTCGGATGATCGTGCGTAC/<br>CCCAAGCTTGCGGGGAATCGGGAG	
OEsRX072F/<br>OEsRX072R	CCCGAATTCAATTGTGATTCCCGCG/<br>CCCAAGCTTGGCGGATGCGGTGCGG	
OEsRX074F/<br>OEsRX074R	CCCGAATTCGGCCTGCACGGCCGCC/<br>CCCAAGCTTGCGGGGGTGTTCTCGG	
OEsRX075F/<br>OEsRX075R	CCCGAATTCCGGAAAGGAAGACTGG/<br>CCCAAGCTTGCCCCCTCTGGTCAG	
OEsRX077F/<br>OEsRX077R	CCCGAATTCCGGCAACGGGCATAC/<br>CCCAAGCTTGTCTTGATCTTGGG	
OEsRX079F/<br>OEsRX079R	CCCGAATTCGGCGGGGCGATCGTGG/<br>CCCAAGCTTGGCGATCCTGATCTC	
OEsRX080F/<br>OEsRX080R	CCCGAATTCCCGGGTGCCGTTCCCC/<br>CCCAAGCTTTCCGGGGATTCTGAGG	
OEsRX081F/<br>OEsRX081R	CCCGAATTCGCAAGGGTCACCGTG/<br>CCCAAGCTTGTGATGAAGCCGAAG	
OEsRX083F/<br>OEsRX083R	CCCGAATTCGCGGGCGTTTCGCTGG/<br>CCCAAGCTTGTGGGATGGCGAC	
OEsRX084F/<br>OEsRX084R	CCCGAATTCCGCAACCGGACGCAG/<br>CCCAAGCTTGCGGGGAGGTCGTC	
OEsRX086F/<br>OEsRX086R	CCCGAATTCGGCGTGATCACCGAG/<br>CCCAAGCTTGCCGGAGATGTTGCG	
OEsRX088F/<br>OEsRX088R	CCCGAATTCGCCTGTTGGCGTTTATCC/<br>CCCAAGCTTCATCCACGGCCGCA	
OEsRX095F/<br>OEsRX095R	CCCGAATTCGGTGGGGCTGCGTC/<br>CCCAAGCTTTCCGGCTTCGAATGG	

OEsRX102F/<br>OEsRX102R	CCCGAATTCTGGCGGCTTTGTTG/<br>CCCAAGCTTTGCCGCGTAAGGTC	
OEsRX116F/<br>OEsRX116R	CCCGAATTCGTCCTGCAACACGAAG/<br>CCCAAGCTTCCGCCAGTCCGAAGCC	
DP1332L-F/<br>DP1332L-R	CCCGAATTCGGTCAGGTTGCCGCCGGTCAGG/<br>GGCTGCGGCGTAGAATGTGCC	Used for construction of the deletion mutant of *XC1332* promoter.
DP1332R-F/<br>DP1332R-R	GGCACATTCTACGCCGCAGCCCGATAGGATTGCTGCATGTGG/<br>CCCAAGCTTACCGGATCCGGCACTACAGCC	
DP1332con-F/<br>DP1332con-R	ACGTGTCGCTGGCGAATTGG<br>ATGGTCCTAGCCAGCATTCC	
D3UTRL-F/<br>D3UTR L-R	CCCGAATTCCATGCCGTCGATGGCCTGGG/<br>TTAACGCCGTCCCACCGGTTTCACC	Used for construction of the deletion mutant of *XC1332* 3'UTR.
D3UTRR-F/<br>D3UTRR-R	CCGGTGGGACGGCGTTAAAAATCGCCCGGTGTTGCACTTGG/<br>CCCAAGCTTAAGAACAGCATCGTGCCCACCAGCGGG	
D3UTRcon-F/<br>D3UTRcon-R	ATGGCTCCCGCCAAGAAGG/<br>TACAGCCACCTAACCCGGG	
C1332F/<br>C1332R	CCCGAATTCTCTACGCCGCAGCCAGGGGG<br>CCCAAGCTTGAACCGTATCTCGGATCCC	Used for cloning of *XC1332.*
061S-iv-F/<br>061S-iv-R	**TAATACGACTCACTATAGGG**GACGCGAGGATGCGTGG/<br>GGCGATTTCACGTCC	Used for production of sRX061S RNA.
061L-iv-F/<br>061L-iv-R	**TAATACGACTCACTATAGGG**GACGCGAGGATGCGTGG/<br>GAAAAACGCCCGGCTGC	Used for production of sRX062L RNA
061LM-iv-F/<br><br>061LM-iv-R	**TAATACGACTCACTATAGGG**GACGCGAAAATGCGTGGCCGCAACCTAACTG<br>GACAAAATGTCTGGGAAATGGCTAAAAGCCGACAAGGACGTG/<br>GAAAAACGCCCGGCTGCACATTTAAGTGCAACACC	Used for production of sRX062LM RNA
1332ORF-iv-F/<br>1332ORF-iv-R	**TAATACGACTCACTATAGGG**ATGAAGTCATTTACCGTAG/<br>TTAACGCCGTCCCACC	Used for production of XC1332ORF RNA
1332Full-iv-F/<br>1332Full-iv-R	**TAATACGACTCACTATAGGG**GGCGATAGGATTGCTGCATGTGG/<br>GAAAAACGCCCGGCTGCACATCC	Used for production of the full-length *XC1332* mRNA
SRC181-iv-F/<br>SRC181-iv-R	**TAATACGACTCACTATAGGG**CCAGCCGACGCGGCTGCTG/<br>CACCTTCCGCACCGCCACC	Used for production of the SRC181 RNA
**DNA Oligos**		
P5-OligoF	**TAATACGACTCACTATAGGG**TAGGTTGCGGCCACGCATCCTCGCGTC	Used for preparation of the Northern probe P5.
P5-OligoR	GACGCGAGGATGCGTGGCCGCAACCTA	
PM-OligoF	**TAATACGACTCACTATAGGG**TCCTTGTCGGCTTCCAGCCATCCCCCAGAC	Used for preparation of the Northern probe PM.
PM-OligoR	GTCTGGGGGATGGCTGGAAGCCGACAAGGA	
P3-OligoF	**TAATACGACTCACTATAGGG**GAAAAACGCCCGGCTGCACATCCAAGTGCA	Used for preparation of the Northern probe P3.
P3-OligoR	TGCACTTGGATGTGCAGCCGGGCGTTTTTC	

*Underlined nucleotides indicate the added restriction sites. Green nucleotides refer to the T7 promoter sequence. All primers and oligos were designed according to the genomic sequence of *Xcc* strain 8004 (Qian *et al*., 2005).

REFERENCE

Qian, W., Jia, Y., Ren, S.X., He, Y.Q., Feng, J.X., Lu, L.F. *et al*. (2005) Comparative and functional genomic analyses of the pathogenicity of phytopathogen *Xanthomonas campestris* pv. *campestris*. *Genome Research*, 15, 757–767.
